# Synergistic Transition Metal and Hydrogen Bonding Phase-Transfer Catalysis Enables Enantioconvergent Allylic Fluorination with KF

**DOI:** 10.1021/jacs.6c00549

**Published:** 2026-03-25

**Authors:** Zian Wang, Claire Dooley, Zijun Chen, Gabija Poškaitė, Robert S. Paton, Guy C. Lloyd-Jones, Véronique Gouverneur

**Affiliations:** † Chemistry Research Laboratory, University of Oxford, Oxford OX1 3TA, U.K.; ‡ Department of Chemistry, 224023 Colorado State University, Fort Collins, Colorado 80528, United States; § School of Chemistry, 3124 University of Edinburgh, Edinburgh EH9 3FJ, U.K.

## Abstract

Synergistic catalysis whereby both the nucleophile and the electrophile can be simultaneously activated by two distinct catalysts can rescue otherwise unattainable chemical transformations, as well as create or improve catalytic enantioselectivity. Herein, we report the merging of transition metal and hydrogen bonding phase-transfer catalysis to allow for allyl bromides to undergo enantioselective fluorination with KF. Individually, these two catalytic strategies are ineffective. Beyond solving the issue of reactivity, this approach represents a new manifold for enantiocontrol with a chiral ion pair composed of a metal-activated electrophilic substrate and a chiral hydrogen-bonded nucleophile. This study offers fresh opportunities for designing catalytic fluorination with high reactivity and enantioselectivity across diverse metal complexes and KF.

## Graphical abstract

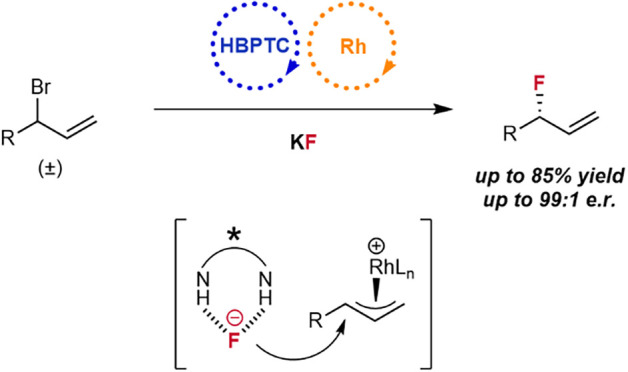

## Introduction

Chiral fluorinated molecules are in demand in modern medicinal chemistry^1^ because molecular three*-*dimensionality is becoming increasingly important in lead optimization, and fluorine substitution controls parameters such as metabolic stability and lipophilicity.^2^ For synthesis, most studies on catalytic enantioselective carbon–fluorine bond construction make use of an electrophilic fluorine reagent.^3^ Asymmetric catalytic nucleophilic fluorinations have developed more slowly because the high basicity of the fluoride ion could out-compete its nucleophilicity,^4^ and the small size of fluoride makes it particularly challenging for stereodifferentiation of prochiral electrophiles.

An elegant approach toward asymmetric nucleophilic fluorination is enabled by transition metal catalysis with a soluble and highly reactive amine-HF complex or AgF as a fluorine source (Figure 1a). Building on pioneering work by Haufe,^5,6^ Doyle and co-workers reported the asymmetric ring-opening hydrofluorination of epoxides^7^ and aziridines^8^ using a chiral Co-salen catalyst with controlled *in situ* formation of HF (Figure 1a­(i)). Mechanistic investigations revealed a fluorination step involving a Co-bifluoride complex as the active fluorinating species.^9^ The groups of Doyle,^10,11^ Lautens,^12^ and Nguyen^13–15^ subsequently developed methods for asymmetric allylic fluorination from substrates bearing various leaving groups (Figure 1a­(ii)). The mechanism commonly initiates with oxidative addition to form a metal-allyl complex, which undergoes rapid π–σ–π isomerization. A chiral π-acceptor ligand with a large bite angle favors either configuration of the complex, selectively leading to one regio- and enantiomer of the product upon outer-sphere attack of the fluoride.^16,17^ Complementing transition metal catalysis, Gouverneur and co-workers developed an organocatalytic approach for asymmetric fluorination by directly harnessing inexpensive but poorly soluble alkali metal fluoride salts. This activation mode, termed hydrogen bonding phase-transfer catalysis (HBPTC), employs a chiral *bis*-urea hydrogen bond donor (HBD) catalyst to solubilize KF or CsF in organic solvents^18^ (Figure 1b). Transient ion pairing with *meso* electrophiles including episulfonium ions,^19^ aziridinium ions^20^ (both formed *in situ*), and azetidinium salts^21^ successfully enabled enantioselective desymmetrizing ring-opening reactions by fluoride (Figure 1b­(i)). More recently, the group achieved enantioconvergent fluorination of neutral electrophiles including benzylic bromides and α-haloketones^22^ by engaging a second onium salt catalyst, which fulfills the key ion-pairing interactions for fluoride solubilization (Figure 1b­(ii)). While HBPTC allows inexpensive metal fluoride to be used as a fluorine source, hydrogen bonding intrinsically lowers the nucleophilicity of fluoride,^23^ thus limiting the technology to highly activated substrates.

**Figure 1:**
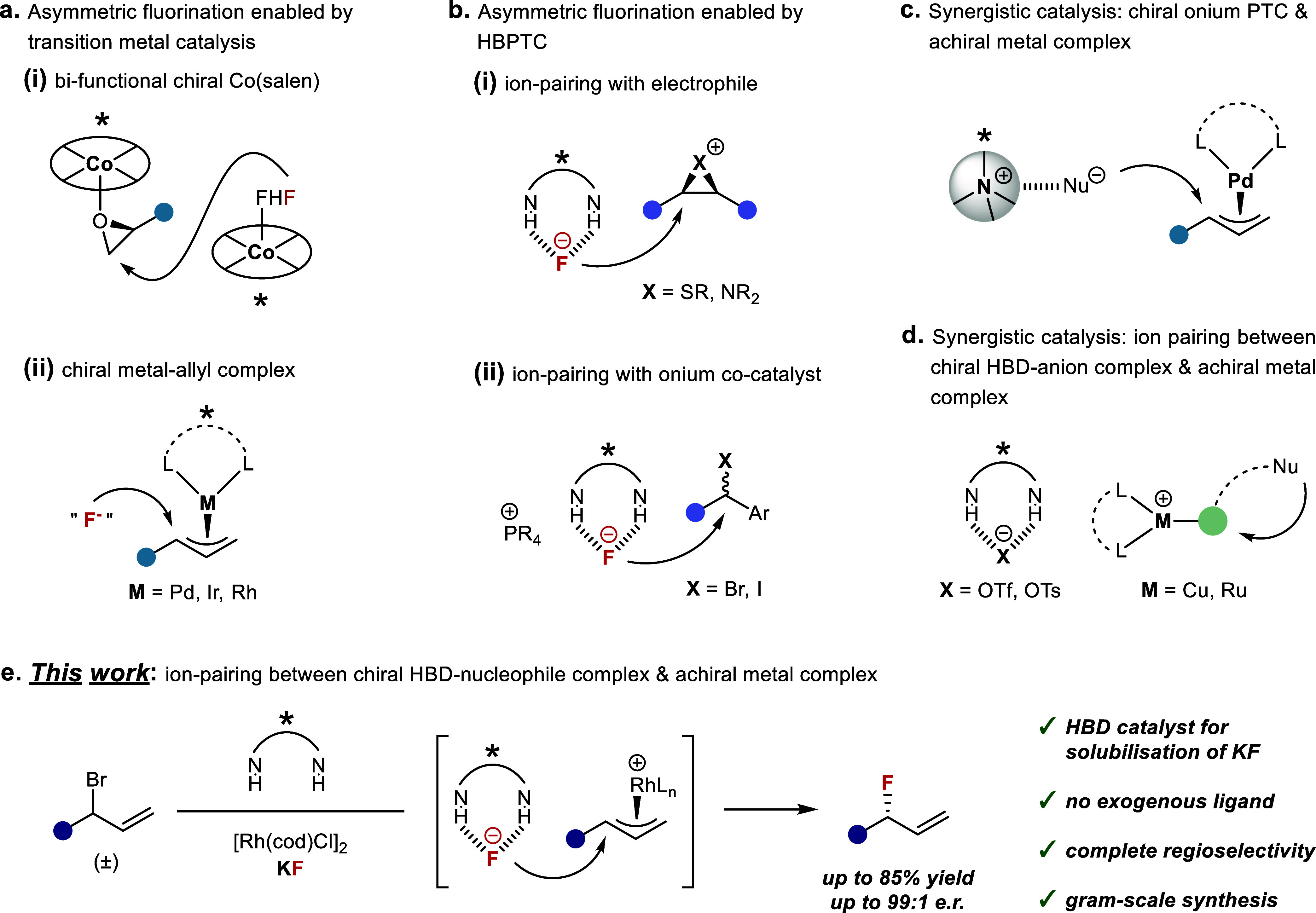
(a) Asymmetric nucleophilic fluorination enabled by transition metal catalysis. (b) Asymmetric nucleophilic fluorination enabled by hydrogen-bonding phase-transfer catalysis (HBPTC). (c) Asymmetric catalysis combining a chiral cationic PTC and an achiral transition metal complex. (d) Asymmetric catalysis combining a chiral hydrogen bond donor (HBD) catalyst and an achiral cationic transition metal complex. (e) This work: asymmetric allylic fluorination with KF via synergistic transition-metal catalysis and HBPTC.

Our goal was to invent a new catalytic manifold by merging transition metal catalysis and HBPTC to expand the range of electrophiles amenable to enantioselective fluorination with KF. Proof-of-concept studies focused on allylic halides because the enantioselective catalytic fluorination of these substrates is not known with inexpensive KF, and HBPTC was not satisfactory when used as the sole catalytic manifold.

At the onset of this study, we were aware that Gong,^24^ Takemoto,^25,26^ and Chen^27^ reported chiral quaternary ammonium or phosphonium phase-transfer catalysts (PTCs) in palladium-catalyzed allylic alkylation, whereby the cationic PTC forms an ion pair with an enolate nucleophile for subsequent addition to the metal-activated substrate (Figure 1c). We also noted the work of Mattson, featuring a chiral silanediol cocatalyst in copper­(II) triflate-catalyzed enantioselective conjugate addition of indoles to alkylidene malonates,^28^ and a seminal study by Jacobsen on the synergistic effect of a chiral *bis*-thiourea catalyst in ruthenium-catalyzed intramolecular propargylic substitution^29^ (Figure 1d). In these transformations, the HBD catalyst coordinates a spectator anion of the metal catalyst precursor, enhancing the electrophilicity of the metal-bound substrate while creating a chiral environment for nucleophile approach. More recently, this concept was extended to the asymmetric nucleophilic addition to Au­(I) π-complexes.^30,31^

Building on these precedents, enantioselective fluorination under synergistic HBPTC and metal catalysis would represent a new catalytic manifold by featuring a chiral hydrogen-bonded nucleophilic fluoride ion-paired with a cationic metal-substrate complex. However, this is not without challenges because *bis*-urea catalysts contain Lewis-basic sites that could deactivate the transition metal. The compatibility between hydrogen-bonded fluoride and the Lewis acidic metal center is not well understood, and transition metal-catalyzed fluorination reactions can involve a metal-fluoride complex.^17,32^ Herein, we report the feasibility of such a synergistic catalytic manifold with the enantioconvergent fluorination of allyl bromides with KF (Figure 1e). The method does not require an exogeneous enantiopure metal-chelating ligand, exhibits complete regioselectivity, and is amenable to gram-scale synthesis. Considering the vast opportunities in alkene functionalization,^33^ this asymmetric allylic fluorination process opens up a wide chemical space of enantioenriched fluorochemicals accessible from KF.

## Results and Discussion

### Reaction Development

Preliminary investigations were carried out on the model substrate *rac*-**1a**, which was selected for facile identification of products by ^19^F NMR. Applying the synergistic HBPTC manifold with the *bis*-urea catalyst (*S*)-**3a** and a cocatalytic phosphonium salt^22^ returned the starting material (Table 1, entry 1), demonstrating that *rac*-**1a** is insufficiently reactive for the hydrogen-bonded fluoride. Screening of transition metals combined with (*S*)-**3a** revealed that Pd_2_(dba)_3_ was unreactive, but both [Ir­(cod)­Cl]_2_ and [Rh­(cod)­Cl]_2_ complexes afforded the desired product **2a** with variable levels of selectivity (Table 1, entries 2–4). Analysis of the crude NMR and isolation of side-products confirmed the formation of linear allyl bromide **4a** and elimination product **5a** besides branched allyl fluoride **2a**. The linear allyl fluoride was not detected. The superior performance of [Rh­(cod)­Cl]_2_ (75% yield, 73:27 e.r.) is uncommon in allylic substitution.^13,34^ Carrying out the reaction with the rhodium catalyst in the absence of (*S*)-**3a** resulted in no fluorination but 26% elimination to **5a** (Figure S17d). The *bis*-urea catalyst was optimized next. Varying the *N*-alkyl group positioned closer to the binaphthyl scaffold had little influence on the reaction outcome (Table S4, entries 18–20); however, removing the alkyl group, as in (*S*)-**3d**, unexpectedly^19–22,35^ improved the e.r. to 89:11 (Table 1, entry 5). Variation of the aryl groups distal to the BINAM established that more electron-withdrawing substituents provided higher yield and enantioselectivity^36^ (Table 1, entries 5–8). The effect of added metal ligand was investigated next in control experiments. Inclusion of achiral phosphine ligands (Table 1, entry 9 and Table S4, entries 8–17) resulted in a slight improvement in yield. More interestingly, common chiral ligands for rhodium (Table 1, entry 10 and Table S4, entries 1–7) failed to enhance the enantioselectivity, and the combination of achiral Schreiner’s urea (**SU**) with chiral ligand **L1** yielded **2a** in racemic form (Table 1, entry 11). These results demonstrate that stereocontrol is solely derived from the organocatalyst (*S*)-**3d** without the requirement for exogenous metal ligands. Further optimization confirmed that DCM was the ideal solvent (Table S5), and lowering the reaction temperature was beneficial for both yield and e.r. (Table S6). The final optimized conditions afforded **2a** in 83% NMR yield and 97:3 e.r., and completely circumvented elimination (**5a**) (Table 1, entry 12).

**Table 1:**
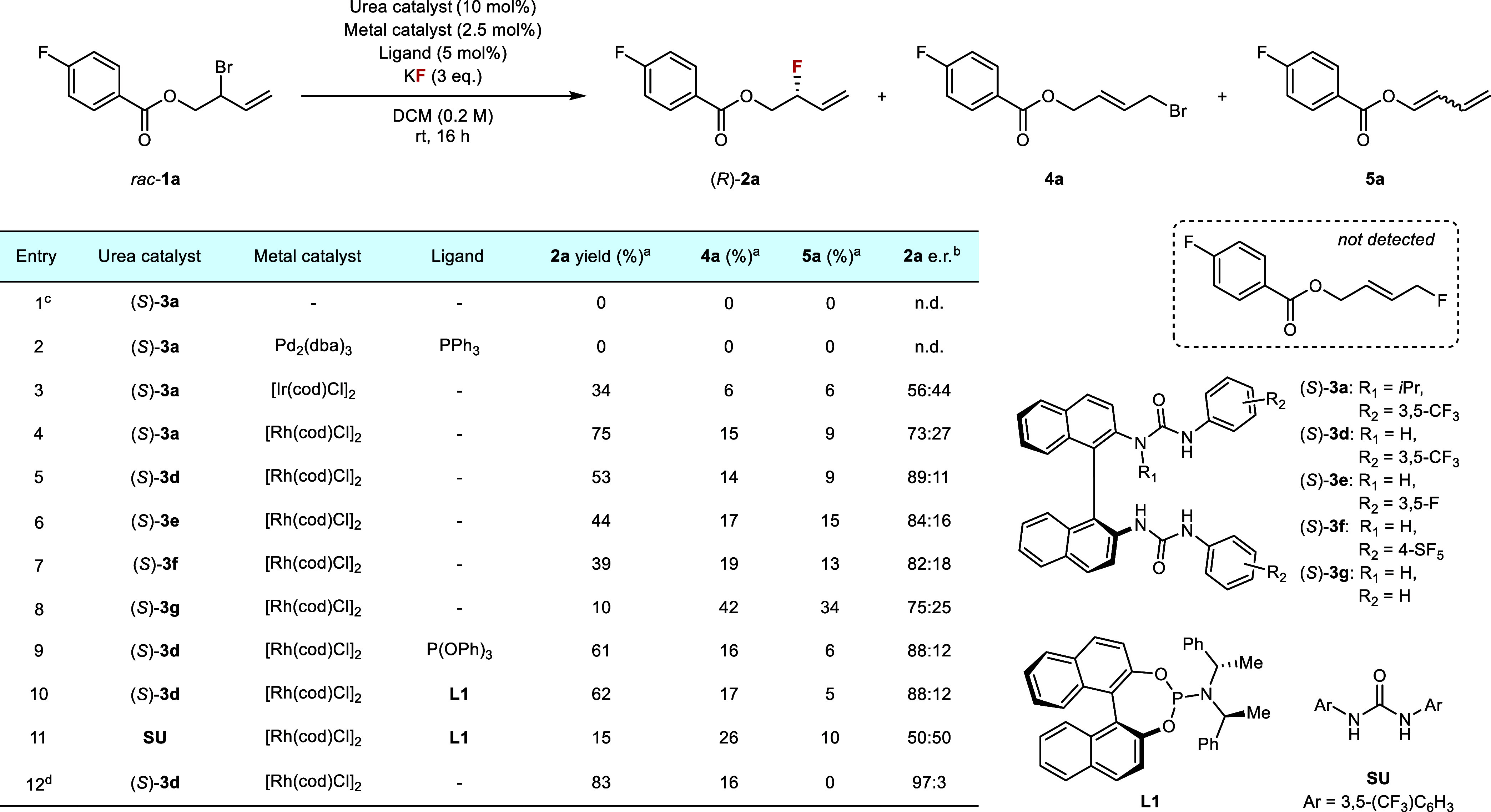
Reaction Optimization

a Yield determined by 19F NMR using 4-fluoroanisole as an internal standard.

b Enantiomeric ratio determined by HPLC analysis using a chiral stationary phase.

c With PPh_4_I (10 mol%) in *p*-xylene, 60 °C, 24 h

d Reaction performed at −30 °C for 72 h. n.d. = not determined.

### Substrate Scope

With the optimized conditions in hand, we examined the scope of the transformation (Figure 2). Substrates with a metal-chelating group in close proximity to the allyl motif reacted with the highest selectivity (>95:5 e.r.), including carboxylic esters (**2a**–**2f**, **2h**), ethers (**2i**, **2j**, **2o**), sulfonate esters (**2l**, **2m**), phosphinate esters (**2n**), and carbamates (**2p**). For benzoate esters **2a**–**2d**, selectivity trends were similar, but the reactivity was strongly dependent on the electronics of the aromatic ring. As the *para*-substituent varied from electron-withdrawing fluoro (**2a**) and cyano groups (**2b**), to hydrogen (**2c**), and to electron-donating alkyl groups (**2d**), the yield decreased substantially, with the corresponding linear allyl bromide being the main side-product. As the chelating group moved further away from the allyl motif, enantiocontrol was diminished (**2g**, **2k**, **2q**). Thioethers reacted poorly (**2r**), possibly due to metal catalyst inhibition.^37^ The beneficial effect of chelation on enantioselectivity is not unprecedented in transition metal-catalyzed allylic functionalization. In the rhodium-catalyzed allylic amination developed by Nguyen,^38^ a β-oxygen substituent was essential for high regio- and enantioselectivity. Recent work by Fletcher on enantioconvergent allylic arylation^39^ also demonstrated the importance of a suitably positioned carbonyl group for high enantiomeric excess.

**Figure 2:**
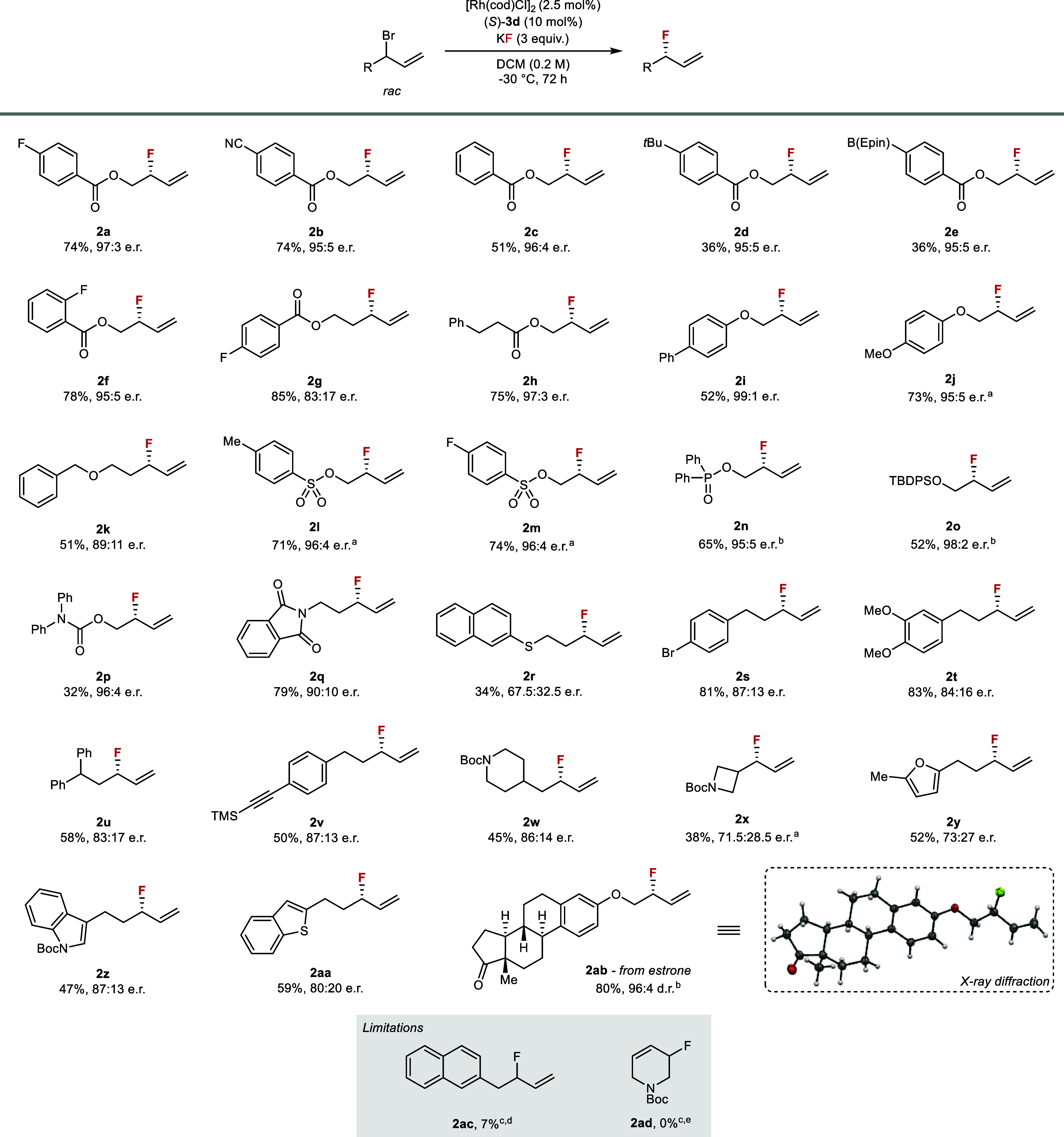
Substrate scope. Isolated product yields reported. Absolute configuration of **2ab** assigned by X-ray diffraction analysis and other products by analogy. ^a^Reaction performed at r.t. for 24 h. ^b^Reaction performed at 0 °C for 72 h. ^c^Yield determined by ^19^F NMR using 4-fluoroanisole as internal standard. ^d^Major product obtained from elimination. ^e^Starting material recovered. Epin = 1,1,2,2-tetraethylethylene glycol.

The generality of the reaction was demonstrated on substrates without an adjacent directing group, making steric differentiation more challenging (**2s**–**2w**, *ca*. 85:15 e.r.). The electronics of the distal phenyl ring had little influence on selectivity (**2s**, **2t**). A significant advantage of our protocol is that the mild fluorination conditions are compatible with fluorophilic boronic esters (**2e**) and silyl protecting groups (**2o**, **2v**). Common heteroaromatic rings were well tolerated, including furan (**2y**), indole (**2z**), and benzothiophene (**2aa**). Substrates with α-branching next to the allylic position (**2x**) were less reactive, presumably due to greater steric crowding. The estrone derivative **2ab** was obtained in 80% yield and high selectivity (96:4 d.r.), exemplifying the utility of this protocol on more complex scaffolds. It was noted that homobenzylic (**2ac**) and cyclic allylic substrates (**2ad**) were unsuitable for the reaction.

To further demonstrate the synthetic applications of this transformation, we carried out large-scale fluorination of *rac*-**1l** (Figure 3, left). The reaction was performed on 7 mmol of the substrate in a round-bottom flask at room temperature for 48 h. Over a gram of the allylic fluoride (*R*)-**2l** was obtained in 63% yield with 97:3 e.r., and 94% of the HBD catalyst (*S*)-**3d** was recovered. The catalyst was reused without loss of efficiency in terms of yield and selectivity. This reaction has several important advantages. It is simple and convenient to set up; it does not require anhydrous solvents; it can be conducted without exclusion of air; and the catalyst (*S*)-**3d** can be easily prepared in a single step from commercially available compounds.^40^ The enantioenriched product (*R*)-**2l** contains two versatile synthetic handles, an alkyl tosylate and a terminal alkene, both of which are amenable to further derivatization (Figure 3, right). The tosylate was replaced by a nitrogen nucleophile^41^ to afford the β-fluoroamine **7**. Substitution by a stabilized carbon nucleophile^42^ led to **2u′**, which provided an alternative to direct fluorination from the corresponding allyl bromide, but with a much higher enantioselectivity (*cf*. Figure 2, **2u**). We also carried out well-established alkene functionalization reactions, including hydroboration/oxidation^11^ to obtain γ-fluoroalcohol **8** and cross-metathesis with a styrene^14^ to obtain the nonterminal allylic fluoride **9**. The fluorinated stereocenter remained intact when subjected to all the derivatization procedures, demonstrating the value of **2l** as a versatile chiral fluorinated building block.

**Figure 3:**
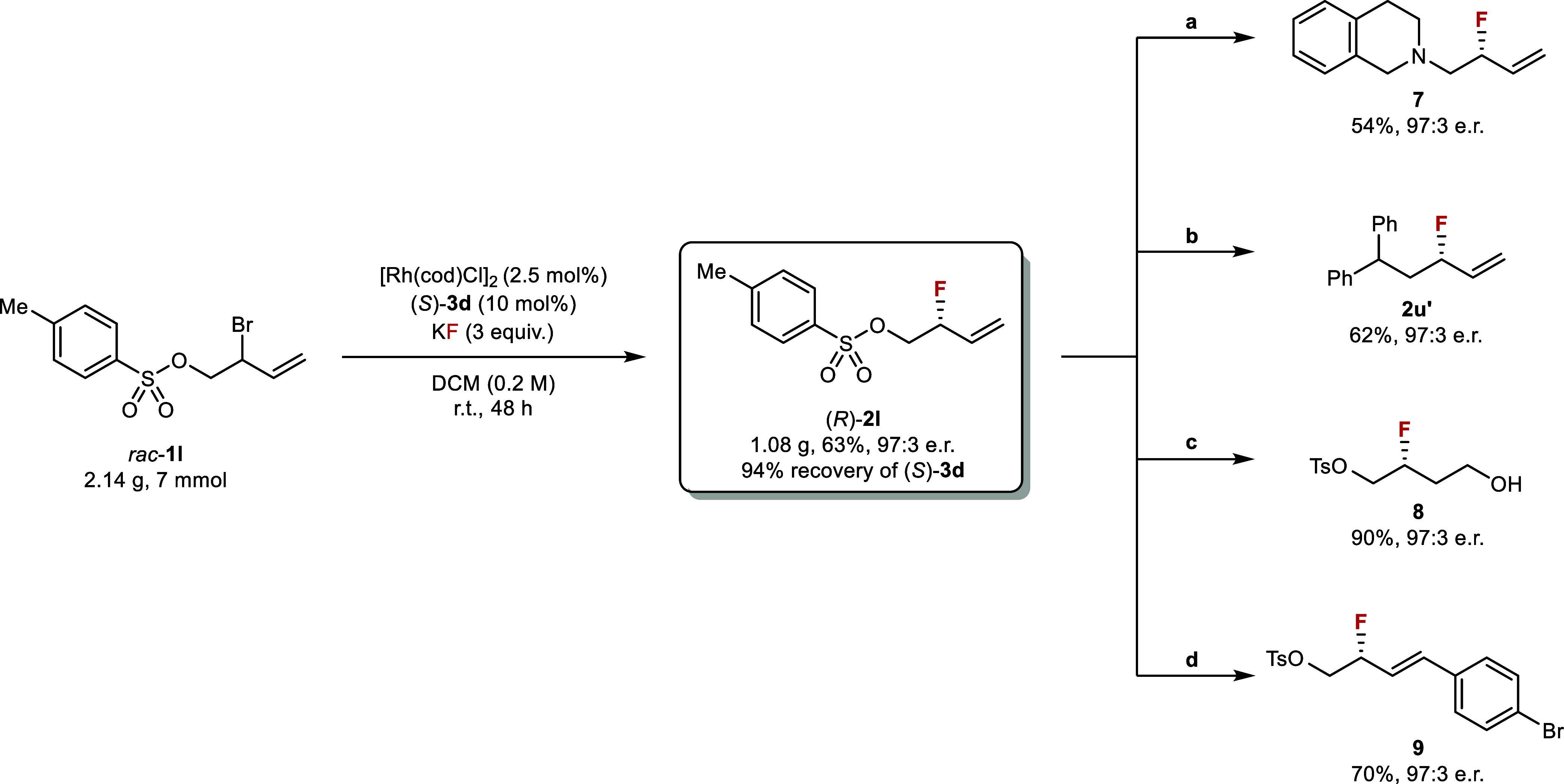
Gram-scale synthesis of (*R*)-**2l** and further product derivatization. Reagents and conditions: (a) 1,2,3,4-tetrahydroisoquinoline, K_2_CO_3_, DMF, 100 °C; (b) diphenylmethane, *n*BuLi, TMEDA, THF, 15 to 40 °C; (c) 9-BBN, THF, r.t. to 40 °C, then aq. NaOH, H_2_O_2_, r.t.; (d) Hoveyda-Grubbs II, 4-bromostyrene, DCM, 60 °C.

### Mechanistic Investigations

Initial mechanistic studies focused on probing the origin of enantioconvergence of the transformation. Subjecting the enantiopure substrate (*R*)-**1a** to the standard conditions led to essentially the same reaction outcome as from *rac*-**1a** (Figure 4a, right). The rhodium catalyst completely racemized (*R*)-**1a**, whereas urea (*S*)-**3d** was inactive (Figure 4a, left). *Ex situ* monitoring showed that **1a** remained racemic throughout the reaction, and the e.e. of **2a** remained high and constant (Figure 4d). These observations point to a dynamic kinetic asymmetric transformation (DYKAT) mechanism, in which a putative rhodium-allyl complex isomerizes between two prochiral faces at a rate faster than fluorination. According to the model proposed by Evans,^43^ this key intermediate is more appropriately described as a rhodium-enyl complex. Rapid isomerization between two regioisomeric enyl species prior to nucleophilic attack is crucial for high branch-selectivity of the overall substitution reaction.

**Figure 4:**
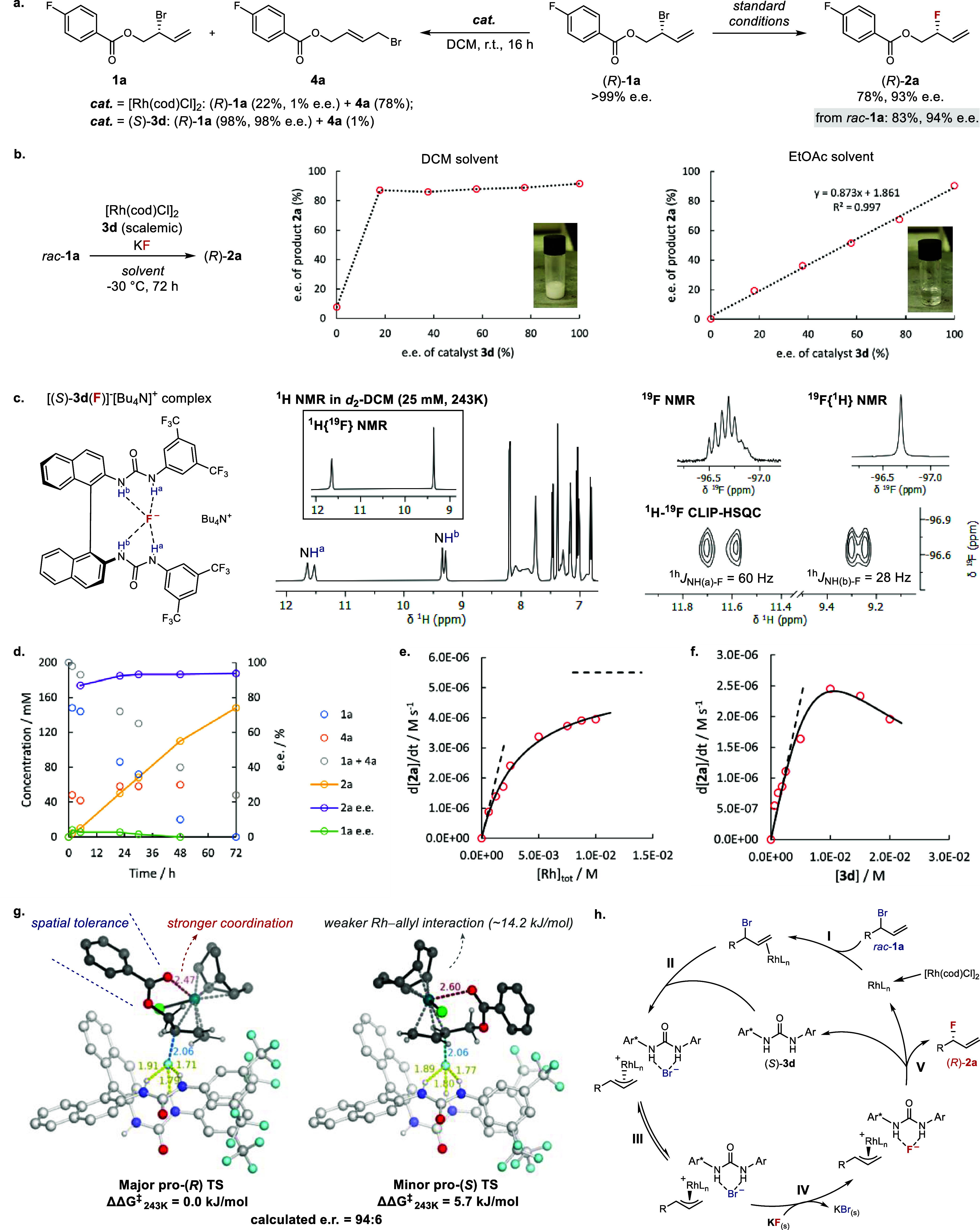
Mechanistic investigations. (a) Control experiments on the enantiopure substrate (*R*)-**1a**. Standard conditions refer to Table 1, entry 12. (b) Nonlinear effect study in DCM and EtOAc, respectively. (c) Spectroscopic evidence for the tetradentate hydrogen bonding between (*S*)-**3d** and fluoride. (d) Evolution of the yield and e.e. (if applicable) of **1a**, **2a**, and **4a** in the model reaction over time through *ex situ* monitoring. (e) Rate of product **2a** formation at varying rhodium concentrations. [**1a**]_0_ = 200 mM, [(*S*)-**3d**] = 20 mM. The black solid line shows fitting to the model in Figure S29. (f) Rate of product **2a** formation at varying concentrations of urea (*S*)-**3d**. [1a]_0_ = 200 mM, [Rh]_tot_ = 10 mM. The black solid line shows fitting to model in Figure S32. (g) Computed major and minor enantio-determining transition structures (ωB97X-D3/(ma)-def2-TZVPP-def2-ECP­(Rh)-CPCM­(DCM)//M06–2X/def2-SVP­(TZVPPD)-SDD­(Rh)-CPCM­(DCM)). (h) Proposed catalytic cycle.

The synergistic action of rhodium and urea catalysts in C–Br bond breaking was studied indirectly by tracking the formation of linear bromide side-product **4a**. The rhodium catalyst led to 78% isomerization of **1a** to **4a** (Figure 4a, left), while 85% conversion to **4a** was observed when 10 mol% of urea (*S*)-**3d** was added (Figure S17c). No isomerization occurred in the absence of rhodium (Figure S17a). Continuous *in situ* monitoring^44^ showed rhodium-catalyzed isomerization taking place, which is consistent with an S_N_2-type (or S_N_2′) displacement of bromide by Rh­(I), a process only slightly more effective in the presence of (*S*)-**3d** (Figure S23). Control reactions employing **4a** as the substrate under standard fluorination conditions demonstrated reduced reactivity compared to **1a** (Figure S19b), explaining the persistence of **4a** throughout the reaction (Figures 4d and S22).

The role of (*S*)-**3d** in enantioinduction was investigated next. Following the e.e. of **2a** when using **3d** with varying levels of enantiopurity resulted in a nonlinear correlation in DCM, whereas a linear relationship was observed in EtOAc (Figure 4b). This was attributed to the poor solubility of *rac*-**3d** in DCM compared to EtOAc (Figure 4b inset, cloudy mixture in DCM vs clear solution in EtOAc), allowing chiral amplification by selective precipitation of the racemate.^45,46^ The use of non-racemic, enantiomerically impure catalyst (*S*)-**3d** in DCM immediately led to the precipitation of *rac*-**3d**, with the catalyst remaining in solution becoming highly enriched in the *S*-configuration (Figure S3). Therefore, the e.e. of product **2a** obtained will approach that observed when enantiopure (*S*)-**3d** was used. Assuming the mode of enantioinduction is solvent-independent, the observations from experiments carried out in EtOAc support a single catalyst molecule being involved in the enantio-determining step.

To gain further insights into the nature of catalyst-bound fluoride, an NMR study was performed on a heterogeneous mixture of (*S*)-**3d**, KF and tetrabutylammonium tetrafluoroborate at 243 K in *d*_2_-DCM. The ammonium salt was necessary as the solubilization of KF by *bis*-urea catalysts was not observed in the absence of an auxiliary phase-transfer reagent.^22^ This resulted in a dominant species characterized as the [(*S*)-**3d**(F)]^−^[Bu_4_N]^+^ complex (Figure 4c, see Supporting Information for full characterization data and assignment of NMR signals). Tetradentate hydrogen bonding to fluoride was indicated by the presence of characteristic deshielded doublets in ^1^H NMR at 11.59 ppm (^1h^ *J*_HF_ = 60 Hz) and 9.32 ppm (^1h^ *J*_HF_ = 28 Hz) and by the corresponding fluoride signal in ^19^F NMR at −96.7 ppm, exhibiting hydrogen-bond scalar couplings to all four NH groups.^35^ The one-bond H···F coupling constants were confirmed by ^1^H–^19^F CLIP-HSQC.^47^ This extensive hydrogen bonding attenuates the reactivity of fluoride to favor nucleophilic attack over elimination.^48^

Kinetic analysis was performed by systematically varying the KF agitation and mass transfer, and the concentrations of bromide **1a** and both catalysts. An *ex situ* NMR sampling method was adopted considering the heterogeneous nature of the reaction where phase transfer of KF was nontrivial.^44^ For all experiments, the product concentration [**2a**] grows linearly with time (Figures S24, S28, and S31). This result indicates independence or kinetic saturation in the bromide substrate **1a** concentration. It also allows convenient estimation of the overall rate directly from the gradient of the temporal concentration profile of **2a**, and thus ready evaluation of the influence of the other experimental parameters. The effect of mass transfer was investigated by changing the stirring speed within the same reaction such that the experimental setup was intrinsically consistent. The rate approximately doubled when the stirring speed was increased from 600 to 1200 rpm (Figure S27), indicating that the reaction is mass transfer-limited under standard conditions. The concentrations of [Rh­(cod)­Cl]_2_ and (*S*)-**3d** were then independently varied to investigate their influence on the rate of generation of **2a**. With increasing [Rh]_tot_ concentrations, the rate increased with a nonlinear proportion, and within the 0 to 10 mM range explored, the data were consistent with either an off-cycle dimerization, e.g., as a μ-halide dimer^49^ (Figure S30), or catalyst saturation kinetics.^50^ The latter gave a better overall correlation (Figure 4e). As the urea concentration was increased, the rate was again nonlinear, first increasing and then decreasing when [**3d**] exceeded 10 mM, suggesting an inhibition pathway at high urea concentration^50^ (Figure 4f). Both catalysts approach first-order dependencies in the low concentration limit when phase transfer is presumably not rate-limiting. Overall, the data suggest that monomeric Rh and a single urea molecule are the catalytically active species. Several possible origins for the inhibitory effect of (*S*)-**3d** were considered, including coordination to the [(*S*)-**3d**(F)]^−^[Bu_4_N]^+^ complex,^36^ or coordination to rhodium in competition with substrate binding. While it has been reported that urea could act as either an *N*- or *O*-donor ligand for transition metals^51^ including Rh­(I),^52,53^ our attempts to spectroscopically observe Rh­(I):(*S*)-**3d** complex were unsuccessful. Adding [Rh­(cod)­Cl]_2_ to a solution of (*S*)-**3d** in *d*_2_-DCM did not result in substantial changes in chemical shifts in ^1^H, ^13^C or ^19^F NMR (Figures S14–S16). Moreover, the rate of catalysis remains independent of the substrate concentration at the highest urea concentrations explored. Taken together, these observations suggest that competitive binding at rhodium is unlikely.

Density functional theory (DFT) calculations were performed to analyze the enantio-determining transition structures (TSs) between the Rh­(III)-allyl complex from model substrate **1c** and (*S*)-**3d**-bound fluoride (Figure 4g). The allyl substituents of the Rh­(III) complex are oriented away from the catalytic pocket. This spatial arrangement accounts for the broad substituent tolerance observed at this position. The computed selectivity of 94:6 e.r. at 243 K (ΔΔ*G* ^‡^ = 5.7 kJ/mol), favoring (*R*)-product formation, aligned well with experimental results. Further Distortion-Interaction/Activation-Strain analysis revealed that the primary contribution to the origin of enantioselectivity arises from the weaker interaction of the Rh­(III)-allyl complex in the minor pro-(*S*) transition state (ΔΔ*E* ^‡^ = 14.2 kJ/mol), where the carbonyl­(O)–Rh distance was elongated by 0.13 Å, reducing the chelating interaction^54^ (Tables S16–S18).

Based on the above data and a computed reaction pathway using Schreiner’s urea as the catalyst (Figure S36), a dual catalytic cycle was proposed (Figure 4h). Alkene coordination to the dissociated monomeric rhodium catalyst (**I**) occurs, potentially with chelation from an appropriate R group in the substrate (Δ*G* = 31.9 kJ/mol). Oxidative addition (**II**) gives an ion pair between the Rh­(III)-allyl complex and hydrogen-bonded bromide (Δ*G* ^‡^ = 73.3 kJ/mol and Δ*G* = 29.1 kJ/mol). Rapid π–σ–π isomerization takes place at the rhodium center (**III**), eliminating chiral information from the bromide substrate. Halide exchange with KF in the solid phase (**IV**, Δ*G* = 7.6 kJ/mol) leads to the reactive ion pair with urea-bound fluoride (Δ*G* = −17.3 kJ/mol). At any stage between **III** and **IV**, the chiral urea catalyst imposes a preference for one configuration of the rhodium-allyl complex, followed by outer-sphere attack at the branched position, yielding (*R*)-**2a** irreversibly and stereoselectively (**IV**), and regenerating both (*S*)-**3d** and the Rh­(I) catalyst (Δ*G* ^‡^ = 41.1 kJ/mol and Δ*G* = −103.0 kJ/mol). Formation of the strong C–F bond provides the thermodynamic driving force for the overall transformation.

## Conclusion

In summary, we have developed a novel synergistic catalytic manifold that enables the highly enantioselective allylic fluorination of racemic allyl bromides with KF. The rhodium­(I) catalyst provides a rapidly racemizing electrophilic π-allyl intermediate, allowing for enantioconvergence. Catalyst (*S*)-**3d** solubilizes fluoride in a well-characterized, 4-fold hydrogen bonding complex, which tunes fluoride reactivity and contributes to the high level of regio- and enantiocontrol. We anticipate that this strategy of synergistic catalysis can be extended to more classes of substrates intractable to HBPTC or transition-metal catalysis alone.

## Supplementary Material



## Notes

Materials and methods, optimization studies, mechanistic studies, ^1^H, ^13^C, ^19^F NMR spectra, and high-resolution mass spectrometry, infrared, and chiral HPLC data (PDF)
