# Supplementary material for: Synergistic Transition Metal and Hydrogen Bonding Phase-Transfer Catalysis Enables Enantioconvergent Allylic Fluorination with KF
Source: J Am Chem Soc. 2026 Mar 25;148(13):14213–22. doi: 10.1021/jacs.6c00549 (PMC13067345; doi:10.1021/jacs.6c00549)
Supplement: Supplementary file 1 [file ja6c00549_si_001.pdf]

Supplementary Information

Synergistic Transition Metal and Hydrogen Bonding Phase-Transfer Catalysis Enables Enantioconvergent Allylic Fluorination with KF

Zian Wang[1], Claire Dooley[1], Zijun Chen[1], Gabija Poškaitė[1], Robert S. Paton[2], Guy C. Lloyd-Jones[3]*, Véronique Gouverneur[1]*

Affiliation: [1]Chemistry Research Laboratory, University of Oxford, Oxford, OX1 3TA, UK; [2]Department of Chemistry, Colorado State University, Fort Collins, CO 80528, USA; [3]School of Chemistry, University of Edinburgh, Edinburgh, EH9 3FJ, UK.

Table of Contents

General information **2**
Reaction Optimisation **3**
Urea Catalyst Synthesis and Characterisation **10**
Substrate Synthesis and Characterisation **13**
General Procedure for Enantioconvergent Allylic Fluorination **33**
Product Characterisation **34**
Procedures for Product Derivatisation **45**
Non-Linear Effect Study **47**
NMR Investigations **50**
Control Reactions **60**
Monitoring Product Distribution and Enantiomeric Ratios over Time **62**
Kinetic Experiments **63**
X-ray Crystallographic Data **74**
Computational Details **76**
NMR Spectra **107**
HPLC Traces **216**
References **247**

General information

Unless stated, reagents were purchased from commercial suppliers and used without further purification. Unless stated, solvents were used without drying or degassing. Unless stated, all reactions were performed in air without exclusion of oxygen or moisture. All reactions that required anhydrous conditions were performed in flamed-dried glassware under an inert atmosphere of nitrogen and solvents from stills were used. CsF (99.9% trace metal basis from Sigma-Aldrich) was ground prior to reactions and used without pre-drying. KF (99.99% trace metal basis from Alfa Aesar) was ground prior to reactions and used without pre-drying. Allyl bromide substrates and allyl fluoride products were stored at -20 °C where they remained stable for several months. Reactions were monitored by thin layer chromatography (TLC) supplied by Merck (Kieselgel 60 F_{254} plates). Visualisation of reactions on TLC was accomplished by irradiation with UV light at 254 nm and/or cerium ammonium molybdate (CAM) stain and/or permanganate stain. Flash column chromatography (FCC) was performed on Merck silica gel (60, particle size 0.040-0.062 mm). Optical rotations were measured on an Autopol L 2000 (Schmidt-Haensh) at 589 nm, 25 °C. Data are reported as $[\alpha]_D^{25\,°C}$ concentration (c in g/100 mL), and solvent. High resolution mass spectra (HRMS, *m/z*) were recorded on a Thermo Exactive mass spectrometer equipped with Waters Acquity liquid chromatography system using the heated electrospray (HESI-II) probe for positive electrospray ionization (ESI^+) or atmospheric pressure chemical ionization (APCI) or on an Agilent 7200 Q-TOF spectrometer equipped with a direct insertion probe supplied by Scientific Instrument Manufacturer (SIM) GmbH using electron ionization (EI – 20eV). Some compounds were found to be unstable under a variety of MS ionization methods (CI, EI, ESI, GC-MS) and therefore no HRMS could be obtained for them; this is stated for the relevant compounds. Infrared spectra were recorded as the neat compound or in liquid solution using a Bruker tensor 27 FT-IR spectrometer, absorptions are reported in wavenumber (cm^{-1}). Melting points of solids were measured on a Griffin apparatus and are uncorrected. All enantiomeric ratios (e.r.) were determined by HPLC analysis on a Shimadzu *i*-Prominence LC-2030 (PDA detector), employing a chiral stationary phase, post purification and compared to traces of the racemic mixtures, which were independently prepared. All NMR spectra were recorded on Bruker AVIIIHD 400, AVIIIHD 500 or AVII 500. Deuterated solvents were purchased from Sigma-Aldrich and used without purification. d_2-DCM was stored at 4 °C over 3Å molecular sieves, under nitrogen atmosphere. NMR spectra are recorded at 298 K, unless otherwise specified. NMR spectra are referenced to the residual solvent peak for ^{1}H and ^{13}C spectra, while ^{19}F NMR spectra are referenced relative to $CFCl_3$ using the Bruker internal referencing procedure. Coupling constants, *J*, are reported in Hz to the nearest 0.1 Hz. Unless otherwise stated, ^{13}C and ^{31}P spectra are ^{1}H decoupled. NMR spectral data are reported as chemical shifts (δ) in parts per million (ppm) relative to the solvent peak. The following abbreviations are used to describe peak multiplicities: s = singlet, d = doublet, t = triplet, q = quartet, m = multiplet.

Reaction Optimisation

Table S1: Preliminary Optimisation

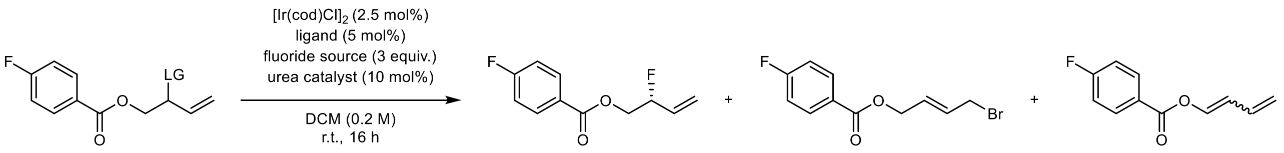

rac-**1a** (*R*)-**2a** **4a** **5a**

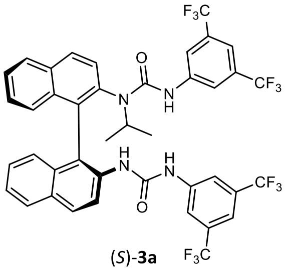

(*S*)-**3a**

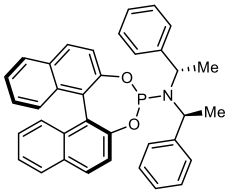

L1

Entry	LG	Urea catalyst	Fluoride source	Ligand	**2a** yield (%)[a]	**4a** (%)[a]	**5a** (%)[a]	e.r.[b]
1	Br	-	KF	**L1**	0	2	0	n.d.
2	Br	-	CsF	**L1**	6	8	4	n.d.
3	Br	-	TBAF·3H_2O	**L1**	22	4	42	57:43
4	Br	(*S*)-**3a**	KF	**L1**	17	2	1	65:35
5	Br	(*S*)-**3a**	KF	-	34	6	6	54:46
6	Br	(*S*)-**3a**	CsF	**L1**	68	15	6	57:43
7	Br	(*S*)-**3a**	CsF	-	88	4	5	51:49
8	Br	(*S*)-**3a**	TBAF·3H_2O	**L1**	22	4	46	56:44
9	Cl	(*S*)-**3a**	KF	**L1**	10	-	2	55:45
10	Cl	(*S*)-**3a**	CsF	**L1**	13	-	2	53:47
11	OC(=NH)CCl_3	(*S*)-**3a**	KF	**L1**	0	-	23	n.d.
12	OAc	(*S*)-**3a**	KF	**L1**	0	-	0	n.d.

General conditions: Substrate (0.1 mmol), urea catalyst (10 mol%), $[Ir(cod)Cl]_2$ (2.5 mol%), ligand **L1** (5 mol%) and fluoride source (3 equiv.) in 500 µL of DCM stirred at 1200 rpm for 16 h at room temperature. [a]Determined by ^{19}F NMR using 4-fluoroanisole as internal standard. [b]e.r. was determined by HPLC analysis using a chiral stationary phase. n.d. = not determined.

Table S2: Transition Metal Optimisation

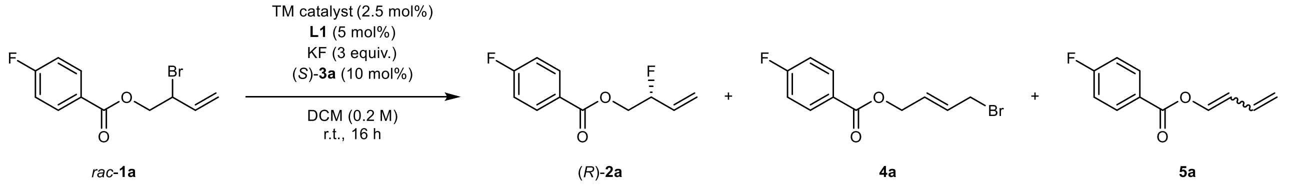

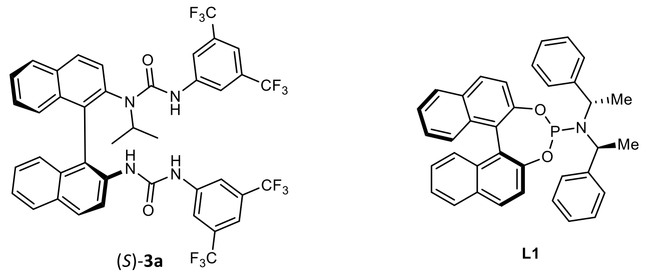

Entry	TM catalyst	Ligand	**2a** yield (%)[a]	**4a** (%)[a]	**5a** (%)[a]	e.r.[b]
1	$[Ir(cod)Cl]_2$	**L1**	17	2	1	65:35
2	$[Ir(coe)_2Cl]_2$	**L1**	4	10	0	58:42
3	$[Rh(cod)Cl]_2$	**L1**	65	14	5	72:28
4	$[Rh(coe)_2Cl]_2$	**L1**	2	21	0	n.d.
5	$Rh(PPh_3)_3Cl$	**L1**	0	0	8	n.d.
6	$[Rh(dppb)(cod)][BF_4]$	**L1**	0	0	0	n.d.
7	$Pd_2(dba)_3$	PPh_3	0	0	0	n.d.
8	$Pd(OAc)_2$ (5 mol%)	PPh_3	0	0	0	n.d.

General conditions: Substrate (0.1 mmol), urea catalyst (*S*)-**3a** (10 mol%), metal catalyst (2.5 mol%), ligand (5 mol%) and KF (3 equiv.) in 500 μL of DCM stirred at 1200 rpm for 16 h at room temperature. [a]Determined by ^{19}F NMR using 4-fluoroanisole as internal standard. [b]e.r. was determined by HPLC analysis using a chiral stationary phase. n.d. = not determined.

Table S3: Urea Catalyst Optimisation

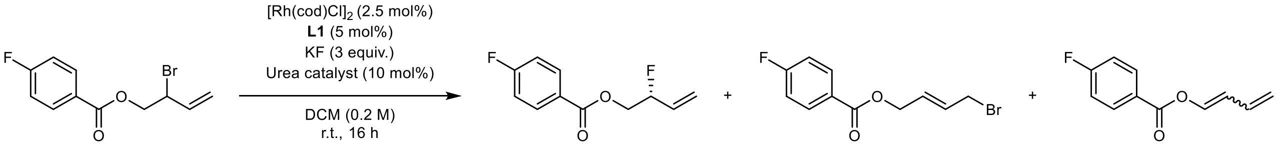

rac-**1a** (*R*)-**2a** **4a** **5a**

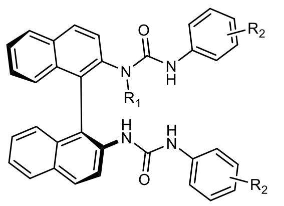

Urea catalyst

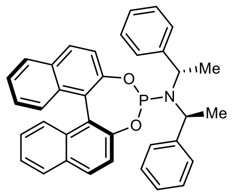

L1

Entry	Urea catalyst	R_1	R_2	**2a** yield (%)[a]	**4a** (%)[a]	**5a** (%)[a]	e.r.[b]
1	(*S*)-**3a**	*i*Pr	3,5-CF_3	65	14	5	72:28
2	(*S*)-**3b**	Et	3,5-CF_3	65	11	4	76:24
3	(*S*)-**3c**	Me	3,5-CF_3	77	14	6	77:23
4	(*S*)-**3d**	H	3,5-CF_3	62	17	5	88:12
5	(*S*)-**3e**	H	3,5-F	47	28	11	80:20
6	(*S*)-**3f**	H	4-SF_5	31	19	9	77:23
7	(*S*)-**3g**	H	H	10	43	19	73:27
8	(*S*)-**3h**	H	3,5-Me	0	6	0	n.d.
9	(*S*)-**3i**	H	2-CF_3	2	43	21	n.d.
10	(*S*)-**3j**	H	pentafluoro	10	49	7	60:40

General conditions: Substrate (0.1 mmol), urea catalyst (10 mol%), $[Rh(cod)Cl]_2$ (2.5 mol%), ligand **L1** (5 mol%) and KF (3 equiv.) in 500 μL of DCM stirred at 1200 rpm for 16 h at room temperature. [a]Determined by ^{19}F NMR using 4-fluoroanisole as internal standard. [b]e.r. was determined by HPLC analysis using a chiral stationary phase. n.d. = not determined.

Table S4: Ligand Optimisation

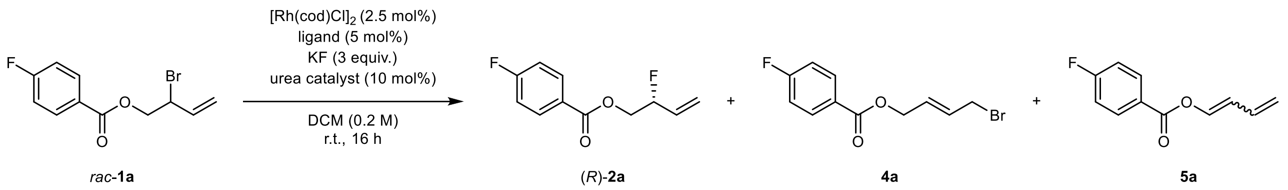

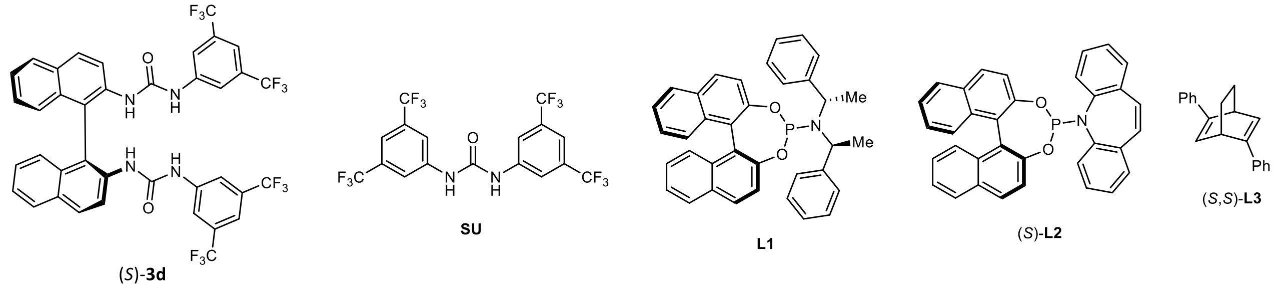

Entry	Ligand	Urea catalyst	**2a** yield (%)[a]	**4a** (%)[a]	**5a** (%)[a]	e.r.[b]
1	**L1**	(*S*)-**3d**	62	17	5	88:12
2	**L1**	**SU**	15	26	10	50:50
3	(*S*)-**L2**	(*S*)-**3d**	58	7	5	34:66
4	(*R*)-**L2**	(*S*)-**3d**	62	8	6	87:13
5	(*S*,*S*)-**L3**	(*S*)-**3d**	73	13	4	89:11
6	(*R*,*R*)-**L3**	(*S*)-**3d**	66	15	4	88:12
7	(*S*,*S*)-**L3**	**SU**	24	34	15	54:46
8	PPh_3	(*S*)-**3d**	57	12	11	67:33
9	P(1-nap)$_3$	(*S*)-**3d**	56	17	13	88:12
10	P(4-C_6H_4OMe)$_3$	(*S*)-**3d**	53	12	15	76:24
11	$P(C_6F_5)_3$	(*S*)-**3d**	27	51	1	90:10
12	P(OEt)$_3$	(*S*)-**3d**	22	52	5	72:28
13	P(OPh)$_3$	(*S*)-**3d**	61	16	6	88:12
14	$P(OCH_2CF_3)_3$	(*S*)-**3d**	25	37	2	90:10
15	*rac*-BINAP	(*S*)-**3d**	24	39	34	85:15
16	dppe	(*S*)-**3d**	60	19	13	88:12
17	Xantphos	(*S*)-**3d**	44	26	14	84:16
18	-	(*S*)-**3a**	75	15	9	73:27
19	-	(*S*)-**3b**	68	8	9	78:22
20	-	(*S*)-**3c**	68	8	10	80:20
21	-	(*S*)-**3d**	53	14	9	89:11
22[c]	-	(*S*)-**3d**	14	-	3	83:17

23	-	(*S*)-**3e**	44	17	15	84:16
24	-	(*S*)-**3f**	39	19	13	82:18
25	-	(*S*)-**3g**	10	42	34	75:25

General conditions: Substrate (0.1 mmol), urea catalyst (10 mol%), $[Rh(cod)Cl]_2$ (2.5 mol%), ligand (5 mol%) and KF (3 equiv.) in 500 µL of DCM stirred at 1200 rpm for 16 h at room temperature. [a]Determined by ^{19}F NMR using 4-fluoroanisole as internal standard. [b]e.r. was determined by HPLC analysis using a chiral stationary phase. [c]Using **S1** as the substrate (with chloride as leaving group). dppe = 1,2-bis(diphenylphosphino)ethane.

Table S5: Solvent and Concentration Optimisation

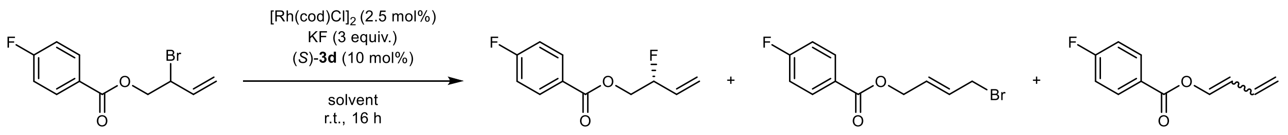

rac-**1a** (*R*)-**2a** **4a** **5a**

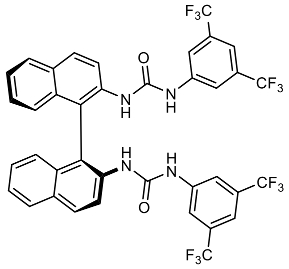

(*S*)-**3d**

Entry	Solvent	Concentration	**2a** yield (%)[a]	**4a** (%)[a]	**5a** (%)[a]	e.r.[b]
1	DCM	0.2 M	53	14	9	89:11
2	$CHCl_3$	0.2 M	18	54	24	85:15
3	toluene	0.2 M	49	26	12	84:46
4	1,2-DCE	0.2 M	57	16	4	90:10
5	1,2-DFB	0.2 M	33	22	0	90:10
6	THF	0.2 M	0	56	13	n.d.
7	MeCN	0.2 M	9	59	3	n.d.
8	EtOAc	0.2 M	23	47	16	89.5:10.5
9	acetone	0.2 M	14	62	7	84.5:15.5
10	DCM	0.1 M	17	47	1	90:10
11	DCM	0.4 M	30	43	0	90:10

General conditions: Substrate (0.1 mmol), urea catalyst (*S*)-**3d** (10 mol%), $[Rh(cod)Cl]_2$ (2.5 mol) and KF (3 equiv.) in a specific volume of solvent stirred at 1200 rpm for 16 h at room temperature. [a]Determined by ^{19}F NMR using 4-fluoroanisole as internal standard. [b]e.r. was determined by HPLC analysis using a chiral stationary phase. n.d. = not determined.

Table S6: Temperature Optimisation

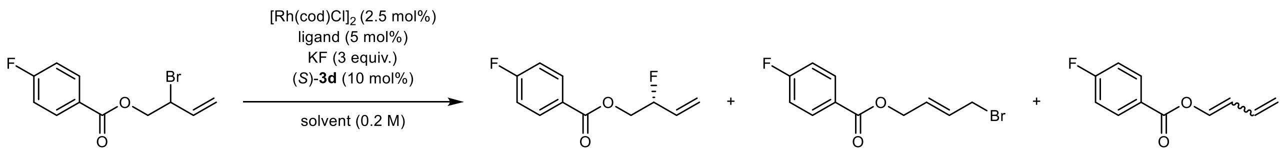

rac-**1a** (*R*)-**2a** **4a** **5a**

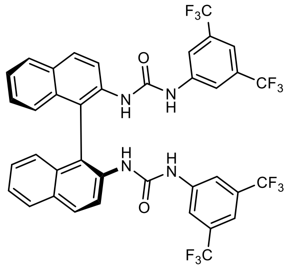

(*S*)-**3d**

Entry	Solvent	Temperature & time	Ligand	**2a** yield (%)[a]	**4a** (%)[a]	**5a** (%)[a]	e.r.[b]
1	DCM	r.t., 16 h	-	53	14	9	89:11
2[c]	DCM	r.t., 16 h	-	32	46	1	90:10
3	DCM	0 °C, 72 h	-	56	27	1	93:7
4	DCM	-15 °C, 72 h	-	65	27	1	95:5
5	DCM	-30 °C, 72 h	-	83	16	0	97:3
6	1,2-DCE	-25 °C, 72 h	-	42	13	4	71:29
7	DCM	0 °C, 72 h	$P(OPh)_3$	39	46	2	92:8
8	DCM	-30 °C, 72 h	P(1-nap)$_3$	30	20	2	92:8
9	DCM	-30 °C, 72 h	dppe	0	2	0	n.d.

General conditions: Substrate (0.1 mmol), urea catalyst (*S*)-**3d** (10 mol%), $[Rh(cod)Cl]_2$ (2.5 mol), ligand (5 mol%) and KF (3 equiv.) in 500 µL of solvent stirred at 1200 rpm. [a]Determined by ^{19}F NMR using 4-fluoroanisole as internal standard. [b]e.r. was determined by HPLC analysis using a chiral stationary phase. [c]Reaction carried out under N_2. n.d. = not determined. dppe = 1,2-bis(diphenylphosphino)ethane.

Urea Catalyst Synthesis and Characterisation

Catalysts **3a–c**[1], **3d**, **3g** and **3h**[2] were known and prepared according to literature procedures.

General Procedure A

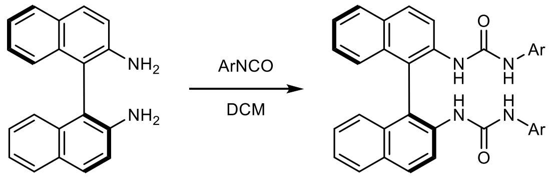

In a flame-dried Schlenk flask under inert atmosphere, (*S*)-BINAM (1 equiv.) was dissolved in dry DCM (0.4 M) and the appropriate isocyanate (2 equiv.) was added dropwise. The mixture was stirred at room temperature for the indicated time. After removal of the solvent under reduced pressure, the crude mixture was directly purified by FCC to afford the desired product.

General Procedure B

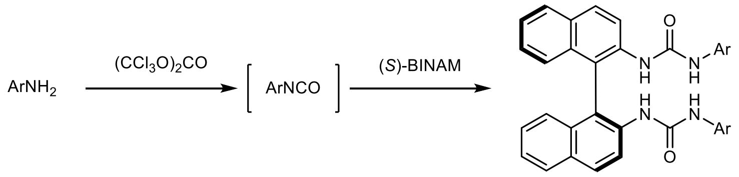

In a flame-dried two-neck flask under inert atmosphere, triphosgene (0.7 equiv.) was dissolved in dry DCM (5 mL). To this solution, a 0.2 M solution of the appropriate aniline (2 equiv.) in DCM was added at 0 °C. Then dry NEt_3 (4 equiv.) was added dropwise at 0 °C and the reaction mixture was allowed to stir at room temperature for 2 h under a flow of nitrogen. (*S*)-BINAM (1 equiv.) was then added and the reaction stirred at room temperature for 24 h. The reaction was quenched by addition of water and the aqueous phase was extracted with DCM three times. The combined organic fractions were washed with brine, dried over $MgSO_4$ and evaporated *in vacuo*. The crude mixture was then purified by FCC.

(*S*)-1,1'-([1,1'-binaphthalene]-2,2'-diyl)bis(3-(3,5-difluorophenyl)urea) (3e)

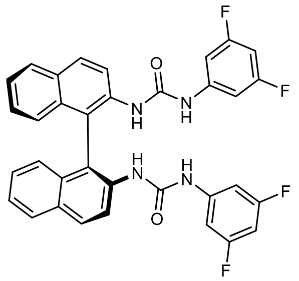

Prepared according to General Procedure A from 3,5-difluorophenyl isocyanate (0.24 mL, 2 mmol), stirred over 24 h. Purification by FCC (85:15 to 80:20 pentane:Et_2O) afforded the title compound (203 mg, 34%) as a white solid. **^{1}H NMR** (400 MHz, DMSO-d_6) δ 9.31 (s, 2H), 8.41 (d, *J* = 9.1 Hz, 2H), 8.12 (d, *J* = 9.1 Hz, 2H), 8.04 – 7.98 (m, 2H), 7.51 (s, 2H), 7.42 (ddd, *J* = 8.1, 6.9, 1.2 Hz, 2H), 7.26 (ddd, *J* = 8.3, 6.8, 1.3 Hz, 2H), 7.01 – 6.90 (m, 4H), 6.80 (dd, *J* = 8.4, 1.2 Hz, 2H), 6.69 (tt, *J* = 9.3, 2.4 Hz, 2H). **^{13}C NMR** (101 MHz, DMSO-d_6) δ 162.5 (dd, *J* = 242.6, 15.6 Hz), 152.4, 142.1 (t, *J* = 14.0 Hz), 136.1, 132.6, 130.3, 129.0, 128.2, 126.8, 124.7, 124.4, 122.1, 119.5, 100.8 (d, *J* = 29.3 Hz), 96.7 (t, *J* = 25.8 Hz).**^{19}F NMR** (377 MHz, DMSO-d_6) δ -109.8 (t, *J* = 9.4 Hz). **IR** (liquid film) ν = 2925, 2856, 1722, 1668, 1602, 1544, 1506, 1477, 1341, 1280, 1255, 1233, 1199, 1162, 1116, 986, 830, 751, 666 cm^{-}

[1]. **HRMS** (ESI$^+$) m/z calculated for $[M+H]^+$ 595.1752, found 595.1747. **m.p.** 153 – 156 °C. **$[\alpha]_D^{25\,°C}$** = -139.6 (c = 1.0, $CHCl_3$).

(*S*)-1,1'-([1,1'-binaphthalene]-2,2'-diyl)bis(3-(4-(pentafluoro-l6-sulfaneyl)phenyl)urea) (3f)

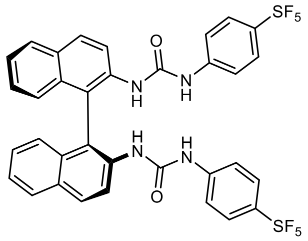

Prepared according to the General Procedure B from 4-pentafluorosulfanyl aniline (175 mg, 0.8 mmol). Purification by FCC (100:0 to 80:20 DCM:Et_2O) afforded the title compound (150 mg, 51 %) as a white solid. **^{1}H NMR** (400 MHz, $CDCl_3$) δ 8.00 (d, *J* = 9.0 Hz, 2H), 7.94 (d, *J* = 9.0 Hz, 2H), 7.89 – 7.82 (m, 2H), 7.40 (ddd, *J* = 8.1, 6.9, 1.2 Hz, 2H), 7.37 – 7.33 (m, 4H), 7.23 (s, 2H), 7.19 (ddd, *J* = 8.3, 6.8, 1.3 Hz, 2H), 6.97 (dd, *J* = 8.4, 1.1 Hz, 2H), 6.89 (d, *J* = 8.8 Hz, 4H), 6.83 (s, 2H). **^{13}C NMR** (101 MHz, $CDCl_3$) δ 153.5, 148.7 (quintet, *J* = 20 Hz), 140.5, 134.8, 132.9, 131.3, 129.9, 128.5, 127.6, 127.0, 125.9, 125.3, 122.9, 122.6, 118.8. **^{19}F NMR** (377 MHz, $CDCl_3$) δ 85.2 (quintet, *J* = 152.0 Hz, 2F), 63.5 (d, *J* = 150.8 Hz, 8F). **IR** (liquid film) ν = 3322, 1666, 1596, 1499, 1427, 1247, 1192, 1102, 832, 746, 670 cm^{-1}. **HRMS** (APCI) m/z calculated for $[M+H]^+$ 775.12538, found 775.12448. **m.p.** 180 – 188 °C. **$[\alpha]_D^{25\,°C}$** = -123.1 (c = 0.5, $CHCl_3$).

(*S*)-1,1'-([1,1'-binaphthalene]-2,2'-diyl)bis(3-(2-(trifluoromethyl)phenyl)urea) (3i)

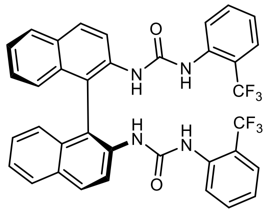

Prepared according to General Procedure A from 2-(trifluoromethyl)phenyl isocyanate (0.30 mL, 2 mmol), stirred over 72 h. Purification by FCC (100:0 to 80:20 DCM:Et_2O) afforded the title compound (580 mg, 88%) as a white solid. **^{1}H NMR** (400 MHz, DMSO-d_6) δ 8.31 (s, 2H), 8.25 (d, *J* = 9.0 Hz, 2H), 8.07 (d, *J* = 9.1 Hz, 2H), 8.03 (s, 2H), 8.00 – 7.95 (m, 2H), 7.59 (dd, *J* = 8.0, 1.5 Hz, 2H), 7.55 (d, *J* = 8.2 Hz, 2H), 7.52 – 7.46 (m, 2H), 7.40 (ddd, *J* = 8.1, 6.8, 1.2 Hz, 2H), 7.30 – 7.21 (m, 4H), 6.92 (dd, *J* = 8.5, 1.2 Hz, 2H). **^{13}C NMR** (101 MHz, DMSO-d_6) δ 153.4, 136.5, 136.0, 132.6, 132.4, 130.4, 128.6, 128.2, 127.4, 126.5, 125.9 (q, *J* = 5.3 Hz), 124.6, 124.5, 124.3, 123.7 (q, *J* = 272.8 Hz), 122.9, 121.4 (q, *J* = 29.1 Hz), 120.6. **^{19}F NMR** (377 MHz, DMSO-d_6) δ -59.4. **IR** (liquid film) ν = 2922, 1670, 1590, 1505, 1457, 1427, 1319, 1271, 1170, 1112, 1035, 767, 652 cm^{-1}. **HRMS** (APCI) m/z calculated for $[M+H]^+$ 659.18762, found 659.18676. **m.p.** 127 – 135 °C. **$[\alpha]_D^{25\,°C}$** = -118.3 (c = 0.5, $CHCl_3$).

(*S*)-1,1'-([1,1'-binaphthalene]-2,2'-diyl)bis(3-(perfluorophenyl)urea) (3j)

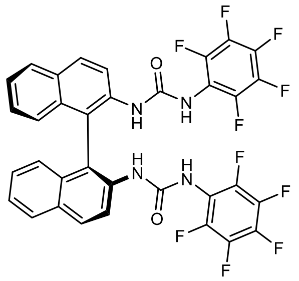

Prepared according to General Procedure A from perfluorophenyl isocyanate (0.26 mL, 2 mmol), stirred over 24 h. Filtration and washing the solid residue with hexane afforded the title compound (611 mg, 87%) as a white solid. **^{1}H NMR** (400 MHz, DMSO-d_6) δ 8.93 (s, 2H), 8.52 (d, *J* = 9.1 Hz, 2H), 8.12 (d, *J* = 9.2 Hz, 2H), 8.00 (d, *J* = 8.1 Hz, 2H), 7.71 (s, 2H), 7.42 (ddd, *J* = 8.1, 6.8, 1.2 Hz, 2H), 7.27 (ddd, *J* = 8.3, 6.8, 1.3 Hz, 2H), 6.85 – 6.78 (m, 2H). **^{13}C NMR** (101 MHz, DMSO-d_6) δ 152.0, 142.6 (d, *J* = 244.1 Hz), 138.3 (d, *J* = 251.0 Hz), 137.1 (d, *J* = 245.2 Hz), 136.5, 132.6, 130.3, 129.3, 128.2, 126.9, 124.7, 124.3, 120.9, 118.7, 113.5 (t, *J* = 14.7 Hz). **^{19}F NMR** (377 MHz, DMSO-d_6) δ -

146.2 – -146.4 (m, 4F), -160.0 (t, *J* = 22.9 Hz, 2F), -164.2 (td, *J* = 24.3, 5.6 Hz, 4F). **IR** (liquid film) ν = 3260, 1704, 1650, 1600, 1523, 1503, 1463, 1429, 1335, 1270, 1217, 1056, 1009, 981, 867, 814, 774, 747, 695, 641 cm^{-1}. **HRMS** (APCI) m/z calculated for $[M+H]^+$ 703.11863, found 703.11804. **m.p.** 162 – 164 °C. **$[\alpha]_D^{25\ °C}$** = -51.5 (c = 0.2, $CHCl_3$).

Substrate Synthesis and Characterisation

Alcohol precursor synthesis

General Procedure C

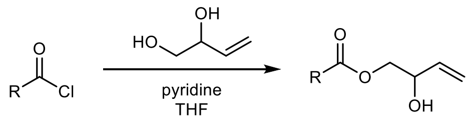

To a solution of but-3-ene-1,2-diol (1 equiv.) and pyridine (5 equiv.) in dry THF (0.5 M) at 0 °C under inert atmosphere was added the acyl chloride (1 equiv.) dropwise. The reaction was warmed to room temperature and stirred for 4 h. Water and 1N HCl were added to quench the reaction. The organic layer was separated and the aqueous layer extracted twice with EtOAc. The combined organic fractions were dried with $MgSO_4$, filtered, and evaporated *in vacuo*. The crude mixture was then purified by FCC.

General Procedure D

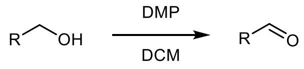

A solution of the primary alcohol (1 equiv.) in DCM (0.25 M) was cooled to 0 °C. DMP (1.2 equiv.) was added portionwise. The reaction mixture was stirred at this temperature for 15 min, warmed to room temperature, and stirred for 2 h. The reaction was quenched with sat. aq. $NaHCO_3$ and sat. aq. $Na_2S_2O_3$ and stirred vigorously for 30 min. The organic layer was separated and the aqueous layer extracted twice with DCM. The combined organic fractions were dried with $MgSO_4$, filtered, and evaporated *in vacuo* to yield the crude aldehyde.

General Procedure E

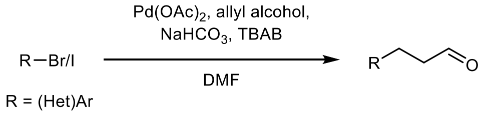

The (hetero)aryl bromide/iodide (1 equiv.), palladium acetate (7.5 mol%), tetrabutylammonium bromide (1.2 equiv.), $NaHCO_3$ (2 equiv.) and allyl alcohol (1.5 equiv.) were dissolved in degassed DMF (0.5 M) under inert atmosphere. The solution was heated to 50 °C (for aryl iodide) or 90 °C (for aryl bromide) and stirred vigorously for 16 h. Water and Et_2O were added, the two layers separated, and the aqueous layer extracted twice with Et_2O. The combined organic fractions were washed with 10% LiCl and brine, dried with $MgSO_4$, and evaporated *in vacuo* to yield the crude aldehyde.

General Procedure F

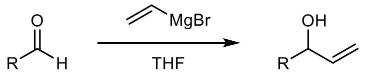

A solution of the (crude) aldehyde (1 equiv.) in dry THF (0.2 M) under inert atmosphere was cooled to 0 °C. Vinylmagnesium bromide (1 M solution in THF, 1.2 – 1.5 equiv.) was added dropwise. The reaction mixture was stirred at this temperature for 30 min, warmed to room temperature, and stirred for a further 2 h. The reaction was quenched with sat. aq. NH_4Cl. The organic layer was separated and the aqueous layer extracted

twice with EtOAc. The combined organic fractions were dried with $MgSO_4$, filtered, and evaporated *in vacuo*. The crude mixture was then purified by FCC.

General Procedure G

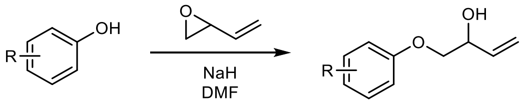

The phenol (1 equiv.) was dissolved in dry DMF (0.5 M) in a sealed tube under inert atmosphere and the solution was cooled to 0 °C. NaH (60% dispersion in mineral oil, 1 equiv.) was added portionwise, and the mixture stirred at this temperature for 30 min. Under the same temperature, butadiene monoxide (2 equiv.) was added slowly and the reaction was stirred at 80 °C for 18 h. The reaction was quenched with H_2O and extracted three times with Et_2O. The combined organic fractions were washed with 10% LiCl and brine, dried with $MgSO_4$, filtered, and evaporated *in vacuo*. The crude mixture was purified by FCC.

2-hydroxybut-3-en-1-yl 4-fluorobenzoate (6a)

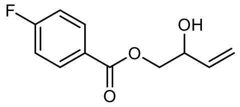

Prepared according to a literature procedure[3] on 12 mmol scale in 55% yield. All spectroscopic data were consistent with those reported in the literature. **^{1}H NMR** (400 MHz, $CDCl_3$) δ 8.13 – 8.00 (m, 2H), 7.17 – 7.06 (m, 2H), 5.94 (ddd, *J* = 17.3, 10.6, 5.6 Hz, 1H), 5.45 (dt, *J* = 17.2, 1.4 Hz, 1H), 5.29 (dt, *J* = 10.5, 1.4 Hz, 1H), 4.53 (dddd, *J* = 7.0, 5.3, 3.3, 1.8 Hz, 1H), 4.41 (dd, *J* = 11.5, 3.5 Hz, 1H), 4.29 (dd, *J* = 11.5, 7.1 Hz, 1H), 2.49 (s, 1H). **^{13}C NMR** (101 MHz, $CDCl_3$) δ 166.1 (d, *J* = 254.4 Hz), 165.9, 136.3, 132.4 (d, *J* = 9.3 Hz), 126.2 (d, *J* = 3.0 Hz), 117.4, 115.8 (d, *J* = 22.0 Hz), 71.3, 68.5. **^{19}F NMR** (377 MHz, $CDCl_3$) δ -105.2 (tt, *J* = 8.5, 5.5 Hz).

2-hydroxybut-3-en-1-yl 4-cyanobenzoate (6b)

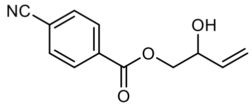

Prepared according to General Procedure C from 4-cyanobenzoyl chloride (828 mg, 5 mmol). Purification by FCC (95:5 DCM:EtOAc) afforded the title compound (664 mg, 61%) as a white solid. **^{1}H NMR** (400 MHz, $CDCl_3$) δ 8.18 – 8.10 (m, 2H), 7.78 – 7.69 (m, 2H), 5.93 (ddd, *J* = 17.2, 10.6, 5.6 Hz, 1H), 5.44 (dt, *J* = 17.3, 1.4 Hz, 1H), 5.29 (dt, *J* = 10.6, 1.4 Hz, 1H), 4.58 – 4.50 (m, 1H), 4.43 (dd, *J* = 11.4, 3.7 Hz, 1H), 4.33 (dd, *J* = 11.4, 7.1 Hz, 1H), 2.16 (s, 1H). **^{13}C NMR** (101 MHz, $CDCl_3$) δ 165.1, 136.1, 133.8, 132.4, 130.3, 118.0, 117.6, 116.7, 71.1, 68.8. **IR** (liquid film) ν = 3424, 2980, 2232, 1720, 1497, 1409, 1276, 1187, 1107, 990, 933, 905, 865, 768, 690 cm^{-1}. **HRMS** (ESI^+) m/z calculated for $[M+H]^+$ 218.0812, found 218.0808. **m.p.** 77 – 80 °C.

2-hydroxybut-3-en-1-yl benzoate (6c)

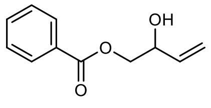

Prepared according to General Procedure C from benzoyl chloride (0.58 mL, 5 mmol). Purification by FCC (90:10 to 85:15 pentane:EtOAc) afforded the title compound (558 mg, 58%) as a white solid. All spectroscopic data were consistent with those reported in the literature[4]. **^{1}H NMR** (400 MHz, $CDCl_3$) δ 8.11 – 8.02 (m, 2H), 7.64 – 7.52 (m, 1H), 7.51 – 7.37 (m, 2H), 5.95 (ddd, *J* = 17.5, 10.6, 5.6 Hz, 1H), 5.45 (dt, *J* = 17.3, 1.4 Hz, 1H), 5.28 (dt, *J* = 10.5, 1.3 Hz, 1H), 4.59 –

4.49 (m, 1H), 4.42 (dd, J = 11.4, 3.6 Hz, 1H), 4.30 (dd, J = 11.4, 7.1 Hz, 1H), 2.63 (s, 1H). **^{13}C NMR** (101 MHz, $CDCl_3$) δ 166.8, 136.3, 133.3, 130.0, 129.8, 128.6, 117.3, 71.3, 68.4.

2-hydroxybut-3-en-1-yl 4-(*tert*-butyl)benzoate (6d)

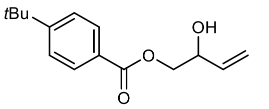

Prepared according to General Procedure C from 4-(*tert*-butyl)benzoyl chloride (0.98 mL, 5 mmol). Purification by FCC (97:3 DCM:EtOAc) afforded the title compound (797 mg, 64%) as a colourless oil. **^{1}H NMR** (400 MHz, $CDCl_3$) δ 8.01 – 7.93 (m, 2H), 7.49 – 7.42 (m, 2H), 5.94 (ddd, J = 17.3, 10.5, 5.5 Hz, 1H), 5.44 (dt, J = 17.3, 1.4 Hz, 1H), 5.27 (dt, J = 10.5, 1.5 Hz, 1H), 4.56 – 4.47 (m, 1H), 4.41 (dd, J = 11.5, 3.6 Hz, 1H), 4.28 (dd, J = 11.4, 7.1 Hz, 1H), 2.33 (s, 1H), 1.33 (s, 9H). **^{13}C NMR** (101 MHz, $CDCl_3$) δ 166.9, 157.1, 136.4, 129.7, 127.1, 125.5, 117.2, 71.3, 68.3, 35.2, 31.2. **IR** (liquid film) ν = 3475, 2965, 1720, 1610, 1409, 1366, 1278, 1190, 1120, 1017, 988, 928, 855, 775, 708 cm^{-1}. **HRMS** (ESI$^+$) m/z calculated for $[M+H]^+$ 249.1485, found 249.1482.

2-hydroxybut-3-en-1-yl 4-(4,4,5,5-tetraethyl-1,3,2-dioxaborolan-2-yl)benzoate (6e)

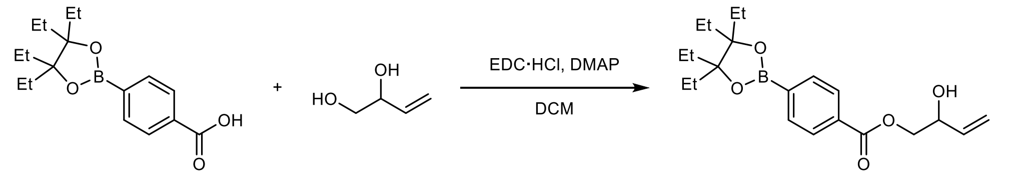

4-(4,4,5,5-tetraethyl-1,3,2-dioxaborolan-2-yl)benzoic acid[5] (1.52 g, 5 mmol, 1 equiv.), but-3-ene-1,2-diol (0.62 mL, 7.5 mmol, 1.5 equiv.) and DMAP (61 mg, 0.5 mmol, 10 mol%) were dissolved in anhydrous DCM (25 mL) under an inert atmosphere. EDC hydrochloride (1.44 g, 7.5 mmol, 1.5 equiv.) was then added and the solution stirred at room temperature overnight. The reaction was quenched with sat. aq. NH_4Cl. The organic layer was separated and the aqueous layer extracted twice with DCM. The combined organic fractions were dried with $MgSO_4$, filtered, and evaporated *in vacuo*. Purification by FCC (90:10 to 80:20 pentane:EtOAc) afforded the title compound (1.25 g, 67%) as a colourless oil. **^{1}H NMR** (400 MHz, $CDCl_3$) δ 8.02 (d, J = 7.9 Hz, 2H), 7.88 (d, J = 7.8 Hz, 2H), 5.94 (ddd, J = 17.5, 10.6, 5.6 Hz, 1H), 5.44 (dt, J = 17.2, 1.5 Hz, 1H), 5.27 (dt, J = 10.6, 1.4 Hz, 1H), 4.53 (dddd, J = 7.0, 5.3, 3.5, 1.6 Hz, 1H), 4.42 (dd, J = 11.5, 3.6 Hz, 1H), 4.30 (dd, J = 11.5, 7.2 Hz, 1H), 2.16 (s, 1H), 1.77 (ddp, J = 21.6, 14.5, 7.4 Hz, 8H), 0.97 (t, J = 7.5 Hz, 12H). **^{13}C NMR** (101 MHz, $CDCl_3$) δ 166.9, 136.3, 134.9, 131.9, 128.8, 117.3, 89.4, 71.3, 68.5, 26.6, 9.0. **IR** (liquid film) ν = 3506, 2979, 1724, 1511, 1459, 1401, 1367, 1354, 1311, 1272, 1114, 1096, 1021, 922, 710 cm^{-1}. **HRMS** (ESI$^+$) m/z calculated for $[M+H]^+$ 375.2337, found 375.2329.

2-hydroxybut-3-en-1-yl 2-fluorobenzoate (6f)

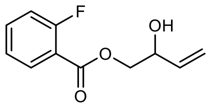

Prepared according to General Procedure C from 2-fluorobenzoyl chloride (0.60 mL, 5 mmol). Purification by FCC (85:15 pentane:EtOAc) afforded the title compound (618 mg, 59%) as a colourless oil. **^{1}H NMR** (400 MHz, $CDCl_3$) δ 7.95 (td, J = 7.5, 1.9 Hz, 1H), 7.58 – 7.48 (m, 1H), 7.21 (t, J = 7.6 Hz, 1H), 7.14 (dd, J = 10.9, 8.3 Hz, 1H), 5.94 (ddd, J = 16.6, 10.6, 5.5 Hz, 1H), 5.44 (dt, J = 17.2, 1.5 Hz, 1H), 5.28 (dt, J = 10.5, 1.5 Hz, 1H), 4.57 – 4.48 (m, 1H), 4.44 (dd, J = 11.3, 3.5 Hz,

1H), 4.29 (dd, *J* = 11.4, 7.2 Hz, 1H), 2.17 (s, 1H). **^{13}C NMR** (101 MHz, $CDCl_3$) δ 164.6 (d, *J* = 3.7 Hz), 162.1 (d, *J* = 259.8 Hz), 136.1, 134.9 (d, *J* = 9.0 Hz), 132.4, 124.2 (d, *J* = 3.9 Hz), 118.5 (d, *J* = 9.8 Hz), 117.4, 117.2 (d, *J* = 22.6 Hz), 71.0, 68.7. **^{19}F NMR** (377 MHz, $CDCl_3$) δ -109.0 (ddd, *J* = 11.5, 7.2, 4.9 Hz). **IR** (liquid film) ν = 3497, 2955, 2921, 1717, 1613, 1489, 1457, 1299, 1256, 1130, 1085, 1034, 990, 931, 803, 756, 692 cm^{-1}. **HRMS** (ESI$^+$) m/z calculated for [M+Na]$^+$ 233.0584, found 233.0591.

3-hydroxypent-4-en-1-yl 4-fluorobenzoate (6g)

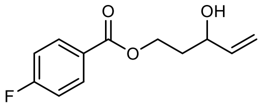

Prepared according to General Procedure C from 4-fluorobenzoyl chloride (0.55 mL, 4.7 mmol) and pent-4-ene-1,3-diol[6] (480 mg, 4.7 mmol). Purification by FCC (85:15 pentane:EtOAc) afforded the title compound (705 mg, 67%) as a colourless oil. **^{1}H NMR** (400 MHz, $CDCl_3$) δ 8.10 – 8.00 (m, 2H), 7.17 – 7.04 (m, 2H), 5.92 (dddd, *J* = 17.6, 10.5, 6.0, 1.4 Hz, 1H), 5.29 (dq, *J* = 17.1, 1.4 Hz, 1H), 5.15 (dq, *J* = 10.4, 1.5 Hz, 1H), 4.60 – 4.48 (m, 1H), 4.45 – 4.36 (m, 1H), 4.34 – 4.26 (m, 1H), 2.14 – 1.89 (m, 3H). **^{13}C NMR** (101 MHz, $CDCl_3$) δ 166.0, 165.9 (d, *J* = 253.9 Hz), 140.4, 132.3 (d, *J* = 9.4 Hz), 126.5 (d, *J* = 3.1 Hz), 115.7 (d, *J* = 22.1 Hz), 115.4, 70.0, 62.0, 36.1. **^{19}F NMR** (377 MHz, $CDCl_3$) δ -105.6 (tt, *J* = 8.5, 5.5 Hz). **IR** (liquid film) ν = 3435, 3080, 2982, 1718, 1604, 1508, 1411, 1279, 1239, 1154, 1119, 1092, 1014, 993, 968, 927, 855, 806, 768, 688, 609 cm^{-1}. **HRMS** (ESI$^+$) m/z calculated for [M+H]$^+$ 225.0922, found 225.0920.

2-hydroxybut-3-en-1-yl 3-phenylpropanoate (6h)

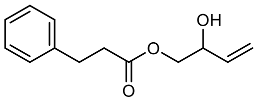

Prepared according to General Procedure C from 3-phenylpropanoyl chloride (1.35 g, 8 mmol). Purification by FCC (90:10 to 80:20 pentane:EtOAc) afforded the title compound (954 mg, 54%) as a yellow oil. **^{1}H NMR** (400 MHz, $CDCl_3$) δ 7.34 – 7.27 (m, 2H), 7.24 – 7.16 (m, 3H), 5.81 (ddd, *J* = 17.3, 10.6, 5.5 Hz, 1H), 5.36 (dt, *J* = 17.3, 1.5 Hz, 1H), 5.23 (dt, *J* = 10.6, 1.4 Hz, 1H), 4.37 – 4.28 (m, 1H), 4.17 (dd, *J* = 11.4, 3.4 Hz, 1H), 4.01 (dd, *J* = 11.5, 7.4 Hz, 1H), 2.97 (t, *J* = 7.7 Hz, 2H), 2.69 (t, *J* = 7.7 Hz, 2H), 1.97 (s, 1H). **^{13}C NMR** (101 MHz, $CDCl_3$) δ 173.1, 140.4, 136.1, 128.7, 128.4, 126.5, 117.2, 71.1, 68.0, 35.8, 31.0. **IR** (liquid film) ν = 3467, 3028, 2947, 1736, 1497, 1384, 1292, 1259, 1163, 1079, 992, 931, 751, 700 cm^{-1}. **HRMS** (ESI$^+$) m/z calculated for [M+H]$^+$ 221.1172, found 221.1169.

1-([1,1'-biphenyl]-4-yloxy)but-3-en-2-ol (6i)

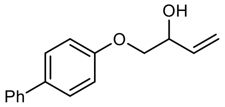

Prepared according to General Procedure G from [1,1'-biphenyl]-4-ol (851 mg, 5 mmol). Purification by FCC (85:15 pentane:Et_2O) afforded the title compound (565 mg, 47%) as a white solid. **^{1}H NMR** (400 MHz, $CDCl_3$) δ 7.61 – 7.51 (m, 4H), 7.43 (t, *J* = 7.7 Hz, 2H), 7.35 – 7.29 (m, 1H), 7.03 – 6.97 (m, 2H), 5.98 (ddd, *J* = 17.2, 10.6, 5.6 Hz, 1H), 5.48 (dt, *J* = 17.3, 1.5 Hz, 1H), 5.31 (dt, *J* = 10.6, 1.4 Hz, 1H), 4.63 – 4.54 (m, 1H), 4.08 (dd, *J* = 9.5, 3.4 Hz, 1H), 3.94 (dd, *J* = 9.4, 7.6 Hz, 1H), 2.39 (s, 1H). **^{13}C NMR** (101 MHz, $CDCl_3$) δ 158.2, 140.8, 136.2, 134.5, 128.9, 128.4, 126.91, 126.89, 117.4, 115.1, 72.0, 71.4. **IR** (liquid film) ν = 3439, 2922, 2865, 1605, 1584, 1524, 1489, 1456, 1319,

1285, 1271, 1247, 1202, 1179, 1149, 1118, 1042, 996, 954, 933, 896, 832, 763, 712, 694, 670, 614 cm^{-1}. **m.p.** 104 – 106 °C. No HRMS obtained.

1-(4-methoxyphenoxy)but-3-en-2-ol (6j)

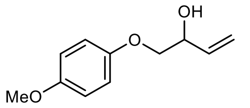

Prepared according to General Procedure G from 4-methoxyphenol (932 mg, 7.5 mmol). Purification by FCC (95:5 to 85:15 pentane:EtOAc) afforded the title compound (688 mg, 47%) as a yellow oil. All spectroscopic data were consistent with those reported in the literature[7]. **^{1}H NMR** (400 MHz, $CDCl_3$) δ 6.91 – 6.79 (m, 4H), 5.94 (ddd, *J* = 17.3, 10.6, 5.6 Hz, 1H), 5.44 (dt, *J* = 17.3, 1.5 Hz, 1H), 5.28 (dt, *J* = 10.6, 1.4 Hz, 1H), 4.57 – 4.47 (m, 1H), 3.98 (dd, *J* = 9.4, 3.4 Hz, 1H), 3.84 (dd, *J* = 9.4, 7.7 Hz, 1H), 3.77 (s, 3H), 2.29 (s, 1H). **^{13}C NMR** (101 MHz, $CDCl_3$) δ 154.4, 152.7, 136.2, 117.2, 115.9, 114.8, 72.7, 71.4, 55.9.

5-(benzyloxy)pent-1-en-3-ol (6k)

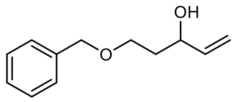

Prepared according to General Procedure F from 3-(benzyloxy)propanal (985 mg, 6 mmol). Purification by FCC (100:0 to 95:5 DCM:EtOAc) afforded the title compound (752 mg, 65%) as a colourless oil. All spectroscopic data were consistent with those reported in the literature[8]. **^{1}H NMR** (400 MHz, $CDCl_3$) δ 7.40 – 7.27 (m, 5H), 5.88 (ddd, *J* = 17.3, 10.5, 5.5 Hz, 1H), 5.27 (dt, *J* = 17.1, 1.6 Hz, 1H), 5.11 (dt, *J* = 10.5, 1.5 Hz, 1H), 4.52 (s, 2H), 4.39 – 4.31 (m, 1H), 3.76 – 3.68 (m, 1H), 3.68 – 3.59 (m, 1H), 2.58 (s, 1H), 1.93 – 1.76 (m, 2H). **^{13}C NMR** (101 MHz, $CDCl_3$) δ 140.7, 138.1, 128.6, 127.9, 127.8, 114.5, 73.4, 72.0, 68.5, 36.4.

2-hydroxybut-3-en-1-yl 4-methylbenzenesulfonate (6l)

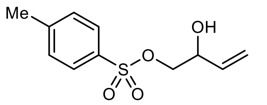

Prepared according to a literature procedure[9] on 10 mmol scale in 89% yield. All spectroscopic data were consistent with those reported in the literature. **^{1}H NMR** (400 MHz, $CDCl_3$) δ 7.80 (d, *J* = 8.4 Hz, 2H), 7.35 (d, *J* = 8.1 Hz, 2H), 5.75 (ddd, *J* = 17.3, 10.6, 5.5 Hz, 1H), 5.38 (dt, *J* = 17.2, 1.4 Hz, 1H), 5.25 (dt, *J* = 10.6, 1.4 Hz, 1H), 4.44 – 4.35 (m, 1H), 4.07 (dd, *J* = 10.3, 3.4 Hz, 1H), 3.91 (dd, *J* = 10.2, 7.4 Hz, 1H), 2.45 (s, 3H), 2.14 (s, 1H). **^{13}C NMR** (101 MHz, $CDCl_3$) δ 145.3, 134.7, 132.8, 130.1, 128.1, 118.3, 73.1, 70.6, 21.8.

2-hydroxybut-3-en-1-yl 4-fluorobenzenesulfonate (6m)

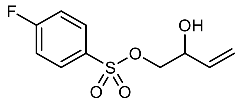

Prepared according to General Procedure C from 4-fluorobenzenesulfonyl chloride (1.56 g, 8 mmol). Purification by FCC (75:25 pentane:EtOAc) afforded the title compound (1.32 g, 67%) as a colourless oil. **^{1}H NMR** (400 MHz, $CDCl_3$) δ 8.01 – 7.91 (m, 2H), 7.30 – 7.20 (m, 2H), 5.77 (ddd, *J* = 17.4, 10.5, 5.5 Hz, 1H), 5.39 (dt, *J* = 17.2, 1.4 Hz, 1H), 5.26 (dt, *J* = 10.5, 1.4 Hz, 1H), 4.42 (dddd, *J* = 7.1, 5.2, 3.4, 1.6 Hz, 1H), 4.11 (dd, *J* = 10.2, 3.4 Hz, 1H), 3.96 (dd, *J* = 10.2, 7.3 Hz, 1H), 2.28 (s, 1H). **^{13}C NMR** (101 MHz, $CDCl_3$) δ 166.0 (d, *J* = 256.7 Hz), 134.7, 131.9 (d, *J* = 3.2 Hz), 131.0 (d, *J* = 9.6 Hz), 118.4, 116.8 (d, *J* = 23.0 Hz), 73.3, 70.5. **^{19}F NMR** (377 MHz, $CDCl_3$) δ -102.7

(tt, *J* = 8.1, 4.5 Hz). **IR** (liquid film) ν = 3524, 2981, 1593, 1495, 1409, 1361, 1296, 1241, 1185, 1159, 1096, 971, 889, 840, 673 cm^{-1}. **HRMS** (ESI^+) m/z calculated for $[M+H]^+$ 247.0435, found 247.0431.

2-hydroxybut-3-en-1-yl diphenylphosphinate (6n)

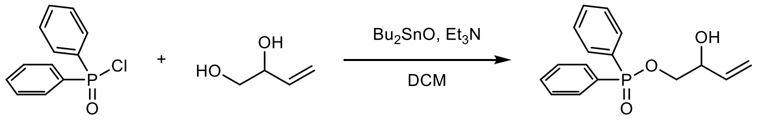

A mixture of but-3-ene-1,2-diol (0.42 mL, 5 mmol, 1 equiv.), dibutyltin oxide (125 mg, 0.5 mmol, 10 mol%) and triethylamine (0.70 mL, 5 mmol, 1 equiv.) in dry DCM under inert atmosphere was stirred at room temperature for 1 h. The suspension was then cooled to 0 °C. Diphenylphosphinic chloride (0.96 mL, 5 mmol, 1 equiv.) was added dropwise. The reaction mixture was warmed to room temperature and stirred for 2 h. Upon completion, the reaction was filtered through Celite and washed with water. The organic fraction was dried with $MgSO_4$ and evaporated *in vacuo*. Purification by FCC (Et_2O) afforded the title compound (1.33 g, 89%) as a white solid. **^{1}H NMR** (400 MHz, $CDCl_3$) δ 7.87 – 7.77 (m, 4H), 7.58 – 7.50 (m, 2H), 7.50 – 7.40 (m, 4H), 5.83 (ddd, *J* = 17.2, 10.6, 5.3 Hz, 1H), 5.42 (dt, *J* = 17.2, 1.6 Hz, 1H), 5.22 (dt, *J* = 10.6, 1.5 Hz, 1H), 4.49 – 4.40 (m, 1H), 4.11 (td, *J* = 11.5, 2.7 Hz, 1H), 3.95 (ddd, *J* = 11.4, 10.5, 6.9 Hz, 1H), 3.92 (s, 1H). **^{13}C NMR** (101 MHz, $CDCl_3$) δ 135.8, 132.6 (d, *J* = 2.8 Hz), 131.9 (d, *J* = 10.3 Hz), 131.8 (d, *J* = 10.3 Hz), 130.6 (d, *J* = 138.3 Hz), 130.5 (d, *J* = 137.1 Hz), 128.81 (d, *J* = 13.4 Hz), 128.79 (d, *J* = 13.4 Hz), 117.3, 71.7 (d, *J* = 3.6 Hz), 70.6 (d, *J* = 6.5 Hz). **^{31}P NMR** (162 MHz, $CDCl_3$) δ 35.3. **IR** (liquid film) ν = 3354, 3059, 2969, 1591, 1486, 1439, 1389, 1199, 1132, 1113, 1074, 1006, 985, 931, 901, 756, 729, 696 cm^{-1}. **HRMS** (ESI^+) m/z calculated for $[M+H]^+$ 289.0988, found 289.0983. **m.p.** 59 – 62 °C.

1-((*tert*-butyldiphenylsilyl)oxy)but-3-en-2-ol (6o)

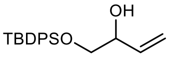

Prepared according to a literature procedure[10] on 12 mmol scale in 98% yield. All spectroscopic data were consistent with those reported in the literature. **^{1}H NMR** (400 MHz, $CDCl_3$) δ 7.70 – 7.63 (m, 4H), 7.47 – 7.35 (m, 6H), 5.79 (ddd, *J* = 17.3, 10.6, 5.6 Hz, 1H), 5.32 (dt, *J* = 17.3, 1.6 Hz, 1H), 5.17 (dt, *J* = 10.6, 1.5 Hz, 1H), 4.30 – 4.19 (m, 1H), 3.70 (dd, *J* = 10.2, 3.7 Hz, 1H), 3.55 (dd, *J* = 10.1, 7.5 Hz, 1H), 2.36 (s, 1H), 1.07 (s, 9H). **^{13}C NMR** (101 MHz, $CDCl_3$) δ 136.7, 135.72, 135.68, 134.9, 133.2, 133.1, 130.0, 129.8, 127.94, 127.87, 116.7, 73.2, 67.8, 27.0, 19.4.

2-hydroxybut-3-en-1-yl diphenylcarbamate (6p)

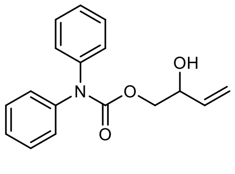

Prepared according to General Procedure C from diphenylcarbamoyl chloride (1.39 g, 6 mmol) at 60 °C for 48 h. Purification by FCC (75:25 pentane:EtOAc) afforded the title compound (395 mg, 23%) as a white solid. **^{1}H NMR** (400 MHz, $CDCl_3$) δ 7.38 – 7.31 (m, 4H), 7.29 – 7.19 (m, 6H), 5.79 (ddd, *J* = 17.2, 10.5, 5.4 Hz, 1H), 5.34 (dt, *J* = 17.2, 1.5 Hz, 1H), 5.21 (dt, *J* = 10.6, 1.5 Hz, 1H), 4.33 (dddt, *J* = 6.9, 5.0, 3.2, 1.5 Hz, 1H), 4.25 (dd, *J* = 11.4, 3.4 Hz, 1H), 4.13 (dd, *J* = 11.4, 7.1 Hz, 1H), 2.28 (s, 1H). **^{13}C NMR** (101 MHz, $CDCl_3$) δ 155.1, 142.4, 136.1, 129.1, 127.0, 126.5, 117.0, 71.4, 69.6. **IR** (liquid film) ν = 3449, 2943, 1691, 1594, 1492, 1447, 1423, 1385,

1340, 1307, 1251, 1222, 1083, 1058, 1026, 993, 932, 872, 797, 760, 694 cm^{-1}. **HRMS** (ESI^+) m/z calculated for $[M+H]^+$ 284.1281, found 284.1277. **m.p.** 91 – 92 °C.

2-(3-hydroxypent-4-en-1-yl)isoindoline-1,3-dione (6q)

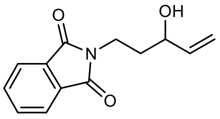

Prepared according to General Procedure F from 3-(1,3-dioxoisoindolin-2-yl)propanal (1.63 g, 8 mmol). Purification by FCC (1st column: 90:10 to 80:20 DCM:EtOAc, 2nd column: 75:25 pentane:EtOAc) afforded the title compound (450 mg, 24%) as a white solid. **^{1}H NMR** (400 MHz, $CDCl_3$) δ 7.84 (dd, *J* = 5.4, 3.1 Hz, 2H), 7.72 (dd, *J* = 5.5, 3.1 Hz, 2H), 5.86 (ddd, *J* = 17.2, 10.5, 5.5 Hz, 1H), 5.26 (dt, *J* = 17.1, 1.5 Hz, 1H), 5.09 (dt, *J* = 10.5, 1.4 Hz, 1H), 4.17 – 4.07 (m, 1H), 3.85 (dd, *J* = 7.5, 5.7 Hz, 2H), 2.44 (s, 1H), 1.97 – 1.75 (m, 2H). **^{13}C NMR** (101 MHz, $CDCl_3$) δ 168.9, 140.0, 134.2, 132.2, 123.5, 115.0, 69.8, 35.8, 34.6. **IR** (liquid film) ν = 3492, 2954, 2865, 1766, 1700, 1613, 1468, 1440, 1396, 1368, 1187, 1157, 1067, 1001, 956, 919, 892, 719 cm^{-1}. **HRMS** (ESI^+) m/z calculated for $[M+H]^+$ 232.0968, found 232.0966. **m.p.** 45 – 47 °C.

5-(naphthalen-2-ylthio)pent-1-en-3-ol (6r)

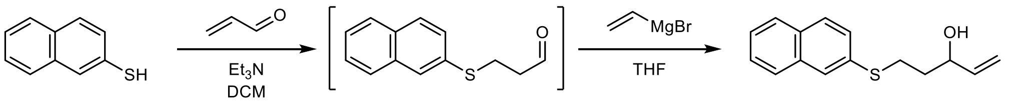

To a solution of naphthalene-2-thiol (801 mg, 5 mmol, 1 equiv.) and acrolein (0.74 mL, 10 mmol, 2 equiv.) in dry DCM (25 mL) at 0 °C under inert atmosphere was added triethylamine (70 µL, 0.5 mmol, 10 mol%) dropwise. The reaction mixture was warmed up to room temperature, stirred for 2 h, and concentrated *in vacuo* to obtain the crude aldehyde, which was then directly subjected to General Procedure F. Purification by FCC (DCM) afforded the title compound (421 mg, 34% over two steps) as a yellow oil. **^{1}H NMR** (400 MHz, $CDCl_3$) δ 7.83 – 7.70 (m, 4H), 7.52 – 7.39 (m, 3H), 5.87 (ddd, *J* = 17.3, 10.4, 6.0 Hz, 1H), 5.27 (dt, *J* = 17.2, 1.4 Hz, 1H), 5.15 (dt, *J* = 10.4, 1.3 Hz, 1H), 4.34 (tdd, *J* = 7.5, 6.1, 1.3 Hz, 1H), 3.22 – 3.06 (m, 2H), 1.98 – 1.85 (m, 2H), 1.73 (s, 1H). **^{13}C NMR** (101 MHz, $CDCl_3$) δ 140.5, 133.90, 133.88, 131.9, 128.6, 127.8, 127.4, 127.2, 127.0, 126.7, 125.8, 115.4, 71.9, 36.1, 29.6. **IR** (liquid film) ν = 3400, 3053, 1624, 1590, 1501, 1427, 1338, 1268, 1133, 1070, 1043, 992, 926, 851, 812, 744 cm^{-1}. **HRMS** (GC EI) m/z calculated for $[M]^{+\cdot}$ 244.09164, found 244.09396.

5-(4-bromophenyl)pent-1-en-3-ol (6s)

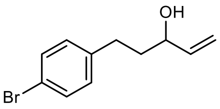

Prepared according to General Procedure F from 3-(4-bromophenyl)propanal (1.0 g, 4.7 mmol). Purification by FCC (100:0 to 95:5 DCM:EtOAc) afforded the title compound (708 mg, 62%) as a yellow oil. All spectroscopic data were consistent with those reported in the literature[11]. **^{1}H NMR** (400 MHz, $CDCl_3$) δ 7.43 – 7.36 (m, 2H), 7.08 (d, *J* = 8.1 Hz, 2H), 5.89 (ddd, *J* = 16.9, 10.4, 6.2 Hz, 1H), 5.24 (dt, *J* = 17.2, 1.4 Hz, 1H), 5.14 (dt, *J* = 10.4, 1.4 Hz, 1H), 4.11 (q, *J* = 6.4 Hz, 1H), 2.76 – 2.59 (m, 2H), 1.91 – 1.73 (m, 2H), 1.54 (s, 1H). **^{13}C NMR** (101 MHz, $CDCl_3$) δ 140.9, 140.8, 131.4, 130.2, 119.6, 115.1, 72.3, 38.3, 31.0.

5-(3,4-dimethoxyphenyl)pent-1-en-3-ol (6t)

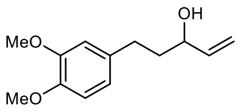

Prepared according to General Procedure D & F from 3-(3,4-dimethoxyphenyl)propan-1-ol (1.57 g, 8 mmol). Purification by FCC (100:0 to 90:10 DCM:EtOAc) afforded the title compound (750 mg, 42% over two steps) as a colourless oil. All spectroscopic data were consistent with those reported in literature[12]. **^{1}H NMR** (400 MHz, $CDCl_3$) δ 6.82 – 6.77 (m, 1H), 6.77 – 6.71 (m, 2H), 5.91 (ddd, *J* = 17.0, 10.4, 6.1 Hz, 1H), 5.25 (dt, *J* = 17.2, 1.4 Hz, 1H), 5.14 (dt, *J* = 10.4, 1.3 Hz, 1H), 4.18 – 4.09 (m, 1H), 3.87 (s, 3H), 3.86 (s, 3H), 2.75 – 2.59 (m, 2H), 1.91 – 1.77 (m, 2H), 1.54 (s, 1H). **^{13}C NMR** (101 MHz, $CDCl_3$) δ 149.0, 147.4, 141.2, 134.6, 120.3, 115.1, 111.9, 111.4, 72.6, 56.1, 56.0, 38.8, 31.4.

5,5-diphenylpent-1-en-3-ol (6u)

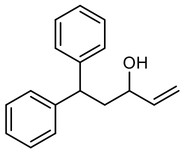

Prepared according to General Procedure D & F from 3,3-diphenylpropan-1-ol (1.70 g, 8 mmol). Purification by FCC (DCM) afforded the title compound (1.32 g, 69% over two steps) as a white solid. **^{1}H NMR** (400 MHz, CD_2Cl_2) δ 7.35 – 7.26 (m, 8H), 7.25 – 7.16 (m, 2H), 5.98 – 5.86 (m, 1H), 5.17 (dd, *J* = 17.2, 1.6 Hz, 1H), 5.12 (dd, *J* = 10.4, 1.6 Hz, 1H), 4.21 (t, *J* = 7.9 Hz, 1H), 3.93 (q, *J* = 7.0 Hz, 1H), 2.32 – 2.18 (m, 2H), 1.66 (s, 1H). **^{13}C NMR** (101 MHz, CD_2Cl_2) δ 145.4, 144.9, 141.7, 128.93, 128.91, 128.4, 128.1, 126.7, 126.6, 114.9, 71.3, 47.7, 43.0. **IR** (liquid film) ν = 3241, 3086, 3062, 3027, 1599, 1494, 1450, 1306, 1133, 1087, 1033, 995, 912, 878, 842, 793, 751, 740, 704, 633 cm^{-1}. **m.p.** 64 – 66 °C. No HRMS obtained.

5-(4-((trimethylsilyl)ethynyl)phenyl)pent-1-en-3-ol (6v)

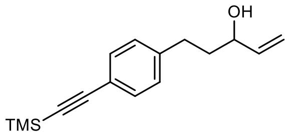

Prepared according to General Procedure E & F from ((4-iodophenyl)ethynyl) trimethylsilane (2.4 g, 8 mmol). Purification by FCC (DCM) afforded the title compound (523 mg, 25% over two steps) as a yellow oil. **^{1}H NMR** (400 MHz, $CDCl_3$) δ 7.42 – 7.34 (m, 2H), 7.16 – 7.10 (m, 2H), 5.88 (ddd, *J* = 16.8, 10.4, 6.2 Hz, 1H), 5.23 (dt, *J* = 17.2, 1.4 Hz, 1H), 5.13 (dt, *J* = 10.4, 1.2 Hz, 1H), 4.16 – 4.04 (m, 1H), 2.79 – 2.61 (m, 2H), 1.90 – 1.74 (m, 2H), 1.54 (d, *J* = 4.1 Hz, 1H), 0.24 (s, 9H). **^{13}C NMR** (101 MHz, $CDCl_3$) δ 142.7, 141.0, 132.2, 128.5, 120.7, 115.2, 105.4, 93.7, 72.5, 38.3, 31.7, 0.2. **IR** (liquid film) ν = 3373, 2960, 2157, 1507, 1409, 1250, 1223, 1048, 991, 925, 866, 842, 760, 699, 637 cm^{-1}. **HRMS** (GC EI) m/z calculated for $[M]^{+\cdot}$ 258.14344, found 258.14636.

***tert*-butyl 4-(2-hydroxybut-3-en-1-yl)piperidine-1-carboxylate (6w)**

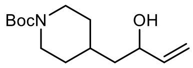

Prepared according to General Procedure F from *tert*-butyl 4-(2-oxoethyl)piperidine-1-carboxylate (1.14 g, 5 mmol). Purification by FCC (90:10 to 70:30 pentane:EtOAc) afforded the title compound (730 mg, 57%) as a colourless oil. **^{1}H NMR** (400 MHz, $CDCl_3$) δ 5.86 (ddd, *J* = 16.9, 10.4, 6.3 Hz, 1H), 5.22 (dt, *J* = 17.2, 1.2 Hz, 1H), 5.10 (dt, *J* = 10.3, 1.2 Hz, 1H), 4.25 – 4.16 (m, 1H), 4.12 – 4.01 (m, 2H), 2.75 – 2.62 (m, 2H), 1.78 – 1.70 (m, 1H), 1.69 – 1.61 (m, 2H), 1.59 (s, 1H), 1.56 – 1.46 (m, 1H), 1.44 (s, 9H), 1.42 – 1.33 (m, 1H), 1.21 – 1.03 (m, 2H). **^{13}C NMR** (101 MHz, $CDCl_3$) δ 155.0, 141.6, 114.8, 79.4, 70.7, 44.1, 44.0, 43.8, 32.8, 32.5, 32.0, 28.6. **IR** (liquid film) ν = 3439, 2978, 2925, 1695, 1672, 1427, 1366,

1278, 1245, 1170, 993, 919, 869, 769 cm^{-1}. **HRMS** (ESI$^+$) m/z calculated for $[M+H]^+$ 256.1907, found 256.1903.

5-(5-methylfuran-2-yl)pent-1-en-3-ol (6y)

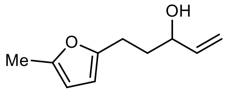

Prepared according to General Procedure F from 3-(5-methylfuran-2-yl)propanal (1.11 g, 8 mmol). Purification by FCC (95:5 to 85:15 pentane:EtOAc) afforded the title compound (1.07 g, 80%) as a colourless oil. All spectroscopic data were consistent with those reported in the literature[13]. **^{1}H NMR** (400 MHz, $CDCl_3$) δ 5.95 – 5.80 (m, 3H), 5.25 (dt, *J* = 17.2, 1.4 Hz, 1H), 5.13 (dt, *J* = 10.4, 1.3 Hz, 1H), 4.20 – 4.12 (m, 1H), 2.68 (td, *J* = 7.4, 2.3 Hz, 2H), 2.25 (d, *J* = 1.0 Hz, 3H), 1.92 – 1.78 (m, 2H), 1.66 (s, 1H). **^{13}C NMR** (101 MHz, $CDCl_3$) δ 153.8, 150.5, 140.9, 115.1, 106.0, 105.7, 72.5, 35.4, 24.1, 13.6.

***tert*-butyl 3-(3-hydroxypent-4-en-1-yl)-1*H*-indole-1-carboxylate (6z)**

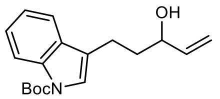

Prepared according to General Procedure E & F from *tert*-butyl 3-iodo-1*H*-indole-1-carboxylate (2.75 g, 8 mmol). Purification by FCC (90:10 pentane:EtOAc) afforded the title compound (631 mg, 26% over two steps) as a yellow oil. **^{1}H NMR** (400 MHz, $CDCl_3$) δ 8.12 (s, 1H), 7.58 – 7.51 (m, 1H), 7.38 (s, 1H), 7.35 – 7.28 (m, 1H), 7.26 – 7.19 (m, 1H), 5.94 (ddd, *J* = 16.9, 10.4, 6.1 Hz, 1H), 5.28 (dt, *J* = 17.2, 1.4 Hz, 1H), 5.16 (dt, *J* = 10.4, 1.3 Hz, 1H), 4.26 – 4.16 (m, 1H), 2.89 – 2.71 (m, 2H), 2.00 – 1.90 (m, 2H), 1.69 (s, 1H), 1.67 (s, 9H). **^{13}C NMR** (101 MHz, $CDCl_3$) δ 150.0, 141.1, 135.7, 130.8, 124.4, 122.5, 122.4, 120.7, 119.1, 115.4, 115.2, 83.5, 72.7, 36.4, 28.4, 20.8. **IR** (liquid film) ν = 3419, 2979, 2934, 1731, 1609, 1454, 1372, 1309, 1255, 1158, 1086, 1048, 923, 857, 767, 746, 639 cm^{-1}. **HRMS** (ESI$^+$) m/z calculated for $[M+H]^+$ 302.1751, found 302.1746.

5-(benzo[*b*]thiophen-2-yl)pent-1-en-3-ol (6aa)

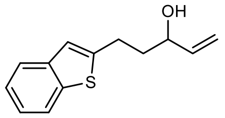

Prepared according to General Procedure E & F from 2-bromobenzo[*b*]thiophene (1.7 g, 8 mmol). Purification by FCC (DCM) afforded the title compound (331 mg, 19% over two steps) as a yellow oil. **^{1}H NMR** (400 MHz, $CDCl_3$) δ 7.77 (d, *J* = 7.8 Hz, 1H), 7.67 (d, *J* = 7.8 Hz, 1H), 7.31 (t, *J* = 7.4 Hz, 1H), 7.26 (t, *J* = 7.5 Hz, 1H), 7.04 (s, 1H), 5.92 (ddd, *J* = 16.9, 10.4, 6.1 Hz, 1H), 5.28 (dd, *J* = 17.2, 1.5 Hz, 1H), 5.17 (dd, *J* = 10.3, 1.5 Hz, 1H), 4.22 (q, *J* = 6.4 Hz, 1H), 3.10 – 2.97 (m, 2H), 2.04 – 1.93 (m, 2H), 1.63 (s, 1H). **^{13}C NMR** (101 MHz, $CDCl_3$) δ 145.8, 140.8, 140.3, 139.5, 124.2, 123.6, 122.9, 122.3, 120.9, 115.4, 72.3, 38.1, 26.8. **IR** (liquid film) ν = 3401, 3058, 2980, 1457, 1436, 1392, 1307, 1252, 1155, 1129, 1064, 991, 926, 857, 825, 745, 727 cm^{-1}. **HRMS** (GC EI) m/z calculated for $[M]^{+\cdot}$ 218.07599, found 218.07813.

(8*R*,9*S*,13*S*,14*S*)-3-((2-hydroxybut-3-en-1-yl)oxy)-13-methyl-6,7,8,9,11,12,13,14,15,16-decahydro-17*H*-cyclopenta[*a*]phenanthren-17-one (6ab)

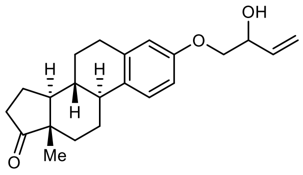

Prepared according to General Procedure G from estrone (2.03 g, 7.5 mmol). Purification by FCC (60:40 pentane:Et_2O) afforded the title compound (649 mg, 25%) as a yellow solid. **^{1}H NMR** (400 MHz, $CDCl_3$) δ 7.20 (d, *J* = 8.5 Hz, 1H), 6.73 (dd, *J* = 8.5, 2.9 Hz, 1H), 6.67 (d, *J* = 2.7 Hz, 1H), 5.94 (ddd, *J* = 17.3, 10.6, 5.6 Hz, 1H), 5.45 (dt, *J* = 17.3, 1.5 Hz, 1H), 5.28 (dt, *J* = 10.6, 1.5 Hz, 1H), 4.57 – 4.48 (m, 1H), 4.00 (ddd, *J* = 9.4, 3.4, 2.3 Hz, 1H), 3.86 (ddd, *J* = 9.8, 7.7, 2.5 Hz, 1H), 2.94 – 2.83 (m, 2H), 2.50 (dd, *J* = 18.8, 8.5 Hz, 1H), 2.44 – 2.34 (m, 1H), 2.25 (td, *J* = 10.5, 3.8 Hz, 1H), 2.20 – 2.11 (m, 1H), 2.11 – 1.91 (m, 3H), 1.69 – 1.37 (m, 7H), 0.91 (s, 3H). **^{13}C NMR** (101 MHz, $CDCl_3$) δ 221.1, 156.6, 138.0, 136.2, 132.8, 126.5, 117.2, 114.8, 112.4, 71.8, 71.3, 50.5, 48.1, 44.1, 38.4, 36.0, 31.7, 29.7, 26.6, 26.0, 21.7, 14.0. **IR** (liquid film) ν = 3436, 2980, 2924, 2857, 1738, 1612, 1573, 1498, 1458, 1376, 1338, 1280, 1255, 1186, 1157, 1086, 1057, 1006, 937, 876, 818, 780, 740, 707, 637, 619 cm^{-1}. **m.p.** 88 – 90 °C. No HRMS obtained.

1-(naphthalen-2-yl)but-3-en-2-ol (6ac)

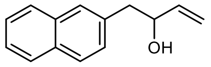

Prepared according to General Procedure D & F from 2-(naphthalen-2-yl)ethan-1-ol (1.38 g, 8 mmol). Purification by FCC (DCM) afforded the title compound (859 mg, 54% over two steps) as a yellow oil. All spectroscopic data were consistent with those reported in the literature[14]. **^{1}H NMR** (400 MHz, $CDCl_3$) δ 7.87 – 7.78 (m, 3H), 7.69 (s, 1H), 7.51 – 7.42 (m, 2H), 7.38 (dd, *J* = 8.4, 1.7 Hz, 1H), 5.99 (ddd, *J* = 16.7, 10.5, 5.8 Hz, 1H), 5.33 – 5.24 (m, 1H), 5.15 (dt, *J* = 10.5, 1.3 Hz, 1H), 4.51 – 4.41 (m, 1H), 3.05 (dd, *J* = 13.6, 5.1 Hz, 1H), 2.96 (dd, *J* = 13.6, 7.9 Hz, 1H), 1.67 (s, 1H). **^{13}C NMR** (101 MHz, $CDCl_3$) δ 140.3, 135.4, 133.7, 132.5, 128.3, 128.2, 128.0, 127.8, 127.7, 126.2, 125.7, 115.2, 73.7, 44.1.

Bromide substrate synthesis

General Procedure H

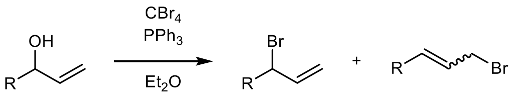

To a solution of the branched allyl alcohol (1 equiv.) in Et_2O (0.2 M) was added CBr_4 (1.2 – 1.5 equiv.) and PPh_3 (1.2 – 1.5 equiv.) at room temperature. The reaction was stirred vigorously until complete consumption of the alcohol was seen by TLC. An equal volume of hexane was added, and the mixture was filtered through Celite. The filtrate was concentrated *in vacuo* to yield a mixture of the branched and linear allyl bromide. The branched bromide substrate was purified by FCC where possible.

2-bromobut-3-en-1-yl 4-fluorobenzoate (1a)

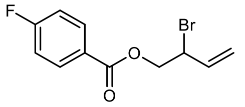

Prepared according to General Procedure H from **6a** (1.05 g, 5 mmol). Purification by FCC (98:2 pentane:Et_2O) afforded the title compound (1.06 g, 78%) as a colourless oil. **^{1}H NMR** (400 MHz, $CDCl_3$) δ 8.12 – 8.02 (m, 2H), 7.16 – 7.08 (m, 2H), 6.03 (ddd, *J* = 16.9, 10.1, 8.9 Hz, 1H), 5.40 (dt, *J* = 16.9, 0.8 Hz, 1H), 5.23 (dt, *J* = 10.1, 0.7 Hz, 1H), 4.79 – 4.70 (m, 1H),

4.61 – 4.51 (m, 2H). **^{13}C NMR** (101 MHz, $CDCl_3$) δ 166.1 (d, *J* = 254.4 Hz), 165.0, 135.4, 132.5 (d, *J* = 9.3 Hz), 126.0 (d, *J* = 3.0 Hz), 119.5, 115.8 (d, *J* = 22.1 Hz), 67.2, 49.6. **^{19}F NMR** (377 MHz, $CDCl_3$) δ -105.0 (tt, *J* = 8.5, 5.5 Hz). **IR** (liquid film) ν = 3431, 3084, 2986, 1722, 1604, 1509, 1412, 1376, 1266, 1240, 1155, 1116, 1090, 1015, 987, 933, 854, 804, 766, 739, 688 cm^{-1}. No HRMS obtained.

4-bromobut-2-en-1-yl 4-fluorobenzoate (4a)

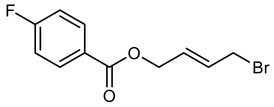

Prepared in the same reaction above (59.1 mg, 4%). **^{1}H NMR** (400 MHz, $CDCl_3$) δ 8.13 – 8.02 (m, 2H), 7.17 – 7.06 (m, 2H), 6.12 – 6.02 (m, 1H), 6.01 – 5.91 (m, 1H), 4.83 (dd, *J* = 5.5, 1.1 Hz, 2H), 3.98 (dd, *J* = 7.2, 0.8 Hz, 2H). **^{13}C NMR** (101 MHz, $CDCl_3$) δ 166.0 (d, *J* = 254.1 Hz), 165.3, 132.4 (d, *J* = 9.3 Hz), 130.3, 129.0, 126.3 (d, *J* = 3.0 Hz), 115.7 (d, *J* = 22.0 Hz), 64.2, 31.4. **^{19}F NMR** (377 MHz, $CDCl_3$) δ -105.3 (tt, *J* = 8.4, 5.4 Hz). **IR** (liquid film) ν = 2929, 1723, 1604, 1508, 1454, 1440, 1413, 1380, 1270, 1239, 1207, 1154, 1115, 1091, 1015, 968, 855, 767, 688 cm^{-1}. No HRMS obtained.

2-bromobut-3-en-1-yl 4-cyanobenzoate (1b)

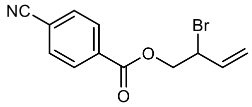

Prepared according to General Procedure H from **6b** (434 mg, 2 mmol). Purification by FCC (100:0 to 90:10 pentane:Et_2O) afforded the title compound (481 mg, 86%) as a white solid. **^{1}H NMR** (400 MHz, $CDCl_3$) δ 8.17 – 8.11 (m, 2H), 7.79 – 7.72 (m, 2H), 6.01 (ddd, *J* = 16.9, 10.1, 8.8 Hz, 1H), 5.40 (dt, *J* = 16.9, 1.0 Hz, 1H), 5.24 (d, *J* = 10.1 Hz, 1H), 4.79 – 4.70 (m, 1H), 4.63 – 4.56 (m, 2H). **^{13}C NMR** (101 MHz, $CDCl_3$) δ 164.3, 135.1, 133.5, 132.4, 130.4, 119.7, 118.0, 116.9, 67.7, 49.2. **IR** (liquid film) ν = 2980, 2231, 1723, 1610, 1460, 1373, 1313, 1272, 1178, 1123, 1110, 999, 943, 865, 768, 740, 690 cm^{-1}. **HRMS** (ESI$^+$) m/z calculated for $[M+Na]^+$ 301.9787, found 301.9779. **m.p.** 50 – 52 °C.

2-bromobut-3-en-1-yl benzoate (1c)

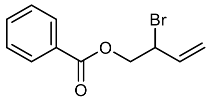

Prepared according to General Procedure H from **6c** (384 mg, 2 mmol). Purification by FCC (98:2 pentane:Et_2O) afforded the title compound (312 mg, 61%) as a colourless oil. **^{1}H NMR** (400 MHz, $CDCl_3$) δ 8.09 – 7.01 (m, 2H), 7.62 – 7.53 (m, 1H), 7.49 – 7.41 (m, 2H), 6.04 (ddd, *J* = 16.9, 10.2, 8.9 Hz, 1H), 5.40 (d, *J* = 16.9 Hz, 1H), 5.23 (d, *J* = 10.1 Hz, 1H), 4.80 – 4.71 (m, 1H), 4.63 – 4.53 (m, 2H). **^{13}C NMR** (101 MHz, $CDCl_3$) δ 166.0, 135.5, 133.4, 129.9, 129.8, 128.6, 119.4, 67.2, 49.6. **IR** (liquid film) ν = 3065, 1725, 1602, 1451, 1376, 1315, 1271, 1177, 1113, 1070, 1026, 986, 934, 710 cm^{-1}. **HRMS** (ESI$^+$) m/z calculated for $[M+Na]^+$ 276.9835, found 276.9828.

2-bromobut-3-en-1-yl 4-(*tert*-butyl)benzoate (1d)

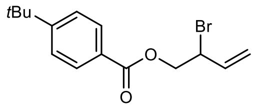

Prepared according to General Procedure H from **6d** (621 mg, 2.5 mmol). Purification by FCC (100:0 to 98:2 pentane:Et_2O) afforded the title compound (584 mg, 75%) as a colourless oil. **^{1}H NMR** (400 MHz, $CDCl_3$) δ 8.03 – 7.94 (m, 2H), 7.50 – 7.42 (m, 2H), 6.03 (ddd, *J* = 16.9, 10.1, 8.9 Hz, 1H), 5.39 (dt, *J* = 17.1, 0.9 Hz, 1H), 5.22 (d, *J* = 10.1 Hz, 1H), 4.75 (dt,

J = 8.9, 6.7 Hz, 1H), 4.62 – 4.51 (m, 2H), 1.34 (s, 9H). **^{13}C NMR** (101 MHz, $CDCl_3$) δ 166.0, 157.2, 135.5, 129.8, 126.9, 125.6, 119.3, 67.0, 49.7, 35.3, 31.2. **IR** (liquid film) ν = 2966, 1725, 1610, 1409, 1271, 1189, 1116, 1017, 932, 854, 774, 707 cm^{-1}. **HRMS** (ESI^+) m/z calculated for $[M+H]^+$ 311.0641, found 311.0636.

2-bromobut-3-en-1-yl 4-(4,4,5,5-tetraethyl-1,3,2-dioxaborolan-2-yl)benzoate (1e)

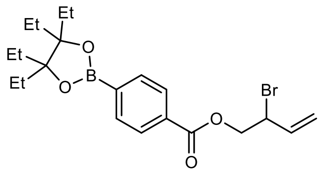

Prepared according to General Procedure H from **6e** (898 mg, 2.4 mmol). Purification by FCC (100:0 to 98:2 pentane:Et_2O) afforded the title compound (826 mg, 79%) as a colourless oil. **^{1}H NMR** (400 MHz, $CDCl_3$) δ 8.02 (d, *J* = 7.7 Hz, 2H), 7.89 (d, *J* = 7.8 Hz, 2H), 6.03 (ddd, *J* = 16.9, 10.1, 8.8 Hz, 1H), 5.39 (d, *J* = 16.9 Hz, 1H), 5.22 (d, *J* = 10.1 Hz, 1H), 4.75 (dt, *J* = 8.9, 6.7 Hz, 1H), 4.64 – 4.53 (m, 2H), 1.77 (th, *J* = 14.5, 7.4 Hz, 8H), 0.97 (t, *J* = 7.4 Hz, 12H). **^{13}C NMR** (101 MHz, $CDCl_3$) δ 166.1, 135.5, 134.9, 131.7, 128.9, 119.4, 89.4, 67.2, 49.6, 26.6, 9.0. **IR** (liquid film) ν = 2979, 1728, 1562, 1510, 1459, 1401, 1367, 1353, 1268, 1109, 1096, 1021, 922, 709 cm^{-1}. **HRMS** (ESI^+) m/z calculated for $[M+H]^+$ 437.1493, found 437.1486.

2-bromobut-3-en-1-yl 2-fluorobenzoate (1f)

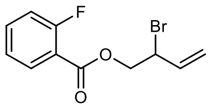

Prepared according to General Procedure H from **6f** (420 mg, 2 mmol). Purification by FCC (98:2 pentane:Et_2O) afforded the title compound (477 mg, 87%) as a colourless oil. **^{1}H NMR** (400 MHz, $CDCl_3$) δ 7.95 (td, *J* = 7.6, 1.8 Hz, 1H), 7.59 – 7.49 (m, 1H), 7.21 (td, *J* = 7.7, 1.1 Hz, 1H), 7.15 (ddd, *J* = 10.8, 8.3, 1.1 Hz, 1H), 6.04 (ddd, *J* = 16.9, 10.1, 9.0 Hz, 1H), 5.40 (d, *J* = 16.9 Hz, 1H), 5.23 (d, *J* = 10.1 Hz, 1H), 4.75 (dt, *J* = 8.9, 6.7 Hz, 1H), 4.59 (d, *J* = 6.6 Hz, 2H). **^{13}C NMR** (101 MHz, $CDCl_3$) δ 163.7 (d, *J* = 3.7 Hz), 162.3 (d, *J* = 261.0 Hz), 135.4, 135.0 (d, *J* = 9.1 Hz), 132.3, 124.2 (d, *J* = 4.1 Hz), 119.5, 118.2 (d, *J* = 9.6 Hz), 117.2 (d, *J* = 22.3 Hz), 67.4, 49.4. **^{19}F NMR** (377 MHz, $CDCl_3$) δ -108.9 (ddd, *J* = 11.4, 7.2, 4.7 Hz). **IR** (liquid film) ν = 3087, 1732, 1613, 1489, 1456, 1295, 1249, 1158, 1124, 1080, 1033, 986, 933, 755, 691 cm^{-1}. **HRMS** (ESI^+) m/z calculated for $[M+Na]^+$ 294.9740, found 294.9755.

3-bromopent-4-en-1-yl 4-fluorobenzoate (1g)

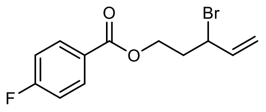

Prepared according to General Procedure H from **6g** (561 mg, 2.5 mmol). Purification by FCC (98:2 pentane:Et_2O) afforded the title compound (577 mg, 80%) as a colourless oil. **^{1}H NMR** (400 MHz, $CDCl_3$) δ 8.10 – 8.01 (m, 2H), 7.17 – 7.07 (m, 2H), 6.05 (ddd, *J* = 16.8, 10.1, 8.9 Hz, 1H), 5.28 (dt, *J* = 16.9, 0.9 Hz, 1H), 5.12 (d, *J* = 10.0 Hz, 1H), 4.70 – 4.60 (m, 1H), 4.51 – 4.39 (m, 2H), 2.44 – 2.30 (m, 2H). **^{13}C NMR** (101 MHz, $CDCl_3$) δ 166.0 (d, *J* = 254.2 Hz), 165.5, 138.5, 132.3 (d, *J* = 9.4 Hz), 126.4 (d, *J* = 3.0 Hz), 117.2, 115.7 (d, *J* = 22.0 Hz), 62.9, 51.0, 37.6. **^{19}F NMR** (377 MHz, $CDCl_3$) δ -105.4 (tt, *J* = 8.3, 5.5 Hz). **IR** (liquid film) ν = 2982, 1721, 1604, 1508, 1412, 1275, 1239, 1154, 1115, 1091, 989, 930, 854, 767, 729, 687, 639, 609 cm^{-1}. **HRMS** (ESI^+) m/z calculated for $[M+H]^+$ 287.0077, found 287.0073.

2-bromobut-3-en-1-yl 3-phenylpropanoate (1h)

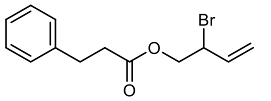

Prepared according to General Procedure H from **6h** (793 mg, 3.6 mmol). Purification by FCC (100:0 to 95:5 pentane:Et_2O) afforded the title compound (859 mg, 84%) as a colourless oil. **^{1}H NMR** (400 MHz, $CDCl_3$) δ 7.33 – 7.27 (m, 2H), 7.24 – 7.17 (m, 3H), 5.91 (ddd, *J* = 16.9, 10.1, 9.0 Hz, 1H), 5.30 (dd, *J* = 16.9, 1.0 Hz, 1H), 5.17 (d, *J* = 10.1 Hz, 1H), 4.58 (dt, *J* = 9.0, 6.8 Hz, 1H), 4.40 – 4.28 (m, 2H), 2.97 (t, *J* = 7.8 Hz, 2H), 2.68 (t, *J* = 7.8 Hz, 2H). **^{13}C NMR** (101 MHz, $CDCl_3$) δ 172.3, 140.3, 135.4, 128.7, 128.4, 126.5, 119.3, 66.7, 49.5, 35.7, 30.9. **IR** (liquid film) ν = 3028, 1742, 1496, 1454, 1420, 1382, 1290, 1234, 1157, 987, 933, 750, 699 cm^{-1}. **HRMS** (ESI^+) m/z calculated for $[M+Na]^+$ 305.0148, found 305.0141.

4-((2-bromobut-3-en-1-yl)oxy)-1,1'-biphenyl (1i)

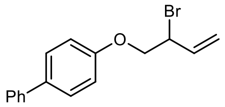

Prepared according to General Procedure H from **6i** (706 mg, 2.94 mmol). Purification by FCC (99:1 pentane:Et_2O) afforded the title compound (390 mg, 52%) as a white solid. **^{1}H NMR** (400 MHz, $CDCl_3$) δ 7.58 – 7.51 (m, 4H), 7.46 – 7.39 (m, 2H), 7.36 – 7.29 (m, 1H), 7.03 – 6.96 (m, 2H), 6.08 (ddd, *J* = 16.9, 10.1, 8.9 Hz, 1H), 5.43 (dt, *J* = 16.9, 0.9 Hz, 1H), 5.26 (dt, *J* = 10.2, 0.7 Hz, 1H), 4.77 (dddt, *J* = 8.7, 6.8, 5.9, 0.8 Hz, 1H), 4.34 (dd, *J* = 10.2, 6.0 Hz, 1H), 4.26 (dd, *J* = 10.3, 7.1 Hz, 1H). **^{13}C NMR** (101 MHz, $CDCl_3$) δ 157.7, 140.8, 135.8, 134.8, 128.9, 128.4, 127.0, 126.9, 119.3, 115.3, 71.6, 50.2. **IR** (liquid film) ν = 2919, 1604, 1583, 1522, 1488, 1455, 1420, 1390, 1269, 1246, 1202, 1078, 1044, 990, 966, 944, 879, 835, 763, 716, 692 cm^{-1}. **HRMS** (APCI) m/z calculated for $[M+H]^+$ 303.0379, found 303.0379. **m.p.** 60 – 61 °C.

1-((2-bromobut-3-en-1-yl)oxy)-4-methoxybenzene (1j)

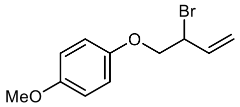

Prepared according to General Procedure H from **6j** (583 mg, 3 mmol). Purification by FCC (100:0 to 98:2 pentane:Et_2O) afforded the title compound (425 mg, 55%) as a colourless oil. **^{1}H NMR** (400 MHz, $CDCl_3$) δ 6.90 – 6.80 (m, 4H), 6.05 (dt, *J* = 16.9, 9.5 Hz, 1H), 5.39 (d, *J* = 16.9 Hz, 1H), 5.23 (d, *J* = 10.1 Hz, 1H), 4.71 (dt, *J* = 8.9, 6.6 Hz, 1H), 4.23 (dd, *J* = 10.3, 6.0 Hz, 1H), 4.16 (dd, *J* = 10.4, 7.1 Hz, 1H), 3.77 (s, 3H). **^{13}C NMR** (101 MHz, $CDCl_3$) δ 154.6, 152.3, 135.9, 119.2, 116.4, 114.8, 72.5, 55.8, 50.4. **IR** (liquid film) ν = 2833, 1507, 1464, 1441, 1420, 1229, 1181, 1107, 1039, 987, 932, 824, 771, 747, 714, 638 cm^{-1}. **HRMS** (ESI^+) m/z calculated for $[M+Na]^+$ 278.9991, found 278.9981.

(((3-bromopent-4-en-1-yl)oxy)methyl)benzene (1k)

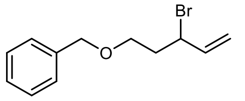

Prepared according to General Procedure H from **6k** (581 mg, 2.8 mmol). Purification by FCC (100:0 to 98:2 pentane:Et_2O) afforded the title compound (550 mg, 77%) as a colourless oil. **^{1}H NMR** (400 MHz, $CDCl_3$) δ 7.42 – 7.27 (m, 5H), 6.02 (dt, *J* = 17.0, 9.5 Hz, 1H), 5.24 (d, *J* = 17.0 Hz, 1H), 5.07 (d, *J* = 10.0 Hz, 1H), 4.73 (q, *J* = 7.9 Hz, 1H), 4.57 – 4.48 (m, 2H), 3.68 – 3.55 (m, 2H), 2.26 – 2.11 (m, 2H). **^{13}C NMR** (101 MHz, $CDCl_3$) δ 139.1, 138.3, 128.5, 127.84, 127.82, 116.6, 73.3, 67.8, 52.3, 38.7. **IR** (liquid film) ν = 3086, 3064, 3030, 2980, 2863, 1495, 1454, 1419,

1363, 1172, 1104, 1028, 988, 928, 735, 697 cm^{-1}. **HRMS** (ESI^+) m/z calculated for $[M+NH_4]^+$ 272.0645, found 272.0638.

2-bromobut-3-en-1-yl 4-methylbenzenesulfonate (1l)

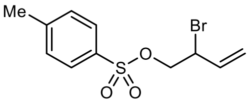

Prepared according to General Procedure H from **6l** (969 mg, 4 mmol). Purification by FCC (90:10 hexane:Et_2O) afforded the title compound (891 mg, 73%) as a colourless oil. **^{1}H NMR** (400 MHz, $CDCl_3$) δ 7.83 – 7.76 (m, 2H), 7.40 – 7.31 (m, 2H), 5.82 (ddd, *J* = 16.9, 10.1, 8.9 Hz, 1H), 5.33 (dt, *J* = 16.9, 0.8 Hz, 1H), 5.21 (dt, *J* = 10.1, 0.7 Hz, 1H), 4.58 – 4.49 (m, 1H), 4.26 (dd, *J* = 10.6, 5.9 Hz, 1H), 4.17 (dd, *J* = 10.6, 7.8 Hz, 1H), 2.46 (s, 3H). **^{13}C NMR** (101 MHz, $CDCl_3$) δ 145.4, 134.3, 132.8, 130.1, 128.1, 120.4, 71.3, 48.0, 21.8. **IR** (liquid film) ν = 2986, 1598, 1495, 1451, 1364, 1293, 1190, 1177, 1097, 1045, 987, 960, 814, 791, 736, 664 cm^{-1}. **HRMS** (ESI^+) m/z calculated for $[M+H]^+$ 304.9842, found 304.9832.

2-bromobut-3-en-1-yl 4-fluorobenzenesulfonate (1m)

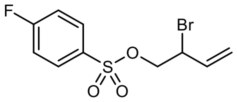

Prepared according to General Procedure H from **6m** (1.18 g, 4.8 mmol). Purification by FCC (90:10 pentane:Et_2O) afforded the title compound (1.36 g, 92%) as a colourless oil. **^{1}H NMR** (400 MHz, $CDCl_3$) δ 8.00 – 7.89 (m, 2H), 7.30 – 7.19 (m, 2H), 5.83 (dt, *J* = 17.2, 9.5 Hz, 1H), 5.35 (d, *J* = 16.9 Hz, 1H), 5.22 (d, *J* = 10.1 Hz, 1H), 4.60 – 4.49 (m, 1H), 4.29 (dd, *J* = 10.6, 5.9 Hz, 1H), 4.22 (dd, *J* = 10.6, 7.6 Hz, 1H). **^{13}C NMR** (101 MHz, $CDCl_3$) δ 166.1 (d, *J* = 257.5 Hz), 134.2, 131.9 (d, *J* = 3.0 Hz), 131.0 (d, *J* = 9.6 Hz), 120.6, 116.9 (d, *J* = 23.1 Hz), 71.5, 47.9. **^{19}F NMR** (377 MHz, $CDCl_3$) δ -102.4 (tt, *J* = 8.2, 4.3 Hz). **IR** (liquid film) ν = 2981, 1593, 1495, 1421, 1368, 1295, 1242, 1186, 1158, 1096, 986, 956, 840, 668 cm^{-1}. **HRMS** (ESI^+) m/z calculated for $[M+Na]^+$ 330.9410, found 330.9406.

2-bromobut-3-en-1-yl diphenylphosphinate (1n)

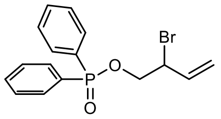

Prepared according to General Procedure H from **6n** (1.01 g, 3.5 mmol). Purification by FCC (Et_2O) afforded the title compound (963 mg, 78%) as a colourless oil. **^{1}H NMR** (400 MHz, $CDCl_3$) δ 7.88 – 7.76 (m, 4H), 7.58 – 7.51 (m, 2H), 7.50 – 7.41 (m, 4H), 5.96 (ddd, *J* = 16.9, 10.1, 9.0 Hz, 1H), 5.37 (d, *J* = 17.1 Hz, 1H), 5.22 (d, *J* = 10.2 Hz, 1H), 4.66 (dt, *J* = 9.0, 6.6 Hz, 1H), 4.29 – 4.17 (m, 2H). **^{13}C NMR** (101 MHz, $CDCl_3$) δ 135.2, 132.6 (d, *J* = 3.0 Hz), 131.8 (d, *J* = 10.3 Hz), 131.0 (d, *J* = 136.8 Hz), 128.8 (d, *J* = 13.2 Hz), 119.8, 66.8 (d, *J* = 5.4 Hz), 51.0 (d, *J* = 7.7 Hz). **^{31}P NMR** (162 MHz, $CDCl_3$) δ 32.4. **IR** (liquid film) ν = 3059, 2980, 1592, 1439, 1382, 1229, 1184, 1131, 1113, 1071, 1019, 996, 934, 840, 754, 730, 695 cm^{-1}. **HRMS** (ESI^+) m/z calculated for $[M+H]^+$ 351.0144, found 351.0140.

((2-bromobut-3-en-1-yl)oxy)(*tert*-butyl)diphenylsilane (1o)

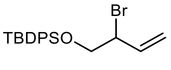

Prepared according to General Procedure H from **6o** (980 mg, 3 mmol). Purification by FCC (100:0 to 98:2 pentane:Et_2O) afforded the title compound (756 mg, 65%) as a colourless oil. **^{1}H NMR** (400 MHz, $CDCl_3$) δ 7.72 – 7.63 (m, 4H), 7.49 – 7.35 (m, 6H), 5.97 (ddd, *J* = 16.9, 10.1, 9.1 Hz,

1H), 5.31 (dt, *J* = 16.9, 1.0 Hz, 1H), 5.17 (dd, *J* = 10.1, 1.2 Hz, 1H), 4.52 – 4.44 (m, 1H), 3.92 (dd, *J* = 10.8, 5.8 Hz, 1H), 3.84 (dd, *J* = 10.8, 6.9 Hz, 1H), 1.07 (s, 9H). **^{13}C NMR** (101 MHz, $CDCl_3$) δ 136.6, 135.80, 135.76, 133.31, 133.26, 130.0, 127.90, 127.88, 118.7, 67.6, 54.4, 26.9, 19.5. **IR** (liquid film) ν = 3071, 2958, 2931, 2858, 1589, 1472, 1428, 1391, 1362, 1258, 1187, 1113, 1068, 986, 928, 824, 807, 741, 702, 613 cm^{-1}. **HRMS** (ESI^+) m/z calculated for $[M+H]^+$ 389.0931, found 389.0928.

2-bromobut-3-en-1-yl diphenylcarbamate (1p)

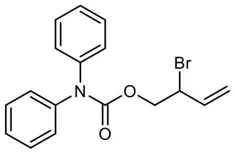

Prepared according to General Procedure H from **6p** (283 mg, 1 mmol). Purification by FCC (100:0 to 90:10 pentane:Et_2O) afforded an inseparable mixture of the title compound and the isomeric 1-bromobut-3-en-2-yl diphenylcarbamate (7:1, 282 mg, 81%) as a colourless oil. The spectroscopic data refer to the title compound. **^{1}H NMR** (400 MHz, $CDCl_3$) δ 7.39 – 7.30 (m, 4H), 7.29 – 7.17 (m, 6H), 5.88 (ddd, *J* = 16.9, 10.1, 9.1 Hz, 1H), 5.29 (dt, *J* = 16.9, 0.9 Hz, 1H), 5.16 (d, *J* = 10.1 Hz, 1H), 4.61 (dt, *J* = 9.1, 6.7 Hz, 1H), 4.47 – 4.34 (m, 2H). **^{13}C NMR** (101 MHz, $CDCl_3$) δ 154.0, 142.3, 135.5, 129.0, 127.1, 126.5, 119.3, 68.2, 49.9. **IR** (liquid film) ν = 2980, 1717, 1593, 1492, 1453, 1383, 1336, 1303, 1283, 1210, 1057, 1026, 987, 933, 761, 696 cm^{-1}. **HRMS** (ESI^+) m/z calculated for $[M+H]^+$ 346.0437, found 346.0433.

2-(3-bromopent-4-en-1-yl)isoindoline-1,3-dione (1q)

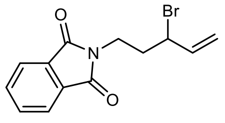

Prepared according to General Procedure H from **6q** (231 mg, 1 mmol). Purification by FCC (80:20 pentane:Et_2O) afforded the title compound (190 mg, 65%) as a white solid. **^{1}H NMR** (400 MHz, $CDCl_3$) δ 7.85 (dd, *J* = 5.5, 3.1 Hz, 2H), 7.72 (dd, *J* = 5.5, 3.1 Hz, 2H), 6.01 (ddd, *J* = 16.9, 10.1, 8.8 Hz, 1H), 5.29 (dt, *J* = 17.0, 0.9 Hz, 1H), 5.10 (d, *J* = 10.1 Hz, 1H), 4.51 (dt, *J* = 8.9, 7.1 Hz, 1H), 3.88 – 3.73 (m, 2H), 2.38 – 2.25 (m, 2H). **^{13}C NMR** (101 MHz, $CDCl_3$) δ 168.3, 138.4, 134.2, 132.2, 123.5, 117.3, 51.4, 37.1, 36.4. **IR** (liquid film) ν = 2927, 2857, 1769, 1710, 1466, 1435, 1398, 1369, 1336, 1202, 1143, 1104, 1088, 1046, 992, 969, 936, 888, 800, 775, 743, 717, 623 cm^{-1}. **HRMS** (ESI^+) m/z calculated for $[M+H]^+$ 294.0124, found 294.0118. **m.p.** 70 – 71 °C.

(3-bromopent-4-en-1-yl)(naphthalen-2-yl)sulfane (1r)

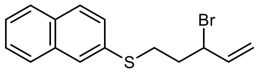

Prepared according to General Procedure H from **6r** (367 mg, 1.5 mmol). Purification by FCC (pentane) afforded an inseparable mixture of the title compound and the linear isomer (11:1, 261 mg, 57%) as a colourless oil. The spectroscopic data refer to the title compound. **^{1}H NMR** (400 MHz, $CDCl_3$) δ 7.84 – 7.71 (m, 4H), 7.53 – 7.39 (m, 3H), 5.99 (ddd, *J* = 16.9, 10.0, 8.9 Hz, 1H), 5.26 (dt, *J* = 16.9, 0.9 Hz, 1H), 5.10 (d, *J* = 10.0 Hz, 1H), 4.74 – 4.63 (m, 1H), 3.24 – 3.05 (m, 2H), 2.35 – 2.11 (m, 2H). **^{13}C NMR** (101 MHz, $CDCl_3$) δ 138.5, 133.9, 133.1, 132.0, 128.7, 127.9, 127.7, 127.6, 127.2, 126.8, 126.0, 117.2, 53.6, 37.8, 31.6. **IR** (liquid film) ν = 3053, 1624, 1590, 1501, 1420, 1133, 1070, 988, 927, 851, 811, 743 cm^{-1}. No HRMS obtained.

1-bromo-4-(3-bromopent-4-en-1-yl)benzene (1s)

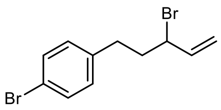

Prepared according to General Procedure H from **6s** (603 mg, 2.5 mmol). Purification by FCC (pentane) afforded the title compound (491 mg, 65%) as a colourless oil. **^{1}H NMR** (400 MHz, $CDCl_3$) δ 7.45 – 7.37 (m, 2H), 7.11 – 7.03 (m, 2H), 6.02 (ddd, *J* = 16.7, 10.1, 8.9 Hz, 1H), 5.21 (dd, *J* = 16.9, 1.0 Hz, 1H), 5.09 (d, *J* = 10.1 Hz, 1H), 4.40 (td, *J* = 8.5, 6.0 Hz, 1H), 2.72 (tdd, *J* = 14.1, 11.2, 7.4 Hz, 2H), 2.23 (dtd, *J* = 14.3, 8.3, 6.0 Hz, 1H), 2.18 – 2.06 (m, 1H). **^{13}C NMR** (101 MHz, $CDCl_3$) δ 139.6, 139.1, 131.7, 130.4, 120.1, 116.8, 54.4, 40.0, 33.3. **IR** (liquid film) ν = 3087, 2950, 2930, 2863, 1898, 1636, 1592, 1488, 1449, 1418, 1404, 1357, 1289, 1204, 1145, 1100, 1073, 1012, 987, 927, 841, 805, 773, 730, 681, 640 cm^{-1}. **HRMS** (GC EI) m/z calculated for $[M]^{+\cdot}$ 301.93003, found 301.93058.

4-(3-bromopent-4-en-1-yl)-1,2-dimethoxybenzene (1t)

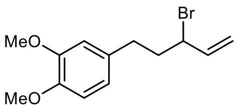

Prepared according to General Procedure H from **6t** (445 mg, 2 mmol). Purification by FCC (95:5 pentane:Et_2O) afforded an inseparable mixture of the title compound and the linear isomer (8:1, 269 mg, 47%) as a yellow oil. The spectroscopic data refer to the title compound. **^{1}H NMR** (400 MHz, $CDCl_3$) δ 6.80 (d, *J* = 8.0 Hz, 1H), 6.77 – 6.67 (m, 2H), 6.03 (ddd, *J* = 16.9, 10.0, 8.9 Hz, 1H), 5.22 (dt, *J* = 17.0, 1.0 Hz, 1H), 5.08 (d, *J* = 10.1 Hz, 1H), 4.43 (td, *J* = 8.5, 6.1 Hz, 1H), 3.88 (s, 3H), 3.86 (s, 3H), 2.81 – 2.61 (m, 2H), 2.31 – 2.07 (m, 2H). **^{13}C NMR** (101 MHz, $CDCl_3$) δ 149.0, 147.6, 139.3, 133.2, 120.5, 116.6, 112.0, 111.5, 56.1, 56.0, 54.9, 40.4, 33.4. **IR** (liquid film) ν = 2998, 2952, 2936, 2834, 1607, 1591, 1516, 1464, 1453, 1418, 1262, 1237, 1158, 1142, 1030, 989, 928, 855, 807, 765, 636 cm^{-1}. **HRMS** (ESI^+) m/z calculated for $[M+H]^+$ 285.0485, found 285.0485.

(3-bromopent-4-ene-1,1-diyl)dibenzene (1u)

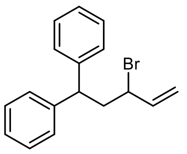

Prepared according to General Procedure H from **6u** (238 mg, 1 mmol). Purification by FCC (pentane) afforded the title compound (212 mg, 70%) as a yellow oil. **^{1}H NMR** (400 MHz, CD_2Cl_2) δ 7.35 – 7.16 (m, 10H), 6.05 (dt, *J* = 16.8, 9.6 Hz, 1H), 5.16 – 5.05 (m, 2H), 4.25 (td, *J* = 8.6, 6.6 Hz, 1H), 4.19 (t, *J* = 7.8 Hz, 1H), 2.74 – 2.53 (m, 2H). **^{13}C NMR** (101 MHz, CD_2Cl_2) δ 144.1, 143.8, 139.4, 129.1, 129.0, 128.2, 128.1, 127.0, 126.9, 117.0, 54.0, 49.5, 44.3. **IR** (liquid film) ν = 3085, 3062, 3027, 1600, 1494, 1451, 1419, 1220, 1156, 1086, 1032, 987, 929, 788, 754, 701, 633 cm^{-1}. No HRMS obtained.

((4-(3-bromopent-4-en-1-yl)phenyl)ethynyl)trimethylsilane (1v)

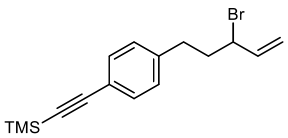

Prepared according to General Procedure H from **6v** (414 mg, 1.6 mmol). Purification by FCC (hexane) afforded the title compound (387 mg, 75%) as a colourless oil. **^{1}H NMR** (400 MHz, $CDCl_3$) δ 7.43 – 7.35 (m, 2H), 7.13 (d, *J* = 7.9 Hz, 2H), 6.01 (ddd, *J* = 17.0, 10.1, 9.0 Hz, 1H), 5.20 (d, *J* = 16.9 Hz, 1H), 5.08 (d, *J* = 10.0 Hz, 1H), 4.38 (td, *J* = 8.5, 6.0 Hz, 1H), 2.84 – 2.67 (m, 2H), 2.24 (dtd, *J* = 14.4, 8.3, 6.0 Hz, 1H), 2.13 (ddt, *J* = 14.6, 8.8, 6.5 Hz, 1H), 0.25 (s, 9H). **^{13}C NMR** (101 MHz, $CDCl_3$) δ 141.3, 139.1, 132.3, 128.6, 121.1, 116.8, 105.2, 93.9, 54.5,

39.9, 33.8, 0.1. **IR** (liquid film) ν = 2960, 2158, 1636, 1506, 1409, 1250, 1223, 1102, 987, 927, 865, 842, 760, 726, 700, 637 cm^{-1}. **HRMS** (GC EI) m/z calculated for $[M]^{+\cdot}$ 320.05904, found 320.06124.

***tert*-butyl 4-(2-bromobut-3-en-1-yl)piperidine-1-carboxylate (1w)**

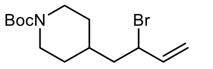

Prepared according to General Procedure H from **6w** (638 mg, 2.5 mmol). Purification by FCC (100:0 to 90:10 pentane:Et_2O) afforded the title compound (501 mg, 63%) as a colourless oil. **^{1}H NMR** (400 MHz, $CDCl_3$) δ 5.98 (ddd, *J* = 17.0, 10.0, 9.1 Hz, 1H), 5.22 (dt, *J* = 17.0, 0.9 Hz, 1H), 5.06 (d, *J* = 10.0 Hz, 1H), 4.60 – 4.50 (m, 1H), 4.12 – 4.03 (m, 2H), 2.68 (tt, *J* = 12.7, 3.0 Hz, 1H), 1.98 – 1.86 (m, 1H), 1.77 – 1.59 (m, 4H), 1.45 (s, 9H), 1.22 – 1.00 (m, 2H). **^{13}C NMR** (101 MHz, $CDCl_3$) δ 154.9, 139.4, 116.4, 79.5, 53.0, 45.2, 43.9 (2C), 34.4, 31.9, 31.4, 28.6. **IR** (liquid film) ν = 2976, 2935, 1691, 1419, 1366, 1278, 1245, 1159, 1114, 966, 932, 865, 738 cm^{-1}. **HRMS** (ESI^+) m/z calculated for $[M+H]^+$ 318.1063, found 318.1059.

***tert*-butyl 3-(1-bromoallyl)azetidine-1-carboxylate (1x)**

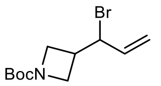

The allyl alcohol was prepared according to General Procedure F from *tert*-butyl 3-formylazetidine-1-carboxylate (1.48 g, 8 mmol). The crude product was directly subjected to General Procedure H. Purification by FCC (95:5 to 90:10 pentane:EtOAc) afforded the title compound (482 mg, 34% over two steps) as a colourless oil. **^{1}H NMR** (400 MHz, $CDCl_3$) δ 5.91 (ddd, *J* = 16.9, 10.1, 8.9 Hz, 1H), 5.27 (dt, *J* = 17.0, 0.9 Hz, 1H), 5.15 (d, *J* = 10.1 Hz, 1H), 4.56 (t, *J* = 9.3 Hz, 1H), 4.02 (t, *J* = 8.6 Hz, 1H), 3.91 (t, *J* = 8.7 Hz, 1H), 3.73 (dd, *J* = 9.0, 5.5 Hz, 1H), 3.56 (dd, *J* = 9.1, 5.4 Hz, 1H), 3.00 – 2.88 (m, 1H), 1.43 (s, 9H). **^{13}C NMR** (101 MHz, $CDCl_3$) δ 156.3, 136.1, 118.4, 79.8, 56.7, 53.8, 52.3, 35.5, 28.5. **IR** (liquid film) ν = 2977, 2885, 1702, 1479, 1403, 1366, 1299, 1254, 1144, 988, 929, 860, 771, 720, 643 cm^{-1}. **HRMS** (ESI^+) m/z calculated for $[M+H]^+$ 276.0594, found 276.0592.

2-(3-bromopent-4-en-1-yl)-5-methylfuran (1y)

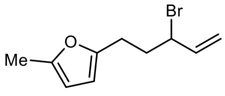

Prepared according to General Procedure H from **6y** (914 mg, 5.5 mmol). Purification by FCC (pentane) afforded the title compound (1.07 g, 85%) as a yellow oil. **^{1}H NMR** (400 MHz, $CDCl_3$) δ 6.02 (ddd, *J* = 16.9, 10.1, 8.9 Hz, 1H), 5.90 (d, *J* = 3.0 Hz, 1H), 5.87 – 5.82 (m, 1H), 5.24 (dt, *J* = 16.9, 0.9 Hz, 1H), 5.09 (dt, *J* = 10.1, 0.6 Hz, 1H), 4.48 (td, *J* = 8.5, 6.6 Hz, 1H), 2.82 – 2.65 (m, 2H), 2.32 – 2.12 (m, 5H). **^{13}C NMR** (101 MHz, $CDCl_3$) δ 152.4, 150.8, 139.1, 116.8, 106.4, 106.0, 54.6, 37.1, 26.4, 13.7. **IR** (liquid film) ν = 3088, 2951, 2920, 1618, 1570, 1432, 1383, 1289, 1218, 1179, 1148, 1066, 1021, 988, 929, 782, 727, 682, 654, 629 cm^{-1}. **HRMS** (APCI) m/z calculated for $[M+H]^+$ 229.0223, found 229.0224.

***tert*-butyl 3-(3-bromopent-4-en-1-yl)-1*H*-indole-1-carboxylate (1z)**

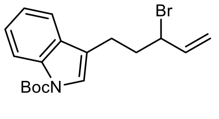

Prepared according to General Procedure H from **6z** (482 g, 1.6 mmol). Purification by FCC (100:0 to 98:2 pentane:Et_2O) afforded an inseparable mixture of the title compound and the linear isomer (10:1, 464 mg, 80%) as a white solid. The spectroscopic data refer to the title compound. **^{1}H NMR** (400 MHz, $CDCl_3$) δ 8.14 (d, *J* = 8.2 Hz, 1H), 7.54 (d, *J* = 7.8 Hz, 1H), 7.41

(s, 1H), 7.37 – 7.30 (m, 1H), 7.30 – 7.21 (m, 1H), 6.07 (ddd, *J* = 17.0, 10.0, 8.8 Hz, 1H), 5.25 (d, *J* = 16.9 Hz, 1H), 5.11 (d, *J* = 10.1 Hz, 1H), 4.54 (td, *J* = 8.5, 6.0 Hz, 1H), 2.97 – 2.75 (m, 2H), 2.41 – 2.19 (m, 2H), 1.68 (s, 9H). **^{13}C NMR** (101 MHz, $CDCl_3$) δ 149.9, 139.1, 135.6, 130.5, 124.5, 122.9, 122.5, 119.4, 119.0, 116.8, 115.4, 83.6, 55.0, 38.1, 28.4, 23.1. **IR** (liquid film) ν = 2977, 2933, 1726, 1607, 1455, 1377, 1270, 1257, 1215, 1157, 1081, 921, 857, 767, 749, 642 cm^{-1}. **HRMS** (ESI^+) m/z calculated for $[M+H]^+$ 364.0907, found 364.0902. **m.p.** 46 – 48 °C.

2-(3-bromopent-4-en-1-yl)benzo[*b*]thiophene (1aa)

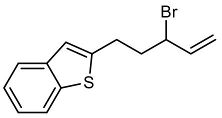

Prepared according to General Procedure H from **6aa** (262 mg, 1.2 mmol). Purification by FCC (hexane) afforded the title compound (236 mg, 70%) as a colourless oil. **^{1}H NMR** (400 MHz, $CDCl_3$) δ 7.81 – 7.74 (m, 1H), 7.72 – 7.65 (m, 1H), 7.37 – 7.27 (m, 2H), 7.07 (q, *J* = 1.0 Hz, 1H), 6.04 (ddd, *J* = 17.0, 10.1, 8.9 Hz, 1H), 5.25 (dt, *J* = 16.9, 0.9 Hz, 1H), 5.11 (d, *J* = 10.1 Hz, 1H), 4.51 (td, *J* = 8.5, 5.9 Hz, 1H), 3.15 – 3.03 (m, 2H), 2.44 – 2.22 (m, 2H). **^{13}C NMR** (101 MHz, $CDCl_3$) δ 144.1, 140.2, 139.5, 138.9, 124.4, 123.9, 123.0, 122.3, 121.6, 117.0, 54.1, 39.6, 28.9. **IR** (liquid film) ν = 3058, 2980, 1458, 1436, 1381, 1307, 1221, 1155, 1131, 1066, 1015, 987, 928, 909, 857, 826, 745, 727 cm^{-1}. **HRMS** (APCI) m/z calculated for $[M+H]^+$ 280.9994, found 280.9994.

(8*R*,9*S*,13*S*,14*S*)-3-((2-bromobut-3-en-1-yl)oxy)-13-methyl-6,7,8,9,11,12,13,14,15,16-decahydro-17*H*-cyclopenta[*a*]phenanthren-17-one (1ab)

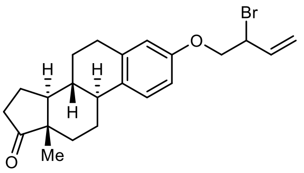

Prepared according to General Procedure H from **6ab** (510 mg, 1.5 mmol). Purification by FCC (95:5 to 80:20 pentane:Et_2O) afforded the title compound (307 mg, 51%) as a white solid. **^{1}H NMR** (400 MHz, $CDCl_3$) δ 7.20 (d, *J* = 8.5 Hz, 1H), 6.72 (dd, *J* = 8.6, 2.8 Hz, 1H), 6.66 (d, *J* = 2.7 Hz, 1H), 6.05 (ddd, *J* = 16.9, 10.2, 8.8 Hz, 1H), 5.40 (d, *J* = 16.9 Hz, 1H), 5.23 (d, *J* = 10.1 Hz, 1H), 4.72 (dt, *J* = 9.0, 6.6 Hz, 1H), 4.26 (ddd, *J* = 10.3, 6.0, 2.3 Hz, 1H), 4.18 (ddd, *J* = 10.0, 7.1, 2.2 Hz, 1H), 2.94 – 2.84 (m, 2H), 2.51 (dd, *J* = 18.8, 8.5 Hz, 1H), 2.43 – 2.36 (m, 1H), 2.30 – 2.21 (m, 1H), 2.20 – 2.11 (m, 1H), 2.11 – 1.91 (m, 3H), 1.70 – 1.36 (m, 6H), 0.91 (s, 3H). **^{13}C NMR** (101 MHz, $CDCl_3$) δ 221.0, 156.2, 138.1, 135.9, 133.1, 126.6, 119.2, 115.2, 112.6, 71.5, 50.5, 50.3, 48.1, 44.1, 38.4, 36.0, 31.7, 29.7, 26.6, 26.0, 21.7, 14.0. **IR** (liquid film) ν = 2980, 2927, 2857, 1733, 1652, 1609, 1572, 1497, 1456, 1427, 1387, 1339, 1280, 1254, 1187, 1159, 1117, 1079, 1059, 1005, 967, 934, 810, 780, 681, 636 cm^{-1}. **m.p.** 87 – 89 °C. No HRMS obtained.

2-(2-bromobut-3-en-1-yl)naphthalene (1ac)

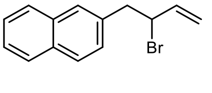

Prepared according to General Procedure H from **6ac** (750 mg, 3.8 mmol). Purification by FCC (100:0 to 98:2 pentane:Et_2O) afforded an inseparable mixture of the title compound and the linear isomer (15:1, 590 mg, 59%) as a yellow oil. The spectroscopic data refer to the title compound. **^{1}H NMR** (400 MHz, $CDCl_3$) δ 7.87 – 7.75 (m, 3H), 7.67 (s, 1H), 7.53 – 7.42 (m, 2H), 7.34 (dd, *J* = 8.4, 1.7 Hz, 1H), 6.09 (ddd, *J* = 16.8, 10.0, 9.1 Hz, 1H), 5.18 (d, *J* = 16.9 Hz, 1H), 5.05 (d, *J* = 10.1 Hz, 1H), 4.79 (dt, *J* = 9.1, 7.3 Hz, 1H), 3.50 – 3.34 (m, 2H). **^{13}C NMR** (101 MHz, $CDCl_3$) δ 138.7, 135.5, 133.5, 132.6, 128.20,

128.16, 127.82, 127.80, 127.6, 126.3, 125.8, 117.2, 55.0, 45.5. **IR** (liquid film) ν = 3053, 1634, 1600, 1508, 1419, 1367, 1271, 1205, 1146, 1047, 926, 895, 858, 816, 757, 705, 683, 646 cm^{-1}. **HRMS** (GC EI) m/z calculated for $[M]^{+\cdot}$ 260.01951, found 260.02193.

tert-butyl 3-bromo-3,6-dihydropyridine-1(2_H_)-carboxylate (1ad)

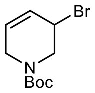

Prepared according to General Procedure H from *tert*-butyl 3-hydroxy-3,6-dihydropyridine-1(2*H*)-carboxylate (797 mg, 4 mmol). Purification by FCC (90:10 pentane:Et_2O) afforded the title compound (984 mg, 94%) as a colourless oil. **^{1}H NMR** (400 MHz, $CDCl_3$) δ 6.00 (dd, *J* = 10.2, 4.2 Hz, 1H), 5.79 (br s, 1H), 4.64 (br s, 1H), 4.35 – 3.62 (m, 4H), 1.48 (s, 9H). **^{13}C NMR** (101 MHz, $CDCl_3$, rotamers observed) δ 154.6, 128.5, 127.5, 80.5, 48.6, 47.4, 43.8, 43.3, 42.4, 28.5. **IR** (liquid film) ν = 2978, 1699, 1647, 1477, 1419, 1365, 1242, 1170, 1121, 1060, 1008, 985, 956, 909, 887, 859, 815, 768, 731 cm^{-1}. **HRMS** (ESI^+) m/z calculated for $[M+Na]^+$ 284.0257, found 284.0255.

2-chlorobut-3-en-1-yl 4-fluorobenzoate (S1)

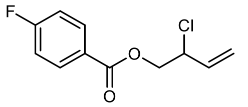

To a suspension of *N*-chlorosuccinimide (100 mg, 0.75 mmol, 1.5 equiv.) in dry THF (1.5 mL) at 0 °C under inert atmosphere was added triphenylphosphine (184 mg, 0.7 mmol, 1.4 equiv.). After 30 min at room temperature, **6a** (105 mg, 0.5 mmol) was added and stirring was continued for 3 h. The solvent was evaporated *in vacuo* and the crude mixture directly purified by FCC (98:2 pentane:Et_2O) to yield the title compound (91 mg, 80%) as a pale yellow oil. **^{1}H NMR** (400 MHz, $CDCl_3$) δ 8.11 – 8.03 (m, 2H), 7.17 – 7.08 (m, 2H), 5.95 (ddd, *J* = 16.9, 10.2, 7.9 Hz, 1H), 5.45 (dt, *J* = 16.9, 1.0 Hz, 1H), 5.31 (dt, *J* = 10.2, 0.9 Hz, 1H), 4.67 (dddt, *J* = 7.8, 6.8, 5.8, 0.9 Hz, 1H), 4.57 – 4.43 (m, 2H). **^{13}C NMR** (101 MHz, $CDCl_3$) δ 166.1 (d, *J* = 254.4 Hz), 165.1, 134.5, 132.5 (d, *J* = 9.4 Hz), 126.0 (d, *J* = 3.1 Hz), 119.6, 115.8 (d, *J* = 22.0 Hz), 67.3, 58.9. **^{19}F NMR** (377 MHz, $CDCl_3$) δ -105.0 (tt, *J* = 8.5, 5.5 Hz). **IR** (liquid film) ν = 3079, 2989, 2958, 1728, 1604, 1508, 1454, 1413, 1378, 1271, 1240, 1155, 1117, 1091, 1015, 986, 936, 855, 807, 766, 688, 608 cm^{-1}. No HRMS obtained.

2-(2,2,2-trichloro-1-iminoethoxy)but-3-en-1-yl 4-fluorobenzoate (S2)

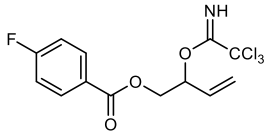

Prepared according to a literature procedure[3] on 1 mmol scale in 93% yield. All spectroscopic data were consistent with those reported in the literature. **^{1}H NMR** (400 MHz, $CDCl_3$) δ 8.43 (s, 1H), 8.12 – 7.98 (m, 2H), 7.16 – 7.04 (m, 2H), 5.96 (ddd, *J* = 16.8, 10.6, 5.6 Hz, 1H), 5.86 – 5.75 (m, 1H), 5.56 (dt, *J* = 17.4, 1.3 Hz, 1H), 5.39 (d, *J* = 10.8 Hz, 1H), 4.60 (dd, *J* = 11.9, 3.6 Hz, 1H), 4.49 (dd, *J* = 11.9, 7.3 Hz, 1H). **^{13}C NMR** (101 MHz, $CDCl_3$) δ 166.0 (d, *J* = 254.3 Hz), 165.3, 161.9, 132.4 (d, *J* = 9.5 Hz), 131.5, 126.2 (d, *J* = 3.0 Hz), 119.4, 115.7 (d, *J* = 22.2 Hz), 91.5, 76.7, 65.1. **^{19}F NMR** (377 MHz, $CDCl_3$) δ -105.2 (tt, *J* = 8.8, 5.4 Hz).

2-acetoxybut-3-en-1-yl 4-fluorobenzoate (S3)

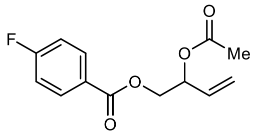

Prepared according to a literature procedure[3] on 1 mmol scale in 95% yield. All spectroscopic data were consistent with those reported in the literature. **^{1}H NMR** (400 MHz, $CDCl_3$) δ 8.03 (dd, *J* = 8.6, 5.5 Hz, 2H), 7.11 (t, *J* = 8.4 Hz, 2H), 5.94 – 5.80 (m, 1H), 5.69 – 5.61 (m, 1H), 5.42 (dt, *J* = 17.1, 1.2 Hz, 1H), 5.32 (dt, *J* = 10.5, 1.2 Hz, 1H), 4.45 (dd, *J* = 11.8, 3.8 Hz, 1H), 4.35 (dd, *J* = 11.7, 7.1 Hz, 1H), 2.10 (s, 3H). **^{13}C NMR** (101 MHz, $CDCl_3$) δ 170.2, 166.0 (d, *J* = 254.3 Hz), 165.3, 132.37 (d, *J* = 9.4 Hz), 132.35, 126.1 (d, *J* = 3.1 Hz), 119.2, 115.7 (d, *J* = 22.0 Hz), 72.1, 65.5, 21.2. **^{19}F NMR** (377 MHz, $CDCl_3$) δ -105.3 (tt, *J* = 8.5, 5.5 Hz).

General Procedure for Enantioconvergent Allylic Fluorination

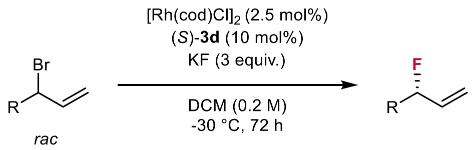

For reaction optimisation: In a 3.5 mL screw-cap vial equipped with a magnetic stirring bar were sequentially added the racemic allyl bromide (0.1 mmol, 1 equiv.), the urea catalyst (10 mol%), the fluoride source (3 equiv.), the transition metal catalyst (2.5 mol%), any other additives and the solvent (0.5 mL, 0.2 M). The vial was sealed and stirred at 1200 rpm at the corresponding temperature for a specified time. The crude mixture was passed through a short silica plug, using Et_2O as the eluent. The solvent was evaporated *in vacuo*, 4-fluoroanisole (5 µL) added as the internal standard, and the sample analysed by quantitative ^{1}H and ^{19}F NMR to determine the yield. An aliquot of the reaction mixture was purified by preparative TLC and analysed by chiral HPLC to determine the e.r.

For substrate scope (*General Procedure I*): In a microwave vial equipped with a magnetic stirring bar were sequentially added the racemic allyl bromide (1 equiv.), (*S*)-**3d** (10 mol%), pre-ground potassium fluoride (3 equiv.), $[Rh(cod)Cl]_2$ (2.5 mol%) and DCM (0.2 M). The vial was sealed, cooled to -30 °C in a cryogenic bath, and stirred at 1200 rpm for 72 h (or otherwise specified reaction temperature & time). The crude mixture was passed through a short silica plug, using Et_2O as the eluent. The solvent was evaporated *in vacuo*, and the crude directly purified by FCC. In cases where the branched/linear allyl bromide co-eluted with the fluoride product, an additional amine workup was performed to remove the allyl bromide: the crude mixture was dissolved in THF (0.2 M) in a 14 mL vial and dimethyl amine (2 M solution in THF, 5 equiv.) was added dropwise. After stirring at room temperature for 2 h, the mixture was concentrated and directly purified by FCC.

Racemic standards for all products were prepared by either (i) deoxyfluorination with (diethylamino)sulfur trifluoride (DAST) from the corresponding alcohol **6** or (ii) the general procedure above using *rac*-**3d** in EtOAc solvent. The products were purified by preparative TLC for HPLC.

Product Characterisation

(*R*)-2-fluorobut-3-en-1-yl 4-fluorobenzoate (2a)

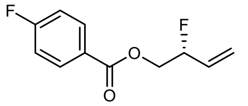

Prepared according to General Procedure I from 0.4 mmol of **1a** (109 mg). Purification by FCC (100:0 to 97:3 pentane:Et_2O) afforded the title compound (62.7 mg, 74%, 97:3 e.r.) as a colourless oil. **^{1}H NMR** (400 MHz, $CDCl_3$) δ 8.13 – 8.03 (m, 2H), 7.17 – 7.07 (m, 2H), 5.95 (dddd, *J* = 17.3, 15.1, 10.8, 5.7 Hz, 1H), 5.52 (ddt, *J* = 17.4, 2.8, 1.3 Hz, 1H), 5.40 (dt, *J* = 10.8, 1.3 Hz, 1H), 5.23 (dtdd, *J* = 48.8, 5.7, 3.0, 1.5 Hz, 1H), 4.58 – 4.33 (m, 2H). **^{13}C NMR** (101 MHz, $CDCl_3$) δ 166.1 (d, *J* = 254.4 Hz), 165.4, 132.5 (d, *J* = 9.3 Hz), 132.0 (d, *J* = 19.3 Hz), 126.0 (d, *J* = 3.0 Hz), 119.7 (d, *J* = 11.5 Hz), 115.8 (d, *J* = 21.9 Hz), 90.8 (d, *J* = 173.6 Hz), 66.1 (d, *J* = 23.3 Hz). **^{19}F NMR** (376 MHz, $CDCl_3$) δ -105.1 (tt, *J* = 8.4, 5.5 Hz), -185.9 – -186.3 (m). **IR** (liquid film) ν = 2954, 2928, 1731, 1605, 1509, 1414, 1386, 1284, 1242, 1156, 1123, 989, 942, 856, 768, 611 cm^{-1}. **HRMS** (GC EI) m/z calculated for $[M]^{+\cdot}$ 212.06434, found 212.06489. **$[\alpha]_D^{25\,°C}$** = -13.3 (c = 0.5, $CHCl_3$). **HPLC separation:** DAICEL CHIRALPAK® OJ-3, heptane:iPrOH = 99.5:0.5, 1 mL/min; t_1 = 9.7 min (major), t_2 = 10.5 min (minor).

(*R*)-2-fluorobut-3-en-1-yl 4-cyanobenzoate (2b)

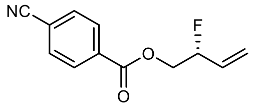

Prepared according to General Procedure I from 0.4 mmol of **1b** (112 mg). Purification by FCC (90:10 pentane:Et_2O) afforded the title compound (64.7 mg, 74%, 95:5 e.r.) as a white solid. **^{1}H NMR** (400 MHz, $CDCl_3$) δ 8.21 – 8.12 (m, 2H), 7.80 – 7.71 (m, 2H), 5.94 (dddd, *J* = 17.3, 15.2, 10.8, 5.7 Hz, 1H), 5.53 (ddt, *J* = 17.3, 2.8, 1.3 Hz, 1H), 5.41 (dt, *J* = 10.9, 1.2 Hz, 1H), 5.24 (dtdd, *J* = 48.7, 5.6, 2.9, 1.5 Hz, 1H), 4.61 – 4.38 (m, 2H). **^{13}C NMR** (101 MHz, $CDCl_3$) δ 164.7, 133.6, 132.4, 131.7 (d, *J* = 19.1 Hz), 130.4, 119.9 (d, *J* = 11.2 Hz), 118.0, 116.9, 90.5 (d, *J* = 174.1 Hz), 66.5 (d, *J* = 23.1 Hz). **^{19}F NMR** (377 MHz, $CDCl_3$) δ -186.0 – -186.4 (m). **IR** (liquid film) ν = 2961, 2924, 2231, 1723, 1610, 1407, 1312, 1280, 1178, 1109, 981, 939, 871, 767, 691 cm^{-1}. **HRMS** (ESI^+) m/z calculated for $[M+Na]^+$ 242.0588, found 242.0580. **m.p.** 44 – 47 °C. **$[\alpha]_D^{25\,°C}$** = -15.8 (c = 0.5, $CHCl_3$). **HPLC separation:** DAICEL CHIRALPAK® ID-3, heptane:iPrOH = 97:3, 1 mL/min; t_1 = 8.7 min (minor), t_2 = 13.9 min (major).

(*R*)-2-fluorobut-3-en-1-yl benzoate (2c)

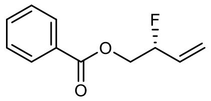

Prepared according to General Procedure I from 0.4 mmol of **1c** (102 mg). Purification by FCC (100:0 to 98:2 pentane:Et_2O) afforded the title compound (39.4 mg, 51%, 96:4 e.r.) as a colourless oil. **^{1}H NMR** (400 MHz, $CDCl_3$) δ 8.11 – 8.04 (m, 2H), 7.62 – 7.53 (m, 1H), 7.49 – 7.41 (m, 2H), 5.96 (dddd, *J* = 17.3, 15.0, 10.8, 5.7 Hz, 1H), 5.52 (ddt, *J* = 17.3, 2.8, 1.3 Hz, 1H), 5.39 (dt, *J* = 10.8, 1.3 Hz, 1H), 5.35 – 5.14 (m, 1H), 4.60 – 4.35 (m, 2H). **^{13}C NMR** (101 MHz, $CDCl_3$) δ 166.3, 133.4, 132.2 (d, *J* = 19.1 Hz), 129.9, 129.8, 128.6, 119.5 (d, *J* = 11.6 Hz), 90.8 (d, *J* = 173.6 Hz), 66.0 (d, *J* = 23.3 Hz). **^{19}F NMR** (377 MHz, $CDCl_3$) δ -185.7 – -186.3 (m). **IR** (liquid film) ν = 3065, 1725, 1602, 1452, 1431, 1382, 1315, 1274, 1178, 1118, 1070, 1027, 987, 939, 711 cm^{-1}. **$[\alpha]_D^{25\,°C}$** = -19.6 (c = 0.5, $CHCl_3$). **HPLC separation:** DAICEL CHIRALPAK® OJ-3, heptane:iPrOH = 99.5:0.5, 1 mL/min; t_1 = 11.9 min (major), t_2 = 13.9 min (minor). No HRMS obtained.

(*R*)-2-fluorobut-3-en-1-yl 4-(tert-butyl)benzoate (2d)

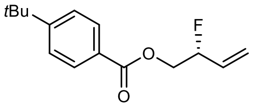

Prepared according to General Procedure I from 0.4 mmol of **1d** (124 mg). Purification by FCC (98:2 pentane:Et_2O) afforded the title compound (35.8 mg, 36%, 95:5 e.r.) as a colourless oil. **^{1}H NMR** (400 MHz, $CDCl_3$) δ 8.04 – 7.95 (m, 2H), 7.51 – 7.42 (m, 2H), 5.96 (dddd, *J* = 17.4, 15.1, 10.8, 5.7 Hz, 1H), 5.52 (ddt, *J* = 17.3, 2.9, 1.3 Hz, 1H), 5.39 (dt, *J* = 10.8, 1.3 Hz, 1H), 5.33 – 5.13 (m, 1H), 4.50 (ddd, *J* = 26.3, 12.4, 3.1 Hz, 1H), 4.41 (ddd, *J* = 20.5, 12.4, 6.9 Hz, 1H), 1.34 (s, 9H). **^{13}C NMR** (101 MHz, $CDCl_3$) δ 166.4, 157.1, 132.3 (d, *J* = 19.1 Hz), 129.8, 127.0, 125.6, 119.5 (d, *J* = 11.9 Hz), 90.8 (d, *J* = 173.3 Hz), 65.8 (d, *J* = 23.6 Hz), 35.3, 31.3. **^{19}F NMR** (376 MHz, $CDCl_3$) δ -185.8 – -186.2 (m). **IR** (liquid film) ν = 2966, 1725, 1610, 1410, 1274, 1189, 1118, 1099, 1017, 987, 938, 855, 775, 708 cm^{-1}. **HRMS** (ESI^+) m/z calculated for $[M+H]^+$ 251.1442, found 251.1440. **$[\alpha]_D^{25\,°C}$** = -9.6 (c = 0.3, $CHCl_3$). **HPLC separation:** DAICEL CHIRALPAK® OJ-3, heptane:iPrOH = 99.5:0.5, 1 mL/min; t_1 = 7.3 min (major), t_2 = 9.4 min (minor).

(*R*)-2-fluorobut-3-en-1-yl 4-(4,4,5,5-tetraethyl-1,3,2-dioxaborolan-2-yl)benzoate (2e)

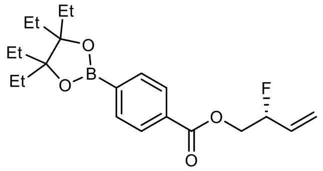

Prepared according to General Procedure I from 0.4 mmol of **1e** (175 mg). Purification by FCC (100:0 to 96:4 pentane:Et_2O) afforded the title compound (54.0 mg, 36%, 95:5 e.r.) as a yellow oil. **^{1}H NMR** (400 MHz, $CDCl_3$) δ 8.04 (d, *J* = 7.8 Hz, 2H), 7.89 (d, *J* = 7.9 Hz, 2H), 5.96 (dddd, *J* = 17.0, 15.1, 10.8, 5.7 Hz, 1H), 5.58 – 5.47 (m, 1H), 5.39 (d, *J* = 10.7 Hz, 1H), 5.35 – 5.14 (m, 1H), 4.59 – 4.34 (m, 2H), 1.77 (th, *J* = 14.6, 7.4 Hz, 8H), 0.97 (t, *J* = 7.4 Hz, 12H). **^{13}C NMR** (101 MHz, $CDCl_3$) δ 166.5, 134.9, 132.2 (d, *J* = 19.2 Hz), 131.8, 128.9, 119.6 (d, *J* = 11.6 Hz), 90.8 (d, *J* = 173.4 Hz), 89.4, 66.0 (d, *J* = 23.8 Hz), 26.6, 9.0. **^{19}F NMR** (377 MHz, $CDCl_3$) δ -185.8 – -186.2 (m). **IR** (liquid film) ν = 2980, 1728, 1562, 1510, 1459, 1401, 1367, 1353, 1267, 1112, 1096, 1021, 921, 736, 710 cm^{-1}. **HRMS** (ESI^+) m/z calculated for $[M+H]^+$ 377.2294, found 377.2288. **$[\alpha]_D^{25\,°C}$** = -10.9 (c = 0.6, $CHCl_3$). **HPLC separation:** DAICEL CHIRALPAK® ID-3, heptane:iPrOH = 98.5:1.5, 1 mL/min; t_1 = 3.2 min (minor), t_2 = 3.8 min (major).

(*R*)-2-fluorobut-3-en-1-yl 2-fluorobenzoate (2f)

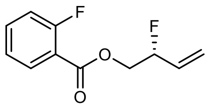

Prepared according to General Procedure I from 0.4 mmol of **1f** (109 mg). Purification by FCC (100:0 to 95:5 pentane:Et_2O) afforded the title compound (65.8 mg, 78%, 95:5 e.r.) as a colourless oil. **^{1}H NMR** (400 MHz, $CDCl_3$) δ 7.96 (td, *J* = 7.6, 1.8 Hz, 1H), 7.59 – 7.49 (m, 1H), 7.21 (t, *J* = 7.6 Hz, 1H), 7.18 – 7.11 (m, 1H), 6.05 – 5.88 (m, 1H), 5.57 – 5.47 (m, 1H), 5.40 (d, *J* = 10.8 Hz, 1H), 5.34 – 5.12 (m, 1H), 4.60 – 4.36 (m, 2H). **^{13}C NMR** (101 MHz, $CDCl_3$) δ 164.1 (d, *J* = 3.8 Hz), 162.3 (d, *J* = 260.8 Hz), 135.0 (d, *J* = 9.0 Hz), 132.3, 132.1 (d, *J* = 19.4 Hz), 124.1 (d, *J* = 3.9 Hz), 119.7 (d, *J* = 11.5 Hz), 118.3 (d, *J* = 9.6 Hz), 117.2 (d, *J* = 22.1 Hz), 90.7 (d, *J* = 173.4 Hz), 66.2 (d, *J* = 23.8 Hz). **^{19}F NMR** (377 MHz, $CDCl_3$) δ -109.0 (dt, *J* = 11.8, 6.3 Hz, 1F), -185.7 – -186.1 (m, 1F). **IR** (liquid film) ν = 3090, 2959, 1733, 1613, 1489, 1457, 1300, 1252, 1160, 1131, 1085, 1034, 987, 941, 842, 756, 692 cm^{-1}. **HRMS** (ESI^+) m/z calculated for $[M+H]^+$ 213.0722, found 213.0711. **$[\alpha]_D^{25\,°C}$** = -15.3 (c = 0.8,

$CHCl_3$). **HPLC separation**: DAICEL CHIRALPAK® OJ-3, heptane:iPrOH = 99.5:0.5, 1 mL/min; t_1 = 18.6 min (major), t_2 = 22.9 min (minor).

(*S*)-3-fluoropent-4-en-1-yl 4-fluorobenzoate (2g)

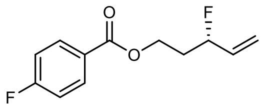

Prepared according to General Procedure I from 0.4 mmol of **1g** (115 mg). Purification by FCC (100:0 to 97:3 pentane:Et_2O) afforded the title compound (76.8 mg, 85%, 83:17 e.r.) as a colourless oil. **^{1}H NMR** (400 MHz, $CDCl_3$) δ 8.10 – 8.00 (m, 2H), 7.17 – 7.07 (m, 2H), 5.94 (dddd, *J* = 16.9, 14.2, 10.7, 5.9 Hz, 1H), 5.39 (ddt, *J* = 17.2, 3.5, 1.3 Hz, 1H), 5.28 (dt, *J* = 10.7, 1.3 Hz, 1H), 5.21 – 4.99 (m, 1H), 4.49 – 4.43 (m, 2H), 2.26 – 2.02 (m, 2H). **^{13}C NMR** (101 MHz, $CDCl_3$) δ 166.0 (d, *J* = 253.9 Hz), 165.6, 135.8 (d, *J* = 19.4 Hz), 132.3 (d, *J* = 9.3 Hz), 126.5 (d, *J* = 3.1 Hz), 117.8 (d, *J* = 11.9 Hz), 115.7 (d, *J* = 21.9 Hz), 90.6 (d, *J* = 168.3 Hz), 61.0 (d, *J* = 5.2 Hz), 34.6 (d, *J* = 22.6 Hz). **^{19}F NMR** (377 MHz, $CDCl_3$) δ -105.5 (tt, *J* = 8.6, 5.4 Hz), -180.0 – -180.4 (m). **IR** (liquid film) ν = 3084, 2980, 1722, 1604, 1508, 1412, 1277, 1239, 1155, 1117, 1091, 1015, 990, 941, 855, 805, 767, 688, 635, 609 cm^{-1}. **HRMS** (ESI^+) m/z calculated for $[M+Na]^+$ 249.0698, found 249.0693. **$[\alpha]_D^{25\ °C}$** = -5.2 (c = 1.0, $CHCl_3$). **HPLC separation:** DAICEL CHIRALPAK® ID-3, heptane:iPrOH = 99.5:0.5, 0.5 mL/min; t_1 = 11.5 min (major), t_2 = 12.5 min (minor).

(*R*)-2-fluorobut-3-en-1-yl 3-phenylpropanoate (2h)

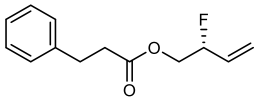

Prepared according to General Procedure I from 0.4 mmol of **1h** (113 mg). Purification by FCC (100:0 to 97:3 pentane:Et_2O) afforded the title compound (67.0 mg, 75%, 97:3 e.r.) as a yellow oil. **^{1}H NMR** (400 MHz, $CDCl_3$) δ 7.34 – 7.27 (m, 2H), 7.24 – 7.16 (m, 3H), 5.84 (dddd, *J* = 17.4, 15.1, 10.8, 5.7 Hz, 1H), 5.45 (ddt, *J* = 17.3, 2.8, 1.3 Hz, 1H), 5.34 (dt, *J* = 10.8, 1.2 Hz, 1H), 5.18 – 4.96 (m, 1H), 4.33 – 4.11 (m, 2H), 2.97 (t, *J* = 7.8 Hz, 2H), 2.70 (t, *J* = 7.8 Hz, 2H). **^{13}C NMR** (101 MHz, $CDCl_3$) δ 172.7, 140.4, 132.0 (d, *J* = 19.5 Hz), 128.7, 128.4, 126.5, 119.5 (d, *J* = 11.4 Hz), 90.7 (d, *J* = 173.0 Hz), 65.5 (d, *J* = 23.2 Hz), 35.8, 31.0. **^{19}F NMR** (377 MHz, $CDCl_3$) δ -185.8 – -186.2 (m). **IR** (liquid film) ν = 2924, 1742, 1497, 1454, 1162, 990, 939, 751, 700 cm^{-1}. **HRMS** (ESI^+) m/z calculated for $[M+Na]^+$ 245.0948, found 245.0943. **$[\alpha]_D^{25\ °C}$** = -14.9 (c = 0.7, $CHCl_3$). **HPLC separation:** DAICEL CHIRALPAK® ID-3, heptane:iPrOH = 98:2, 1 mL/min; t_1 = 4.4 min (minor), t_2 = 7.0 min (major).

(*R*)-4-((2-fluorobut-3-en-1-yl)oxy)-1,1'-biphenyl (2i)

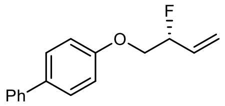

Prepared according to General Procedure I from 0.4 mmol of **1i** (121 mg). Purification by FCC (99:1 pentane:Et_2O) afforded the title compound (50.0 mg, 52%, 99:1 e.r.) as a white solid. **^{1}H NMR** (400 MHz, $CDCl_3$) δ 7.60 – 7.49 (m, 4H), 7.47 – 7.38 (m, 2H), 7.36 – 7.28 (m, 1H), 7.06 – 6.97 (m, 2H), 6.03 (dddd, *J* = 17.4, 14.8, 10.8, 5.8 Hz, 1H), 5.54 (ddt, *J* = 17.3, 2.9, 1.3 Hz, 1H), 5.42 (dt, *J* = 10.8, 1.3 Hz, 1H), 5.39 – 5.19 (m, 1H), 4.25 – 4.07 (m, 2H). **^{13}C NMR** (101 MHz, $CDCl_3$) δ 158.1, 140.8, 134.6, 132.6 (d, *J* = 19.2 Hz), 128.9, 128.4, 126.93, 126.91, 119.4 (d, *J* = 11.5 Hz), 115.1, 91.3 (d, *J* = 172.9 Hz), 70.1 (d, *J* = 24.0 Hz). **^{19}F NMR** (377 MHz, $CDCl_3$) δ -185.0 – -185.6 (m). **IR** (liquid film) ν = 2923, 1605, 1584, 1524, 1488, 1449, 1402, 1358, 1283, 1269, 1245, 1206, 1182, 1131,

1049, 1001, 965, 930, 888, 838, 766, 716, 692, 673, 637, 613 cm^{-1}. **HRMS** (APCI) m/z calculated for $[M+H]^+$ 243.1180, found 243.1179. **m.p.** 99 – 101 °C. **$[\alpha]_D^{25\ °C}$** = -16.5 (c = 0.5, $CHCl_3$). **HPLC separation:** DAICEL CHIRALPAK® IB-3, heptane:iPrOH = 99.5:0.5, 1 mL/min; t_1 = 9.1 min (major), t_2 = 15.0 min (minor).

(*R*)-1-((2-fluorobut-3-en-1-yl)oxy)-4-methoxybenzene (2j)

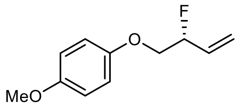

Prepared according to General Procedure I from 0.4 mmol of **1j** (103 mg) at room temperature for 24 h. Purification by FCC (100:0 to 98:2 pentane:Et_2O) afforded the title compound (57.3 mg, 73%, 95:5 e.r.) as a yellow oil. **^{1}H NMR** (400 MHz, $CDCl_3$) δ 6.91 – 6.79 (m, 4H), 5.99 (dddd, *J* = 16.9, 14.9, 10.8, 5.8 Hz, 1H), 5.50 (ddt, *J* = 17.3, 3.0, 1.4 Hz, 1H), 5.38 (dd, *J* = 10.7, 1.4 Hz, 1H), 5.33 – 5.13 (m, 1H), 4.15 – 3.98 (m, 2H), 3.77 (s, 3H). **^{13}C NMR** (101 MHz, $CDCl_3$) δ 154.4, 152.7, 132.7 (d, *J* = 19.3 Hz), 119.2 (d, *J* = 11.6 Hz), 116.0, 114.8, 91.5 (d, *J* = 172.7 Hz), 70.9 (d, *J* = 23.9 Hz), 55.9. **^{19}F NMR** (377 MHz, $CDCl_3$) δ -185.2 – -185.7 (m). **IR** (liquid film) ν = 2945, 2835, 1508, 1455, 1289, 1232, 1181, 1108, 1049, 990, 940, 880, 825, 773, 744, 670 cm^{-1}. **HRMS** (ESI^+) m/z calculated for $[M+Na]^+$ 219.0792, found 219.0790. **$[\alpha]_D^{25\ °C}$** = -12.1 (c = 0.6, $CHCl_3$). **HPLC separation:** DAICEL CHIRALPAK® IB-3, heptane:iPrOH = 99:1, 1 mL/min; t_1 = 4.8 min (major), t_2 = 9.5 min (minor).

(*S*)-(((3-fluoropent-4-en-1-yl)oxy)methyl)benzene (2k)

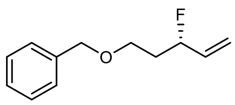

Prepared according to General Procedure I from 0.4 mmol of **1k** (102 mg). Purification by FCC (100:0 to 98:2 pentane:Et_2O) afforded the title compound (40.0 mg, 51%, 89:11 e.r.) as a colourless oil. **^{1}H NMR** (400 MHz, $CDCl_3$) δ 7.41 – 7.27 (m, 5H), 5.91 (dddd, *J* = 16.9, 14.2, 10.6, 6.0 Hz, 1H), 5.34 (ddt, *J* = 17.4, 3.2, 1.4 Hz, 1H), 5.23 (dt, *J* = 10.9, 1.3 Hz, 1H), 5.20 – 5.00 (m, 1H), 4.58 – 4.47 (m, 2H), 3.70 – 3.53 (m, 2H), 2.10 – 1.84 (m, 2H). **^{13}C NMR** (101 MHz, $CDCl_3$) δ 138.4, 136.5 (d, *J* = 19.2 Hz), 128.5, 127.8 (2C), 117.1 (d, *J* = 11.9 Hz), 91.0 (d, *J* = 166.4 Hz), 73.3, 65.9 (d, *J* = 4.8 Hz), 35.7 (d, *J* = 22.3 Hz). **^{19}F NMR** (377 MHz, $CDCl_3$) δ -180.2 (ddtd, *J* = 48.8, 30.6, 15.2, 3.8 Hz). **IR** (liquid film) ν = 3088, 3065, 3030, 2980, 2866, 1496, 1454, 1363, 1102, 1028, 990, 939, 737, 698 cm^{-1}. **HRMS** (ESI^+) m/z calculated for $[M+NH_4]^+$ 212.1445, found 212.1442. **$[\alpha]_D^{25\ °C}$** = -7.5 (c = 0.5, $CHCl_3$). **HPLC separation:** DAICEL CHIRALPAK® IB-3, heptane:iPrOH = 99.5:0.5, 1 mL/min; t_1 = 4.2 min (major), t_2 = 4.5 min (minor).

(*R*)-2-fluorobut-3-en-1-yl 4-methylbenzenesulfonate (2l)

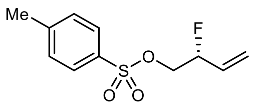

Prepared according to General Procedure I from 0.4 mmol of **1l** (122 mg) at room temperature for 24 h. Purification by FCC (100:0 to 90:10 pentane:Et_2O) afforded the title compound (69.5 mg, 71%, 96:4 e.r.) as a colourless oil. **^{1}H NMR** (400 MHz, $CDCl_3$) δ 7.84 – 7.77 (m, 2H), 7.36 (d, *J* = 8.1 Hz, 2H), 5.79 (dddd, *J* = 16.7, 14.8, 10.8, 5.8 Hz, 1H), 5.44 (ddt, *J* = 17.2, 2.7, 1.2 Hz, 1H), 5.36 (dt, *J* = 10.7, 1.1 Hz, 1H), 5.07 (dtdd, *J* = 48.3, 5.7, 3.1, 1.5 Hz, 1H), 4.23 – 4.02 (m, 2H), 2.45 (s, 3H). **^{13}C NMR** (101 MHz, $CDCl_3$) δ 145.3, 132.8, 130.9 (d, *J* = 19.3 Hz), 130.1, 128.1, 120.4 (d, *J* = 11.3 Hz), 90.0 (d, *J* = 176.0 Hz), 70.4 (d, *J* = 24.4 Hz), 21.8. **^{19}F NMR** (377 MHz, $CDCl_3$) δ -185.7 – -186.2 (m). **IR** (liquid film) ν = 2951, 1598, 1495, 1431, 1364, 1308, 1190, 1177, 1097, 1058, 988,

910, 884, 816, 789, 733, 669 cm^{-1}. **HRMS** (ESI$^+$) m/z calculated for $[M+H]^+$ 245.0642, found 245.0637. **$[\alpha]_D^{25}$ °C** = -24.4 (c = 0.7, $CHCl_3$). **HPLC separation:** DAICEL CHIRALPAK® ID-3, heptane:iPrOH = 97:3, 1 mL/min; t_1 = 15.2 min (minor), t_2 = 16.3 min (major).

Scale up: A 150 mL round-bottom flask was charged with **1l** (2.14 g, 7 mmol, 1 equiv.), (*S*)-**3d** (556 mg, 10 mol%), pre-ground KF (1.22 g, 3 equiv.) and $[Rh(cod)Cl]_2$ (86 mg, 2.5 mol%) in air. DCM (35 mL, 0.2 M) was added and the reaction was stirred at room temperature at 700 rpm for 48 h. The crude mixture was filtered and the solvent evaporated *in vacuo*. Purification by FCC (80:20 to 50:50 pentane:DCM) afforded the title compound (1.08 g, 63%, 97:3 e.r.) as a pale yellow oil. Further elution with DCM recovered (*S*)-**3d** (523 mg, 94% recovery).

(*R*)-2-fluorobut-3-en-1-yl 4-fluorobenzenesulfonate (2m)

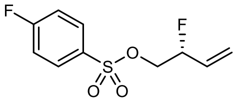

Prepared according to General Procedure I from 0.4 mmol of **1m** (124 mg). Purification by FCC (100:0 to 90:10 pentane:Et_2O) afforded the title compound (73.3 mg, 74%, 96:4 e.r.) as a yellow oil. **^{1}H NMR** (400 MHz, $CDCl_3$) δ 8.00 – 7.89 (m, 2H), 7.30 – 7.19 (m, 2H), 5.79 (dddd, *J* = 16.9, 14.7, 10.7, 5.7 Hz, 1H), 5.44 (ddt, *J* = 17.3, 2.7, 1.2 Hz, 1H), 5.37 (dt, *J* = 10.7, 1.2 Hz, 1H), 5.08 (dtdd, *J* = 48.4, 5.6, 3.0, 1.5 Hz, 1H), 4.27 – 4.07 (m, 2H). **^{13}C NMR** (101 MHz, $CDCl_3$) δ 166.0 (d, *J* = 256.9 Hz), 131.9 (d, *J* = 3.2 Hz), 131.0 (d, *J* = 9.6 Hz), 130.7 (d, *J* = 19.4 Hz), 120.6 (d, *J* = 11.1 Hz), 116.8 (d, *J* = 22.5 Hz), 89.9 (d, *J* = 176.4 Hz), 70.6 (d, *J* = 24.2 Hz). **^{19}F NMR** (377 MHz, $CDCl_3$) δ -102.6 (tt, *J* = 8.7, 4.3 Hz), -185.7 – -186.0 (m). **IR** (liquid film) ν = 2980, 1594, 1496, 1409, 1369, 1296, 1242, 1187, 1159, 1096, 1058, 987, 882, 840, 804, 672 cm^{-1}. **HRMS** (ESI$^+$) m/z calculated for $[M+H]^+$ 249.0392, found 249.0387. **$[\alpha]_D^{25}$ °C** = -25.6 (c = 0.8, $CHCl_3$). **HPLC separation:** DAICEL CHIRALPAK® ID-3, heptane:iPrOH = 97:3, 1 mL/min; t_1 = 9.2 min (minor), t_2 = 9.8 min (major).

(*R*)-2-fluorobut-3-en-1-yl diphenylphosphinate (2n)

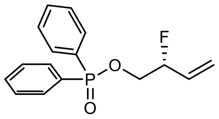

Prepared according to General Procedure I from 0.4 mmol of **1n** (140 mg) at 0 °C for 72 h. Purification by FCC (Et_2O) afforded the title compound (75.8 mg, 65%, 95:5 e.r.) as a yellow oil. **^{1}H NMR** (400 MHz, $CDCl_3$) δ 7.88 – 7.78 (m, 4H), 7.60 – 7.50 (m, 2H), 7.49 – 7.40 (m, 4H), 5.86 (dddd, *J* = 17.4, 14.9, 10.8, 5.7 Hz, 1H), 5.45 (ddt, *J* = 17.3, 2.8, 1.3 Hz, 1H), 5.34 (dt, *J* = 10.8, 1.3 Hz, 1H), 5.27 – 5.06 (m, 1H), 4.27 – 4.00 (m, 2H). **^{13}C NMR** (101 MHz, $CDCl_3$) δ 132.52 (d, *J* = 2.4 Hz), 132.49 (d, *J* = 2.6 Hz), 131.9 (d, *J* = 10.1 Hz), 131.8 (d, *J* = 18.0 Hz), 131.7 (d, *J* = 10.4 Hz), 131.2 (d, *J* = 138.3 Hz), 130.9 (d, *J* = 136.2 Hz), 128.8 (d, *J* = 13.2 Hz), 128.7 (d, *J* = 13.3 Hz), 119.8 (d, *J* = 11.4 Hz), 91.6 (dd, *J* = 174.6, 7.4 Hz), 65.7 (dd, *J* = 23.6, 5.8 Hz). **^{19}F NMR** (377 MHz, $CDCl_3$) δ -186.1 – -186.5 (m). **^{31}P NMR** (162 MHz, $CDCl_3$) δ 32.9. **IR** (liquid film) ν = 2980, 1592, 1439, 1388, 1229, 1131, 1114, 1025, 997, 909, 870, 832, 753, 731, 696 cm^{-1}. **HRMS** (ESI$^+$) m/z calculated for $[M+H]^+$ 291.0945, found 291.0939. **$[\alpha]_D^{25}$ °C** = -21.6 (c = 0.7, $CHCl_3$). **HPLC separation:** DAICEL CHIRALPAK® IB-3, heptane:iPrOH = 97:3, 1 mL/min; t_1 = 12.2 min (major), t_2 = 13.1 min (minor).

(*R*)-*tert*-butyl((2-fluorobut-3-en-1-yl)oxy)diphenylsilane (2o)

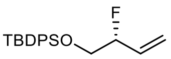

Prepared according to General Procedure I from 0.4 mmol of **1o** (156 mg) at 0 °C for 72 h. Purification by FCC (100:0 to 97:3 pentane:Et_2O) afforded the title compound (67.9 mg, 52%, 98:2 e.r.) as a colourless oil. **^{1}H NMR** (400 MHz, $CDCl_3$) δ 7.72 – 7.65 (m, 4H), 7.49 – 7.35 (m, 6H), 5.99 – 5.81 (m, 1H), 5.43 – 5.32 (m, 1H), 5.28 (dt, *J* = 10.7, 1.6 Hz, 1H), 5.08 – 4.86 (m, 1H), 3.86 – 3.70 (m, 2H), 1.07 (s, 9H). **^{13}C NMR** (101 MHz, $CDCl_3$) δ 135.78, 135.76, 133.5 (d, *J* = 19.4 Hz), 133.4, 133.3, 129.9, 127.9, 118.5 (d, *J* = 11.6 Hz), 93.6 (d, *J* = 172.3 Hz), 66.2 (d, *J* = 24.6 Hz), 26.9, 19.4. **^{19}F NMR** (377 MHz, $CDCl_3$) δ -185.5 – -186.1 (m). **IR** (liquid film) ν = 3072, 2959, 2931, 2859, 1589, 1473, 1428, 1390, 1362, 1113, 1081, 990, 937, 907, 866, 824, 801, 741, 702, 614 cm^{-1}. **HRMS** (ESI^+) m/z calculated for $[M+H]^+$ 329.1732, found 329.1729. **$[\alpha]_D^{25\,°C}$** = -6.7 (c = 0.6, $CHCl_3$).

The e.r. was determined as follows:

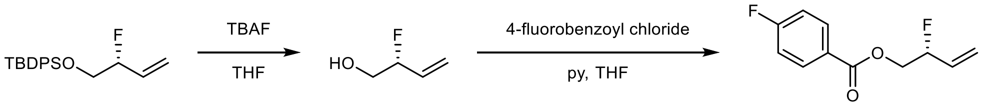

To a solution of **2o** (16.4 mg, 0.05 mmol, 1 equiv.) in THF (0.4 mL) was added TBAF (1 M in THF, 0.1 mL, 2 equiv.). The solution was stirred at room temperature for 3 h, filtered through silica, and concentrated *in vacuo*. The crude alcohol was then re-dissolved in THF (0.5 mL). Pyridine (0.02 mL, 5 equiv.) and 4-fluorobenzoyl chloride (0.01 mL, 2 equiv.) were added at 0 °C. The reaction mixture was warmed to room temperature and stirred overnight. The reaction was quenched with water (0.1 mL), dried with $MgSO_4$, filtered, and the solvent removed *in vacuo*. The ester product was isolated by preparative TLC, and its e.r. was determined using the same conditions as compound **2a**.

(*R*)-2-fluorobut-3-en-1-yl diphenylcarbamate (2p)

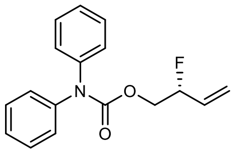

Prepared according to General Procedure I from 0.4 mmol of **1p** (138 mg). Purification by FCC (100:0 to 90:10 pentane:Et_2O) afforded the title compound (36.8 mg, 32%, 96:4 e.r.) as a yellow oil. **^{1}H NMR** (400 MHz, $CDCl_3$) δ 7.34 (dd, *J* = 8.5, 7.1 Hz, 4H), 7.29 – 7.19 (m, 6H), 5.80 (dddd, *J* = 16.8, 14.9, 10.7, 5.8 Hz, 1H), 5.41 (ddt, *J* = 17.2, 2.8, 1.3 Hz, 1H), 5.31 (dt, *J* = 10.8, 1.3 Hz, 1H), 5.19 – 4.98 (m, 1H), 4.41 – 4.19 (m, 2H). **^{13}C NMR** (101 MHz, $CDCl_3$) δ 154.4, 142.4, 132.1 (d, *J* = 19.2 Hz), 129.1, 127.0, 126.4, 119.4 (d, *J* = 11.5 Hz), 90.8 (d, *J* = 173.1 Hz), 67.0 (d, *J* = 23.7 Hz). **^{19}F NMR** (377 MHz, $CDCl_3$) δ -186.2 – -186.6 (m). **IR** (liquid film) ν = 2956, 2926, 2856, 1707, 1592, 1492, 1455, 1397, 1337, 1320, 1308, 1287, 1222, 1171, 1100, 1049, 1024, 983, 898, 847, 822, 761, 751, 736, 702, 689 cm^{-1}. **HRMS** (ESI^+) m/z calculated for $[M+H]^+$ 286.1238, found 286.1235. **$[\alpha]_D^{25\,°C}$** = -12.8 (c = 0.7, $CHCl_3$). **HPLC separation:** DAICEL CHIRALPAK® IF-3, heptane:iPrOH = 99.2:0.8, 1 mL/min; t_1 = 14.2 min (minor), t_2 = 16.0 min (major).

(*S*)-2-(3-fluoropent-4-en-1-yl)isoindoline-1,3-dione (2q)

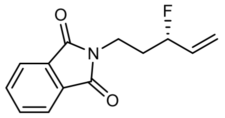

Prepared according to General Procedure I from 0.4 mmol of **1q** (114 mg). Purification by FCC (70:25:5 pentane:DCM:Et_2O) afforded the title compound (73.4 mg, 79%, 90:10 e.r.) as a yellow oil. **^{1}H NMR** (400 MHz, $CDCl_3$) δ 7.85 (dd, *J* = 5.4, 3.1 Hz, 2H), 7.72 (dd, *J* = 5.5, 3.0 Hz, 2H), 5.90 (dddd, *J* = 17.3, 14.4, 10.7, 5.8 Hz, 1H), 5.37 (ddt, *J* = 17.3, 3.4, 1.3 Hz, 1H), 5.25 (dt, *J* = 10.6, 1.2 Hz, 1H), 5.10 – 4.88 (m, 1H), 3.92 – 3.77 (m, 2H), 2.22 – 1.94 (m, 2H). **^{13}C NMR** (101 MHz, $CDCl_3$) δ 168.4, 135.6 (d, *J* = 19.5 Hz), 134.1, 132.2, 123.4, 117.7 (d, *J* = 11.8 Hz), 91.5 (d, *J* = 168.6 Hz), 34.3 (d, *J* = 4.9 Hz), 34.0 (d, *J* = 22.2 Hz). **^{19}F NMR** (377 MHz, $CDCl_3$) δ -179.6 – -179.9 (m). **IR** (liquid film) ν = 2953, 2926, 2856, 1772, 1712, 1468, 1444, 1398, 1377, 1063, 938, 891, 795, 720, 647 cm^{-1}. **HRMS** (ESI^+) m/z calculated for $[M+Na]^+$ 256.0744, found 256.0746. **$[\alpha]_D^{25\ °C}$** = -6.0 (c = 0.6, $CHCl_3$). **HPLC separation:** DAICEL CHIRALPAK® ID-3, heptane:iPrOH = 95:5, 1 mL/min; t_1 = 27.1 min (major), t_2 = 37.1 min (minor).

(*S*)-(3-fluoropent-4-en-1-yl)(naphthalen-2-yl)sulfane (2r)

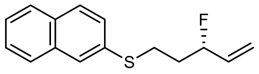

Prepared according to General Procedure I from 0.4 mmol of **1r** (123 mg). Purification by FCC (100:0 to 98:2 pentane:Et_2O) afforded an inseparable mixture of the title compound and the linear isomer (>20:1, 33.8 mg, 34%, 67.5:32.5 e.r.) as a yellow oil. The spectroscopic data refer to the title compound. **^{1}H NMR** (400 MHz, $CDCl_3$) δ 7.83 – 7.72 (m, 4H), 7.52 – 7.40 (m, 3H), 5.88 (dddd, *J* = 17.0, 14.6, 10.7, 5.8 Hz, 1H), 5.35 (ddt, *J* = 17.3, 3.2, 1.4 Hz, 1H), 5.25 (dt, *J* = 10.7, 1.3 Hz, 1H), 5.20 – 4.99 (m, 1H), 3.24 – 3.04 (m, 2H), 2.19 – 1.88 (m, 2H). **^{13}C NMR** (101 MHz, $CDCl_3$) δ 135.9 (d, *J* = 19.7 Hz), 133.9, 133.5, 132.0, 128.7, 127.9, 127.5, 127.3, 127.2, 126.7, 125.9, 117.6 (d, *J* = 11.8 Hz), 92.1 (d, *J* = 168.6 Hz), 35.0 (d, *J* = 22.6 Hz), 28.9 (d, *J* = 4.1 Hz). **^{19}F NMR** (377 MHz, $CDCl_3$) δ -180.1 (ddddd, *J* = 49.1, 28.6, 17.7, 14.6, 3.3 Hz). **IR** (liquid film) ν = 3054, 2925, 1625, 1590, 1501, 1429, 1268, 1133, 1071, 988, 942, 851, 812, 743 cm^{-1}. **HRMS** (GC EI) m/z calculated for $[M]^{+\cdot}$ 246.08730, found 246.08955. **$[\alpha]_D^{25\ °C}$** = -12.4 (c = 0.6, $CHCl_3$). **HPLC separation:** DAICEL CHIRALPAK® ID-3, heptane:iPrOH = 99.9:0.1, 1 mL/min; t_1 = 8.1 min (minor), t_2 = 10.0 min (major).

(*S*)-1-bromo-4-(3-fluoropent-4-en-1-yl)benzene (2s)

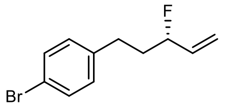

Prepared according to General Procedure I from 0.4 mmol of **1s** (122 mg). Purification by FCC (pentane) afforded the title compound (79.3 mg, 81%, 87:13 e.r.) as a colourless oil. **^{1}H NMR** (400 MHz, $CDCl_3$) δ 7.45 – 7.37 (m, 2H), 7.08 (d, *J* = 8.2 Hz, 2H), 5.89 (dddd, *J* = 16.9, 14.2, 10.7, 5.9 Hz, 1H), 5.33 (ddt, *J* = 17.3, 3.0, 1.4 Hz, 1H), 5.25 (dt, *J* = 10.6, 1.4 Hz, 1H), 4.87 (dddd, *J* = 48.6, 8.0, 6.0, 4.5 Hz, 1H), 2.81 – 2.61 (m, 2H), 2.11 – 1.77 (m, 2H). **^{13}C NMR** (101 MHz, $CDCl_3$) δ 140.3, 136.3 (d, *J* = 19.5 Hz), 131.7, 130.4, 119.9, 117.4 (d, *J* = 11.8 Hz), 92.6 (d, *J* = 168.0 Hz), 36.8 (d, *J* = 22.4 Hz), 30.5 (d, *J* = 4.5 Hz). **^{19}F NMR** (377 MHz, $CDCl_3$) δ -179.1 (ddddd, *J* = 48.6, 28.2, 17.3, 14.1, 3.5 Hz). **IR** (liquid film) ν = 3084, 3022, 2981, 2952, 2866, 1897, 1649, 1592, 1489, 1453, 1428, 1405, 1179, 1103, 1073, 1030, 1012, 989, 970, 933, 881, 832, 801, 763, 713, 630 cm^{-1}. **HRMS** (GC EI) m/z

calculated for $[M]^{+\cdot}$ 242.01009, found 242.01064. **$[\alpha]_D^{25\ °C}$** = -9.9 (c = 0.9, $CHCl_3$). **HPLC separation:** DAICEL CHIRALPAK® IF-3, 100% heptane, 0.5 mL/min; t_1 = 11.5 min (minor), t_2 = 12.3 min (major).

(*S*)-4-(3-fluoropent-4-en-1-yl)-1,2-dimethoxybenzene (2t)

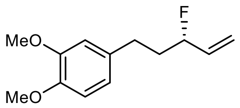

Prepared according to General Procedure I from 0.4 mmol of **1t** (114 mg). Purification by FCC (60:40 pentane:DCM) afforded the title compound (74.8 mg, 83%, 84:16 e.r.) as a yellow oil. **^{1}H NMR** (400 MHz, $CDCl_3$) δ 6.80 (d, *J* = 7.9 Hz, 1H), 6.78 – 6.68 (m, 2H), 5.91 (dddd, *J* = 17.0, 14.2, 10.7, 6.0 Hz, 1H), 5.33 (ddt, *J* = 17.3, 3.3, 1.4 Hz, 1H), 5.24 (dt, *J* = 10.7, 1.3 Hz, 1H), 4.99 – 4.77 (m, 1H), 3.87 (s, 3H), 3.86 (s, 3H), 2.80 – 2.60 (m, 2H), 2.12 – 1.80 (m, 2H). **^{13}C NMR** (101 MHz, $CDCl_3$) δ 149.0, 147.5, 136.6 (d, *J* = 19.5 Hz), 133.9, 120.4, 117.2 (d, *J* = 11.9 Hz), 111.9, 111.4, 92.8 (d, *J* = 167.4 Hz), 56.1, 56.0, 37.3 (d, *J* = 22.3 Hz), 30.7 (d, *J* = 4.5 Hz). **^{19}F NMR** (377 MHz, $CDCl_3$) δ -178.9 (ddddd, *J* = 48.7, 28.4, 17.2, 14.1, 3.5 Hz). **IR** (liquid film) ν = 2998, 2951, 2936, 2836, 1608, 1591, 1517, 1465, 1419, 1329, 1262, 1237, 1157, 1142, 1030, 991, 934, 877, 855, 809, 764, 638 cm^{-1}. **HRMS** (ESI^+) m/z calculated for $[M+H]^+$ 225.1285, found 225.1287. **$[\alpha]_D^{25\ °C}$** = -12.2 (c = 0.8, $CHCl_3$). **HPLC separation:** DAICEL CHIRALPAK® IB-3, heptane:iPrOH = 99.5:0.5, 1 mL/min; t_1 = 10.8 min (major), t_2 = 11.8 min (minor).

(*S*)-(3-fluoropent-4-ene-1,1-diyl)dibenzene (2u)

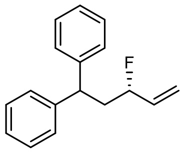

Prepared according to General Procedure I from 0.4 mmol of **1u** (120 mg). Purification by FCC (100:0 to 98:2 pentane:Et_2O) afforded the title compound (55.4 mg, 58%, 83:17 e.r.) as a colourless oil. **^{1}H NMR** (400 MHz, CD_2Cl_2) δ 7.37 – 7.25 (m, 8H), 7.25 – 7.15 (m, 2H), 5.92 (dddd, *J* = 16.9, 13.3, 10.6, 6.1 Hz, 1H), 5.35 – 5.20 (m, 2H), 4.69 (ddt, *J* = 48.7, 10.1, 5.5 Hz, 1H), 4.18 (dd, *J* = 9.7, 6.1 Hz, 1H), 2.55 – 2.25 (m, 2H). **^{13}C NMR** (101 MHz, CD_2Cl_2) δ 144.9, 144.2, 136.9 (d, *J* = 19.4 Hz), 129.1, 129.0, 128.4, 128.0, 126.9, 126.8, 117.6 (d, *J* = 11.7 Hz), 92.1 (d, *J* = 166.6 Hz), 47.1 (d, *J* = 4.2 Hz), 41.3 (d, *J* = 22.6 Hz). **^{19}F NMR** (377 MHz, CD_2Cl_2) δ -178.8 (ddtd, *J* = 47.6, 30.2, 13.4, 4.0 Hz). **IR** (liquid film) ν = 3086, 3063, 3028, 2946, 1600, 1495, 1452, 1429, 1093, 1030, 986, 938, 860, 788, 752, 739, 701, 636 cm^{-1}. **$[\alpha]_D^{25\ °C}$** = -50.4 (c = 0.5, $CHCl_3$). **HPLC separation:** DAICEL CHIRALPAK® OJ-3, heptane:iPrOH = 99:1, 1 mL/min; t_1 = 15.5 min (major), t_2 = 25.3 min (minor). No HRMS obtained.

(*S*)-((4-(3-fluoropent-4-en-1-yl)phenyl)ethynyl)trimethylsilane (2v)

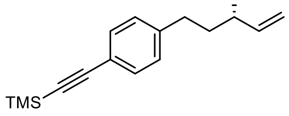

Prepared according to General Procedure I from 0.4 mmol of **1v** (129 mg). Purification by FCC (100:0 to 99:1 pentane:Et_2O) afforded the title compound (51.6 mg, 50%, 87:13 e.r.) as a yellow oil. **^{1}H NMR** (400 MHz, $CDCl_3$) δ 7.43 – 7.36 (m, 2H), 7.19 – 7.09 (m, 2H), 5.89 (dddd, *J* = 17.3, 14.2, 10.6, 6.0 Hz, 1H), 5.32 (ddt, *J* = 17.3, 3.5, 1.4 Hz, 1H), 5.24 (dt, *J* = 10.6, 1.3 Hz, 1H), 4.85 (dddt, *J* = 48.5, 11.8, 5.8, 1.3 Hz, 1H), 2.83 – 2.64 (m, 2H), 2.11 – 1.79 (m, 2H), 0.24 (s, 9H). **^{13}C NMR** (101 MHz, $CDCl_3$) δ 142.0, 136.4 (d, *J* = 19.4 Hz), 132.2, 128.5, 120.9, 117.4 (d, *J* = 11.8 Hz), 105.2, 93.8, 92.7 (d, *J* = 167.8 Hz), 36.8 (d, *J* = 22.4 Hz), 31.0 (d, *J* = 4.6 Hz), 0.2. **^{19}F NMR** (377 MHz, $CDCl_3$) δ -179.0 (ddddd, *J* = 48.7, 28.2, 17.2, 14.1, 3.5 Hz). **IR** (liquid film) ν = 2960, 2158, 1507,

1410, 1250, 1223, 1032, 988, 933, 865, 842, 760, 700, 637 cm^{-1}. **HRMS** (ESI^+) m/z calculated for $[M+H]^+$ 261.1469, found 261.1481. **$[\alpha]_D^{25\ °C}$** = -11.4 (c = 0.6, $CHCl_3$). **HPLC separation:** DAICEL CHIRALPAK® IB-3, heptane:iPrOH = 99.9:0.1, 0.5 mL/min; t_1 = 5.8 min (major), t_2 = 6.1 min (minor).

***tert*-butyl (*S*)-4-(2-fluorobut-3-en-1-yl)piperidine-1-carboxylate (2w)**

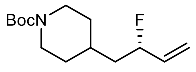

Prepared according to General Procedure I from 0.4 mmol of **1w** (127 mg). Purification by FCC (90:10 pentane:Et_2O) afforded the title compound (46.6 mg, 45%, 86:14 e.r.) as a yellow oil. **^{1}H NMR** (400 MHz, $CDCl_3$) δ 5.96 – 5.79 (m, 1H), 5.37 – 5.27 (m, 1H), 5.22 (dt, *J* = 10.6, 1.2 Hz, 1H), 5.09 – 4.87 (m, 1H), 4.14 – 4.02 (m, 2H), 2.69 (td, *J* = 12.9, 2.7 Hz, 2H), 1.81 – 1.59 (m, 4H), 1.55 – 1.36 (m, 1H), 1.45 (s, 9H), 1.23 – 1.04 (m, 2H). **^{13}C NMR** (101 MHz, $CDCl_3$) δ 155.0, 136.8 (d, *J* = 19.5 Hz), 117.1 (d, *J* = 11.7 Hz), 91.6 (d, *J* = 166.9 Hz), 79.4, 44.0, 43.9, 42.1 (d, *J* = 21.8 Hz), 32.7, 32.4 (d, *J* = 3.1 Hz), 31.8, 28.6. **^{19}F NMR** (377 MHz, $CDCl_3$) δ -177.1 – -177.6 (m). **IR** (liquid film) ν = 2978, 2930, 1694, 1424, 1366, 1279, 1247, 1173, 968, 934, 869, 769 cm^{-1}. **HRMS** (ESI^+) m/z calculated for $[M+H]^+$ 258.1864, found 258.1860. **$[\alpha]_D^{25\ °C}$** = -13.9 (c = 0.5, $CHCl_3$). **HPLC separation:** DAICEL CHIRALPAK® OJ-3, heptane:iPrOH = 99:1, 1 mL/min; t_1 = 4.7 min (minor), t_2 = 5.0 min (major).

***tert*-butyl (*R*)-3-(1-fluoroallyl)azetidine-1-carboxylate (2x)**

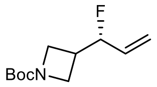

Prepared according to General Procedure I from 0.4 mmol of **1x** (110 mg) at room temperature for 24 h. Purification by FCC (70:25:5 pentane:DCM:Et_2O) afforded the title compound (32.6 mg, 38%, 71.5:28.5 e.r.) as a yellow oil. **^{1}H NMR** (400 MHz, $CDCl_3$) δ 5.80 (dddd, *J* = 17.0, 13.8, 10.7, 6.0 Hz, 1H), 5.39 (ddt, *J* = 17.3, 3.5, 1.3 Hz, 1H), 5.31 (dt, *J* = 10.4, 1.1 Hz, 1H), 5.07 – 4.88 (m, 1H), 4.01 – 3.91 (m, 2H), 3.86 (dd, *J* = 8.8, 5.5 Hz, 1H), 3.73 (dd, *J* = 8.8, 5.6 Hz, 1H), 2.83 – 2.67 (m, 1H), 1.43 (s, 9H). **^{13}C NMR** (101 MHz, $CDCl_3$) δ 156.4, 133.4 (d, *J* = 19.8 Hz), 119.1 (d, *J* = 11.4 Hz), 93.6 (d, *J* = 170.9 Hz), 79.7, 50.5 (d, *J* = 5.1 Hz), 50.0 (d, *J* = 7.9 Hz), 32.4 (d, *J* = 23.7 Hz), 28.5. **^{19}F NMR** (377 MHz, $CDCl_3$) δ -186.4 (dt, *J* = 48.3, 16.1 Hz). **IR** (liquid film) ν = 2976, 2890, 1707, 1479, 1407, 1367, 1255, 1144, 973, 945, 773, 644 cm^{-1}. **HRMS** (ESI^+) m/z calculated for $[M+H]^+$ 216.1394, found 216.1392. **$[\alpha]_D^{25\ °C}$** = -6.4 (c = 0.6, $CHCl_3$). **HPLC separation:** DAICEL CHIRALPAK® OJ-3, heptane:iPrOH = 98:2, 1 mL/min; t_1 = 4.4 min (minor), t_2 = 5.2 min (major).

(*S*)-2-(3-fluoropent-4-en-1-yl)-5-methylfuran (2y)

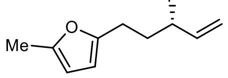

Prepared according to General Procedure I from 0.4 mmol of **1y** (91.6 mg). Purification by FCC (pentane) afforded the title compound (34.8 mg, 52%, 73:27 e.r.) as a colourless oil. **^{1}H NMR** (400 MHz, $CDCl_3$) δ 5.98 – 5.81 (m, 3H), 5.34 (ddt, *J* = 17.3, 3.5, 1.4 Hz, 1H), 5.24 (dt, *J* = 10.6, 1.3 Hz, 1H), 5.02 – 4.82 (m, 1H), 2.80 – 2.62 (m, 2H), 2.25 (d, *J* = 1.0 Hz, 3H), 2.12 – 1.86 (m, 2H). **^{13}C NMR** (101 MHz, $CDCl_3$) δ 153.1, 150.7, 136.4 (d, *J* = 19.5 Hz), 117.3 (d, *J* = 11.9 Hz), 106.0 (2C), 92.8 (d, *J* = 167.6 Hz), 33.9 (d, *J* = 22.4 Hz), 23.5 (d, *J* = 5.2 Hz), 13.6. **^{19}F NMR** (377 MHz, $CDCl_3$) δ -179.1 – -179.4 (m). **IR** (liquid film) ν = 3098, 2950, 2924, 1651, 1618, 1571, 1431, 1385, 1357, 1220, 1171, 1105, 1020, 990, 970, 938, 876, 784, 745, 665, 651, 642 cm^{-1}. **HRMS** (APCI) m/z calculated for $[M+H]^+$ 169.1023, found

169.1025. **$[\alpha]_D^{25\ °C}$** = -11.3 (c = 0.5, $CHCl_3$). **HPLC separation:** DAICEL CHIRALPAK® ID-3, 100% heptane, 0.8 mL/min; t_1 = 5.7 min (minor), t_2 = 6.6 min (major).

***tert*-butyl (*S*)-3-(3-fluoropent-4-en-1-yl)-1*H*-indole-1-carboxylate (2z)**

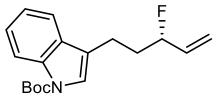

Prepared according to General Procedure I from 0.4 mmol of **1z** (146 mg). Purification by FCC (100:0 to 98:2 pentane:Et_2O) afforded the title compound (56.9 mg, 47%, 87:13 e.r.) as a colourless oil. **^{1}H NMR** (400 MHz, $CDCl_3$) δ 8.14 (d, *J* = 8.1 Hz, 1H), 7.54 (d, *J* = 7.7 Hz, 1H), 7.40 (s, 1H), 7.36 – 7.29 (m, 1H), 7.25 (t, *J* = 7.5 Hz, 1H), 5.94 (dddd, *J* = 16.9, 14.3, 10.7, 5.9 Hz, 1H), 5.36 (ddt, *J* = 17.3, 3.1, 1.4 Hz, 1H), 5.26 (d, *J* = 10.7 Hz, 1H), 5.09 – 4.87 (m, 1H), 2.96 – 2.74 (m, 2H), 2.22 – 1.92 (m, 2H), 1.68 (s, 9H). **^{13}C NMR** (101 MHz, $CDCl_3$) δ 149.9, 136.5 (d, *J* = 19.7 Hz), 135.7, 130.7, 124.5, 122.7, 122.5, 120.1, 119.0, 117.3 (d, *J* = 11.6 Hz), 115.4, 92.9 (d, *J* = 167.8 Hz), 83.6, 35.0 (d, *J* = 22.3 Hz), 28.4, 20.2 (d, *J* = 4.8 Hz). **^{19}F NMR** (377 MHz, $CDCl_3$) δ -179.0 – -179.5 (m). **IR** (liquid film) ν = 2934, 1733, 1609, 1455, 1379, 1309, 1256, 1226, 1161, 1090, 1019, 931, 858, 767, 747, 642 cm^{-1}. **HRMS** (ESI^+) m/z calculated for $[M+H]^+$ 304.1707, found 304.1702. **$[\alpha]_D^{25\ °C}$** = -13.6 (c = 0.3, $CHCl_3$). **HPLC separation:** DAICEL CHIRALPAK® IB-3, heptane:iPrOH = 99.5:0.5, 1 mL/min; t_1 = 3.8 min (minor), t_2 = 4.4 min (major).

(*S*)-2-(3-fluoropent-4-en-1-yl)benzo[*b*]thiophene (2aa)

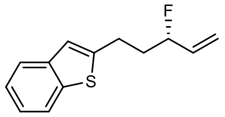

Prepared according to General Procedure I from 0.4 mmol of **1aa** (112 mg). Purification by FCC (pentane) afforded the title compound (51.7 mg, 59%, 80:20 e.r.) as a colourless oil. **^{1}H NMR** (400 MHz, $CDCl_3$) δ 7.78 (dd, *J* = 8.0, 1.2 Hz, 1H), 7.72 – 7.65 (m, 1H), 7.36 – 7.30 (m, 1H), 7.32 – 7.23 (m, 1H), 7.05 (q, *J* = 1.0 Hz, 1H), 6.01 – 5.85 (m, 1H), 5.37 (ddt, *J* = 17.3, 3.5, 1.4 Hz, 1H), 5.28 (dt, *J* = 10.6, 1.3 Hz, 1H), 5.09 – 4.87 (m, 1H), 3.15 – 2.98 (m, 2H), 2.27 – 1.97 (m, 2H). **^{13}C NMR** (101 MHz, $CDCl_3$) δ 145.0, 140.2, 139.5, 136.2 (d, *J* = 19.5 Hz), 124.3, 123.8, 123.0, 122.3, 121.3, 117.6 (d, *J* = 11.9 Hz), 92.4 (d, *J* = 168.1 Hz), 36.6 (d, *J* = 22.6 Hz), 26.1 (d, *J* = 4.8 Hz). **^{19}F NMR** (377 MHz, $CDCl_3$) δ -179.6 (ddddd, *J* = 48.4, 28.1, 17.2, 14.2, 3.4 Hz). **IR** (liquid film) ν = 3060, 2980, 1458, 1436, 1380, 1252, 1215, 1155, 1066, 1031, 1015, 988, 967, 935, 880, 857, 825, 746, 726, 707 cm^{-1}. **HRMS** (ESI^+) m/z calculated for $[M+H]^+$ 221.0795, found 221.0794. **$[\alpha]_D^{25\ °C}$** = -9.5 (c = 0.6, $CHCl_3$). **HPLC separation:** DAICEL CHIRALPAK® IB-3, heptane:iPrOH = 99.9:0.1, 1 mL/min; t_1 = 5.5 min (major), t_2 = 6.1 min (minor).

(8*R*,9*S*,13*S*,14*S*)-3-(((*R*)-2-fluorobut-3-en-1-yl)oxy)-13-methyl-6,7,8,9,11,12,13,14,15,16-decahydro-17*H*-cyclopenta[*a*]phenanthren-17-one (2ab)

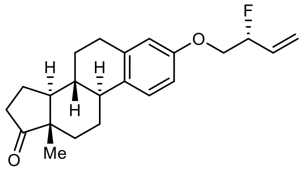

Prepared according to General Procedure I from 0.4 mmol of **1ab** (161 mg). Purification by FCC (80:10:10 pentane:Et_2O:DCM) afforded the title compound (110 mg, 80%, 96:4 d.r.) as a white solid. **^{1}H NMR** (400 MHz, $CDCl_3$) δ 7.21 (d, *J* = 8.5 Hz, 1H), 6.74 (dd, *J* = 8.6, 2.8 Hz, 1H), 6.68 (d, *J* = 2.7 Hz, 1H), 5.99 (dddd, *J* = 16.9, 14.9, 10.8, 5.8 Hz, 1H), 5.51 (ddt, *J* = 17.4, 2.9, 1.3 Hz, 1H), 5.38 (dt, *J* = 10.8, 1.2 Hz, 1H),

5.34 – 5.14 (m, 1H), 4.17 – 3.99 (m, 2H), 2.95 – 2.84 (m, 2H), 2.51 (dd, *J* = 18.8, 8.6 Hz, 1H), 2.43 – 2.34 (m, 1H), 2.31 – 2.21 (m, 1H), 2.21 – 2.11 (m, 1H), 2.11 – 1.89 (m, 3H), 1.70 – 1.36 (m, 6H), 0.91 (s, 3H). **^{13}C NMR** (101 MHz, $CDCl_3$) δ 221.1, 156.6, 138.0, 132.9, 132.7 (d, *J* = 19.2 Hz), 126.6, 119.2 (d, *J* = 11.3 Hz), 115.0, 112.4, 91.4 (d, *J* = 172.6 Hz), 70.0 (d, *J* = 24.1 Hz), 50.5, 48.1, 44.1, 38.5, 36.0, 31.7, 29.8, 26.6, 26.0, 21.7, 14.0. **^{19}F NMR** (377 MHz, $CDCl_3$) δ -185.2 – -185.6 (m). **IR** (liquid film) ν = 2980, 2922, 2888, 1729, 1653, 1611, 1574, 1495, 1473, 1393, 1342, 1280, 1255, 1186, 1157, 1059, 1007, 993, 941, 872, 813, 782, 668, 657 cm^{-1}. **HRMS** (ESI^+) m/z calculated for $[M+H]^+$ 343.2068, found 343.2062. **m.p.** 71 – 74 °C. **$[\alpha]_D^{25\ °C}$** = +127.9 (c = 0.7, $CHCl_3$).

The d.r. was determined by quantitative $^{19}F\{^1H\}$ NMR:

Reference prepared through deoxyfluorination of **6ab** with DAST (~1:1 d.r.):	Diastereoselective reaction (96:4 d.r.):
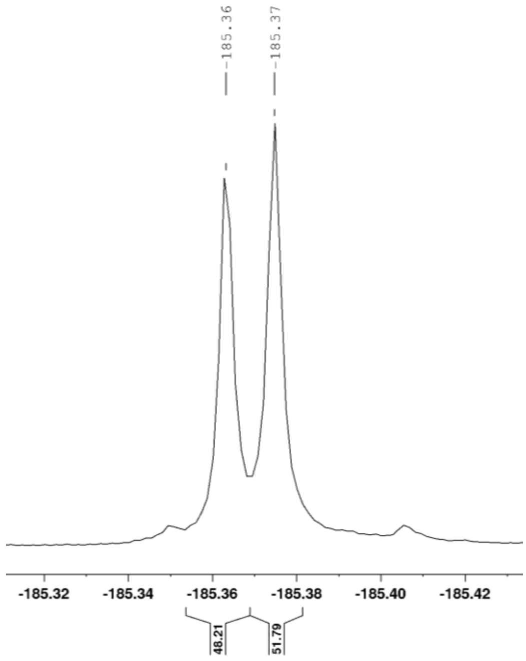	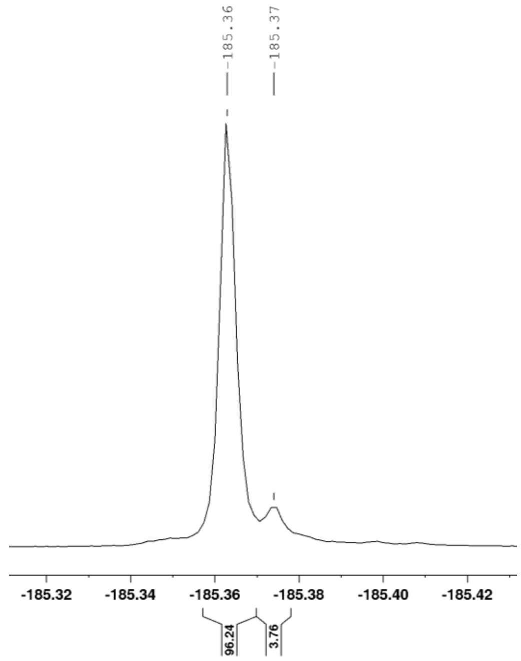

Procedures for Product Derivatisation

Racemic standards for compounds **7–9** were prepared by the same procedures from *rac*-**2l**, which was independently synthesised following a literature procedure[15]. The products were purified by preparative TLC for HPLC.

(*R*)-2-(2-fluorobut-3-en-1-yl)-1,2,3,4-tetrahydroisoquinoline (7)

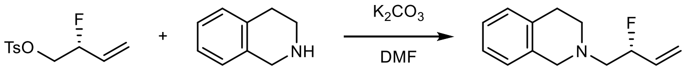

Using a modified literature procedure[16]: To a solution of **2l** (98 mg, 0.4 mmol, 1 equiv., 97:3 e.r.) in anhydrous DMF (1.6 mL) under an inert atmosphere were added K_2CO_3 (61 mg, 1.1 equiv.) and 1,2,3,4-tetrahydroisoquinoline (59 mg, 1.1 equiv.). The reaction mixture was heated to 100 °C for 16 h. The reaction was cooled to room temperature and poured into a mixture of water and Et_2O. The organic layer was separated and the aqueous layer extracted twice with Et_2O. The combined organic fractions were washed with brine, dried with $MgSO_4$, filtered, and evaporated *in vacuo*. Purification by FCC (90:10 pentane:Et_2O) afforded the title compound (44.4 mg, 54%, 97:3 e.r.) as a pale yellow oil. **^{1}H NMR** (400 MHz, $CDCl_3$) δ 7.18 – 7.07 (m, 3H), 7.05 – 6.98 (m, 1H), 5.96 (dddd, *J* = 17.0, 15.3, 10.7, 5.7 Hz, 1H), 5.42 (ddt, *J* = 17.4, 3.0, 1.4 Hz, 1H), 5.33 – 5.10 (m, 2H), 3.79 (s, 2H), 2.97 – 2.82 (m, 5H), 2.76 (ddd, *J* = 30.1, 14.0, 3.2 Hz, 1H). **^{13}C NMR** (101 MHz, $CDCl_3$) δ 135.0 (d, *J* = 19.5 Hz), 134.5, 134.2, 128.8, 126.7, 126.4, 125.8, 117.6 (d, *J* = 11.9 Hz), 92.2 (d, *J* = 170.8 Hz), 62.1 (d, *J* = 22.3 Hz), 56.5, 51.5, 29.0. **^{19}F NMR** (377 MHz, $CDCl_3$) δ -178.9 (ddddd, *J* = 48.9, 29.9, 18.8, 15.0, 3.2 Hz). **IR** (liquid film) ν = 3023, 2925, 2811, 1498, 1466, 1455, 1428, 1141, 1101, 1031, 990, 937, 748, 643 cm^{-1}. **HRMS** (ESI^+) m/z calculated for $[M+H]^+$ 206.1340, found 206.1334. **$[\alpha]_D^{25\,°C}$** = -2.2 (c = 0.5, $CHCl_3$). **HPLC separation:** DAICEL CHIRALPAK® OJ-3, heptane:iPrOH = 99.5:0.5, 0.5 mL/min; t_1 = 18.5 min (minor), t_2 = 19.8 min (major).

(*S*)-(3-fluoropent-4-ene-1,1-diyl)dibenzene (2u')

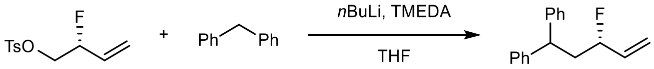

Using a modified literature procedure[17]: To a solution of diphenylmethane (42 mg, 0.25 mmol, 1 equiv.) in anhydrous THF (0.9 mL) at -78 °C was added *n*-butyllithium (2.5 M in hexane, 0.1 mL, 1 equiv.). The solution was warmed to 15 °C and stirred for 0.5 h. At the same temperature, TMEDA (37.5 µL, 1 equiv.) and **2l** (61 mg in 0.5 mL THF, 0.25 mmol, 1 equiv., 97:3 e.r.) were sequentially added. The reaction mixture was stirred at room temperature for 4 h and then at 40 °C for 16 h. The reaction was quenched with sat. NH_4Cl and extracted three times with EtOAc. The combined organic fractions were dried with $MgSO_4$, filtered, and evaporated *in vacuo*. Purification by FCC (pentane) afforded the title compound (37.3 mg, 62%, 97:3 e.r.) as a colourless oil. All spectroscopic data were identical with compound **2u**.

(*R*)-2-fluoro-4-hydroxybutyl 4-methylbenzenesulfonate (8)

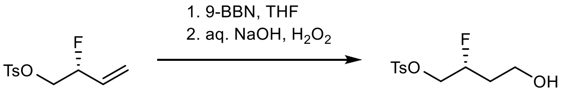

Using a modified literature procedure[18]: **2l** (49 mg, 0.2 mmol, 1 equiv., 97:3 e.r.) was dissolved in anhydrous THF (0.4 mL) under an inert atmosphere at 0 °C. 9-BBN (0.5 M in THF, 2 mL, 5 equiv.) was added dropwise. The reaction mixture was stirred at room temperature for 30 min and then at 40 °C for 16 h. The reaction was cooled to 0 °C and charged with 3 M NaOH (0.25 mL) and 30% w/w aq. H_2O_2. After stirring at room temperature for 1 h, the reaction mixture was poured into brine and extracted three times with Et_2O. The combined organic fractions were dried with $MgSO_4$, filtered, and evaporated *in vacuo*. Purification by FCC (50:50 pentane:EtOAc) afforded the title compound (47.1 mg, 90%, 97:3 e.r.) as a colourless oil. **^{1}H NMR** (400 MHz, $CDCl_3$) δ 7.80 (d, *J* = 8.4 Hz, 2H), 7.35 (d, *J* = 8.1 Hz, 2H), 5.00 – 4.78 (m, 1H), 4.28 – 4.07 (m, 2H), 3.83 – 3.72 (m, 2H), 2.45 (s, 3H), 2.00 – 1.78 (m, 2H), 1.75 (s, 1H). **^{13}C NMR** (101 MHz, $CDCl_3$) δ 145.3, 132.8, 130.1, 128.1, 88.4 (d, *J* = 174.7 Hz), 70.9 (d, *J* = 23.0 Hz), 58.2 (d, *J* = 5.1 Hz), 33.7 (d, *J* = 20.6 Hz), 21.8. **^{19}F NMR** (377 MHz, $CDCl_3$) δ -189.4 – -189.8 (m). **IR** (liquid film) ν = 3412, 2931, 1598, 1450, 1363, 1191, 1178, 1097, 1056, 996, 974, 952, 881, 816, 669 cm^{-1}. **HRMS** (ESI^+) m/z calculated for $[M+NH_4]^+$ 263.0748, found 263.0745. **$[\alpha]_D^{25\ °C}$** = +3.9 (c = 0.7, $CHCl_3$). **HPLC separation:** DAICEL CHIRALPAK® ID-3, heptane:EtOH = 90:10, 1 mL/min; t_1 = 21.8 min (minor), t_2 = 27.3 min (major).

(*R*,*E*)-4-(4-bromophenyl)-2-fluorobut-3-en-1-yl 4-methylbenzenesulfonate (9)

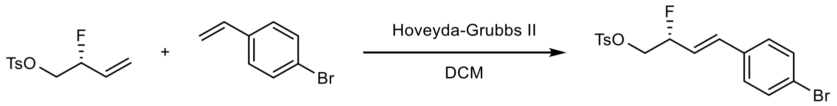

Using a modified literature procedure[19]: In a sealed tube under nitrogen atmosphere, **2l** (49 mg, 0.2 mmol, 1 equiv., 97:3 e.r.), 4-bromostyrene (183 mg, 5 equiv.) and Hoveyda-Grubbs II catalyst (12.5 mg, 10 mol%) were dissolved in degassed DCM (4 mL). The solution was heated at 60 °C for 16 h. After cooling to room temperature, the reaction mixture was passed through a silica plug, eluting with ether. Purification by FCC (80:20 pentane:Et_2O) afforded the title compound (55.5 mg, 70%, 97:3 e.r.) as a colourless oil. **^{1}H NMR** (400 MHz, $CDCl_3$) δ 7.80 (d, *J* = 8.3 Hz, 2H), 7.46 (d, *J* = 8.5 Hz, 2H), 7.34 (d, *J* = 8.1 Hz, 2H), 7.21 (d, *J* = 8.5 Hz, 2H), 6.65 (dd, *J* = 16.1, 3.4 Hz, 1H), 6.08 (ddd, *J* = 16.0, 13.3, 6.5 Hz, 1H), 5.32 – 5.13 (m, 1H), 4.31 – 4.11 (m, 2H), 2.44 (s, 3H). **^{13}C NMR** (101 MHz, $CDCl_3$) δ 145.3, 134.4 (d, *J* = 11.4 Hz), 134.3, 132.8, 132.0, 130.1, 128.5, 128.1, 122.9, 122.2 (d, *J* = 18.5 Hz), 89.9 (d, *J* = 175.6 Hz), 70.4 (d, *J* = 25.5 Hz), 21.8. **^{19}F NMR** (377 MHz, $CDCl_3$) δ -181.7 – -182.1 (m). **IR** (liquid film) ν = 2950, 2925, 1598, 1489, 1365, 1190, 1177, 1097, 1073, 1009, 985, 830, 813, 763, 667 cm^{-1}. **HRMS** (ESI^+) m/z calculated for $[M+Na]^+$ 420.9880, found 420.9872. **$[\alpha]_D^{25\ °C}$** = -26.2 (c = 0.8, $CHCl_3$). **HPLC separation:** DAICEL CHIRALPAK® IB-3, heptane:iPrOH = 95:5, 1 mL/min; t_1 = 14.7 min (minor), t_2 = 17.3 min (major).

Non-Linear Effect Study

The non-linear effect study was performed on model substrate (*rac*-**1a**) with scalemic mixtures of catalyst **3d** (10 mol%), $[Rh(cod)Cl]_2$ (2.5 mol%), KF (3 equiv.) in DCM or EtOAc at -30 °C. No background reaction was observed under these conditions and the results shown are mean values of two sets of experiments.

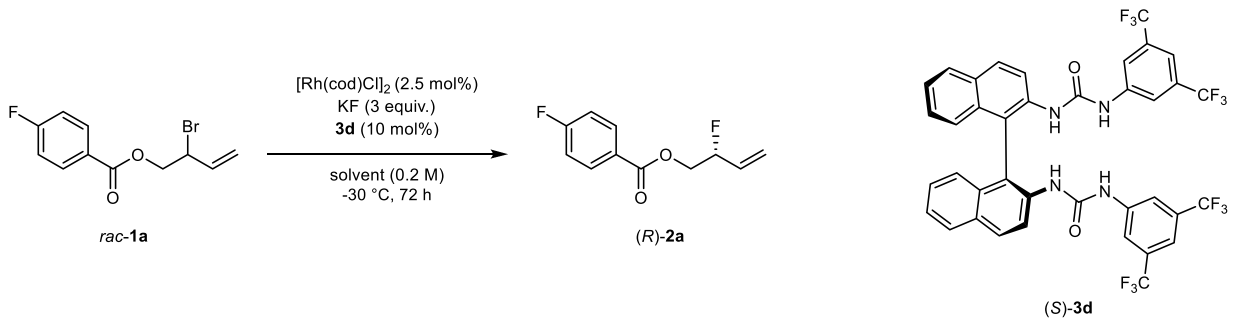

Table S7: Non-linear effect study in DCM solvent

Entry	%e.e. of catalyst **3d**[a]	%e.e. of product **2a**	yield of **2a** (%)[b]
1	0.1	7.7	4
2	17.8	87.2	29
3	37.7	86.0	47
4	57.6	87.9	71
5	77.4	89.0	80
6	100	91.6	77

[a]Scalemic mixtures of catalyst **3d** were prepared by mixing (*S*)-**3d** with *rac*-**3d**, dissolving in acetone, and evaporating to dryness. The enantiomeric excess (%e.e.) of each catalyst batch was determined by chiral HPLC. **HPLC separation:** DAICEL CHIRALPAK® OJ-3, heptane: iPrOH = 97.5:2.5, 0.5 mL/min; t_1 = 4.6, t_2 = 5.3 min. [b]Determined by ^{19}F NMR using 4-fluoroanisole as internal standard.

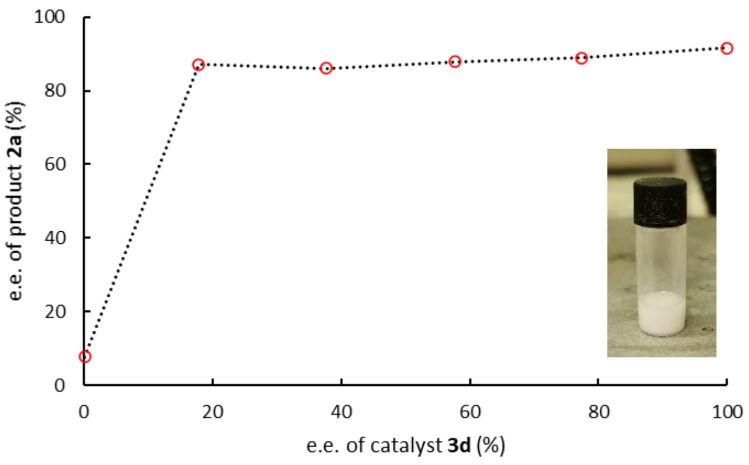

Figure S1: Non-linear effect study in DCM solvent. Inset: *rac*-**3d** is poorly soluble in DCM.

Table S8: Non-linear effect study in EtOAc solvent

Entry	%e.e. of catalyst **3d**[a]	%e.e. of product **2a**
1	0.1	0.2
2	17.8	19.4
3	37.7	36.2
4	57.6	51.4
5	77.4	67.4
6	100	90.3

[a]Scalemic mixtures of catalyst **3d** were prepared by mixing (*S*)-**3d** with *rac*-**3d**, dissolving in acetone, and evaporating to dryness. The enantiomeric excess (%e.e.) of each catalyst batch was determined by chiral HPLC. **HPLC separation:** DAICEL CHIRALPAK® OJ-3, heptane: iPrOH = 97.5:2.5, 0.5 mL/min; t_1 = 4.6, t_2 = 5.3 min.

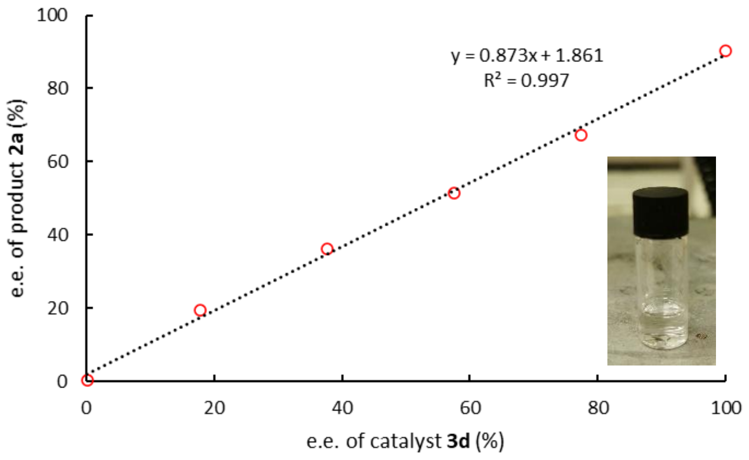

Figure S2: Non-linear effect study in EtOAc solvent. Inset: *rac*-**3d** is fully soluble in EtOAc (20 mM).

To investigate the behavior of **3d** in DCM, partially enantioenriched (*S*)-**3d** (4 mg, 38% e.e.) was added to DCM (0.25 mL). The resulting mixture was sonicated for 5 min and filtered. The e.e. of the resulting filtrate and the precipitate were separately measured. It was found that (*S*)-**3d** in solution is enantiopure (opposite enantiomer not observable) and the precipitated **3d** is essentially racemic.

(a)

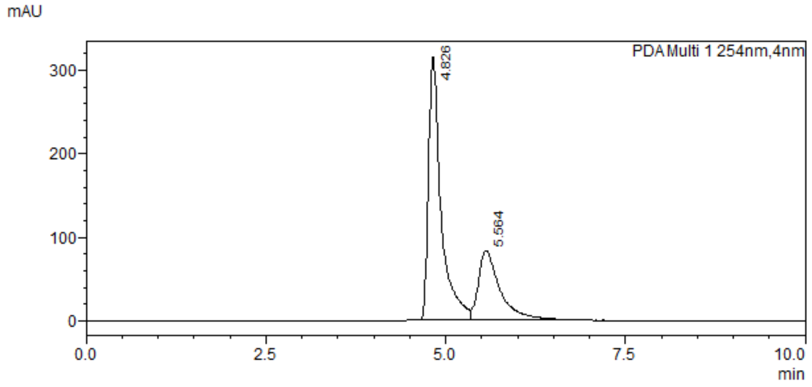

PDA Ch1 254nm

Peak#	Ret. Time	Area%
1	4.826	68.864
2	5.564	31.136
Total		100.000

(b)

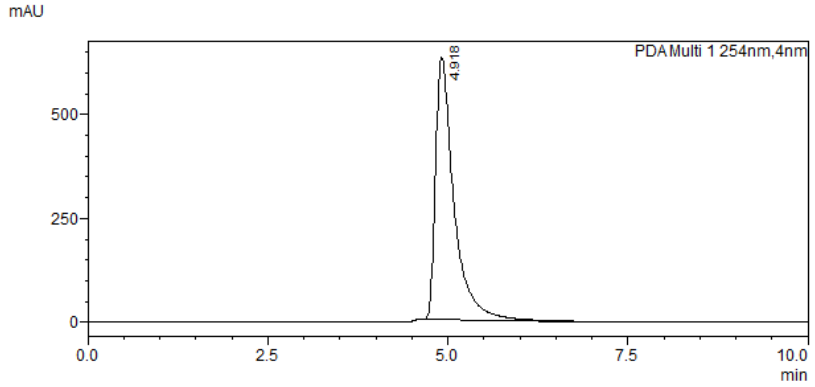

PDA Ch1 254nm

Peak#	Ret. Time	Area%
1	4.918	100.000
Total		100.000

(c)

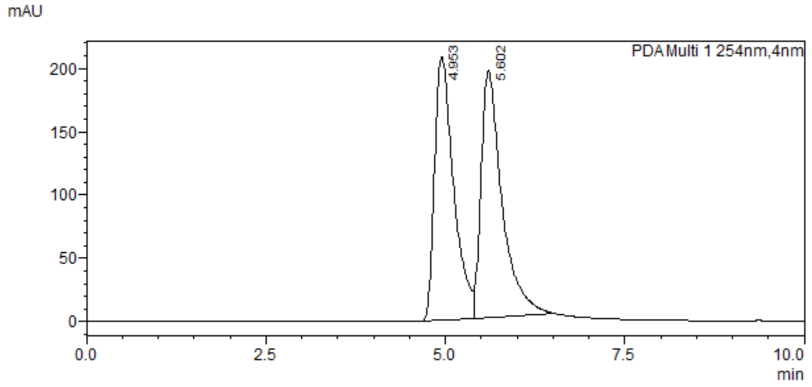

PDA Ch1 254nm

Peak#	Ret. Time	Area%
1	4.953	49.321
2	5.602	50.679
Total		100.000

Figure S3: HPLC traces for (a) partially enantioenriched (*S*)-**3d**; (b) **3d** in DCM solution; (c) **3d** precipitated from DCM.

NMR Investigations

[(*S*)-**3d**(F)]$^-$[Bu_4N]$^+$ Complex Assignment

Sample Preparation: (*S*)-**3d** (0.0125 mmol), Bu_4N^+ BF_4^- (1 equiv.) and KF (10 equiv.) were weighed into an NMR tube and *d*$_2$-DCM (0.5 mL) was added. The tube was sealed and sonicated for 45 minutes to form the complex.

NMR spectra of [(*S*)-**3d**(F)]$^-$[Bu_4N]$^+$ complex was recorded on an AVIII HD 500 spectrometer at 243 K.

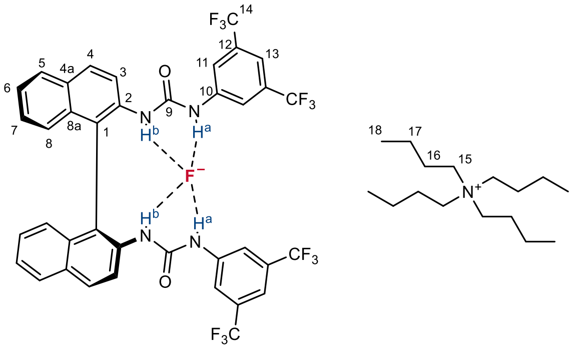

^{1}H	δ [ppm]	Multiplicity ^{n}J [Hz]
**NH(a)**	11.59	d, $^{1h}J_{FH}$ = 60
**NH(b)**	9.32	d, $^{1h}J_{FH}$ = 28
**3**	8.20	d, $^{3}J_{HH}$ = 9.1
**11**	7.75	s
**5**	7.47	d, $^{3}J_{HH}$ = 8.1
**13**	7.38	s
**6**	7.16	t, $^{3}J_{HH}$ = 7.4
**4 and 7**	7.07 – 6.99	overlapping
**8**	6.81	d, $^{3}J_{HH}$ = 8.6
**15**	3.02 – 2.88	m
**16**	1.53 – 1.40	m
**17**	1.25	sextet, $^{3}J_{HH}$ = 7.3
**18**	0.90	t, $^{3}J_{HH}$ = 7.3

^{19}F	δ [ppm]	Multiplicity ^{n}J [Hz]
**CF_3**	-62.4	s
**F^-**	-96.7	tt, $^{1h}J_{FH}$ = 60, 28

^{13}C	δ [ppm]	Multiplicity ^{n}J [Hz]
**9**	154.5	s
**10**	143.3	s
**2**	137.9	s
**8a**	132.4	s
**12**	130.4	q, $^{2}J_{CF}$ = 32.4
**4a**	129.5	s
**5**	127.8	s
**4**	127.3	s
**7**	125.8	s
**8**	124.3	s
**14**	123.9	q, $^{1}J_{CF}$ = 272.6
**6**	123.4	s
**3**	123.1	s
**1**	119.8	s
**11**	118.0	s
**13**	112.6	s
**15**	58.3	s
**16**	23.5	s
**17**	19.6	s
**18**	13.4	s

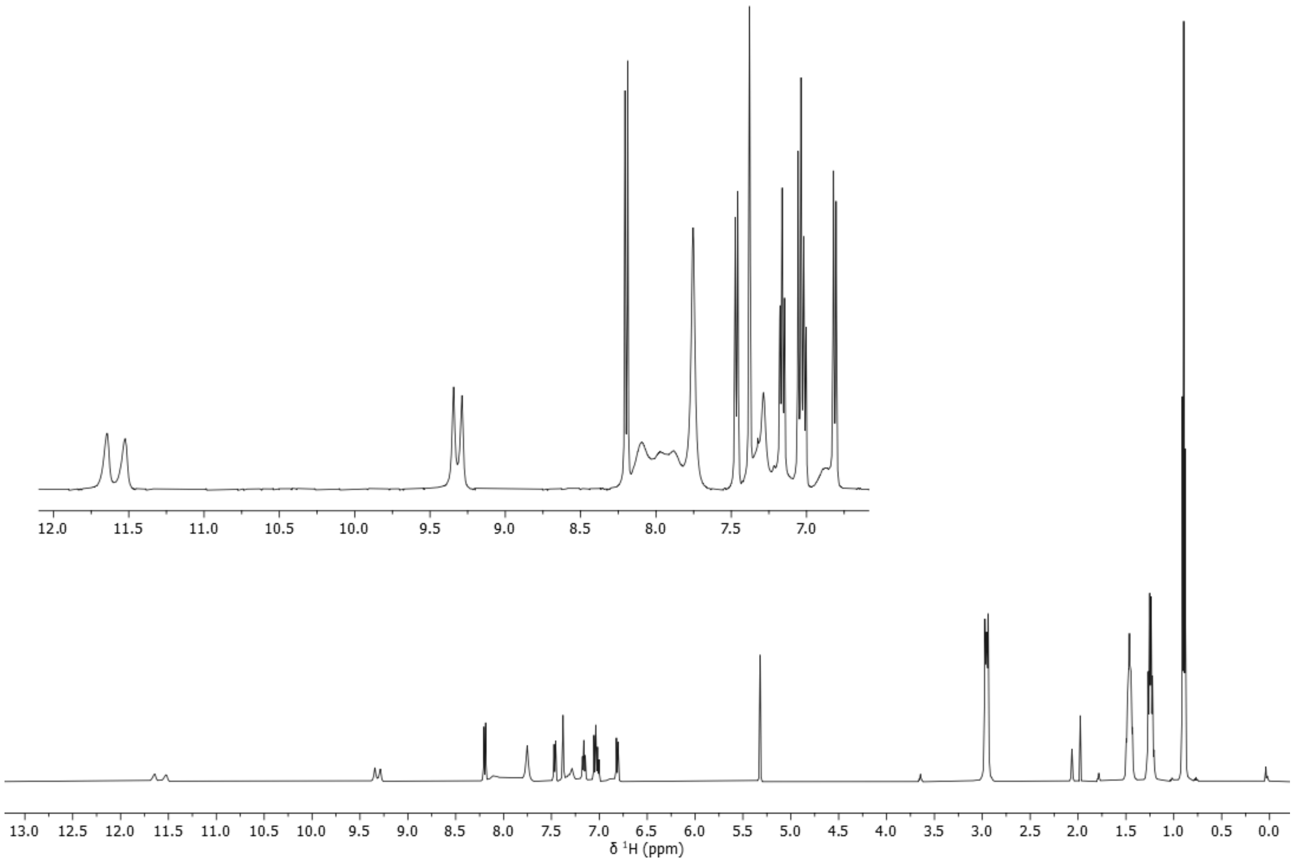

Figure S4: ^{1}H NMR of [(*S*)-**3d**(F)]$^{-}$[Bu_4N]$^{+}$ complex (500 MHz, d_2-DCM, 25 mM, 243 K).

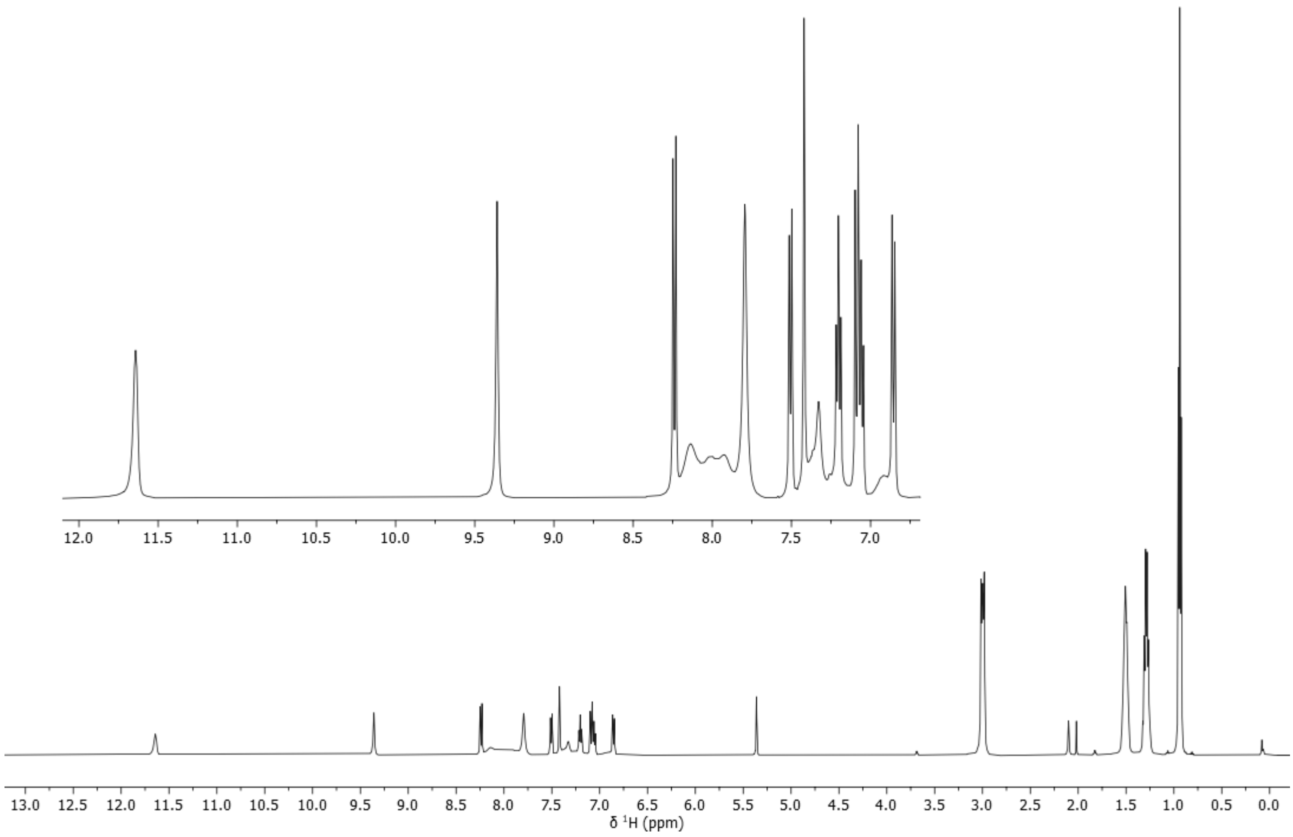

Figure S5: $^{1}H\{^{19}F\}$ NMR of [(*S*)-**3d**(F)]$^{-}$[Bu_4N]$^{+}$ complex (500 MHz, d_2-DCM, 25 mM, 243 K).

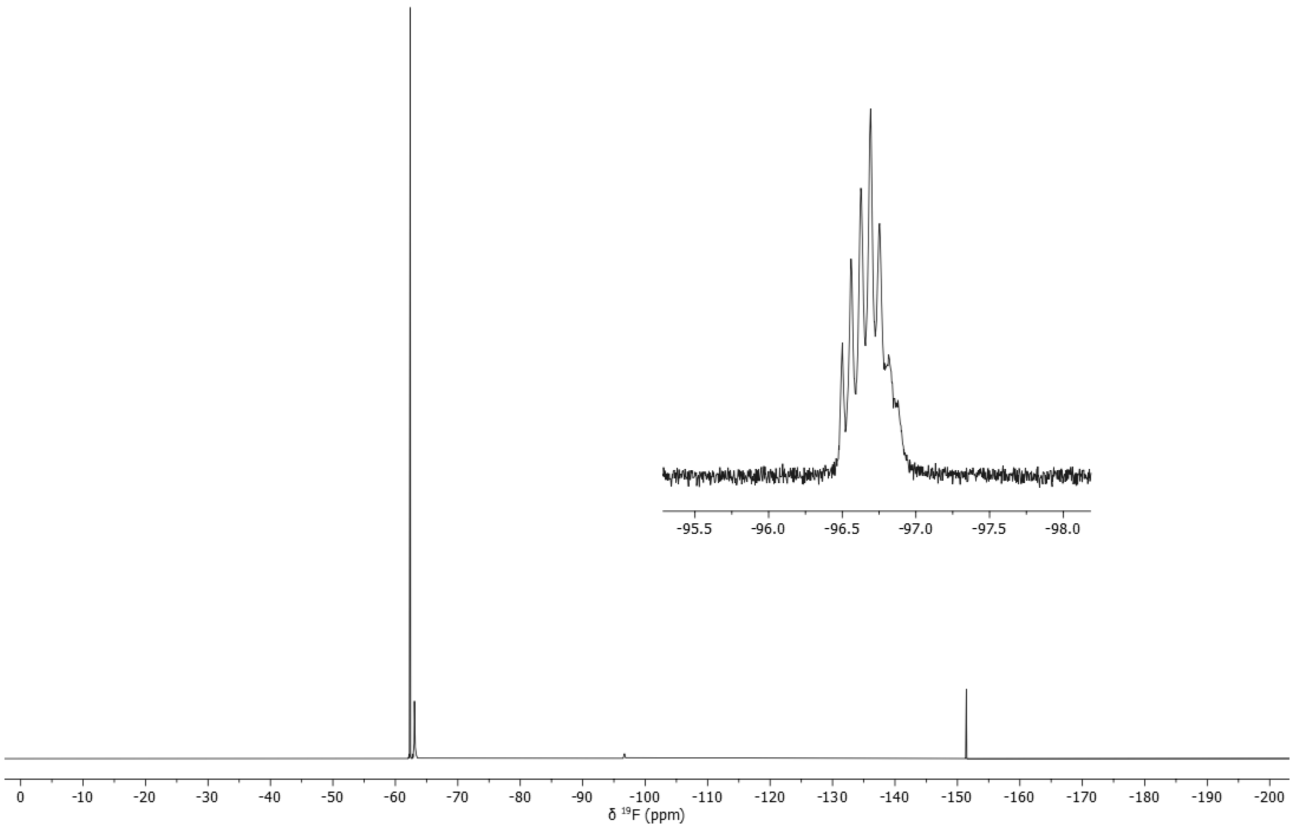

Figure S6: ^{19}F NMR of [(*S*)-**3d**(F)]$^{-}$[Bu_4N]$^{+}$ complex (470 MHz, d_2-DCM, 25 mM, 243 K).

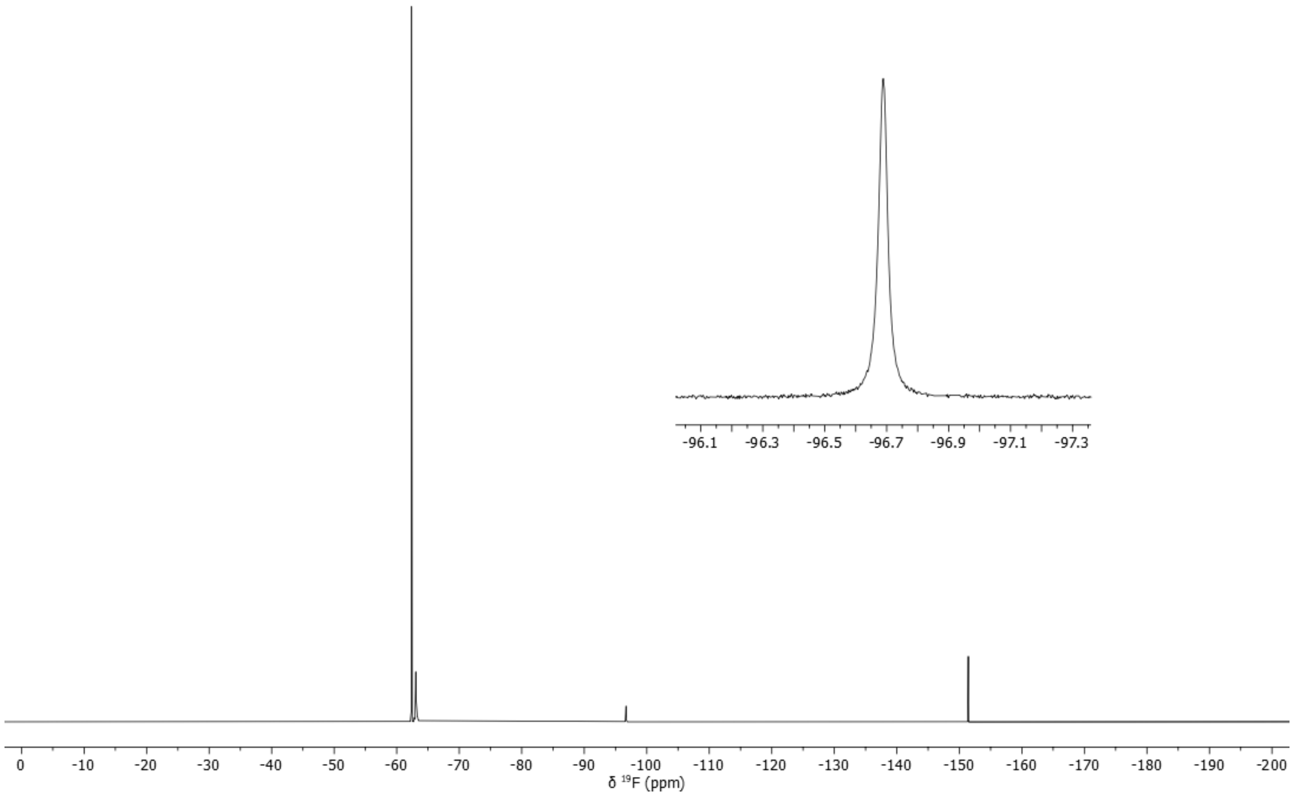

Figure S7: $^{19}F\{^{1}H\}$ NMR of [(*S*)-**3d**(F)]$^{-}$[Bu_4N]$^{+}$ complex (470 MHz, d_2-DCM, 25 mM, 243 K).

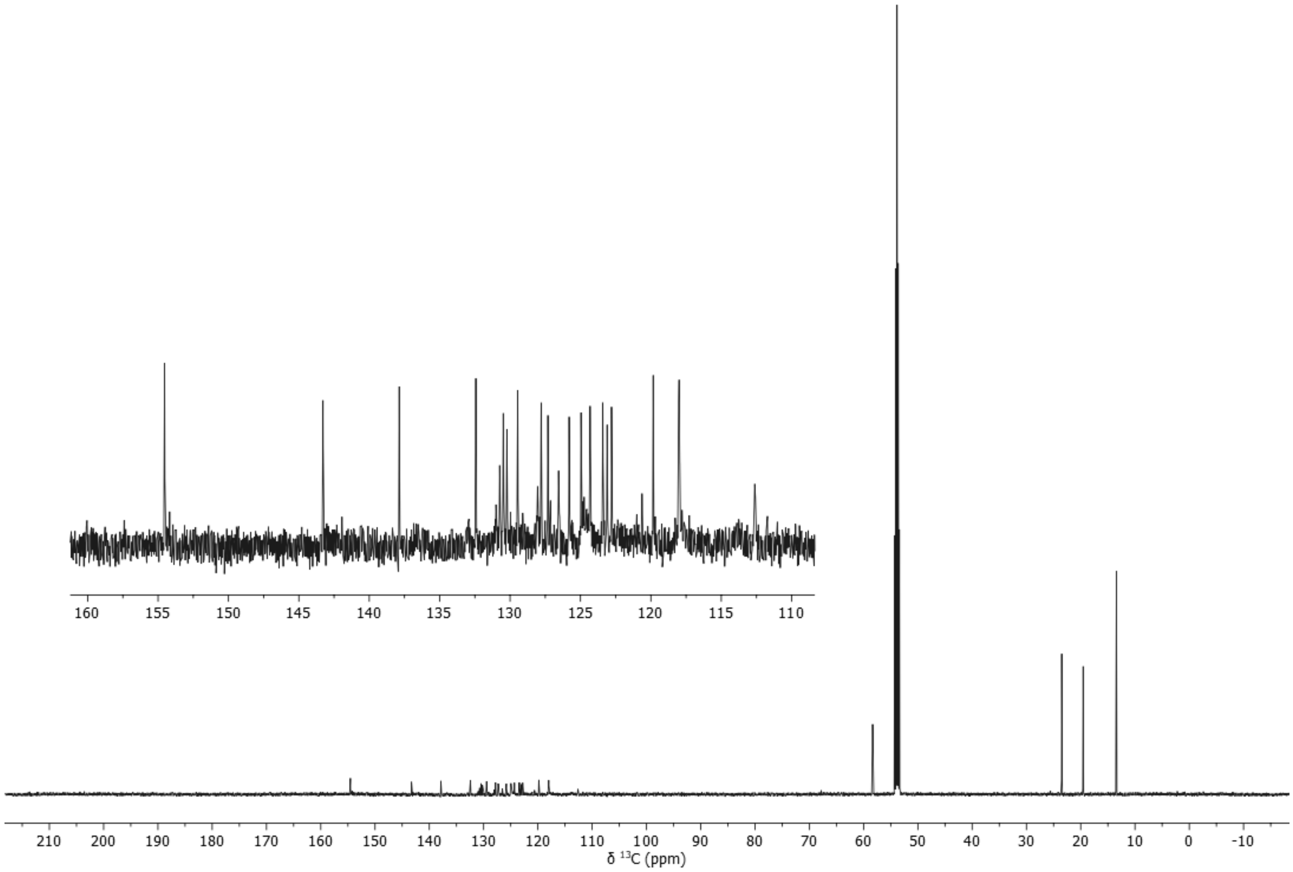

Figure S8: ^{13}C NMR of [(*S*)-**3d**(F)]$^{-}$[Bu_4N]$^{+}$ complex (126 MHz, d_2-DCM, 25 mM, 243 K).

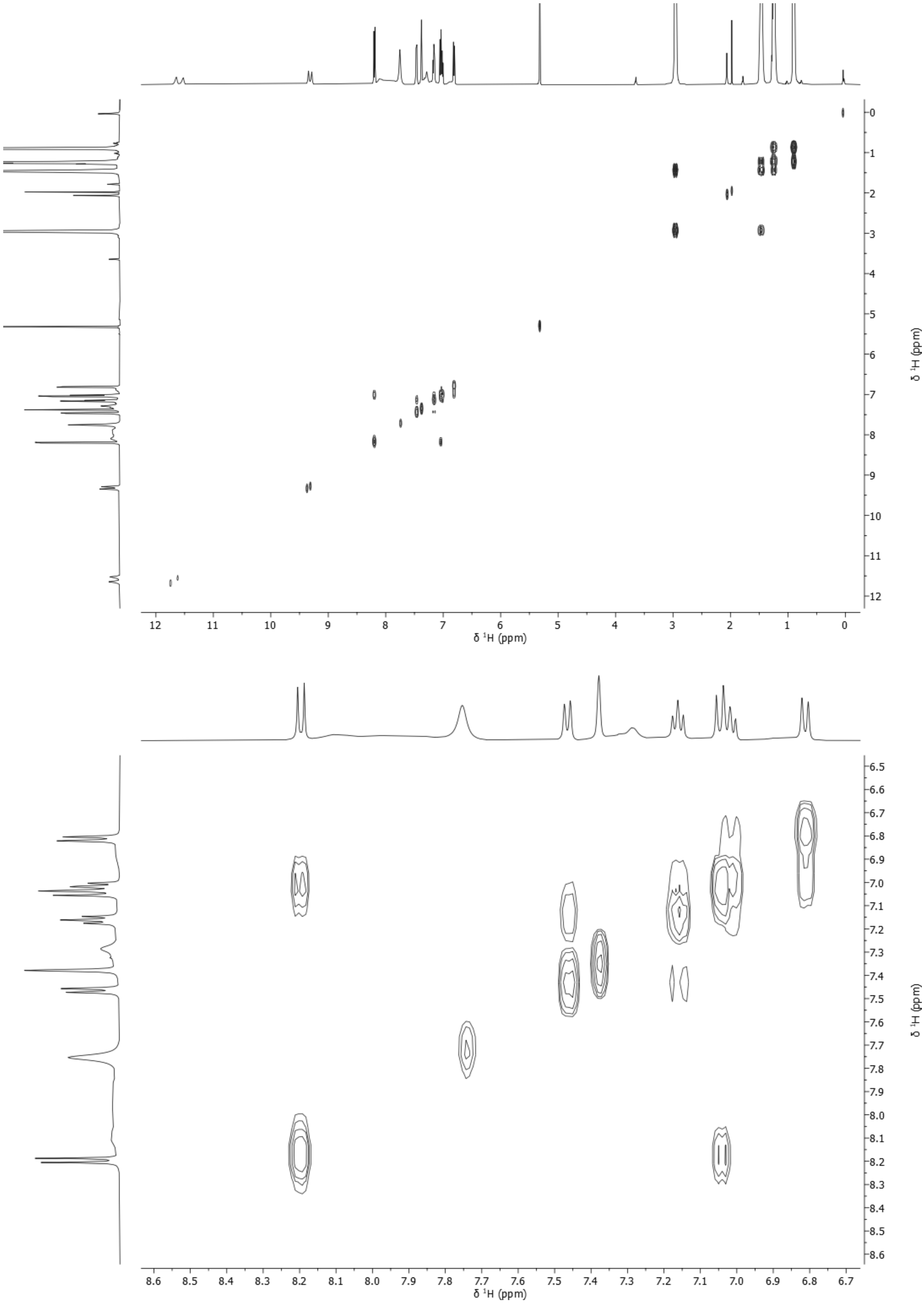

Figure S9: ¹H COSY of [(*S*)-**3d**(F)]⁻[Bu_4N]⁺ complex (500 MHz, d_2-DCM, 25 mM, 243 K).

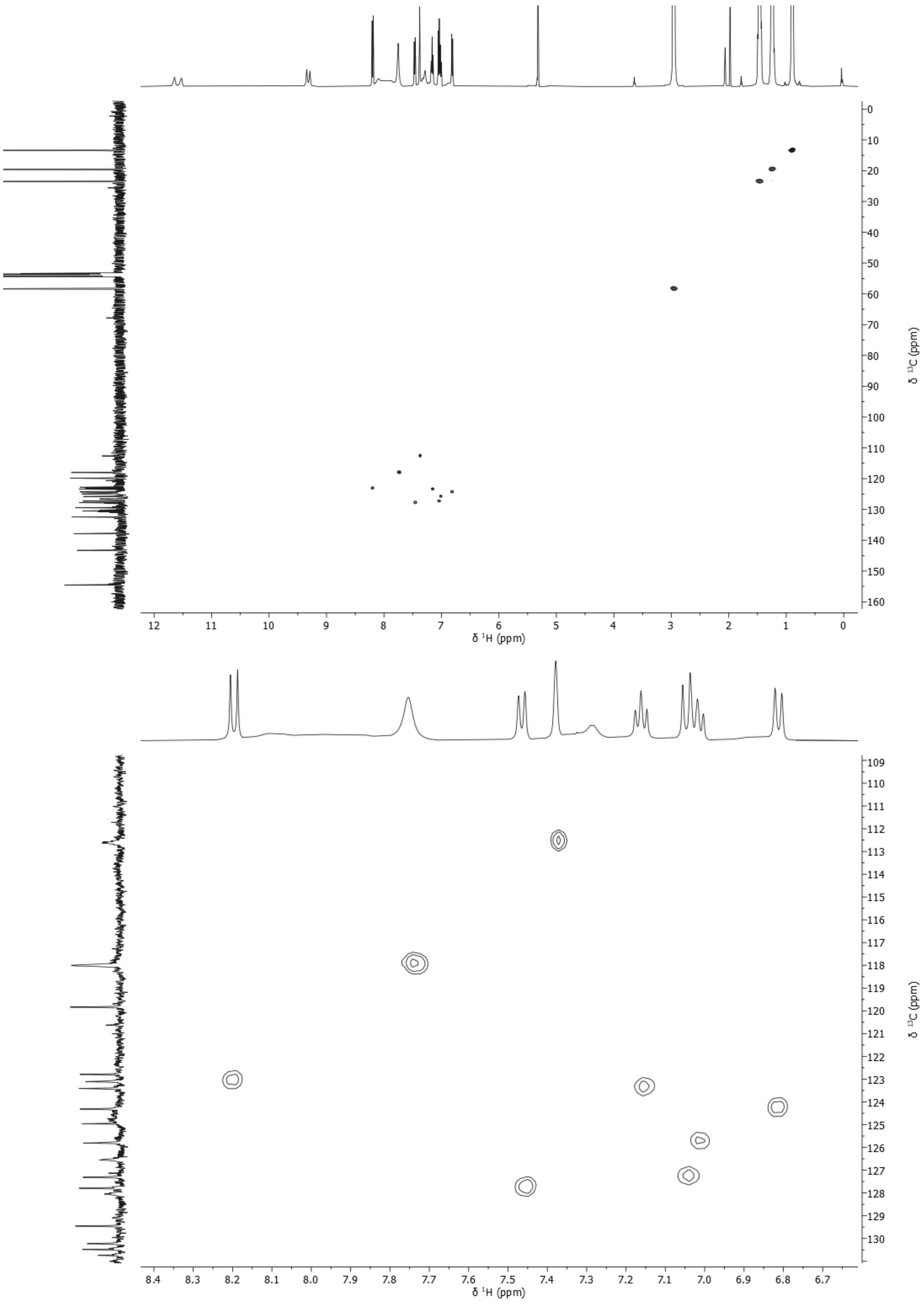

Figure S10: ^{1}H-^{13}C HSQC of [(*S*)-**3d**(F)]$^{-}$[Bu_4N]$^{+}$ complex (500 MHz, d_2-DCM, 25 mM, 243 K).

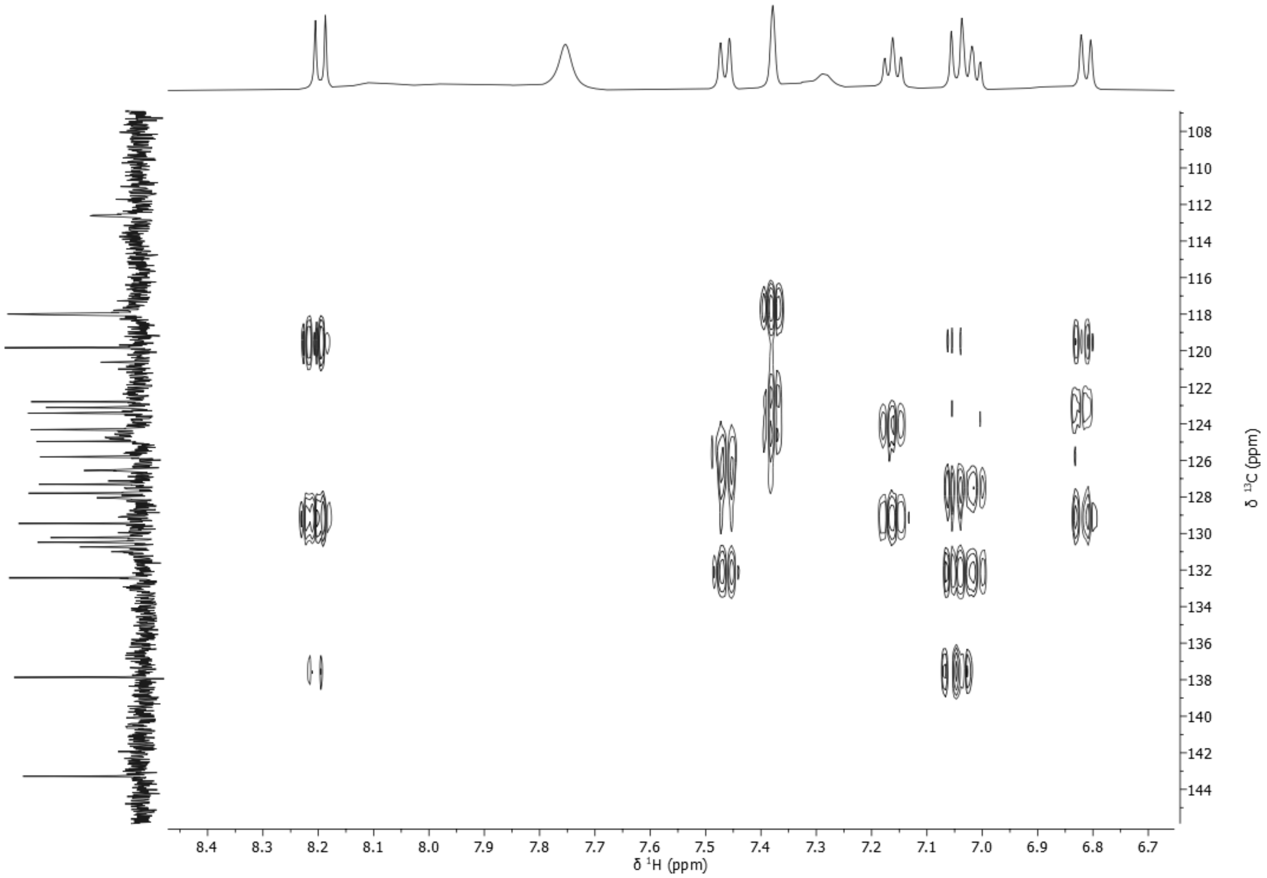

Figure S11: ^{1}H-^{13}C HMBC of [(*S*)-**3d**(F)]$^{-}$[Bu$_4$N]$^{+}$ complex (500 MHz, *d*$_2$-DCM, 25 mM, 243 K).

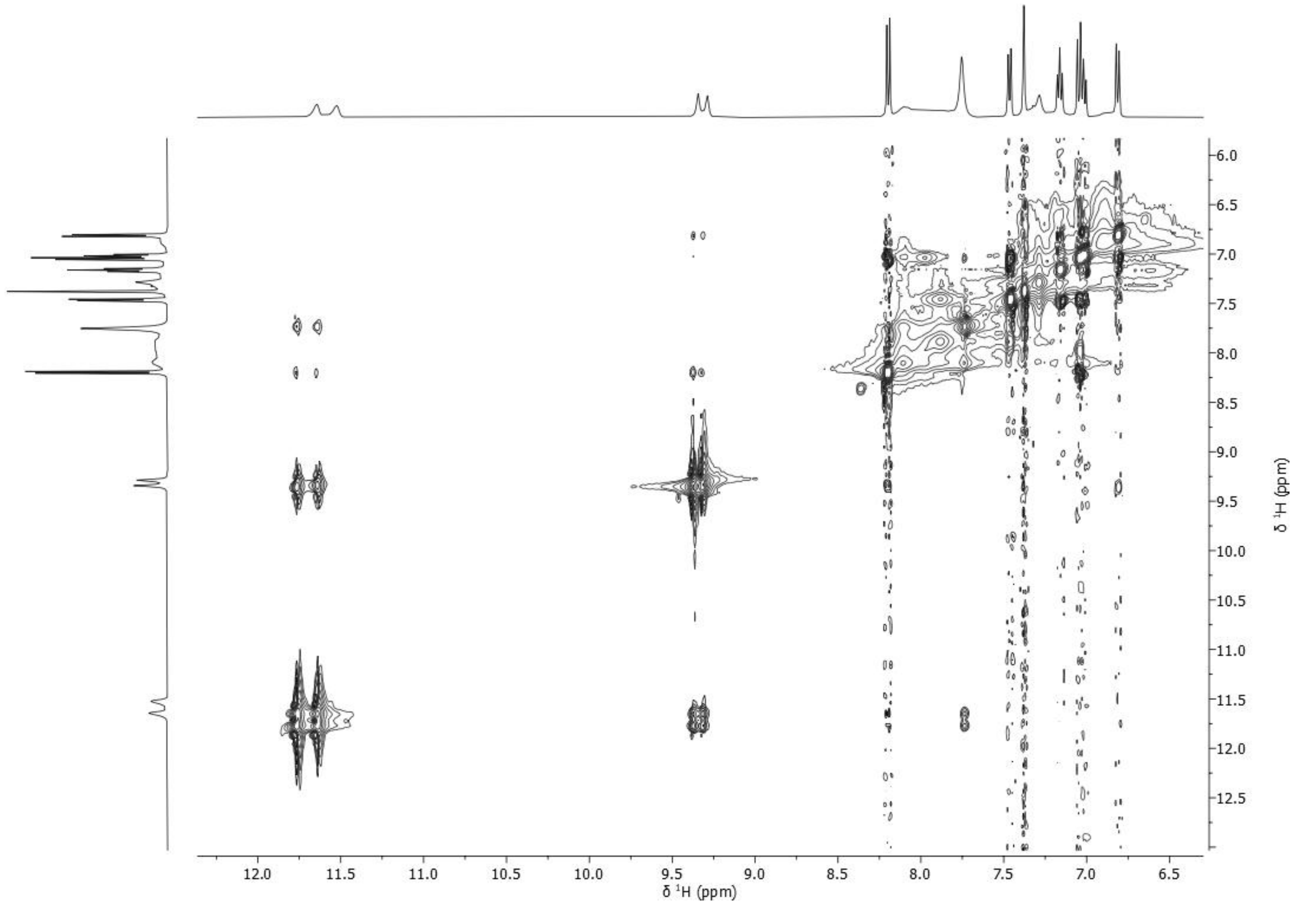

Figure S12: ^{1}H ROESY of [(*S*)-**3d**(F)]$^{-}$[Bu$_4$N]$^{+}$ complex, spin lock pulse (p15) = 100 ms (500 MHz, *d*$_2$-DCM, 25 mM, 243 K).

^{1}H-^{19}F CLIP-HSQC

^{1}H-^{19}F CLIP-HSQC experiments were recorded on an AVIII HD 500 using a 25 mM sample of [(*S*)-**3d**(F)]$^{-}$ [Bu$_4$N]$^{+}$ complex in d_2-DCM at 243 K. The experiment was acquired using pulse sequence (*hsqcetgpclip*)[20] under the following parameters:

Parameter	Value
**TD**	2048 (f2) × 128 (f1)
**SW**	10 (f2) × 6 (f1)
**o1p/ o2p**	10 / -96.66 ppm
**NS (DS)**	4 (4)
**relaxation delay d1**	2 s
**delay for evolution of coupling CNST[2]**	48

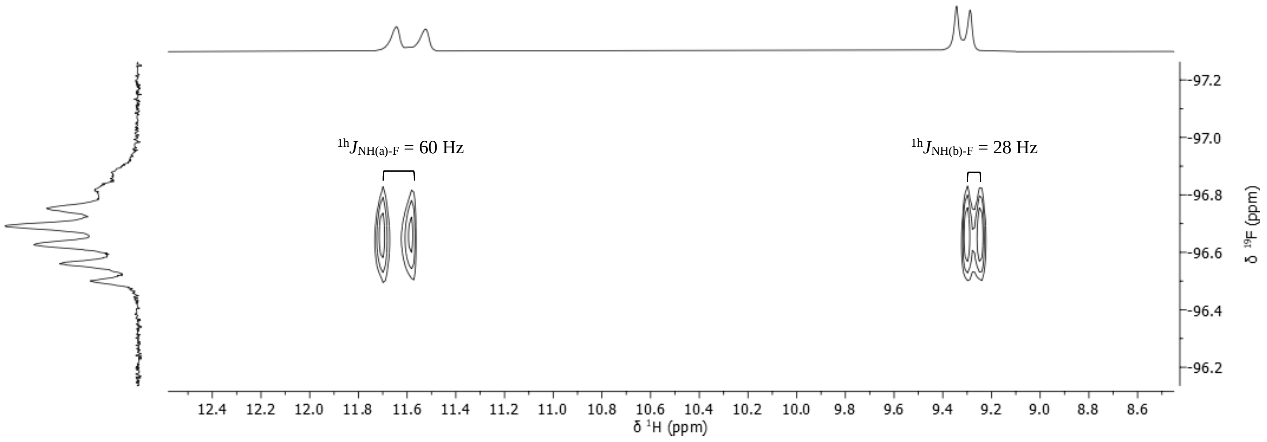

Figure S13: ^{1}H-^{19}F CLIP-HSQC of [(*S*)-**3d**(F)]$^{-}$[Bu$_4$N]$^{+}$ complex (500 MHz, d_2-DCM, 25 mM, 243 K).

Control experiments investigating interaction between (*S*)-3d and [Rh(cod)Cl]$_2$

(*S*)-**3d** (7.95 mg, 0.01 mmol) was weighed into an NMR tube and dissolved in d_2-DCM (0.5 mL, 20 mM). ^{1}H, ^{13}C, and ^{19}F NMR of this solution were recorded on an AVIII HD 500 spectrometer at 298 K. Subsequently, [Rh(cod)Cl]$_2$ (1.24 mg, 2.5 µmol) was directly added into the same NMR tube. The content was mixed until a homogeneous solution was obtained. ^{1}H, ^{13}C, and ^{19}F NMR spectra were then recorded similarly and compared. No significant difference in chemical shifts was observed.

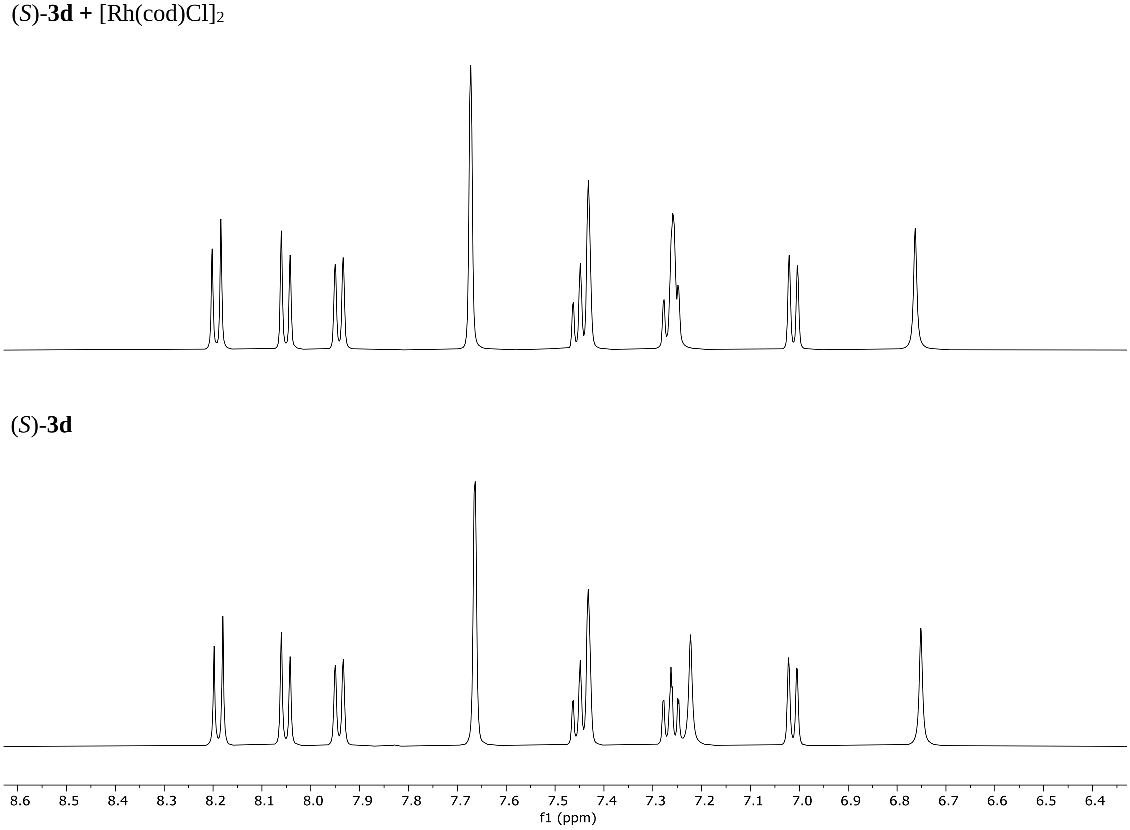

Figure S14: Comparison of ^{1}H NMR (500 MHz, d_2-DCM, 298 K).

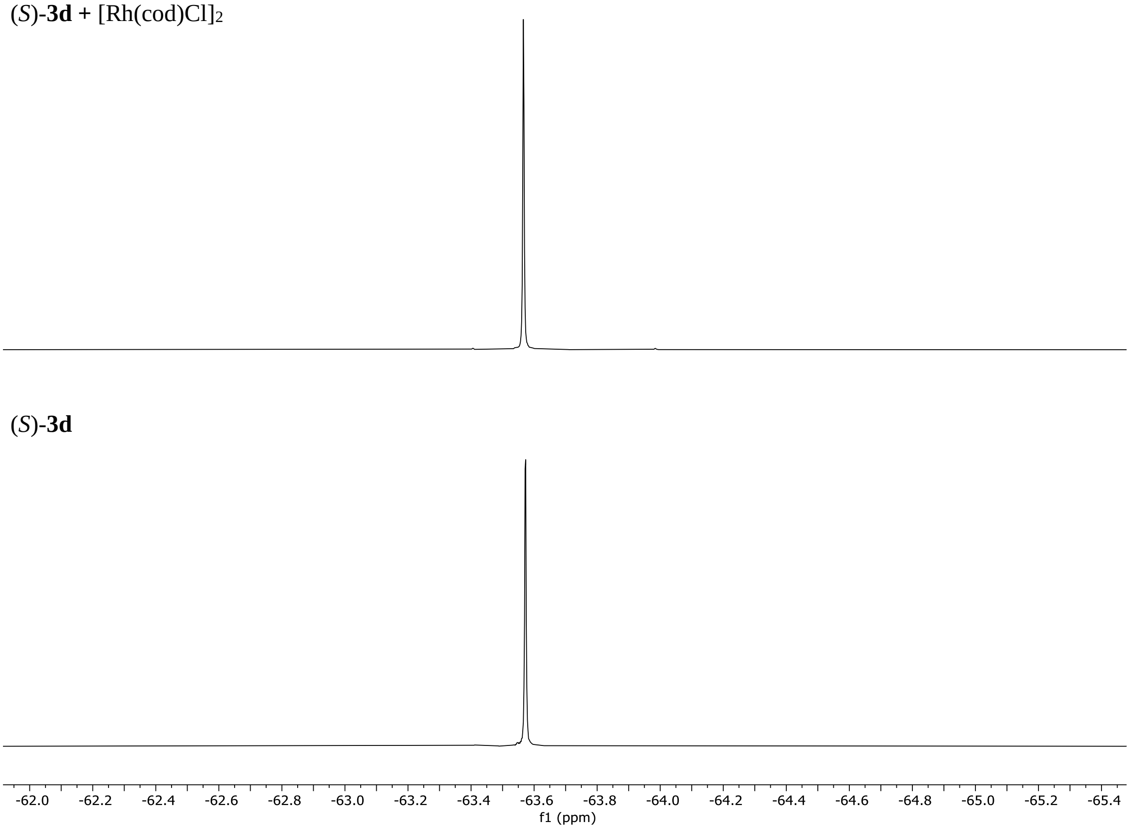

Figure S15: Comparison of ^{19}F NMR (471 MHz, d_2-DCM, 298 K).

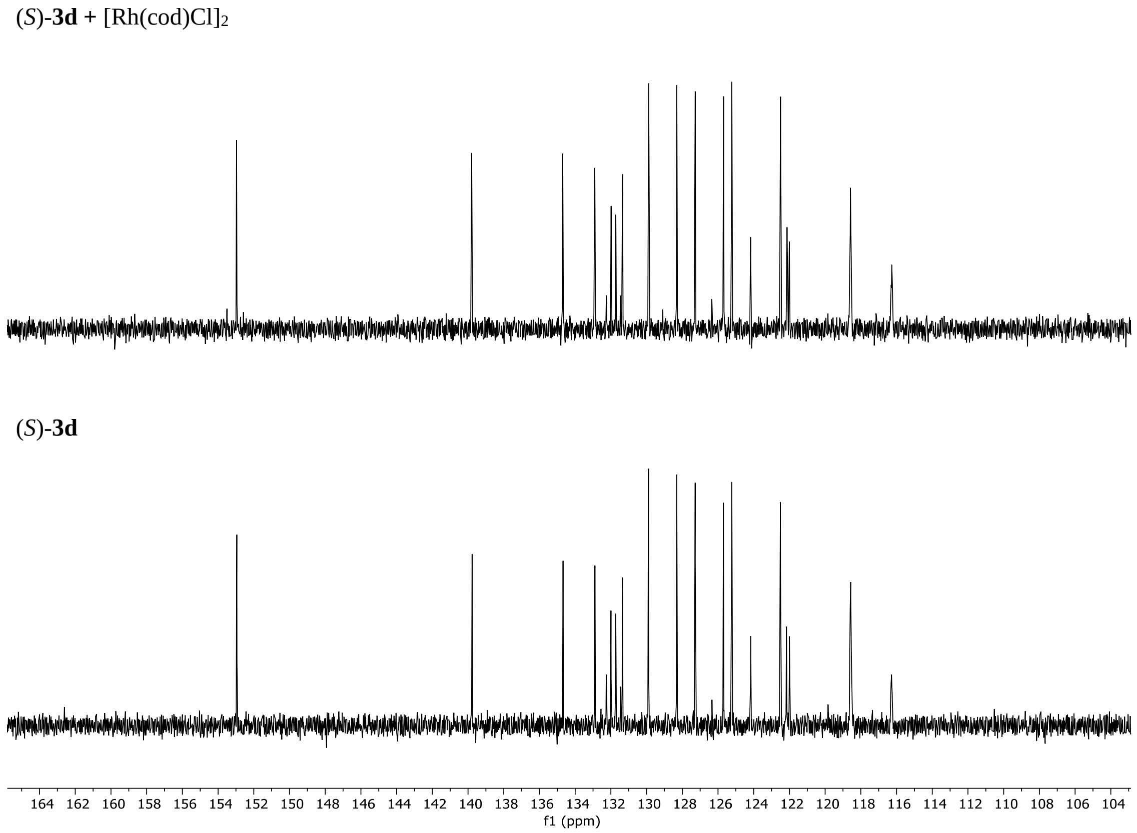

Figure S16: Comparison of ^{13}C NMR (126 MHz, d_2-DCM, 298 K).

Control Reactions

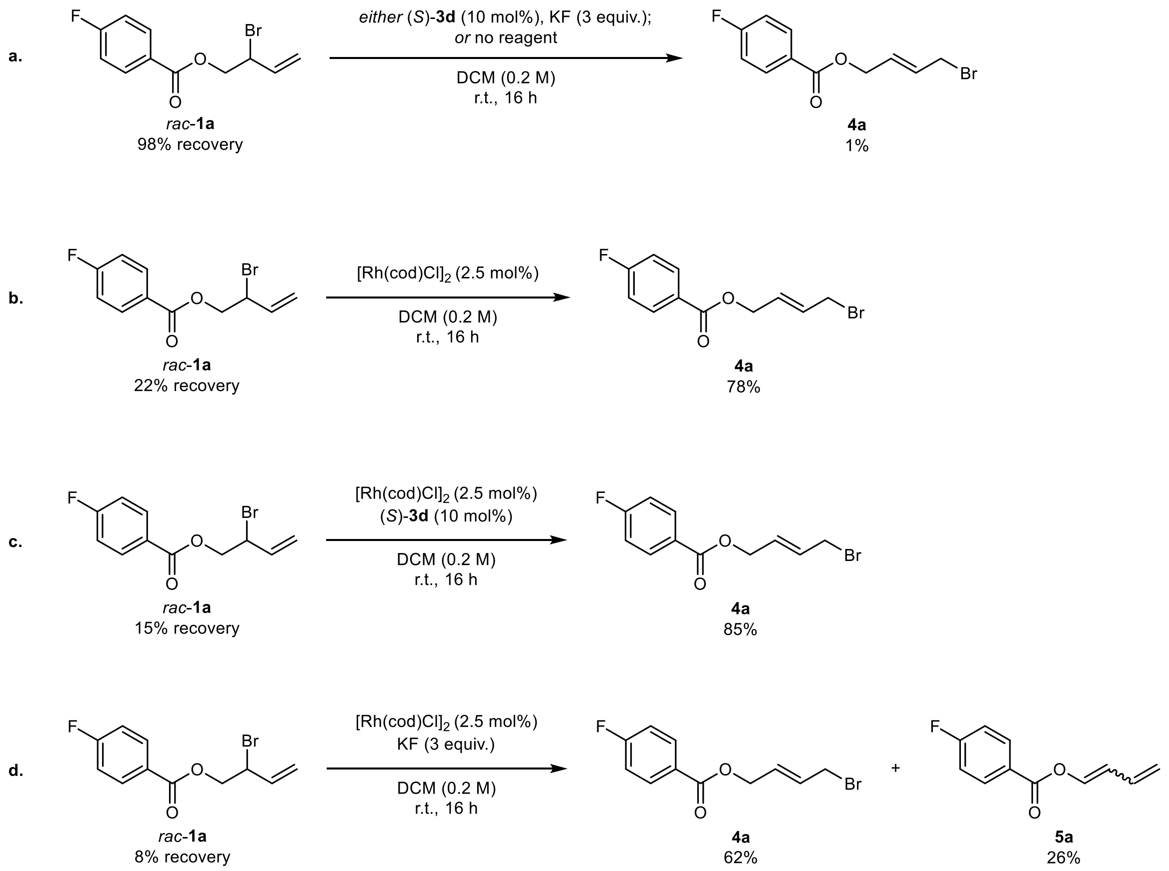

Figure S17: Control reactions to study substrate isomerization. Yields determined by ^{19}F NMR using 4-fluoroanisole as internal standard.

Enantiopure substrate (*R*)-**1a** was prepared in an analogous way to *rac*-**1a** from (*S*)-but-3-ene-1,2-diol, and subjected to the following conditions:

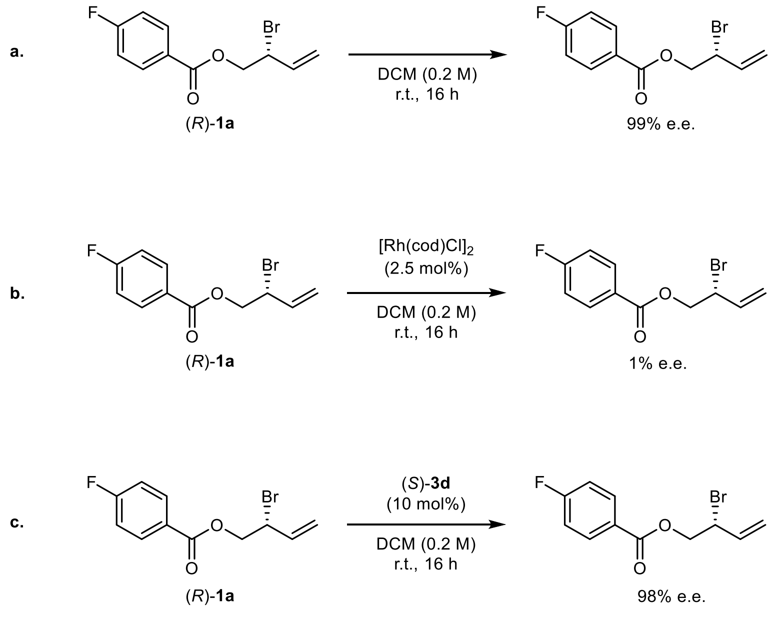

Figure S18: Control reactions to study substrate racemisation. e.e. determined by chiral HPLC.

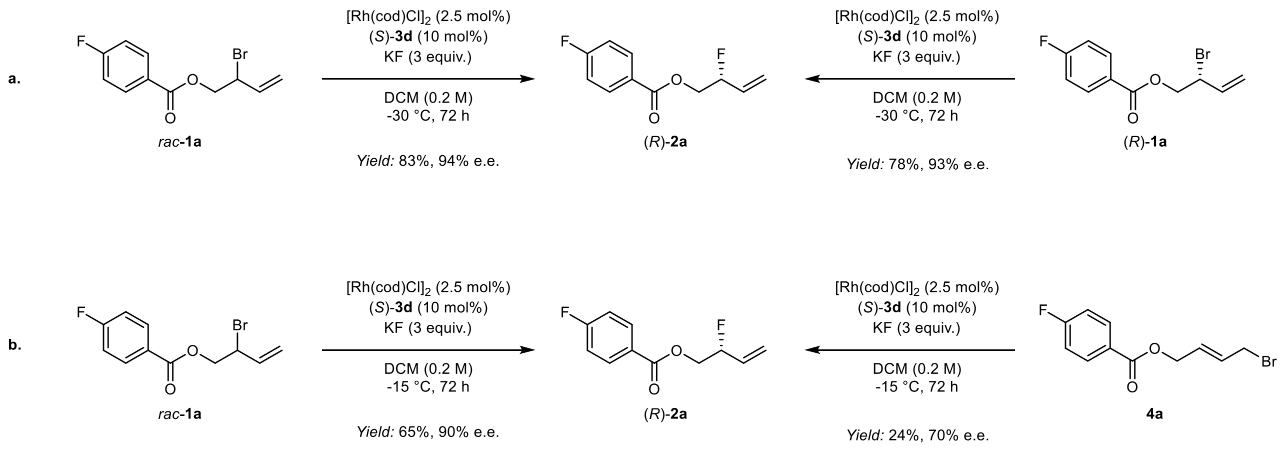

Figure S19: Subjecting enantiopure substrate (*R*)-**1a** and linear bromide **4a** respectively to the standard conditions. Yields determined by ^{19}F NMR using 4-fluoroanisole as internal standard. e.e. determined by chiral HPLC.

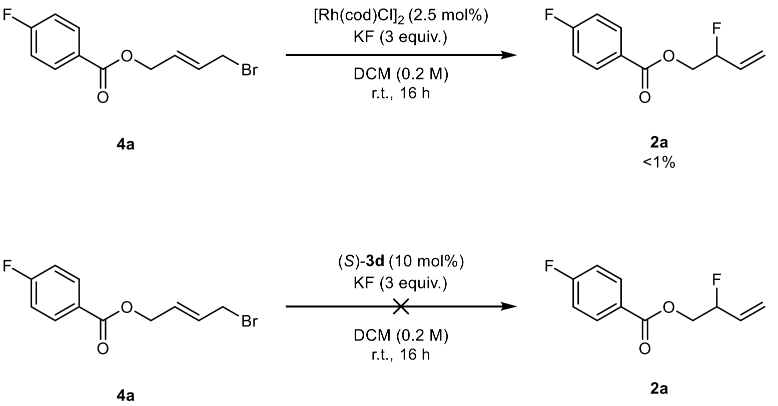

Figure S20: Control reactions on the background reactivity of linear bromide **4a**.

Di-alkylated urea catalysts (*S*)-**3k** and (*S*)-**3l** did not afford any fluorination products, illustrating the requirement of at least three NH motifs as hydrogen bond donor:

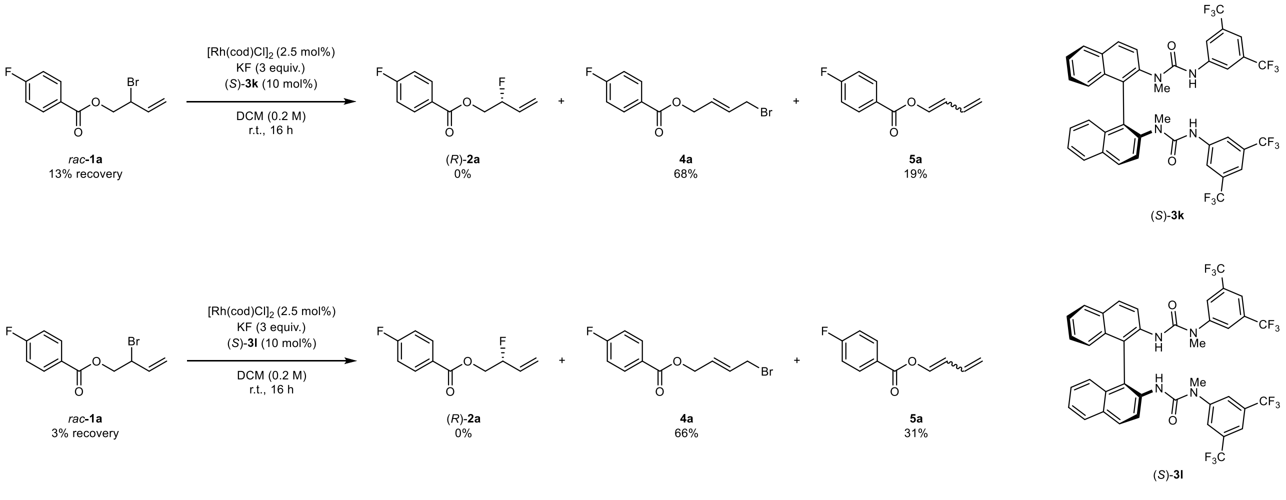

Figure S21: Control reactions using di-alkylated *bis*-ureas.

Monitoring Product Distribution and Enantiomeric Ratios over Time

Table S9: *Ex situ* monitoring of the yield and enantiomeric ratio (if applicable) of **1a**, **2a** and **4a** over the time-course of reaction

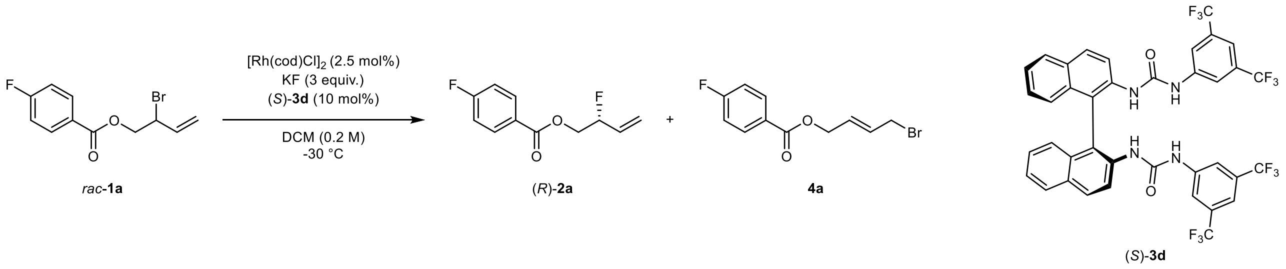

Entry	Time (h)	2a yield (%)[a]	1a (%)[a]	4a (%)[a]	2a e.r.[b]	1a e.r.[b]
**1**	0	0	100	0	n.d.	50:50
**2**	1.5	2	74	24	n.d.	48:52
**3**	5	5	72	21	93:7	49:51
**4**	22	25	43	29	96:4	49:51
**5**	30	34	36	29	97:3	51:49
**6**	48	55	10	30	97:3	50:50
**7**	72	74	0	24	97:3	n.d.

General conditions: Substrate (0.1 mmol), urea catalyst (*S*)-**3d** (10 mol%), $[Rh(cod)Cl]_2$ (2.5 mol%) and KF (3 equiv.) in 500 µL of DCM stirred at 1200 rpm. [a]Determined by ^{19}F NMR using 4-fluoroanisole as internal standard. [b]e.r. was determined by HPLC analysis using a chiral stationary phase. n.d. = not determined.

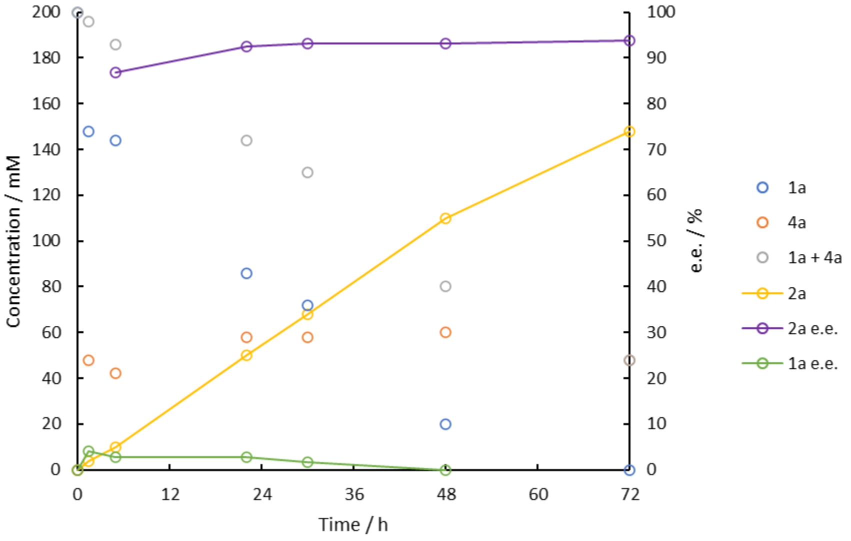

Figure S22: Plot of the yield and enantiomeric excess (if applicable) of **1a**, **2a** and **4a** over time.

We hypothesize that there is an induction period for phase transfer as KF cannot be brought into solution in DCM by the urea catalyst alone. Instead, the process relies on halide exchange with a bromide ion and the precipitation of KBr, which provides sufficient driving force. As such, displacement of bromide by Rh has to precede KF solubilization. Isomerization from **1a** to **4a** may therefore take place to a significant extent before fluorination starts.

Kinetic Experiments

T_1 Measurements

The longitudinal relaxation time constants T_1 of the allyl fluoride product **2a**, allyl bromide substrate **1a**, and internal standard 4-fluoroanisole were measured *via* ^{19}F inversion recovery experiments (t1ir) on an AVIII HD 500. T_1 values were calculated by non-linear fitting using Dynamic centre on Topspin version 4.4.0. The results are summarised in Table S11.

Sample preparation: A sample of **1a** (10 µmol), **2a** (10 µmol), (*S*)-**3d** (2 µmol), and internal standard 4-fluoroanisole (1 µL) was prepared in CH_2Cl_2 (100 µL) and $CDCl_3$ (450 µL). The experiment was acquired with the following parameters.

Table S10: Parameters for ^{19}F T_1 measurement:

Parameter	Value
**TD**	65536
**SW**	40 / 20
**O1P**	-115.0 / -186
**NS (DS)**	2 (2)
**Relaxation delay d1**	45 s
**Variable delay d1**	0.001 – 60 s (12)

Table S11: Summary of T_1 measurements

Compound	^{19}F T_1 (s)
**1a**	3.458
**2a**	3.602 (-105.3 ppm)
	3.856 (-186.1 ppm)
**4-fluoroanisole**	5.724

t_{D1} value of 30.0 s was selected for quantitative ^{19}F NMR.

Isomerisation kinetics: continuous *in situ* monitoring

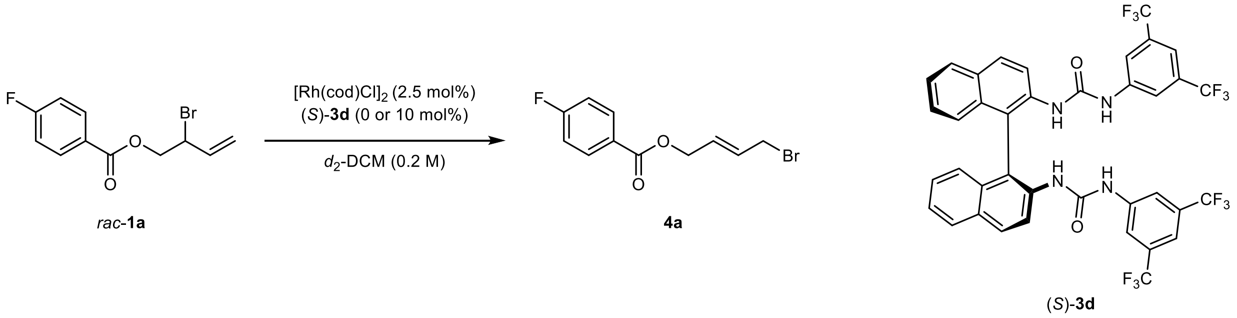

1a (27.3 mg, 0.1 mmol, 1 equiv.), (*S*)-**3d** (8.0 mg, 10 mol%), and 4-fluoroanisole (internal standard, 5 µL) were dissolved in CD_2Cl_2 (0.5 mL) in a J-Young's valve NMR tube. The NMR tube was sealed and analysed by quantitative $^{19}F\{^1H\}$ NMR (90° excitation pulse (zgig), relaxation delay of t_{D1} = 30.0 s, NS (DS) = 8 (4), o1p = -115 ppm, SW = 40 ppm, 298 K) to obtain *t* = 0 spectrum.

$[Rh(cod)Cl]_2$ (1.2 mg, 2.5 mol%) was added to the NMR tube as a solid and quickly mixed at room temperature, noting the starting time of the reaction. A similar $^{19}F\{^1H\}$ NMR spectrum was recorded approximately every 30 min for a total of 13–14 h. Throughout the experiment, the NMR tube was left inside the spectrometer at 298 K. Concentrations of **1a** and **4a** were calculated by comparison of the corresponding integral to that of the internal standard.

The same procedure was repeated in the absence of (*S*)-**3d**. Both experiments were conducted in duplicates.

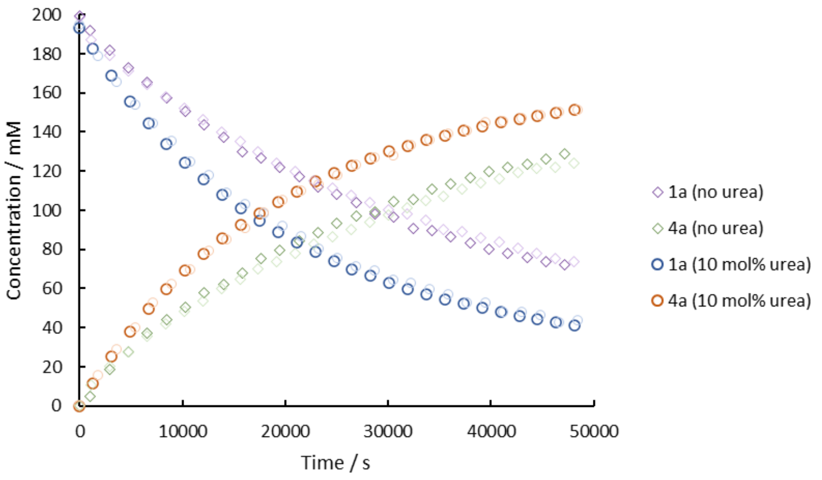

Figure S23: Plot of the concentrations of **1a** and **4a** over time in the presence or absence of urea (*S*)-**3d**. Duplicate experiment in each case was shown in a lighter colour.

General procedure for *ex situ* sampling

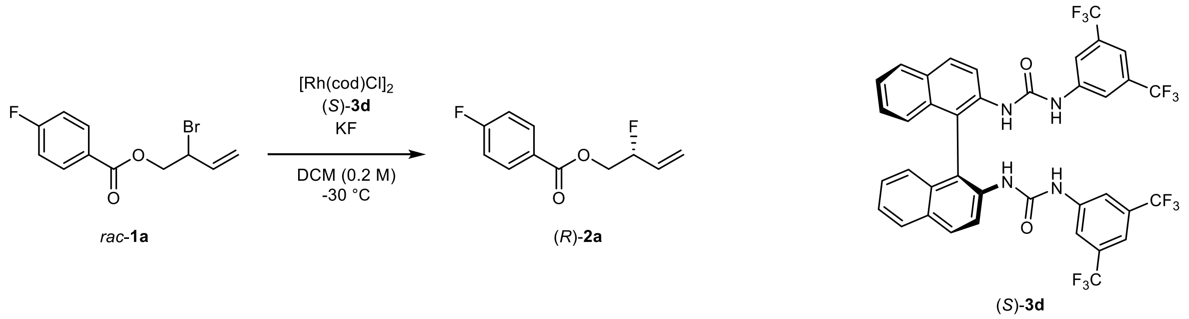

1a, (*S*)-**3d**, $[Rh(cod)Cl]_2$, pre-ground KF, 4-fluoroanisole (internal standard), and DCM (0.2 M) were sequentially loaded into a microwave vial with a magnetic stirrer. (*S*)-**3d** and $[Rh(cod)Cl]_2$ were added either directly as a solid or as a stock solution in DCM. The vial was sealed and placed in a cryogenic bath at -30 °C. The reaction mixture was stirred at 1200 rpm unless otherwise specified.

At specified time points, a small aliquot was removed and diluted in $CDCl_3$ to a total volume of 550 μL. The mixture was directly filtered into an NMR tube through a 0.2 μm syringe filter to remove the KF, stopping the reaction. All aliquots were kept in -20 °C freezer until analysis by quantitative $^{19}F\{^1H\}$ NMR (90° excitation pulse (zgig), relaxation delay of t_{D1} = 30.0 s, NS (DS)= 128 (4), *either* o1p = -115 ppm, SW = 40 ppm *or* o1p = -155 ppm, SW = 120 ppm).

All NMR spectra were recorded on AVIIIHD 500 NMR spectrometer at 298 K. All spectra acquired were processed using Topspin version 4.4.0, with 128 K points zero filling, automatic phase correction and baseline correction. Examples and more details of spectral processing are included in later section. Each peak was integrated, and concentrations of substrates and products were calculated by comparison of the corresponding integral to that of internal standard.

Representative spectra and concentration-time profile

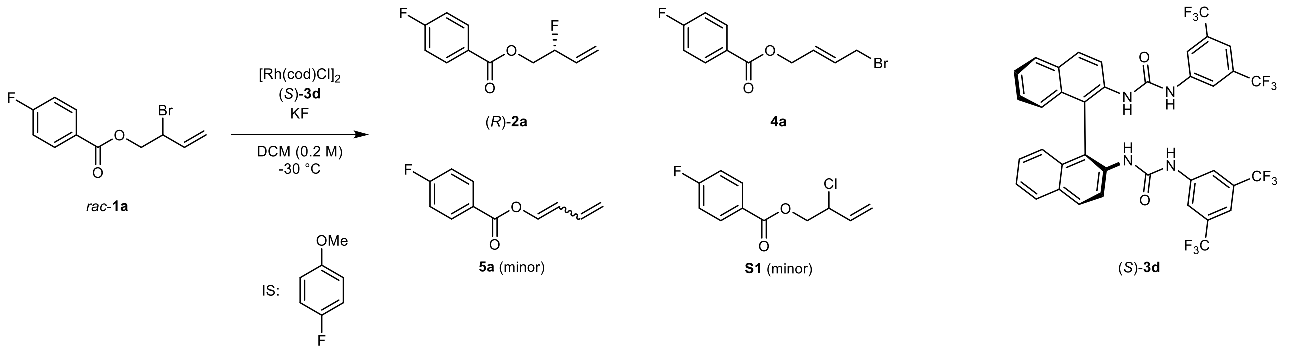

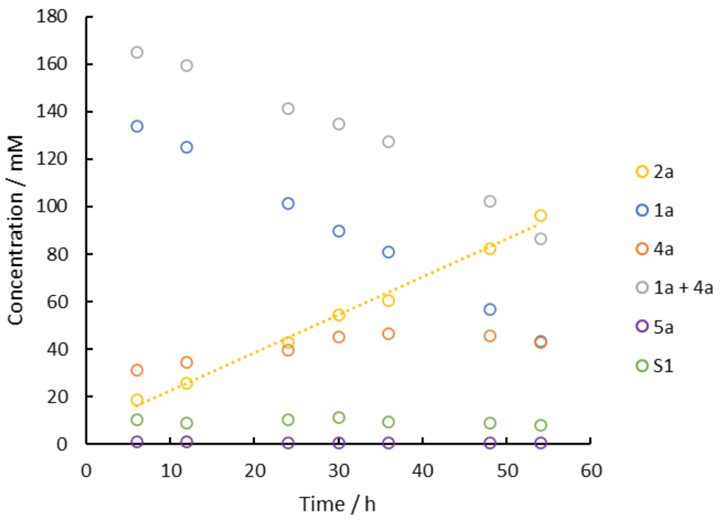

Figure S24: Kinetic profile of the reaction under standard conditions.

All spectra acquired were processed via the same method to minimise the error introduced. Typical $^{19}F\{^1H\}$ NMR spectra after automatic phase correction and baseline correction were shown in Figure S25. By referring to the integral of the internal standard, the concentrations of **2a**, **4a**, and **5a** (as an *E*/*Z* isomeric mixture) were calculated. The concentration of fluoride **2a** was calculated based on the peak at -186.1 ppm for greater

accuracy due to absence of any peak overlap. After performing line fitting on the region of -105.0 to -105.2 ppm, the peaks of **1a** and **S1** can be deconvoluted and their concentrations were calculated.

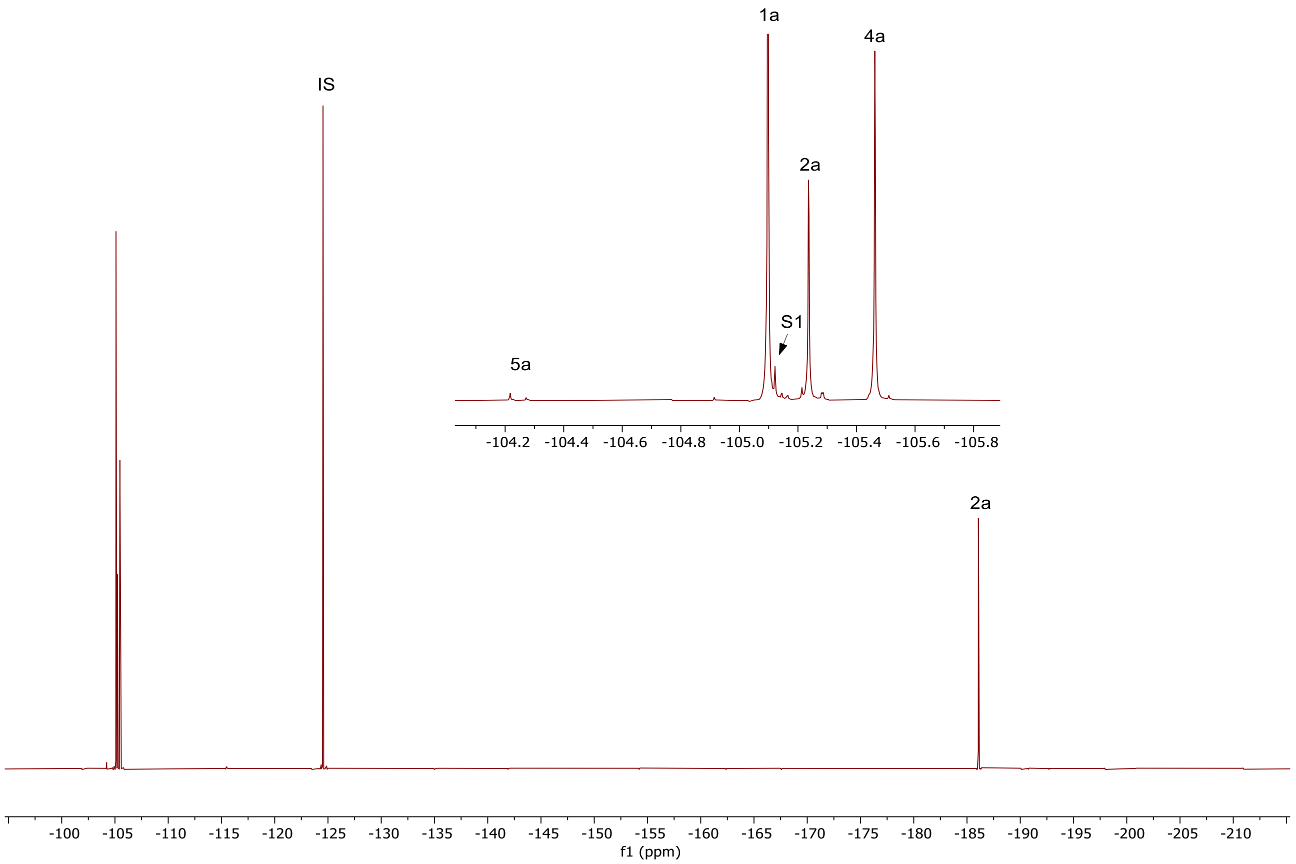

Figure S25: Typical $^{19}F\{^{1}H\}$ NMR spectrum of the reaction mixture at ~20% conversion.

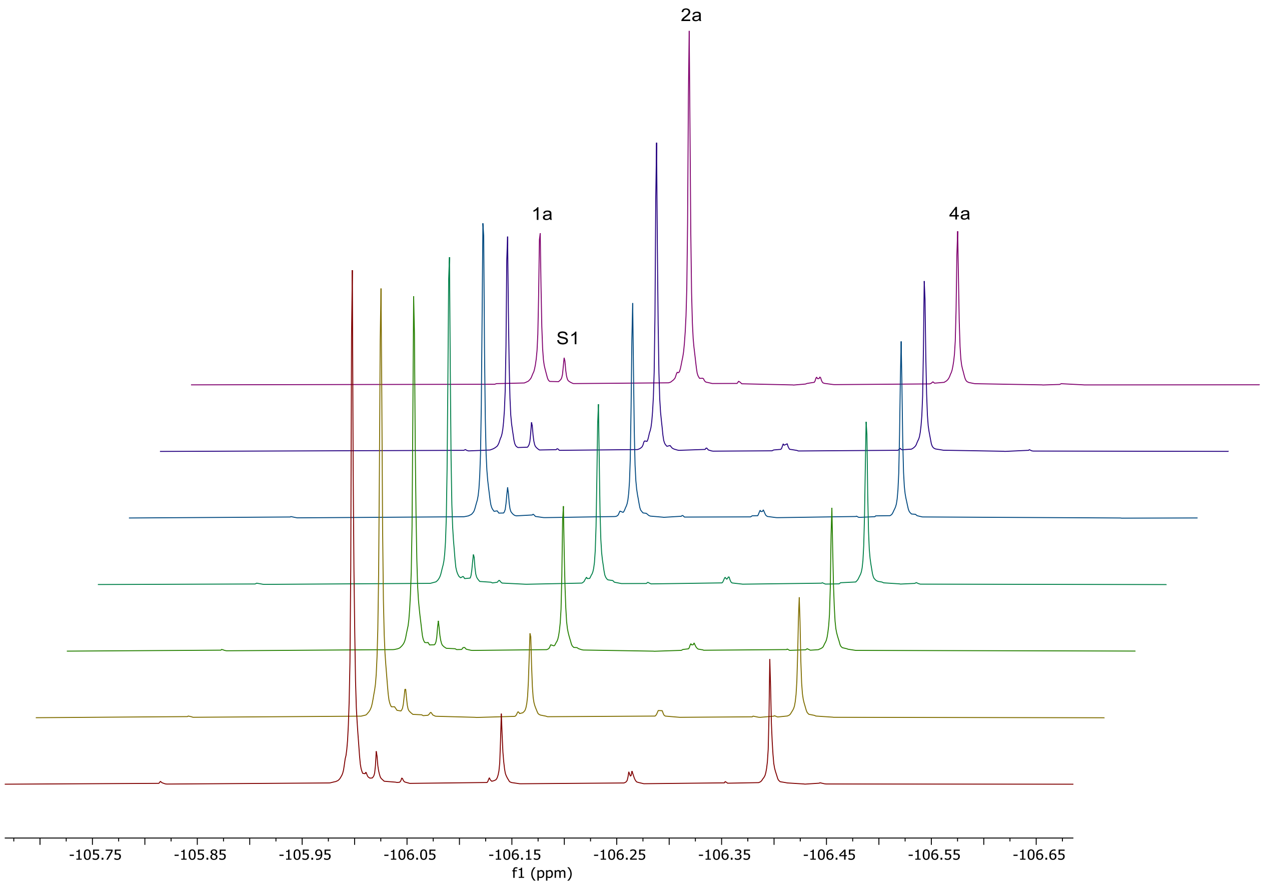

Figure S26: Stacked $^{19}F\{^{1}H\}$ NMR spectra in the aryl fluoride region.

In each of the experiments, a linear relation was observed between the concentration of product **2a** and reaction time up to >60% conversion. There was no detectable decomposition of catalyst (*S*)-**3d** by ^{19}F NMR. It was thus concluded that the reaction is zeroth order with respect to substrate **1a**. The pseudo-zeroth order rate constants k_{obs} were extracted by linearisation of the product concentration profile using least-square fitting in Microsoft Excel according to $[\mathbf{2a}]_t = k_{obs}\, t$.

Varying efficiency of mass transfer

The reaction was set up according to the general procedure using **1a** (81.9 mg, 0.3 mmol, 1 equiv.), (*S*)-**3d** (23.8 mg, 10 mol%), $[Rh(cod)Cl]_2$ (3.7 mg, 2.5 mol%), 4-fluoroanisole (15 µL) and KF (52.3 mg, 3 equiv.). The stirring speed was set at 600 rpm for 0 – 48 h and then increased to 1200 rpm for 48 – 96 h. Aliquots of 80 µL were taken at several time points for both stirring speeds.

k_{obs} approximately doubled as the stirring speed was doubled.

a)

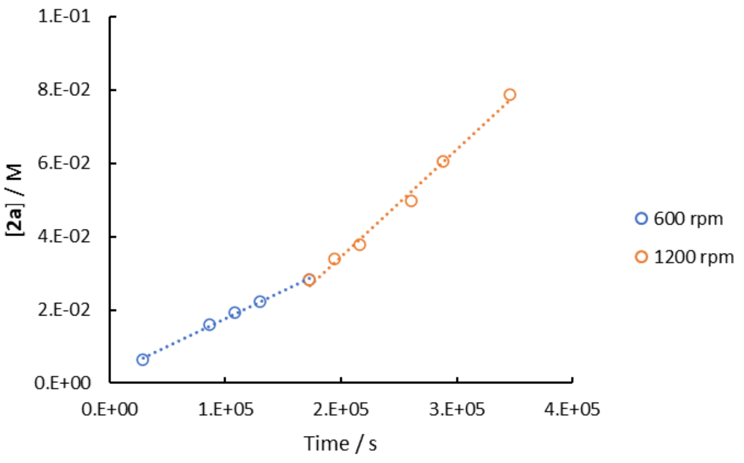

b) 600 rpm

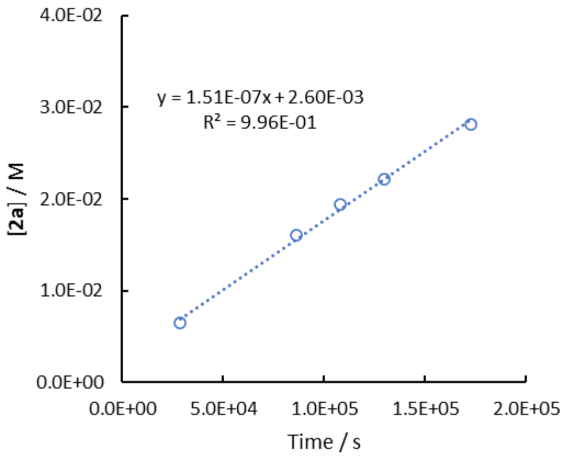

c) 1200 rpm

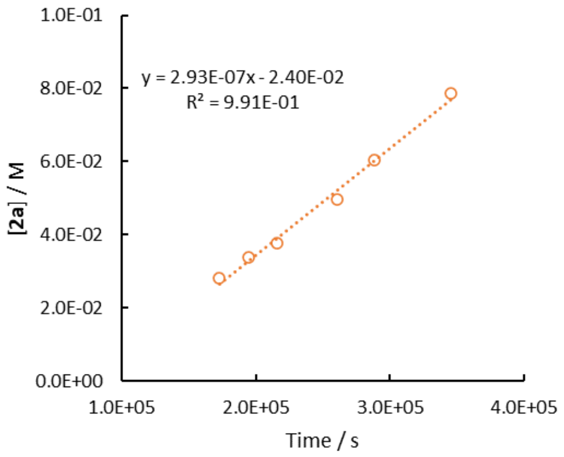

Figure S27: Product concentration profile under varying stirring speeds in a single reaction (a) and linear fitting for each stirring speed (b,c).

Determination of kinetic dependence on Rh catalyst

Each reaction was set up according to the general procedure using **1a** (81.9 mg, 0.3 mmol, 1 equiv.), (*S*)-**3d** (23.8 mg, 10 mol%), $[Rh(cod)Cl]_2$ (varying concentration, added as stock solution in DCM), 4-fluoroanisole (15 µL) and KF (104.6 mg, 6 equiv.) in DCM (1.5 mL total volume). An aliquot of 100 µL was taken every two hours between 1 h – 9 h. To ensure consistent rate of mass transfer, the same reaction vessel, the same magnetic stirrer and the same stirring speed (1200 rpm) were employed while fixing the position of the vial on the hot plate stirrer. Rate data from all experiments were summarised in a plot of k_{obs} against total Rh concentration $[Rh]_{tot}$.

a) $[Rh]_{tot}$ = 0.625 mM

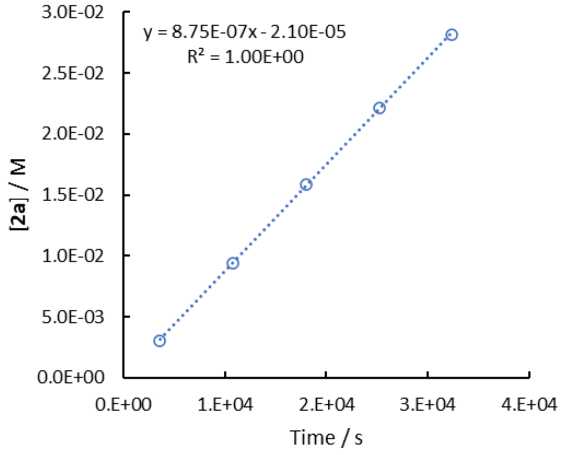

b) $[Rh]_{tot}$ = 1.25 mM

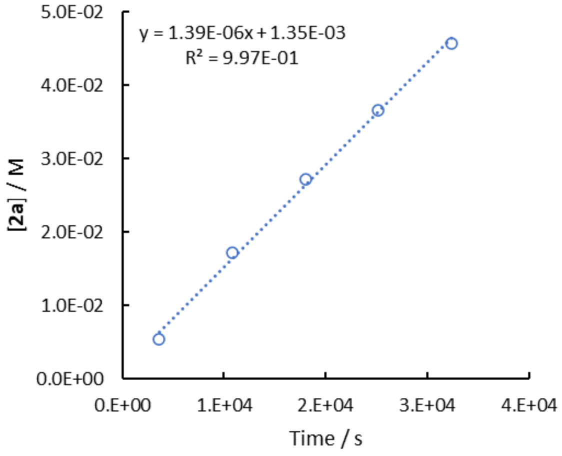

c) $[Rh]_{tot}$ = 1.875 mM

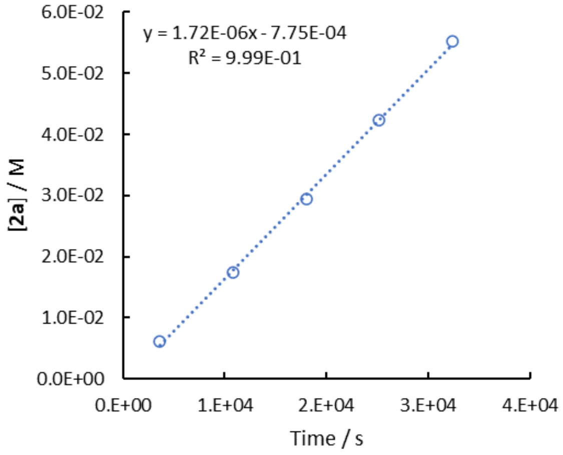

d) $[Rh]_{tot}$ = 2.5 mM

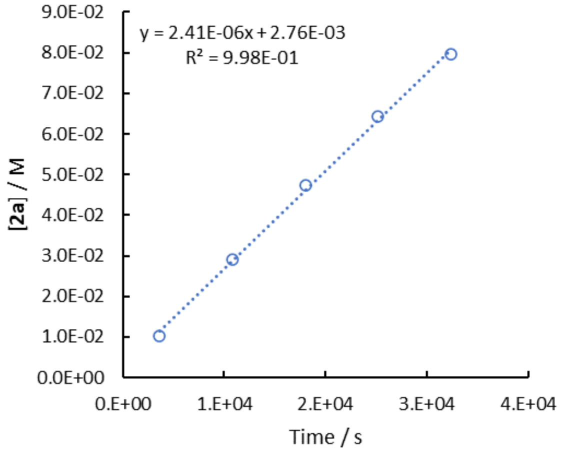

e) $[Rh]_{tot}$ = 5 mM

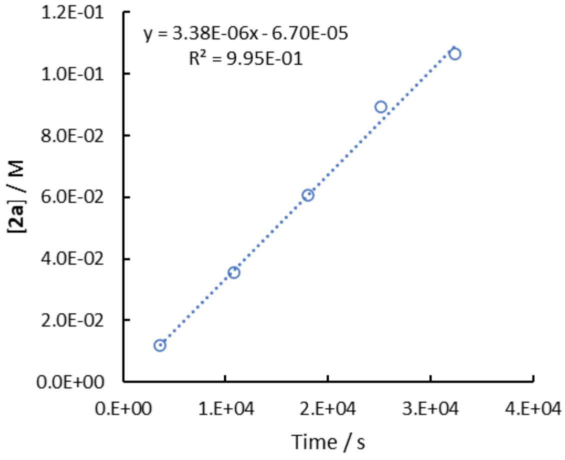

f) $[Rh]_{tot}$ = 7.5 mM

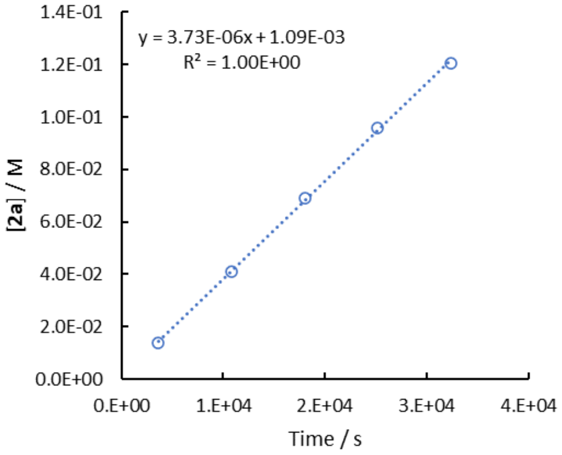

g) $[\mathrm{Rh}]_{tot}$ = 8.75 mM

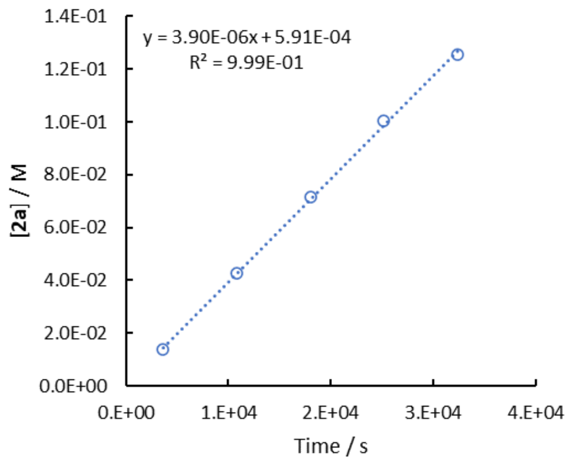

h) $[\mathrm{Rh}]_{tot}$ = 10 mM

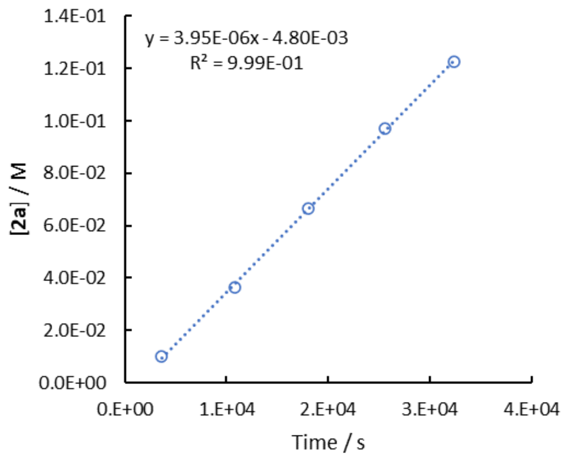

Figure S28: Product concentration profile under varying Rh concentrations ranging from 0.625 mM to 10 mM. [**1a**] = 200 mM, [**3d**] = 20 mM.

The rate dependence on rhodium can be fitted to the following equation describing saturation kinetics[21]:

$$\frac{d[\mathbf{2a}]}{dt} = \frac{a}{1 + \frac{b}{[\mathrm{Rh}]_{\mathrm{tot}}}}$$

$$a = 5.5 \times 10^{-6}\ \mathrm{M\ s^{-1}}, b = 3.4 \times 10^{-3}\ \mathrm{M}, R^2 = 0.9945$$

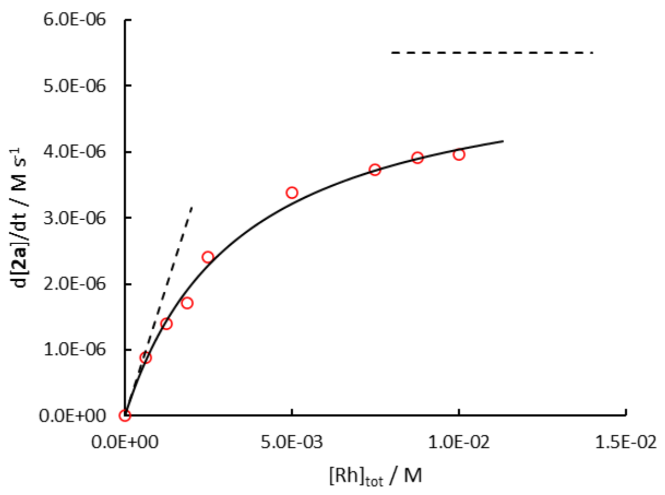

Figure S29: Plot of rate against total Rh concentration $[\mathrm{Rh}]_{tot}$. The black dotted lines show the initial gradient in linear regime and the limiting rate at saturation respectively.

Alternatively, the rate data can be fitted to the following equation describing half-order kinetics, though with a poorer fit than the model above:

$$\frac{d[\mathbf{2a}]}{dt} = a[\mathrm{Rh}]_{\mathrm{tot}}{}^{0.5}$$

$$a = 4.2 \times 10^{-5}\ \mathrm{M^{0.5}\ s^{-1}}, R^2 = 0.9786$$

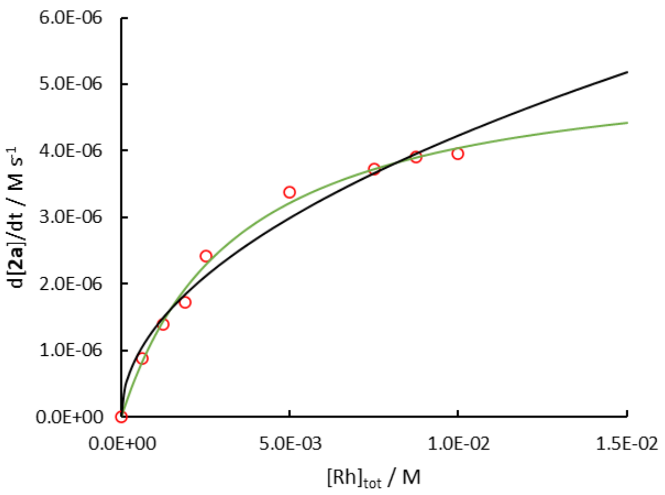

Figure S30: Plot of rate against total Rh concentration $[Rh]_{tot}$. The black line shows fitting to the half-order rate equation. The green line shows fitting to the saturation kinetics model above for comparison.

Determination of kinetic dependence on urea catalyst

Each reaction was set up according to the general procedure using **1a** (54.6 mg, 0.2 mmol, 1 equiv.), (*S*)-**3d** (varying concentration, added as stock solution in DCM), $[Rh(cod)Cl]_2$ (2.5 mg, 2.5 mol%), 4-fluoroanisole (10 µL) and KF (69.7 mg, 6 equiv.) in DCM (1.0 mL total volume). An aliquot of 100 µL was taken every two hours between 1 h – 9 h. To ensure consistent rate of mass transfer, the same reaction vessel, the same magnetic stirrer and the same stirring speed (1200 rpm) were employed while fixing the position of the vial on the hot plate stirrer. Rate data from all experiments were summarised in a plot of k_{obs} against urea concentration [**3d**].

a) [**3d**] = 0.625 mM

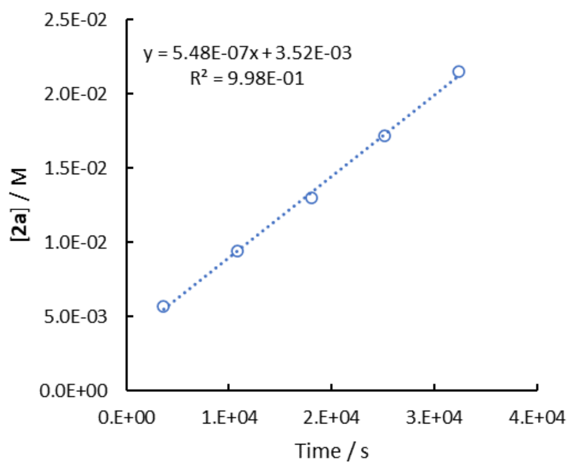

b) [**3d**] = 1.25 mM

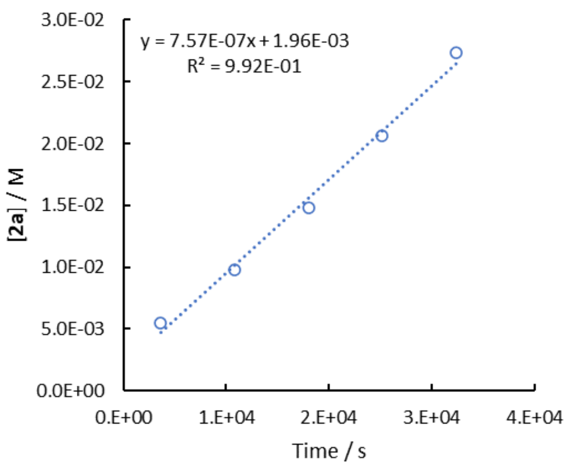

c) [**3d**] = 1.875 mM

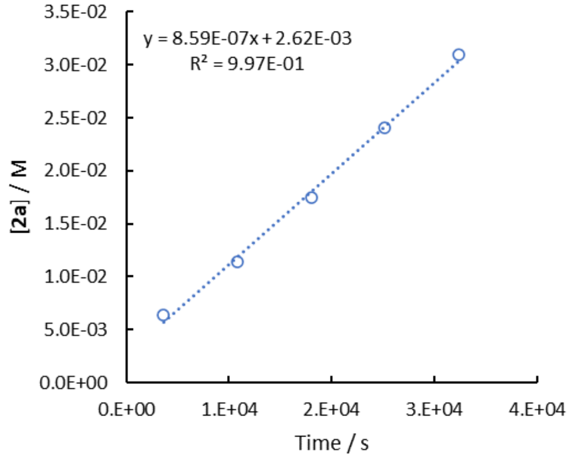

d) [**3d**] = 2.5 mM

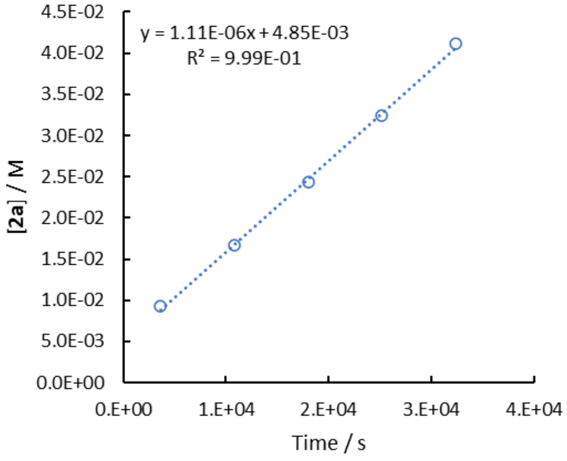

e) [**3d**] = 5 mM

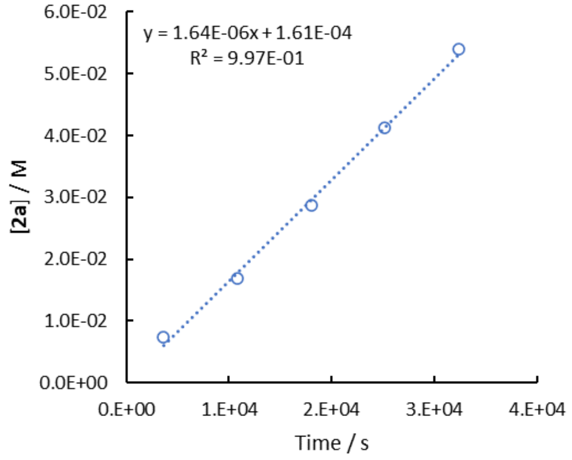

f) [**3d**] = 10 mM

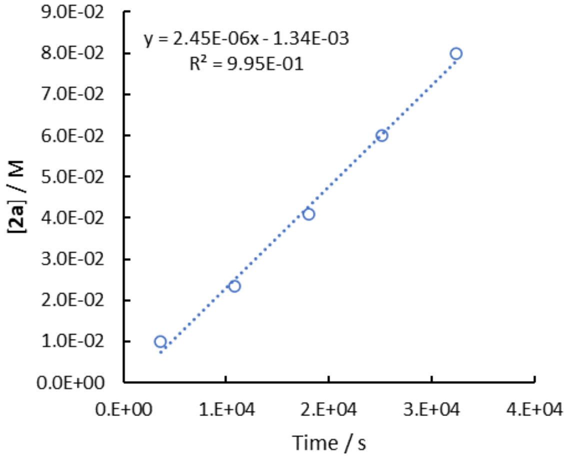

g) [**3d**] = 15 mM

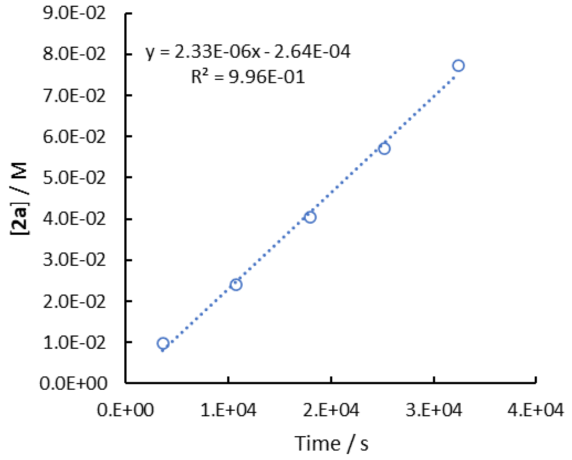

h) [**3d**] = 20 mM

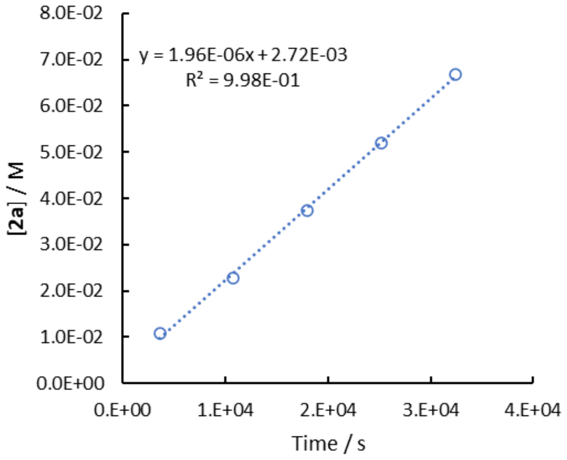

Figure S31: Product concentration profile under varying urea (*S*)-**3d** concentrations ranging from 0.625 mM to 20 mM. [**1a**] = 200 mM, $[Rh]_{tot}$ = 10 mM.

The rate dependence on urea **3d** can be fitted to the following equation describing competitive inhibition at high concentration[21]:

$$\frac{d[\mathbf{2a}]}{dt} = \frac{1}{\frac{a}{[\mathbf{3d}]} + b[\mathbf{3d}]}$$

$$a = 2.2 \times 10^3 \text{ s}, b = 1.9 \times 10^7 \text{ M}^{-2} \text{ s}, R^2 = 0.9709$$

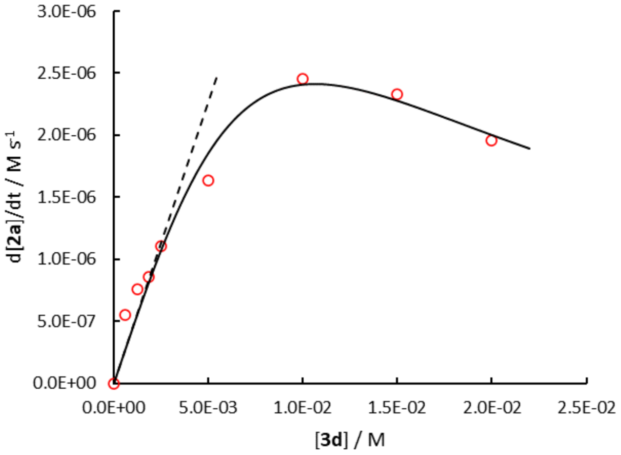

Figure S32: Plot of rate against urea concentration [**3d**]. The black dotted line shows the initial gradient in linear regime.

X-ray Crystallographic Data

Compound **2ab** was crystalized *via* vapor diffusion using a Et_2O/hexane solvent system. Several attempts to determine the absolute configuration for this compound were made, but the nature of the crystals made collecting good data very challenging. In each case, the indicative results gave the same result and the best determination is presented here. Solid-state data for **2ab** was collected using a Rigaku XtaLAB Synergy-R DW diffractometer with a HyPix-Arc 150 detector. Crystals were selected under perfluoropolyether oil, mounted on a MiTeGen Micromount loop and quench-cooled using an Oxford Cryosystems open flow N_2 cooling device[22]. Selected details of the data collection are given in Table S12. Data were reduced using the CrysAlisPro package[23], including unit cell parameter refinement and inter-frame scaling (which was carried out using SCALE3 ABSPACK within CrysAlisPro). Equivalent reflections were merged, and corrected. Structures were solved *ab initio* from the integrated intensities using SuperFlip[24] and refined using full-matrix least-squares on F2 using CRYSTALS[25]. Hydrogen atoms were included in the refinement with soft restraints[26].

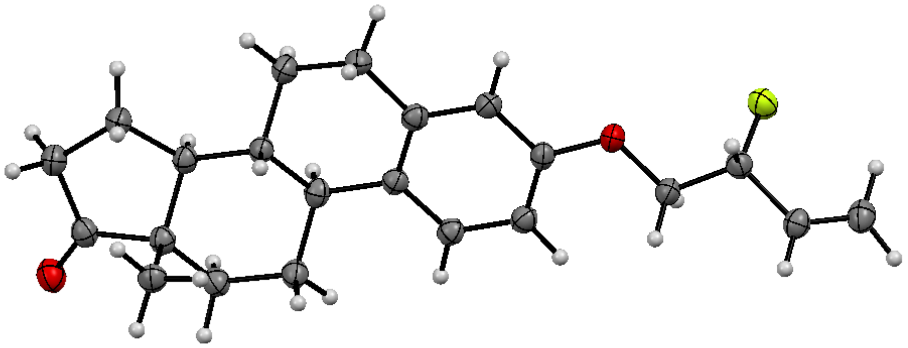

Figure S33: Crystal structure of **2ab** with 50% thermal ellipsoids.

Table S12: Selected X-ray data collection and refinement parameters for **2ab**.

**Name**	(8*R*,9*S*,13*S*,14*S*)-3-(((*R*)-2-fluorobut-3-en-1-yl)oxy)-13-methyl-6,7,8,9,11,12,13,14,15,16-decahydro-17*H*-cyclopenta[*a*]phenanthren-17-one (**2ab**)
**Formula**	$C_{22}H_{27}FO_2$
**Fw (g mol^{-1})**	342.45
**Cell system**	monoclinic
**Space group**	P 2_1
***a* (Å)**	6.73750(10)
***b* (Å)**	11.9937(2)
***c* (Å)**	11.2283(2)
***α* (°)**	90
***β* (°)**	91.8085(14)
***γ* (°)**	90
***V* (Å^{-3})**	906.88(3)
Z	2
**ρ_{calc} (g cm^{-3})**	1.254
**Radiation, λ (Å)**	CuKα (λ = 1.54184)
**Absorption**	Multi-scan
**µ (mm^{-1})**	0.684
**$R_{(int)}$**	0.0440
**Parameters**	227
**R_1 (all data/*I* >2σ(I))**	0.0440 / 0.0420
**Ωr_2 (all data/*I* >2σ(I))**	0.1122 / 0.1106
**GooF**	1.0090
***T* (K)**	100
**Largest diff. peak/hole (e Å^{-3})**	0.24 / -0.17
**Flack parameter**	-0.05(15)
**CCDC Deposition No.**	2514272

Computational Details

Computational methods

Geometry optimizations and vibrational frequency calculations were performed using *Gaussian 16, rev. C.01* at the M06-2X/def2-SVP(TZVPPD)-SDD(Rh) level of theory, where the double-zeta def2-SVP basis set was used for C, H, and O atoms and the triple-zeta def2-TZVPPD basis set was used for all other heteroatoms, including a Stuttgart/Dresden effective core potential (ECP) description, SDD, for Rh.[27–31] Solvation in dichloromethane was modeled using the conductor-like polarizable continuum model (CPCM).[32] Minima were identified by the absence of imaginary frequency vibrational modes; transition structures by the presence of a single imaginary mode. Single-point energy calculations were performed using the *ORCA 6.0.0* software package at the ωB97X-D3/(ma)-def2-TZVPP-def2-ECP(Rh) level of theory, where the triple-zeta def2-TZVPP basis set was used for C and H atoms and the ma-def-TZVPP basis set was used for all other heteroatoms, including the corresponding def2-ECP for Rh.[31b, 33–35] All bond lengths are reported in Angstrom (Å). Thermochemistry was evaluated at 298.15 K and a concentration of 0.25 mol·L^{-1} using Paton's *GoodVibes* script.[36] The entropic contribution of low vibrational modes was corrected using a frequency cutoff value of 100 cm^{-1}.[37] Non-covalent interaction (NCI) was evaluated using *NCIPLOT-4.2*.[38] Natural Bonding Orbital (NBO) analysis was performed using *NBO7.0* integrated in *ORCA*.[39]

Conformational sampling was performed prior to geometry optimization of key structures using the Global Optimizer Algorithm (GOAT) algorithm implemented in *ORCA 6.0.0*, using a GFN2-xTB Hamiltonian.[40,41] Conformational sampling was performed in dichloromethane using the extended conductor-like polarizable continuum model (CPCM-X).[42] A 25 kJ/mol energy window was applied during sampling.

Conformational analysis of [Rh(I)] complexes with substrate 2c

Two distinct low energy binding modes of Rh(I) complexes with model substrate **2c** were obtained, specifically via η^1-(σ-coordination) with the carbonyl oxygen atom in mode **i**, and η^2-(π-coordination) with the olefin in mode **ii** (Figure S34). The most stable conformers of each mode possess similar stability. An alternative binding mode involving both the carbonyl oxygen atom and the olefin lies significantly (23.4 kJ/mol) higher in energy than **i-a**.

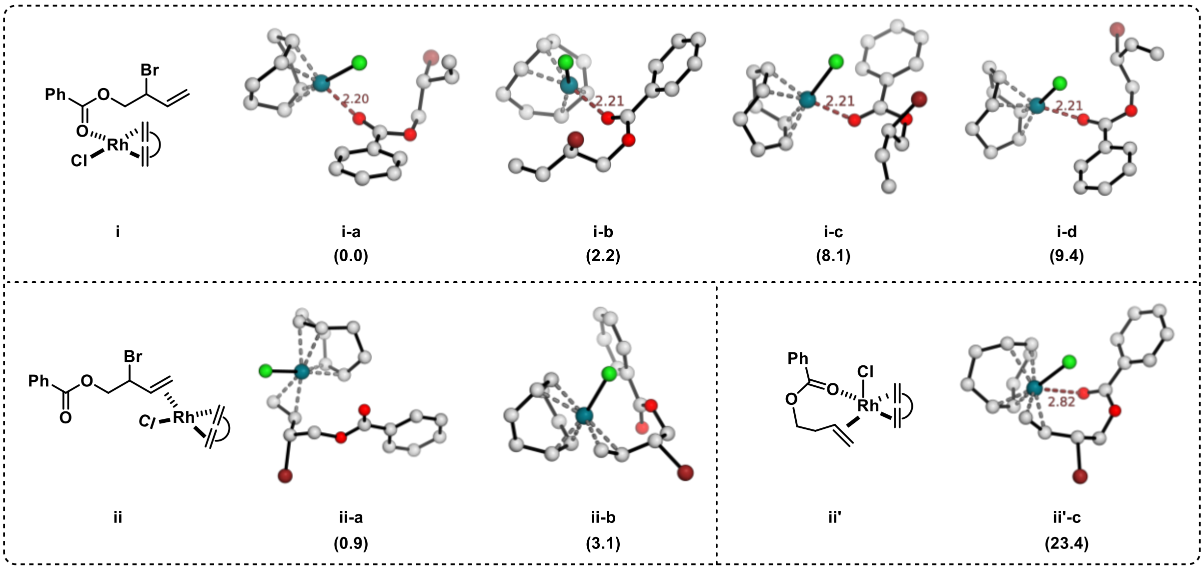

Figure S34: Relative Gibbs free energy (kJ/mol, labeled in brackets) of [Rh(I)] complexes with substrate **2c**.

Thermodynamic approach to quantify the phase-transfer step

To calculate the energy of the phase-transfer step, computed solution phase energies and literature experimental data were combined to construct the Hess cycle shown in Figure S35. This approach is in accordance with previously publish literature by Gouverneur.[1]

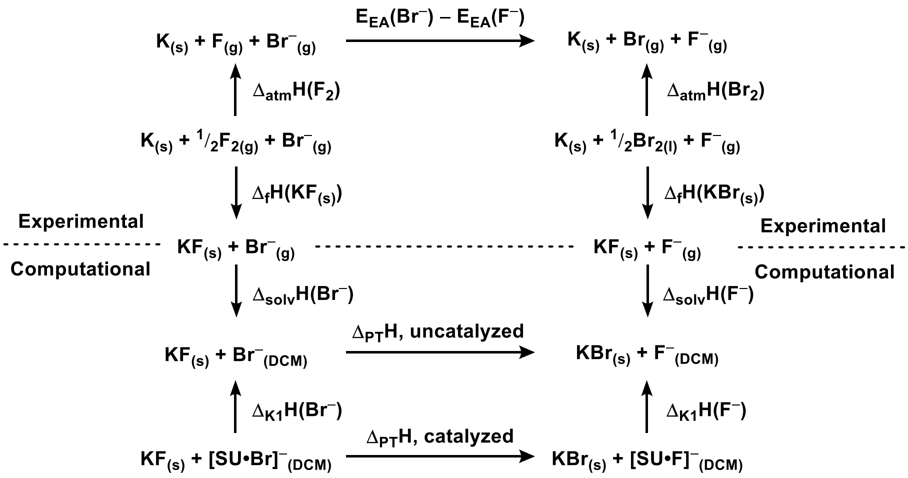

Figure S35: Hess cycle for calculation of the phase-transfer step.

	Source	ΔH (kJ/mol)	ΔS (J/K/mol)
$\Delta_f(KF_{(s)})$	Ref (43)	−567.3	–
$\Delta_f(KBr_{(s)})$	Ref (43)	−393.8	–
$\Delta_{atm}(F_2)$	Ref (43)	79.4	–
$\Delta_{atm}(Br_2)$	Ref (43)	111.9	–
$E_{EA}(F)$	Ref (43)	438.2	–
$E_{EA}(Br)$	Ref (43)	324.5	–
$\Delta_{solv}(F^-)$	Computed	−328.7	−26.6*
$\Delta_{solv}(Br^-)$	Computed	−250.6	−26.6*
$\Delta_{K1}(F^-)$	Computed	93.1	85.1
$\Delta_{K1}(Br^-)$	Computed	44.6	84.4

Table S13: Standard enthalpy and entropy changes at 298.15 K. *Entropy change associated with a change in standard state from 1 bar to 1 M.

	Source	S (J/K/mol)
$KF_{(s)}$	Ref (43)	66.6
$KBr_{(s)}$	Ref (43)	95.9
$F^-_{(g)}$	Ref (44)	145.6
$Br^-_{(g)}$	Ref (44)	163.6

Table S14: Standard molar entropies at 298.15 K.

	$\Delta_{PT}G$ (kJ/mol)	$\Delta_{PT}H$ (kJ/mol)	$\Delta_{PT}S$ (J/K/mol)
Uncatalyzed	**55.9**	59.3	11.3
SU catalyzed	**7.6**	10.8	10.7

Table S15: Thermochemical evaluation of the phase transfer step at 298.15 K.

Proposed mechanistic pathway

The proposed reaction pathway was computed, using Schreiner's urea (**SU**) as the catalyst and **1c** as model substrate (Fig. S36). The carbonyl oxygen atom of **1c** coordinates to the dissociated monomeric rhodium catalyst (ΔG = 31.1 kJ/mol), followed by isomerization to form the alkene coordinated complex (ΔG = 0.8 kJ/mol). Urea-assisted oxidative addition ($\Delta G^{\ddagger}$ = 73.3 kJ/mol via **ii-TS-a**) gives an ion pair (**iii-a**) between the Rh(III)-allyl complex and hydrogen-bonded bromide (ΔG = 29.1 kJ/mol). Dissociation of this ion pair in solution (ΔG = 0.6 kJ/mol) and halide exchange with KF in the solid phase (**IV**, ΔG = 7.6 kJ/mol) are both thermodynamically facile, leading to the reactive ion pair (**v-a**) with urea-bound fluoride (ΔG = −17.3/mol). Outer-sphere attack at the branched position via **v-TS-a** ($\Delta G^{\ddagger}$ = 41.1 kJ/mol) yields **vi** irreversibly (ΔG = −96.6 kJ/mol). Branched fluoride product **2c** is released from this complex with regeneration of the Rh(I) catalyst (ΔG = −47.5 kJ/mol). In contrast, the computed pathway in the absence of urea (red line, Fig. S30) shows thermodynamically unfavorable formation of the fluoride ion-pair from KF, also contributing to a prohibitive barrier for nucleophilic attack.

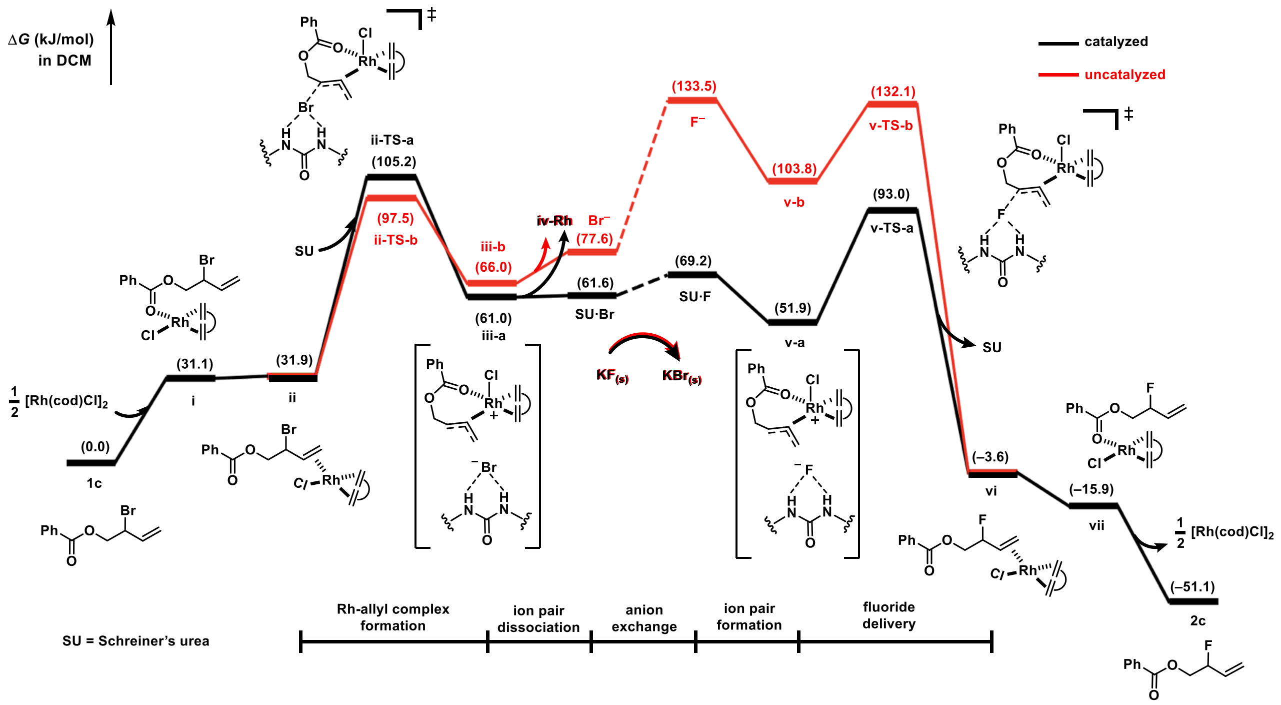

Figure S36: Computed Gibbs free energy profile (kJ/mol) of rhodium catalyzed allylic fluorination where Schreiner's urea (**SU**) is employed as the catalyst.

The overall energy barrier starting with Rh-alkene complex **ii** to linear bromide **viii** under urea catalyzed or uncatalyzed pathway differs by only 0.5 kJ/mol ($\Delta G^{\ddagger}_{cat}$ = 78 kJ/mol and $\Delta G^{\ddagger}_{uncat}$ = 77.5 kJ/mol, Figure S37). Pathways from **ii** to **iii-a** and from **ii** to **iii-b** are both kinetically feasible. It is therefore reasonable to expect both the catalyzed and uncatalyzed pathways to operate under experimental conditions.

For both branched (**1c**) and linear bromide (**4c**), the most stable binding mode with Rh(I) complex is via η^1-(σ-coordination) at the carbonyl oxygen atom. The Rh(I) complex with linear bromide **(ix)** is lower in energy than the corresponding complex **i** with the branched bromide (ΔΔG = −6.3 kJ/mol), due to the inherent stability

of internal alkene. To undergo bromide abstraction, a Rh-η^2-alkene complex is likely involved. This complex with linear bromide (**viii**) is found to be less accessible than the corresponding branched bromide (**ii**, ΔΔG = 4.1 kJ/mol). This is likely due to the increased steric bulk of the internal alkene, justifying the observed reduced reactivity of **4a**.

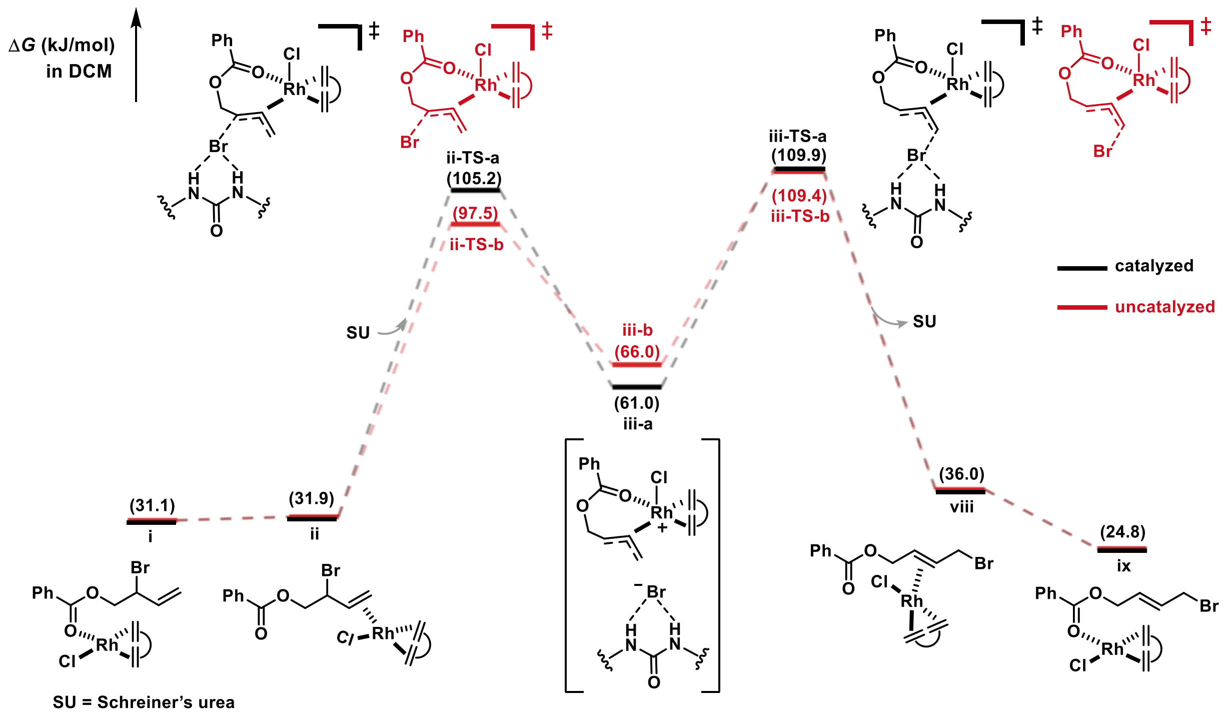

Figure S37: Computed Gibbs free energy profile (kJ/mol) of rhodium catalyzed isomerization from branched to linear allyl bromide.

Origin of enantioselectivity

The computed major and minor transition structures (TSs) of the enantio-determining step between the chiral urea bound fluoride and the Rh-allyl complex were located, giving a predicted selectivity of 94:6 e.r. at 243 K ($\Delta\Delta G^{\ddagger}$ = 5.7 kJ/mol), favoring (*R*)-product formation (Figure S38). Non-covalent interaction (NCI) plots of the major and minor TSs are presented in Figure S39. Purple/blue isosurface values, corresponding to large negative values of sign(λ_2)ρ, represent strong attractive interactions. Green isosurface values, corresponding to values of sign(λ_2)ρ close to 0, represent attractive, dispersion-dominated interactions. Orange/red isosurface values, corresponding to large positive values of sign(λ_2)ρ, represent repulsive steric interactions. The NCI plot reveals dispersion-dominated interactions within urea and between urea and the Rh-allyl complex.

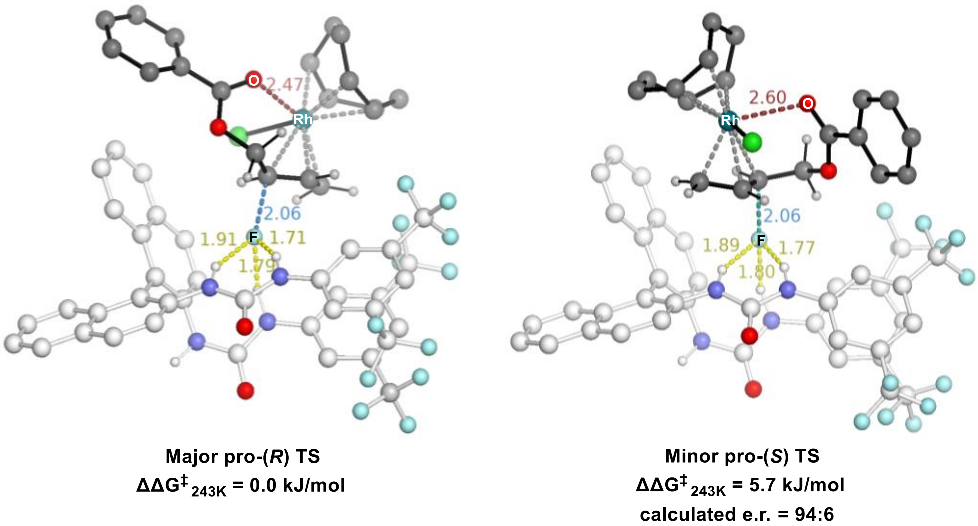

Figure S38: Major and minor transition state structures of the enantio-determining step.

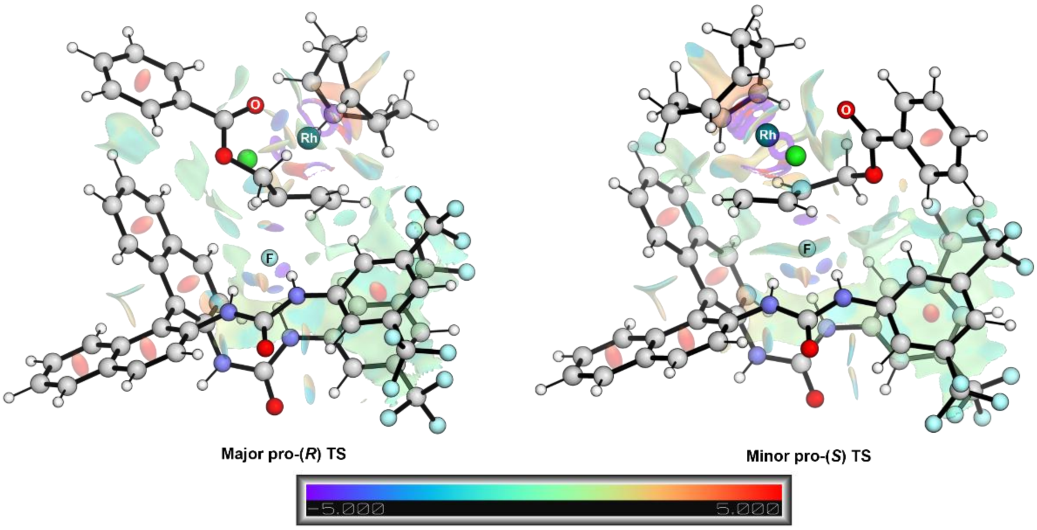

Figure S39: NCI plot of the major and minor transition state structures.

The major contribution to the difference in TS stabilities is from the single-point energy ($\Delta\Delta E^{\ddagger}$ = 3.2 kJ/mol, Table S16). Subsequent Distortion-Interaction/Activation-Strain (DIAS) analysis identified the difference in distortion energies of the electrophilic Rh-allyl species as being the major difference between the major and minor transition states ($\Delta\Delta E_{dist}$([Rh–allyl]$^{+}$) = −11.6 kJ/mol, Table S17). Further dissecting the Rh–allyl components into two fragments, [Rh]$^{2+}$ and [allyl]$^{-}$, revealed little difference in their distortion terms. However, a more favorable interaction between [Rh]$^{2+}$ and [allyl]$^{-}$ preferentially stabilizes the electrophile in the major TS ($\Delta\Delta E_{int}$ = −14.2 kJ/mol, Table S18). Second-order perturbative estimates of donor-acceptor interactions in each TS in the NBO basis identified stronger coordination of carbonyl lone pairs and Rh ($\Delta\Delta E$ = 31.8 kJ/mol) in the major TS, as well as a greater donor-acceptor interaction between carbonyl lone pair and antibonding $\sigma^{*}_{C\text{-}Rh}$ orbital between Rh and the terminal allylic carbon ($\Delta\Delta E$ = 15.8 kJ/mol, Table S19).

	ΔΔE‡	ΔΔH‡	T·ΔΔS‡	ΔΔG‡
Major − Minor	−3.2 kJ/mol	−4.0 kJ/mol	1.6 kJ/mol	−5.7 kJ/mol

Table S16: Energy difference between the major and minor transition states at 243 K. Discrepancy between ΔΔG‡ and sum of ΔΔH‡ and T·ΔΔS‡ is due to propagation of rounding error.

	ΔΔE‡	$\Delta\Delta E_{dist}$([Urea·F]⁻)	$\Delta\Delta E_{dist}$([Rh–allyl]⁺)	$\Delta\Delta E_{int}$
Major − Minor	−3.2 kJ/mol	−0.1 kJ/mol	−11.6 kJ/mol	8.6 kJ/mol

Table S17: Distortion/interaction analysis of the transition state structures. Discrepancy between ΔΔE‡ and sum of ΔΔE‡ of each component and $\Delta\Delta E^{\ddagger}_{int}$ is due to propagation of rounding error.

	ΔΔE‡([Rh–allyl]⁺)	$\Delta\Delta E_{dist}$ ([Rh]$^{2+}$)	$\Delta\Delta E_{dist}$([allyl]⁻)	$\Delta\Delta E_{int}$
Major − Minor	−11.6 kJ/mol	2.4 kJ/mol	0.1 kJ/mol	−14.2 kJ/mol

Table S18: Distortion/interaction analysis of the Rh–allyl components at the transition state. Discrepancy between ΔΔE‡([Rh–allyl]⁺) and sum of ΔΔE‡ of each fragment and $\Delta\Delta E^{\ddagger}_{int}$ is due to propagation of rounding error.

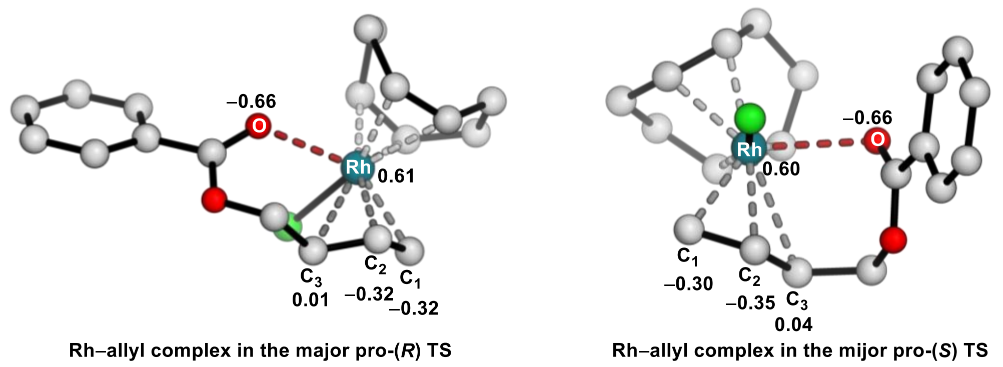

Figure S40: Truncated Rh–allyl complexes in the corresponding transition state structures. Natural charges are labeled next to the atom of interest.

	WBIs	NBO (Rh–C_1)	2nd order perturbation (kJ/mol)
Major	Rh–O: 0.08	Rh (66%)	LP_1 (O) to LV_2 (Rh): 39.3
	Rh–C_1: 0.62	C_1 (34%)	LP_2 (O) to LV_1 (Rh): 5.6
	Rh–C_2: 0.17		LP_2 (O) to LV_2 (Rh): 22.5
	Rh–C_3: 0.25		LP_1 (O) to BD* (Rh–C_1): 30.5
Minor	Rh–O: 0.06	Rh (67%)	LP_1 (O) to LV_2 (Rh): 29.7
	Rh–C_1: 0.63	C_1 (33%)	LP_2 (O) to LV_1 (Rh): 5.9
	Rh–C_2: 0.17		LP_1 (O) to BD* (Rh–C_1): 14.7
	Rh–C_3: 0.22		

Table S19: NBO analysis of the transition state structures. WBI = Wiberg bond index.

Despite the lack of a strong coordinating oxygen substituent, **2j** showed good enantioselectivity toward fluorination. Thus, the major and minor transition structures of the enantio-determining step with substrate **2j** were further investigated. The computed selectivity is 88:12 e.r. at 298 K ($\Delta\Delta G^{\ddagger}$ = 4.9 kJ/mol), favoring the formation of (*R*)-product (Figure S41).

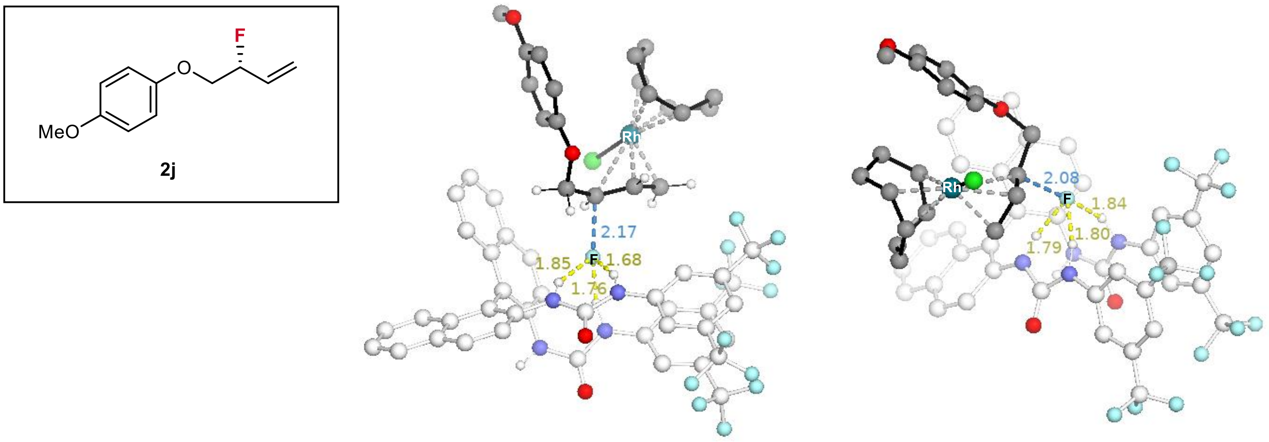

Major pro-(*R*) TS-2j
$\Delta\Delta G^{\ddagger}_{298K}$ = 0.0 kJ/mol

Minor pro-(*S*) TS-2j
$\Delta\Delta G^{\ddagger}_{298K}$ = 4.9 kJ/mol
calculated e.r. = 88:12

Figure S41: Major and minor transition state structures of the enantio-determining step.

The major contribution to the difference in TS stabilities is from the single-point energy ($\Delta\Delta E^{\ddagger}$ = 3.3 kJ/mol, Table S20). A similar DIAS approach was applied, indicating the difference in distortion energies of the electrophilic Rh-allyl species as being the major difference between the major and minor transition states ($\Delta\Delta E_{dist}$([Rh–allyl]$^{+}$) = –11.3 kJ/mol, Table S21). Further dissecting the Rh–allyl components into two fragments, [Rh]$^{2+}$ and [allyl]$^{-}$, revealed the primary contribution to the energy difference originates from the distortion term of the [allyl]$^{-}$ component (($\Delta\Delta E_{dist}$([allyl]$^{-}$) = −13.9 kJ/mol, Table S22).

	$\Delta\Delta E^{\ddagger}$	$\Delta\Delta H^{\ddagger}$	$T\cdot\Delta\Delta S^{\ddagger}$	$\Delta\Delta G^{\ddagger}$
Major − Minor	−3.3 kJ/mol	−4.4 kJ/mol	0.5 kJ/mol	−4.9 kJ/mol

Table S20: Energy difference between the major and minor transition states with substrate **2j** at 298 K. Discrepancy between $\Delta\Delta G^{\ddagger}$ and sum of $\Delta\Delta H^{\ddagger}$ and $T\cdot\Delta\Delta S^{\ddagger}$ is due to propagation of rounding error.

	$\Delta\Delta E^{\ddagger}$	$\Delta\Delta E_{dist}$([Urea·F]$^{-}$)	$\Delta\Delta E_{dist}$([Rh–allyl]$^{+}$)	$\Delta\Delta E_{int}$
Major − Minor	−3.2 kJ/mol	−3.4 kJ/mol	−11.3 kJ/mol	11.5 kJ/mol

Table S21: Distortion/interaction analysis of the transition state structures with substrate **2j**. Discrepancy between $\Delta\Delta E^{\ddagger}$ and sum of $\Delta\Delta E^{\ddagger}$ of each component and $\Delta\Delta E^{\ddagger}_{int}$ is due to propagation of rounding error.

	ΔΔE‡([Rh–allyl]+)	ΔΔE$_{dist}$ ([Rh]$^{2+}$)	ΔΔE$_{dist}$([allyl]$^{-}$)	ΔΔE$_{int}$
Major − Minor	−11.3 kJ/mol	3.7 kJ/mol	−13.9 kJ/mol	−1.1 kJ/mol

Table S22: Distortion/interaction analysis of the Rh–allyl components at the transition state. Discrepancy between ΔΔE‡([Rh–allyl]+) and sum of ΔΔE‡ of each fragment and ΔΔE‡$_{int}$ is due to propagation of rounding error.

In both cases, whether or not the substrate bears a strong coordinating group, these results demonstrated that by interacting with the (*S*)-BINAM catalyst, the Rh-allyl complex in the pro-(*R*) transition state structure always adopt a more stable conformation than in the pro-(*S*) structure.

XYZ coordinates

i–a

C 3.158367 -0.683576 1.260226
C 2.465650 -1.901204 1.332326
C 2.853864 -3.143557 0.543583
H 2.641033 -4.033441 1.150546
C 2.088426 -3.240979 -0.785876
C 1.769396 -1.884780 -1.378315
C 2.667229 -0.817268 -1.496228
C 4.128574 -0.891613 -1.081770
H 4.729009 -0.280544 -1.768745
C 4.337975 -0.392442 0.357206
H 4.475321 0.698866 0.343019
H 5.261447 -0.814508 0.790361
H 4.489085 -1.923090 -1.198071
H 2.407875 -0.028918 -2.210572
Rh 1.369399 -0.508711 0.175365
Cl 0.186617 0.445667 2.090942
O -0.208249 0.396038 -1.066980
C -0.865091 1.423166 -0.966505
C -0.305613 2.757342 -0.649477
C 1.082051 2.940513 -0.688191
C 1.623026 4.183653 -0.375659
C 0.781399 5.239797 -0.018422
C -0.602080 5.057457 0.019124
C -1.150302 3.817983 -0.301151
H -2.228468 3.661921 -0.271276
H -1.255710 5.883677 0.300165
H 1.207768 6.212304 0.232012
H 2.703002 4.330224 -0.408530
H 1.732025 2.107923 -0.965072
O -2.171279 1.420831 -1.158625
C -2.811803 0.151755 -1.327744
H -2.209603 -0.482141 -1.988584
H -3.779817 0.368450 -1.794078
C -2.996335 -0.495020 0.041403
H -2.023462 -0.656391 0.525072
Br -3.716291 -2.297175 -0.287844
C -3.921060 0.268758 0.929417
C -3.567737 0.703167 2.139506
H -4.262927 1.277731 2.754412
H -2.568935 0.502441 2.538125
H -4.917249 0.476798 0.523570
H 0.875570 -1.854083 -2.011219
H 1.131990 -3.755550 -0.614290
H 2.644225 -3.855552 -1.515044
H 3.939704 -3.143276 0.374356
H 1.856437 -2.067912 2.225671
H 3.047019 -0.001967 2.109395

i–b

C -2.801064 -1.272878 1.277025
C -3.198587 0.067962 1.395765
C -4.408333 0.655067 0.682434
H -4.872324 1.411756 1.328744
C -4.028640 1.299051 -0.661235
C -2.857154 0.610619 -1.329281
C -2.702568 -0.773064 -1.473667
C -3.743174 -1.788258 -1.027440
H -3.745904 -2.632306 -1.729841
C -3.462775 -2.307793 0.392204
H -2.784821 -3.171500 0.333350
H -4.389225 -2.675755 0.866414
H -4.742799 -1.336205 -1.090733
H -1.979901 -1.117994 -2.220606
Rh -1.502110 -0.071471 0.146439
Cl 0.115582 0.016634 1.983343
O 0.176647 0.422232 -1.195861
C 1.251277 0.991457 -1.061717

C 1.410742 2.378772 -0.570697
C 0.264658 3.152823 -0.350072
C 0.392196 4.456029 0.119774
C 1.660583 4.984371 0.372205
C 2.803086 4.213081 0.151857
C 2.682513 2.909468 -0.322634
H 3.566940 2.296798 -0.496242
H 3.791120 4.628731 0.351409
H 1.758514 6.005538 0.743619
H -0.497909 5.061928 0.291162
H -0.721604 2.728804 -0.547546
O 2.385942 0.392875 -1.374588
C 2.317860 -0.990057 -1.726651
H 3.261766 -1.217675 -2.233823
H 1.476630 -1.150781 -2.414661
C 2.116111 -1.855700 -0.494132
H 1.303453 -1.455221 0.125438
Br 3.706869 -1.705721 0.663723
C 1.860629 -3.285450 -0.835155
C 0.755529 -3.916721 -0.436893
H 0.563672 -4.955542 -0.710692
H 0.009951 -3.404851 0.180278
H 2.612000 -3.793698 -1.448451
H -2.259317 1.246846 -1.991288
H -3.738403 2.345905 -0.489381
H -4.896817 1.326868 -1.342193
H -5.163861 -0.130685 0.542514
H -2.851645 0.608425 2.281675
H -2.187928 -1.679245 2.087444

i–c

C -2.924399 -1.391290 1.365475
C -3.196899 -0.016033 1.408085
C -4.290824 0.691057 0.636853
H -4.073133 1.768538 0.677814
C -4.369088 0.249357 -0.833785
C -2.999058 -0.111196 -1.386757
C -2.480663 -1.411534 -1.397389
C -3.166292 -2.635817 -0.828818
H -2.413530 -3.435150 -0.771062
C -3.751091 -2.392547 0.571834
H -3.785664 -3.342964 1.120538
H -4.789836 -2.039215 0.509269
H -3.945898 -2.994061 -1.523179
H -1.678850 -1.620035 -2.114388
Rh -1.539156 -0.337381 0.160081
Cl 0.038546 0.055117 1.989009
O 0.140300 0.138070 -1.187641
C 1.053459 0.946728 -1.087180
C 0.887410 2.368795 -0.706799
C -0.403425 2.905722 -0.624607
C -0.575920 4.236538 -0.257305
C 0.537324 5.029303 0.031895
C 1.824134 4.494843 -0.051247
C 2.003826 3.165196 -0.424345
H 3.003673 2.736387 -0.489911
H 2.690778 5.116025 0.176440
H 0.400231 6.071534 0.323862
H -1.579947 4.657276 -0.195291
H -1.268321 2.278393 -0.848874
O 2.302537 0.602261 -1.336416
C 2.579481 -0.779443 -1.564463
H 3.564197 -0.805820 -2.043703
H 1.822229 -1.196372 -2.242442
C 2.568726 -1.558600 -0.263457
H 1.641037 -1.361450 0.292406
Br 3.951595 -0.828913 0.944700
C 2.789099 -3.029335 -0.413566
C 3.065787 -3.656720 -1.557885
H 3.196919 -4.739702 -1.575585
H 3.167934 -3.133559 -2.511289
H 2.700452 -3.601994 0.513959
H -2.562407 0.599905 -2.095760
H -4.796928 1.062864 -1.434621
H -5.047933 -0.606480 -0.953110
H -5.262421 0.552433 1.142170
H -2.804332 0.537618 2.266952
H -2.329552 -1.799834 2.187740

i–d

C 3.106989 -0.896485 1.262838
C 2.200392 -1.959593 1.392717
C 2.270900 -3.272736 0.643246
H 1.298945 -3.771974 0.767429
C 2.555146 -3.089796 -0.856308
C 1.952303 -1.800240 -1.390837
C 2.656961 -0.594552 -1.485097
C 4.079635 -0.367135 -1.019124
H 4.246495 0.719904 -0.996627
C 4.346090 -0.945490 0.380247

H 5.150662 -0.373773 0.861334
H 4.705550 -1.981979 0.315839
H 4.794602 -0.774145 -1.754903
H 2.278740 0.142491 -2.202437
Rh 1.345003 -0.479395 0.172926
Cl 0.136563 0.484338 2.071269
O -0.186350 0.476137 -1.103603
C -0.864425 1.474112 -0.894519
C -0.315731 2.811912 -0.574391
C -1.158578 3.850373 -0.161432
C -0.614456 5.091960 0.157671
C 0.762636 5.297643 0.056967
C 1.602110 4.263038 -0.363003
C 1.065203 3.018472 -0.676505
H 1.711953 2.200909 -1.000810
H 2.676932 4.427675 -0.443680
H 1.185719 6.271554 0.307567
H -1.266027 5.901483 0.487809
H -2.231512 3.676249 -0.082168
O -2.180449 1.436492 -0.963132
C -2.803871 0.165149 -1.179091
H -2.275168 -0.368684 -1.977205
H -3.826988 0.390504 -1.500369
C -2.794061 -0.654545 0.103132
H -1.763530 -0.877134 0.407697
Br -3.531941 -2.423227 -0.358341
C -3.558780 -0.088979 1.256954
C -4.297855 1.020804 1.224990
H -4.815868 1.365070 2.121451
H -4.403851 1.630718 0.325035
H -3.467187 -0.656227 2.187796
H 1.063409 -1.907230 -2.021131
H 2.136595 -3.939287 -1.412142
H 3.635528 -3.097122 -1.056762
H 3.020633 -3.936234 1.108199
H 1.587365 -1.976637 2.299133
H 3.118989 -0.158668 2.070757

ii−a

C -3.421164 1.664643 0.227775
C -2.430361 2.055015 1.085869
C -1.438551 3.162534 0.808387
H -1.217072 3.676993 1.752895
C -0.119156 2.639840 0.217180
C -0.272182 1.522759 -0.791197
C -1.286887 1.396453 -1.741087
C -2.381789 2.425690 -1.988854
H -2.648028 2.398174 -3.053589
C -3.660746 2.191637 -1.165977
H -4.292799 1.458536 -1.686423
H -4.252658 3.121581 -1.112150
H -1.976954 3.430184 -1.807475
H -1.076046 0.734498 -2.585847
Rh -1.709401 0.118190 -0.038742
Cl -2.808335 -1.292905 1.612168
O 1.762559 0.545637 2.420105
C 2.177972 0.222916 1.334864
C 3.133912 1.032567 0.525316
C 3.574346 0.611911 -0.735280
C 4.452174 1.414101 -1.461323
C 4.889060 2.629655 -0.931598
C 4.451546 3.047360 0.327299
C 3.575181 2.249074 1.057080
H 3.219562 2.554485 2.042130
H 4.795879 3.996620 0.739203
H 5.576028 3.255481 -1.503288
H 4.797593 1.089753 -2.443524
H 3.228533 -0.339835 -1.139964
O 1.798621 -0.896287 0.709163
C 0.805389 -1.698869 1.335903
H 0.108314 -1.058711 1.896690
H 1.280997 -2.401787 2.034043
C 0.029089 -2.424336 0.259121
H -0.801048 -2.966699 0.721777
Br 1.168718 -3.828614 -0.546029
C -0.449655 -1.538983 -0.855508
C -1.714119 -1.625547 -1.398925
H -1.927988 -1.232637 -2.393107
H -2.449425 -2.311430 -0.972292
H 0.348252 -1.055460 -1.426412
H 0.654053 0.967408 -0.972321
H 0.509823 2.264672 1.037896
H 0.449681 3.466106 -0.244818
H -1.897523 3.911518 0.151129
H -2.458359 1.648986 2.102104
H -4.184989 0.988582 0.622487

ii−b

C -1.614018 2.533569 -0.938333
C -2.219807 1.351957 -0.556513

C -2.956148 1.122782 0.752101
H -3.713385 0.344023 0.580494
C -2.060031 0.677585 1.929268
C -0.604370 1.071414 1.806835
C -0.177831 2.367534 1.529788
C -1.143889 3.527422 1.353739
H -0.700078 4.432891 1.788180
C -1.484641 3.792317 -0.118064
H -0.689585 4.401244 -0.571869
H -2.412573 4.384571 -0.200452
H -2.056145 3.324942 1.932446
H 0.845645 2.634939 1.806426
Rh -0.008168 1.136407 -0.260605
Cl 0.281568 0.454590 -2.583602
O -0.321576 -2.341424 2.036471
C -0.505746 -2.204212 0.852778
C -1.840276 -2.312388 0.194412
C -2.931627 -2.696629 0.983252
C -4.197974 -2.792931 0.415356
C -4.375130 -2.507326 -0.941749
C -3.285810 -2.133391 -1.729489
C -2.013523 -2.037920 -1.165746
H -1.157904 -1.724006 -1.765316
H -3.426657 -1.910867 -2.788186
H -5.368696 -2.580242 -1.386815
H -5.049593 -3.091159 1.027830
H -2.770065 -2.912607 2.040559
O 0.472127 -1.927538 -0.021580
C 1.822322 -1.917412 0.429865
H 2.292471 -2.855721 0.107679
H 1.857102 -1.852877 1.525936
C 2.522508 -0.726883 -0.206123
H 2.410665 -0.738643 -1.295830
Br 4.454667 -0.998730 0.116360
C 2.136666 0.600847 0.367931
C 2.166051 1.766361 -0.356955
H 2.194947 2.739562 0.133519
H 2.396832 1.742814 -1.423973
H 2.131998 0.647987 1.462849
H 0.096202 0.384859 2.294675
H -2.105274 -0.412278 2.034371
H -2.453232 1.086055 2.876566
H -3.507075 2.038005 1.014641
H -2.401626 0.623844 -1.351528
H -1.371188 2.637359 -1.999560

ii'−c

C -0.379021 3.087421 -1.107002
C -1.251836 2.490338 -0.241686
C -1.411222 2.854770 1.215828
H -2.018767 2.075259 1.687516
C -0.088900 2.986875 2.000168
C 1.067212 2.129702 1.504743
C 1.967964 2.519634 0.518766
C 1.929511 3.783272 -0.309993
H 2.523511 3.594439 -1.217251
C 0.521683 4.239368 -0.721424
H 0.609034 4.923646 -1.576172
H 0.047923 4.812565 0.086620
H 2.449495 4.592565 0.231425
H 2.955489 2.050490 0.555435
Rh 0.598317 1.067622 -0.330901
Cl -0.602578 -0.225278 -2.018105
O -1.086072 -0.194720 1.541946
C -1.476277 -1.186443 0.967840
C -2.863458 -1.365109 0.454308
C -3.255702 -2.532906 -0.209700
C -4.559061 -2.651357 -0.686549
C -5.470152 -1.609458 -0.500960
C -5.080643 -0.446692 0.166521
C -3.778806 -0.325383 0.645830
H -3.459007 0.574108 1.172804
H -5.793422 0.365362 0.314108
H -6.489782 -1.705148 -0.877331
H -4.865899 -3.559407 -1.206901
H -2.535664 -3.337720 -0.354412
O -0.705990 -2.248314 0.734646
C 0.648079 -2.252873 1.174378
H 0.838078 -3.282766 1.490486
H 0.772707 -1.576234 2.030839
C 1.583211 -1.861756 0.022757
H 1.107582 -2.108427 -0.932555
Br 3.170199 -3.055146 0.103977
C 2.104718 -0.455115 0.025545
C 2.404706 0.220004 -1.156758
H 3.174707 0.990272 -1.197040
H 2.134613 -0.235479 -2.112186
H 2.625905 -0.175385 0.948827
H 1.399158 1.348593 2.194412
H -0.284043 2.721436 3.047287

H 0.246274 4.034079 2.011845
H -1.981411 3.795724 1.297178
H -1.988485 1.795505 -0.657886
H -0.440938 2.821900 -2.166321

1c
C 1.176064 1.184663 -0.261337
C 2.419916 0.405042 0.001335
C 3.635812 0.957289 -0.414966
C 2.392226 -0.838715 0.641941
C 4.823772 0.266924 -0.191573
H 3.630688 1.928109 -0.912006
C 3.583971 -1.526015 0.863262
H 1.439677 -1.260514 0.962681
C 4.797206 -0.974465 0.447733
H 5.772804 0.695770 -0.515422
H 3.566568 -2.495833 1.361823
H 5.728357 -1.515563 0.622939
O 1.142156 2.255036 -0.812643
O 0.075035 0.566355 0.183276
C -1.155762 1.231298 -0.046493
H -1.272217 1.454720 -1.117410
H -1.171262 2.184237 0.506154
C -2.282121 0.357601 0.460230
H -2.102093 0.065661 1.501061
C -3.613259 1.014661 0.300898
H -3.861900 1.364548 -0.706616
C -4.461451 1.193045 1.313012
H -4.223372 0.842981 2.320984
H -5.419391 1.694118 1.164658
Br -2.277818 -1.347352 -0.538233

[Rh(cod)Cl]$_2$
Rh 1.664930 -0.319610 -0.010286
C 2.754555 0.875995 1.375647
C 3.245401 -0.436307 1.385628
C 4.506276 -0.908608 0.694038
H 4.477015 -2.007549 0.676083
H 5.393307 -0.632703 1.290309
C 4.629940 -0.384287 -0.745762
C 3.270541 -0.215703 -1.407232
C 2.552138 0.987111 -1.414094
C 2.973301 2.268498 -0.727852
H 2.094891 2.929643 -0.703925
H 3.736969 2.790084 -1.330622
C 3.470704 2.039848 0.708103
H 3.304042 2.950030 1.299313
H 4.554476 1.859207 0.725847
H 1.801711 1.110435 -2.201685
H 3.017554 -0.947185 -2.181005
H 5.231169 -1.087487 -1.337104
H 5.168389 0.573369 -0.771692
H 2.879059 -1.096732 2.178458
H 2.026409 1.137552 2.149624
Cl -0.010708 -1.090974 -1.696320
Rh -1.664964 -0.319687 0.010118
C -2.551800 0.986734 1.414448
C -3.270343 -0.215976 1.407453
C -4.629899 -0.384155 0.746214
H -5.231237 -1.087220 1.337604
H -5.168064 0.573654 0.772273
C -4.506550 -0.908368 -0.693633
C -3.245785 -0.436008 -1.385376
C -2.754860 0.876259 -1.375348
C -3.470607 2.040125 -0.707396
H -3.303755 2.950402 -1.298404
H -4.554435 1.859808 -0.725043
C -2.972937 2.268323 0.728555
H -2.094460 2.929378 0.704641
H -3.736448 2.789854 1.331572
H -2.026926 1.137850 -2.149508
H -2.879658 -1.096314 -2.178408
H -4.477335 -2.007314 -0.675773
H -5.393673 -0.632368 -1.289722
H -3.017311 -0.947689 2.180990
H -1.801198 1.109792 2.201915
Cl 0.010742 -1.092027 1.695230

SU
C 3.476306 -1.568740 0.029234
C 2.465535 -0.590981 0.007011
C 2.827203 0.759234 -0.022282
C 4.181164 1.103942 -0.028640
C 5.188812 0.151001 -0.006927
C 4.809931 -1.194013 0.021881
H 3.209292 -2.625794 0.051786
H 2.063586 1.531803 -0.039676
H 1.041694 -2.051495 0.034750
C 0.000005 -0.281874 -0.000298
H -1.041680 -2.051436 -0.037854

C -2.465529 -0.590971 -0.008014
C -2.827188 0.759243 0.021558
C -3.476297 -1.568734 -0.029851
C -4.181143 1.103945 0.028609
H -2.063551 1.531799 0.038656
C -4.809923 -1.194011 -0.021882
H -3.209293 -2.625785 -0.052583
C -5.188796 0.150995 0.007252
O 0.000010 0.931153 0.000684
N 1.146185 -1.048572 0.013274
N -1.146175 -1.048542 -0.015116
H 6.239842 0.438993 -0.012346
C 4.515522 2.573319 -0.061203
F 4.008000 3.170182 -1.151577
F 5.833471 2.797265 -0.063983
F 4.004265 3.219080 0.999065
C 5.897039 -2.237003 0.045646
F 6.698045 -2.136989 -1.026853
F 5.411866 -3.482803 0.063254
F 6.685032 -2.101524 1.123958
C -4.515522 2.573313 0.061345
F -4.005745 3.218891 -0.999752
F -4.006566 3.170416 1.150905
F -5.833483 2.797186 0.065843
C -5.897042 -2.237001 -0.045177
F -6.697267 -2.137313 1.027940
F -5.411873 -3.482791 -0.063489
F -6.685820 -2.101208 -1.122873
H -6.239828 0.438976 0.013185

ii−TS−a

C -0.941739 4.183370 -1.731144
C -2.265189 3.804479 -1.668693
C -3.316321 4.409274 -0.756980
H -3.066046 5.460549 -0.568572
H -4.271905 4.415499 -1.296917
C -3.491645 3.636419 0.557028
C -2.202409 3.088225 1.115053
C -0.975467 3.743559 1.138077
C -0.722811 5.152451 0.631466
H -1.620331 5.765629 0.782153
H 0.062211 5.604216 1.250998
C -0.260714 5.191317 -0.836528
H 0.817712 4.987982 -0.880406
H -0.406691 6.198732 -1.261569
H -0.209441 3.316507 1.791717
Rh -0.960209 2.303136 -0.528434
Cl -0.182554 0.648977 1.027220
C -0.110384 0.287535 -2.176069
C -1.348095 0.952002 -2.277408
C -2.353153 0.883956 -1.275996
H -3.343549 1.288523 -1.493139
H -2.324234 0.080624 -0.536863
H -1.528713 1.539205 -3.182221
C 1.093348 0.783267 -2.948316
H 0.916767 1.804619 -3.314719
H 1.325467 0.132485 -3.794686
O 2.257242 0.764665 -2.124004
C 2.365273 1.698371 -1.180786
O 1.493946 2.512877 -0.948387
C 3.662721 1.656907 -0.462718
C 3.709812 2.125864 0.854945
C 4.925180 2.142053 1.534338
C 6.090627 1.721321 0.889181
C 6.042892 1.265913 -0.429847
C 4.825298 1.216594 -1.105180
H 4.772863 0.860009 -2.134049
H 6.955597 0.943656 -0.932435
H 7.043454 1.747532 1.420522
H 4.965677 2.486394 2.568284
H 2.789390 2.462402 1.336195
H 0.109415 -0.270622 -1.264090
H -2.309441 2.222070 1.773622
H -4.171845 2.787674 0.396851
H -3.974541 4.272386 1.318045
H -2.650046 3.235274 -2.517537
H -0.378116 3.893084 -2.623666
Br -0.401556 -1.996915 -3.288972
C -0.231152 -2.468576 0.713683
H -1.278629 -2.585489 -1.049994
H 0.764092 -2.547089 -1.080389
N -1.384622 -2.508541 -0.042021
N 0.895394 -2.434865 -0.079970
O -0.213013 -2.479481 1.927205
C -2.676004 -2.259589 0.411338
C -2.963210 -1.714611 1.668044
C -3.727794 -2.489422 -0.492772
C -4.285187 -1.406708 1.992554
H -2.158501 -1.519134 2.372993
C -5.030915 -2.169773 -0.140422

H -3.510948 -2.909066 -1.476422
C -5.334793 -1.622883 1.108237
H -6.360969 -1.376067 1.378056
C 2.193563 -2.155959 0.340277
C 3.229705 -2.353666 -0.583467
C 2.500793 -1.644392 1.608197
C 4.542703 -2.061164 -0.233825
H 2.999855 -2.736224 -1.580442
C 3.826959 -1.359530 1.927304
C 4.867274 -1.566293 1.027328
H 5.897053 -1.334918 1.292577
C -4.537103 -0.790814 3.344376
C -6.146913 -2.360612 -1.133911
F -5.837119 -0.578514 3.575567
F -4.076675 -1.565099 4.338579
F -3.915366 0.395095 3.466435
F -6.493951 -1.195802 -1.710504
F -5.814605 -3.199237 -2.121329
F -7.254191 -2.843300 -0.552842
H 1.707917 -1.466511 2.330046
C 4.108457 -0.832921 3.310260
C 5.617295 -2.309438 -1.258447
F 5.370308 -0.411124 3.452601
F 3.304620 0.194225 3.628131
F 3.904018 -1.776129 4.247428
F 6.816039 -1.871385 -0.858765
F 5.748026 -3.618769 -1.533061
F 5.341953 -1.705676 -2.426991

ii−TS−b

C 1.959041 -1.194333 -1.791793
C 3.064852 -0.663719 -1.168855
C 4.139235 -1.458912 -0.452493
H 4.226258 -2.446160 -0.922703
H 5.100812 -0.956128 -0.617525
C 3.896584 -1.584250 1.057649
C 2.444480 -1.744433 1.430436
C 1.508562 -2.519217 0.754727
C 1.793815 -3.380914 -0.462136
H 2.815654 -3.776612 -0.400049
H 1.127586 -4.252157 -0.426788
C 1.559685 -2.649716 -1.797383
H 0.493327 -2.694741 -2.052425
H 2.100145 -3.158304 -2.613558
H 0.572656 -2.719122 1.284486
Rh 1.198694 -0.411927 0.181402
Cl -0.258036 -0.208309 2.084074
C -0.021007 1.923162 -0.248402
C 1.354952 1.737911 -0.396137
C 2.175397 1.389518 0.716637
H 3.259516 1.379505 0.594806
H 1.817308 1.612148 1.724741
H 1.785385 1.819228 -1.397657
C -0.999721 1.756468 -1.383109
H -0.525244 1.281935 -2.253007
H -1.423458 2.721281 -1.673035
O -2.111074 0.971524 -0.942997
C -1.948172 -0.340146 -0.817360
O -0.892460 -0.900945 -1.050960
C -3.169776 -1.049383 -0.366550
C -3.074351 -2.422236 -0.109469
C -4.195639 -3.122492 0.324887
C -5.409206 -2.452935 0.498844
C -5.504136 -1.083976 0.240757
C -4.385207 -0.376949 -0.191902
H -4.446418 0.691942 -0.394855
H -6.453758 -0.565986 0.378277
H -6.288005 -3.002679 0.839270
H -4.125699 -4.191208 0.529505
H -2.115605 -2.923789 -0.250046
H -0.452272 1.904392 0.751710
H 2.182953 -1.431932 2.444675
H 4.275626 -0.687497 1.567381
H 4.469627 -2.431423 1.470685
H 3.335079 0.359536 -1.438322
H 1.428301 -0.557586 -2.506450
Br -0.280642 4.598801 0.076861

iii−a

C -4.624771 -0.955625 -2.580867
C -4.853886 -2.233889 -2.111700
C -5.554338 -2.593524 -0.826261
H -6.642663 -2.581254 -1.004624
H -5.303912 -3.636234 -0.588359
C -5.210145 -1.694724 0.373141
C -3.773539 -1.210964 0.411717
C -3.325404 -0.033354 -0.126930
C -4.148616 0.946438 -0.919471
H -4.690086 1.586005 -0.199781
H -3.462088 1.614545 -1.453796

C -5.134619 0.307486 -1.911809
H -5.354983 1.041334 -2.697102
H -6.091795 0.086607 -1.422139
H -2.351019 0.336462 0.213630
Rh -2.688624 -1.698425 -1.640516
Cl -2.148744 -2.640354 -3.736619
C -0.777959 -1.850801 -0.491830
C -1.155580 -3.098940 -0.983454
C -2.460031 -3.604871 -0.698212
H -2.806875 -4.444389 -1.304764
H -2.868154 -3.560987 0.311198
H -0.612044 -3.510325 -1.835654
C 0.357282 -1.099065 -1.121078
H 0.515698 -1.431175 -2.157179
H 1.275078 -1.264050 -0.540099
O 0.163486 0.322333 -1.107718
C -0.736532 0.861565 -1.923291
O -1.621082 0.215792 -2.467493
C -0.597319 2.323948 -2.101931
C 0.441551 3.037875 -1.490576
C 0.517840 4.417142 -1.667457
C -0.425201 5.075771 -2.459344
C -1.448516 4.359023 -3.084040
C -1.537289 2.982531 -2.906241
H -2.325591 2.405140 -3.391424
H -2.176953 4.875420 -3.709608
H -0.358764 6.156192 -2.595698
H 1.319233 4.980333 -1.187926
H 1.175919 2.515650 -0.875921
H -1.088243 -1.525264 0.505246
H -3.120230 -1.691902 1.144951
H -5.402224 -2.254547 1.297611
H -5.867736 -0.816652 0.409277
H -4.704077 -3.055887 -2.816666
H -4.281698 -0.864545 -3.615020
Br 1.219650 -3.159833 2.053300
C 1.948555 0.666362 1.409658
H 2.641895 -1.252630 1.558421
H 0.809280 -0.828597 2.231109
N 2.908475 -0.307751 1.278055
N 0.788225 0.154365 1.951161
O 2.098843 1.830113 1.086040
C 4.188960 -0.166932 0.749771
C 4.745971 1.049634 0.344008
C 4.950388 -1.344603 0.628256
C 6.042210 1.066909 -0.177633
H 4.176419 1.970632 0.435027
C 6.233461 -1.292595 0.109277
H 4.521479 -2.296924 0.944557
C 6.804115 -0.085294 -0.305601
H 7.813807 -0.052782 -0.714381
C -0.414735 0.817156 2.139864
C -0.684423 2.106963 1.658685
C -1.428177 0.117638 2.821953
C -1.947757 2.661351 1.857005
H 0.087666 2.670448 1.143991
C -2.676660 0.698415 2.998987
C -2.960956 1.978896 2.522376
H -3.946089 2.424793 2.660319
C 6.593308 2.402029 -0.606698
C 7.052592 -2.549023 -0.032014
F 6.607853 3.280860 0.408551
F 7.843611 2.314044 -1.072096
F 5.846845 2.955443 -1.576936
F 8.220596 -2.451702 0.622991
F 6.421733 -3.628849 0.438988
F 7.356987 -2.795560 -1.316985
H -1.225699 -0.885476 3.203780
C -3.772516 -0.061708 3.700569
C -2.227207 4.018428 1.261298
F -4.936925 0.044222 3.038000
F -3.497396 -1.367383 3.805297
F -3.990369 0.399606 4.939599
F -1.155045 4.817491 1.305597
F -2.584440 3.917240 -0.030940
F -3.226092 4.648954 1.891196

iii‒b

C 1.990308 2.050557 -0.973822
C 3.018025 1.176331 -1.236960
C 3.100858 0.346263 -2.500754
H 2.602879 0.895732 -3.310579
H 4.156275 0.262457 -2.789749
C 2.517290 -1.064942 -2.366926
C 1.269892 -1.160619 -1.528428
C 0.229400 -0.270692 -1.493725
C 0.105285 0.965054 -2.362842
H 0.517714 0.719673 -3.351421
H -0.964479 1.159407 -2.517488
C 0.752777 2.249016 -1.811176

H 0.024507 2.780479 -1.188034
H 0.998439 2.927694 -2.644993
H -0.680055 -0.640358 -1.006058
Rh 1.595880 0.236937 0.371626
Cl 2.614200 1.567166 2.048935
C 0.582077 -1.348770 1.560691
C 1.936905 -1.282914 1.884363
C 2.917015 -1.370103 0.847827
H 3.932142 -1.052707 1.095529
H 2.840802 -2.141077 0.083451
H 2.226282 -0.833357 2.835448
C -0.445152 -0.805960 2.513150
H -0.021630 -0.009395 3.142187
H -0.827478 -1.613338 3.150663
O -1.599283 -0.314165 1.830296
C -1.505230 0.770447 1.083065
O -0.451107 1.350853 0.859549
C -2.802852 1.204937 0.512449
C -3.947334 0.405156 0.626944
C -5.140785 0.830910 0.050103
C -5.194144 2.050455 -0.627745
C -4.054622 2.851191 -0.733617
C -2.856727 2.428757 -0.165827
H -1.958988 3.044670 -0.229950
H -4.102183 3.805931 -1.257949
H -6.132212 2.380613 -1.076423
H -6.032932 0.209179 0.129037
H -3.892291 -0.546025 1.156048
H 0.189379 -2.008492 0.777277
H 1.058638 -2.146328 -1.099860
H 3.275475 -1.735964 -1.940792
H 2.285886 -1.478465 -3.363062
H 3.922571 1.268022 -0.629538
H 2.156514 2.798894 -0.195648
Br -1.721887 -3.251290 -0.528644

iv−Rh

C 1.506148 1.643224 1.233081
C 2.775133 1.230313 0.875165
C 3.577414 1.742982 -0.292712
H 4.091219 2.668469 0.016987
H 4.375562 1.016722 -0.498053
C 2.768185 1.997981 -1.574905
C 1.643724 1.012259 -1.828194
C 0.346027 1.177961 -1.424507
C -0.183350 2.311506 -0.585965
H -0.396948 3.156676 -1.262159
H -1.152021 2.005674 -0.172245
C 0.743842 2.765516 0.553483
H 0.131127 3.265516 1.313875
H 1.461166 3.516559 0.198336
H -0.412161 0.555551 -1.912263
Rh 1.293423 -0.326968 0.126912
Cl 1.591900 -1.403973 2.199994
C 0.622926 -1.994477 -1.198020
C 1.842877 -2.307136 -0.600356
C 2.940584 -1.398585 -0.708803
H 3.773750 -1.550547 -0.018912
H 3.205705 -0.953444 -1.667206
H 1.852772 -3.034353 0.213378
C -0.641820 -2.653886 -0.726818
H -0.529796 -3.008239 0.308072
H -0.894893 -3.500915 -1.376379
O -1.780907 -1.790640 -0.809174
C -1.899400 -0.769531 0.027249
O -0.973843 -0.331903 0.696562
C -3.258097 -0.182211 0.062770
C -4.288164 -0.684362 -0.743297
C -5.549915 -0.100118 -0.684844
C -5.784297 0.973081 0.177518
C -4.759190 1.467882 0.986963
C -3.494218 0.891859 0.931473
H -2.683560 1.258241 1.563157
H -4.948664 2.301730 1.663219
H -6.775807 1.426048 0.221392
H -6.355198 -0.483928 -1.311653
H -4.094880 -1.525080 -1.408834
H 0.582820 -1.495632 -2.170309
H 1.819520 0.264246 -2.604942
H 3.452250 1.956273 -2.431982
H 2.343727 3.009967 -1.574077
H 3.330962 0.642384 1.610520
H 1.144553 1.323967 2.214320

SU·Br

C -0.008698 0.329159 -0.000067
H 0.984537 -1.462592 -0.000058
H -1.008978 -1.458265 -0.000051
N 1.125891 -0.451207 -0.000054
N -1.146659 -0.446400 -0.000045

O -0.005747 1.545606 -0.000044
C 2.445610 -0.019431 -0.000035
C 3.436460 -1.020807 -0.000027
C 2.838520 1.323552 -0.000021
C 4.777363 -0.674224 -0.000004
H 3.136746 -2.070142 -0.000037
C 4.199763 1.638422 0.000002
H 2.089281 2.110754 -0.000027
C 5.186433 0.663143 0.000011
H 6.243569 0.927559 0.000029
C -2.464835 -0.010038 -0.000019
C -3.461115 -1.000400 -0.000006
C -2.851536 1.338012 -0.000000
C -4.804083 -0.645310 0.000025
H -3.169100 -2.053574 -0.000020
C -4.207839 1.660383 0.000030
C -5.204651 0.690142 0.000044
H -6.258270 0.961955 0.000068
C 5.842640 -1.738710 0.000007
C 4.567438 3.099111 0.000019
F 6.641769 -1.636801 1.075485
F 5.335900 -2.975188 -0.000013
F 6.641808 -1.636783 -1.075440
F 4.073505 3.734707 1.075485
F 5.890898 3.295051 0.000024
F 4.073510 3.734731 -1.075435
H -2.097000 2.120257 -0.000009
C -4.566440 3.123457 0.000054
C -5.825713 -1.752337 0.000038
F -4.068755 3.756454 1.075454
F -4.068731 3.756497 -1.075309
F -5.888701 3.327683 0.000045
F -7.080281 -1.287973 0.000073
F -5.697462 -2.546473 -1.075415
F -5.697410 -2.546495 1.075470
Br -0.015137 -3.656028 -0.000086

SU·F

C -0.010079 -0.054545 0.000057
F 0.039862 -3.174246 0.000026
H 0.904343 -1.877572 0.000025
H -0.939780 -1.864656 0.000030
N 1.114521 -0.846086 0.000031
N -1.141207 -0.843241 0.000038
O -0.011935 1.166230 0.000048
C 2.428096 -0.422173 0.000011
C 3.419389 -1.426524 -0.000015
C 2.834825 0.919816 0.000017
C 4.761987 -1.087325 -0.000033
H 3.113596 -2.473368 -0.000019
C 4.197258 1.227315 -0.000002
H 2.089203 1.710636 0.000037
C 5.180269 0.247705 -0.000028
H 6.238775 0.506163 -0.000042
C -2.455177 -0.418635 0.000028
C -3.450869 -1.412528 0.000012
C -2.856349 0.928023 0.000031
C -4.795840 -1.065718 -0.000001
H -3.154219 -2.463646 0.000008
C -4.214063 1.242187 0.000018
C -5.206616 0.266957 0.000002
H -6.261777 0.532287 -0.000009
C 5.821065 -2.156972 -0.000063
C 4.572164 2.685575 -0.000004
F 6.622137 -2.060718 1.075316
F 5.309353 -3.392101 -0.000049
F 6.622076 -2.060718 -1.075487
F 4.082156 3.325382 1.075418
F 5.897070 2.876234 0.000048
F 4.082245 3.325355 -1.075484
H -2.105806 1.714263 0.000043
C -4.580684 2.702755 0.000039
C -5.809587 -2.179066 -0.000018
F -4.087370 3.340000 1.075505
F -4.087131 3.340094 -1.075261
F -5.904509 2.900732 -0.000098
F -7.068180 -1.724606 -0.000035
F -5.676994 -2.973797 -1.075391
F -5.677024 -2.973803 1.075353

v−a

C 3.713874 -2.549125 1.568402
C 3.865259 -3.622509 0.709429
C 4.396017 -3.561624 -0.700414
H 5.498197 -3.574932 -0.658823
H 4.107759 -4.492972 -1.206754
C 3.916435 -2.356253 -1.527072
C 2.492570 -1.916782 -1.248403
C 2.128511 -0.976874 -0.321838
C 3.040640 -0.259594 0.639212

H 3.485398 0.602308 0.111204
H 2.421915 0.166284 1.438007
C 4.147432 -1.131071 1.251660
H 4.480385 -0.660315 2.185202
H 5.026559 -1.164519 0.594790
H 1.113950 -0.577155 -0.425641
Rh 1.682127 -3.040932 0.709097
Cl 1.408279 -4.550739 2.507312
C -0.395041 -2.979292 -0.196038
C 0.083467 -4.283675 -0.137563
C 1.355714 -4.592594 -0.712767
H 1.805606 -5.549272 -0.437211
H 1.621807 -4.218851 -1.701238
H -0.322190 -4.964292 0.613208
C -1.446279 -2.527718 0.775752
H -1.406800 -3.111868 1.706826
H -2.446036 -2.623412 0.330475
O -1.324737 -1.135911 1.084187
C -0.312929 -0.726060 1.835534
O 0.639196 -1.434263 2.130630
C -0.435912 0.686366 2.266730
C -1.551983 1.451428 1.910874
C -1.637605 2.779645 2.321776
C -0.617341 3.337223 3.092137
C 0.492402 2.569647 3.460381
C 0.584909 1.244224 3.051136
H 1.433610 0.625983 3.346796
H 1.282778 3.007651 4.069990
H -0.684978 4.378025 3.411586
H -2.500678 3.380394 2.032650
H -2.345982 1.002935 1.313699
H -0.220142 -2.324632 -1.067870
H 1.725375 -2.143786 -1.994722
H 3.990237 -2.614038 -2.591410
H 4.577419 -1.493049 -1.376909
H 3.810306 -4.621291 1.151047
H 3.519635 -2.784011 2.618383
F -0.213216 -1.113612 -2.496619
C -1.173383 1.668645 -1.315631
H -1.558776 -0.227914 -1.932182
H 0.203415 0.396573 -2.130286
N -2.024634 0.611546 -1.551952
N 0.111409 1.327668 -1.670396
O -1.506547 2.752266 -0.868933
C -3.364345 0.525805 -1.225284
C -4.002722 -0.704563 -1.460979
C -4.109818 1.570599 -0.655535
C -5.335043 -0.885572 -1.114075
H -3.438682 -1.520376 -1.918913
C -5.445332 1.356127 -0.318506
H -3.639242 2.534294 -0.473398
C -6.083156 0.138606 -0.533824
H -7.125189 -0.011044 -0.258052
C 1.255238 2.054689 -1.386817
C 2.397618 1.796892 -2.158628
C 1.341059 2.966284 -0.319646
C 3.598078 2.438186 -1.867811
H 2.335147 1.088415 -2.986910
C 2.551680 3.601964 -0.064044
C 3.696170 3.352093 -0.822565
H 4.635940 3.858642 -0.604971
C -5.963798 -2.224434 -1.398243
C -6.184206 2.503830 0.319257
F -5.186980 -3.235758 -0.975466
F -7.154574 -2.364065 -0.806531
F -6.153101 -2.415820 -2.714632
F -5.629993 2.854828 1.492848
F -6.160807 3.601330 -0.453677
F -7.467845 2.216937 0.560768
H 0.467074 3.163943 0.300178
C 2.660590 4.547634 1.104083
C 4.821827 2.075208 -2.665966
F 3.169590 3.934025 2.188118
F 3.473389 5.580120 0.835609
F 1.476855 5.055734 1.462595
F 5.771522 3.014195 -2.597484
F 5.372824 0.930740 -2.215840
F 4.541826 1.879280 -3.960950

v–b

C -1.313673 -1.963193 1.109626
C -2.524560 -1.840360 0.473675
C -2.814528 -2.454593 -0.878264
H -2.188888 -3.349121 -0.998011
H -3.855544 -2.802725 -0.885017
C -2.609252 -1.490121 -2.050681
C -1.411927 -0.586038 -1.931160
C -0.184372 -0.902903 -1.407743
C 0.247299 -2.274873 -0.924362
H -0.198638 -3.019118 -1.599397

H 1.334368 -2.353687 -1.060166
C -0.078834 -2.617536 0.542821
H 0.763362 -2.319266 1.177128
H -0.176753 -3.710400 0.654376
H 0.601529 -0.173391 -1.631607
Rh -1.348307 0.191032 0.310435
Cl -2.020890 0.741734 2.527958
C -0.680271 2.148052 -0.539346
C -1.964910 2.248451 -0.004476
C -2.974565 1.341285 -0.452269
H -3.884755 1.266818 0.146640
H -3.089864 1.137019 -1.515212
H -2.112053 2.762082 0.947185
C 0.474977 2.734938 0.218696
H 0.279783 2.773304 1.300414
H 0.686347 3.746540 -0.151207
O 1.686523 2.008685 -0.017715
C 1.826249 0.806686 0.514879
O 0.927544 0.217138 1.096823
C 3.178217 0.227545 0.317021
C 4.125269 0.858820 -0.499184
C 5.374375 0.271530 -0.680857
C 5.680140 -0.933345 -0.044818
C 4.738605 -1.557458 0.776436
C 3.486081 -0.978961 0.957313
H 2.742064 -1.445583 1.603850
H 4.983238 -2.494465 1.277371
H 6.661068 -1.388768 -0.188590
H 6.113012 0.755035 -1.320751
H 3.875310 1.798721 -0.990497
H -0.488225 1.905556 -1.616204
H -1.409381 0.333202 -2.530936
H -3.504992 -0.864374 -2.169566
H -2.515313 -2.050685 -2.996408
H -3.373031 -1.487688 1.066476
H -1.279902 -1.732630 2.176813
F -0.143586 1.719284 -3.250917

v−TS−a

C 0.589579 4.619497 0.584472
C 1.945927 4.395823 0.494284
C 2.851493 4.904184 -0.610943
H 2.447207 5.846469 -1.000637
H 3.824284 5.149854 -0.166085
C 3.062344 3.882681 -1.735450
C 1.831818 3.074780 -2.060635
C 0.528809 3.553519 -2.119442
C 0.111338 4.997948 -1.906507
H 0.904150 5.666159 -2.265869
H -0.763830 5.196766 -2.537709
C -0.265695 5.314777 -0.447307
H -1.305083 5.009294 -0.268403
H -0.223582 6.401983 -0.266034
H -0.206296 2.899821 -2.597703
Rh 0.785404 2.526598 -0.184036
Cl 0.180371 0.477364 -1.299349
C 0.249657 0.985243 1.849766
C 1.464396 1.674889 1.776386
C 2.392594 1.476733 0.713309
H 3.347382 2.005120 0.750340
H 2.397959 0.532366 0.165309
H 1.654464 2.453345 2.520020
C -0.917114 1.513641 2.633827
H -0.851480 2.602773 2.765094
H -0.949725 1.023102 3.610677
O -2.147049 1.181548 1.990099
C -2.422069 1.792150 0.840786
O -1.654087 2.563034 0.296409
C -3.761611 1.456575 0.299364
C -3.967009 1.562702 -1.081164
C -5.227288 1.298097 -1.610358
C -6.283144 0.959384 -0.760194
C -6.078469 0.865887 0.617713
C -4.812412 1.098568 1.150784
H -4.636988 1.021121 2.223984
H -6.905765 0.604210 1.278773
H -7.273222 0.765479 -1.176220
H -5.388843 1.359391 -2.687164
H -3.131295 1.840738 -1.726182
H 0.019849 0.232238 1.099855
H 2.017997 2.101755 -2.522411
H 3.857397 3.179856 -1.449558
H 3.416740 4.384046 -2.651815
H 2.451873 4.077884 1.408379
H 0.120499 4.474005 1.562594
C 0.419009 -2.353962 0.341642
H 1.357698 -1.496229 1.939008
H -0.584214 -1.694536 1.999158
N 1.541305 -1.983274 1.045757
N -0.724829 -2.049784 1.052457

O 0.420928 -2.891049 -0.750318
C 2.858553 -2.079779 0.631087
C 3.271803 -2.640040 -0.587431
C 3.832740 -1.545081 1.492422
C 4.627981 -2.649168 -0.911715
H 2.535417 -3.058962 -1.268767
C 5.174763 -1.563087 1.136203
H 3.521117 -1.104510 2.442751
C 5.600239 -2.115819 -0.071116
H 6.653257 -2.128022 -0.345105
C -2.024501 -2.098922 0.556659
C -3.079754 -2.193070 1.473877
C -2.317169 -2.001707 -0.810607
C -4.396041 -2.207819 1.024907
H -2.861187 -2.259643 2.541521
C -3.645377 -2.008304 -1.228735
C -4.702749 -2.120460 -0.330611
H -5.735282 -2.123645 -0.674656
C 5.016230 -3.264353 -2.231340
C 6.162150 -0.941584 2.088276
F 4.413816 -2.648247 -3.261831
F 6.334161 -3.207964 -2.454651
F 4.658196 -4.556994 -2.300548
F 6.088409 -1.488585 3.311961
F 7.425740 -1.070843 1.671242
F 5.930926 0.373944 2.247277
H -1.508559 -1.905407 -1.531865
C -3.912395 -1.896118 -2.706506
C -5.492445 -2.339767 2.047819
F -3.220424 -0.889697 -3.264338
F -3.549861 -3.013044 -3.362365
F -5.206171 -1.687536 -2.979201
F -5.452762 -3.533090 2.665967
F -5.391117 -1.412421 3.015564
F -6.711288 -2.218316 1.510136
F 0.524578 -0.637654 3.085357

v−TS−b

C -1.434373 -2.194761 0.680706
C -2.707152 -1.728969 0.382584
C -3.413144 -1.863789 -0.942595
H -3.886877 -2.858488 -0.995133
H -4.237741 -1.137568 -0.955963
C -2.514166 -1.640387 -2.168127
C -1.451990 -0.575762 -1.973204
C -0.171232 -0.805621 -1.543320
C 0.396536 -2.130881 -1.102745
H 0.730845 -2.677382 -2.000960
H 1.302744 -1.930204 -0.516764
C -0.565459 -2.994649 -0.272497
H 0.029797 -3.702319 0.318375
H -1.207437 -3.604547 -0.921237
H 0.565041 -0.018395 -1.737951
Rh -1.312491 -0.000276 0.315620
Cl -1.822464 0.328693 2.609227
C -0.616144 2.236742 -0.508952
C -1.814714 2.155551 0.191484
C -2.889353 1.317920 -0.226916
H -3.744668 1.231198 0.447010
H -3.120685 1.196908 -1.284922
H -1.843609 2.568794 1.200881
C 0.633384 2.688165 0.192075
H 0.495230 2.675108 1.282146
H 0.863284 3.697226 -0.161479
O 1.779011 1.895251 -0.143542
C 1.922017 0.701550 0.413831
O 1.026703 0.129210 1.012520
C 3.272148 0.115341 0.220119
C 4.249424 0.772450 -0.537755
C 5.501510 0.185607 -0.699752
C 5.779572 -1.045409 -0.102205
C 4.807370 -1.696411 0.660518
C 3.552348 -1.117907 0.822481
H 2.781216 -1.607041 1.419693
H 5.030185 -2.654678 1.130484
H 6.763054 -1.500176 -0.229730
H 6.264922 0.690816 -1.292090
H 4.022213 1.734862 -0.995350
H -0.528760 1.923223 -1.546223
H -1.636468 0.381823 -2.464966
H -3.143112 -1.339728 -3.015836
H -2.021752 -2.573302 -2.470312
H -3.346300 -1.462876 1.228362
H -1.162457 -2.228590 1.739283
F -0.732211 4.127955 -1.622586

vi

C -2.007067 -0.507974 1.677718
C -3.074376 -0.277263 0.823082
C -3.912238 -1.366542 0.172458

H -4.948230 -1.010533 0.096361
H -3.940945 -2.240542 0.836674
C -3.418468 -1.756226 -1.228696
C -1.915831 -1.731839 -1.376659
C -0.994648 -2.182729 -0.451105
C -1.348818 -2.851180 0.864584
H -0.574760 -3.594768 1.097443
H -2.283661 -3.415665 0.748072
C -1.444147 -1.862636 2.042123
H -2.051036 -2.294289 2.857041
H -0.439929 -1.694598 2.451214
H 0.041197 -2.276599 -0.792350
Rh -1.168144 0.056062 -0.244626
Cl 0.499189 0.357869 -1.996540
O 0.918531 0.066422 1.460288
C 1.882047 0.545091 0.901931
O 2.012991 1.848006 0.669196
C 0.994251 2.733648 1.123022
H 1.504189 3.682369 1.323233
H 0.547667 2.349928 2.050020
C -0.063535 2.988363 0.046052
C -1.344381 2.212195 0.165466
C -2.112595 1.893503 -0.949266
H -3.193758 1.769306 -0.886081
H -1.707997 2.075953 -1.947813
H -1.829485 2.309029 1.144394
F -0.406963 4.349609 0.168597
H 0.387060 2.854412 -0.946428
C 3.038461 -0.256011 0.417528
C 2.991231 -1.643792 0.587981
C 4.047130 -2.433462 0.140925
C 5.150528 -1.836567 -0.473051
C 5.199559 -0.451023 -0.638932
C 4.144841 0.343093 -0.194041
H 4.170294 1.425119 -0.321087
H 6.063240 0.011778 -1.117778
H 5.977963 -2.455217 -0.823936
H 4.011927 -3.515720 0.270663
H 2.119375 -2.088131 1.071148
H -1.545319 -1.521731 -2.383443
H -3.830608 -1.050485 -1.964106
H -3.804781 -2.751780 -1.508366
H -3.546937 0.705890 0.889909
H -1.706402 0.305901 2.344620

vii

C 1.742774 -1.392844 -1.533300
C 2.330361 -0.122394 -1.505521
C 3.770884 0.129973 -1.087857
H 4.176845 0.958505 -1.683392
H 4.379681 -0.750648 -1.336663
C 3.886177 0.472869 0.407183
C 2.814279 -0.188354 1.247875
C 2.450305 -1.541818 1.170812
C 3.136875 -2.542794 0.252296
H 3.168785 -3.521776 0.748455
H 4.183627 -2.241060 0.105582
C 2.410157 -2.679618 -1.095687
H 3.099739 -3.040144 -1.878466
H 1.622416 -3.441697 -1.004326
H 1.914195 -1.959947 2.028143
Rh 1.023123 -0.355828 0.164384
Cl -0.309915 0.104007 2.166754
O -0.728710 0.283679 -1.012773
C -1.931546 0.369853 -0.802441
O -2.561981 1.523367 -0.913796
C -1.753733 2.671739 -1.197462
H -2.448270 3.447172 -1.538773
H -1.037992 2.428162 -1.991729
C -1.022171 3.131603 0.052466
C 0.109568 4.062040 -0.253344
C 1.375662 3.719269 -0.019077
H 2.206226 4.380248 -0.273323
H 1.613428 2.750138 0.434365
H -0.148934 5.025268 -0.705117
F -1.956853 3.774834 0.873463
H -0.650526 2.260770 0.614097
C -2.799927 -0.772213 -0.437170
C -2.240211 -2.055120 -0.388497
C -3.036633 -3.142614 -0.044366
C -4.388151 -2.950040 0.252135
C -4.946550 -1.671592 0.201684
C -4.155518 -0.579347 -0.145070
H -4.579900 0.423423 -0.187233
H -6.001822 -1.525655 0.433932
H -5.010542 -3.803762 0.524363
H -2.604211 -4.142766 -0.005711
H -1.182113 -2.193246 -0.619311
H 2.548917 0.343806 2.166828
H 3.787567 1.559839 0.538580

H 4.885669 0.208278 0.794002
H 1.860123 0.657231 -2.114072
H 0.866710 -1.519459 -2.178901

2c
C -0.491458 -0.949556 -0.041078
C -1.818074 -0.271220 0.032394
C -1.933940 1.116962 0.163287
C -2.963629 -1.071467 -0.032794
C -3.197970 1.699800 0.227001
H -1.035332 1.731340 0.215123
C -4.224334 -0.484766 0.030962
H -2.846620 -2.151135 -0.133879
C -4.340892 0.900896 0.160636
H -3.292372 2.781565 0.328693
H -5.118643 -1.106887 -0.020282
H -5.328979 1.361056 0.210412
O -0.338734 -2.140469 -0.140902
O 0.534171 -0.092665 0.014982
C 1.833389 -0.662782 -0.061903
H 1.937213 -1.235433 -0.995651
H 1.997496 -1.345542 0.784629
C 2.836555 0.466263 -0.017808
H 2.652676 1.097981 0.863955
C 4.247176 -0.036562 -0.036069
H 4.533312 -0.622378 -0.915906
C 5.112489 0.189708 0.949895
H 4.829000 0.777307 1.827353
H 6.128536 -0.207201 0.916657
F 2.624927 1.266748 -1.146765

iii–TS-a
C 0.267437 -3.498230 -1.644843
C 1.348931 -2.640140 -1.773202
C 2.235728 -2.150053 -0.655286
H 3.008161 -2.910425 -0.448435
H 2.780327 -1.265531 -1.019694
C 1.477310 -1.795549 0.631218
C 0.114777 -1.176433 0.383501
C -1.082730 -1.854076 0.326886
C -1.268526 -3.349059 0.381610
H -1.339996 -3.655927 1.439346
H -2.244335 -3.575631 -0.069608
C -0.176392 -4.147859 -0.346598
H -0.574111 -5.143702 -0.579574
H 0.694253 -4.310744 0.300894
H -1.990559 -1.264435 0.500782
Rh -0.518993 -1.422623 -1.811899
Cl -0.548235 -1.727831 -4.174315
C -1.673454 0.577610 -1.559864
C -0.628242 0.737399 -2.444529
C 0.746184 0.675719 -2.052861
H 1.497888 0.429010 -2.800717
H 1.051490 0.706844 -1.012014
H -0.837696 0.793247 -3.513893
C -3.100159 0.592192 -2.023872
H -3.192430 0.173877 -3.035859
H -3.479314 1.623956 -2.024743
O -3.945597 -0.109639 -1.117829
C -3.920693 -1.444900 -1.172703
O -3.195178 -2.053888 -1.928838
C -4.834534 -2.083829 -0.191253
C -4.994106 -3.474122 -0.241130
C -5.821285 -4.107992 0.681145
C -6.484570 -3.355647 1.653617
C -6.328287 -1.969030 1.701803
C -5.507462 -1.328888 0.776729
H -5.378862 -0.246848 0.801678
H -6.847801 -1.384387 2.461760
H -7.130279 -3.854105 2.378054
H -5.950784 -5.189956 0.643119
H -4.469751 -4.043070 -1.010469
H -1.527849 0.722038 -0.486945
H 0.041981 -0.104801 0.581855
H 2.068725 -1.079038 1.218213
H 1.354486 -2.675838 1.274665
H 1.735673 -2.484220 -2.784159
H -0.119586 -3.936425 -2.568811
Br 1.482482 3.095320 -2.245079
C 1.496473 1.787911 1.517868
H 0.680489 3.115440 0.185939
H 2.581762 2.443613 -0.091446
N 0.465252 2.545933 0.999517
N 2.647103 1.912367 0.773286
O 1.388111 1.068169 2.491625
C -0.891643 2.424567 1.296083
C -1.776115 3.203337 0.525913
C -1.413799 1.523244 2.231316
C -3.145740 3.050650 0.674138
H -1.377126 3.901859 -0.211904

C -2.800123 1.391421 2.352560
H -0.748037 0.920317 2.844364
C -3.683798 2.138567 1.586760
H -4.762127 2.025285 1.693923
C 3.800729 1.136329 0.917200
C 4.622639 0.999104 -0.210926
C 4.161679 0.502550 2.111522
C 5.776310 0.228371 -0.141123
H 4.344507 1.493822 -1.144739
C 5.320864 -0.272066 2.146603
C 6.141798 -0.426324 1.034589
H 7.043604 -1.034506 1.080836
C -4.094100 3.882407 -0.151372
C -3.314720 0.368447 3.334298
F -4.616318 4.892437 0.558823
F -3.496926 4.417187 -1.221980
F -5.125288 3.146819 -0.595421
F -2.794342 0.548466 4.555774
F -4.645432 0.405714 3.455850
F -2.984636 -0.879078 2.956883
H 3.541210 0.609055 2.998344
C 5.653303 -0.961716 3.444974
C 6.599265 0.052033 -1.391122
F 4.724081 -1.878234 3.764302
F 6.830977 -1.592323 3.401028
F 5.701236 -0.095526 4.468439
F 6.682285 1.186104 -2.099128
F 7.847094 -0.347206 -1.122995
F 6.058494 -0.871554 -2.205059

iii–TS-b

C -0.687210 2.509023 0.819550
C 0.460336 2.940315 0.204021
C 0.531252 3.375781 -1.241031
H -0.445492 3.770941 -1.547912
H 1.241902 4.208555 -1.320217
C 0.986187 2.250976 -2.176488
C 0.407944 0.896361 -1.854926
C -0.867747 0.611569 -1.407296
C -1.979425 1.631806 -1.224088
H -1.890413 2.380195 -2.023308
H -2.937641 1.119659 -1.390761
C -2.028009 2.307089 0.157834
H -2.634650 1.692394 0.834186
H -2.540983 3.280538 0.081238
H -1.191654 -0.429806 -1.503113
Rh 0.516809 0.595369 0.387993
Cl 1.290971 0.875899 2.640283
C 0.823421 -1.579855 -0.098779
C 1.962141 -1.047468 0.489248
C 2.732500 -0.038771 -0.158635
H 3.369225 0.607167 0.441759
H 2.720535 0.105855 -1.233623
H 2.186261 -1.269357 1.533187
C -0.073497 -2.527849 0.645982
H 0.001221 -2.377948 1.730938
H 0.202423 -3.561153 0.397303
O -1.428559 -2.391820 0.220160
C -2.135267 -1.383410 0.737219
O -1.674101 -0.601267 1.539475
C -3.514211 -1.295764 0.189465
C -3.895617 -2.017666 -0.947935
C -5.178831 -1.856121 -1.465290
C -6.079408 -0.989104 -0.843482
C -5.703076 -0.281474 0.300616
C -4.419765 -0.432062 0.817396
H -4.108129 0.106656 1.713435
H -6.412409 0.387066 0.789330
H -7.083840 -0.866432 -1.251299
H -5.478108 -2.408759 -2.356356
H -3.184412 -2.692013 -1.425159
H 0.723080 -1.599097 -1.186495
H 0.960426 0.064700 -2.299892
H 2.082109 2.166175 -2.135235
H 0.742643 2.495372 -3.224371
H 1.320455 3.161792 0.842398
H -0.682169 2.454882 1.910784
Br 4.988791 -1.399282 -0.613106

viii

C 3.647795 -1.461715 -0.372827
C 3.828917 -0.640722 0.732227
C 3.554566 -1.019723 2.168100
H 3.508573 -0.088153 2.750067
H 4.405950 -1.594180 2.572299
C 2.240696 -1.793498 2.348689
C 1.160449 -1.336258 1.380585
C 0.904361 -1.934855 0.143912
C 1.693389 -3.071376 -0.467653
H 1.356256 -4.034152 -0.046149

H 1.445790 -3.102990 -1.539118
C 3.213789 -2.913715 -0.307797
H 3.718829 -3.467905 -1.109852
H 3.559777 -3.356835 0.635377
H -0.101003 -1.808720 -0.268593
Rh 1.943270 -0.099120 -0.211551
Cl 3.169283 1.662259 -1.392573
O -3.065553 0.949045 -1.706916
C -2.836174 -0.160511 -1.300880
O -1.671542 -0.793410 -1.531592
C -0.681669 -0.069690 -2.246916
C 0.029678 0.943286 -1.393548
C -0.158507 1.100508 -0.059218
C 0.310922 2.297727 0.699423
H 0.658864 2.054457 1.707900
H 1.070717 2.865930 0.154495
Br -1.208543 3.519192 0.971356
H -0.852479 0.435954 0.466186
H 0.621788 1.679791 -1.943016
H 0.016417 -0.817981 -2.646073
H -1.145043 0.457807 -3.093059
C -3.778137 -0.977171 -0.484780
C -3.454949 -2.266795 -0.047384
C -4.371941 -2.984452 0.717782
C -5.605629 -2.418353 1.043866
C -5.928147 -1.132105 0.605585
C -5.015592 -0.411092 -0.158963
H -5.244995 0.594568 -0.513288
H -6.892794 -0.692339 0.861169
H -6.321066 -2.983641 1.643084
H -4.124054 -3.989860 1.059859
H -2.491309 -2.703482 -0.309626
H 0.345182 -0.767036 1.836302
H 1.878082 -1.647888 3.374703
H 2.398706 -2.874238 2.233567
H 4.435562 0.257123 0.587984
H 4.100134 -1.127990 -1.311287

ix

C -0.337630 -2.762658 1.041561
C -1.545368 -2.049785 1.061927
C -2.735128 -2.353469 0.176077
H -3.451270 -1.527731 0.286403
H -3.251978 -3.259078 0.538512
C -2.356589 -2.500204 -1.308108
C -1.130986 -1.677628 -1.676391
C 0.174476 -2.184574 -1.648340
C 0.567137 -3.572411 -1.186581
H 0.346072 -4.315328 -1.972474
H 1.659636 -3.579634 -1.056954
C -0.099773 -3.966921 0.141336
H 0.538086 -4.689197 0.667866
H -1.056438 -4.477968 -0.038272
H 0.914683 -1.676675 -2.276584
Rh -0.097401 -0.924583 0.028077
Cl 0.476503 0.330960 2.056183
O 0.863460 0.701869 -1.124369
C 1.824694 1.408851 -0.852525
O 1.826492 2.699164 -1.113446
C 0.629376 3.278861 -1.660905
C -0.457296 3.376789 -0.635692
C -1.698660 2.941193 -0.857060
C -2.805803 3.098931 0.122416
H -2.480728 3.578708 1.050466
H -3.667112 3.627940 -0.297789
Br -3.513292 1.339330 0.632325
H -1.945078 2.448782 -1.804458
H -0.195977 3.858234 0.313261
H 0.949044 4.273689 -1.993361
H 0.304664 2.690107 -2.528799
C 3.089172 0.921160 -0.247346
C 3.341984 -0.454978 -0.213726
C 4.519499 -0.928967 0.356389
C 5.441157 -0.030865 0.899383
C 5.191430 1.342024 0.862525
C 4.019297 1.822151 0.283260
H 3.814523 2.891888 0.249213
H 5.912628 2.041122 1.286968
H 6.361070 -0.403856 1.352104
H 4.719019 -2.000781 0.379043
H 2.615190 -1.150074 -0.637650
H -1.309877 -0.797883 -2.303737
H -3.202030 -2.180845 -1.932038
H -2.171511 -3.553819 -1.562448
H -1.768013 -1.469220 1.962871
H 0.307573 -2.678290 1.921210

Pro−(*R*)−TS

C 2.637733 -4.376474 1.165945
C 1.914397 -4.806718 0.076985

C 2.295603 -5.954195 -0.837709
H 2.870155 -6.692893 -0.265585
H 1.372046 -6.459388 -1.148858
C 3.060858 -5.503044 -2.088177
C 4.026341 -4.372822 -1.837109
C 4.845261 -4.227224 -0.724909
C 4.948310 -5.210782 0.427360
H 4.795668 -6.230919 0.052882
H 5.975589 -5.178600 0.811731
C 3.989852 -4.895487 1.591100
H 4.443877 -4.133368 2.237518
H 3.841185 -5.790258 2.218781
H 5.647214 -3.488161 -0.808163
Rh 3.102202 -2.891590 -0.461982
Cl 4.309457 -1.223597 -1.686031
C 1.690100 -0.733186 0.084673
C 1.109335 -1.922028 -0.363279
C 1.405530 -2.469919 -1.646635
H 0.850520 -3.344497 -1.990096
H 1.776381 -1.808065 -2.433245
H 0.471229 -2.474527 0.331269
C 1.820591 -0.416072 1.549572
H 1.687052 -1.314624 2.168113
H 1.075318 0.336072 1.823933
O 3.084923 0.181168 1.829015
C 4.167089 -0.588317 1.789762
O 4.139407 -1.764762 1.478234
C 5.407426 0.135305 2.158352
C 6.628195 -0.373304 1.702136
C 7.809140 0.301492 2.001118
C 7.769228 1.464977 2.773160
C 6.550578 1.960014 3.243249
C 5.364309 1.302889 2.928063
H 4.404492 1.690324 3.270224
H 6.525525 2.866090 3.849899
H 8.695815 1.989216 3.011921
H 8.762571 -0.080168 1.634683
H 6.635666 -1.284608 1.101747
H 2.291907 -0.131379 -0.598614
H 4.271326 -3.762232 -2.709780
H 2.348269 -5.170662 -2.856026
H 3.608687 -6.351496 -2.531682
H 0.870127 -4.491330 0.022813
H 2.117285 -3.759730 1.905320
F 0.210837 0.668482 -0.234278
O -3.033867 3.054419 -2.718618
C -1.914484 2.653310 -2.446880
N -1.621109 1.358728 -2.101827
C -2.544323 0.320270 -1.982660
C -3.917618 0.513191 -1.767685
C -4.745610 -0.592170 -1.602003
C -4.260139 -1.900116 -1.656110
C -2.898050 -2.074228 -1.878028
C -2.042896 -0.985053 -2.029679
H -0.976001 -1.136950 -2.203280
C -2.296699 -3.449828 -1.969782
F -1.483012 -3.706427 -0.924454
F -1.534800 -3.584412 -3.069591
F -3.214469 -4.416447 -2.003134
H -4.924839 -2.755012 -1.532225
C -6.222833 -0.374512 -1.399230
F -6.911792 -0.584957 -2.535879
F -6.501169 0.870326 -1.000872
F -6.735527 -1.213272 -0.485595
H -4.319142 1.521413 -1.713593
H -0.706789 1.147443 -1.692454
N -0.829304 3.503109 -2.490151
H -1.100745 4.476009 -2.454985
C 0.498225 3.186833 -2.110645
C 1.078246 3.828050 -1.029926
C 2.422100 3.494929 -0.657824
C 3.065943 4.096083 0.461966
C 4.358914 3.763151 0.791544
C 5.080474 2.818708 0.019519
C 4.482608 2.214752 -1.059710
C 3.141672 2.527470 -1.418609
C 2.506453 1.902754 -2.528438
C 1.219986 2.226639 -2.869299
H 0.732675 1.768574 -3.731161
H 3.067319 1.167965 -3.109323
H 5.022489 1.479215 -1.657625
H 6.106406 2.564281 0.294602
H 4.835863 4.229260 1.655645
H 2.519024 4.828620 1.057401
C 0.294495 4.838695 -0.261011
C -0.764141 4.431090 0.544617
C -1.511649 5.391445 1.286399
C -1.203490 6.722538 1.195627
C -0.144617 7.184589 0.368779
C 0.170605 8.566253 0.260863

C 1.192940 8.993280 -0.551765
C 1.941962 8.048394 -1.296505
C 1.662866 6.704796 -1.209421
C 0.613406 6.229675 -0.369464
H 2.245909 5.988951 -1.790567
H 2.747574 8.391652 -1.947348
H 1.428123 10.055498 -0.629716
H -0.419943 9.282005 0.836574
H -1.777316 7.451620 1.771095
H -2.322891 5.050961 1.922319
N -1.035481 3.061853 0.642050
C -2.212438 2.478143 1.056146
O -3.213676 3.087882 1.386747
N -2.110321 1.102988 1.051265
C -3.074899 0.189678 1.441668
C -2.686514 -1.162160 1.473815
C -3.587921 -2.142986 1.860677
C -4.899974 -1.826361 2.213761
C -5.275203 -0.488295 2.172970
C -4.392106 0.522628 1.795888
H -4.714169 1.559953 1.773353
C -6.664853 -0.097782 2.606169
F -7.532326 -1.112788 2.502723
F -6.682540 0.295552 3.893999
F -7.153920 0.921521 1.889828
H -5.603777 -2.601040 2.516743
C -3.169519 -3.588545 1.883485
F -3.637323 -4.256379 0.814358
F -1.838077 -3.736832 1.888239
F -3.641914 -4.226096 2.965004
H -1.664320 -1.429768 1.203177
H -1.233441 0.724769 0.672475
H -0.321059 2.419173 0.299032

Pro−(*S*)−TS

C 3.979473 3.045925 1.740308
C 4.685122 1.937929 1.337457
C 6.120823 1.944784 0.854954
H 6.603039 1.028467 1.217104
H 6.658401 2.775684 1.326098
C 6.235995 2.000010 -0.671563
C 5.243949 2.932606 -1.316794
C 4.816763 4.159385 -0.819967
C 5.303785 4.829286 0.452597
H 5.256408 5.915239 0.303056
H 6.361657 4.591602 0.617646
C 4.449699 4.478994 1.683918
H 4.997488 4.707376 2.613861
H 3.551878 5.112127 1.691505
H 4.281208 4.800463 -1.526087
Rh 3.264305 2.653619 -0.380476
Cl 2.302958 3.153431 -2.501045
C 1.480812 1.095780 0.971759
C 1.773369 1.000267 -0.389351
C 3.057797 0.596220 -0.853465
H 3.192489 0.449252 -1.927292
H 3.706963 -0.001284 -0.209415
H 1.008878 1.299249 -1.108702
C 0.388682 1.972329 1.513442
H -0.371349 1.343822 1.991246
H 0.806351 2.648979 2.272467
O -0.269390 2.727704 0.507544
C 0.314234 3.870575 0.117598
O 1.400193 4.215023 0.540530
C -0.490479 4.640292 -0.857773
C -1.696544 4.143741 -1.364914
C -2.412806 4.891814 -2.296009
C -1.927584 6.132283 -2.714367
C -0.723465 6.627777 -2.206912
C -0.001425 5.881632 -1.280979
H 0.945619 6.244634 -0.879873
H -0.347773 7.596340 -2.538088
H -2.490671 6.717088 -3.443197
H -3.350455 4.504419 -2.696006
H -2.060676 3.170065 -1.038530
H 2.199347 0.728955 1.705606
H 5.034504 2.734529 -2.371489
H 7.255721 2.291954 -0.974060
H 6.070792 0.994941 -1.088049
H 4.295350 0.957880 1.629206
H 3.076851 2.885487 2.336754
F 0.478297 -0.661559 1.350981
O -0.773202 -2.202355 -2.717371
C -0.368612 -1.737155 -1.668199
N -1.080958 -0.841370 -0.898180
C -2.373371 -0.393287 -1.137381
C -3.248231 -0.971099 -2.068538
C -4.546745 -0.477611 -2.185682
C -5.011002 0.581758 -1.414253
C -4.127413 1.150279 -0.496261

C -2.831025 0.677752 -0.351709
H -2.158052 1.139190 0.371776
C -4.603874 2.313971 0.331577
F -5.755615 2.042514 0.963591
F -3.710767 2.673259 1.261911
F -4.835377 3.398176 -0.429444
H -6.032758 0.947950 -1.514205
C -5.461844 -1.098279 -3.209725
F -5.331883 -0.501695 -4.409573
F -5.204986 -2.398184 -3.399085
F -6.751506 -0.989880 -2.864935
H -2.919481 -1.807756 -2.677676
H -0.639791 -0.562344 -0.017162
N 0.852318 -2.031429 -1.101157
C 1.769623 -2.964185 -1.601532
C 1.867218 -3.208408 -3.002694
C 2.768070 -4.119919 -3.482963
C 3.619039 -4.850026 -2.609613
C 4.535768 -5.818912 -3.100034
C 5.342863 -6.522911 -2.239205
C 5.259924 -6.286213 -0.844806
C 4.387384 -5.349632 -0.342111
C 3.543008 -4.597331 -1.209530
C 2.617742 -3.620009 -0.715651
C 2.566873 -3.310369 0.742836
C 1.466185 -3.673999 1.500863
C 1.417962 -3.405716 2.897037
C 2.440075 -2.733407 3.509043
C 3.581297 -2.317819 2.768187
C 4.660134 -1.633570 3.393512
C 5.777164 -1.275271 2.677059
C 5.853737 -1.575970 1.293808
C 4.819175 -2.223203 0.658727
C 3.654870 -2.622410 1.377470
H 4.889663 -2.444838 -0.406987
H 6.744437 -1.299933 0.726155
H 6.605527 -0.764004 3.170353
H 4.588477 -1.411036 4.459965
H 2.400794 -2.527720 4.580104
H 0.556161 -3.753660 3.467027
N 0.381077 -4.346707 0.898408
C -0.939651 -3.937210 0.889009
O -1.790198 -4.586645 0.305992
N -1.181448 -2.777131 1.578092
C -2.410580 -2.149996 1.763861
C -3.601601 -2.517656 1.117849
C -4.763218 -1.790923 1.367526
C -4.792392 -0.712865 2.251627
C -3.601954 -0.357164 2.879408
C -2.422881 -1.056158 2.641511
H -1.496099 -0.765055 3.139881
C -3.567426 0.771005 3.876146
F -2.497946 1.564017 3.694709
F -4.655867 1.543129 3.813783
F -3.485540 0.310957 5.137603
H -5.712567 -0.160715 2.436180
C -6.040945 -2.212892 0.687899
F -6.716174 -3.114713 1.423464
F -5.817806 -2.780685 -0.504518
F -6.871647 -1.180049 0.490609
H -3.604246 -3.349657 0.418508
H -0.384045 -2.174604 1.790955
H 0.593073 -4.991769 0.149278
H 4.332097 -5.182204 0.734386
H 5.893330 -6.855177 -0.162590
H 6.042442 -7.266372 -2.623246
H 4.581973 -5.994593 -4.176762
H 2.841267 -4.296027 -4.557872
H 1.219149 -2.659189 -3.678868
H 1.006556 -1.668812 -0.159364

Pro−(*R*)−TS−2j

C 4.036534 -3.075505 1.593442
C 3.141022 -3.934413 0.998718
C 3.474082 -5.254367 0.328875
H 4.346449 -5.697405 0.824005
H 2.639126 -5.943205 0.510066
C 3.694144 -5.140462 -1.186431
C 4.424733 -3.894430 -1.613386
C 5.480140 -3.296842 -0.951922
C 6.140042 -3.810719 0.311835
H 6.107057 -4.906777 0.328271
H 7.202012 -3.535466 0.274441
C 5.538234 -3.210478 1.594988
H 5.955583 -2.205630 1.753377
H 5.839227 -3.803277 2.475369
H 6.006582 -2.504217 -1.493467
Rh 3.599443 -2.162828 -0.418875
Cl 3.924712 -0.814328 -2.367571
C 1.851809 -0.107824 -0.024957

C 1.564872 -1.451554 0.183721
C 1.572932 -2.388336 -0.895528
H 1.185102 -3.394026 -0.730407
H 1.482262 -2.015152 -1.918861
H 1.347458 -1.778596 1.204282
C 2.095259 0.839392 1.125946
H 1.111476 1.087644 1.545351
H 2.559469 1.770810 0.767007
O 2.846592 0.267038 2.173694
H 2.013913 0.256550 -1.038640
H 4.228086 -3.554975 -2.633925
H 2.723944 -5.152533 -1.702657
H 4.243317 -6.020651 -1.560935
H 2.091021 -3.797326 1.266903
H 3.628687 -2.332222 2.286944
F -0.071259 0.815030 -0.423105
O -3.961387 2.792286 -2.206973
C -2.766808 2.562854 -2.118627
N -2.228634 1.312997 -1.964507
C -2.948188 0.117997 -1.927563
C -4.292819 0.024697 -1.541155
C -4.906016 -1.225363 -1.498237
C -4.228557 -2.395194 -1.839803
C -2.894596 -2.285823 -2.221846
C -2.252150 -1.051519 -2.257658
H -1.207638 -0.977980 -2.566909
C -2.104223 -3.500492 -2.628142
F -1.509226 -3.324683 -3.820646
F -2.858662 -4.598577 -2.722190
F -1.116174 -3.767951 -1.755041
H -4.726029 -3.363265 -1.809273
C -6.361992 -1.299293 -1.117917
F -6.698849 -2.503809 -0.640622
F -7.159431 -1.065229 -2.176450
F -6.689756 -0.392303 -0.187234
H -4.839530 0.923711 -1.265189
H -1.250885 1.220522 -1.668741
N -1.842065 3.586921 -2.189514
H -2.245838 4.489342 -1.979824
C -0.441733 3.451731 -2.043462
C 0.209394 4.078785 -0.995971
C 1.625534 3.907465 -0.852102
C 2.351065 4.502230 0.219109
C 3.708907 4.313030 0.341116
C 4.413214 3.524524 -0.601473
C 3.738102 2.927894 -1.640977
C 2.334705 3.099986 -1.790079
C 1.623816 2.491736 -2.862778
C 0.273772 2.672458 -2.993469
H -0.276352 2.228115 -3.823764
H 2.176528 1.891106 -3.588180
H 4.266664 2.305812 -2.366836
H 5.489633 3.383196 -0.492688
H 4.248260 4.774094 1.169701
H 1.814972 5.110523 0.948942
C -0.571575 4.886196 -0.013512
C -1.419299 4.247618 0.886836
C -2.164474 5.009859 1.832911
C -2.062152 6.375278 1.849980
C -1.222965 7.068542 0.936745
C -1.121394 8.486143 0.943128
C -0.309387 9.137522 0.046215
C 0.434372 8.391141 -0.901528
C 0.359100 7.018403 -0.929799
C -0.468434 6.313298 -0.007653
H 0.934684 6.456492 -1.666894
H 1.071915 8.912049 -1.617512
H -0.237285 10.225801 0.057493
H -1.704975 9.047229 1.676031
H -2.631805 6.951732 2.581536
H -2.808049 4.491690 2.537026
N -1.477930 2.850979 0.865705
C -2.492527 2.057025 1.355220
O -3.505416 2.473201 1.888859
N -2.207370 0.721925 1.161340
C -3.005514 -0.361709 1.484145
C -2.468842 -1.637765 1.235574
C -3.210218 -2.776955 1.517781
C -4.503648 -2.693347 2.033259
C -5.022737 -1.424867 2.277098
C -4.299560 -0.263156 2.020526
H -4.730760 0.713060 2.218574
C -6.396252 -1.319062 2.888434
F -6.368413 -1.602185 4.205111
F -6.920597 -0.095155 2.768442
F -7.260129 -2.181999 2.334566
H -5.084076 -3.591087 2.248415
C -2.627601 -4.138969 1.249381
F -3.117264 -4.679116 0.119220
F -1.294622 -4.108124 1.123191

F -2.912069 -5.003651 2.235112
H -1.462257 -1.723170 0.822149
H -1.345894 0.529203 0.630537
H -0.753594 2.358041 0.339564
C 4.207633 0.274278 2.058818
C 4.887514 0.485814 0.861050
C 4.939361 0.032613 3.231523
C 6.290349 0.446124 0.822495
H 4.356282 0.705757 -0.065081
C 6.323417 0.006000 3.196584
H 4.399100 -0.124290 4.166012
C 7.016990 0.208320 1.988906
H 6.786430 0.611603 -0.133465
H 6.902888 -0.176892 4.102214
O 8.365234 0.145503 2.052810
C 9.092129 0.322758 0.859491
H 8.831665 -0.447273 0.115062
H 8.911210 1.318505 0.424281
H 10.151681 0.229844 1.119042

Pro−(*S*)−TS−2j

C -4.458997 -0.080363 -0.791883
C -3.547640 0.496295 -1.643694
C -3.771043 0.825993 -3.106601
H -3.211137 1.743638 -3.331564
H -4.829444 1.067712 -3.261804
C -3.307505 -0.282916 -4.061595
C -3.576457 -1.679710 -3.563932
C -4.706877 -2.108171 -2.883698
C -5.914318 -1.252849 -2.551271
H -6.802837 -1.897013 -2.570953
H -6.063124 -0.500034 -3.334954
C -5.824097 -0.604961 -1.159601
H -6.555108 0.216144 -1.063198
H -6.104244 -1.346863 -0.397357
H -4.834368 -3.190990 -2.789354
Rh -3.074355 -1.777206 -1.402480
Cl -2.358988 -4.017383 -1.755562
C -1.575615 -0.739925 0.749796
C -1.124637 -1.565230 -0.278444
C -1.087181 -1.171389 -1.653389
H -0.589068 -1.844643 -2.354684
H -1.053034 -0.114556 -1.919804
H -0.782219 -2.568922 -0.014870
C -1.913261 -1.236609 2.134369
H -1.601214 -2.282825 2.275771
H -1.371949 -0.602549 2.844704
O -3.282971 -1.052519 2.414443
H -1.953398 0.261754 0.533581
H -2.929426 -2.458356 -3.977526
H -3.779659 -0.160496 -5.050770
H -2.225265 -0.192207 -4.232874
H -2.697102 1.006007 -1.180335
H -4.272818 0.022232 0.283665
F 0.169530 0.173161 1.405311
O 1.481625 2.004465 -2.481838
C 1.087338 1.471035 -1.461936
N 1.675517 0.365515 -0.888545
C 2.871239 -0.226280 -1.280099
C 3.797885 0.382757 -2.136896
C 5.003960 -0.261169 -2.408779
C 5.320687 -1.499626 -1.862814
C 4.383951 -2.096453 -1.017967
C 3.175201 -1.480859 -0.725922
H 2.461163 -1.960567 -0.054315
C 4.703472 -3.448650 -0.438394
F 5.944054 -3.492401 0.071432
F 3.858069 -3.796324 0.538309
F 4.642271 -4.413459 -1.372511
H 6.273877 -1.983200 -2.075536
C 5.975145 0.405397 -3.348369
F 5.691481 0.118436 -4.632852
F 5.948156 1.739928 -3.240854
F 7.236700 0.010640 -3.135830
H 3.584960 1.358052 -2.565254
H 1.227057 0.010758 -0.039016
N -0.018026 1.878428 -0.741182
C -0.811264 2.993085 -1.038838
C -1.007068 3.396266 -2.392199
C -1.804935 4.469709 -2.682571
C -2.442474 5.214918 -1.653776
C -3.241889 6.352394 -1.948573
C -3.836928 7.073459 -0.941891
C -3.648558 6.684540 0.407188
C -2.887559 5.583147 0.721768
C -2.265855 4.807912 -0.299625
C -1.460006 3.657831 -0.003354
C -1.324068 3.186327 1.407110
C -0.105767 3.261559 2.062766
C 0.021061 2.865099 3.423355

C -1.056393 2.368969 4.103572
C -2.324648 2.251703 3.469469
C -3.459927 1.776062 4.178624
C -4.685830 1.688154 3.565730
C -4.819094 2.046767 2.203239
C -3.738721 2.519226 1.492917
C -2.461705 2.656755 2.108955
H -3.861439 2.807282 0.447455
H -5.789556 1.953825 1.711404
H -5.555167 1.337603 4.124235
H -3.337709 1.488393 5.224689
H -0.960729 2.079224 5.151462
H 0.987232 2.984202 3.914017
N 1.035175 3.762498 1.398115
C 2.234662 3.098981 1.217667
O 3.146266 3.615331 0.594826
N 2.286629 1.852028 1.787244
C 3.372536 0.980983 1.814108
C 4.607451 1.223488 1.191346
C 5.613976 0.265419 1.281298
C 5.447884 -0.927473 1.984109
C 4.216342 -1.154522 2.591275
C 3.185511 -0.224048 2.506698
H 2.220904 -0.423616 2.977343
C 3.979016 -2.402617 3.400000
F 2.797779 -2.969923 3.110669
F 4.927654 -3.324497 3.207541
F 3.956216 -2.134877 4.718759
H 6.250803 -1.660526 2.046886
C 6.945431 0.551728 0.635925
F 6.831438 1.357014 -0.428110
F 7.558732 -0.567647 0.227554
F 7.787063 1.160618 1.491355
H 4.763167 2.142990 0.633835
H 1.398182 1.400862 2.009178
H 0.896845 4.507127 0.728301
H -2.748293 5.301424 1.766004
H -4.109710 7.267423 1.205830
H -4.446834 7.946973 -1.175429
H -3.367763 6.643978 -2.993245
H -1.957645 4.768675 -3.721441
H -0.517613 2.836030 -3.184288
H -0.112078 1.453315 0.184917
C -4.201888 -1.955958 1.972132
C -3.910662 -3.075079 1.193923
C -5.534689 -1.701912 2.332695
C -4.942849 -3.920360 0.750732
H -2.888026 -3.347497 0.936856
C -6.548799 -2.545522 1.911509
H -5.749182 -0.830772 2.951810
C -6.266811 -3.658825 1.099886
H -4.671672 -4.773168 0.129604
H -7.586359 -2.353376 2.187463
O -7.324433 -4.402233 0.704350
C -7.076634 -5.506944 -0.133817
H -6.433252 -6.250825 0.362454
H -8.048638 -5.960378 -0.353492
H -6.600212 -5.193304 -1.077272

NMR Spectra

(*S*)-**3e** ^{1}H NMR (400 MHz, DMSO-d_6)

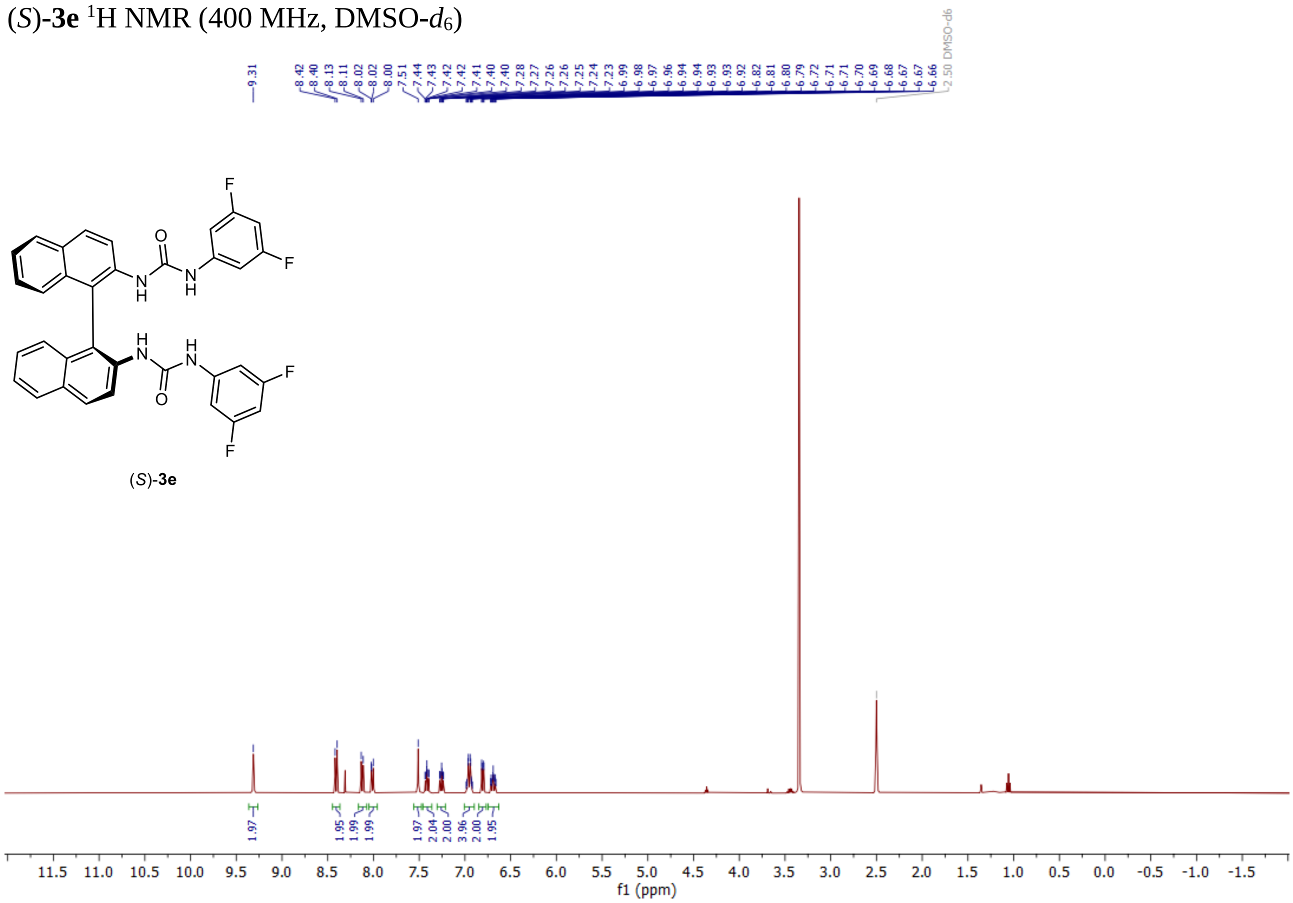

(*S*)-**3e** ^{13}C NMR (101 MHz, DMSO-d_6)

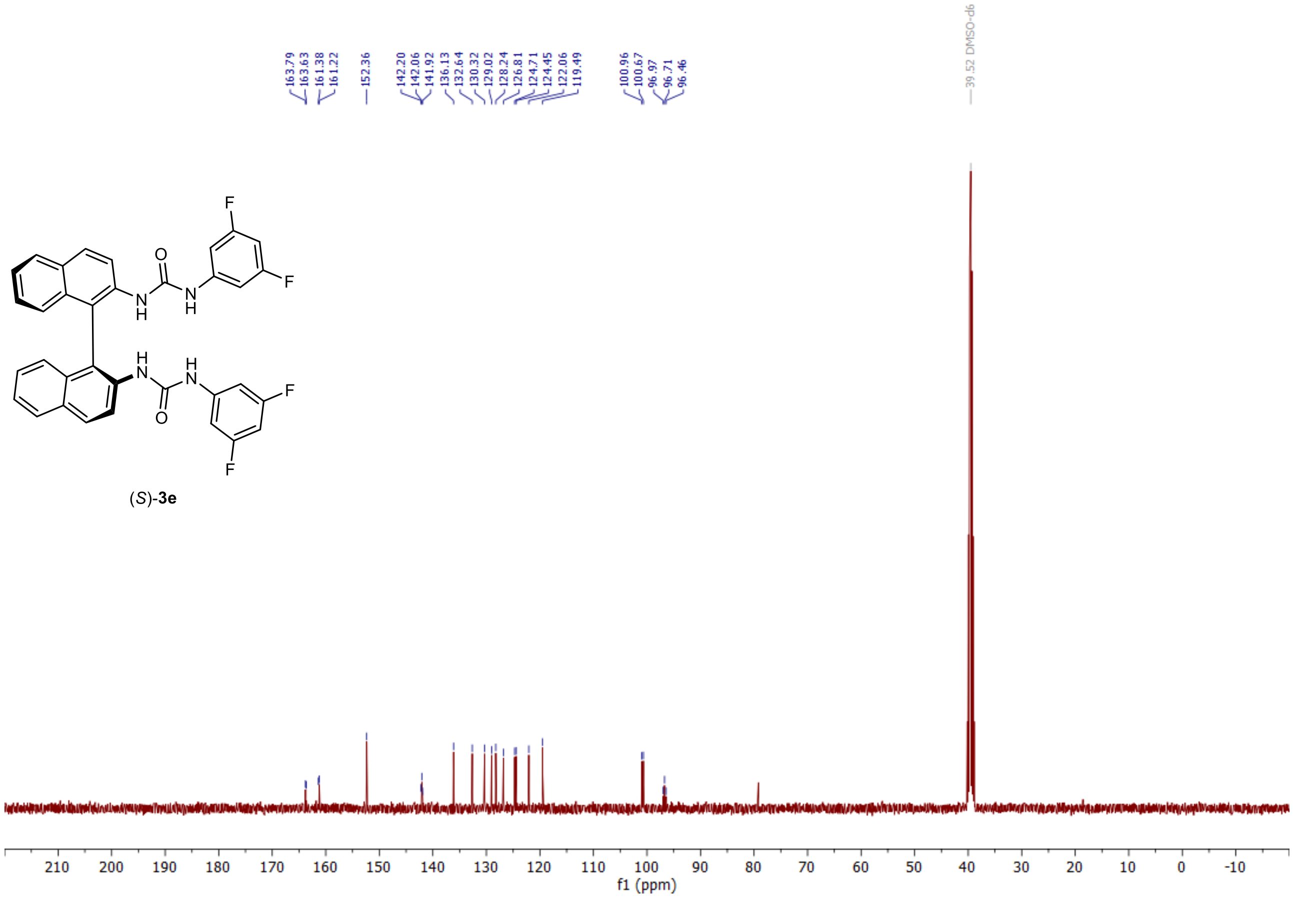

(*S*)-**3e** ^{19}F NMR (377 MHz, DMSO-d_6)

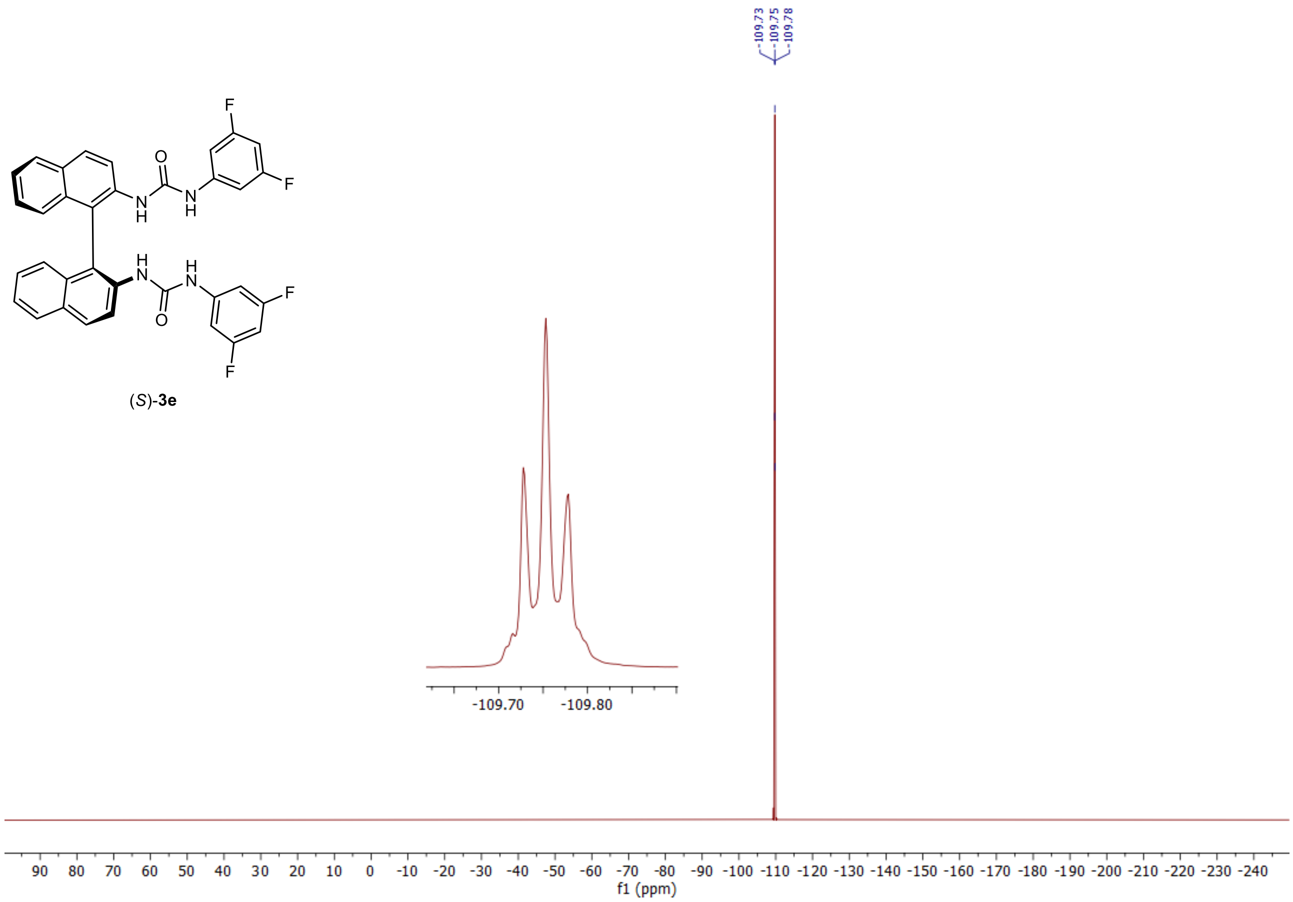

(*S*)-**3f** ^{1}H NMR (400 MHz, $CDCl_3$)

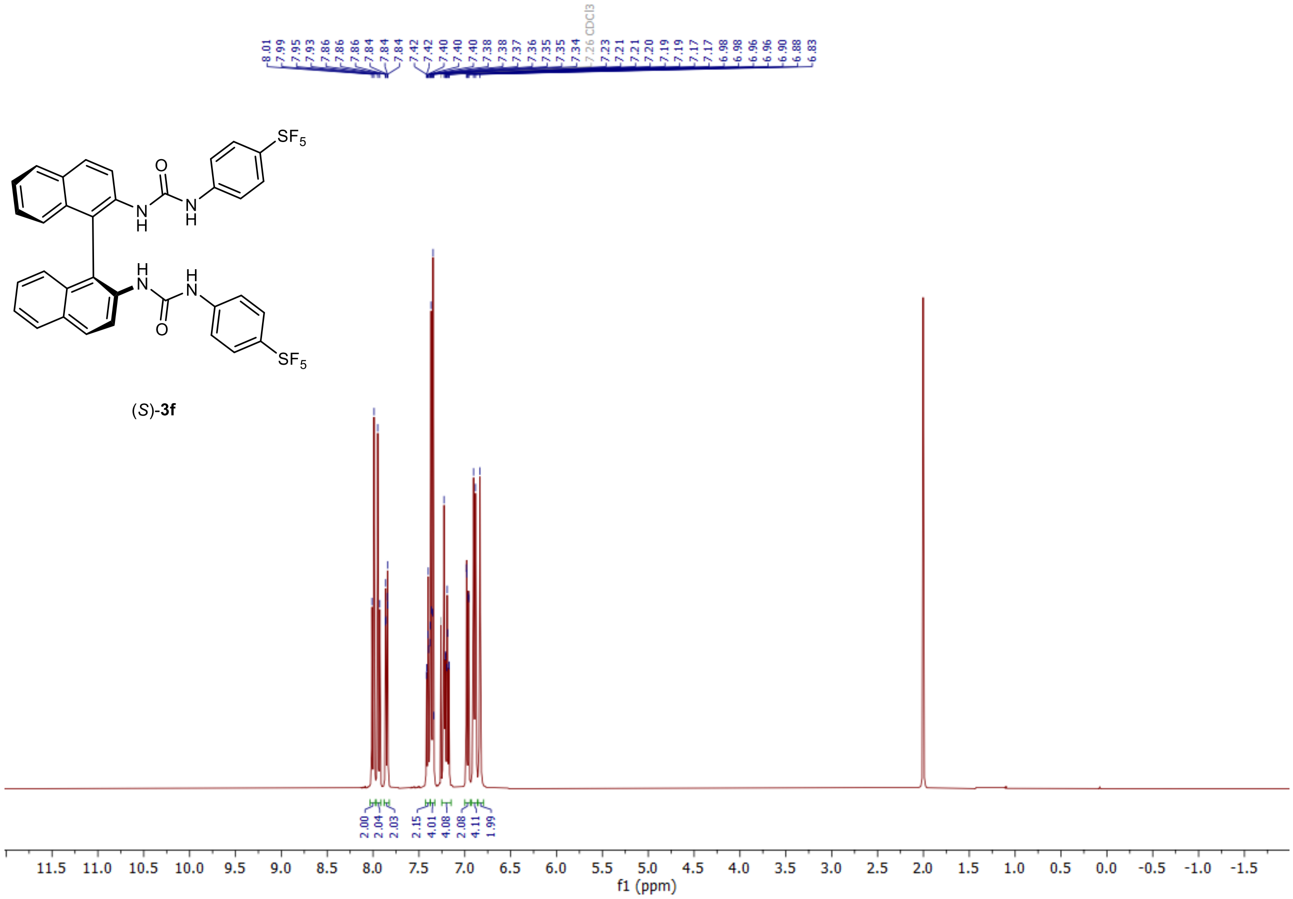

(*S*)-**3f** ^{13}C NMR (101 MHz, $CDCl_3$)

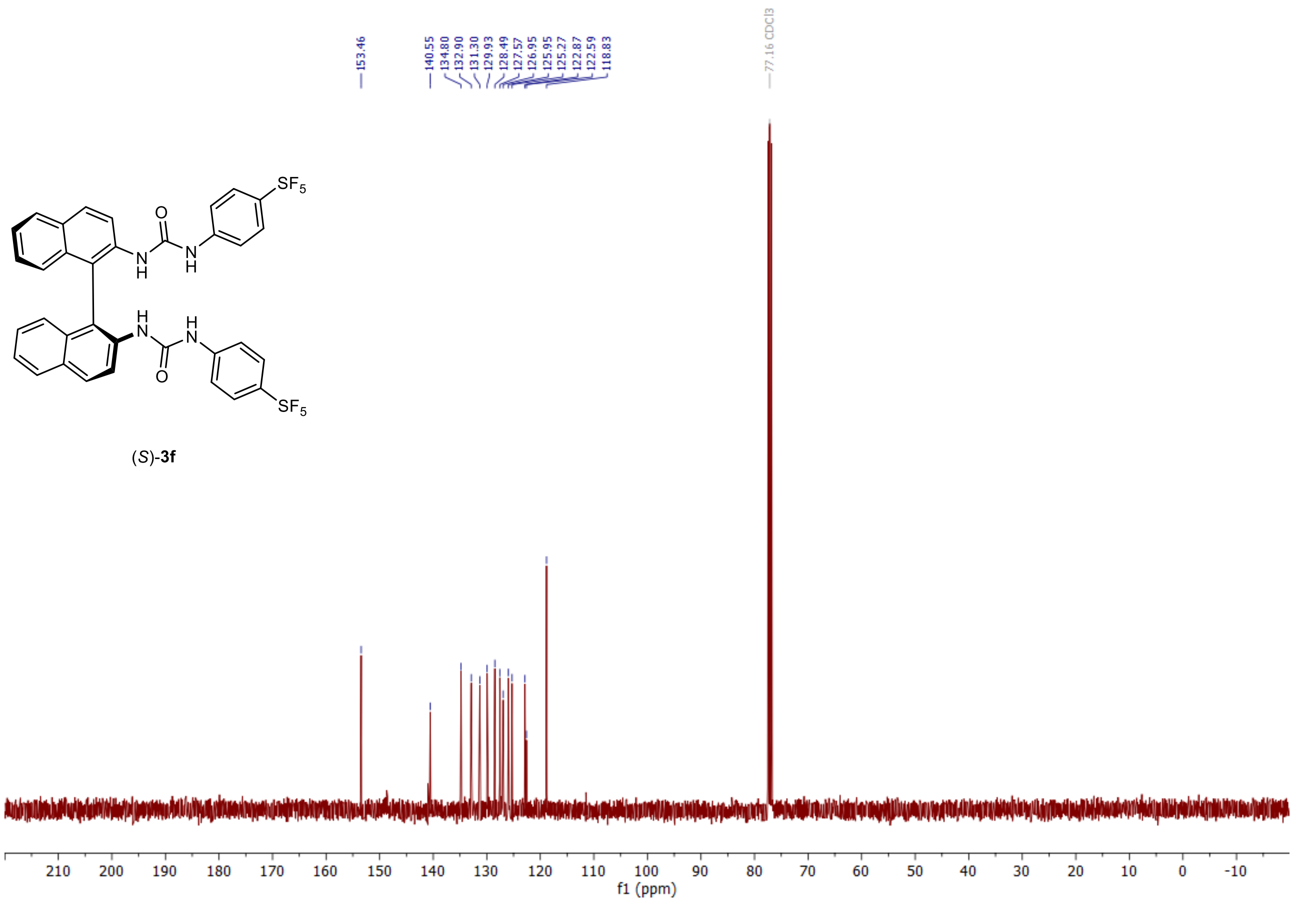

(*S*)-**3f** ^{19}F NMR (377 MHz, $CDCl_3$)

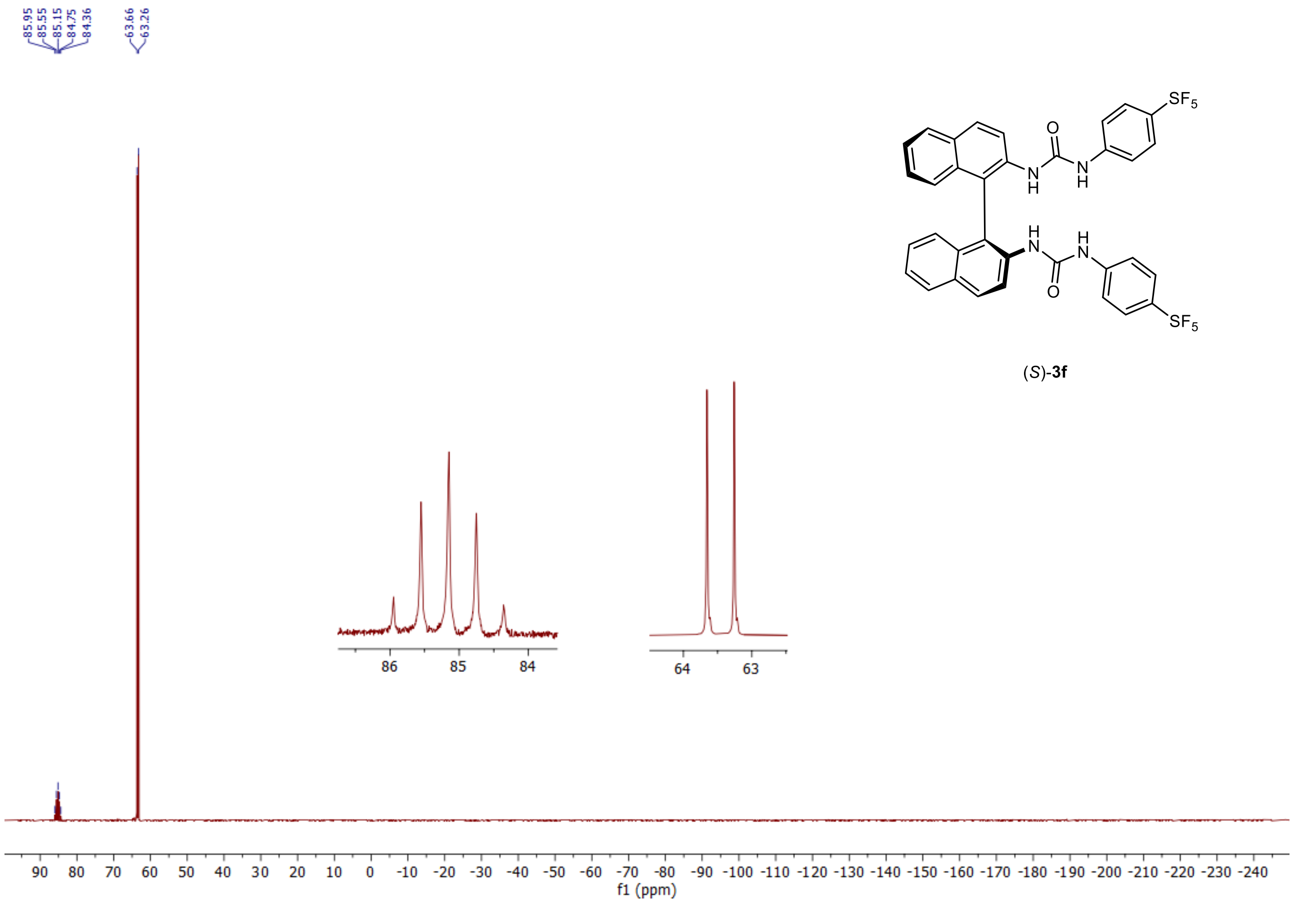

(*S*)-**3i** ^{1}H NMR (400 MHz, DMSO-d_6)

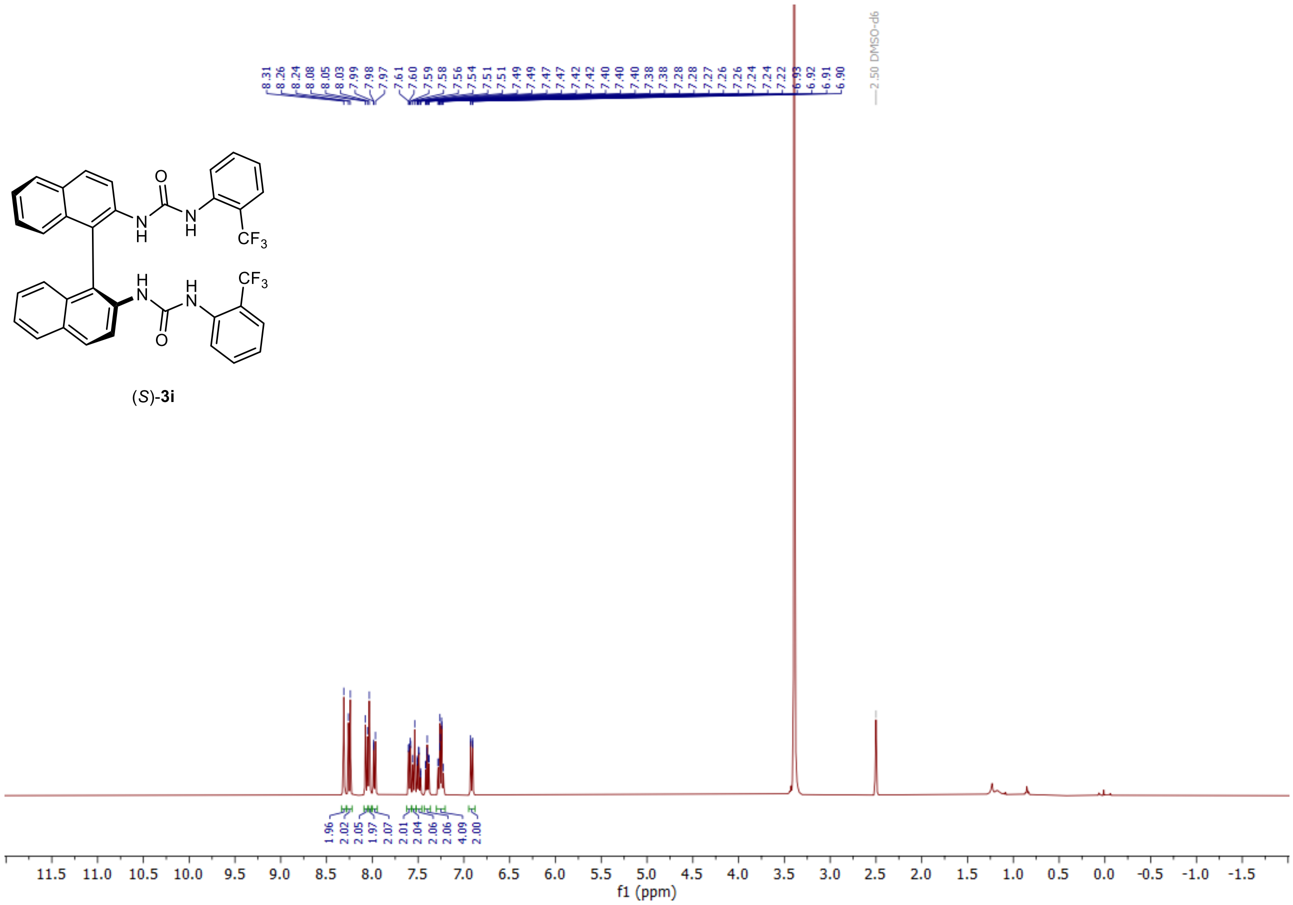

(*S*)-**3i** ^{13}C NMR (101 MHz, DMSO-d_6)

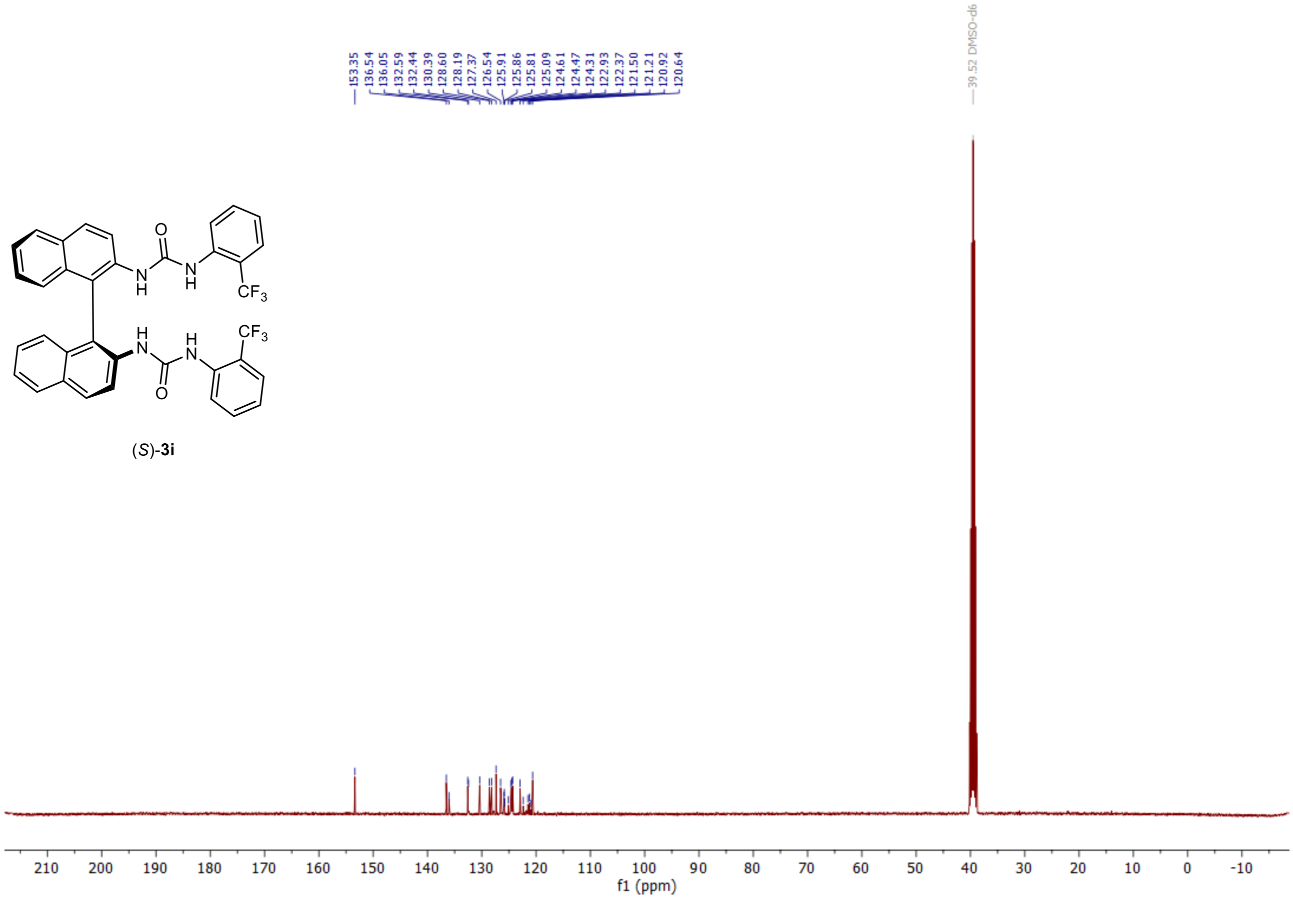

(*S*)-**3i** ^{19}F NMR (377 MHz, DMSO-d_6)

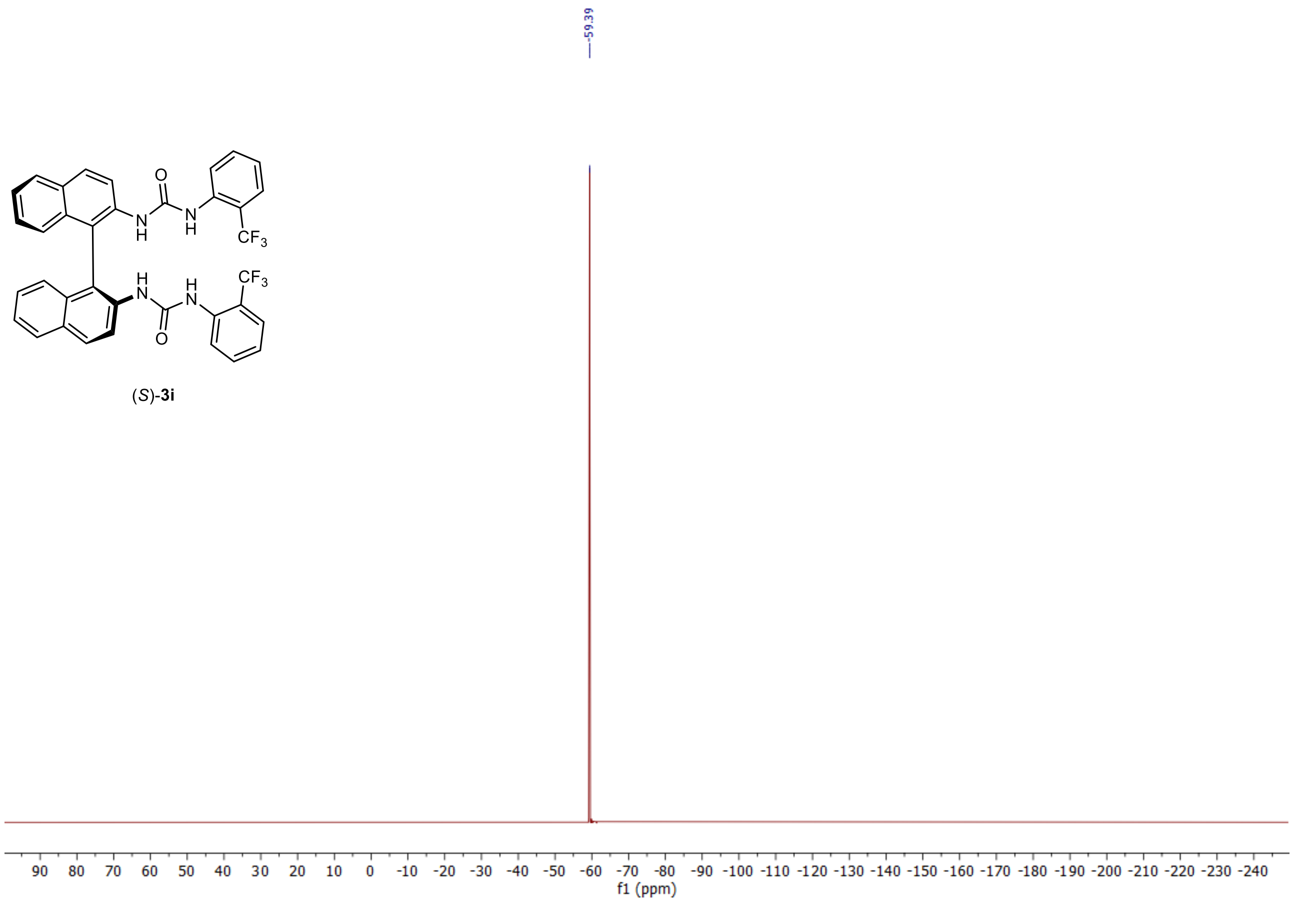

(*S*)-**3j** ^{1}H NMR (400 MHz, DMSO-d_6)

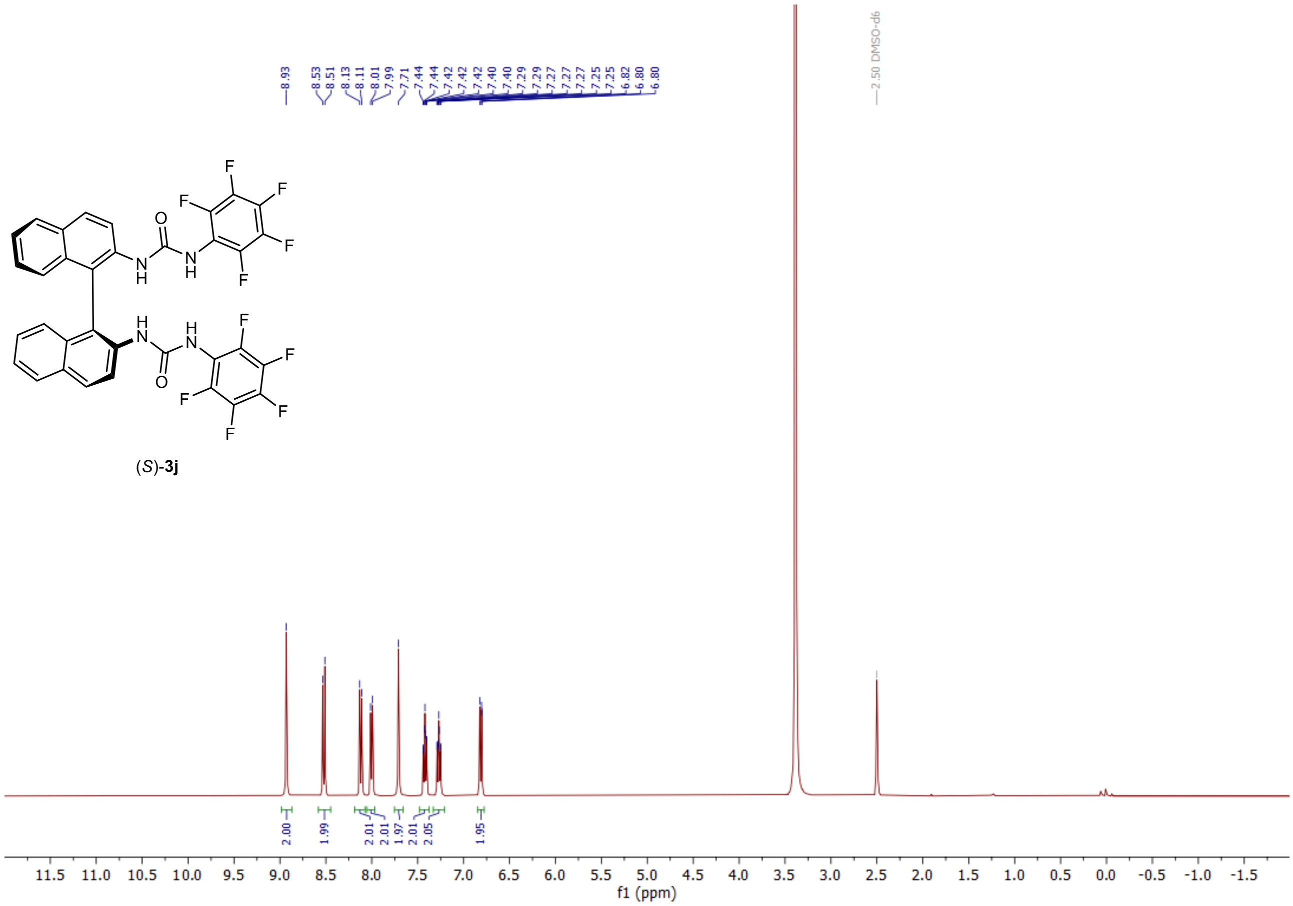

(*S*)-**3j** ^{13}C NMR (101 MHz, DMSO-d_6)

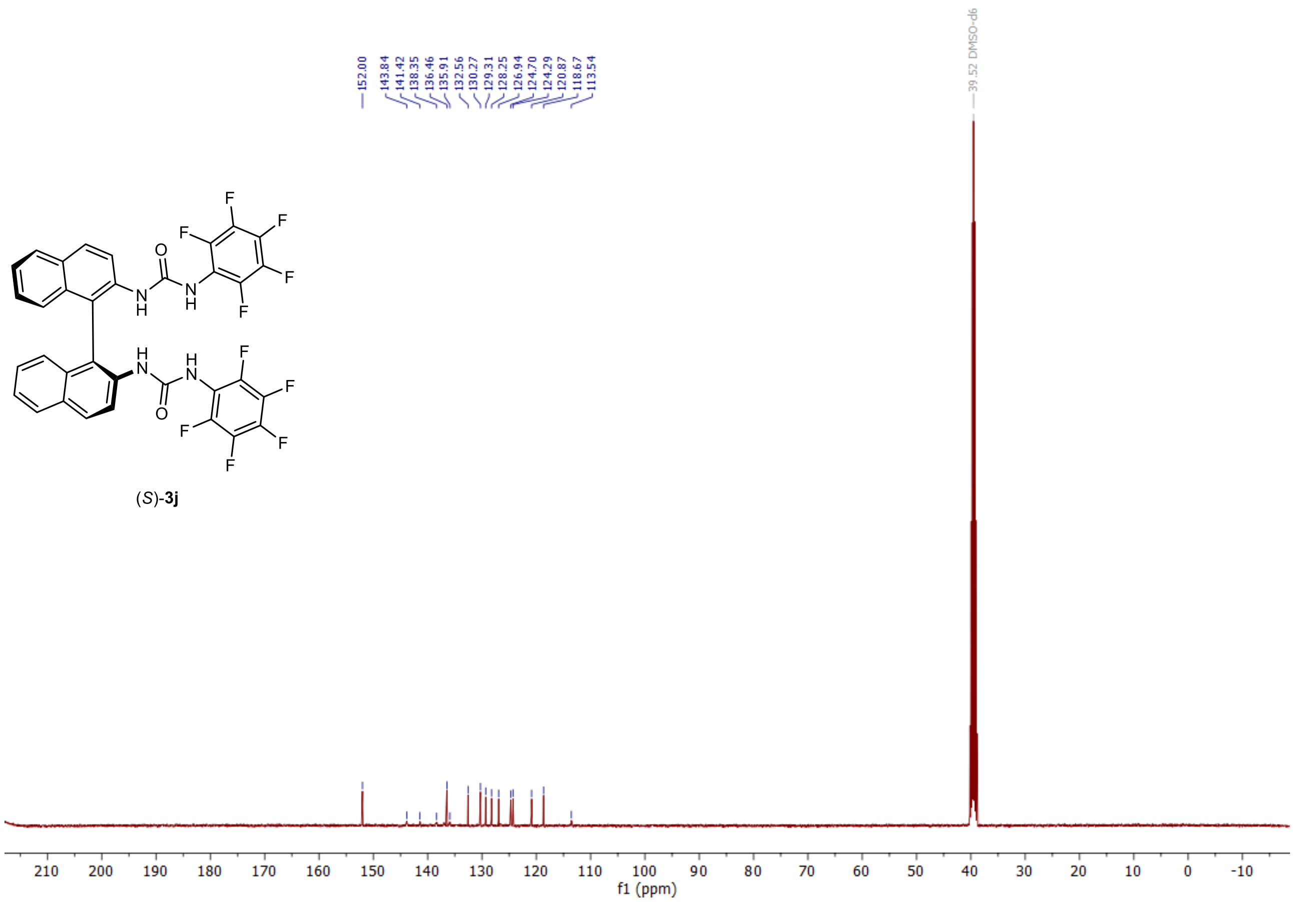

(*S*)-**3j** ^{19}F NMR (377 MHz, DMSO-d_6)

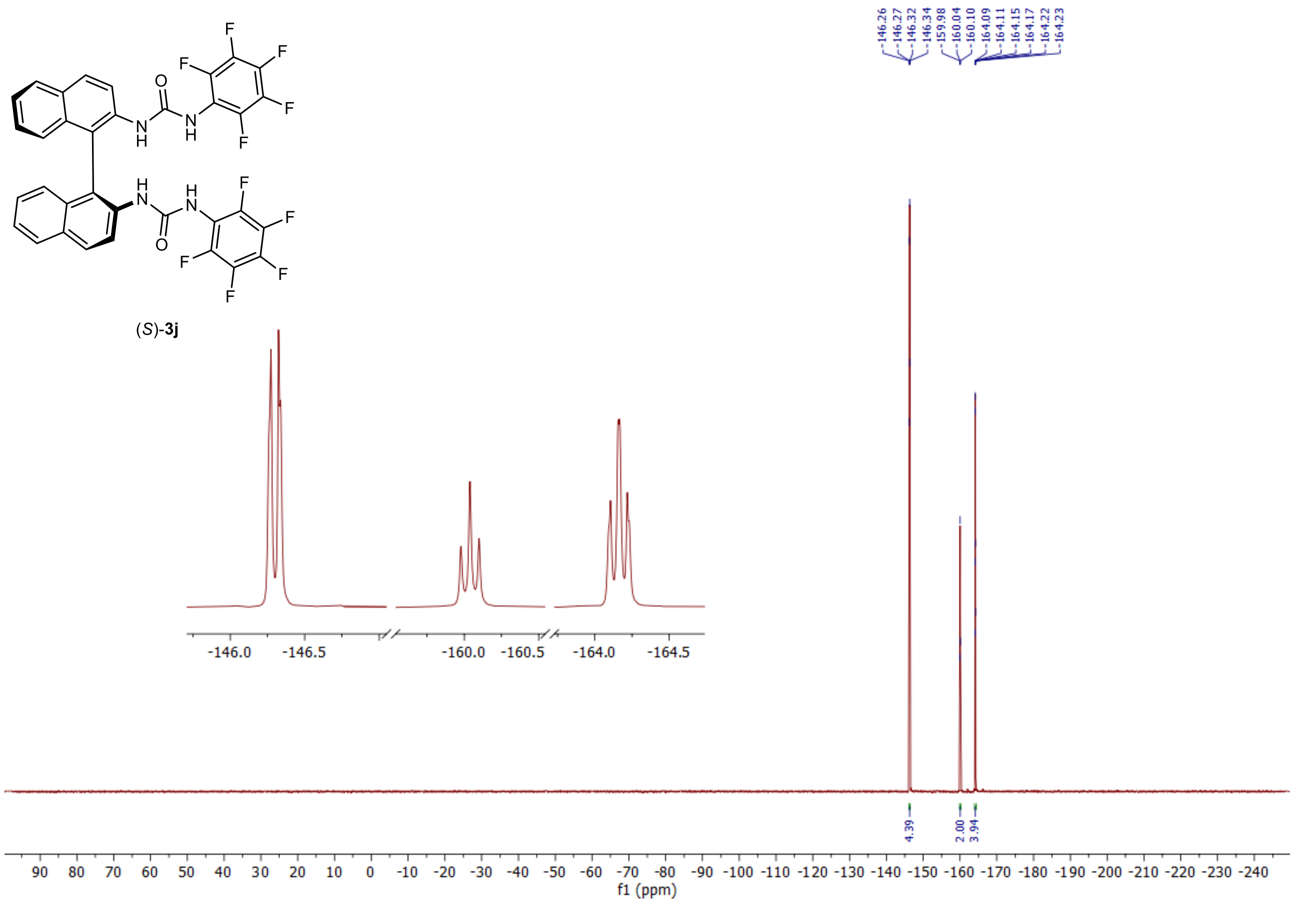

6b ^{1}H NMR (400 MHz, $CDCl_3$)

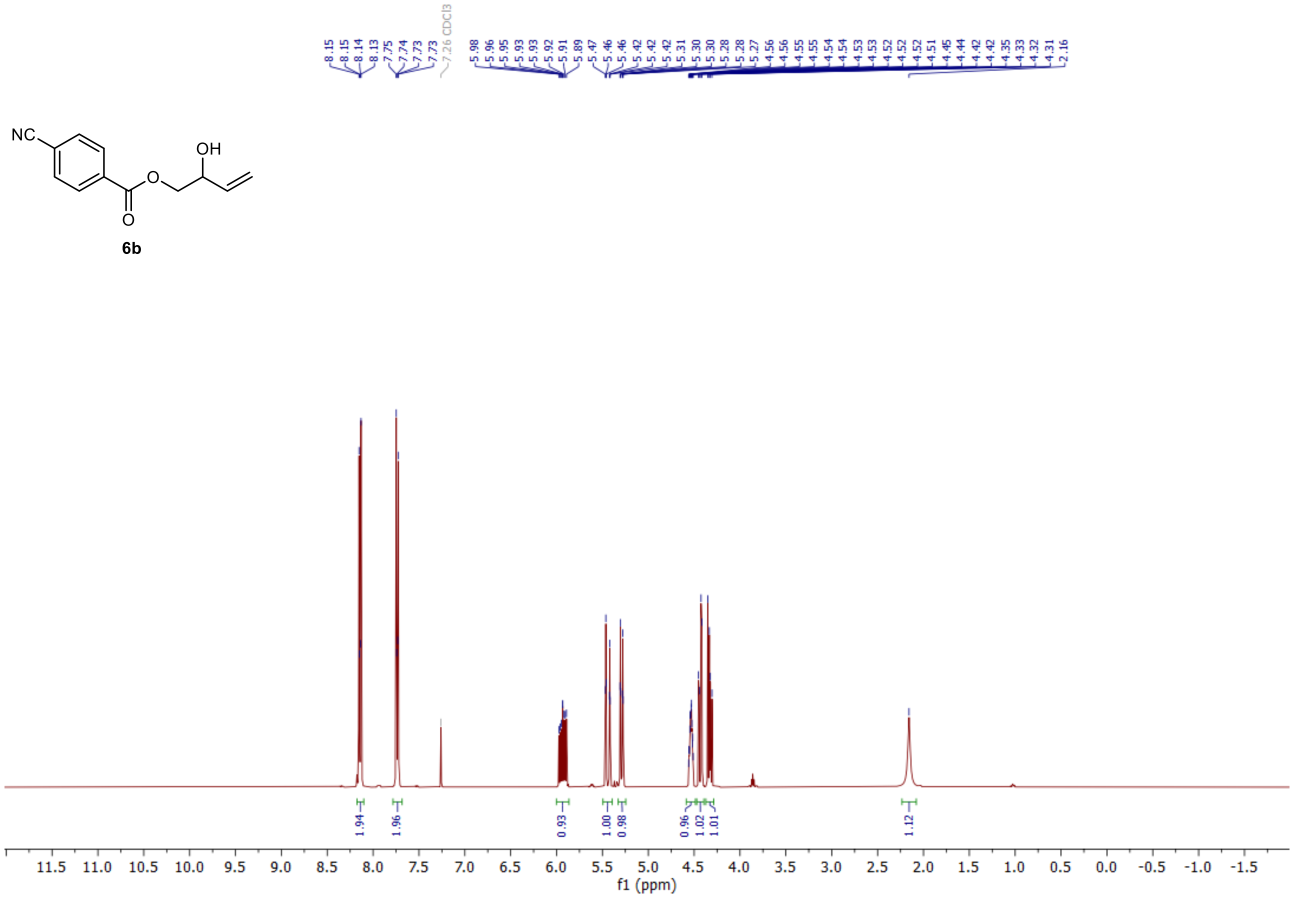

6b ^{13}C NMR (101 MHz, $CDCl_3$)

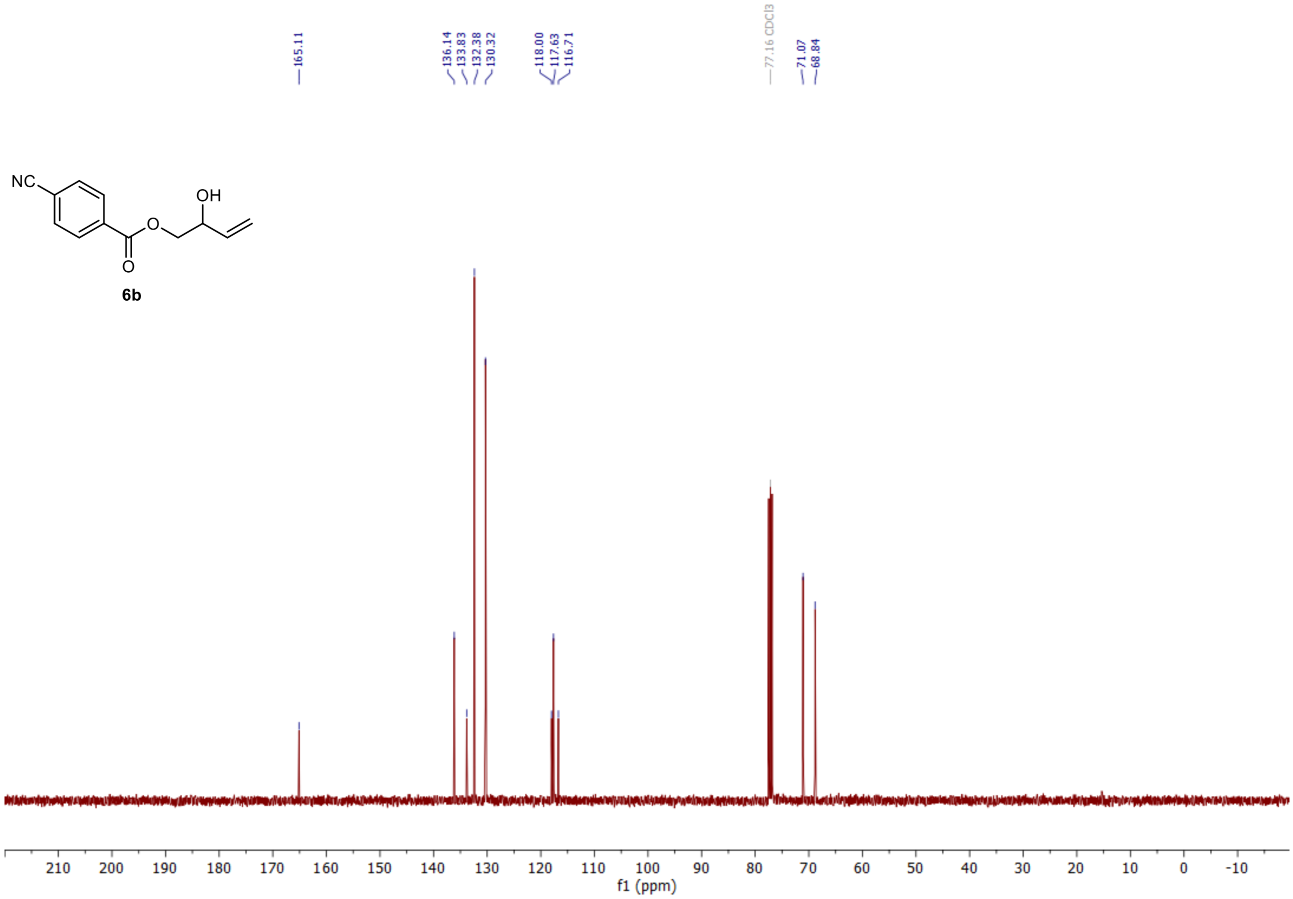

6d ^{1}H NMR (400 MHz, $CDCl_3$)

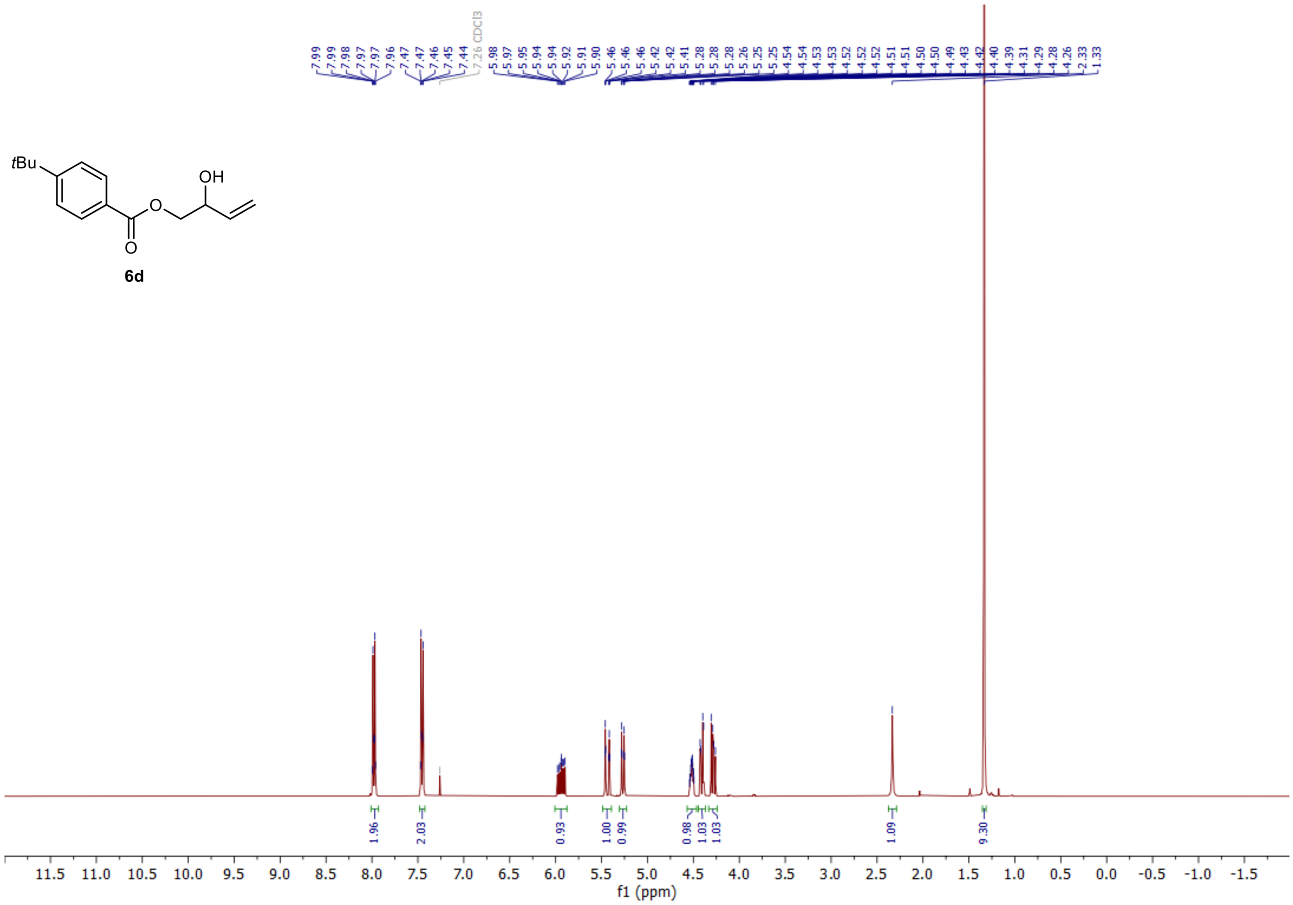

6d ^{13}C NMR (101 MHz, $CDCl_3$)

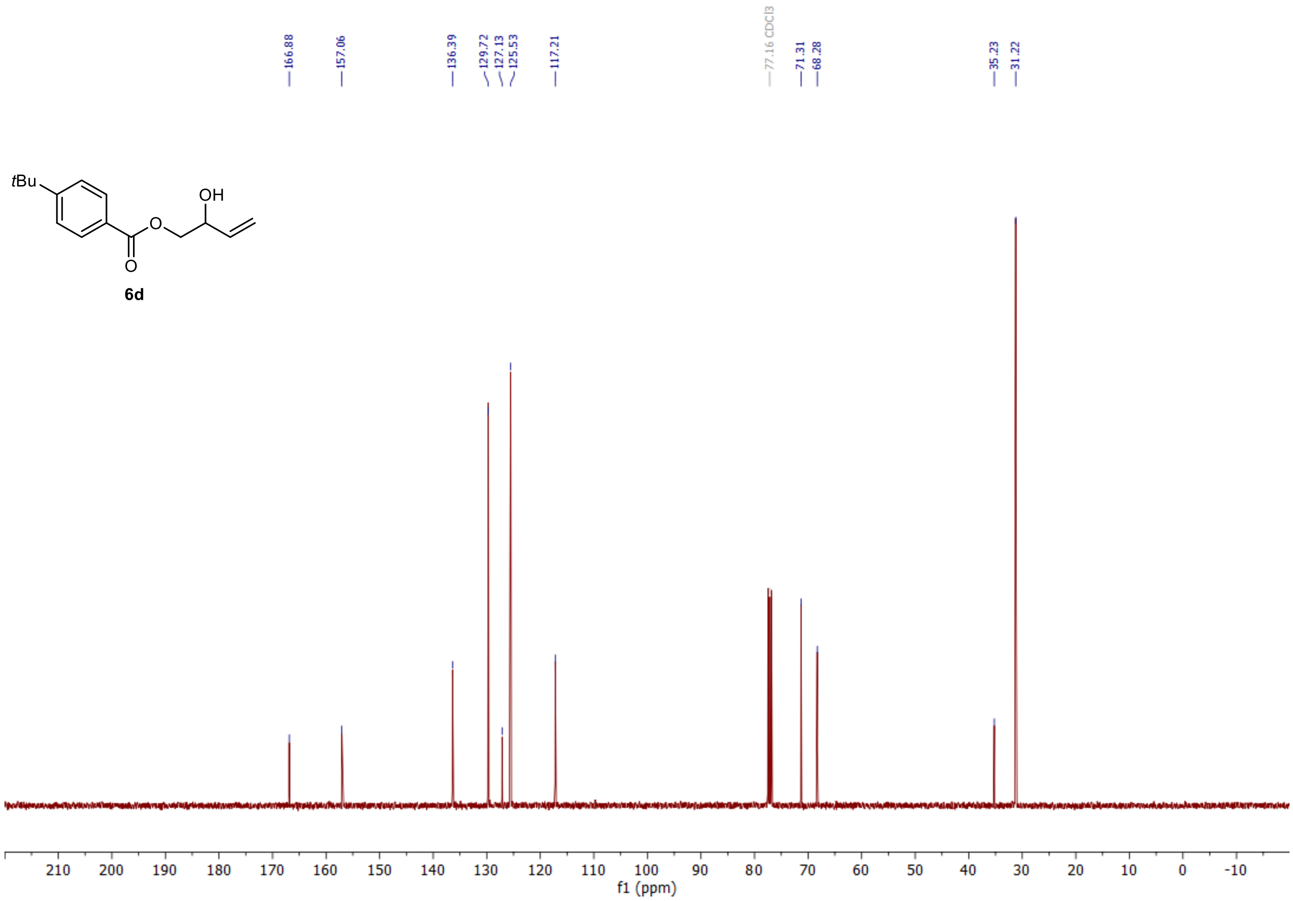

6e ^{1}H NMR (400 MHz, $CDCl_3$)

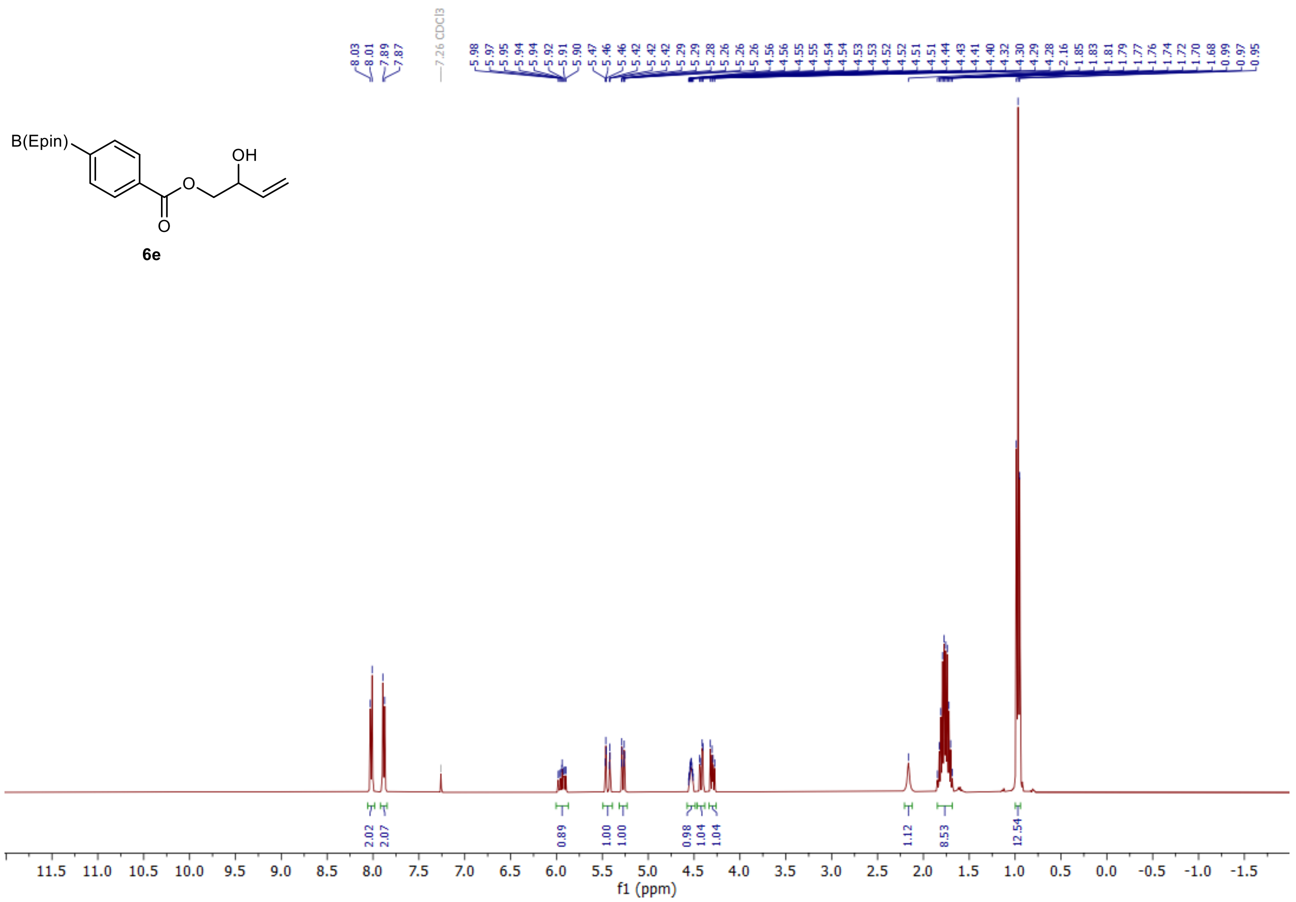

6e ^{13}C NMR (101 MHz, $CDCl_3$)

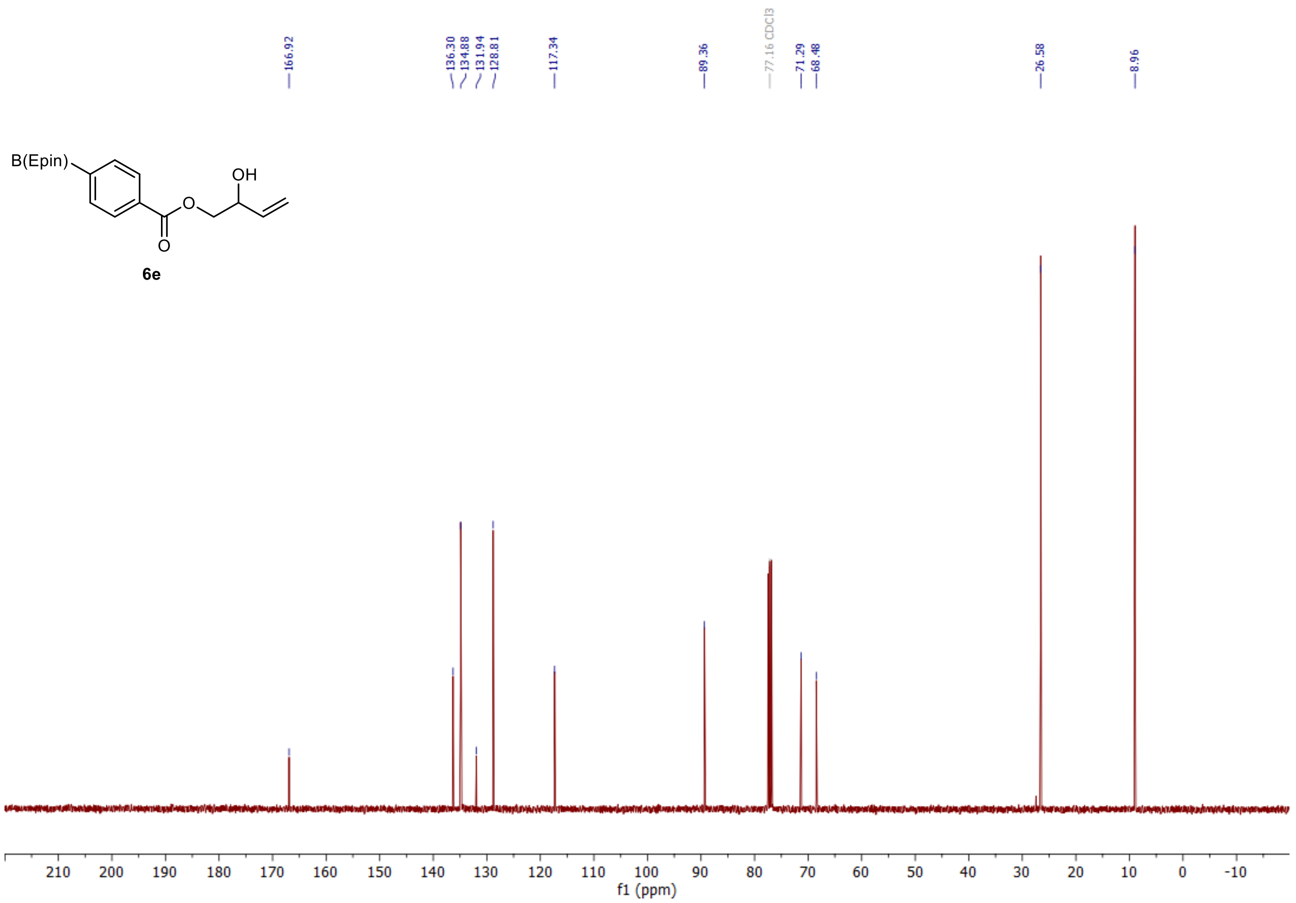

6f ^{1}H NMR (400 MHz, $CDCl_3$)

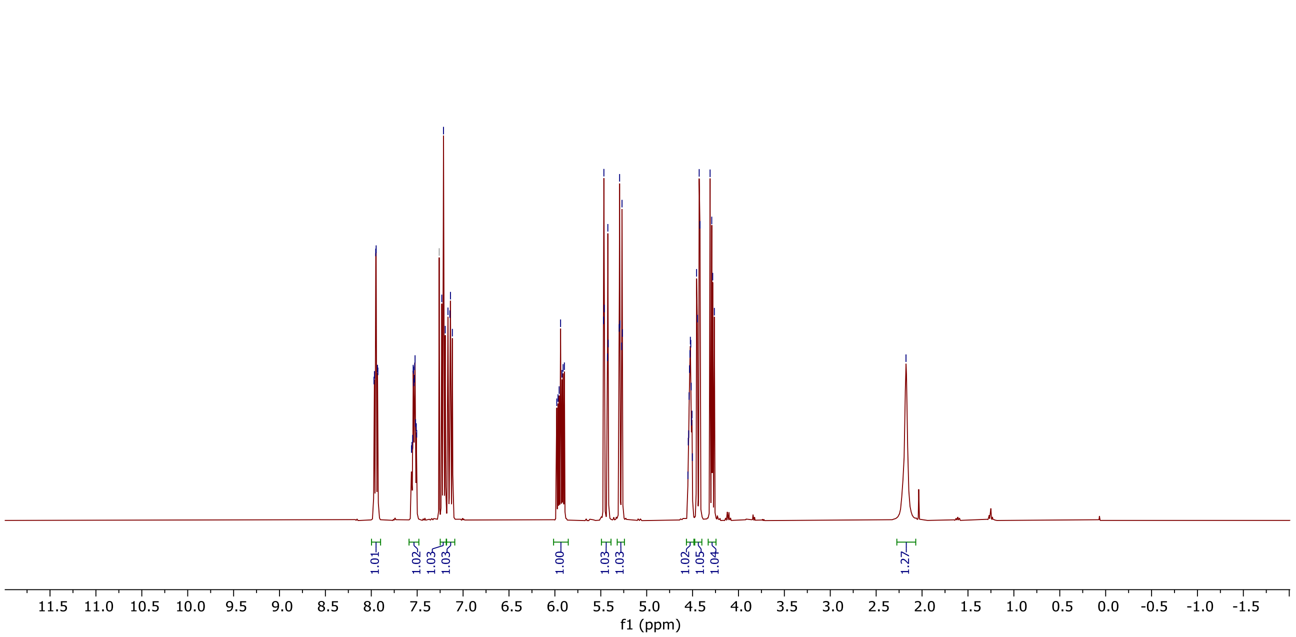

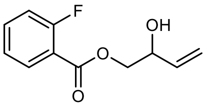

6f

6f ^{13}C NMR (101 MHz, $CDCl_3$)

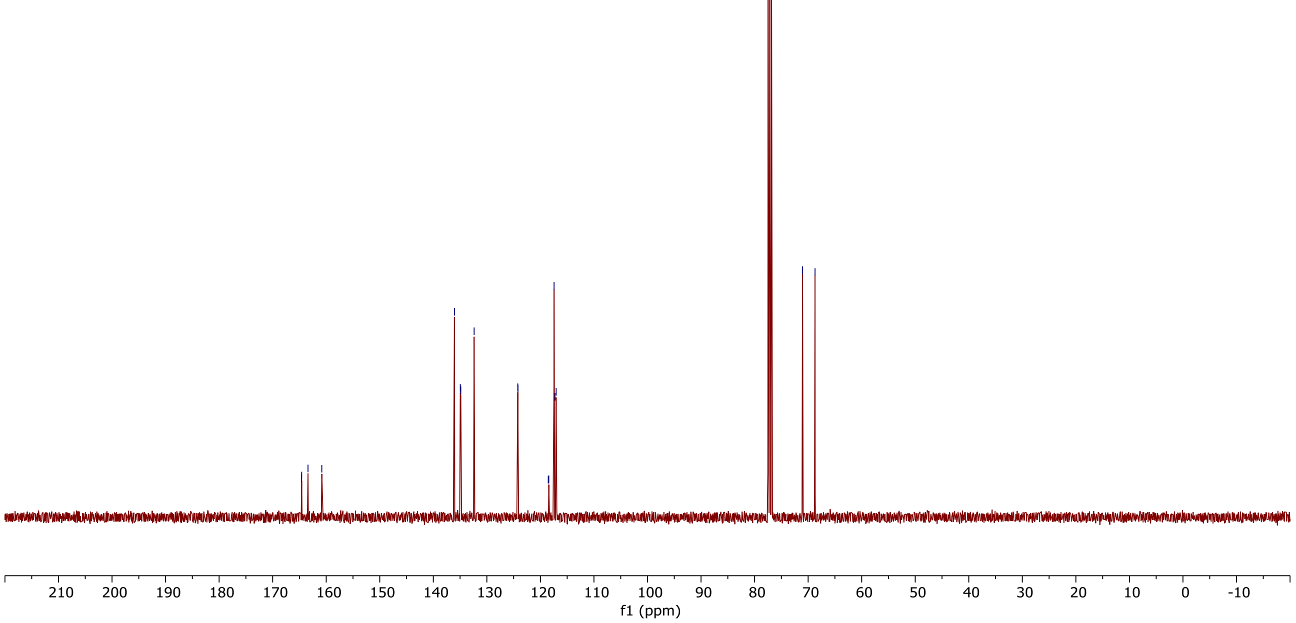

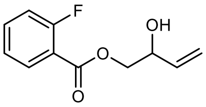

6f

6f ^{19}F NMR (377 MHz, $CDCl_3$)

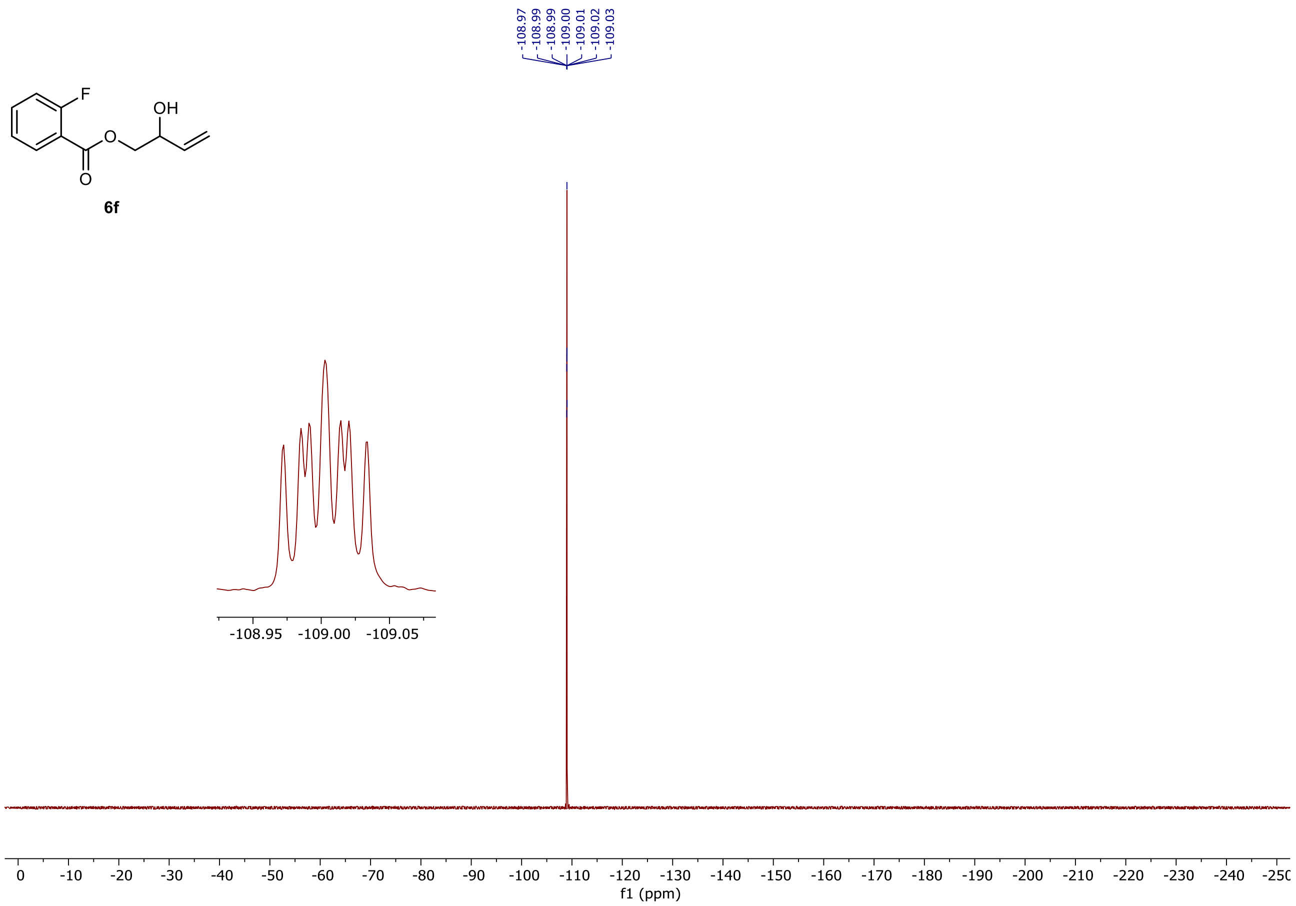

6g ^{1}H NMR (400 MHz, $CDCl_3$)

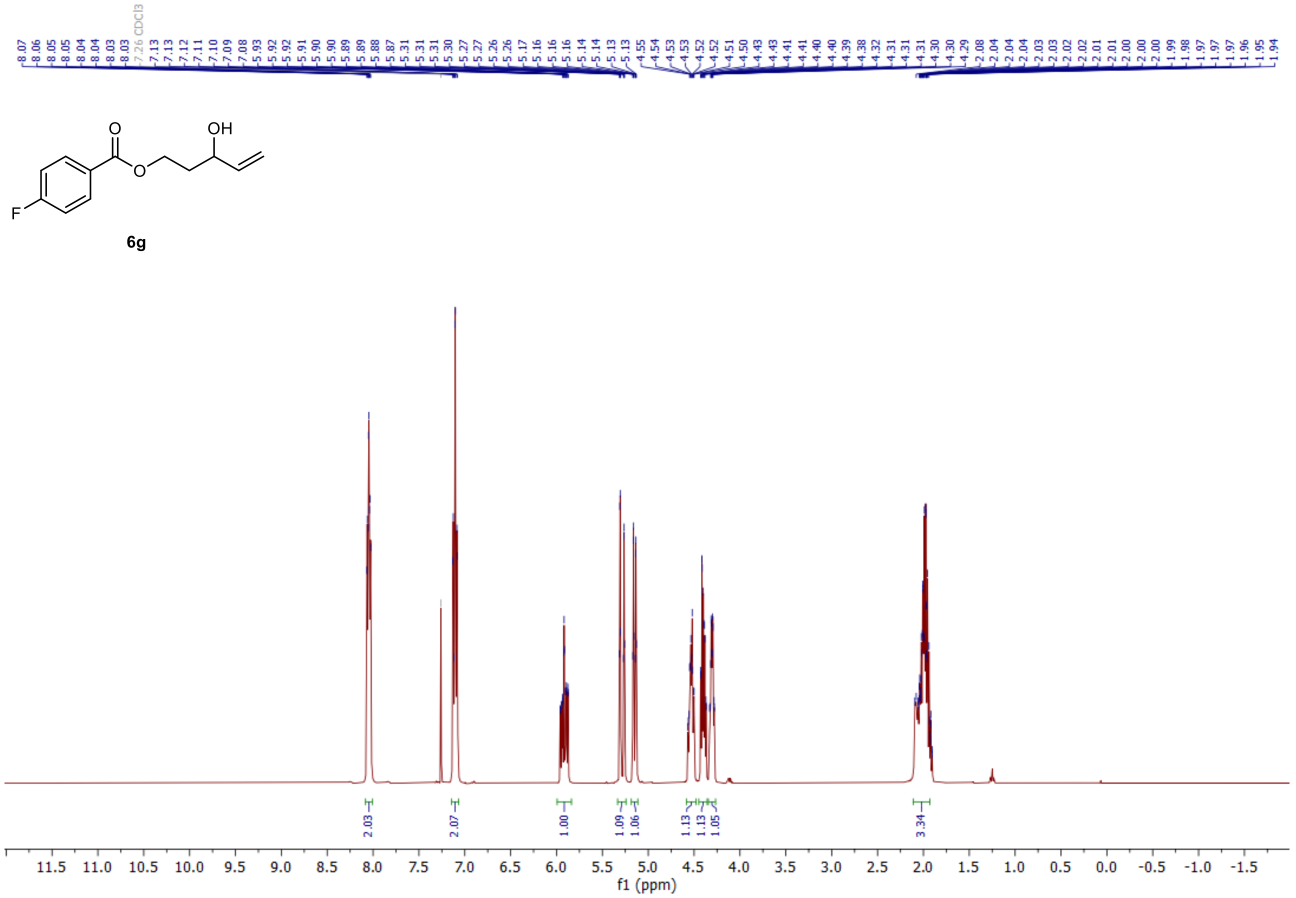

6g ^{13}C NMR (101 MHz, $CDCl_3$)

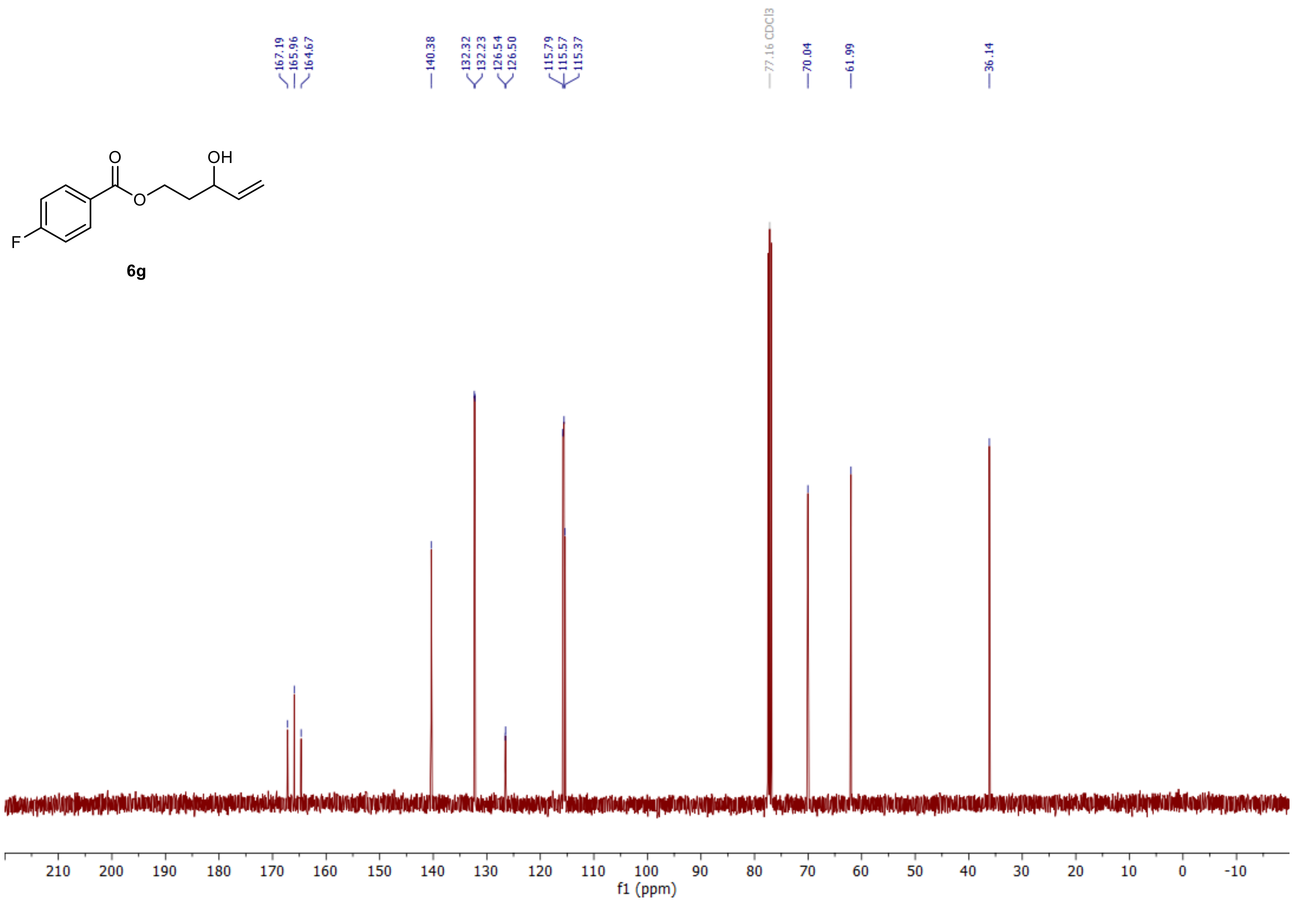

6g ^{19}F NMR (377 MHz, $CDCl_3$)

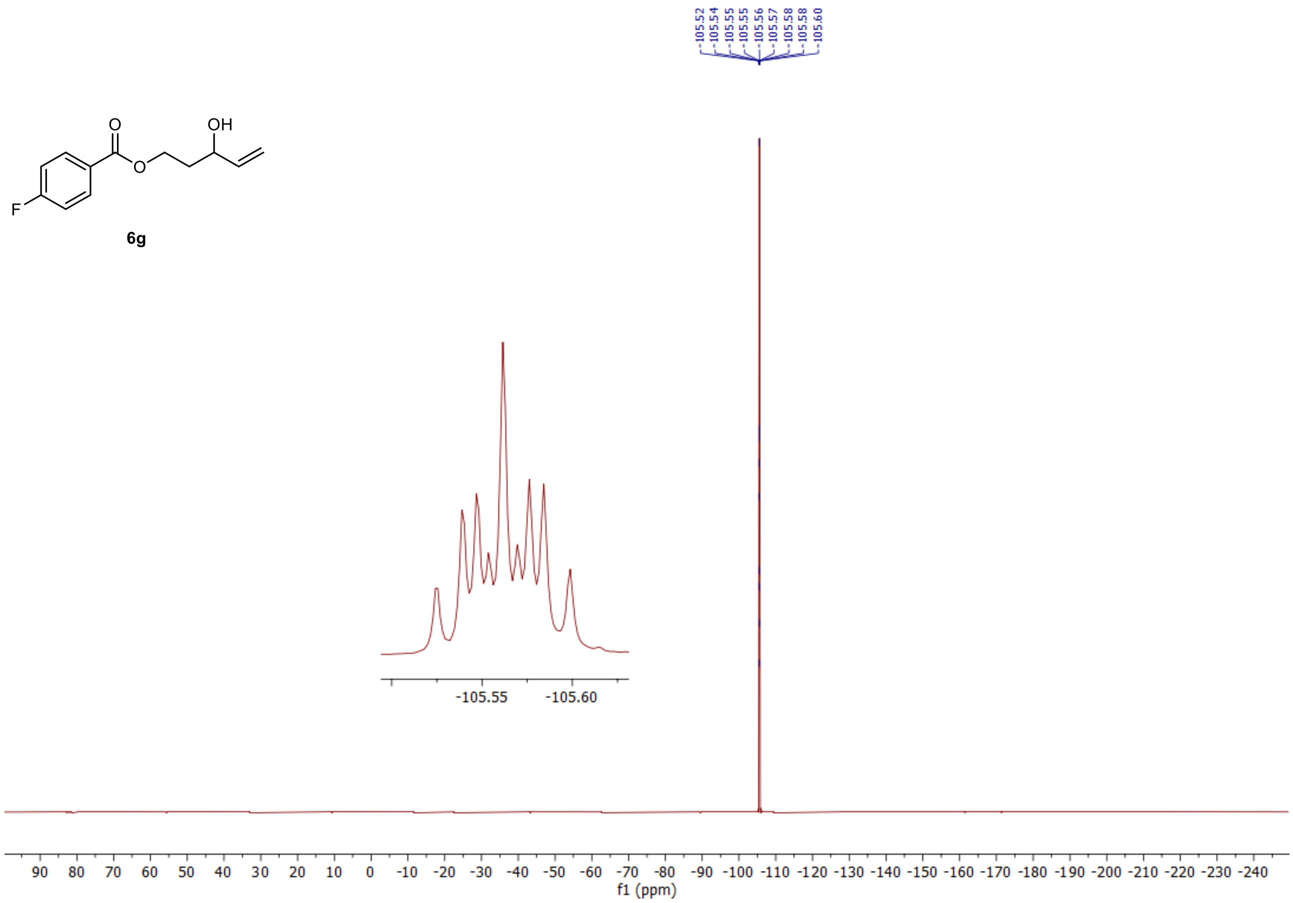

6h ^{1}H NMR (400 MHz, $CDCl_3$)

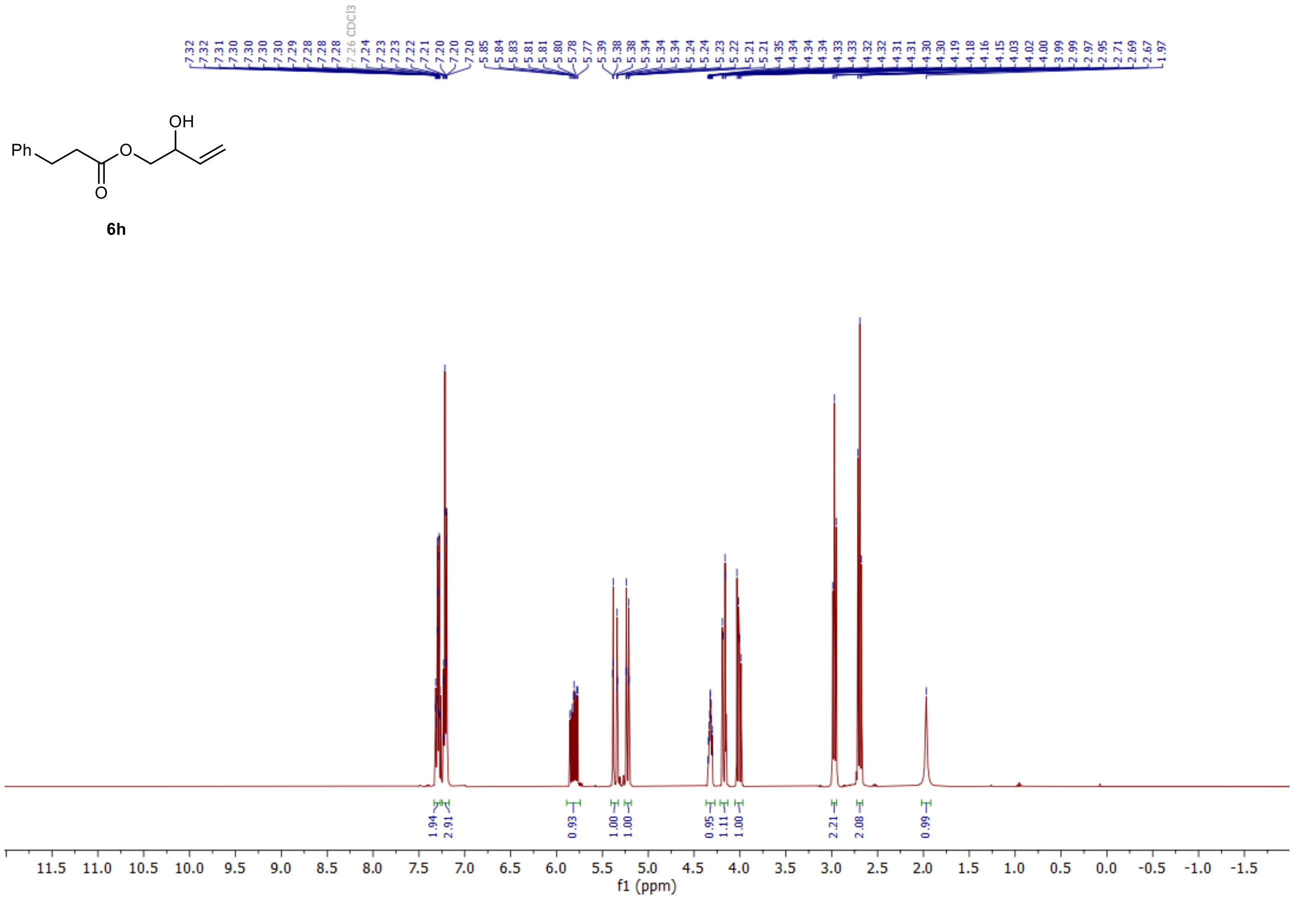

6h ^{13}C NMR (101 MHz, $CDCl_3$)

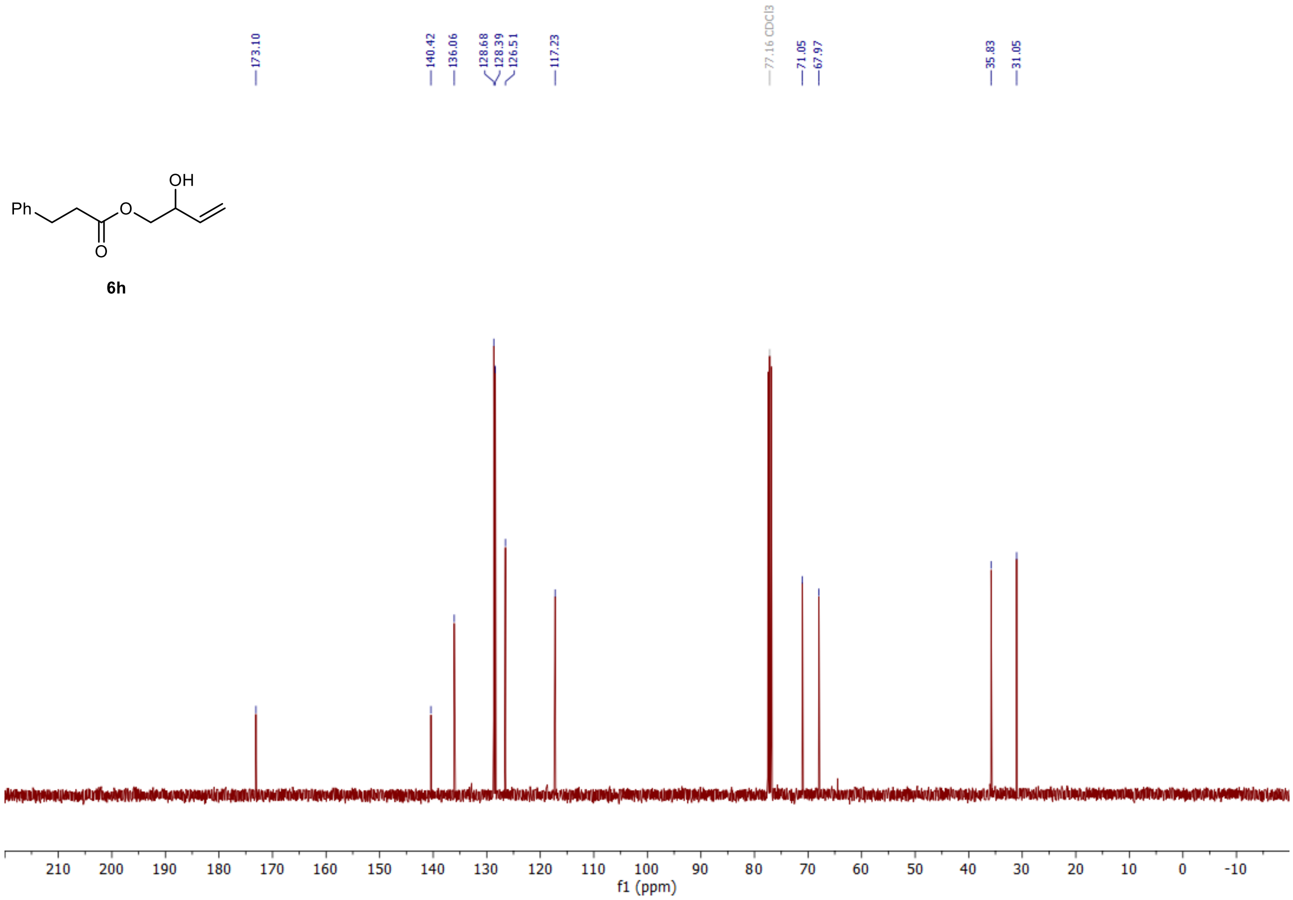

6i ^{1}H NMR (400 MHz, $CDCl_3$)

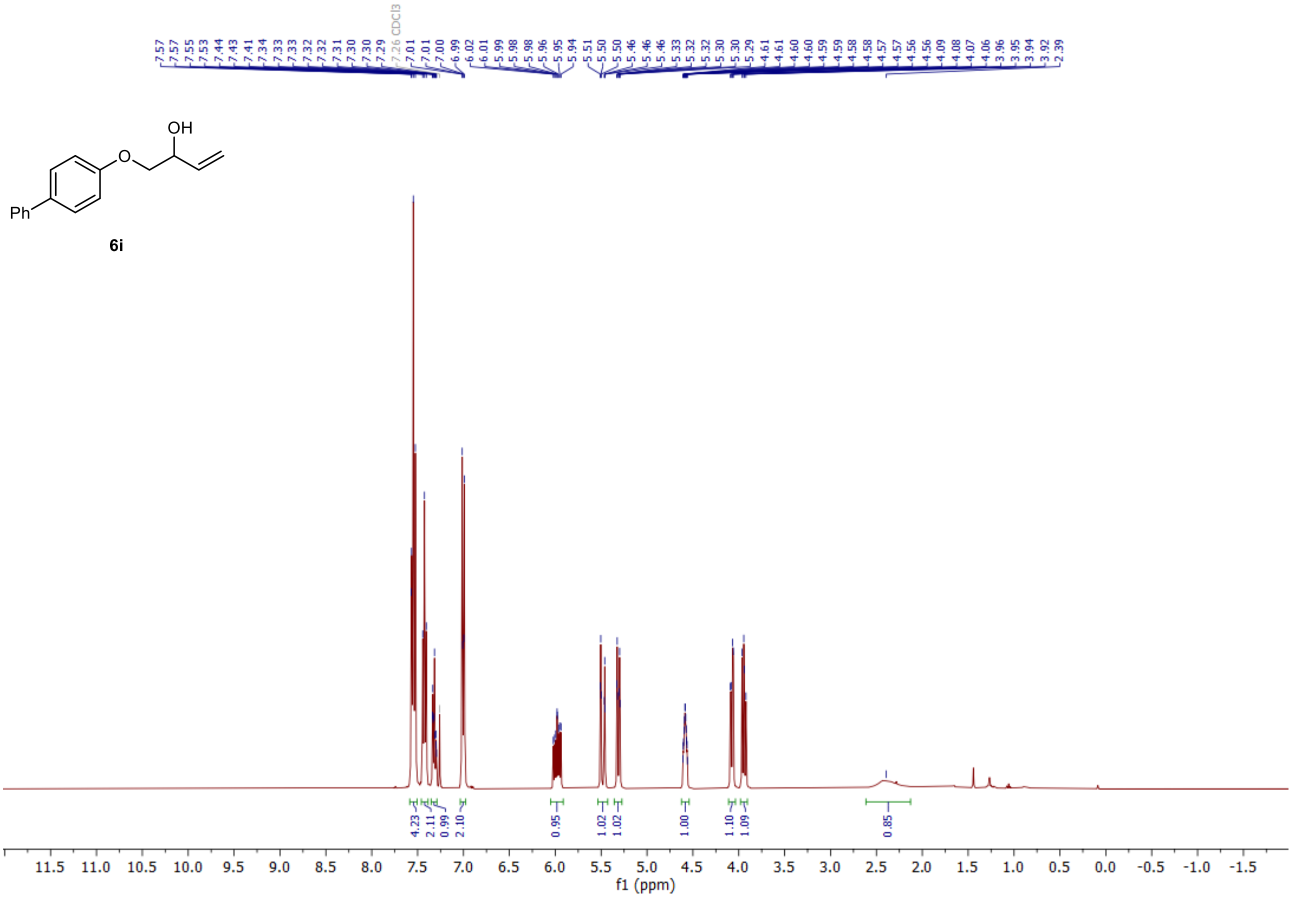

6i ^{13}C NMR (101 MHz, $CDCl_3$)

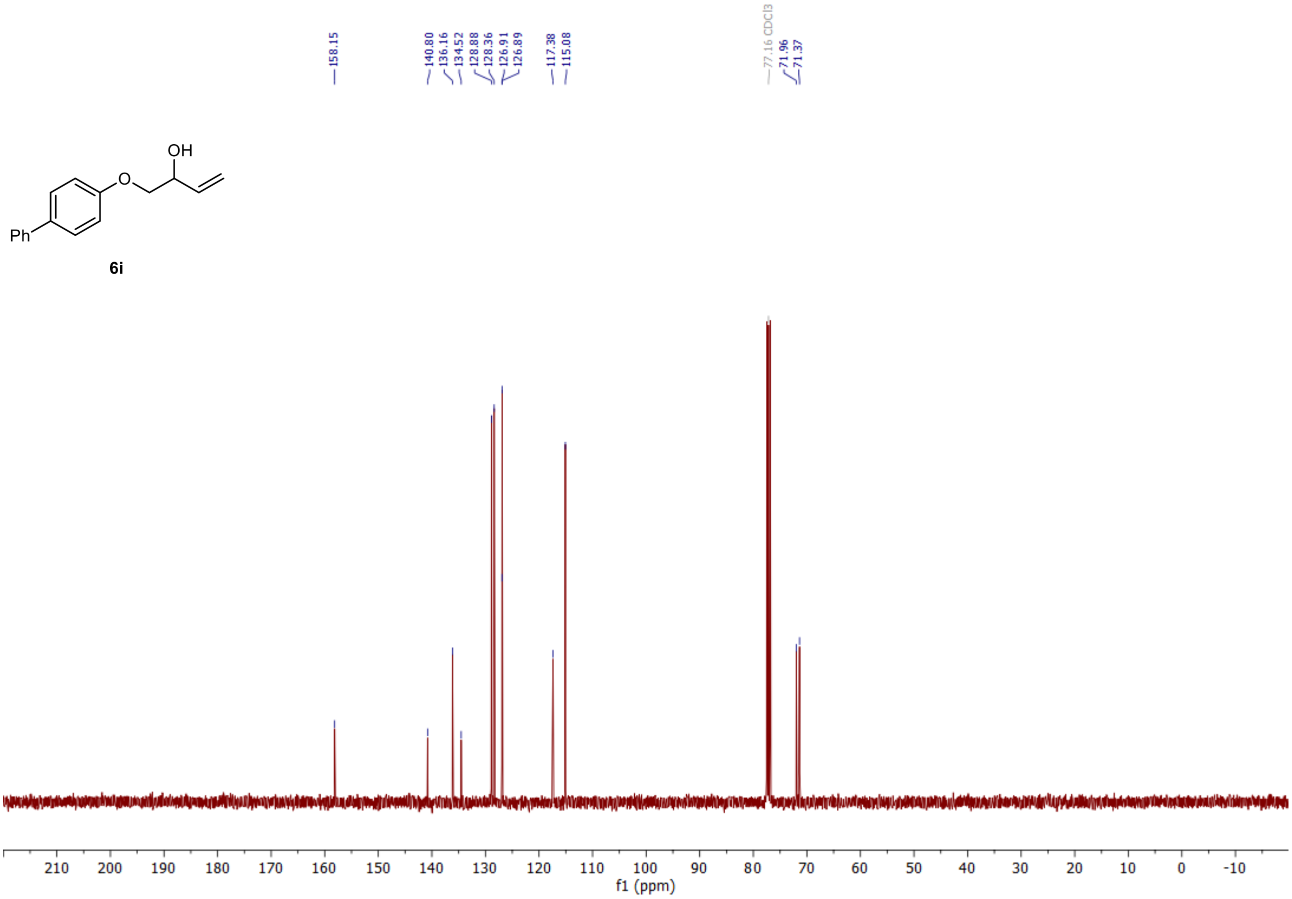

6m ^{1}H NMR (400 MHz, $CDCl_3$)

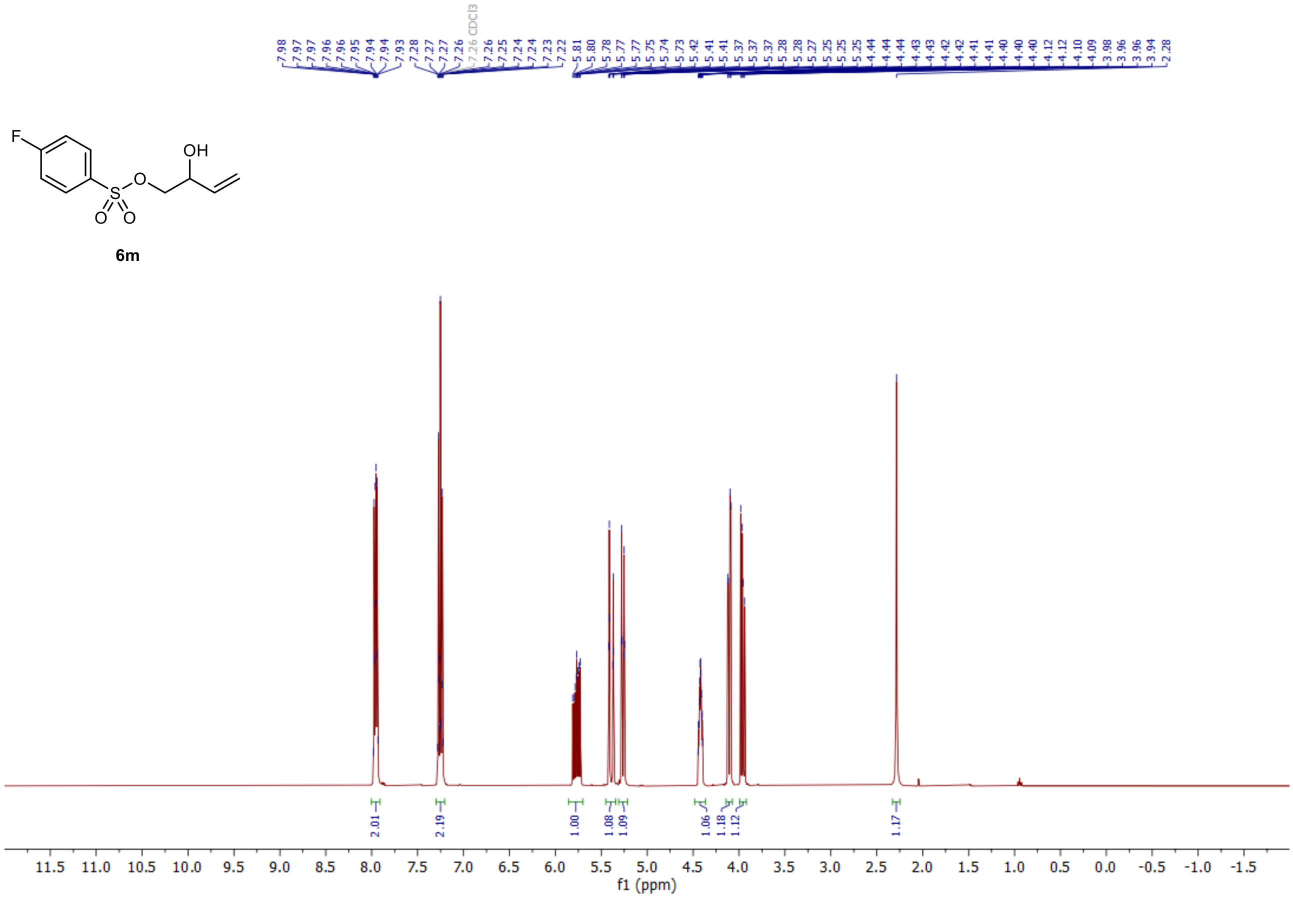

6m ^{13}C NMR (101 MHz, $CDCl_3$)

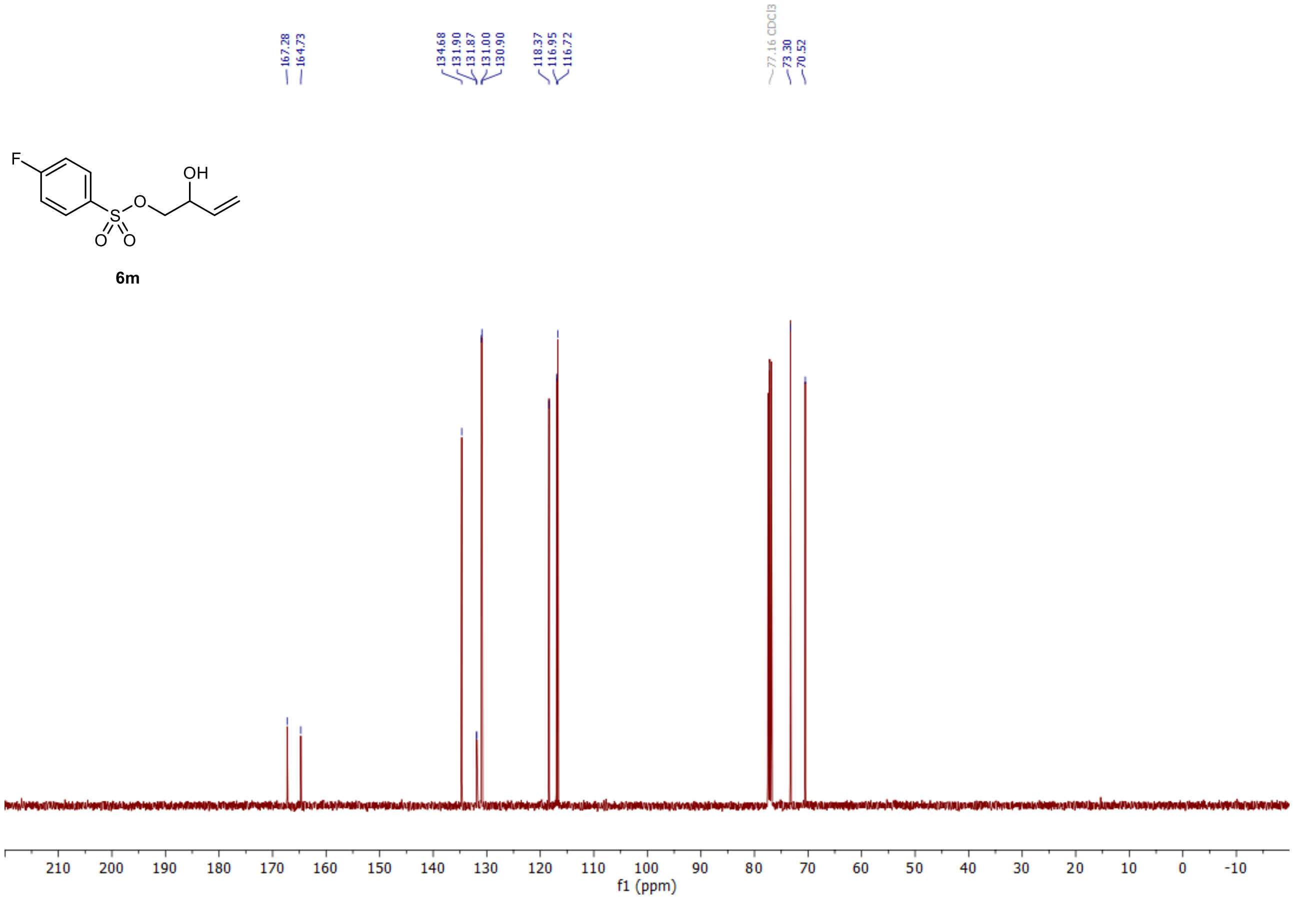

6m ^{19}F NMR (377 MHz, $CDCl_3$)

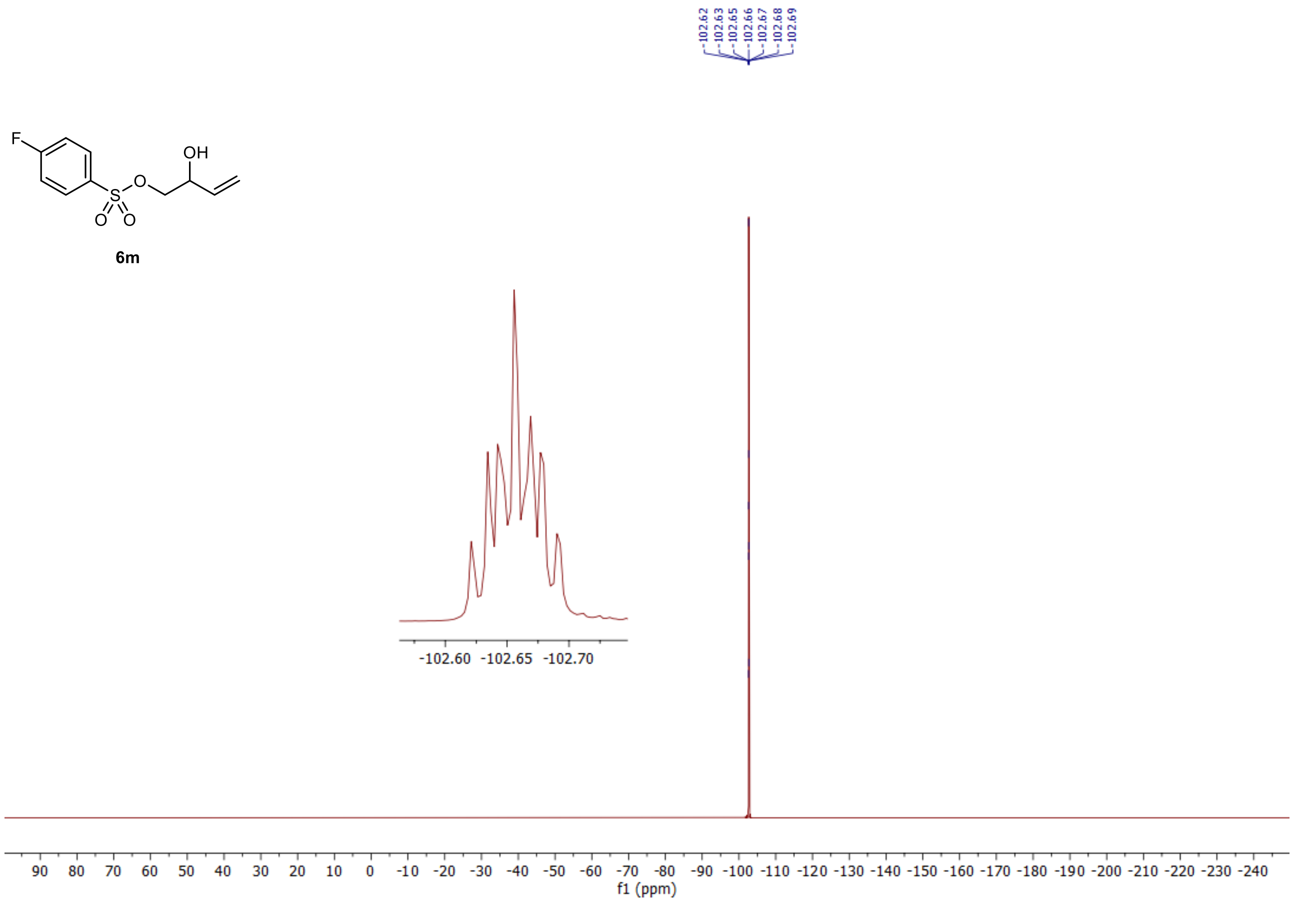

6n ^{1}H NMR (400 MHz, $CDCl_3$)

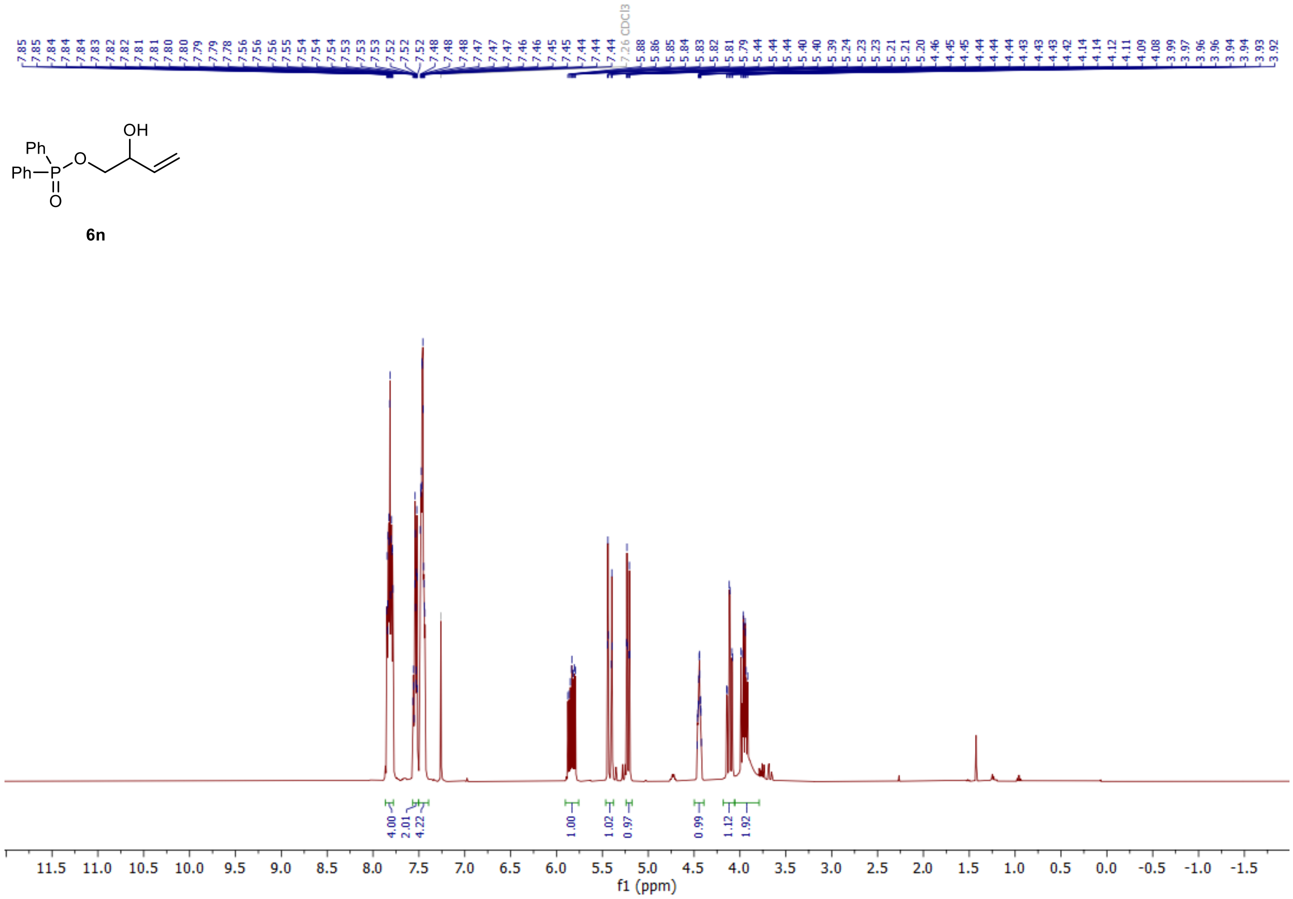

6n ^{13}C NMR (101 MHz, $CDCl_3$)

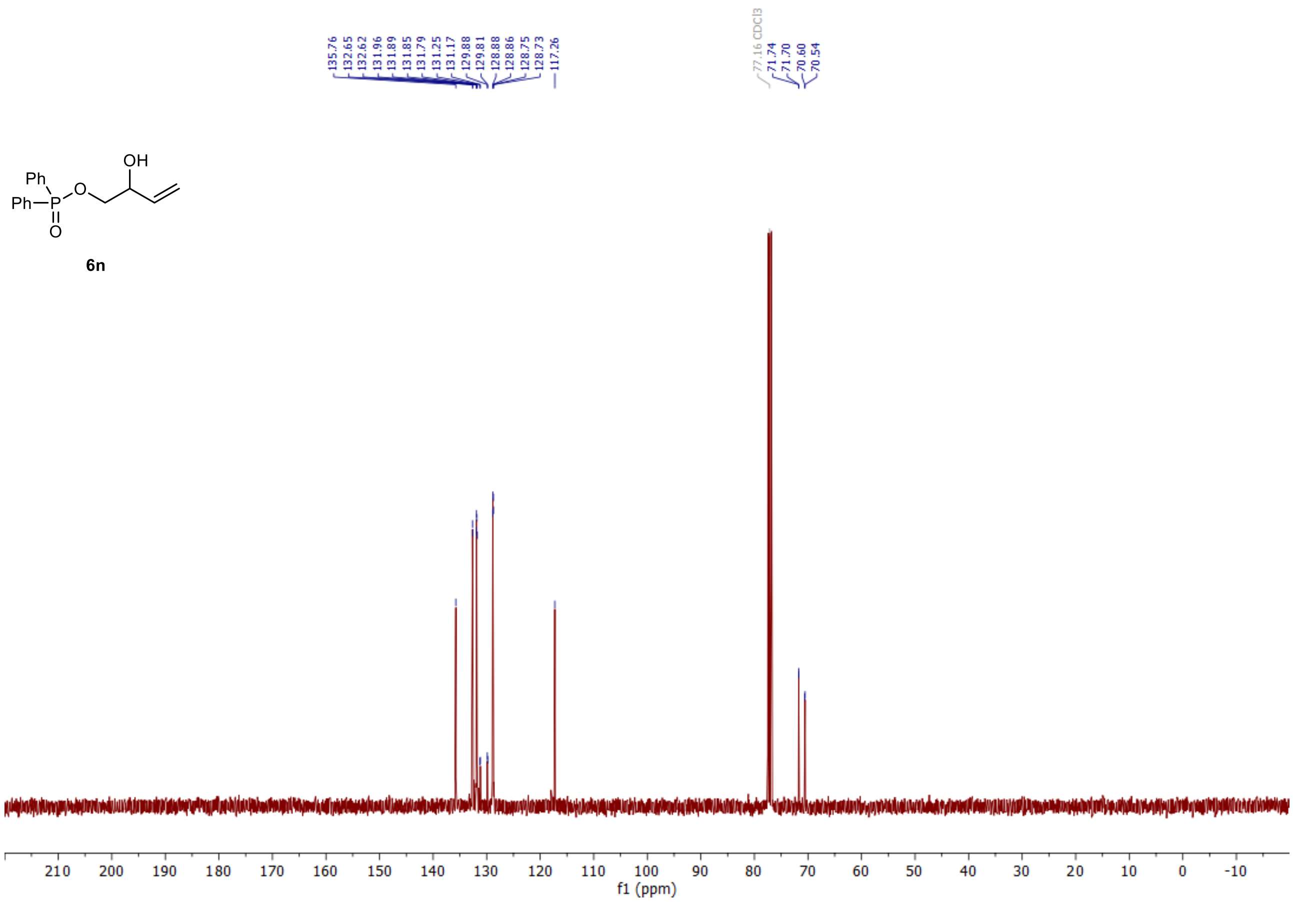

6n ^{31}P NMR (162 MHz, $CDCl_3$)

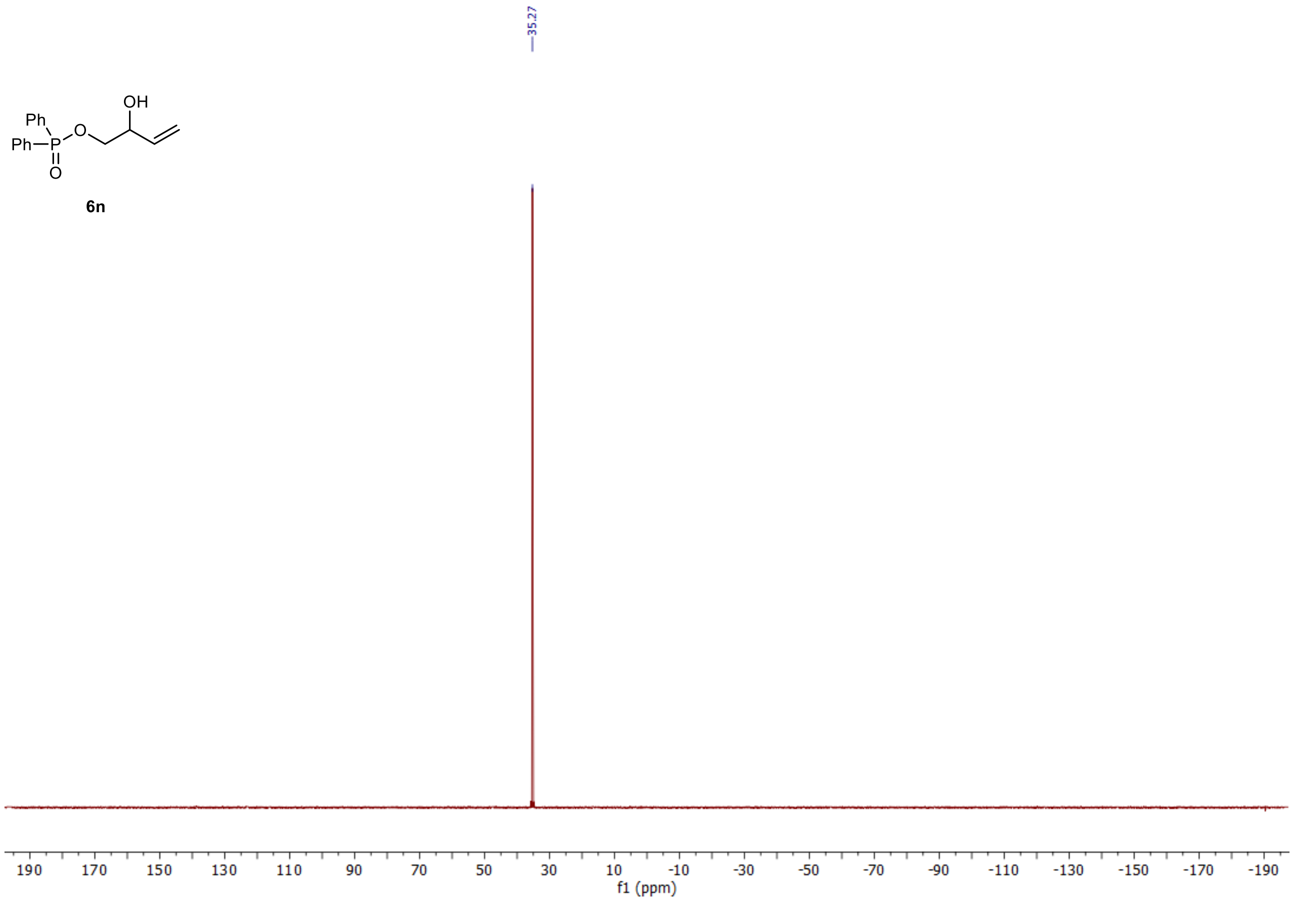

6p ^{1}H NMR (400 MHz, $CDCl_3$)

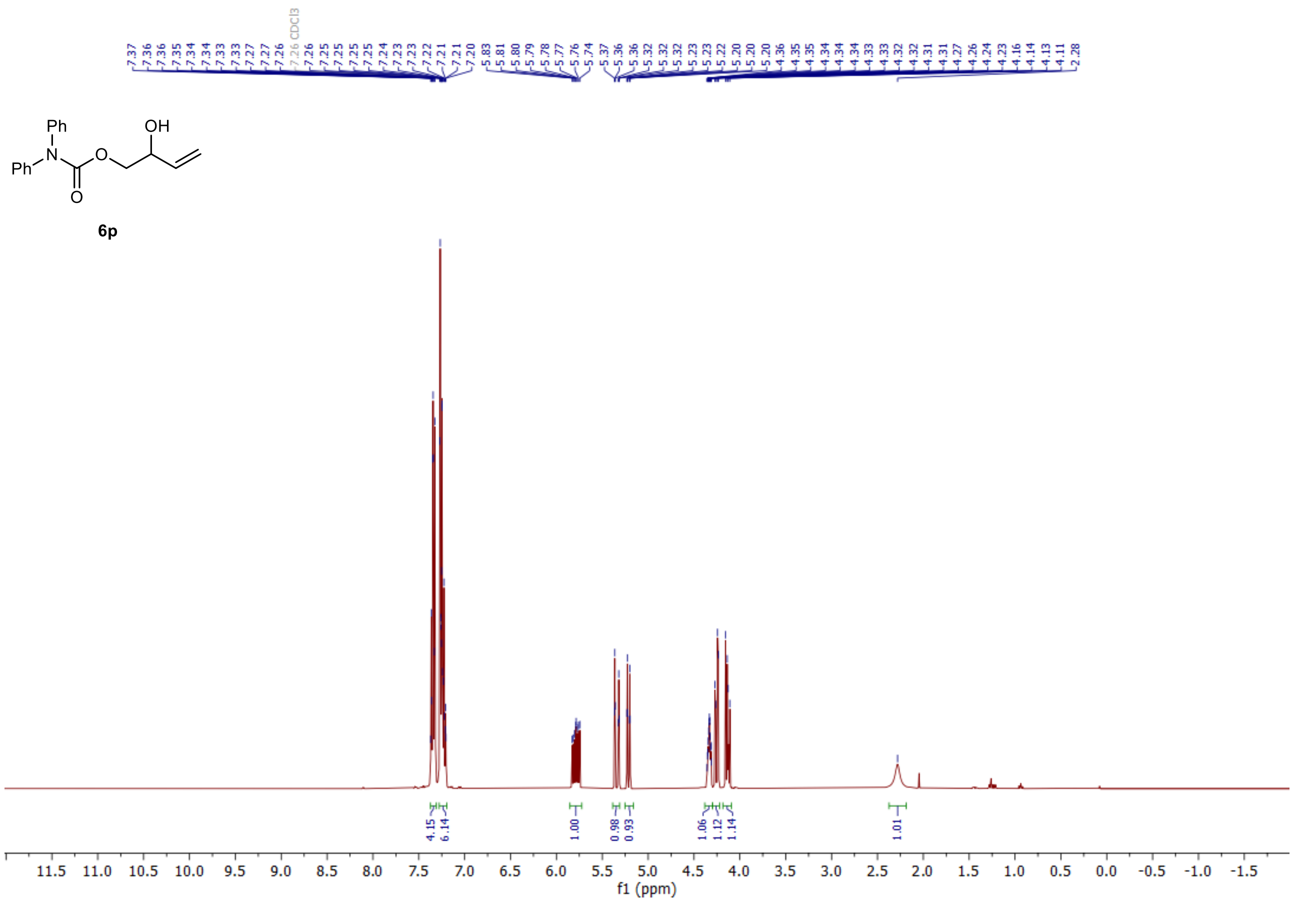

6p ^{13}C NMR (101 MHz, $CDCl_3$)

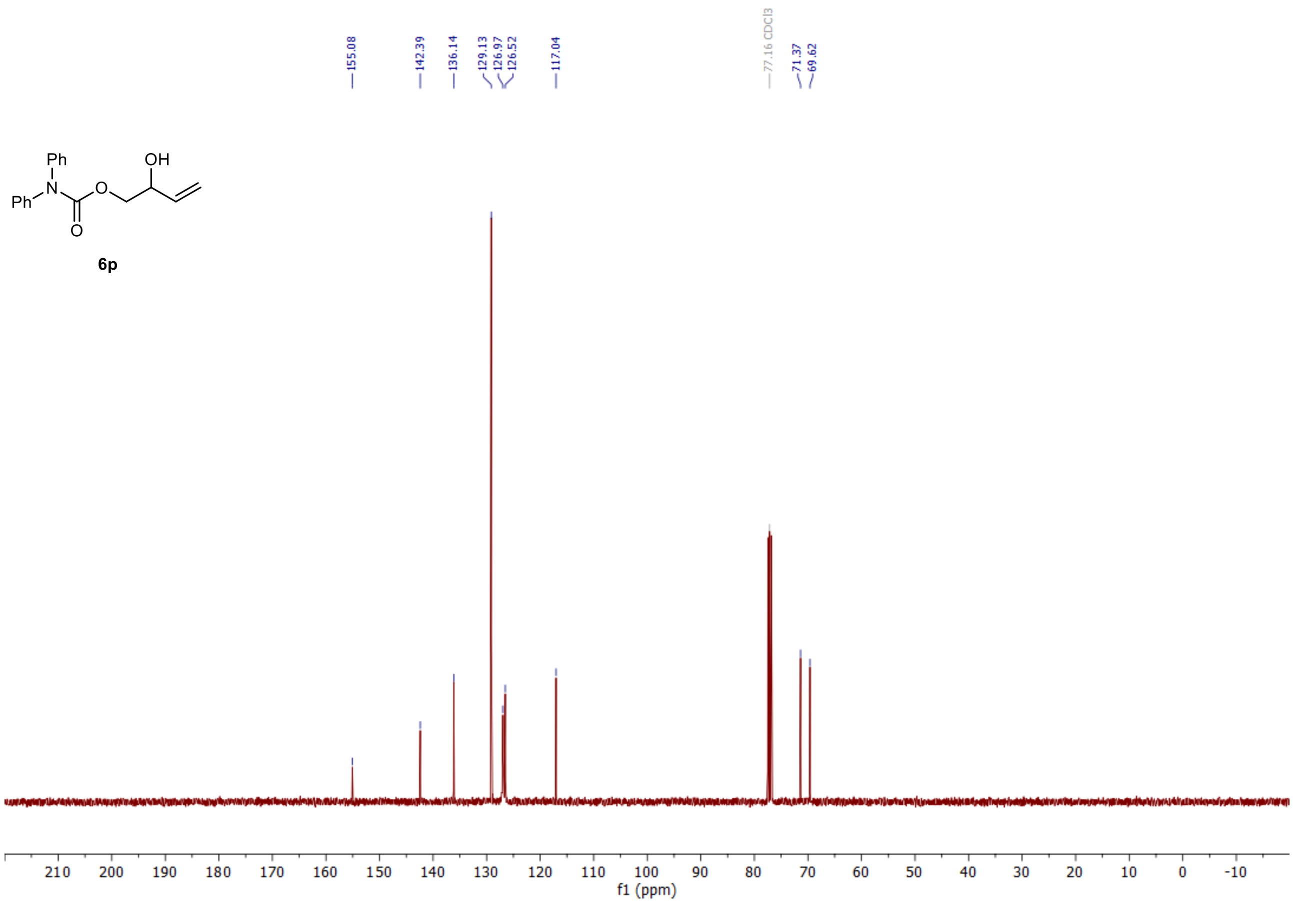

6q ^{1}H NMR (400 MHz, $CDCl_3$)

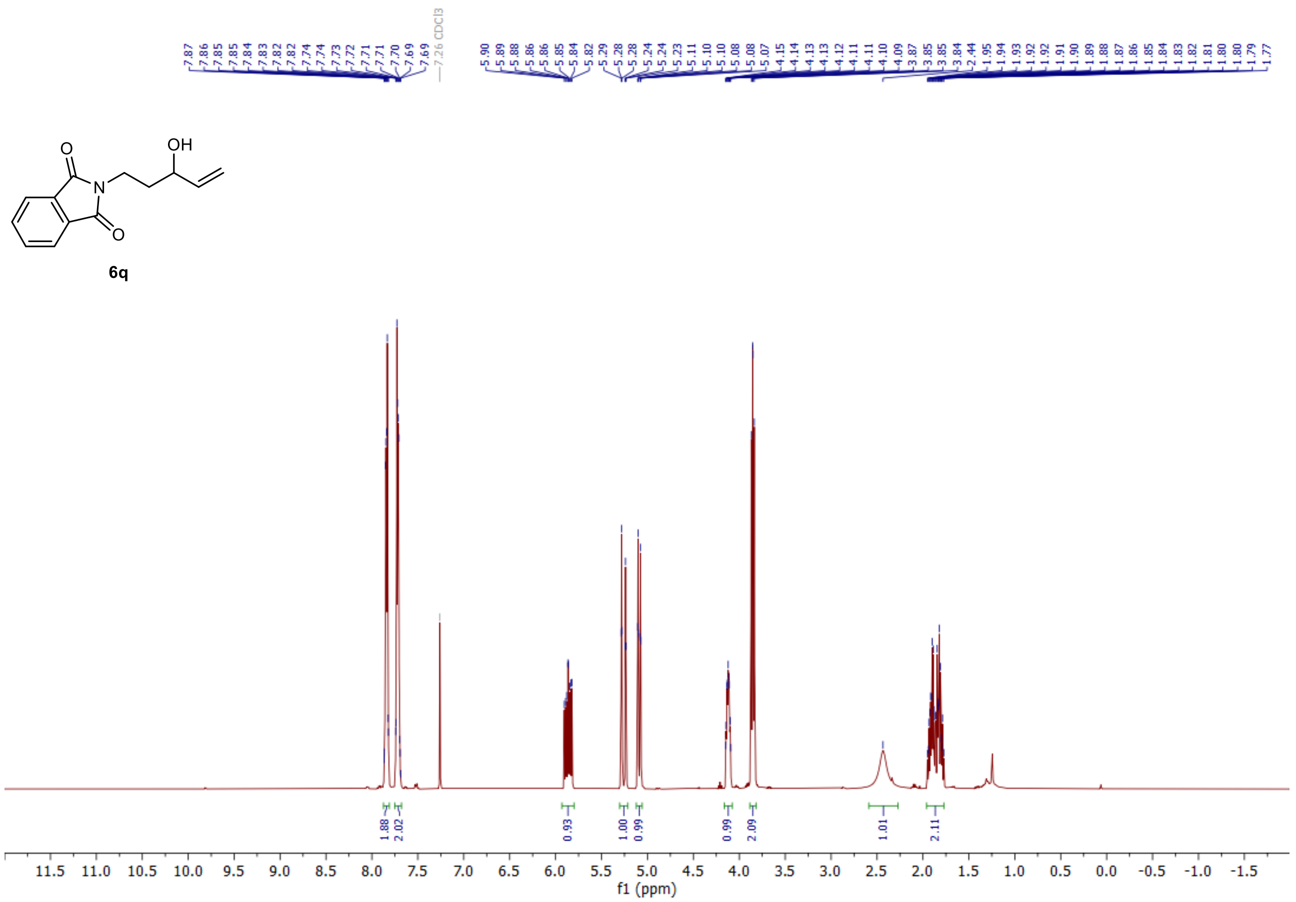

6q ^{13}C NMR (101 MHz, $CDCl_3$)

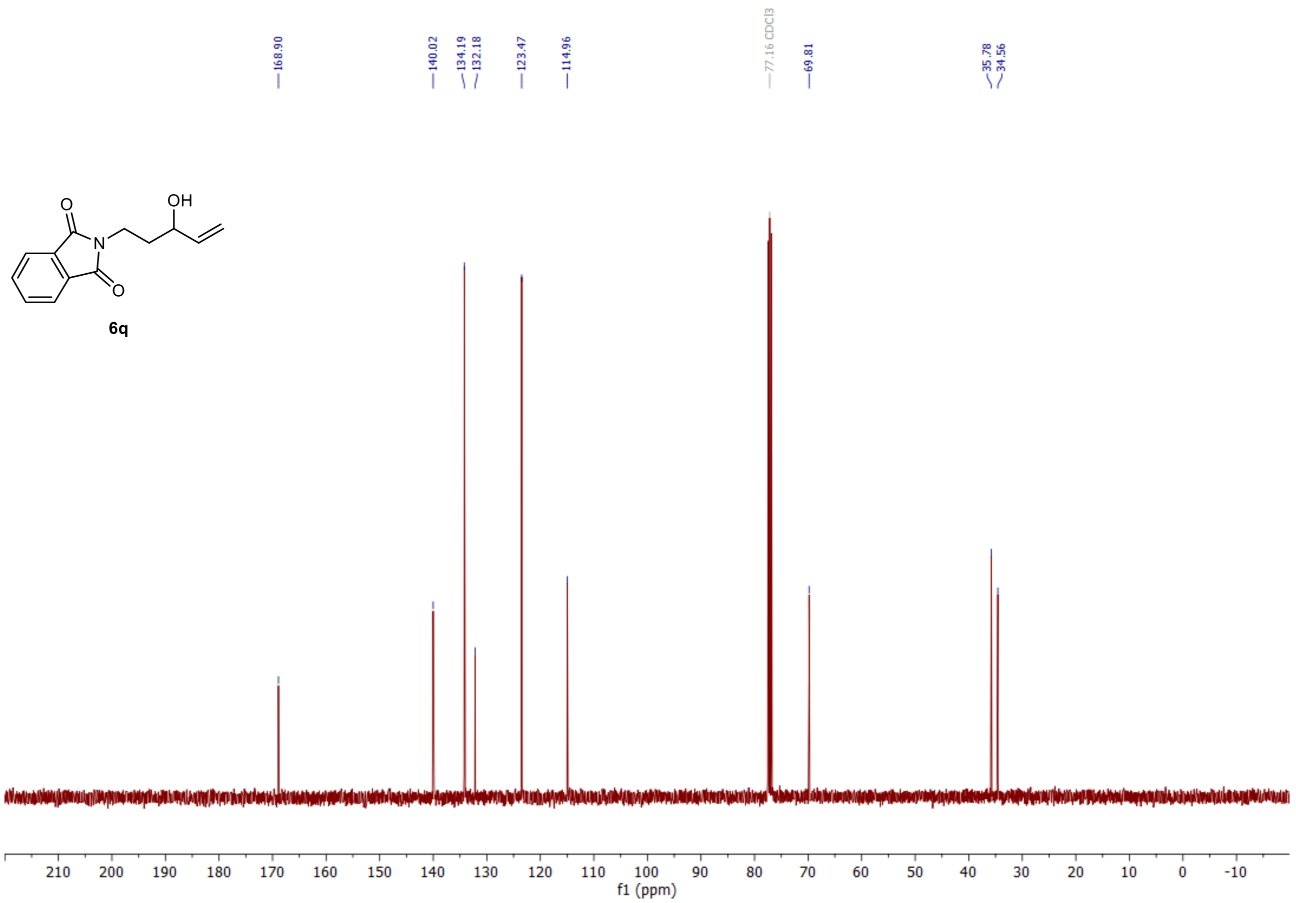

6r ^{1}H NMR (400 MHz, $CDCl_3$)

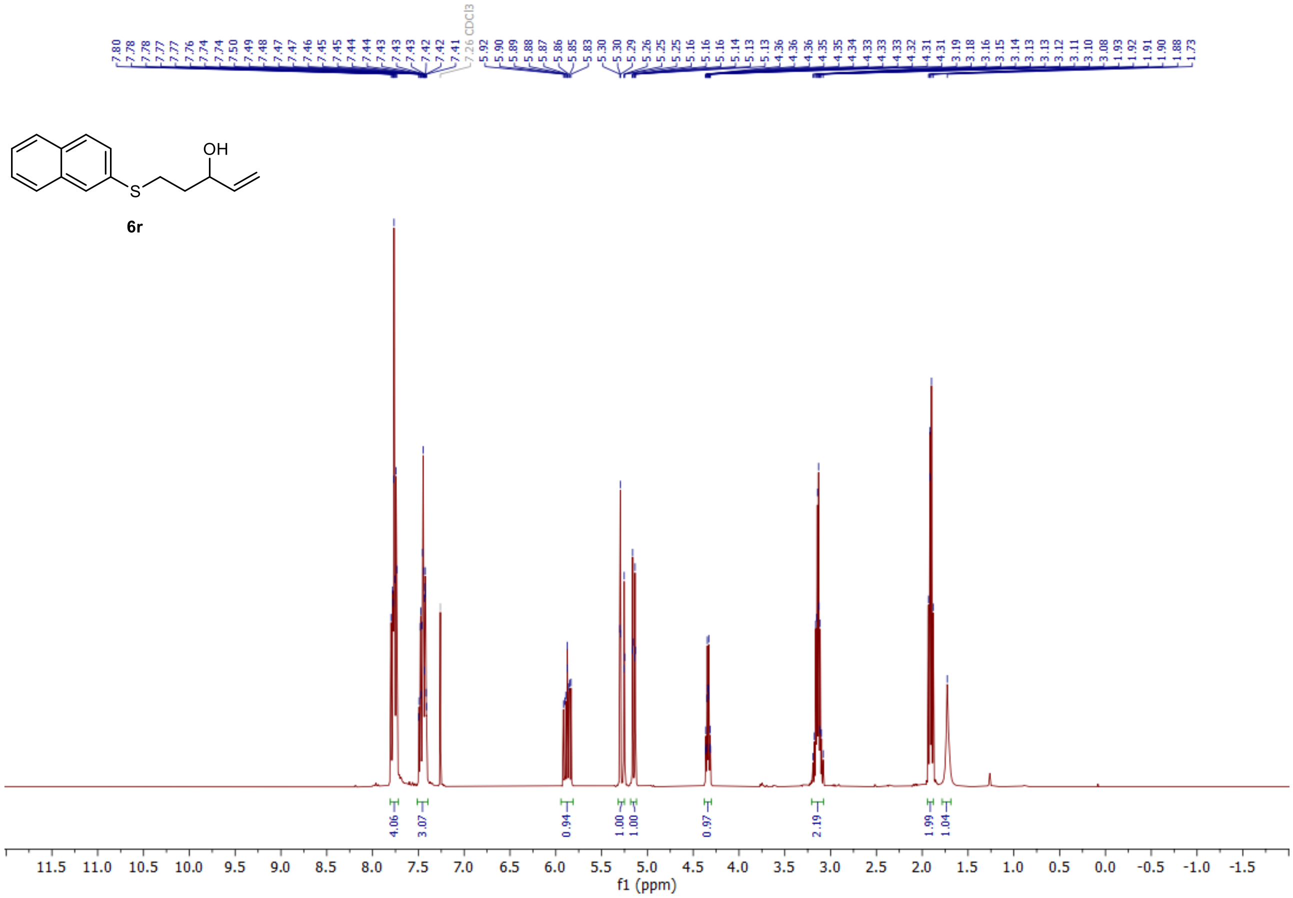

6r ^{13}C NMR (101 MHz, $CDCl_3$)

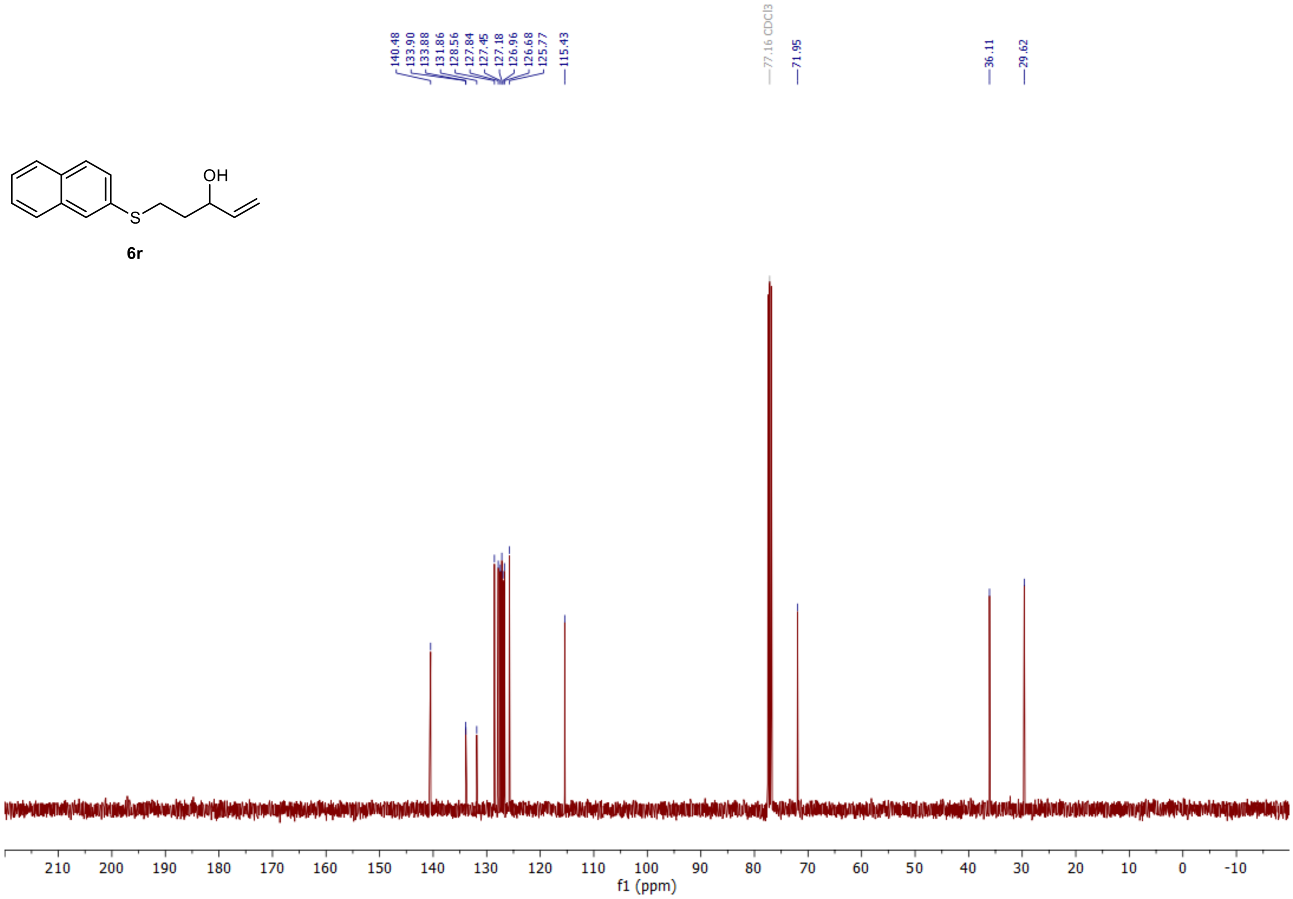

6u ^{1}H NMR (400 MHz, CD_2Cl_2)

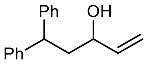

6u

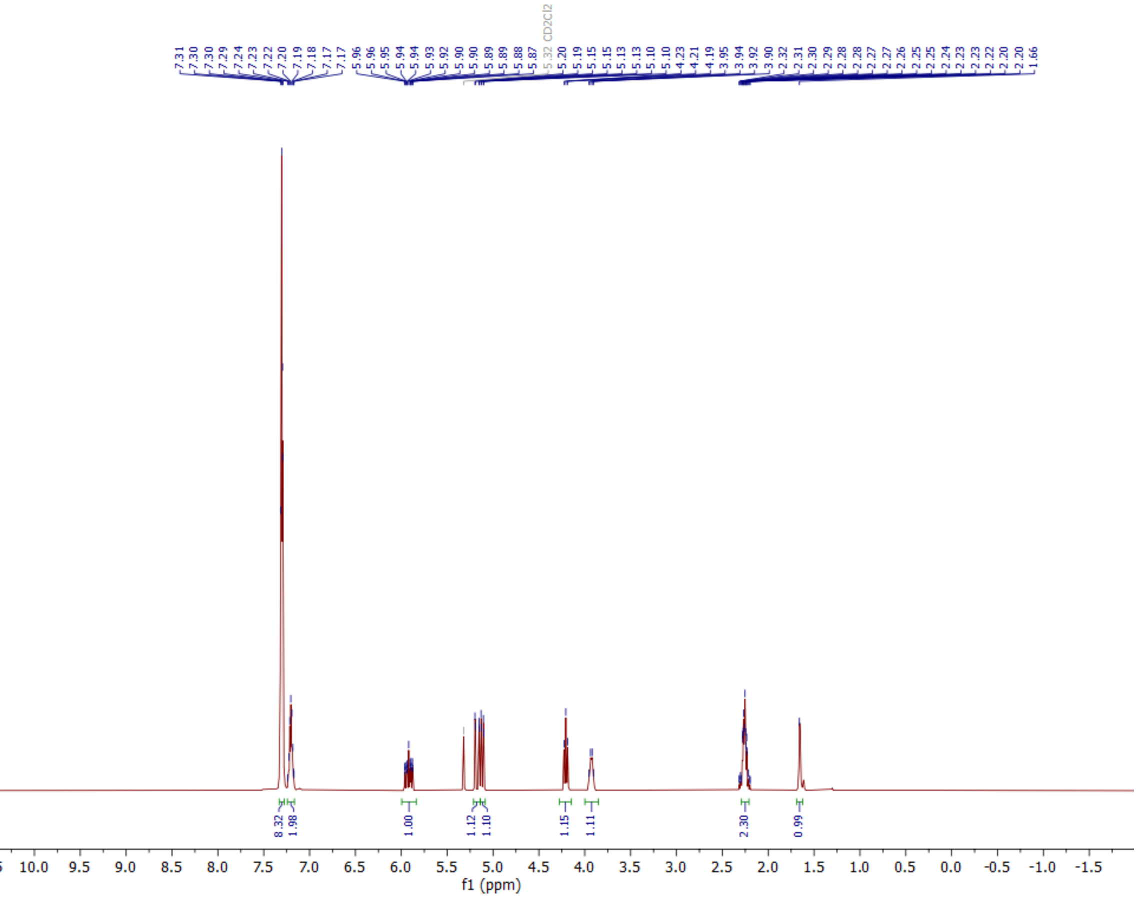

6u ^{13}C NMR (101 MHz, CD_2Cl_2)

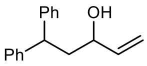

6u

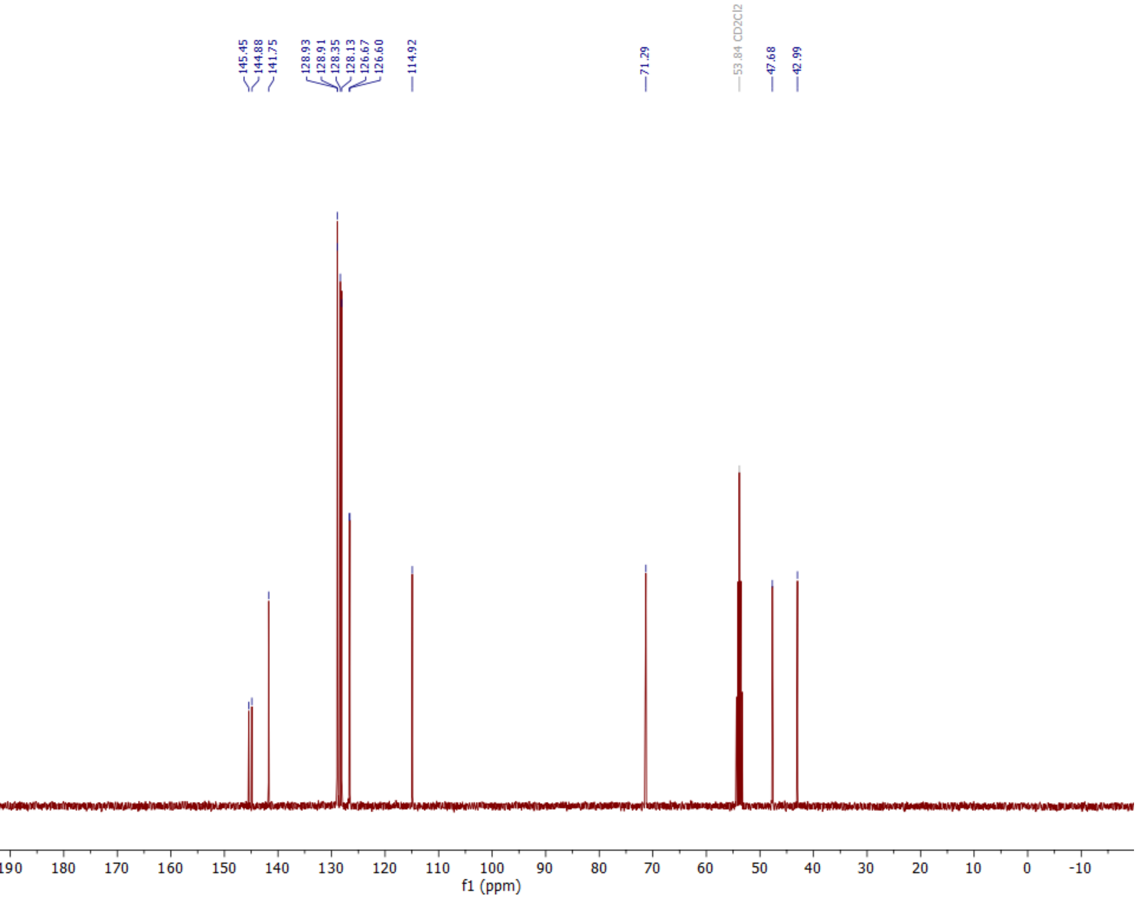

6v ^{1}H NMR (400 MHz, $CDCl_3$)

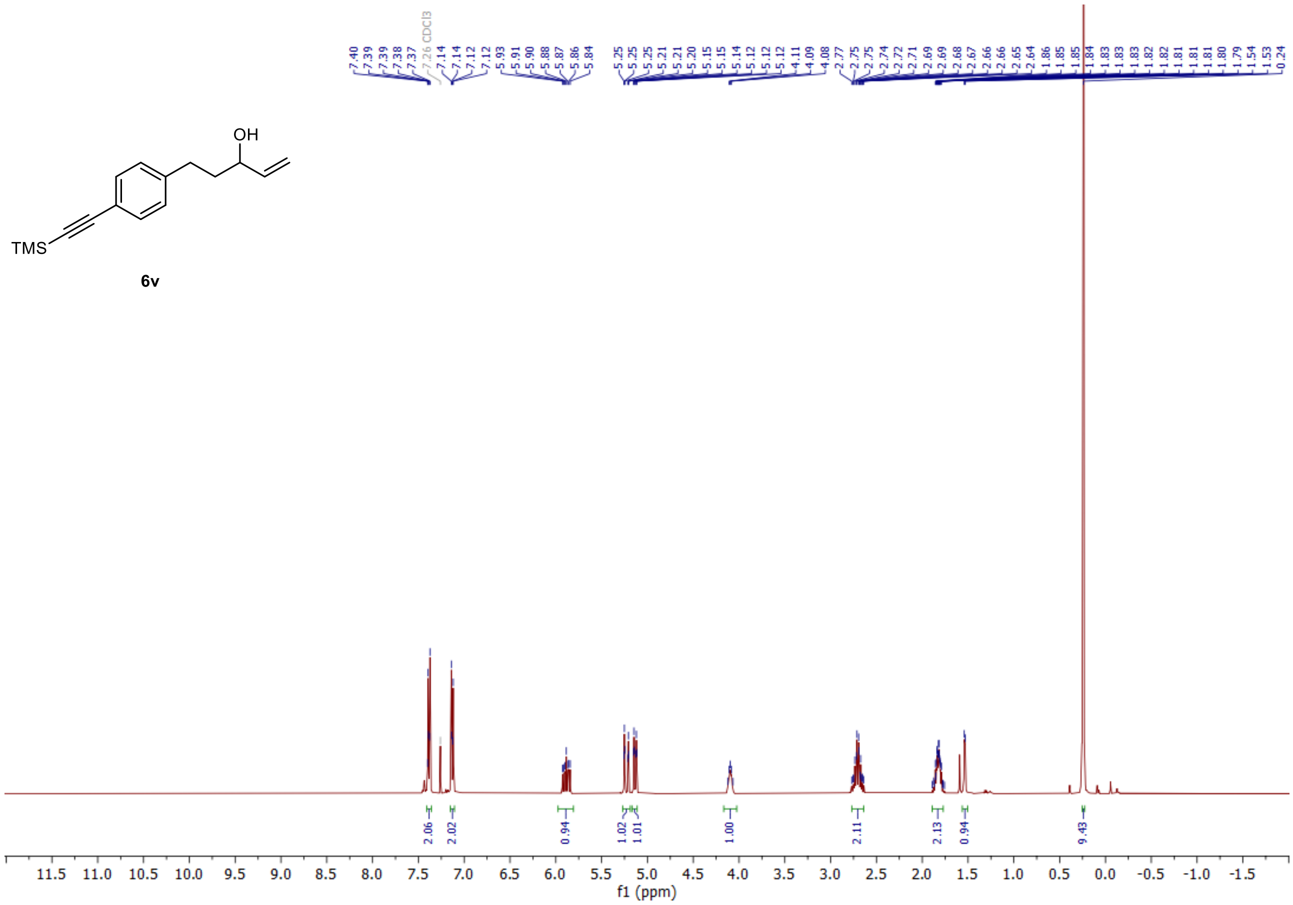

6v ^{13}C NMR (101 MHz, $CDCl_3$)

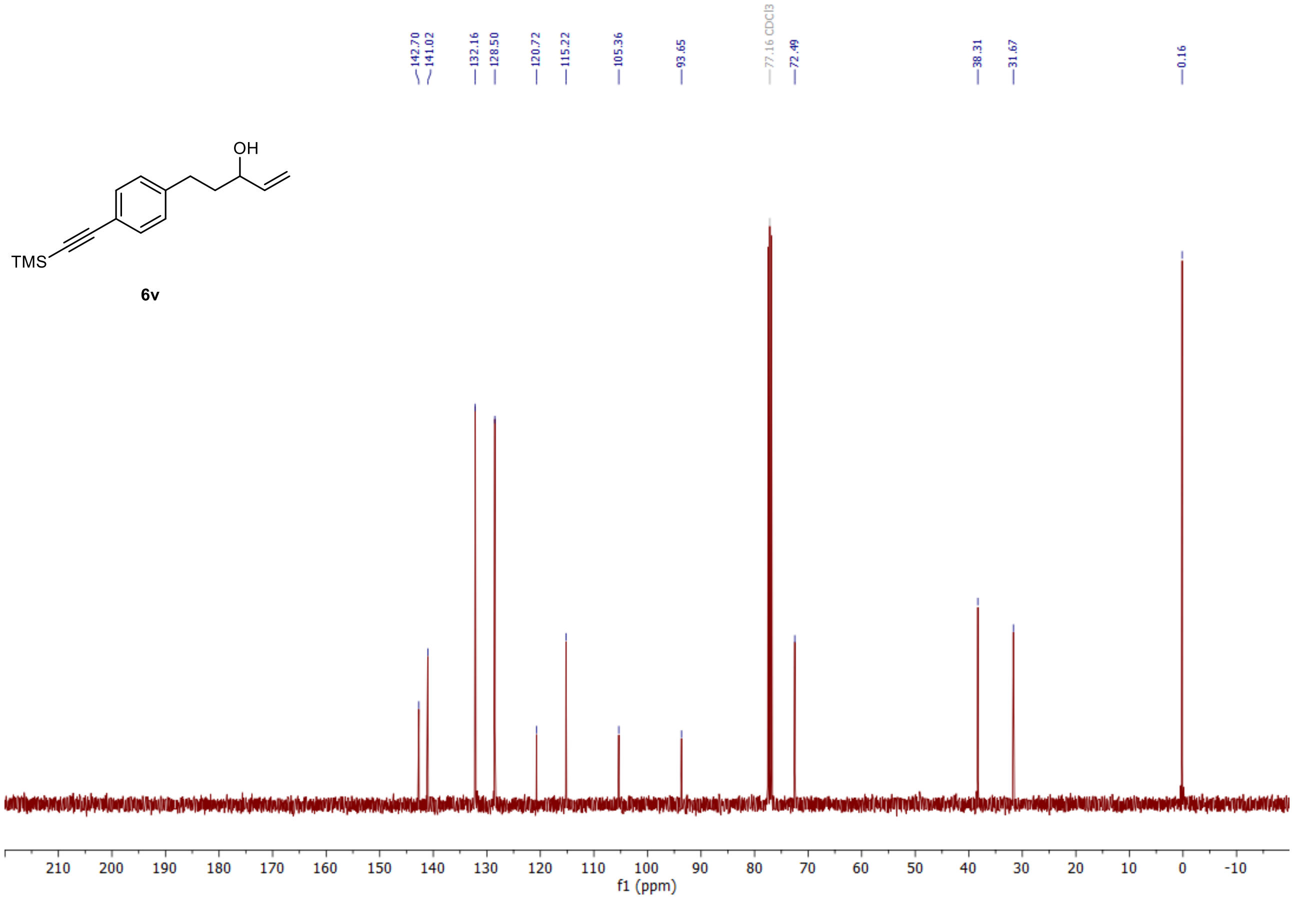

6w ^{1}H NMR (400 MHz, $CDCl_3$)

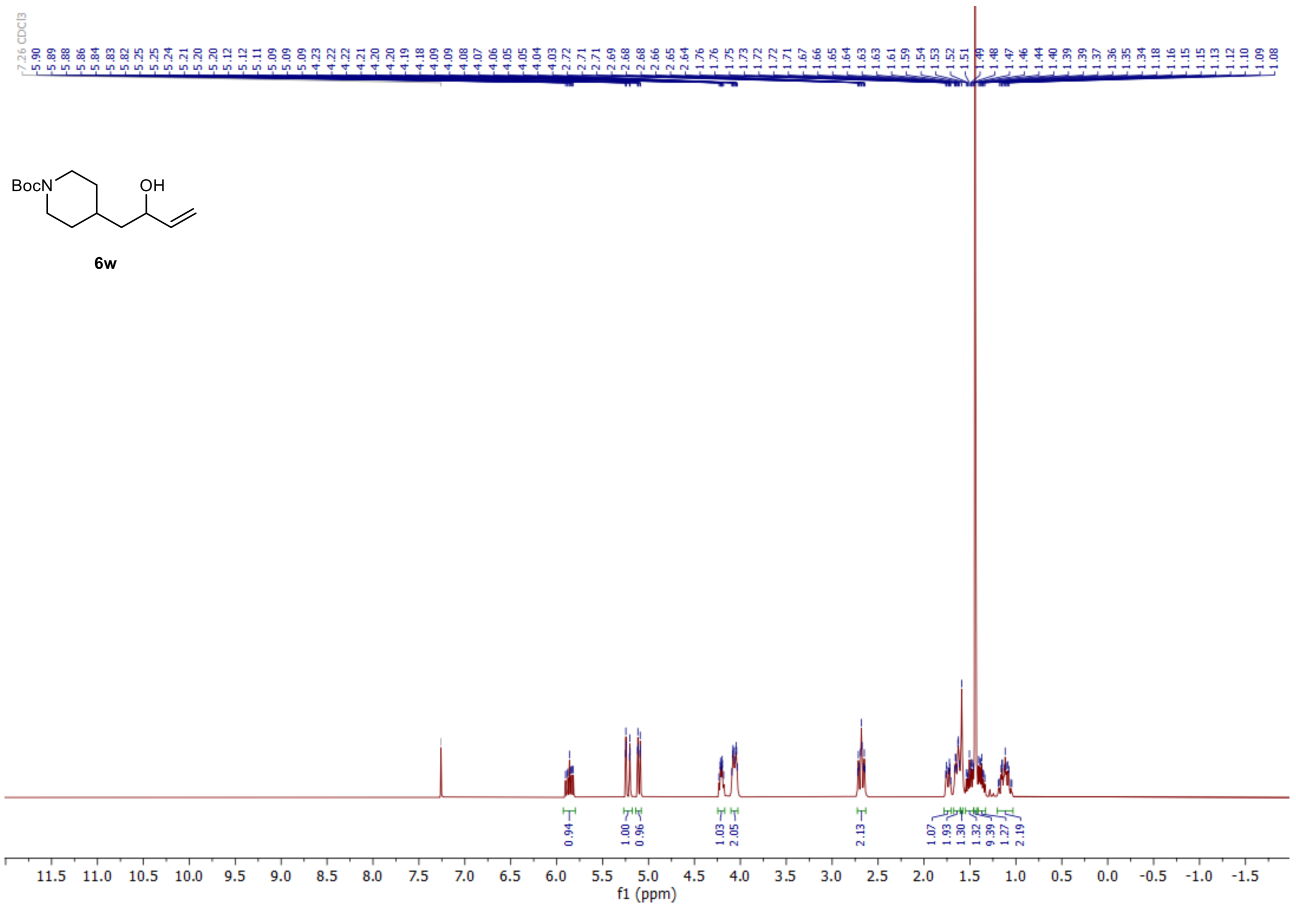

6w ^{13}C NMR (101 MHz, $CDCl_3$)

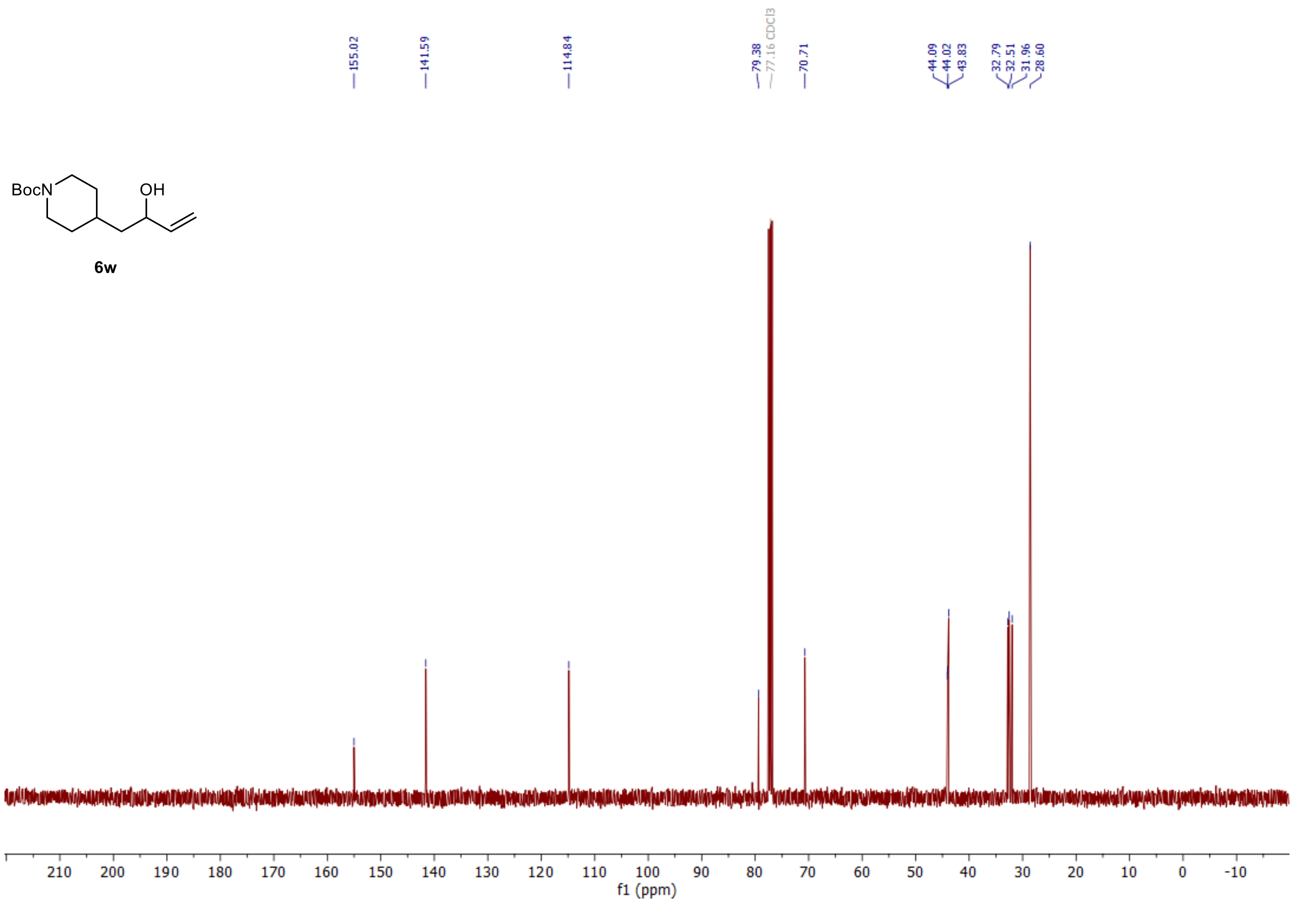

6z ^{1}H NMR (400 MHz, $CDCl_3$)

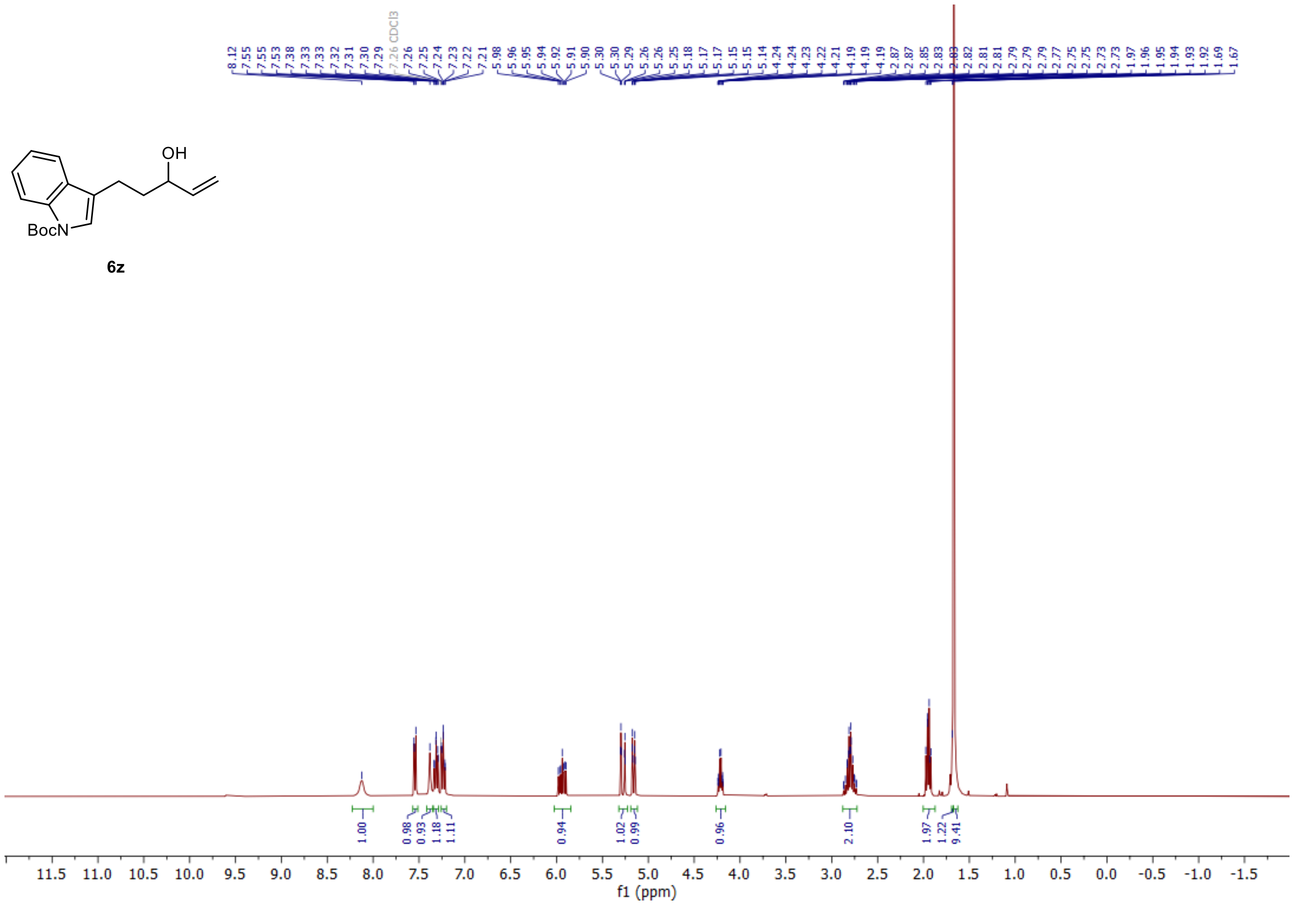

6z ^{13}C NMR (101 MHz, $CDCl_3$)

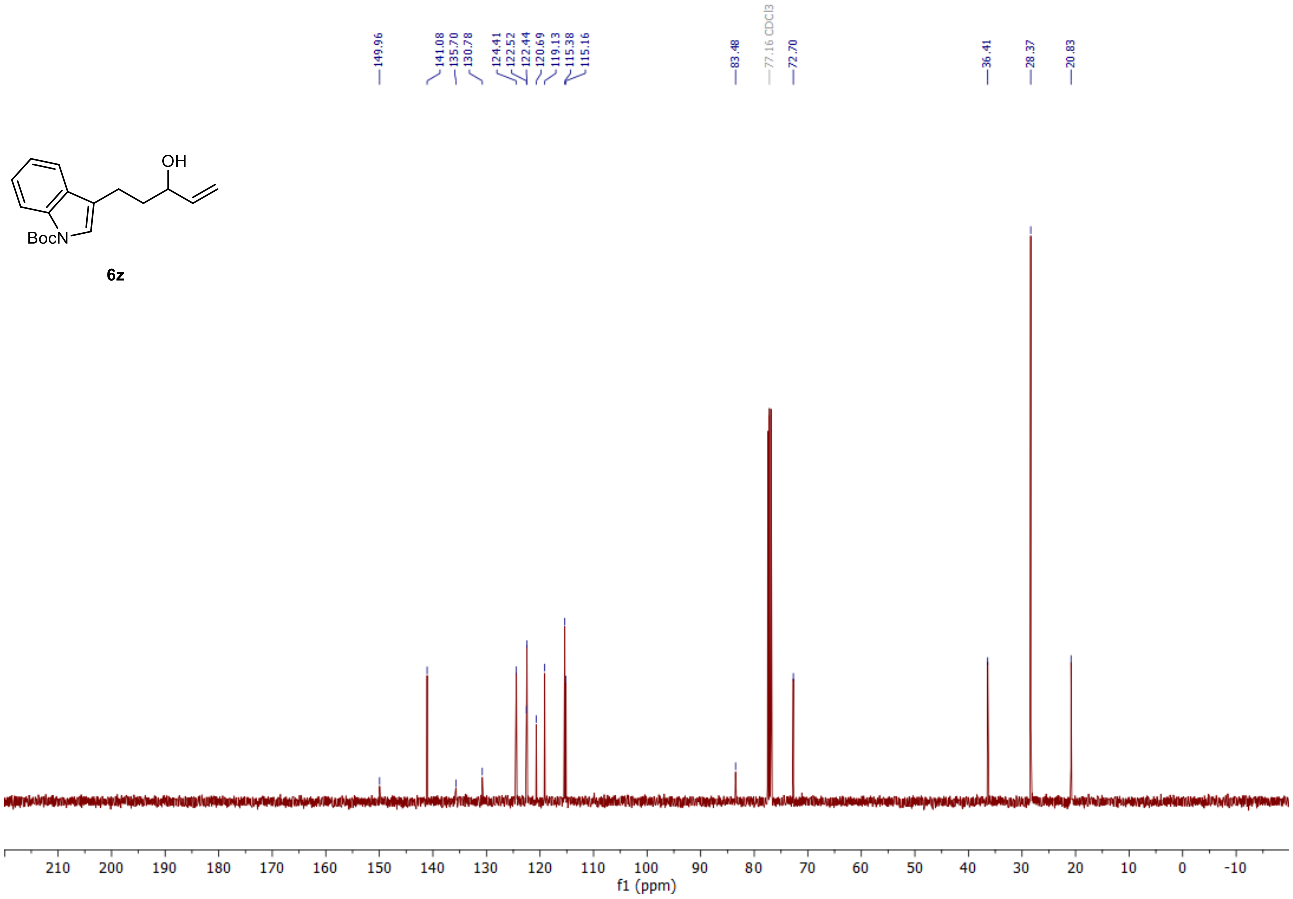

6aa ^{1}H NMR (400 MHz, $CDCl_3$)

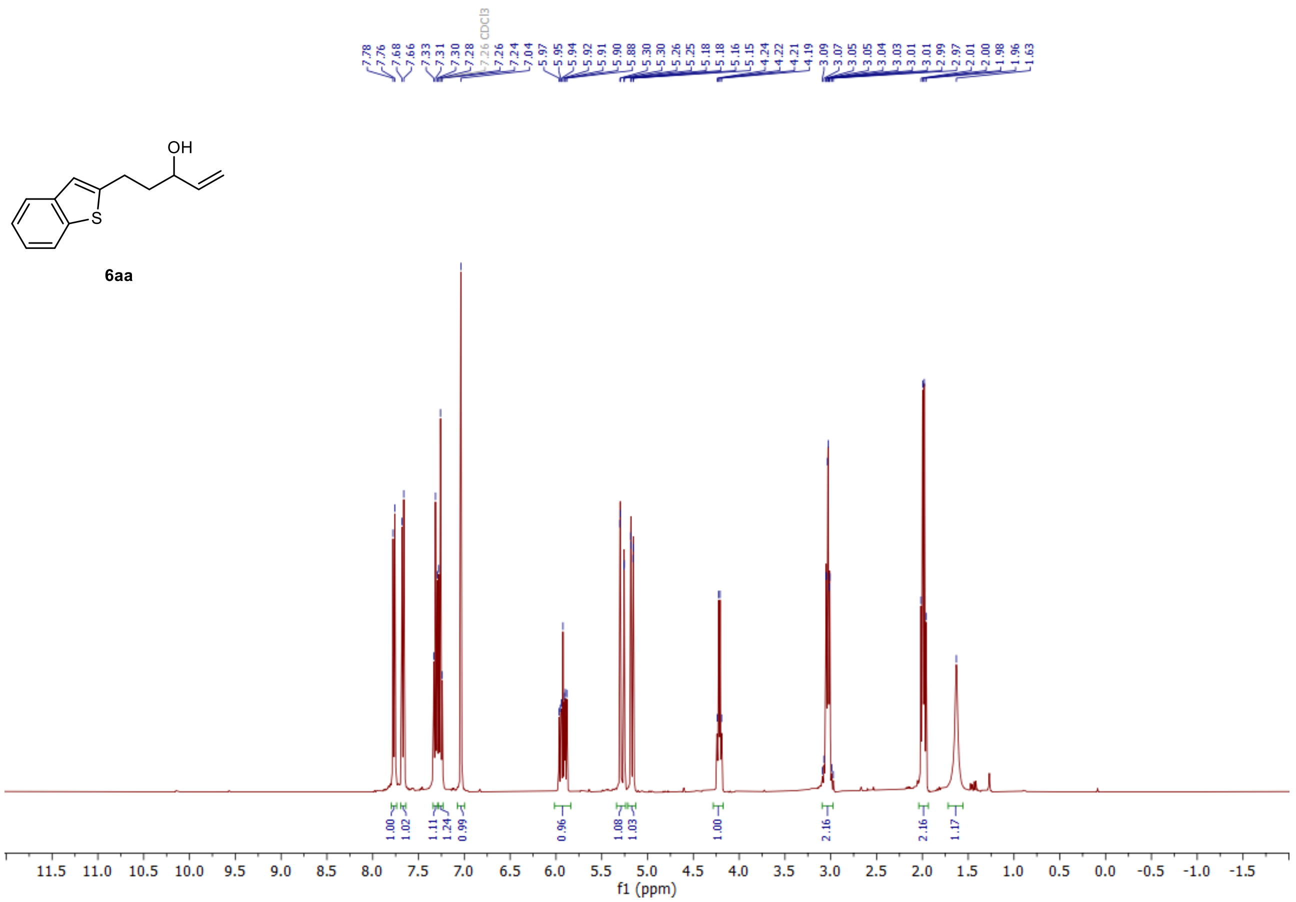

6aa ^{13}C NMR (101 MHz, $CDCl_3$)

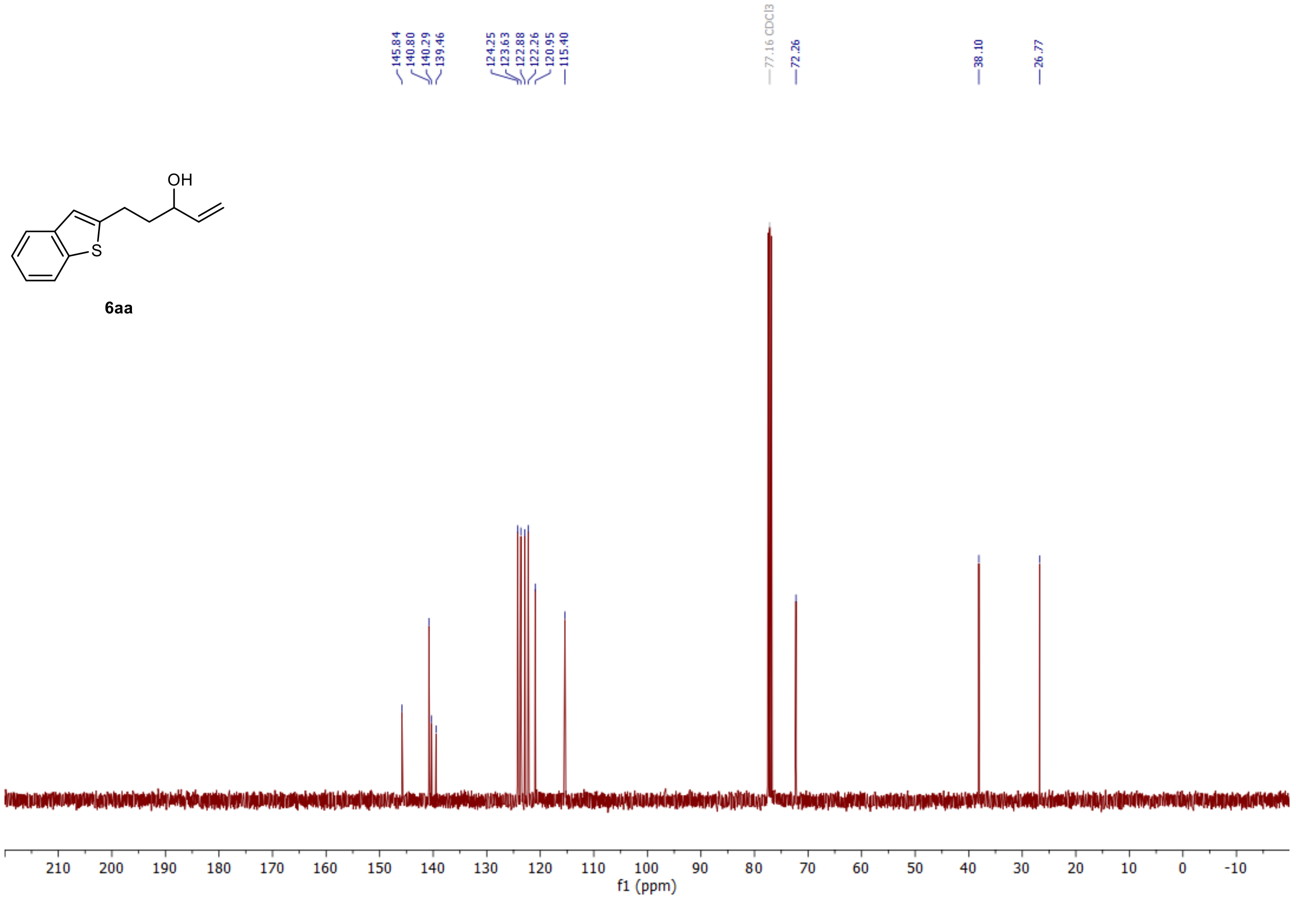

6ab ^{1}H NMR (400 MHz, $CDCl_3$)

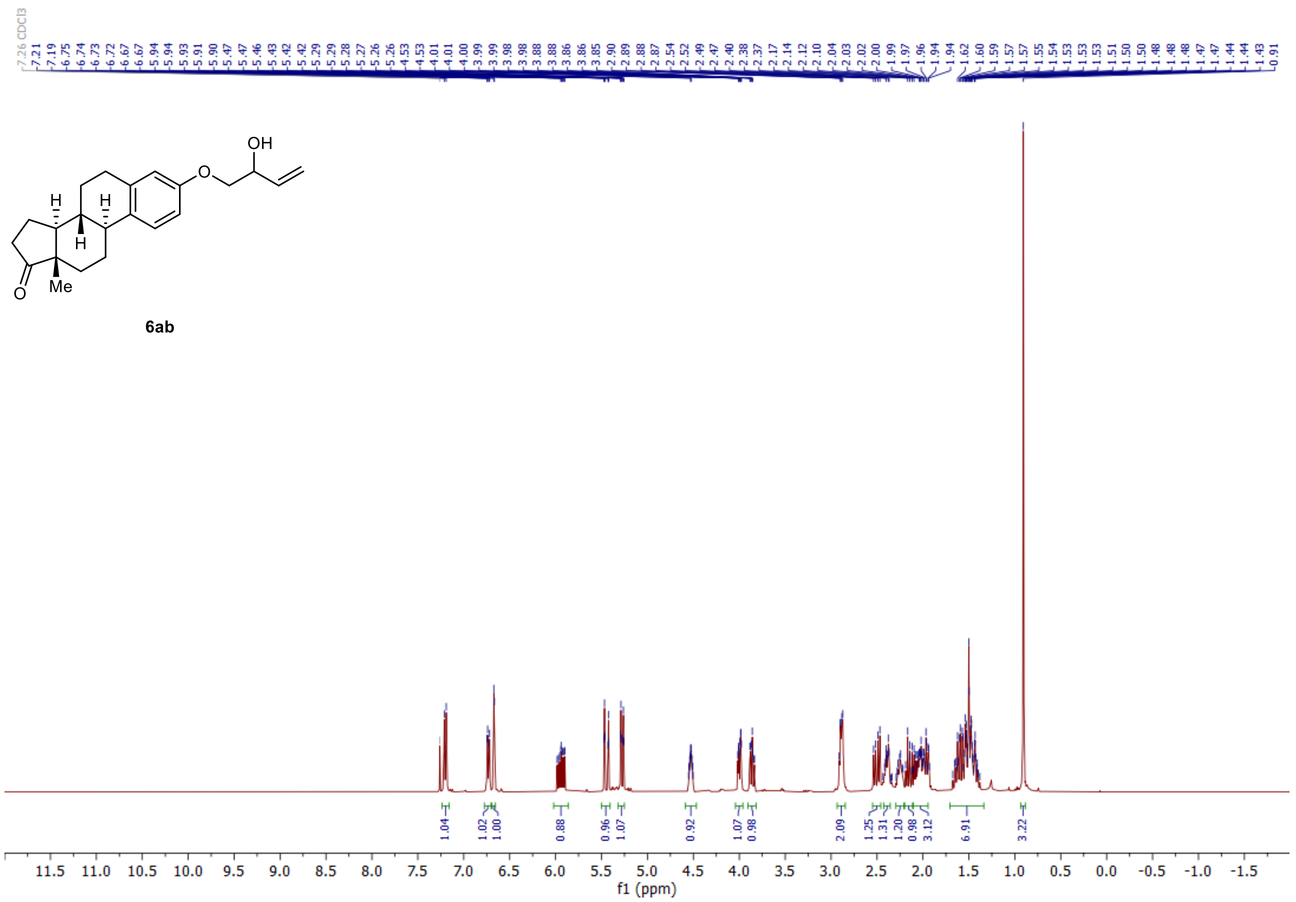

6ab ^{13}C NMR (101 MHz, $CDCl_3$)

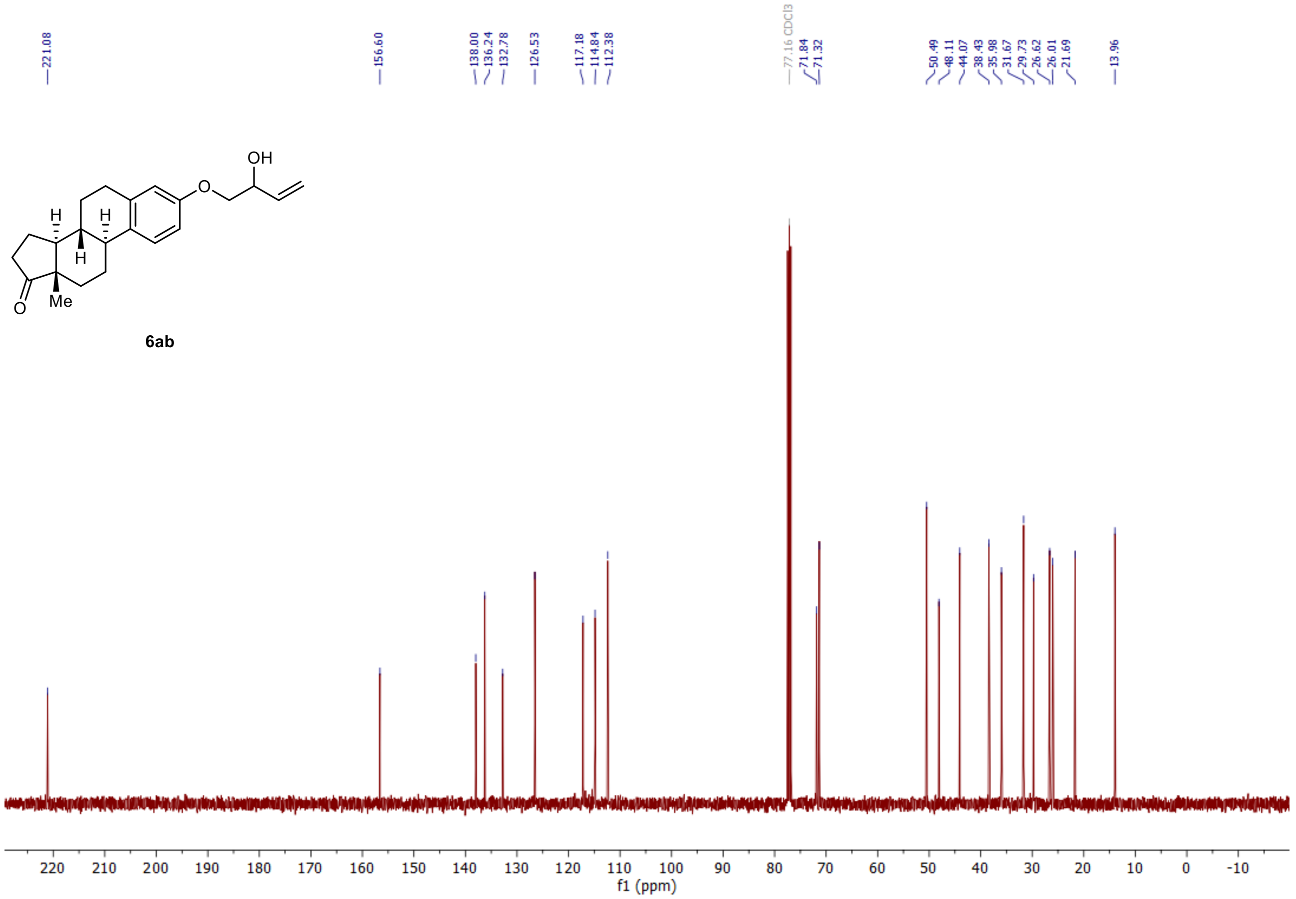

1a ^{1}H NMR (400 MHz, $CDCl_3$)

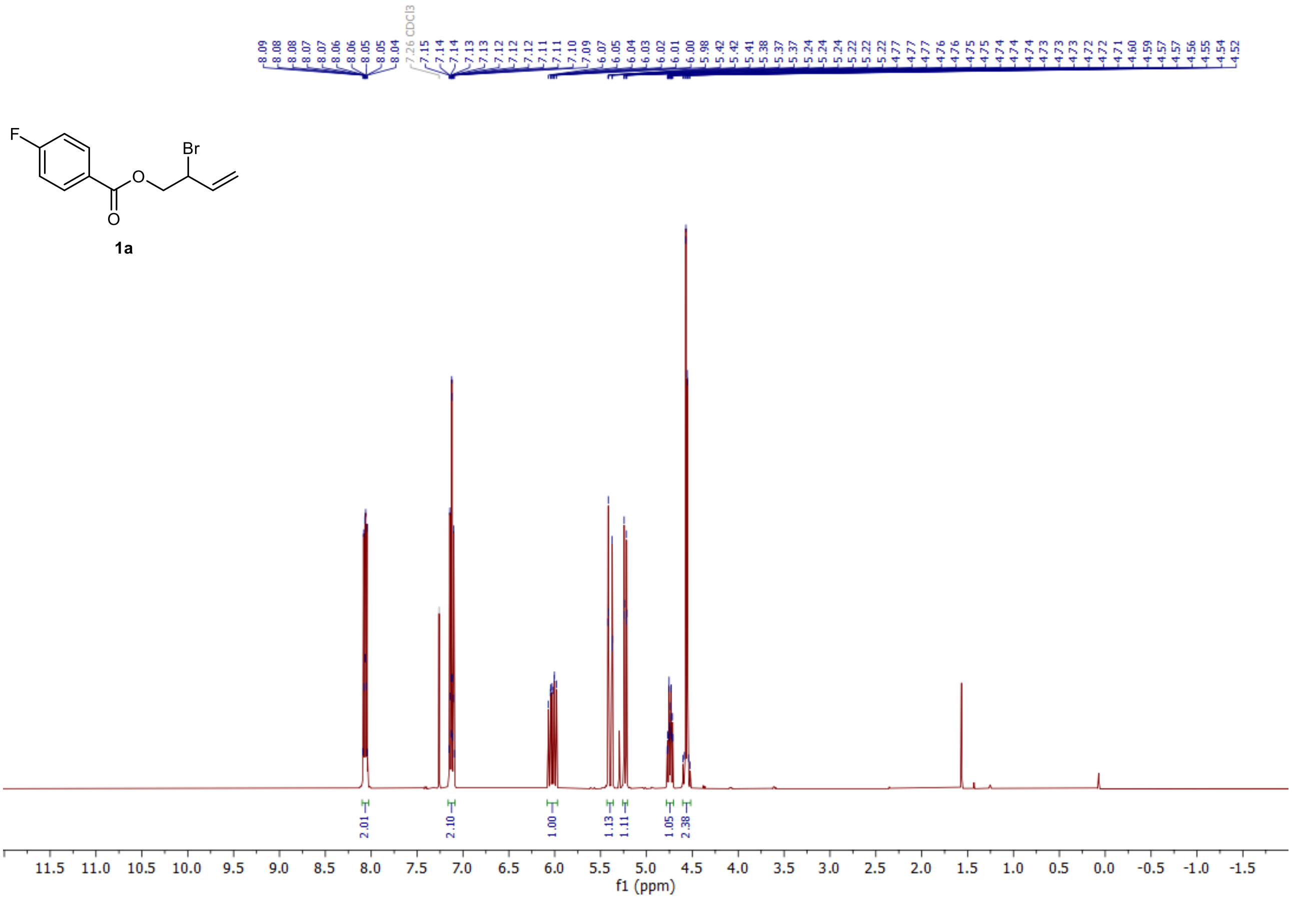

1a ^{13}C NMR (101 MHz, $CDCl_3$)

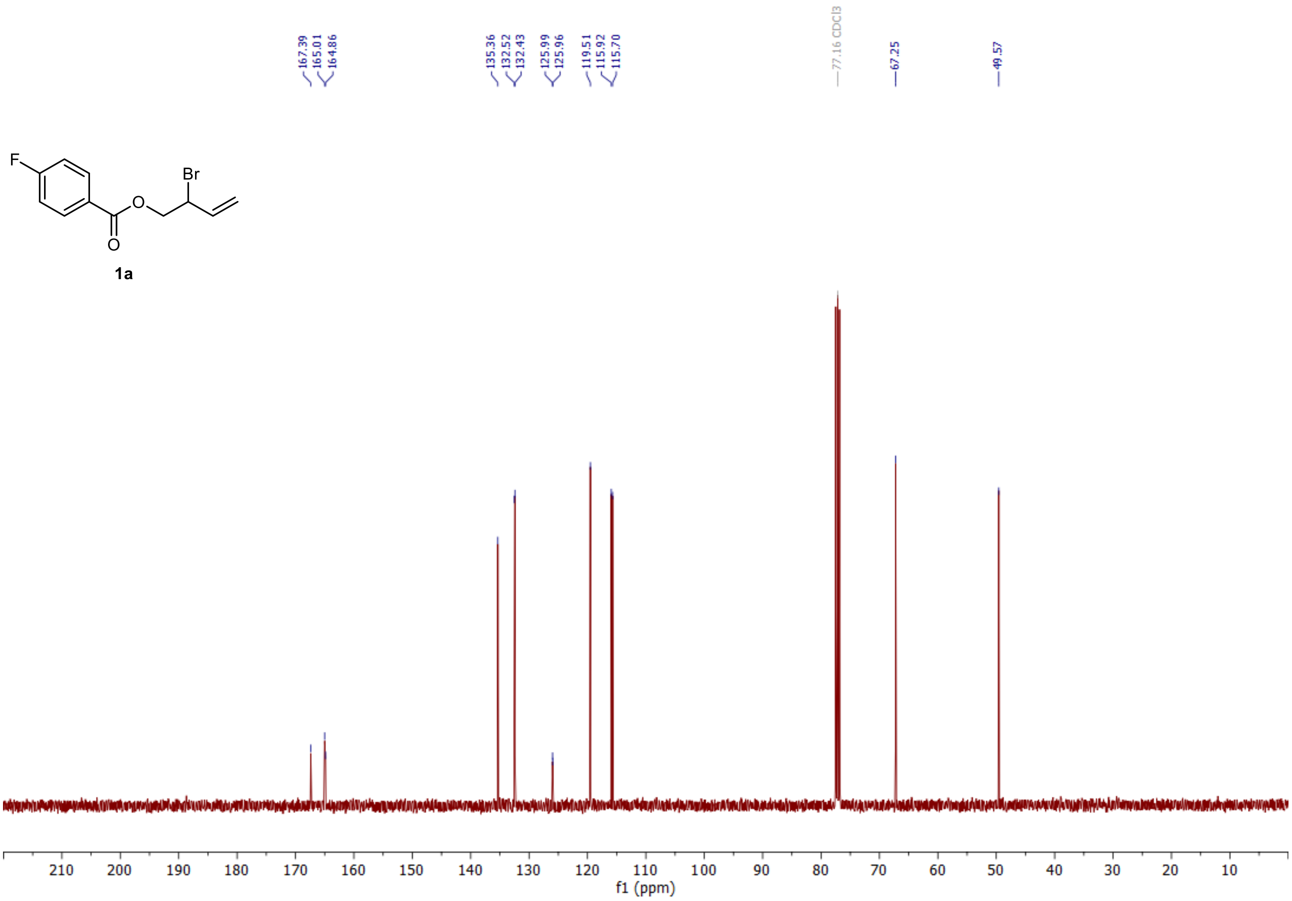

1a ^{19}F NMR (377 MHz, $CDCl_3$)

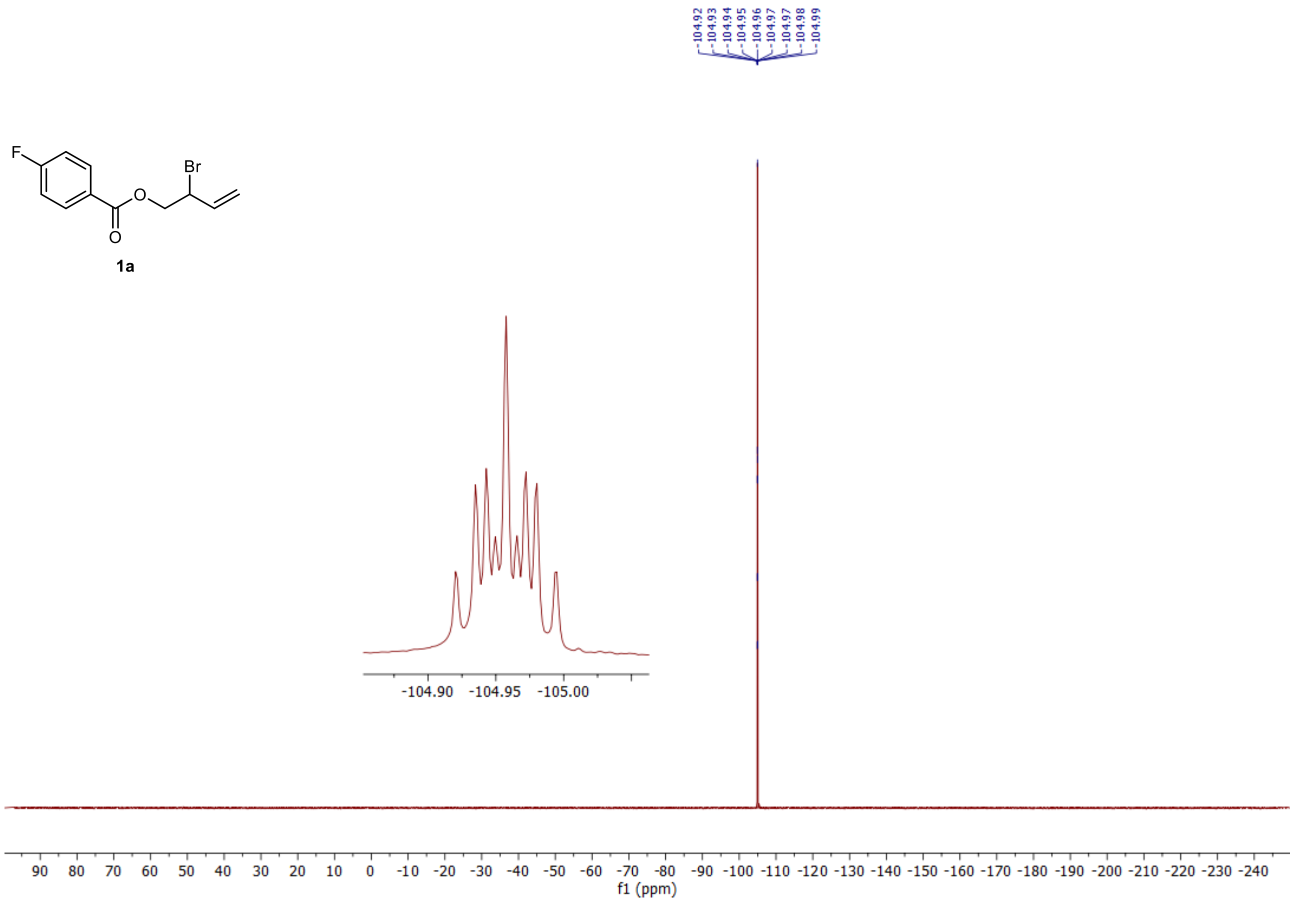

4a ^{1}H NMR (400 MHz, $CDCl_3$)

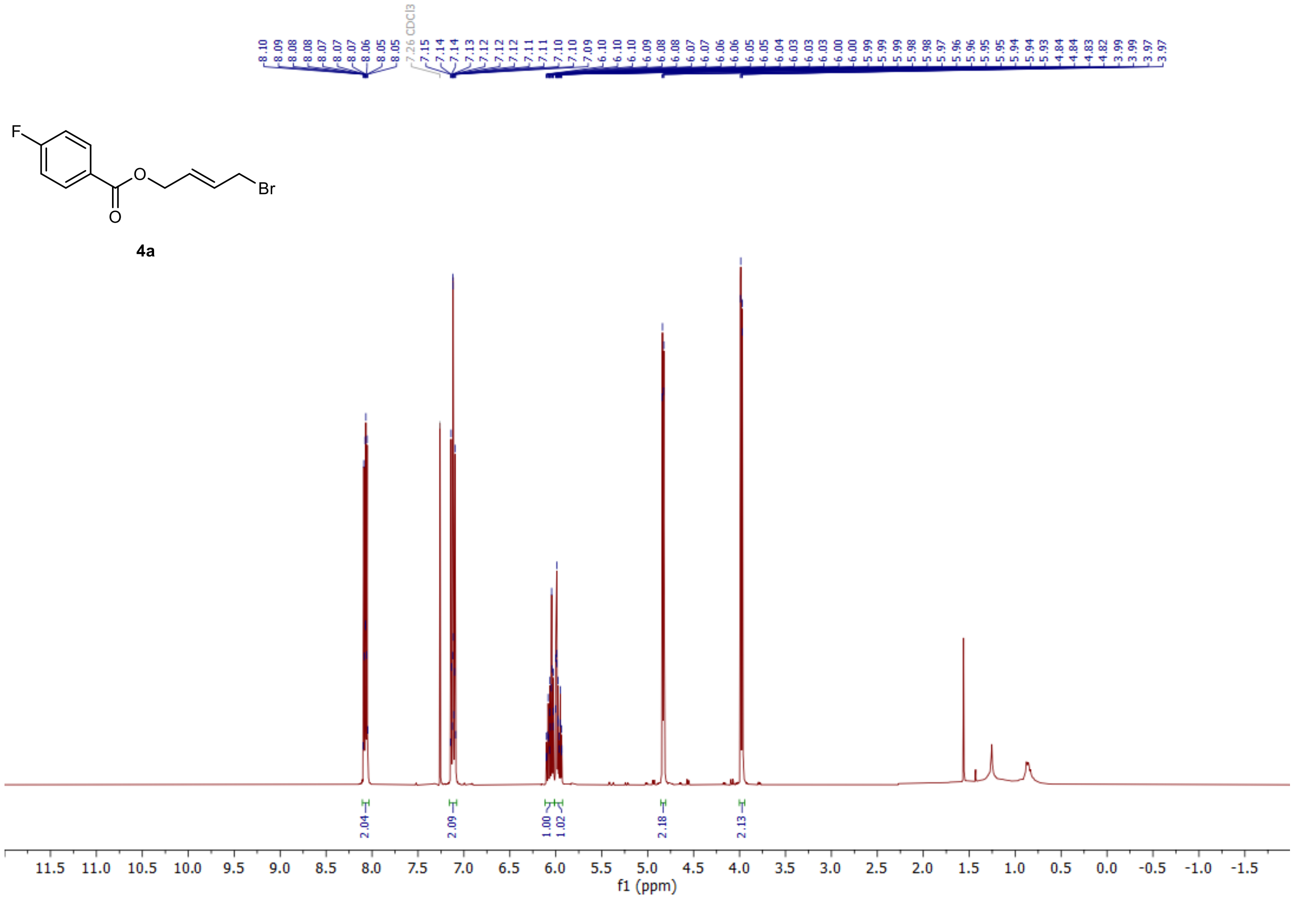

4a ^{13}C NMR (101 MHz, $CDCl_3$)

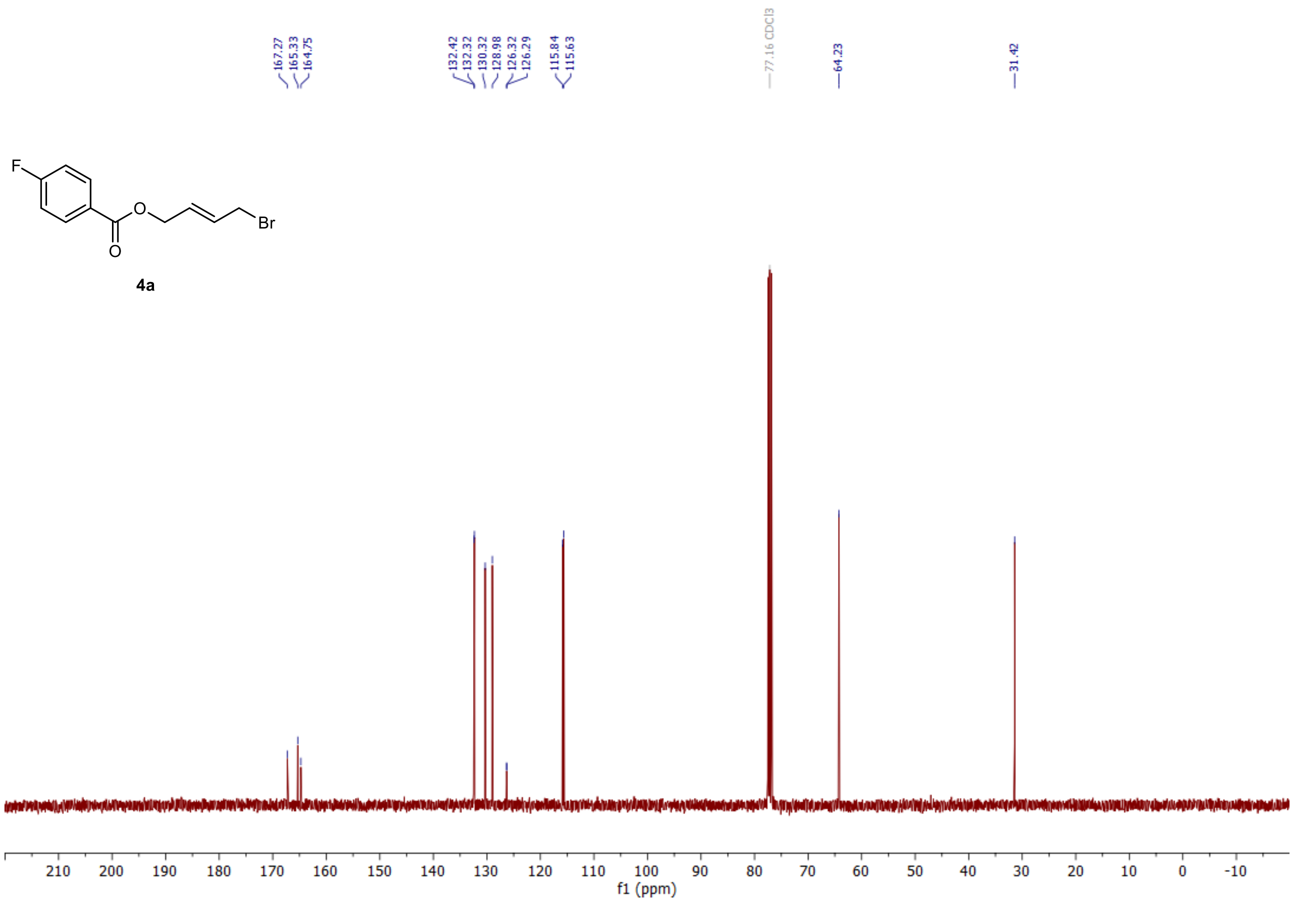

4a ^{19}F NMR (377 MHz, $CDCl_3$)

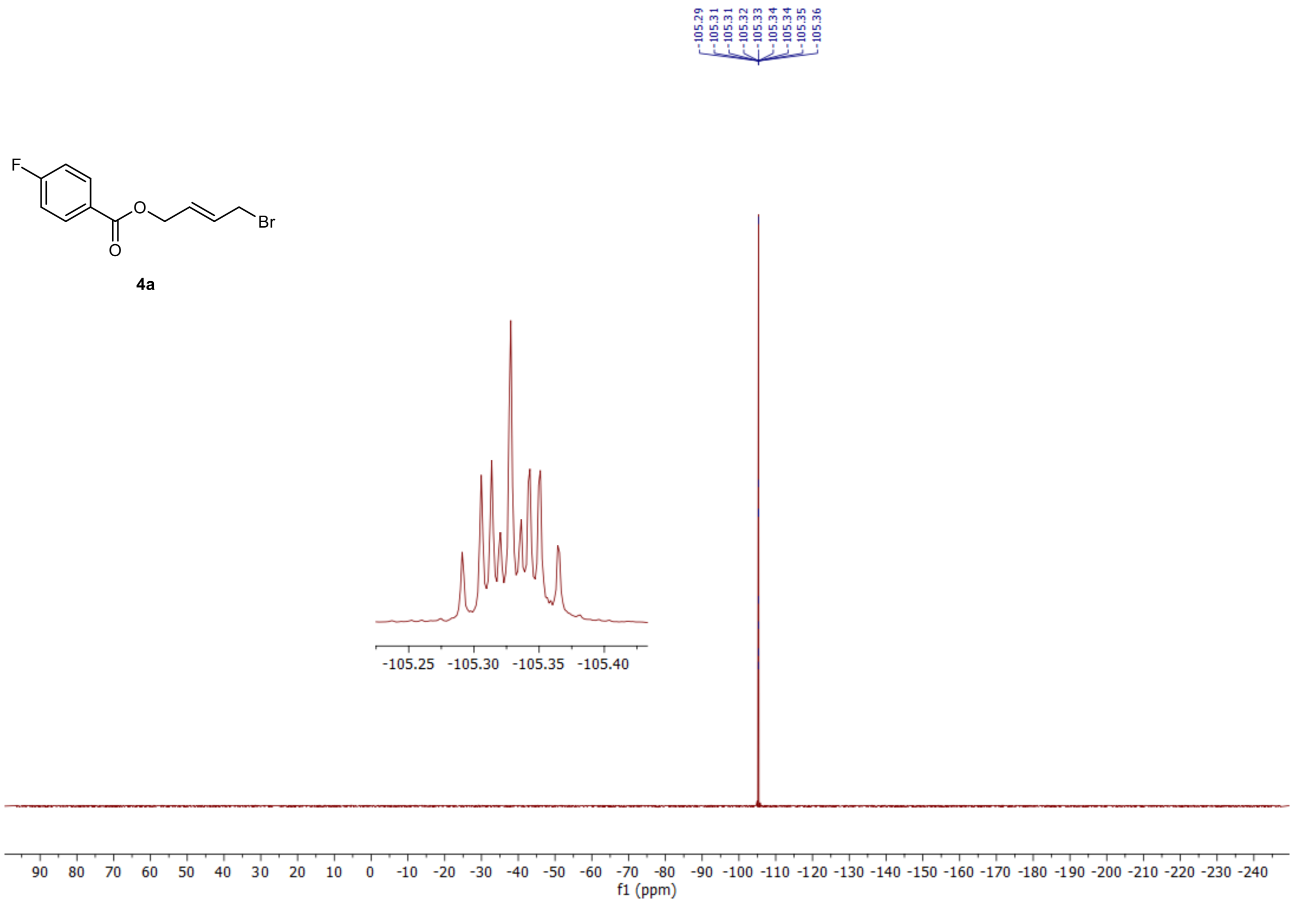

1b ^{1}H NMR (400 MHz, $CDCl_3$)

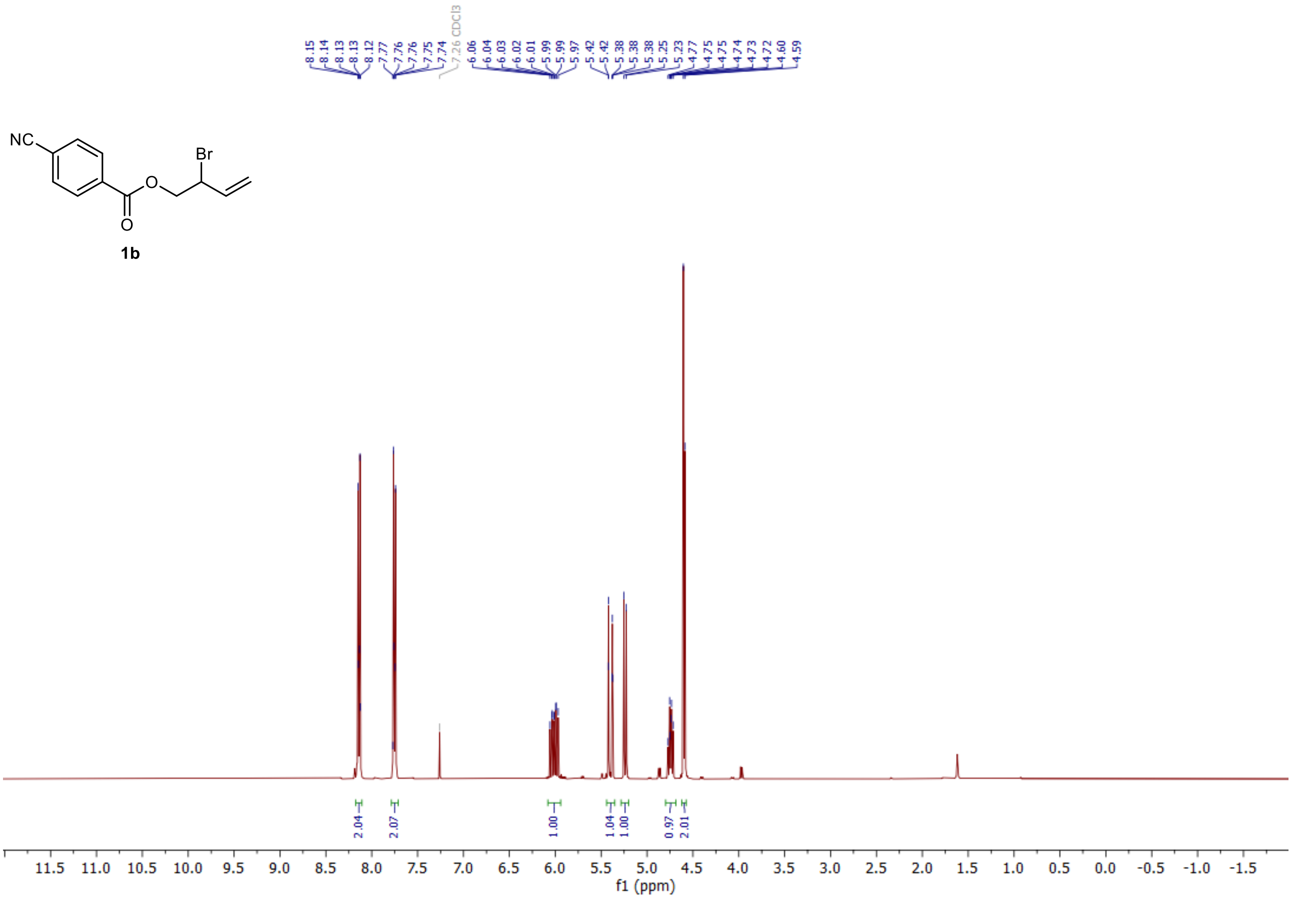

1b ^{13}C NMR (101 MHz, $CDCl_3$)

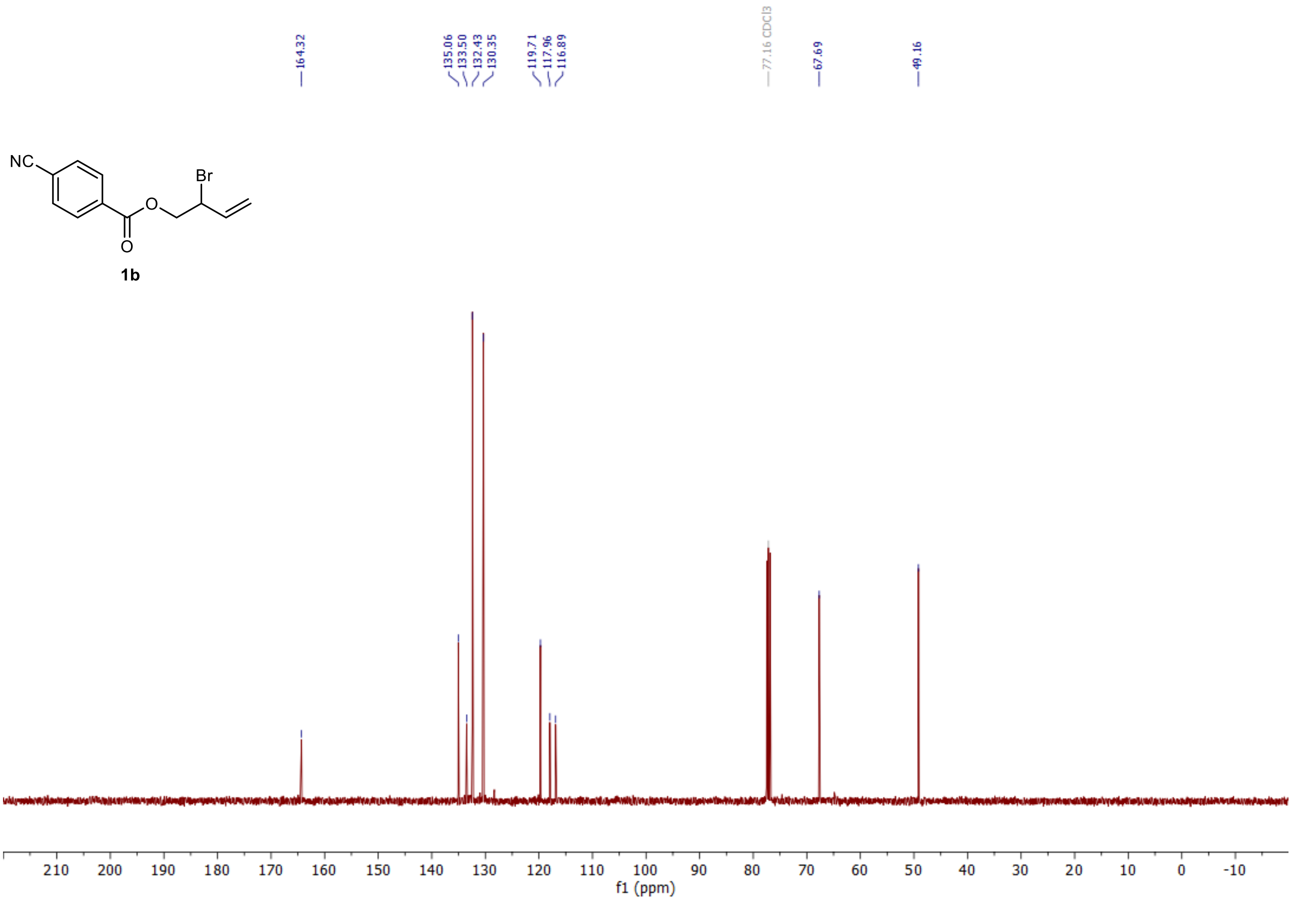

1c ^{1}H NMR (400 MHz, $CDCl_3$)

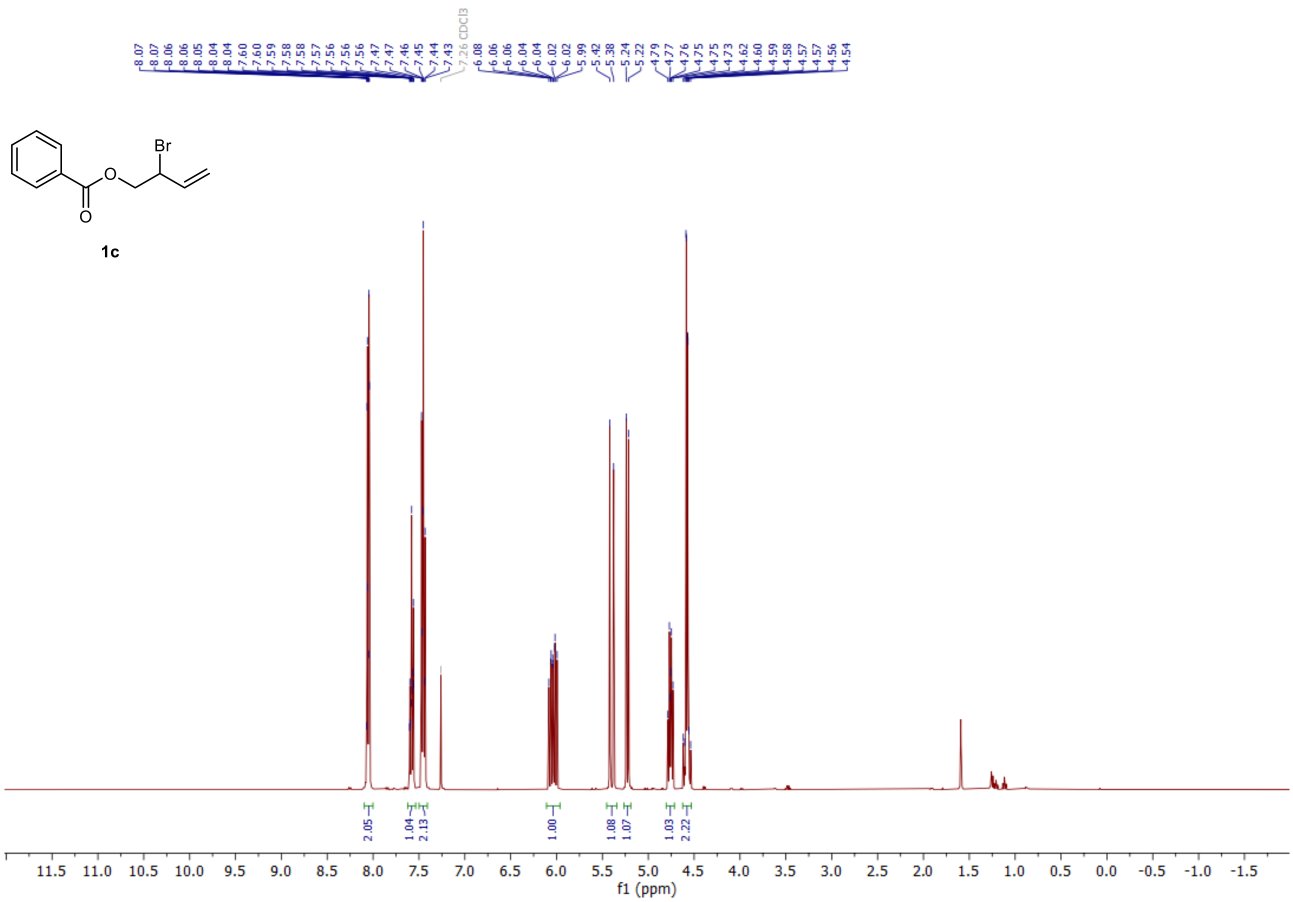

1c ^{13}C NMR (101 MHz, $CDCl_3$)

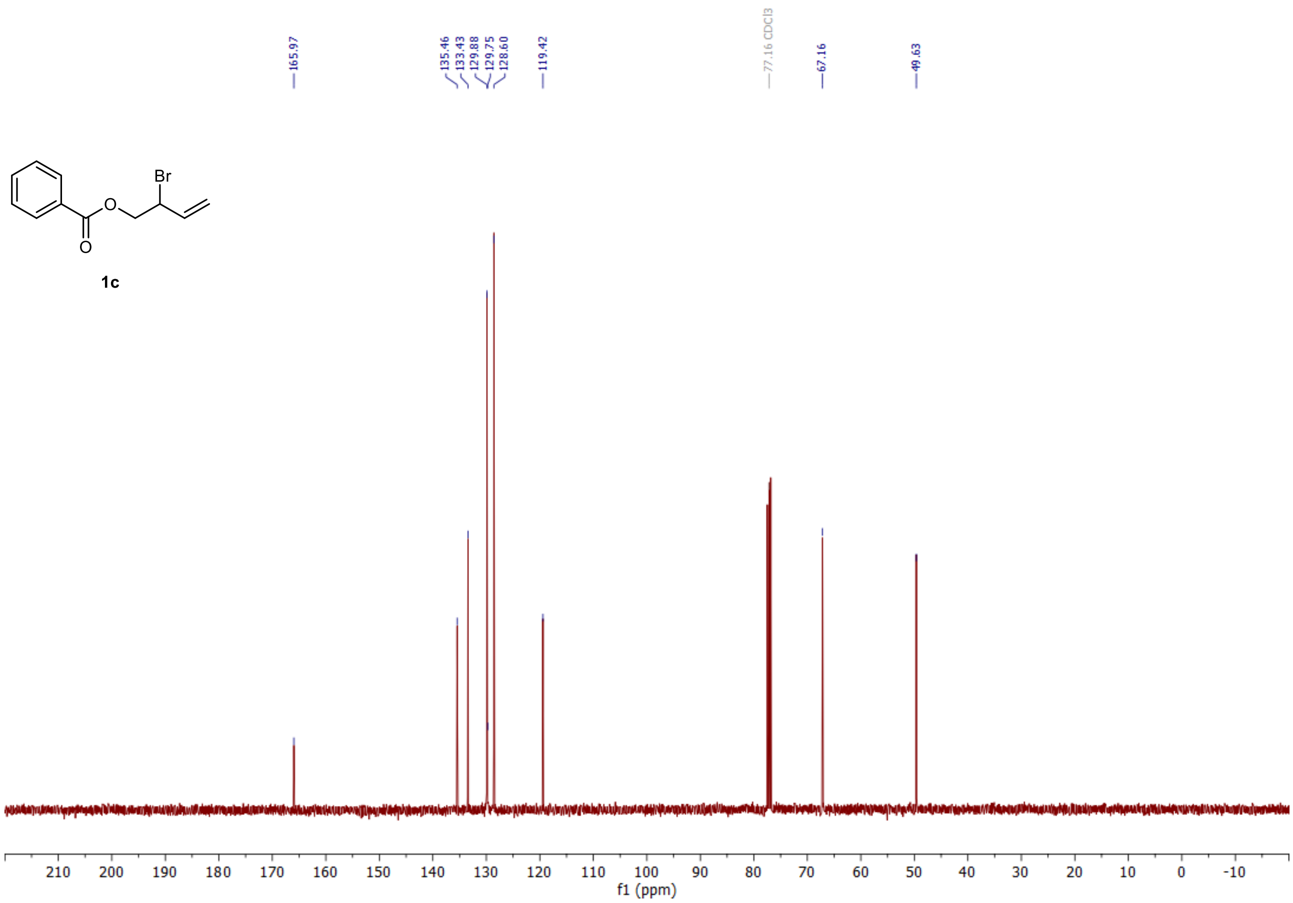

1d ^{1}H NMR (400 MHz, $CDCl_3$)

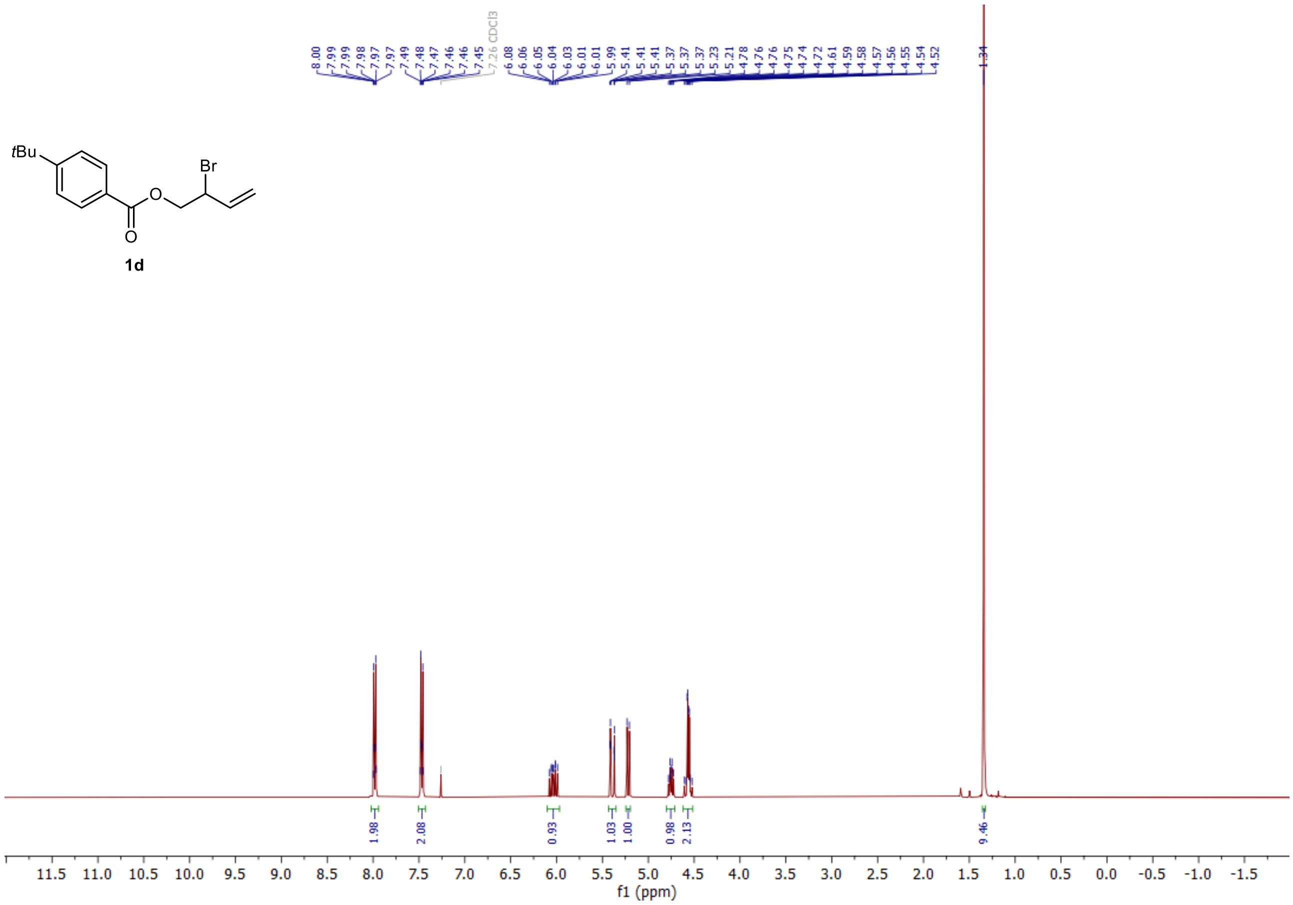

1d ^{13}C NMR (101 MHz, $CDCl_3$)

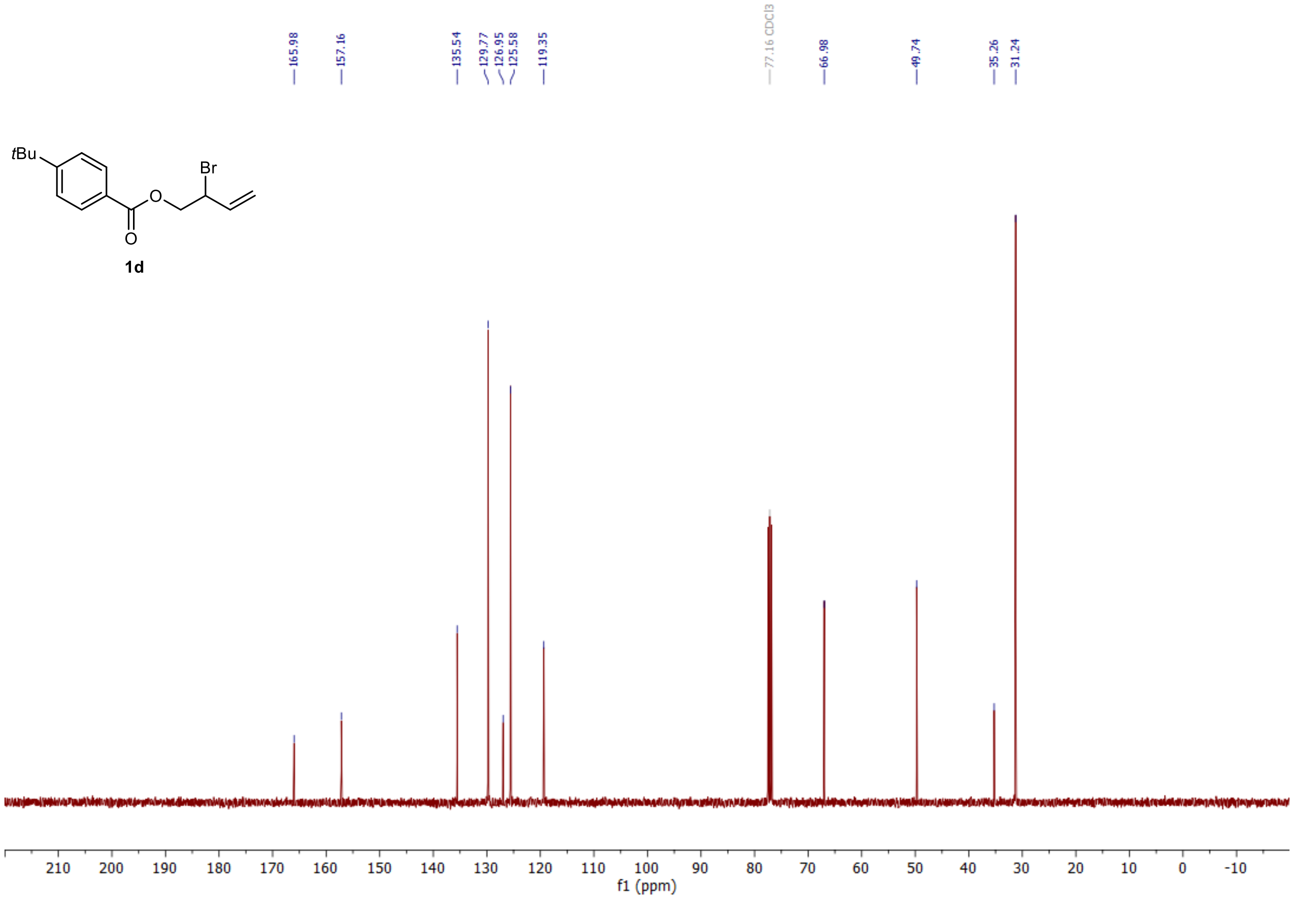

1e ^{1}H NMR (400 MHz, $CDCl_3$)

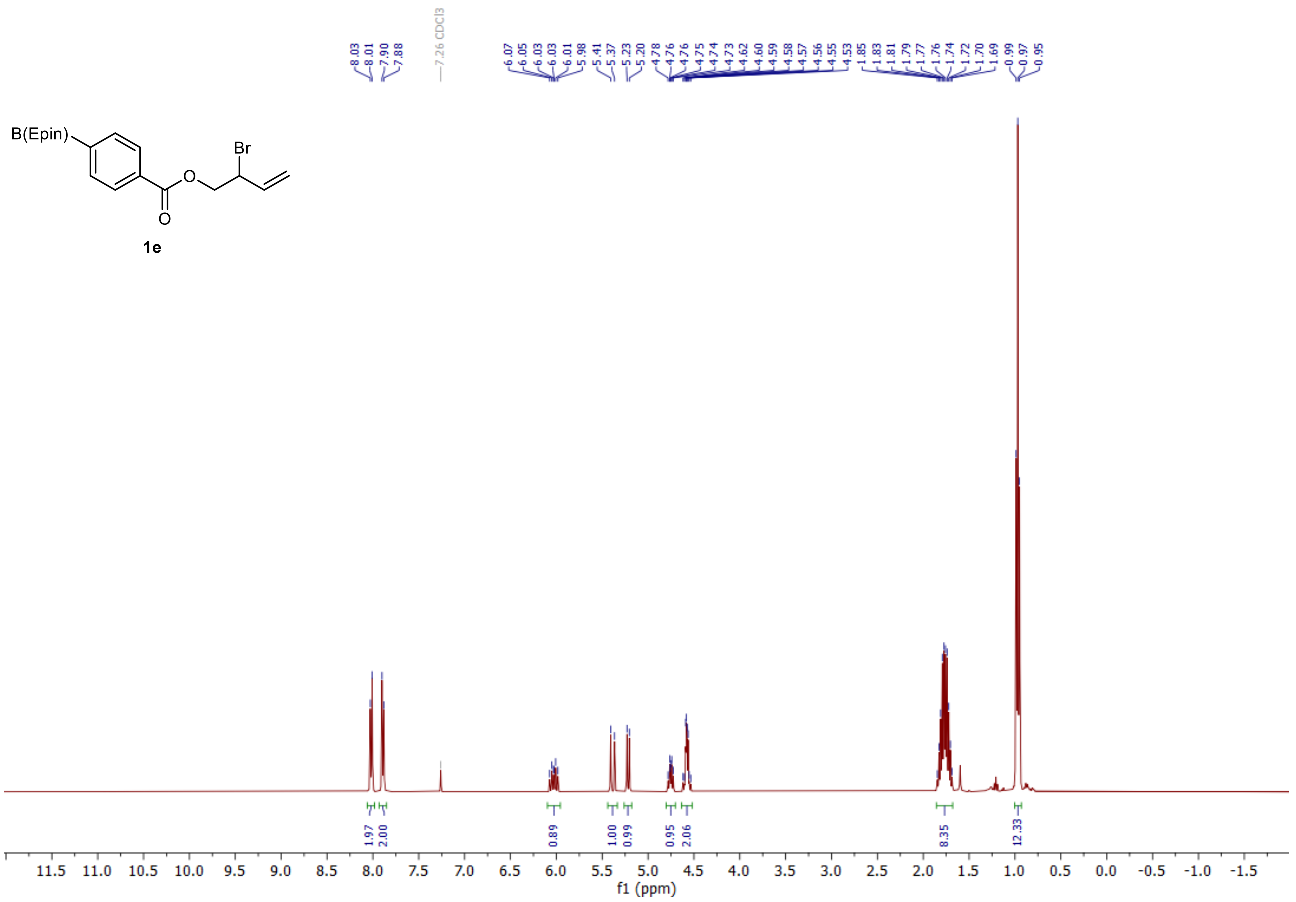

1e ^{13}C NMR (101 MHz, $CDCl_3$)

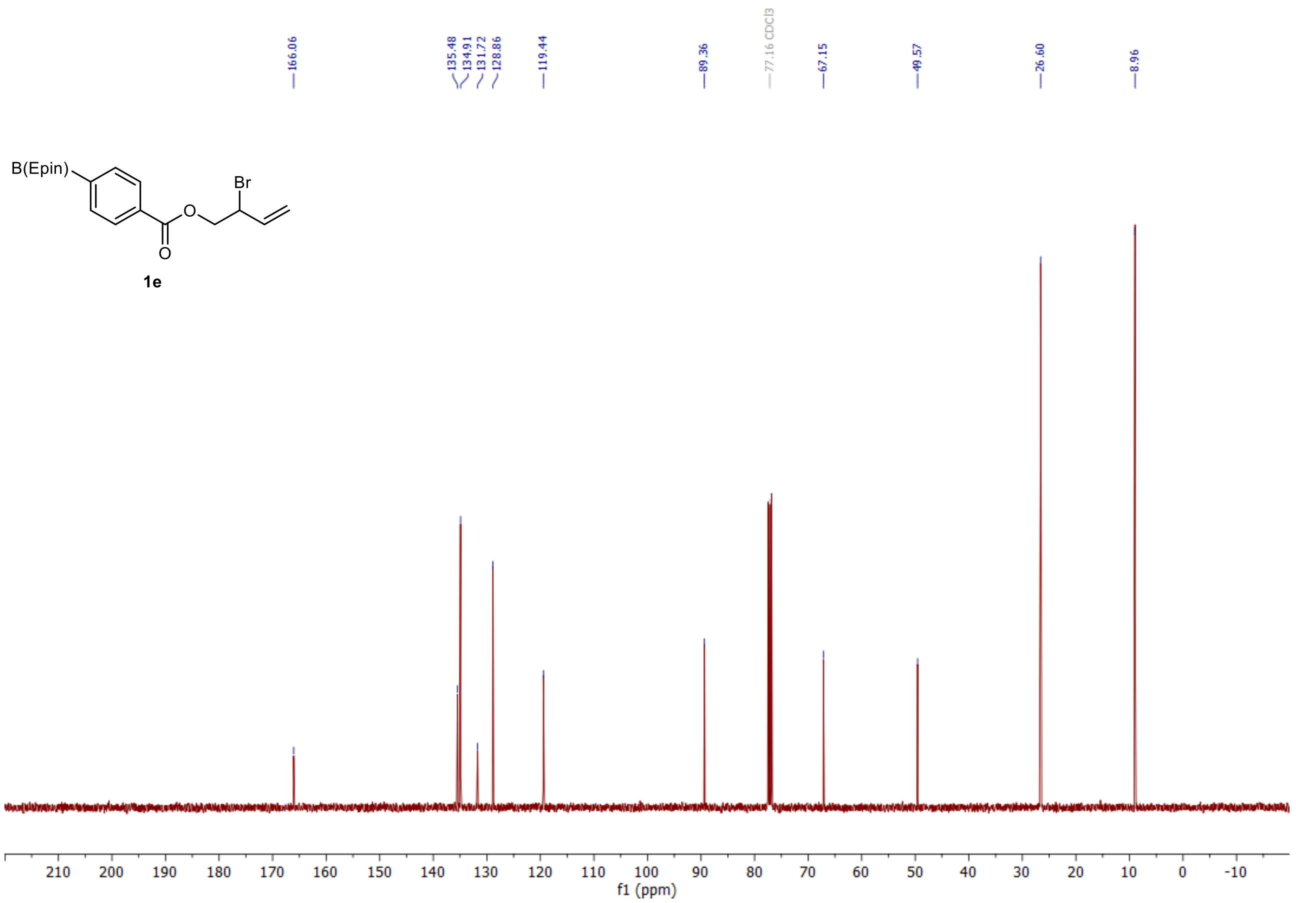

1f ^{1}H NMR (400 MHz, CDCl$_3$)

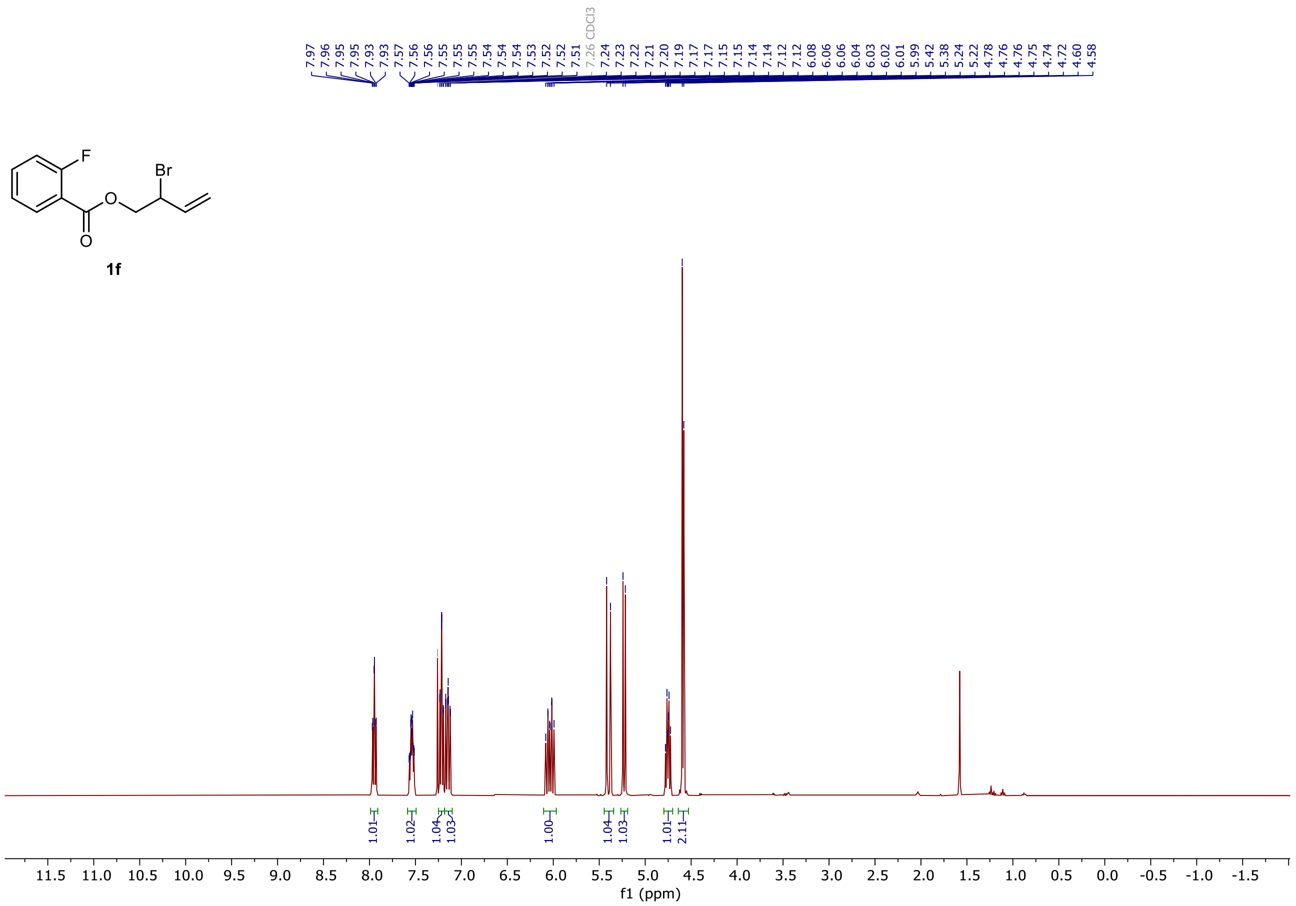

1f ^{13}C NMR (101 MHz, CDCl$_3$)

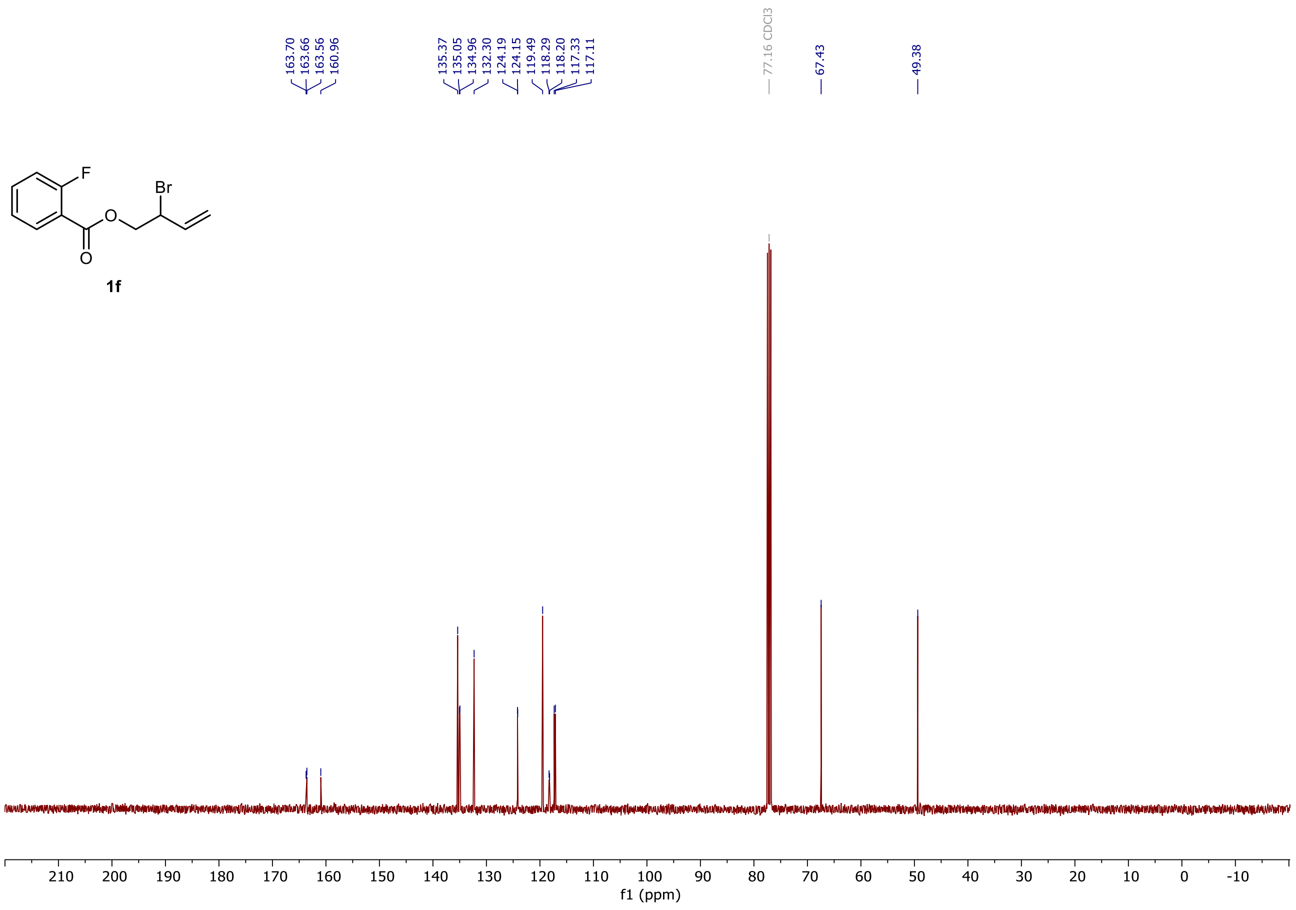

1f ^{19}F NMR (377 MHz, CDCl$_3$)

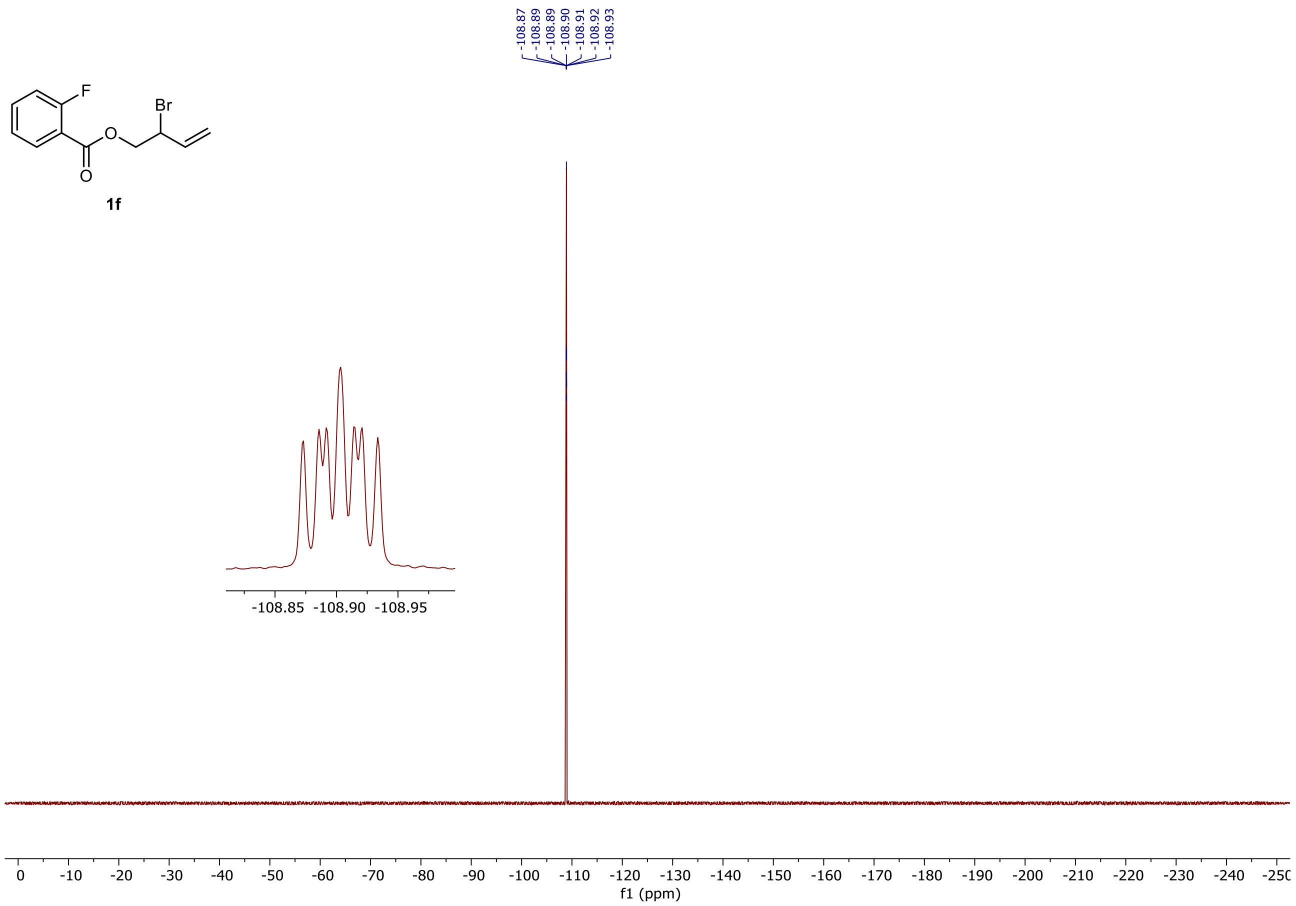

1g ^{1}H NMR (400 MHz, CDCl$_3$)

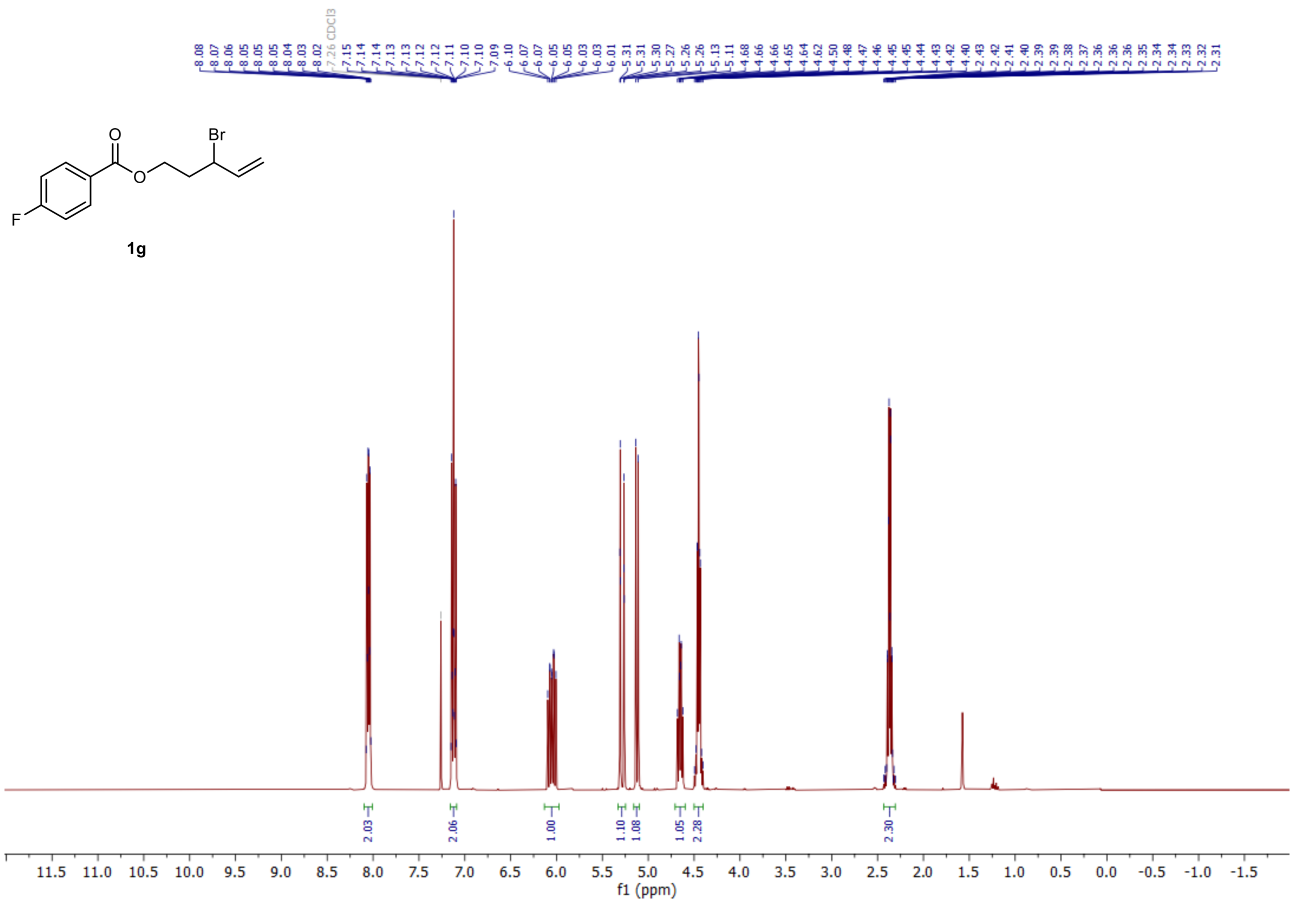

1g ^{13}C NMR (101 MHz, $CDCl_3$)

1g

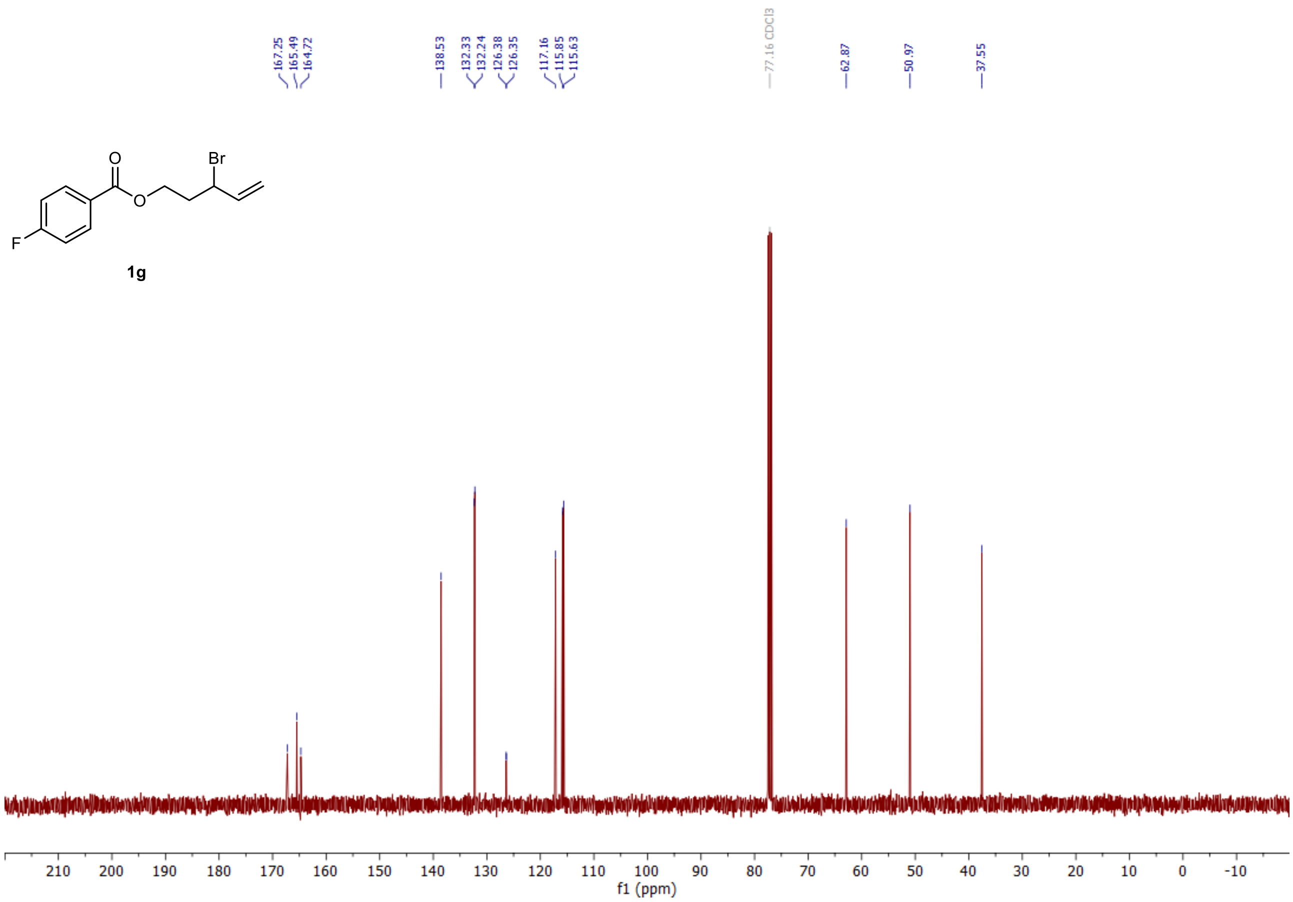

1g ^{19}F NMR (377 MHz, $CDCl_3$)

1g

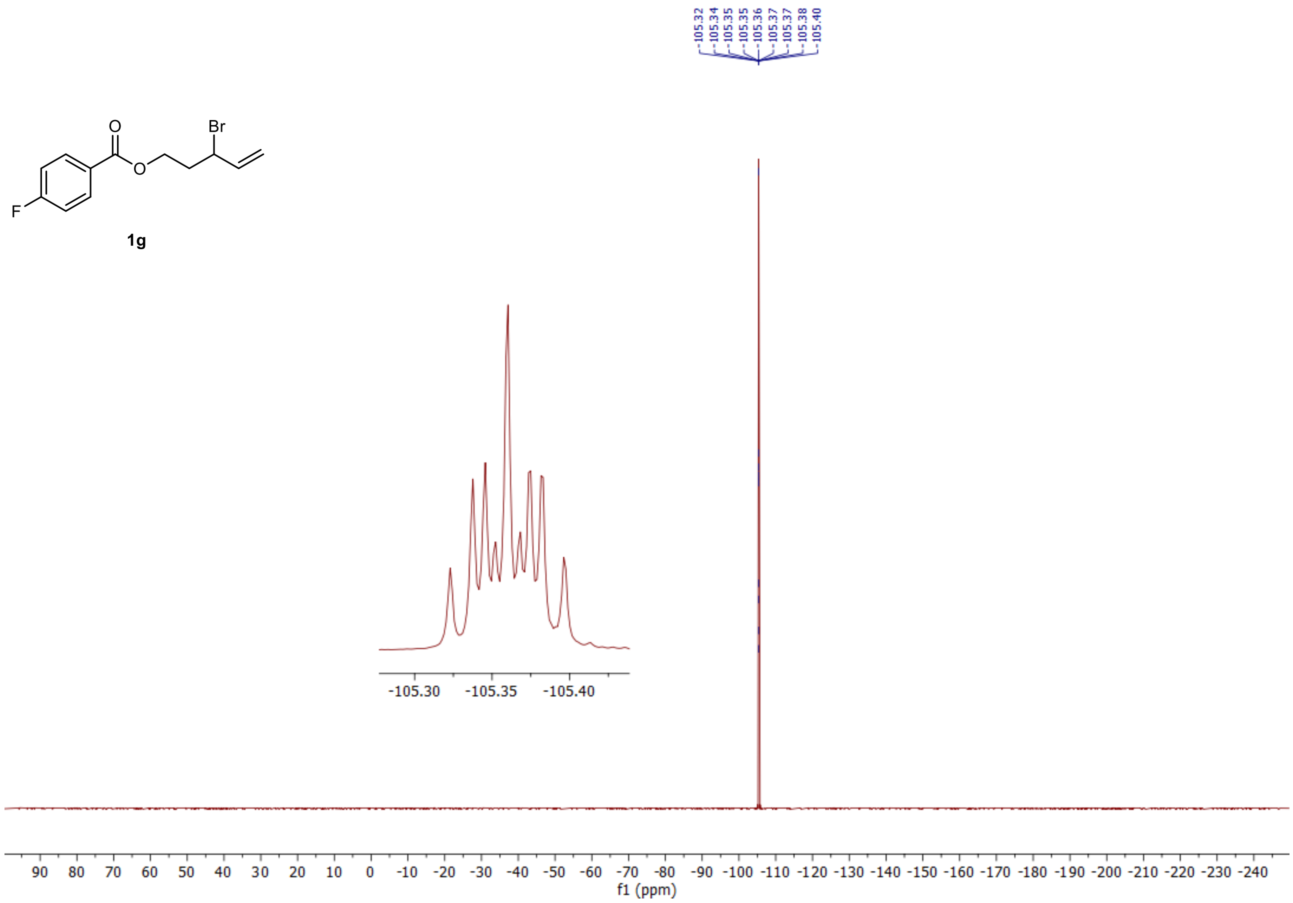

1h ^{1}H NMR (400 MHz, $CDCl_3$)

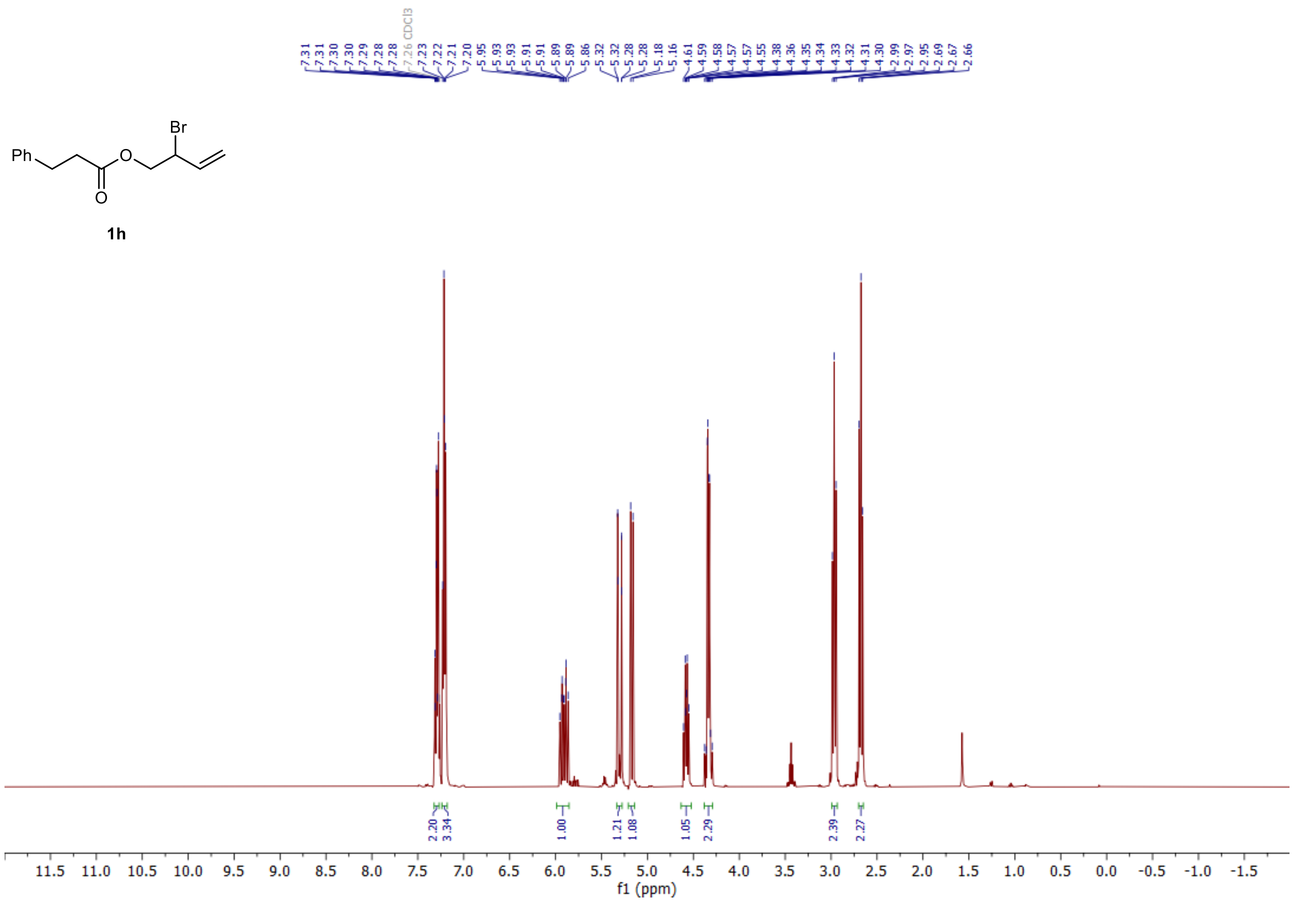

1h ^{13}C NMR (101 MHz, $CDCl_3$)

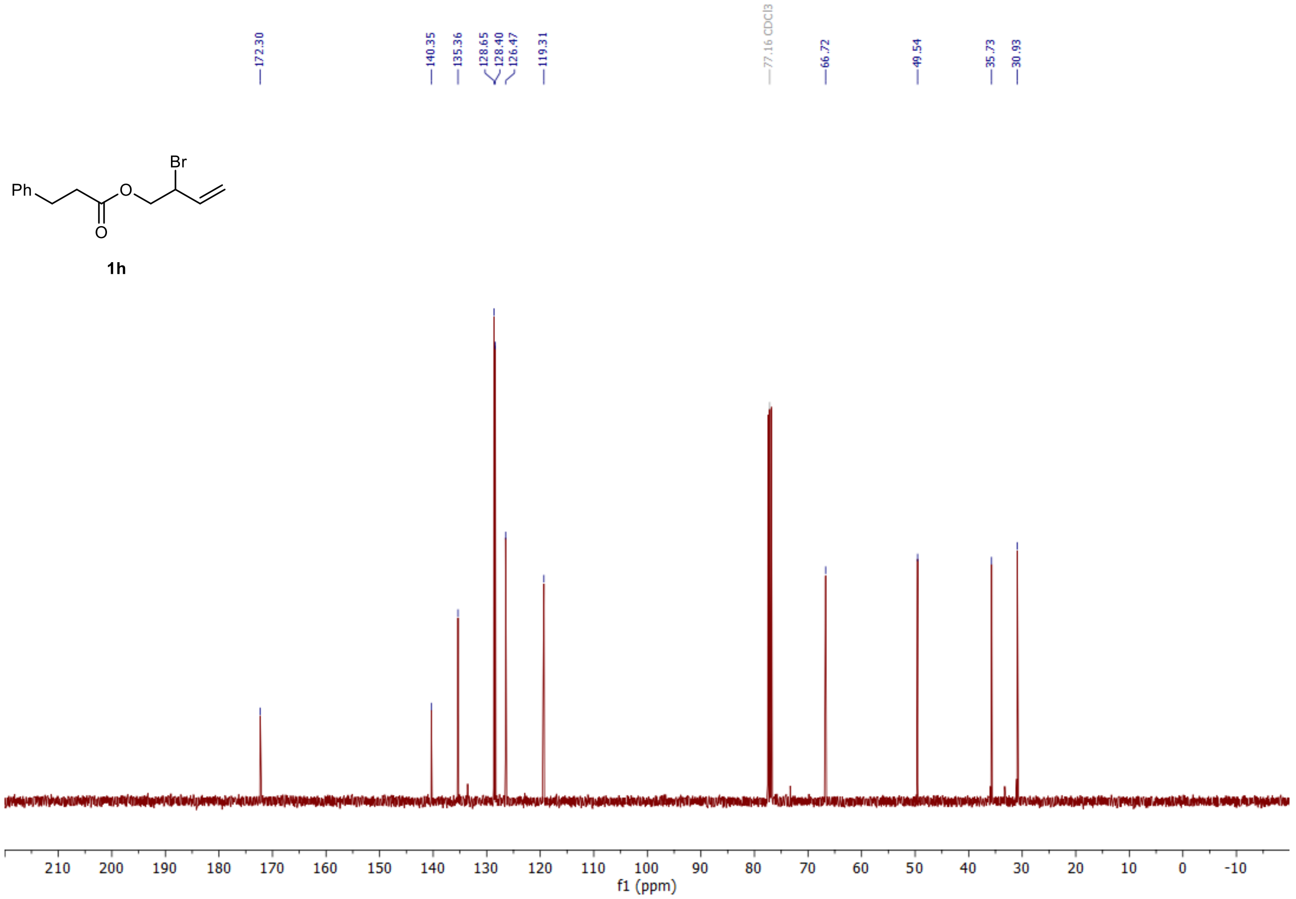

1i ^{1}H NMR (400 MHz, $CDCl_3$)

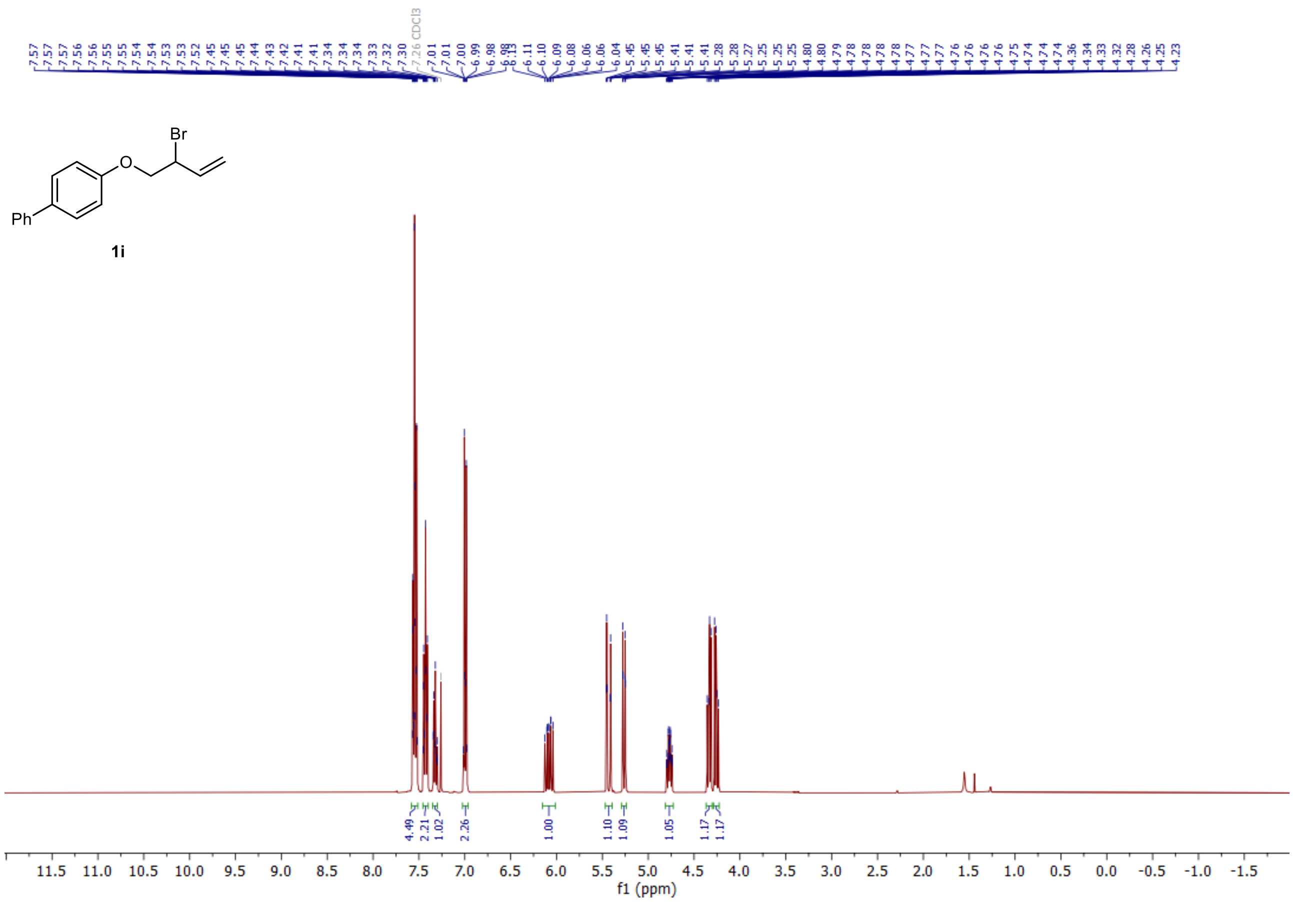

1i ^{13}C NMR (101 MHz, $CDCl_3$)

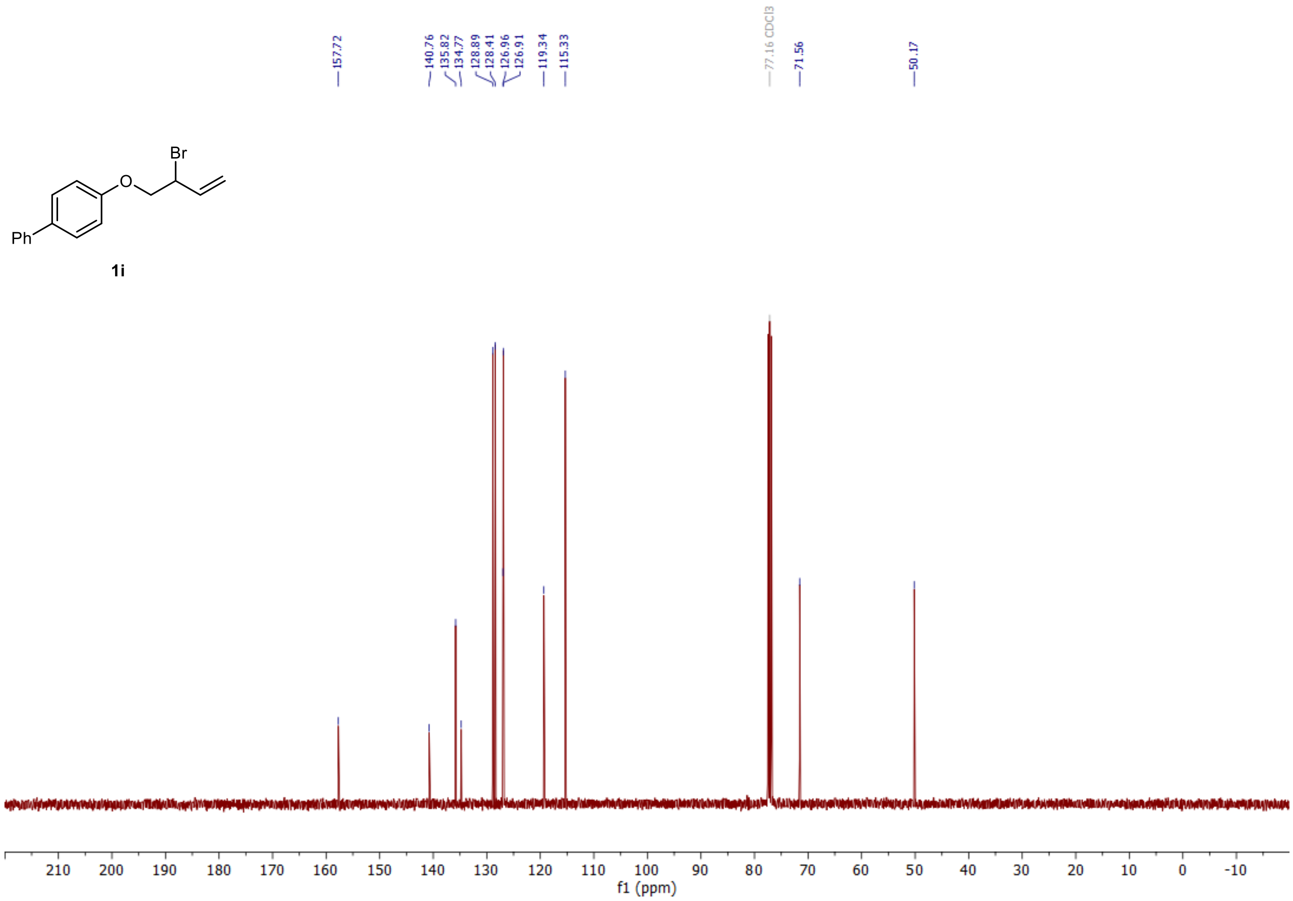

1j ^{1}H NMR (400 MHz, $CDCl_3$)

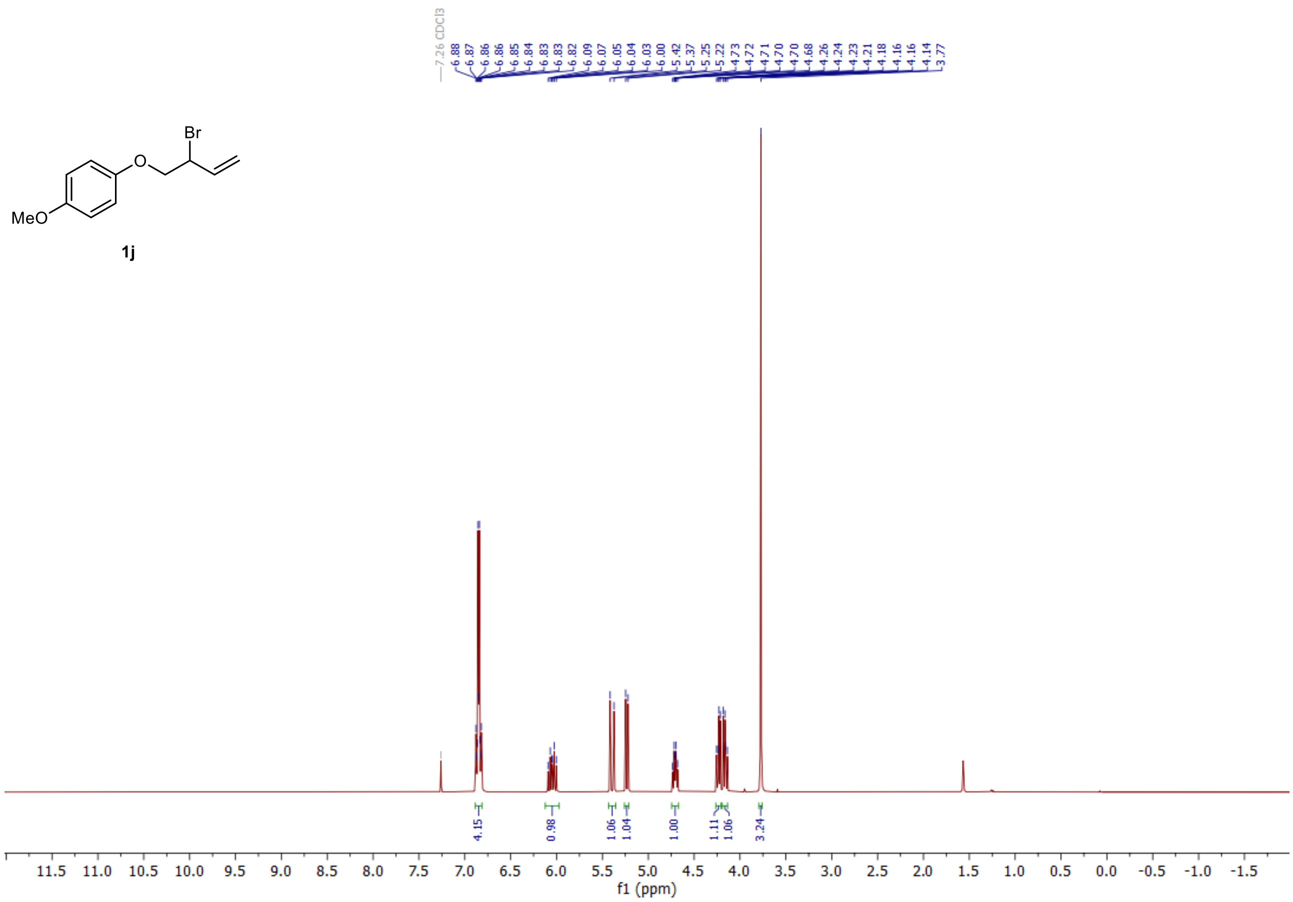

1j ^{13}C NMR (101 MHz, $CDCl_3$)

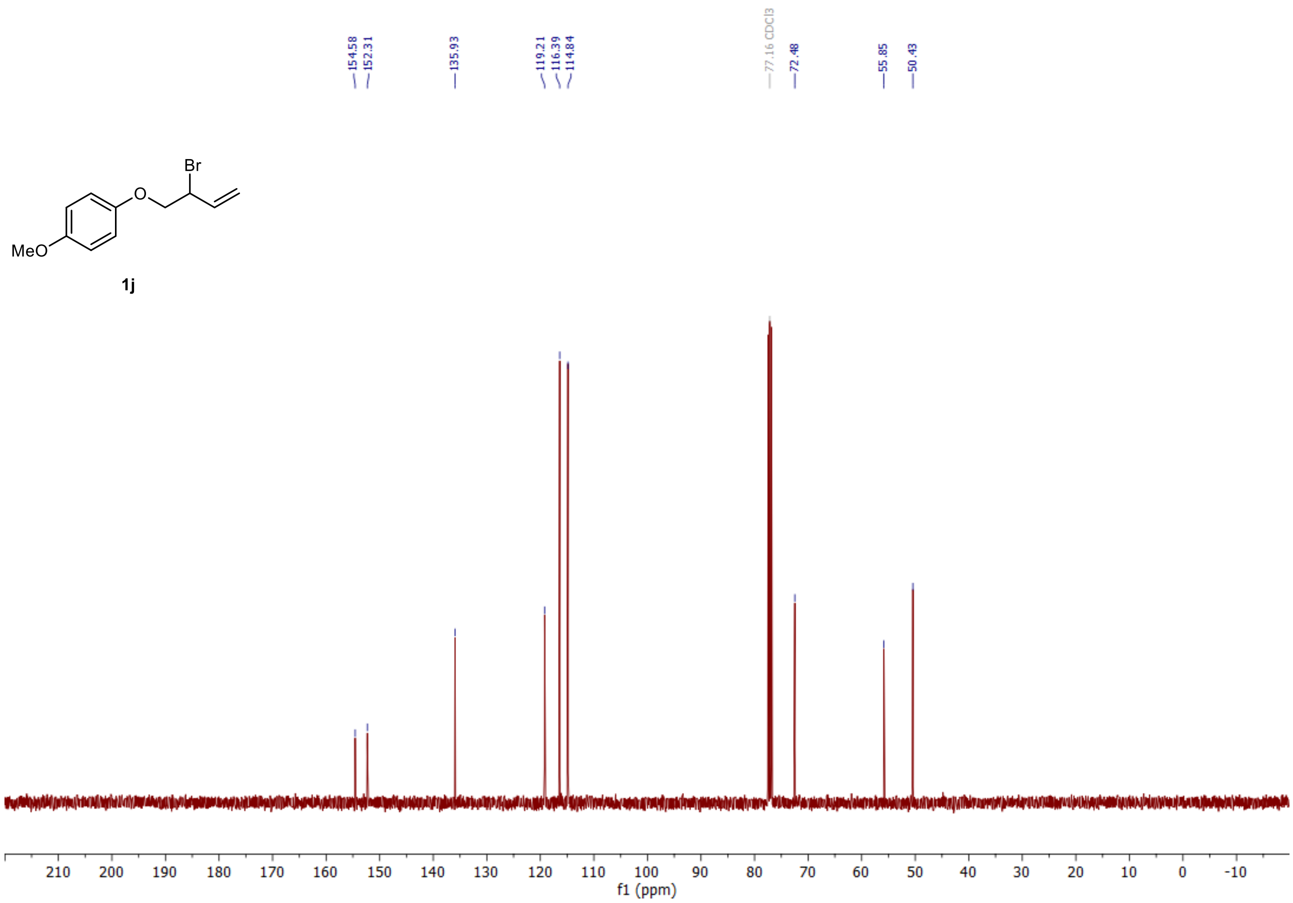

1k ^{1}H NMR (400 MHz, $CDCl_3$)

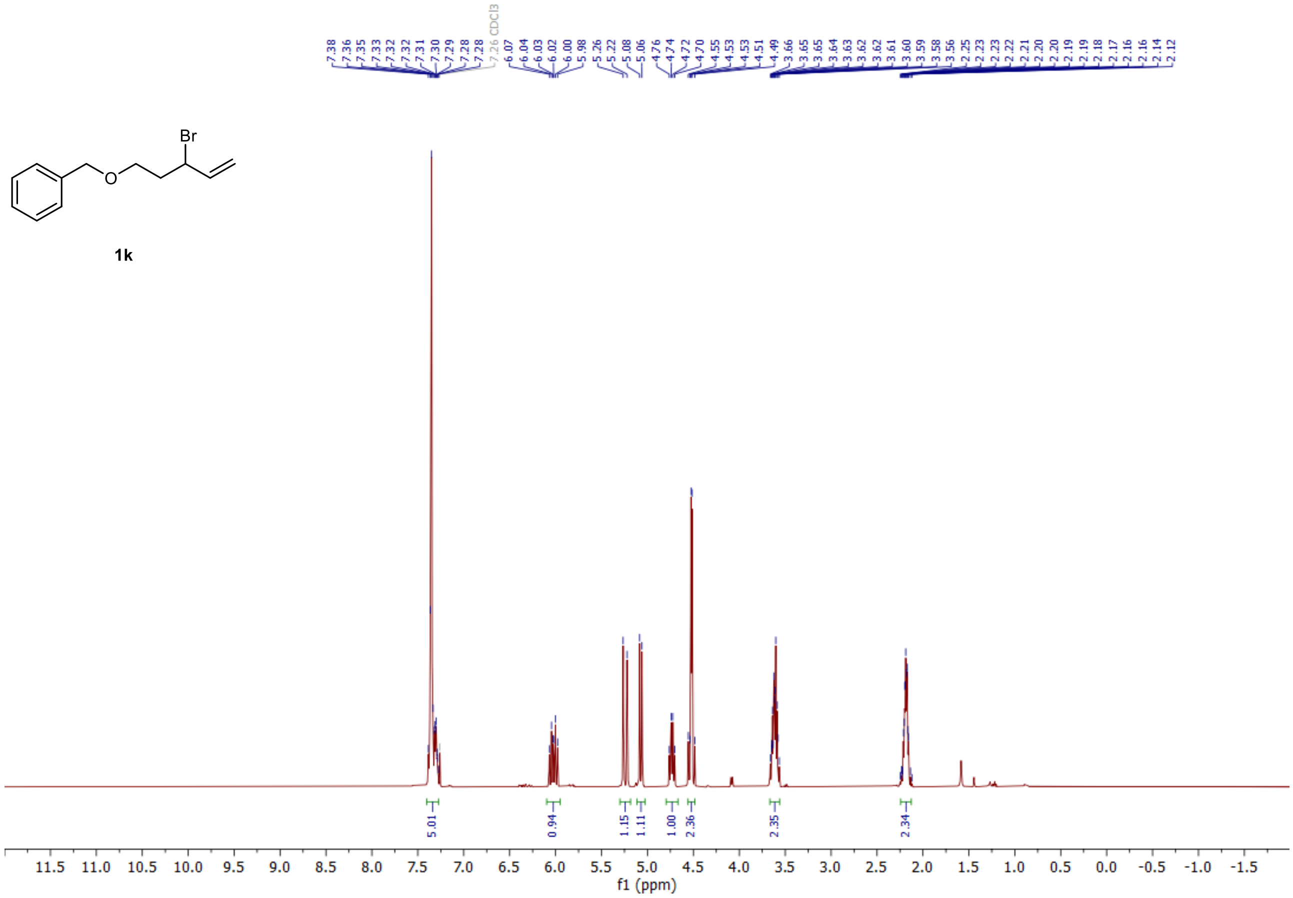

1k ^{13}C NMR (101 MHz, $CDCl_3$)

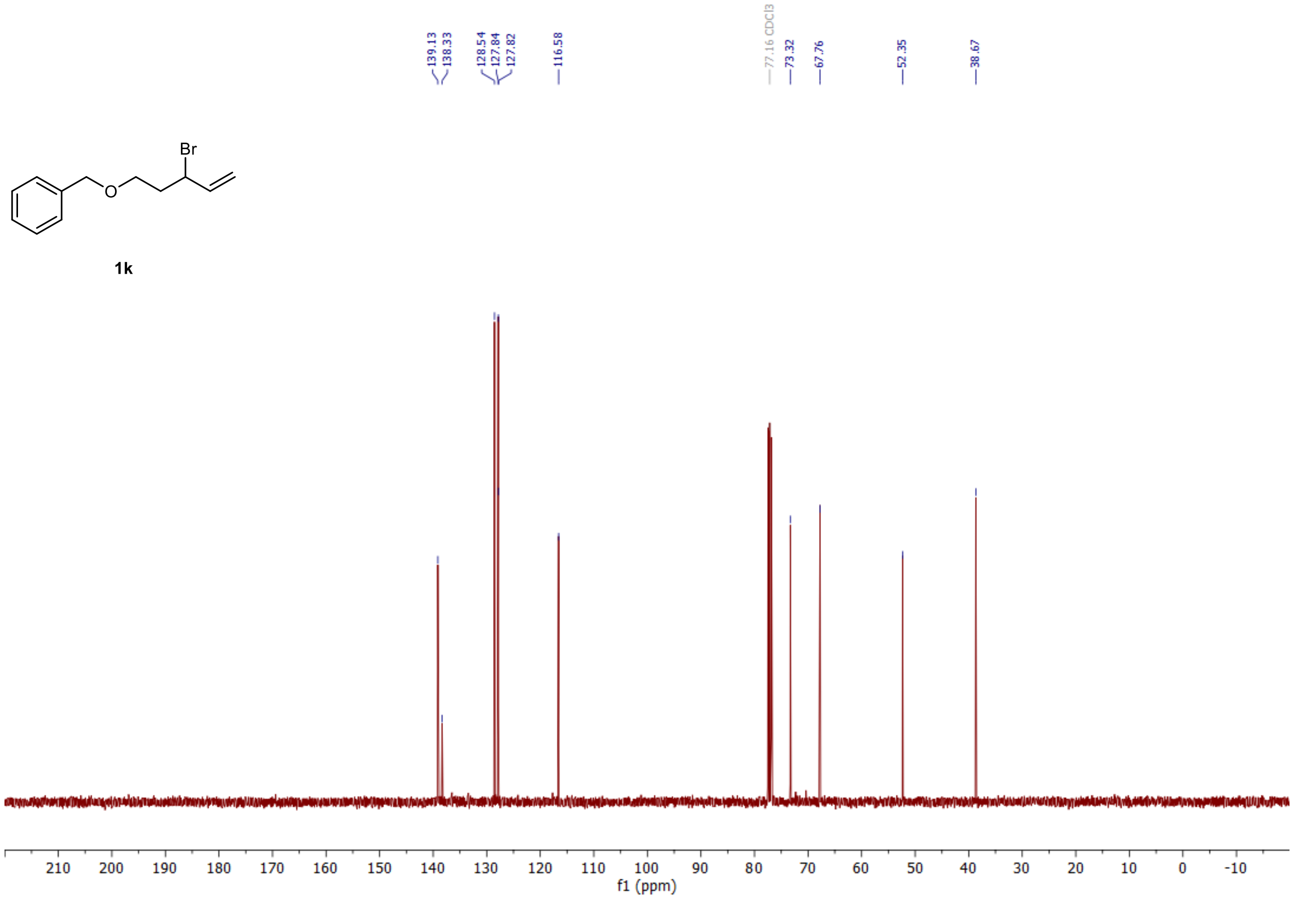

1l ^{1}H NMR (400 MHz, $CDCl_3$)

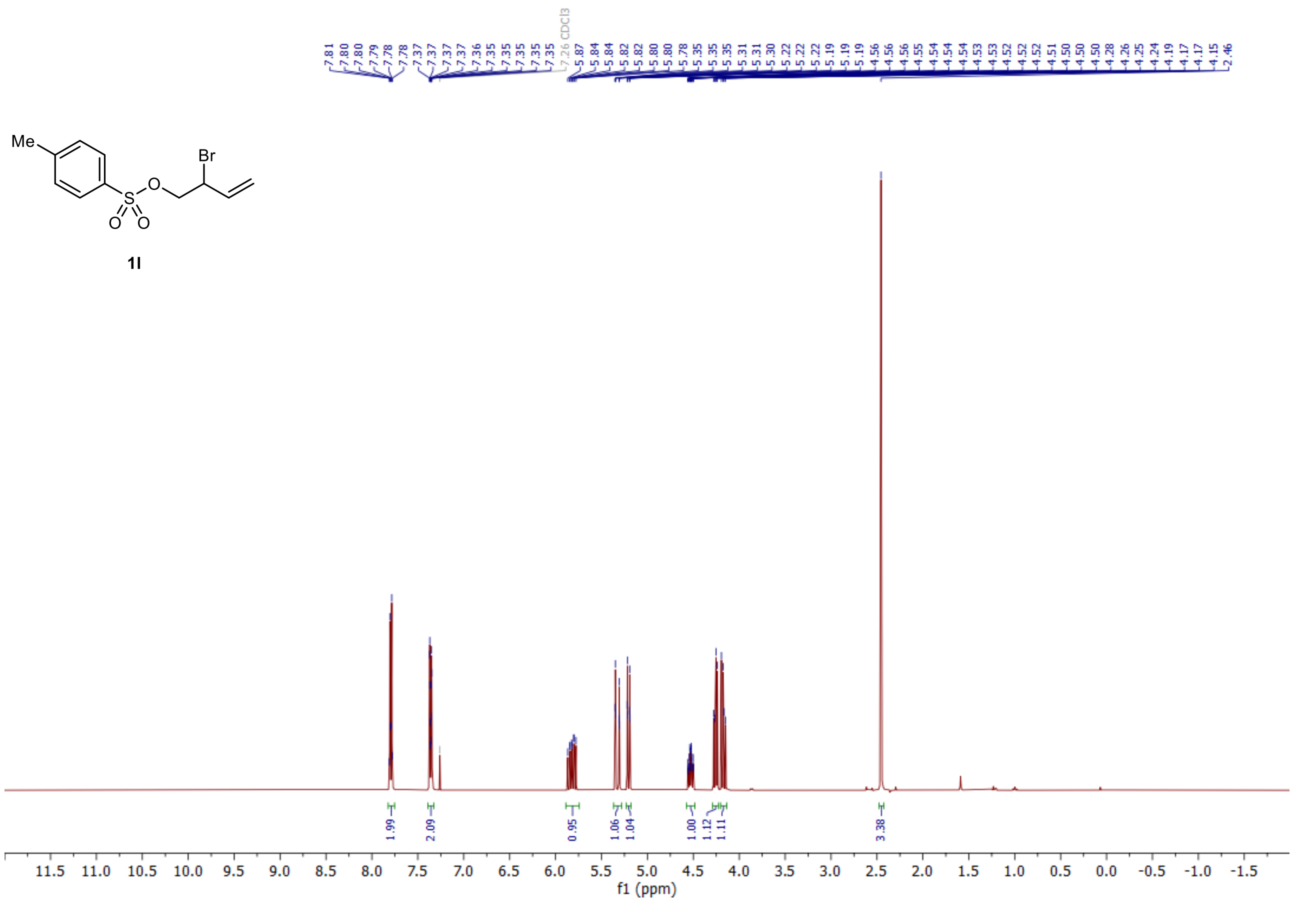

1l ^{13}C NMR (101 MHz, $CDCl_3$)

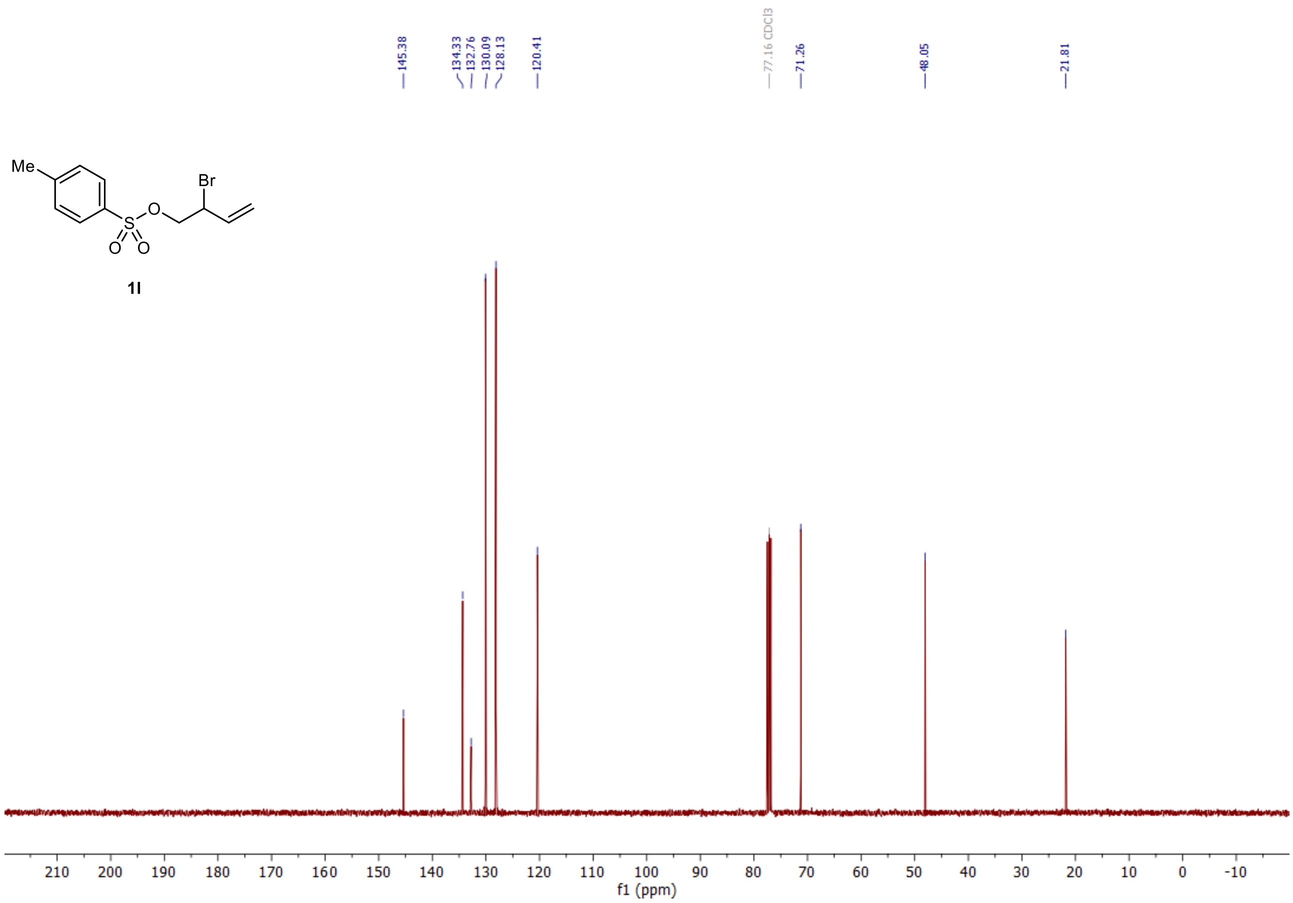

1m ^{1}H NMR (400 MHz, $CDCl_3$)

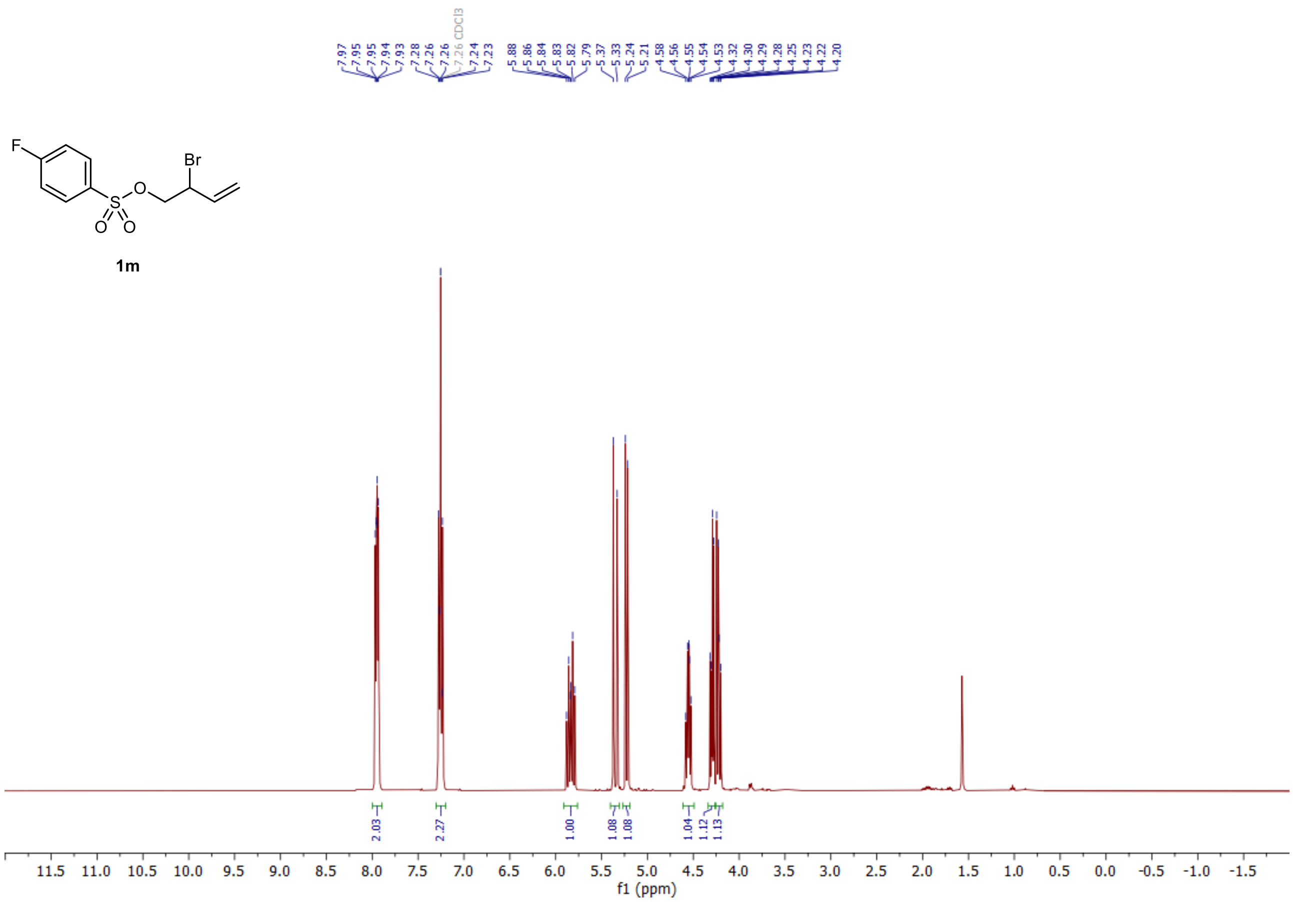

1m ^{13}C NMR (101 MHz, $CDCl_3$)

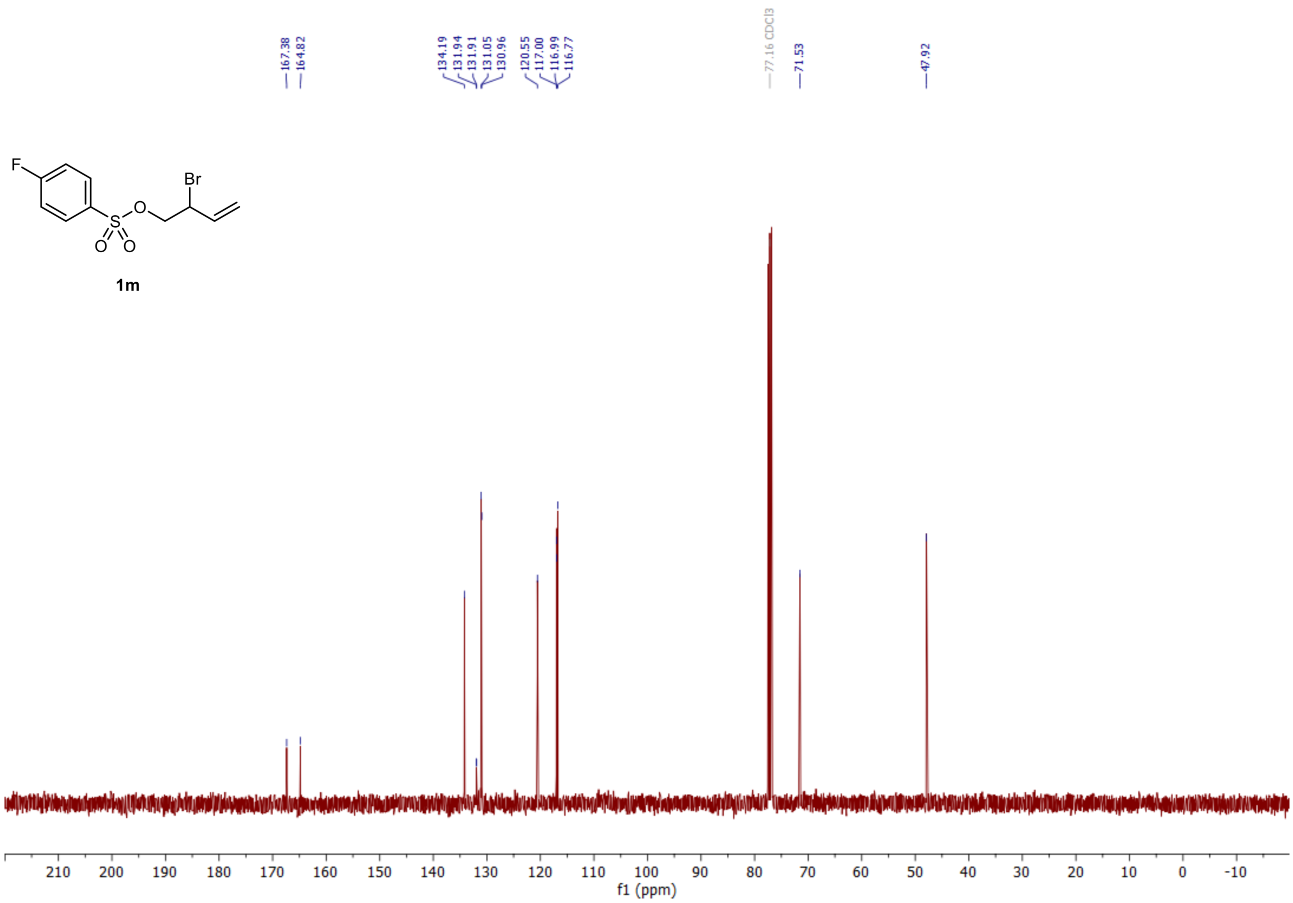

1m ^{19}F NMR (377 MHz, CDCl$_3$)

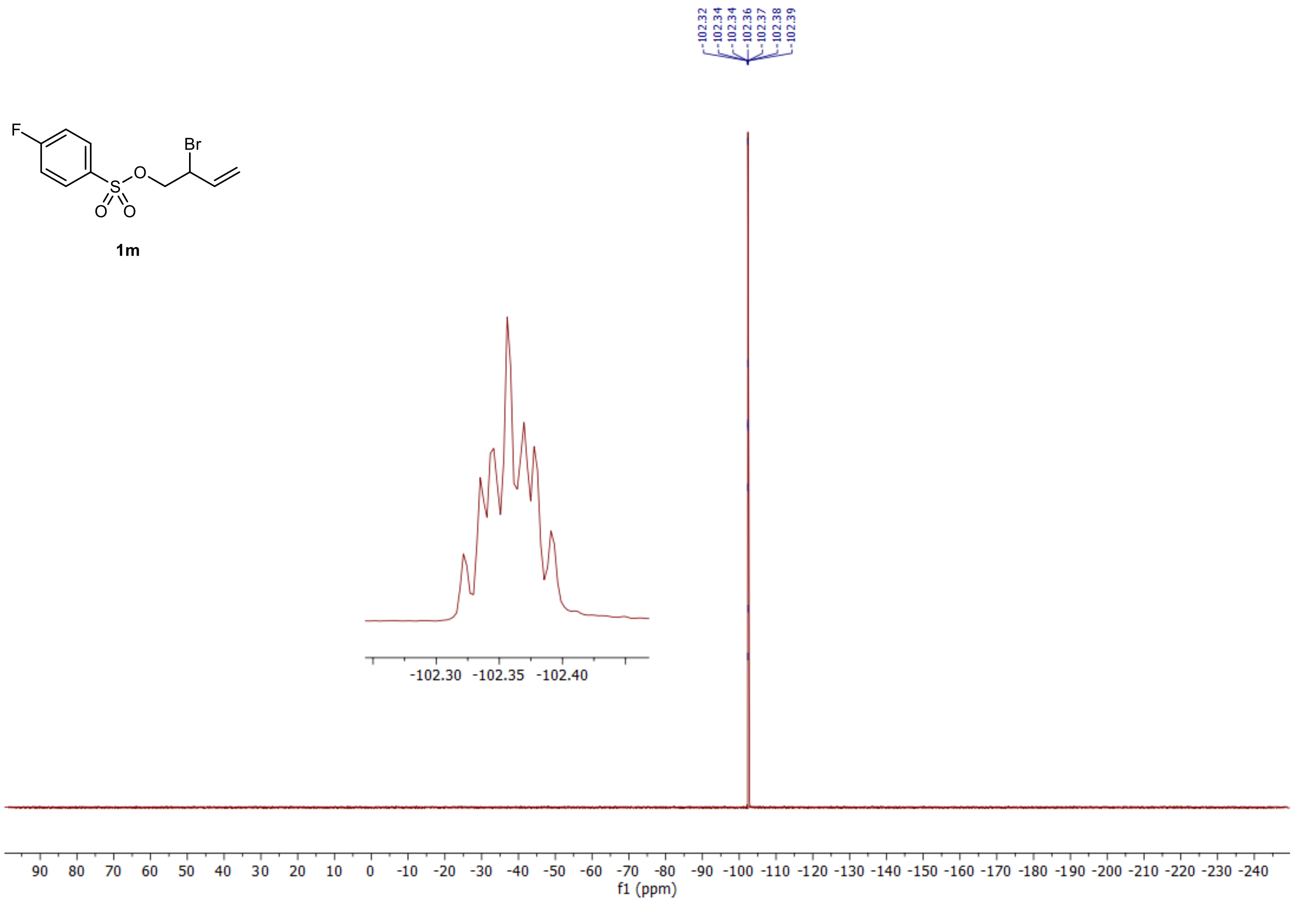

1n ^{1}H NMR (400 MHz, CDCl$_3$)

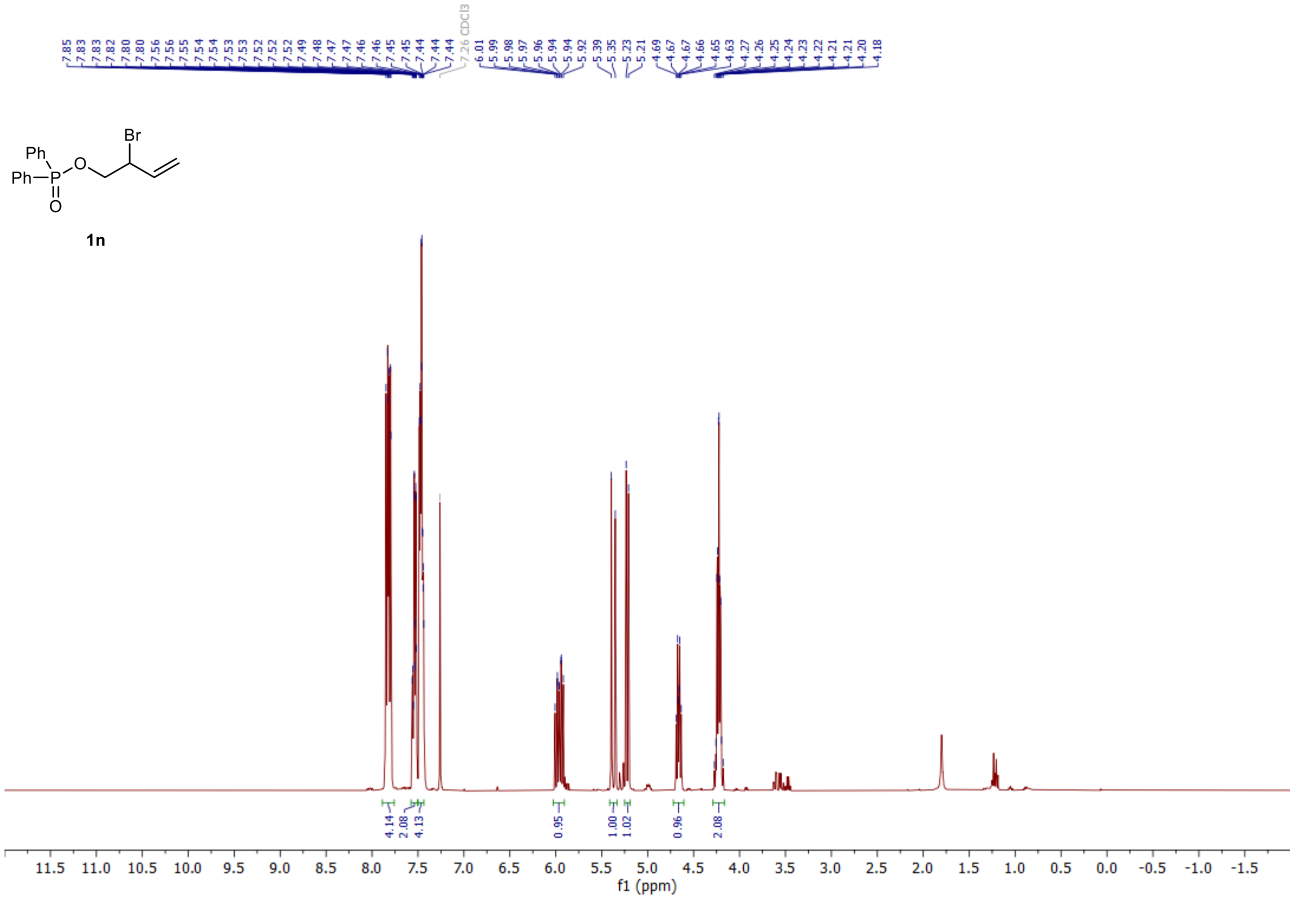

1n ^{13}C NMR (101 MHz, $CDCl_3$)

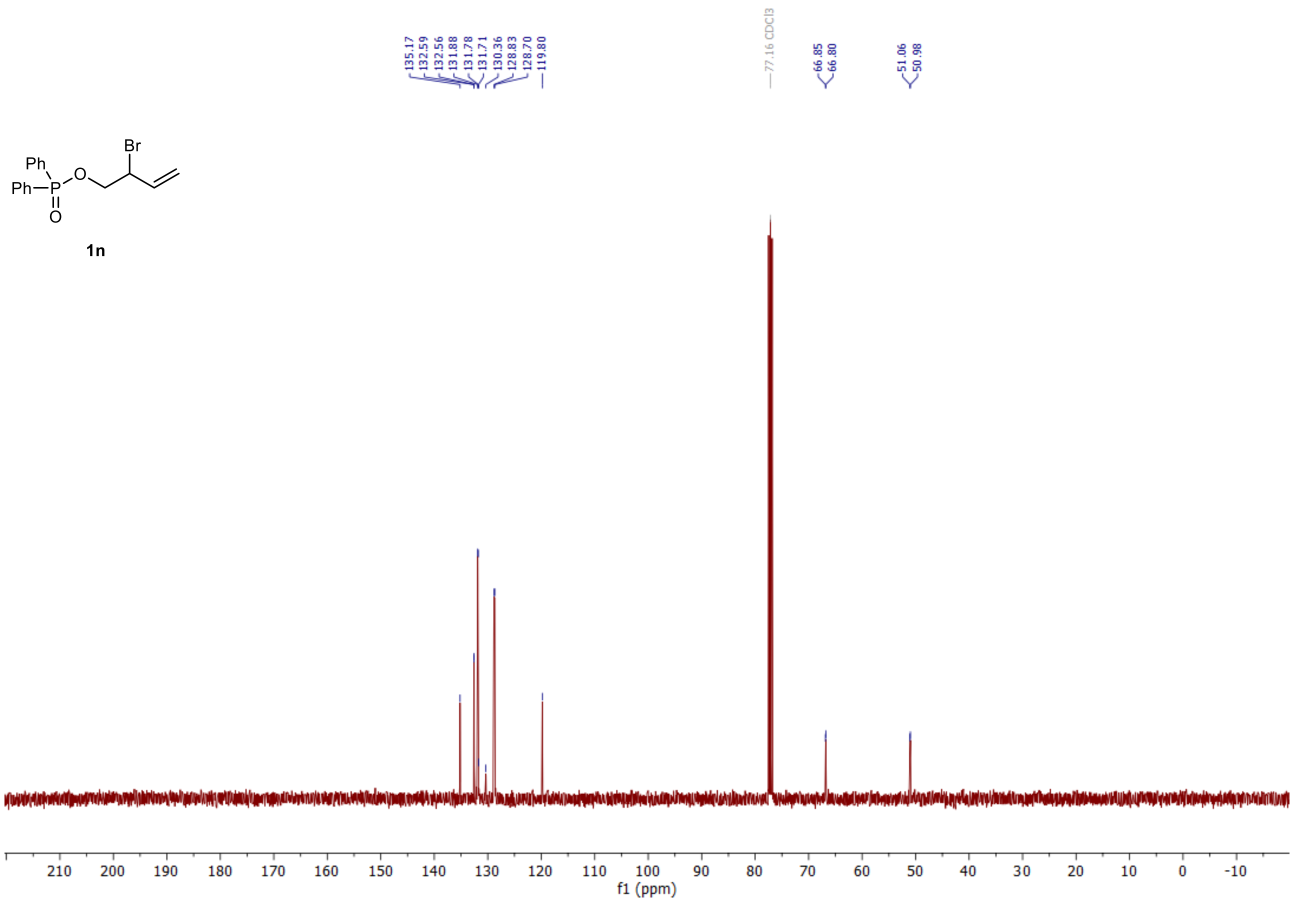

1n ^{31}P NMR (162 MHz, $CDCl_3$)

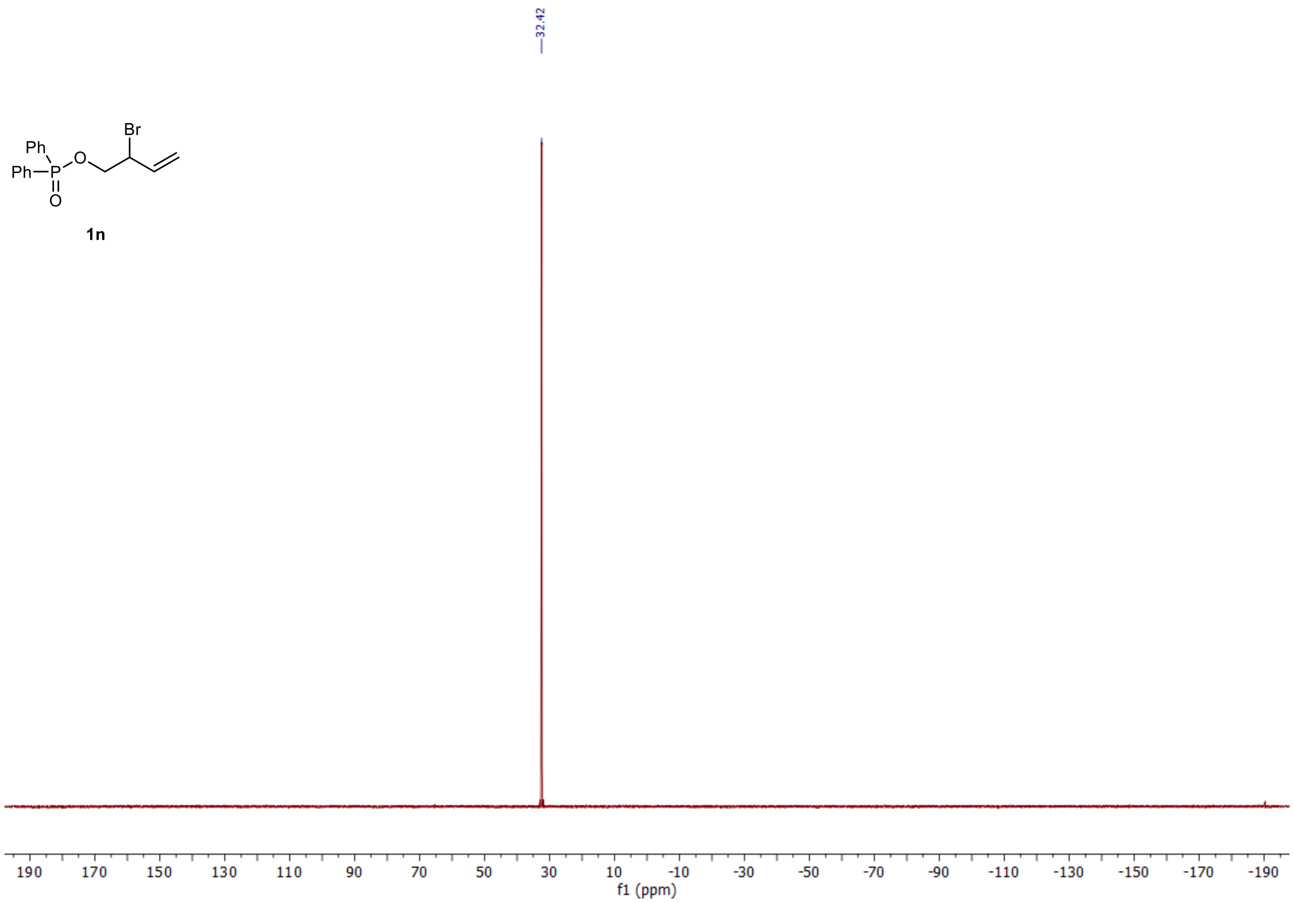

1o ^{1}H NMR (400 MHz, $CDCl_3$)

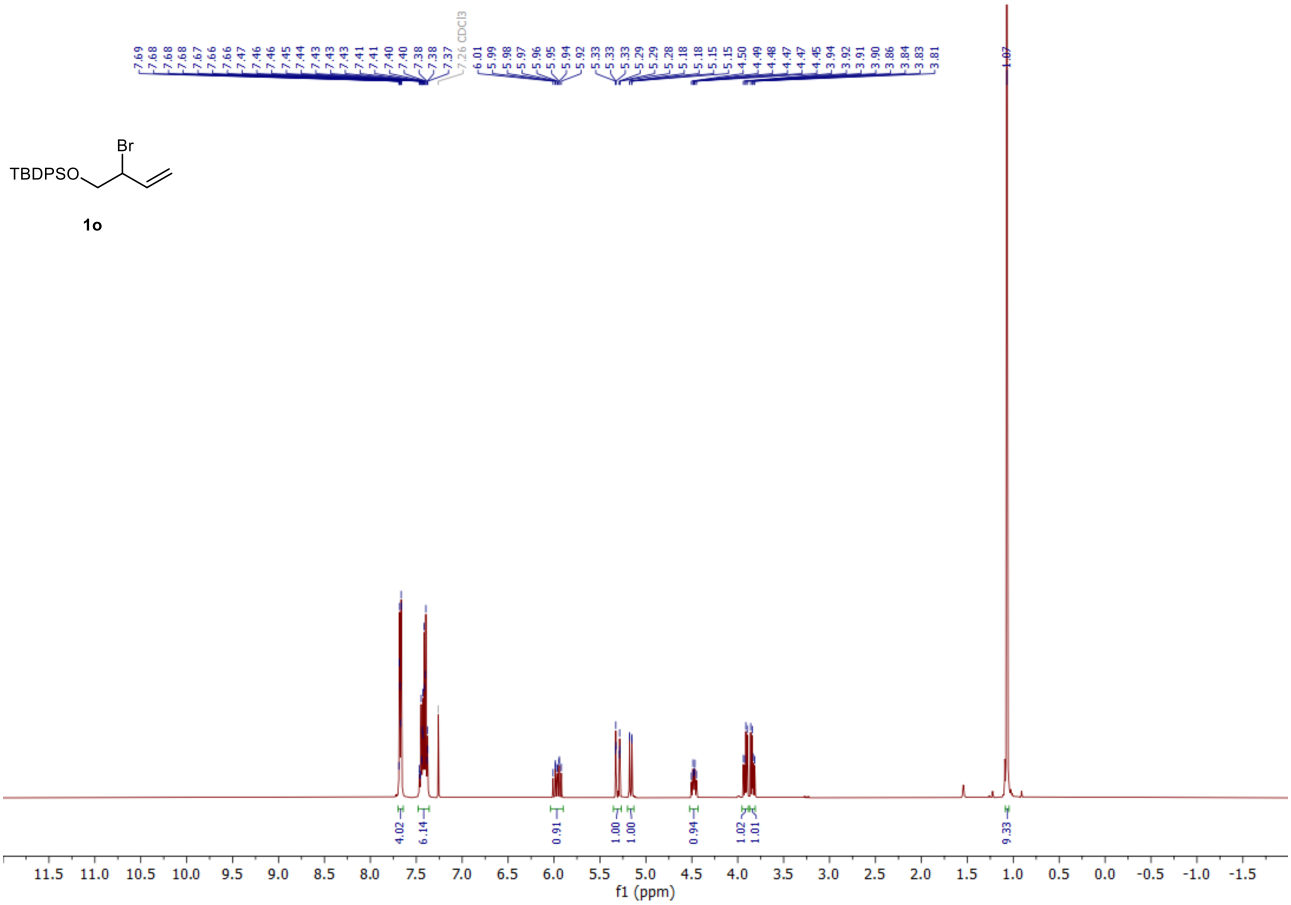

1o ^{13}C NMR (101 MHz, $CDCl_3$)

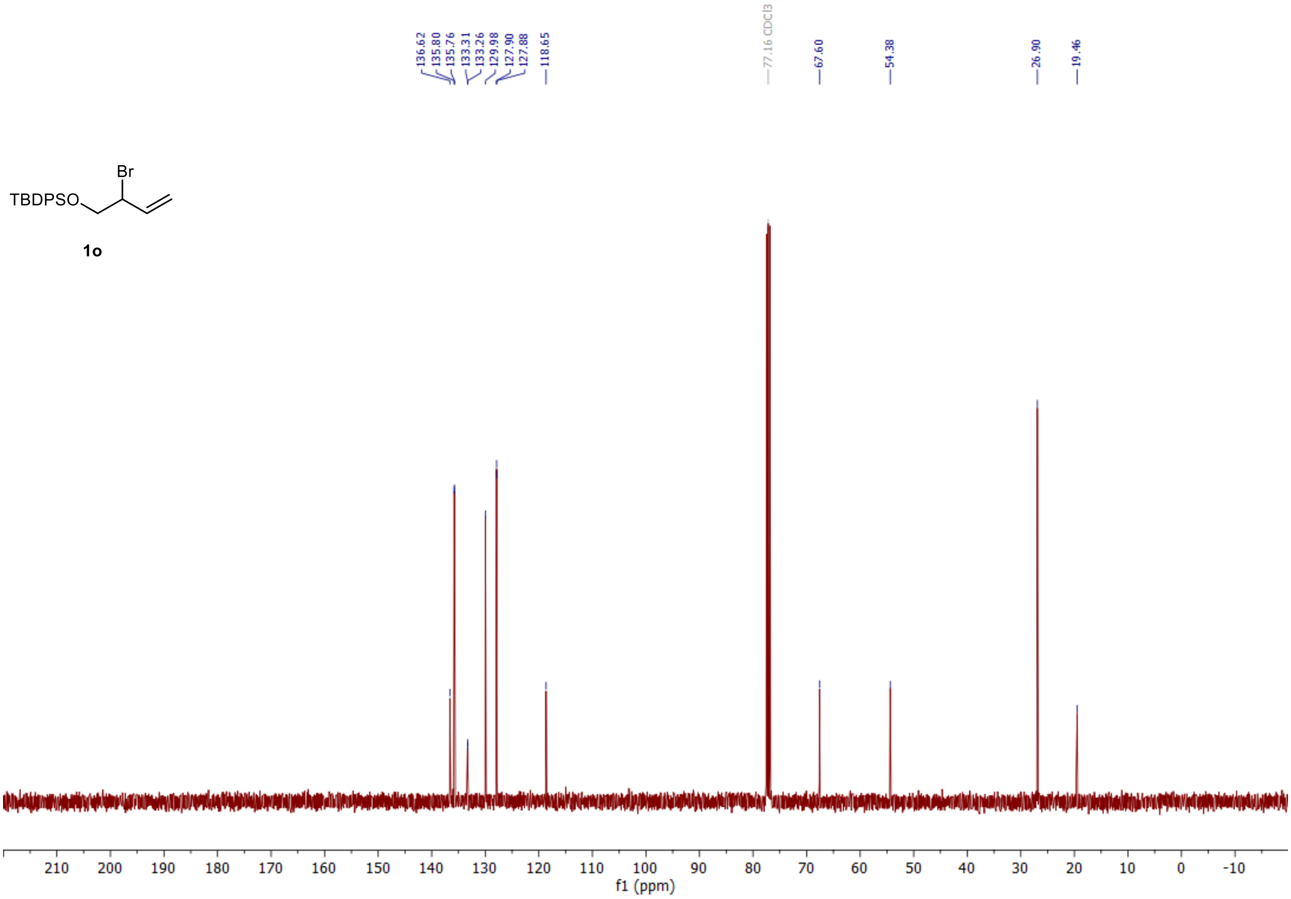

1p ^{1}H NMR (400 MHz, $CDCl_3$)

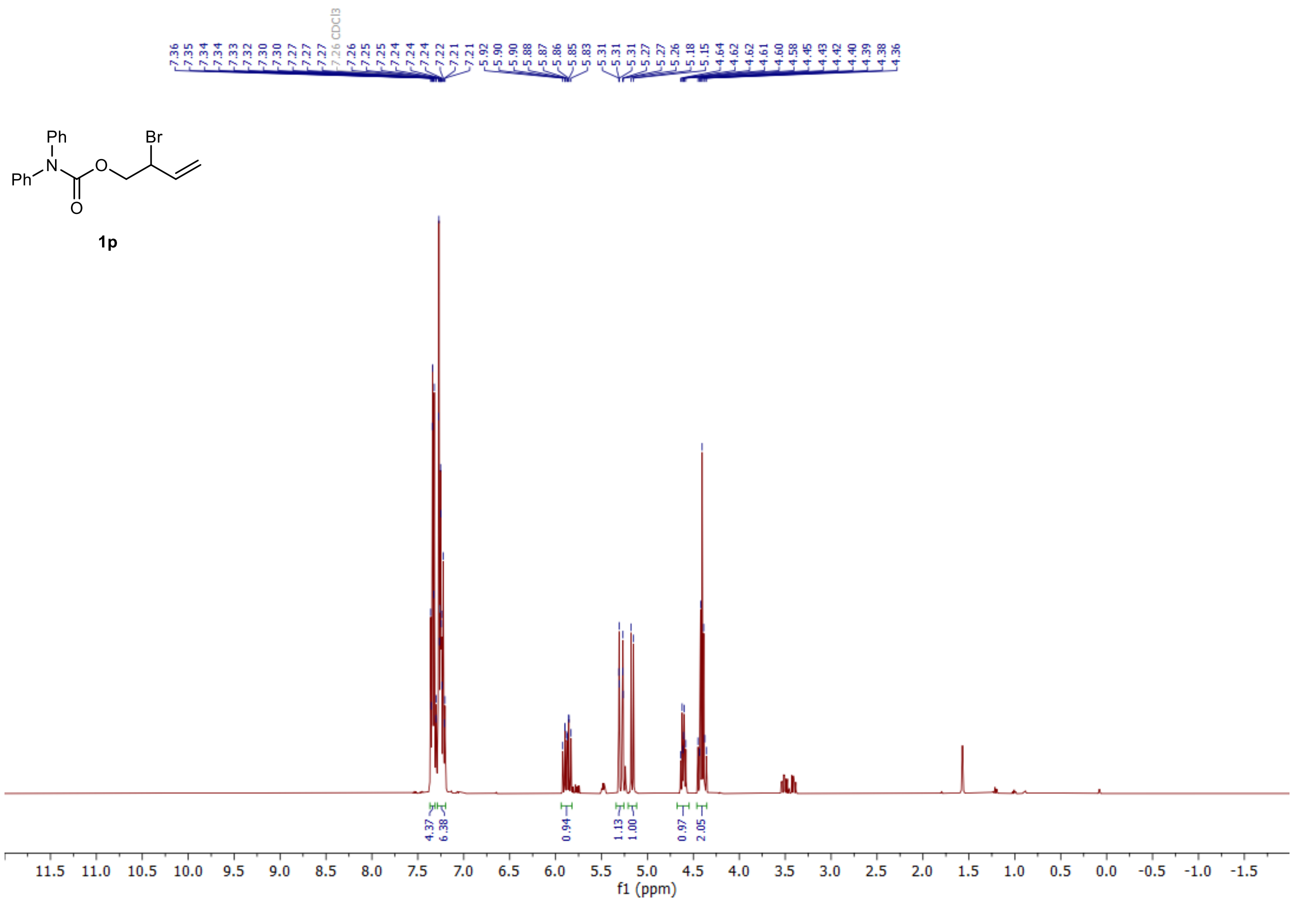

1p ^{13}C NMR (101 MHz, $CDCl_3$)

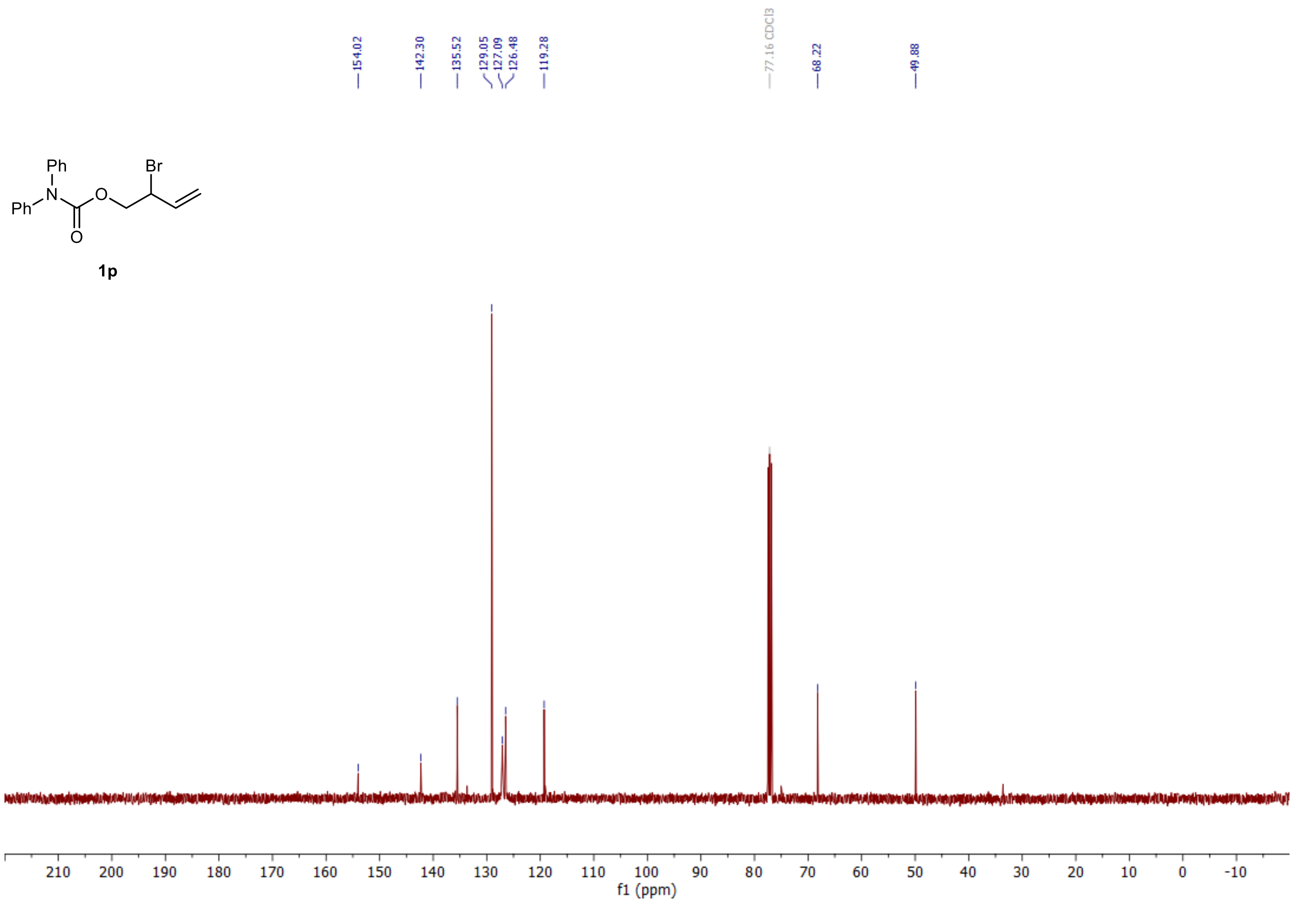

1q ^{1}H NMR (400 MHz, $CDCl_3$)

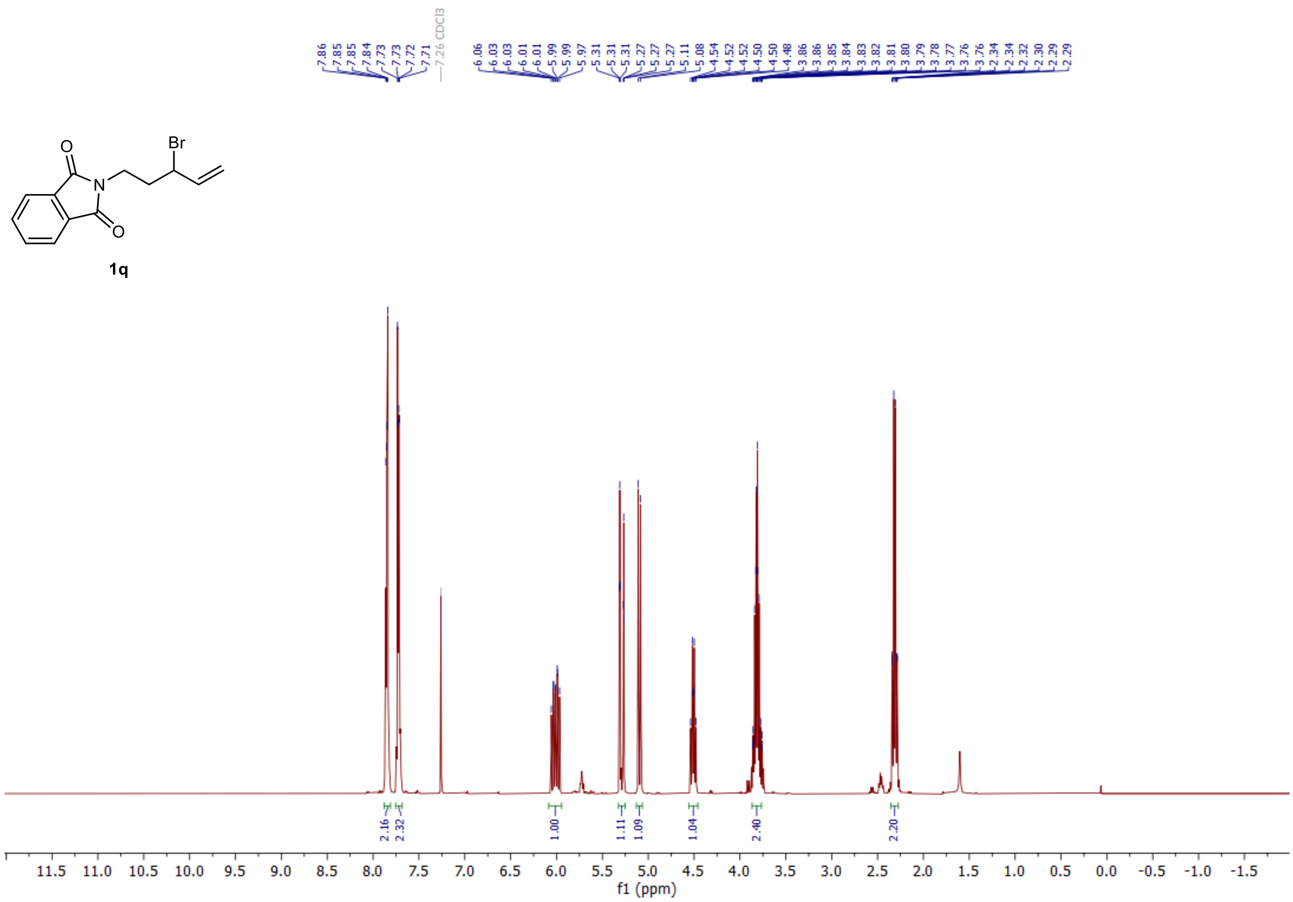

1q ^{13}C NMR (101 MHz, $CDCl_3$)

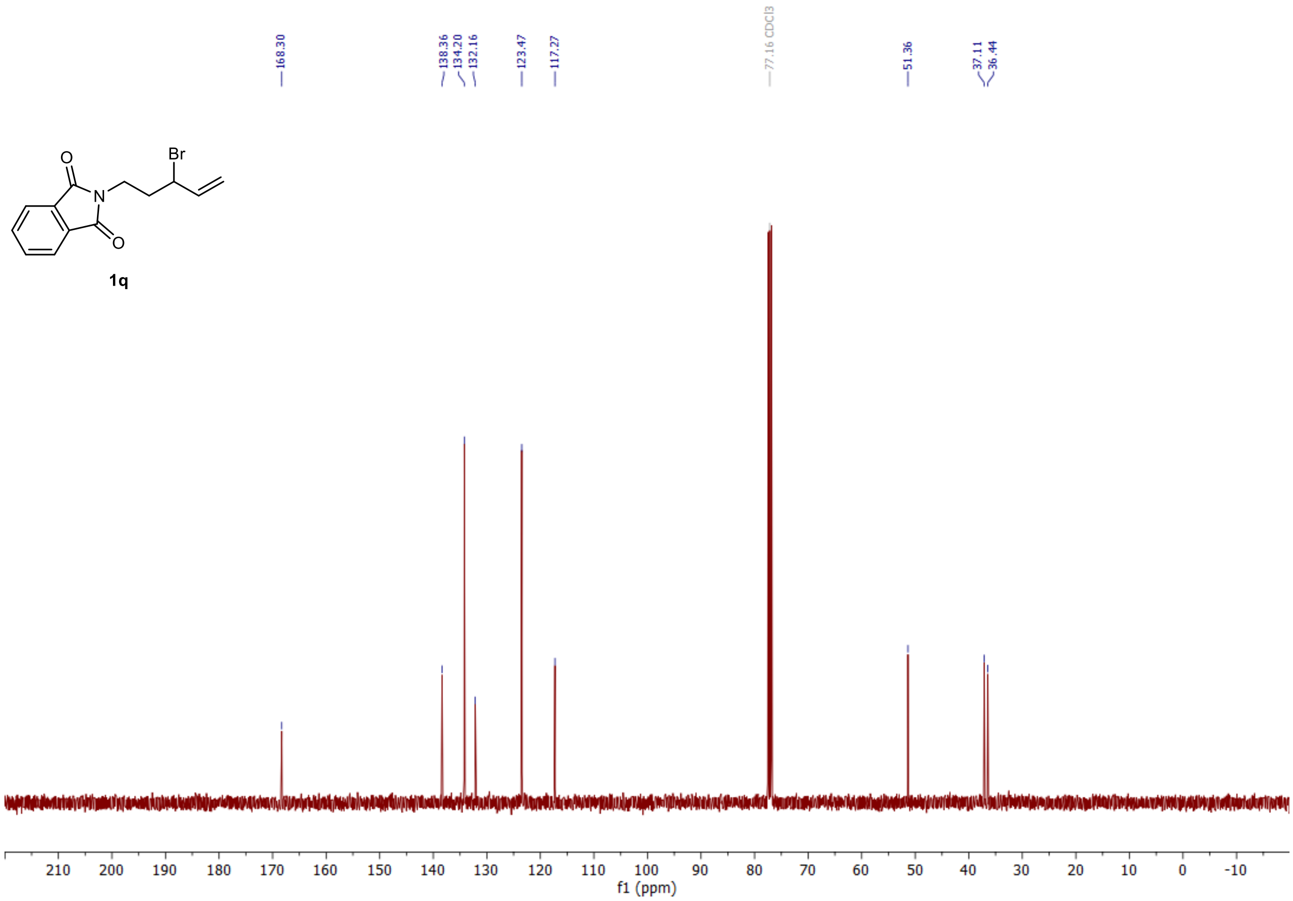

1r ^{1}H NMR (400 MHz, $CDCl_3$)

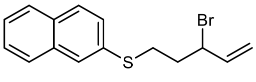

1r

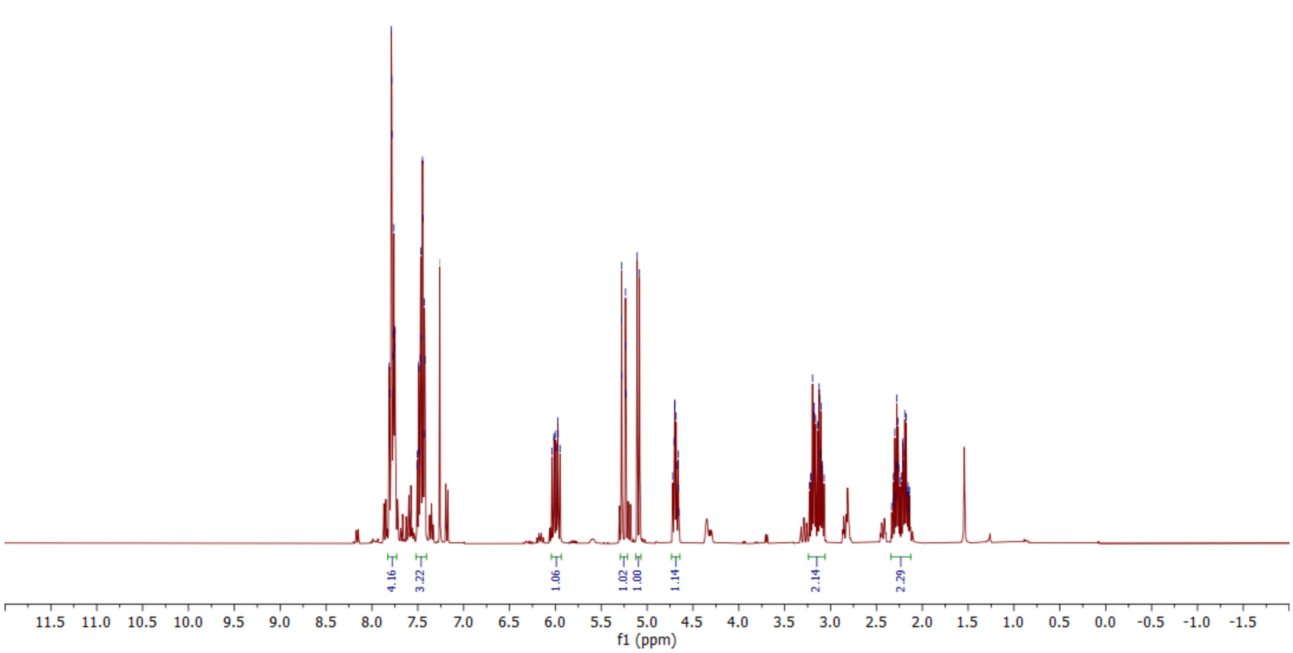

1r ^{13}C NMR (101 MHz, $CDCl_3$)

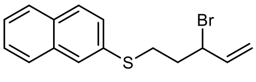

1r

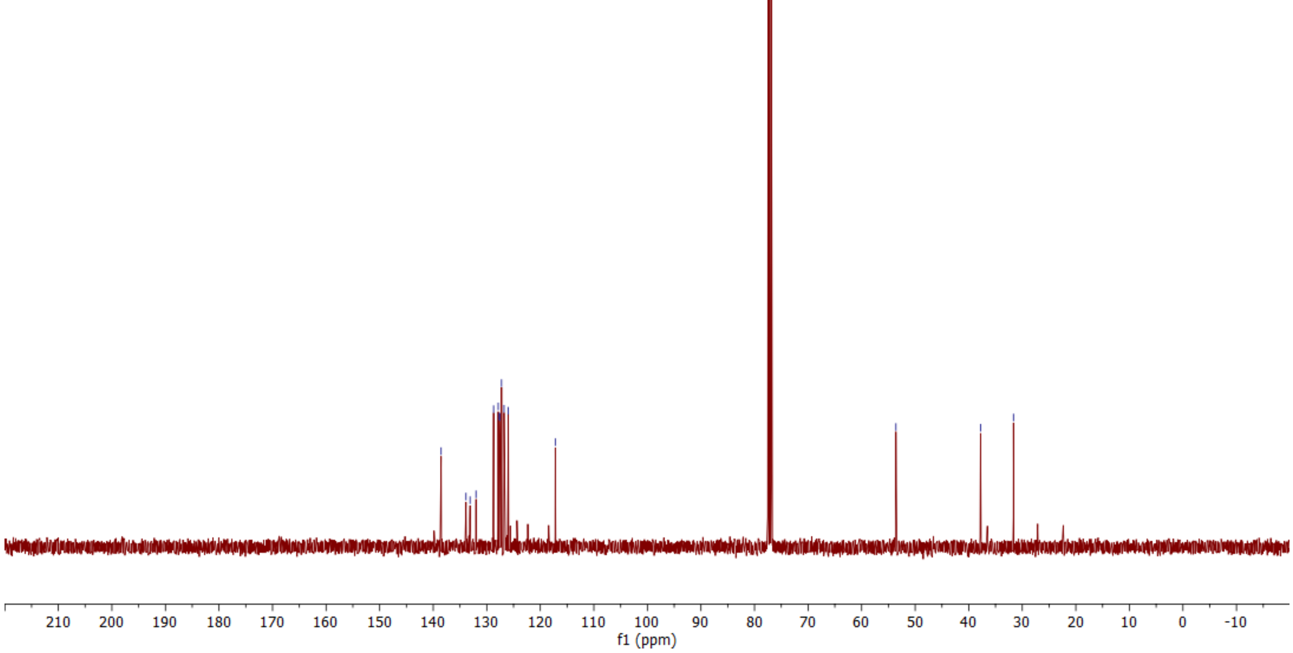

1s ^{1}H NMR (400 MHz, $CDCl_3$)

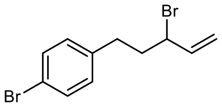

1s

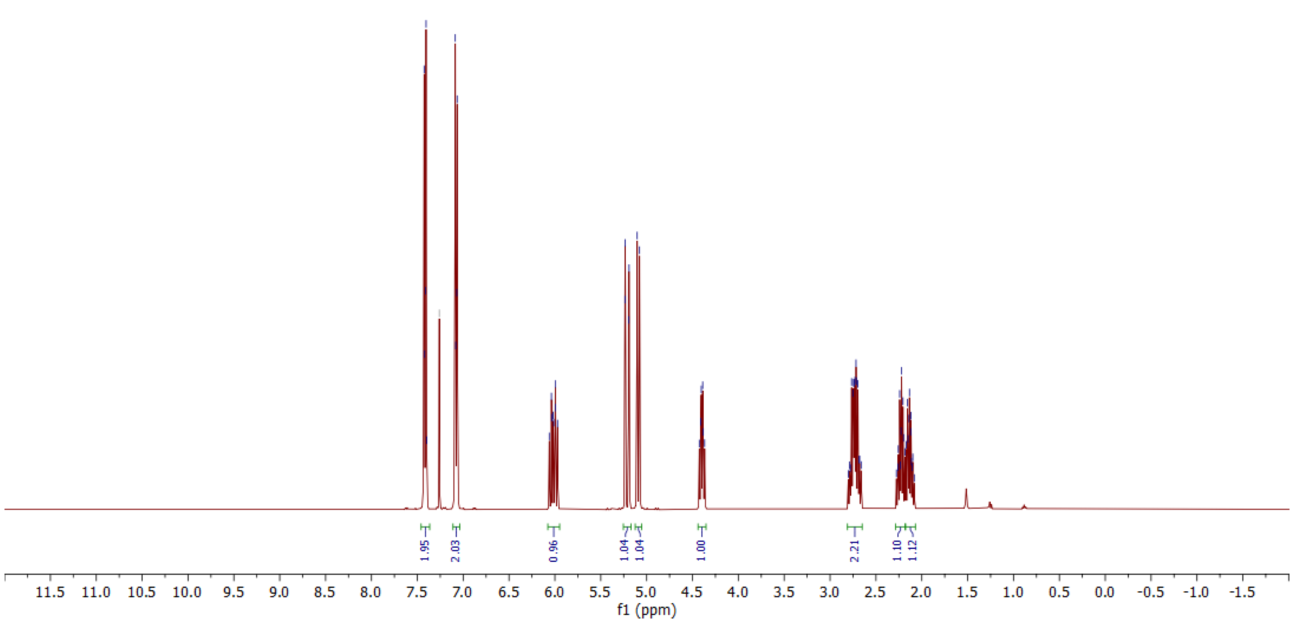

1s ^{13}C NMR (101 MHz, $CDCl_3$)

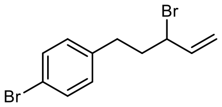

1s

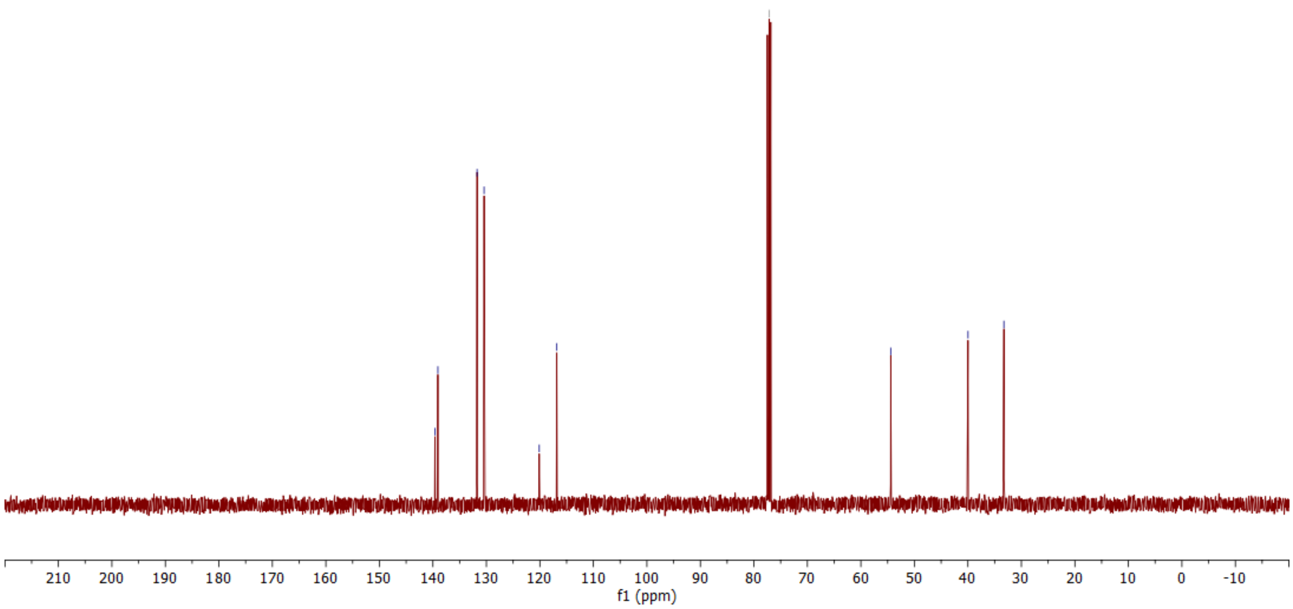

1t ^{1}H NMR (400 MHz, $CDCl_3$)

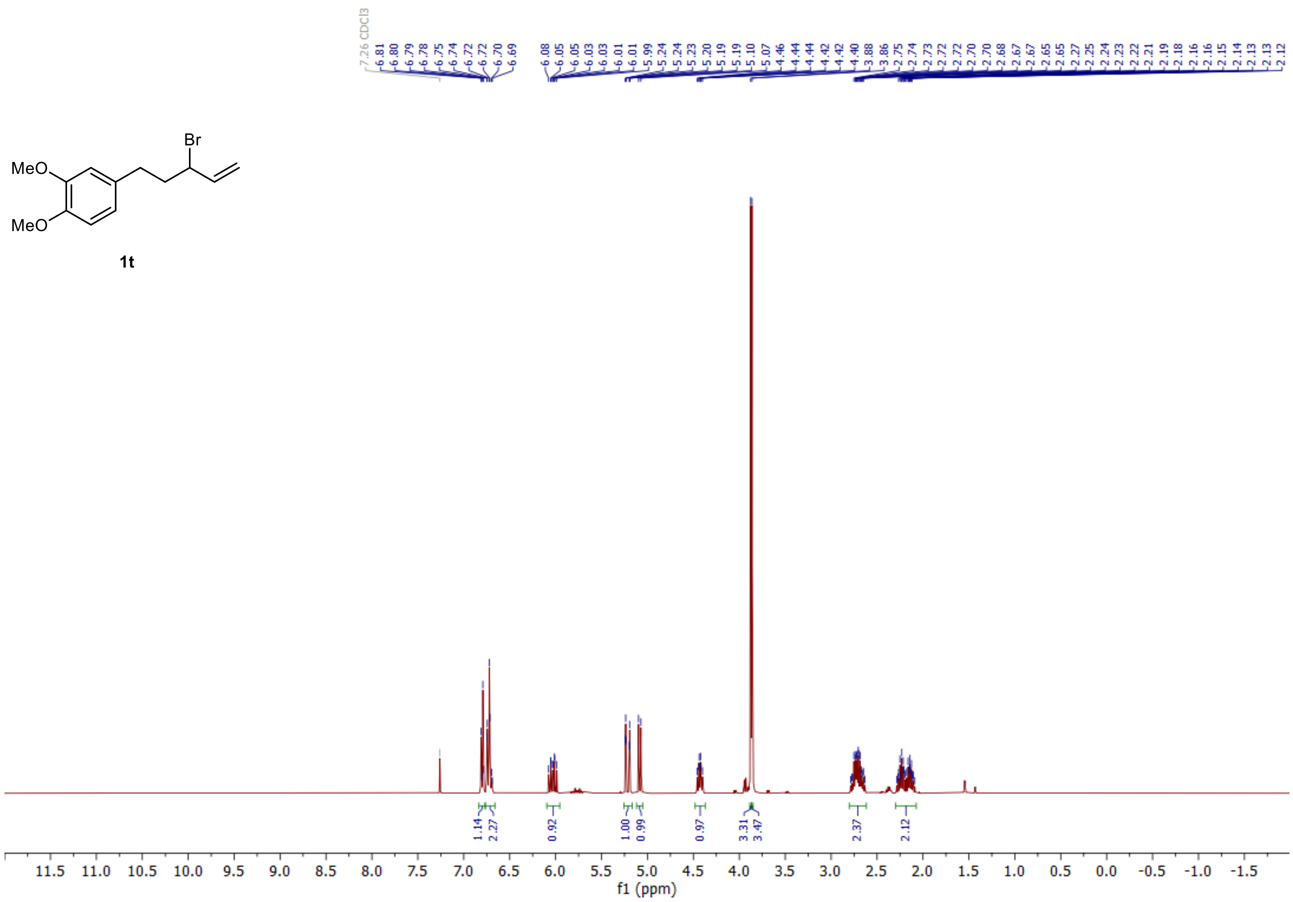

1t ^{13}C NMR (101 MHz, $CDCl_3$)

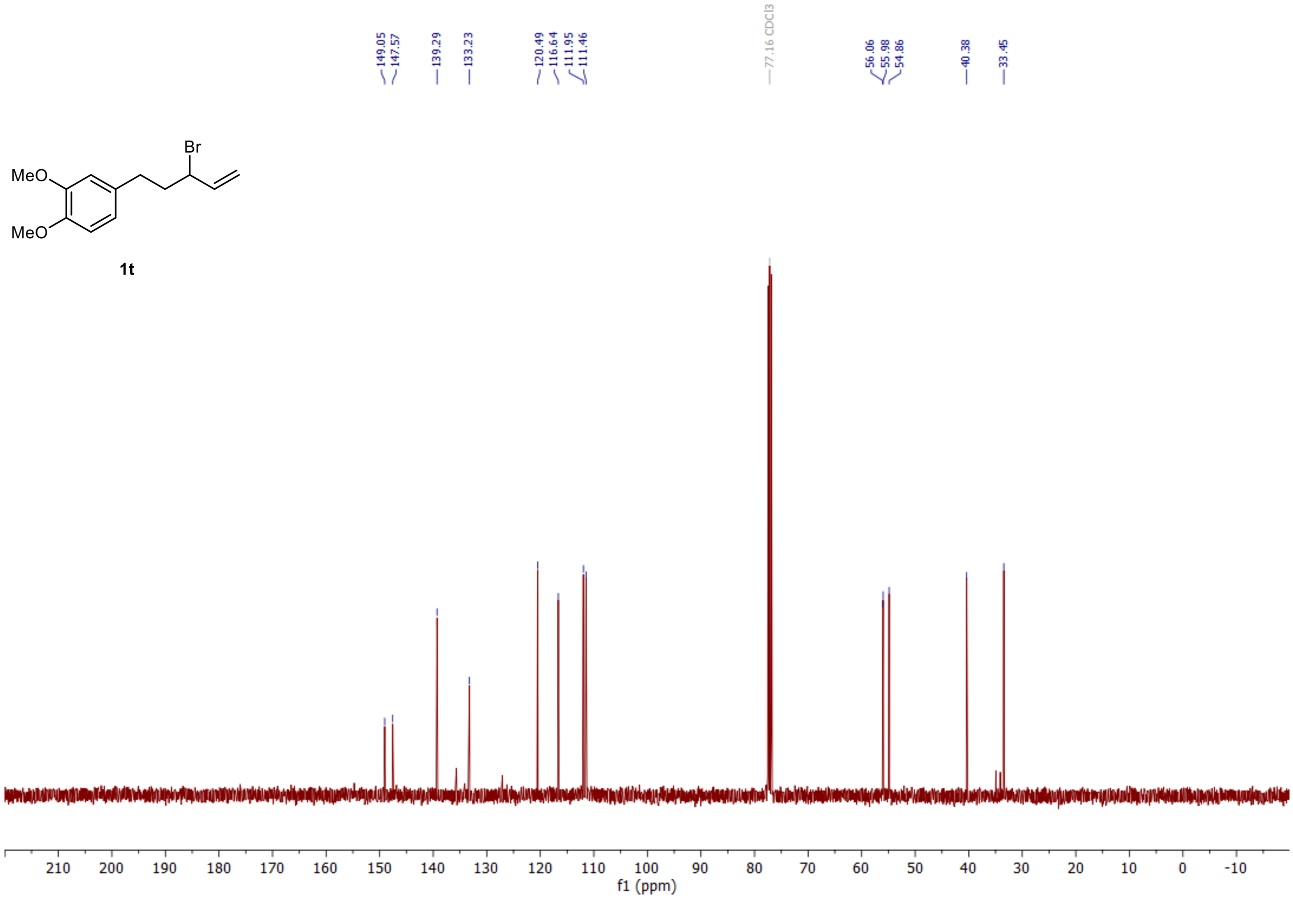

1u ^{1}H NMR (400 MHz, CD_2Cl_2)

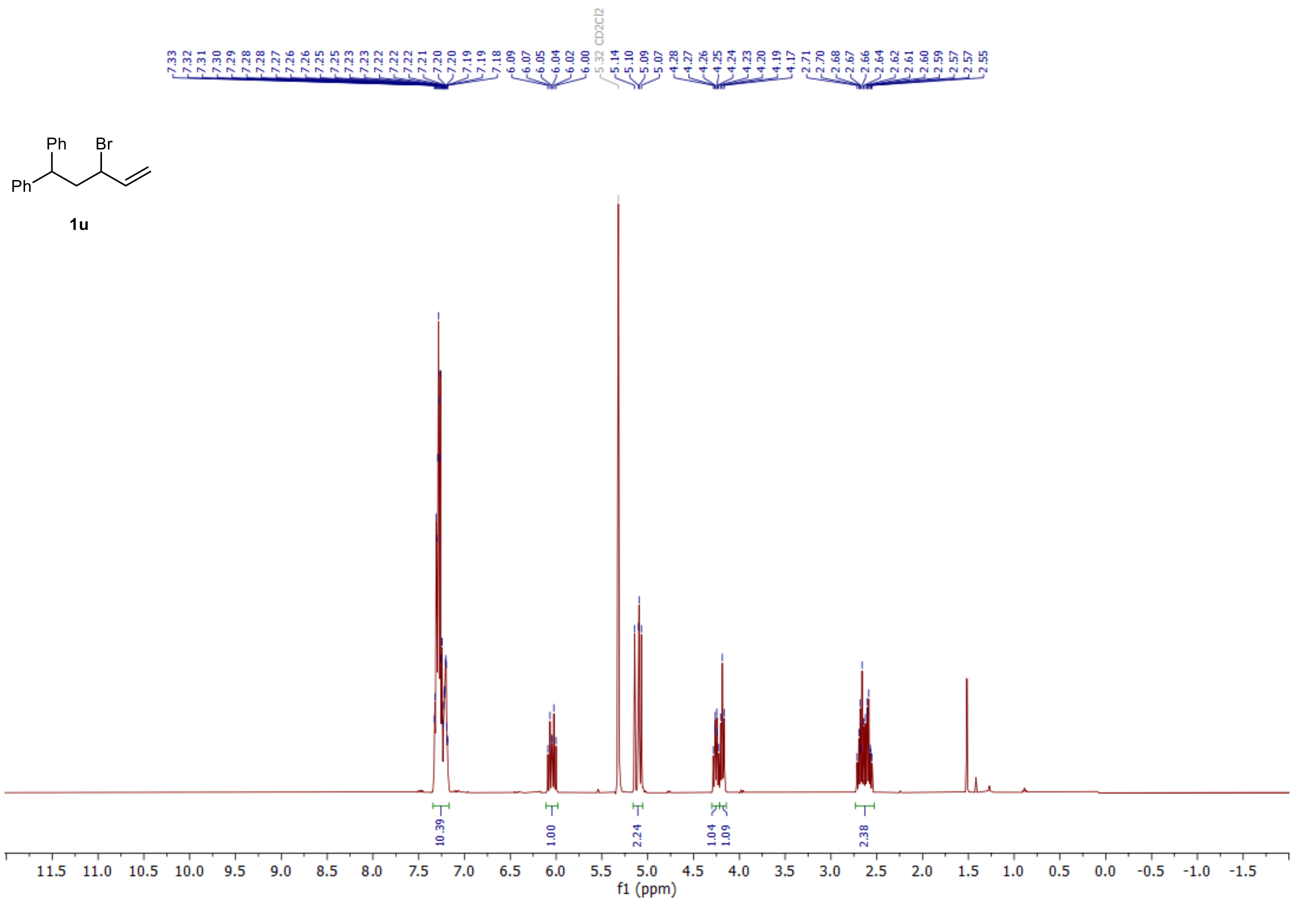

1u ^{13}C NMR (101 MHz, CD_2Cl_2)

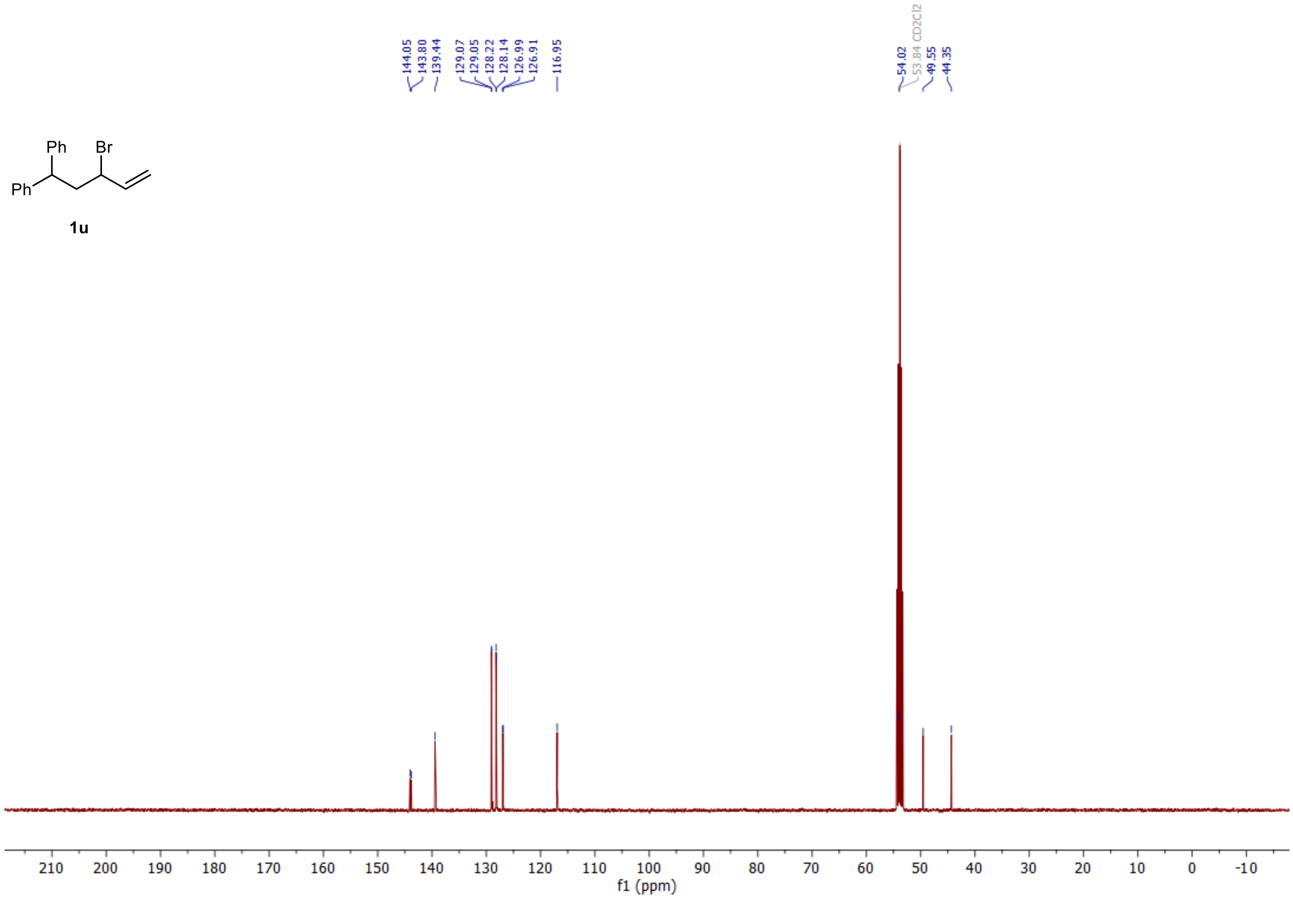

1v ^{1}H NMR (400 MHz, $CDCl_3$)

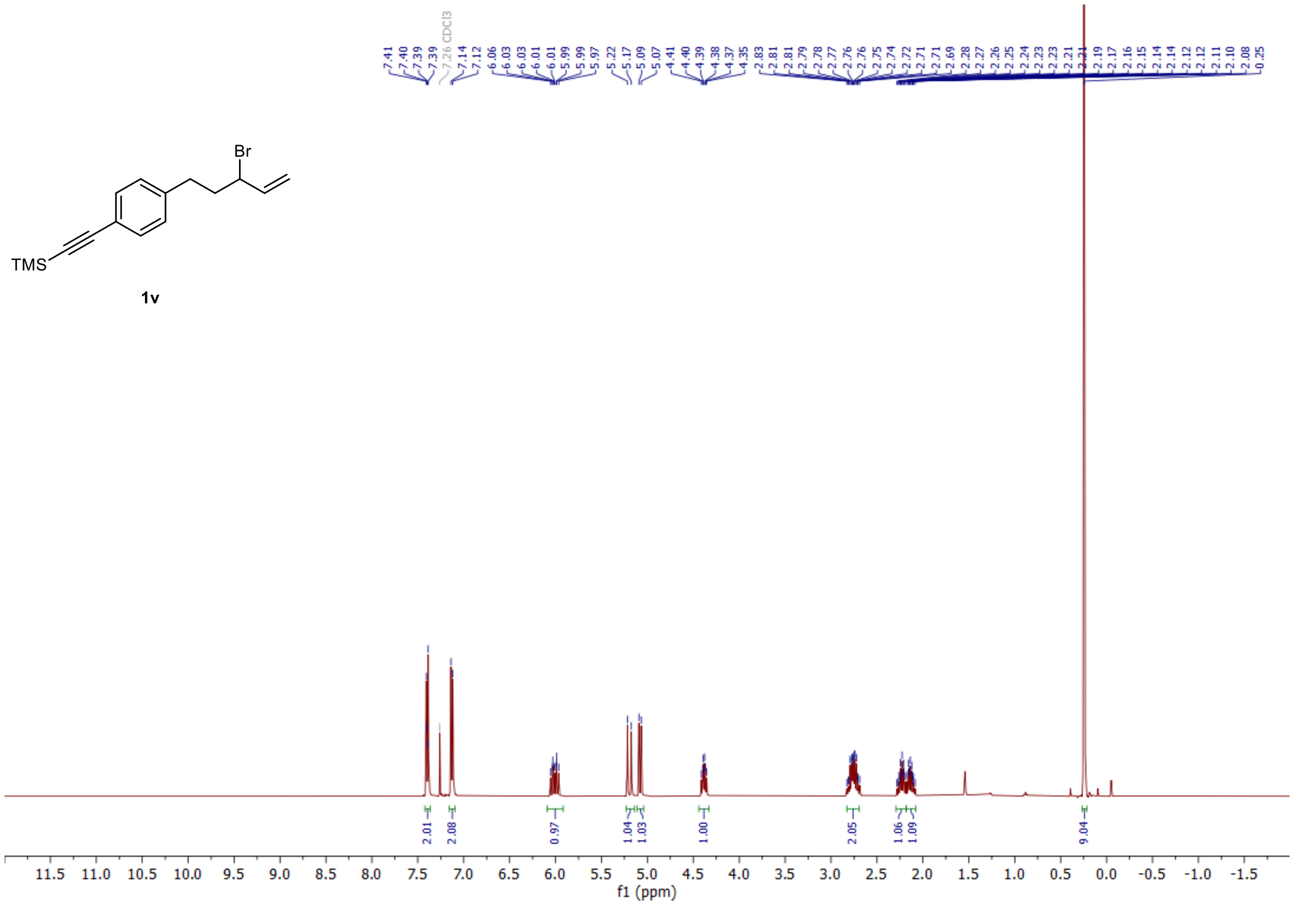

1v ^{13}C NMR (101 MHz, $CDCl_3$)

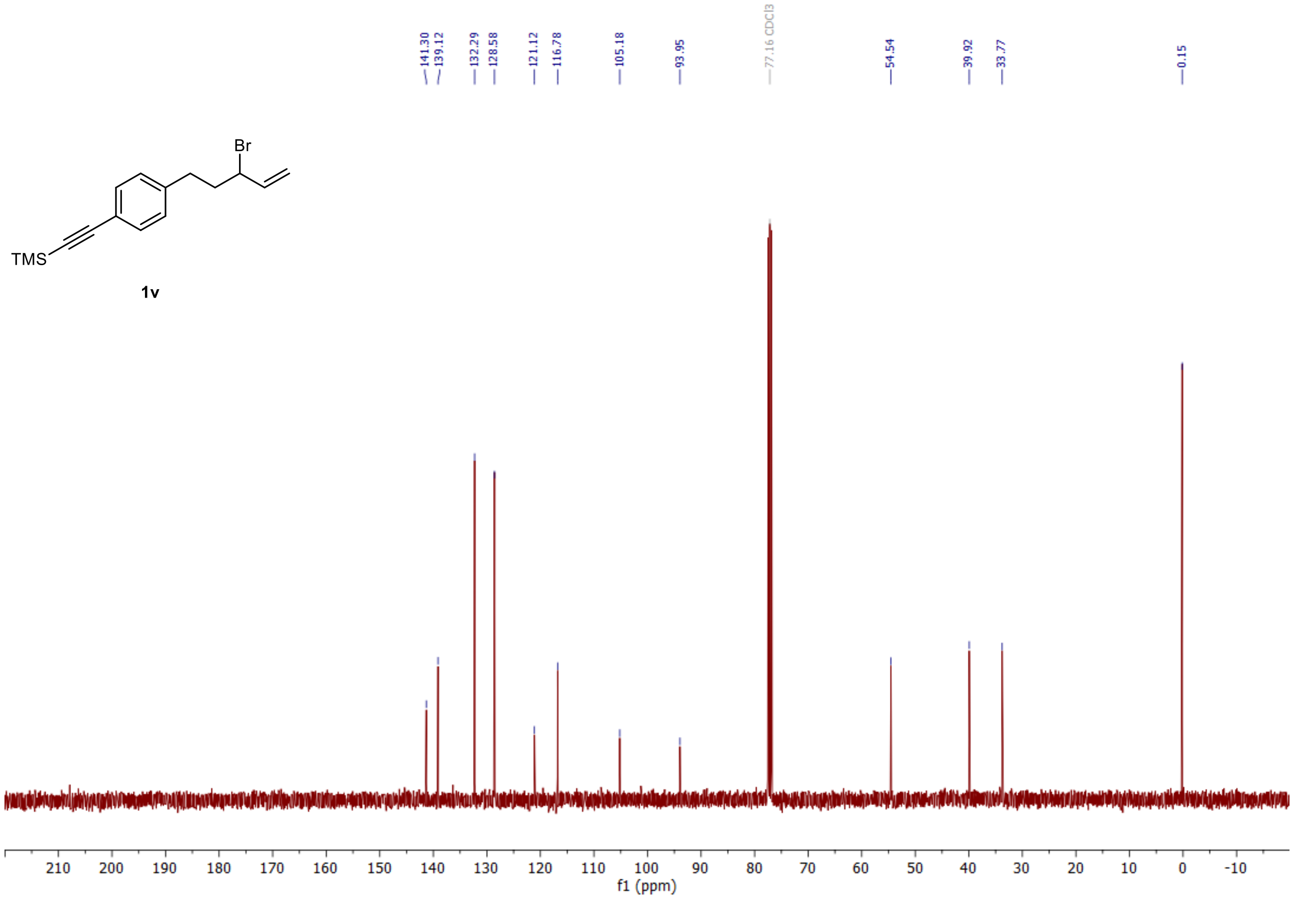

1w ^{1}H NMR (400 MHz, $CDCl_3$)

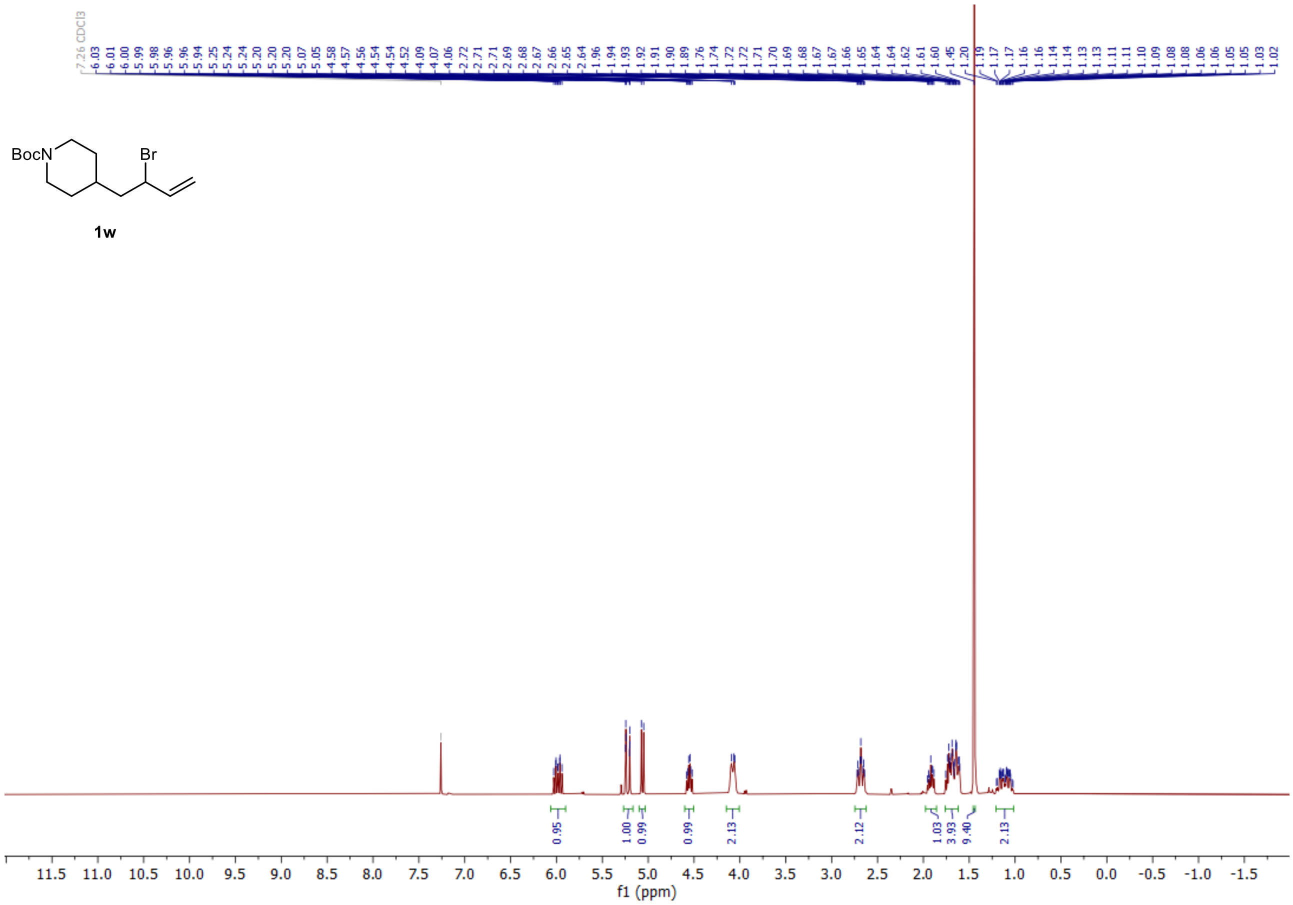

1w ^{13}C NMR (101 MHz, $CDCl_3$)

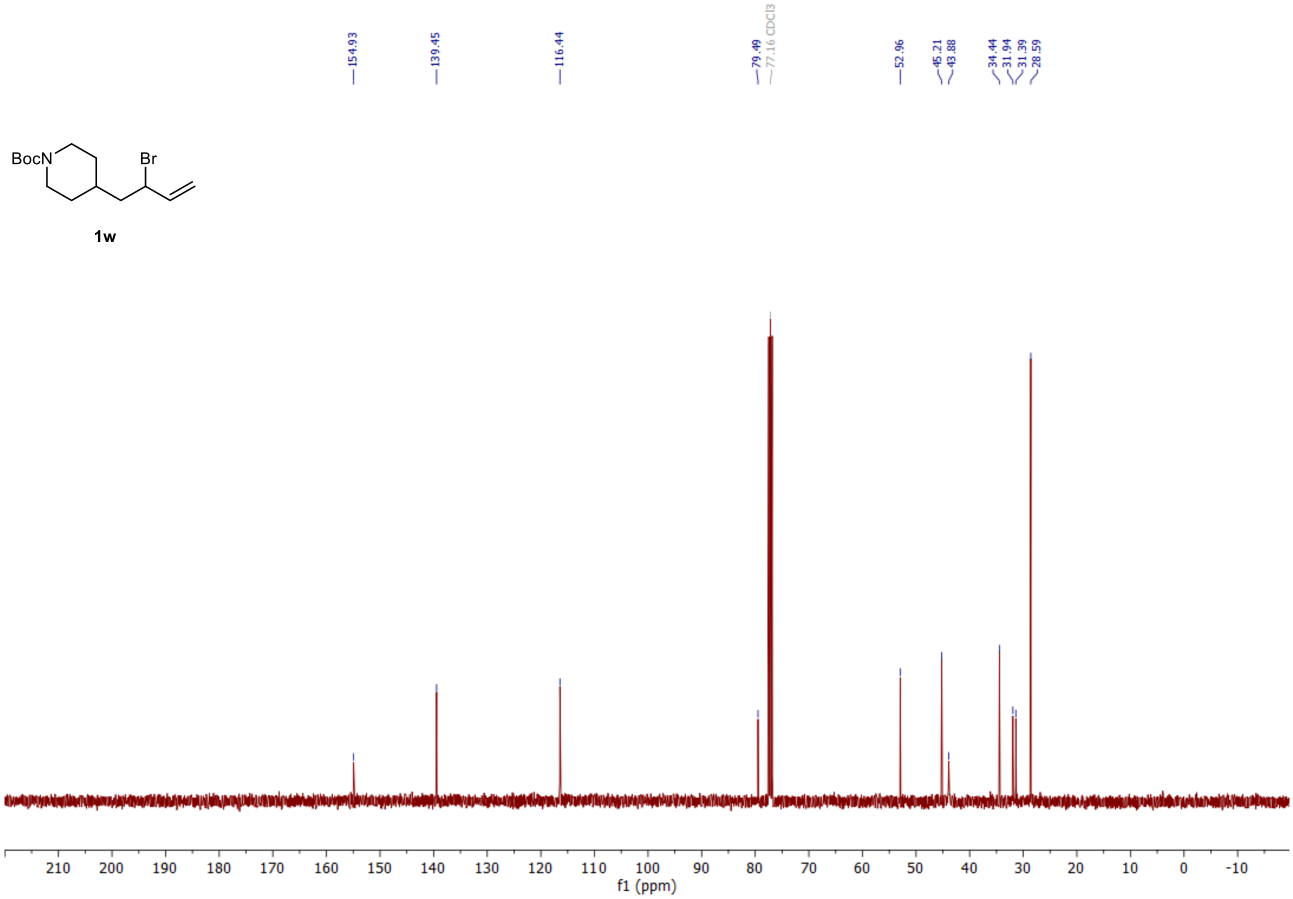

1x ^{1}H NMR (400 MHz, $CDCl_3$)

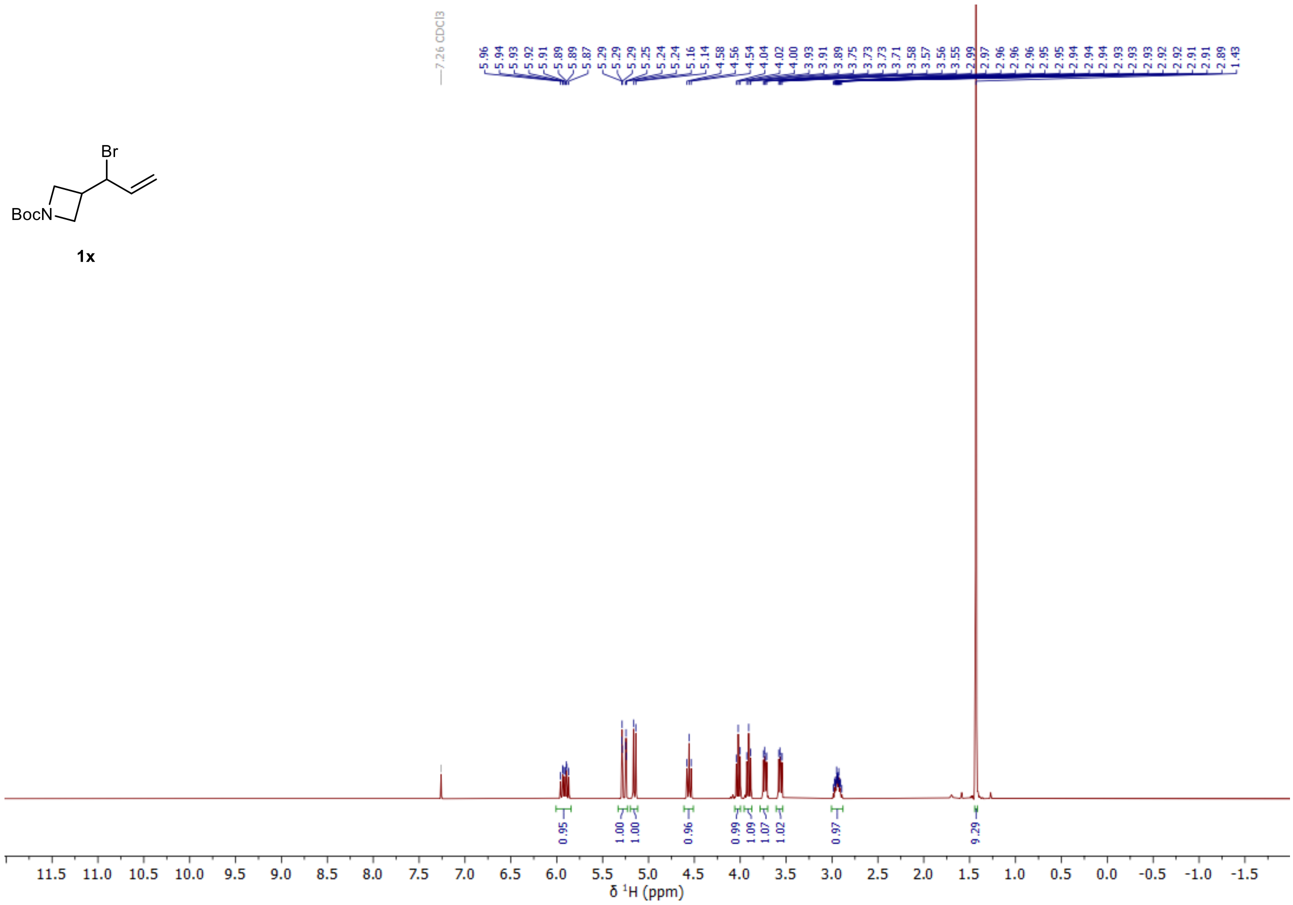

1x ^{13}C NMR (101 MHz, $CDCl_3$)

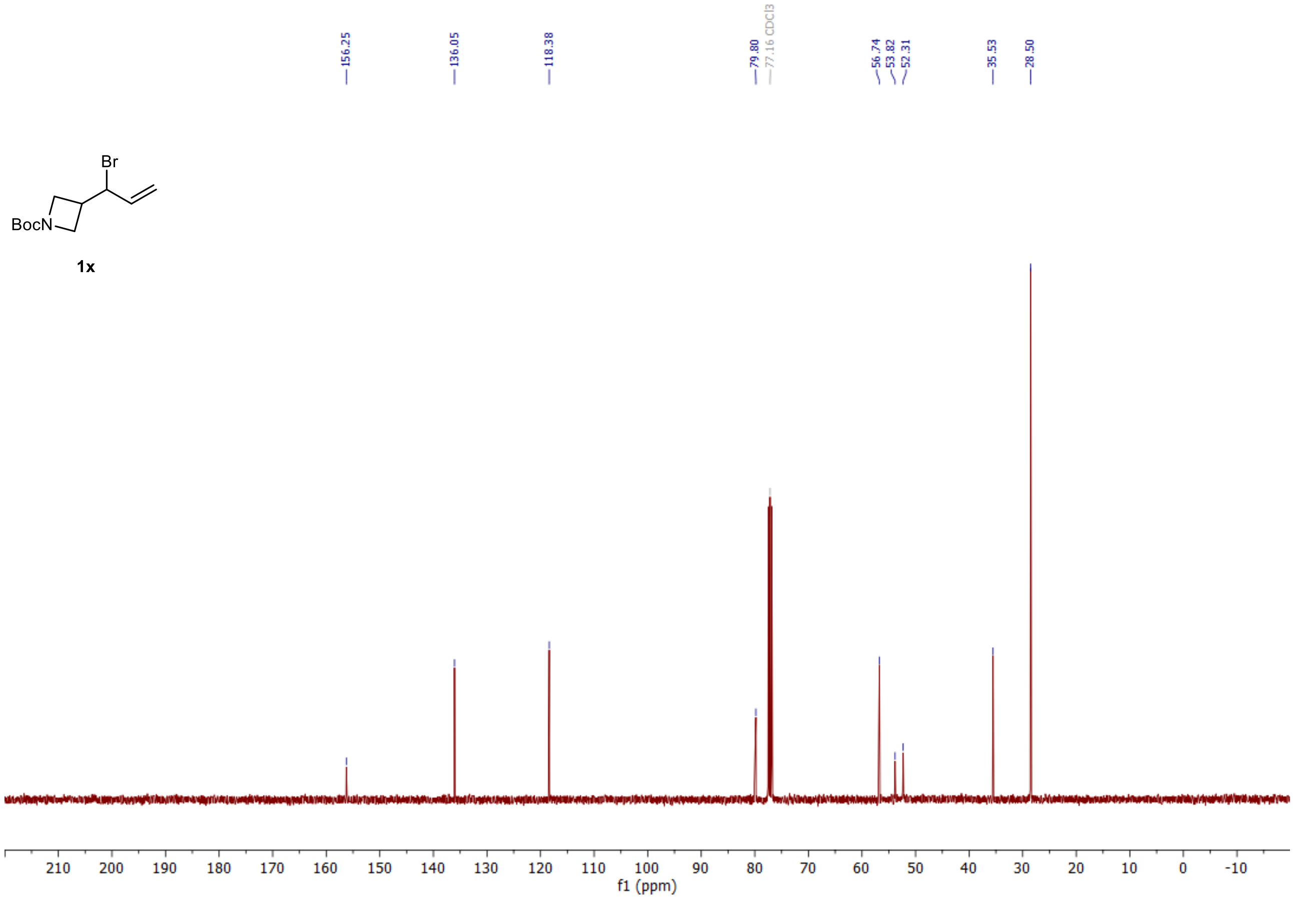

1y ^{1}H NMR (400 MHz, $CDCl_3$)

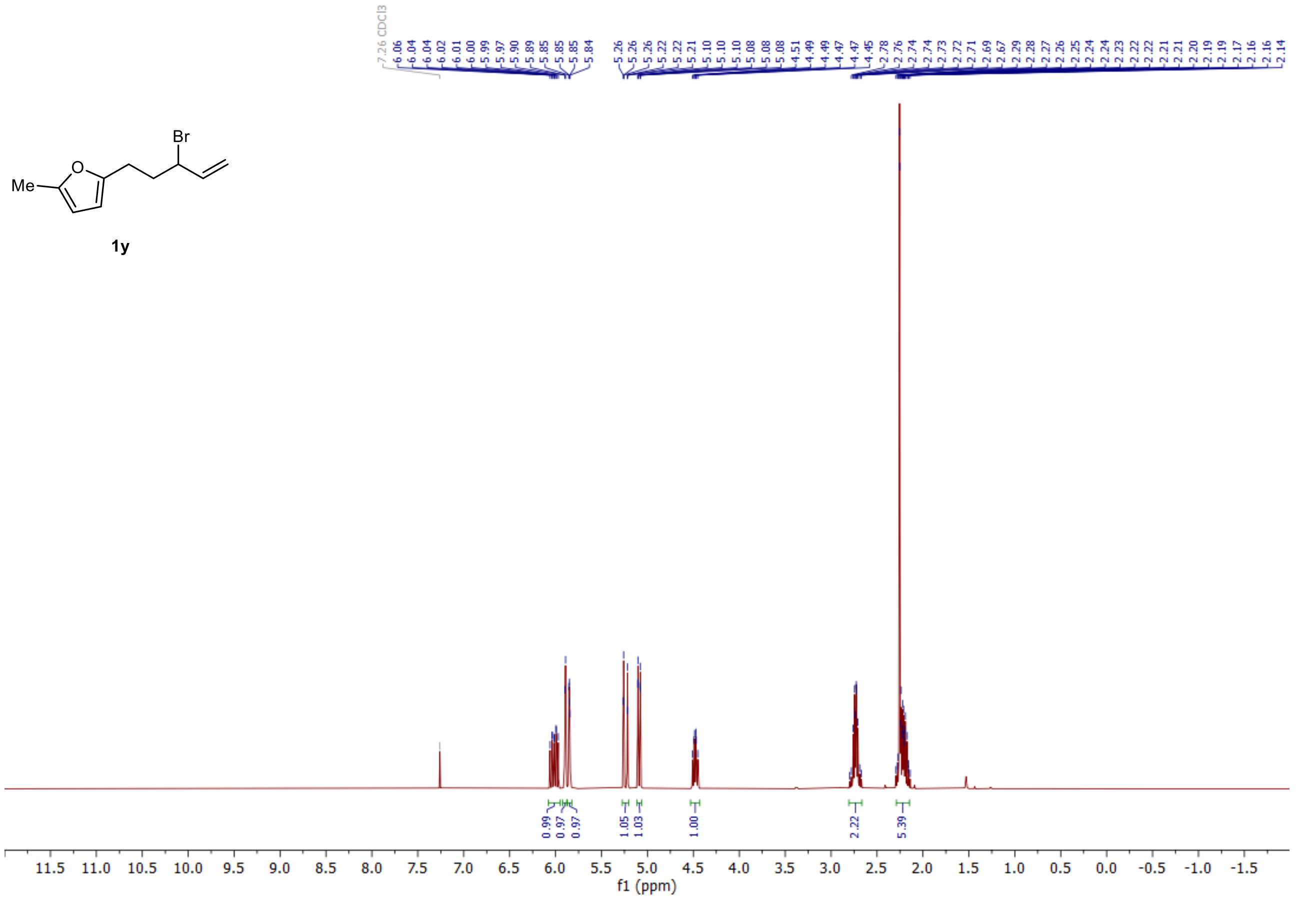

1y ^{13}C NMR (101 MHz, $CDCl_3$)

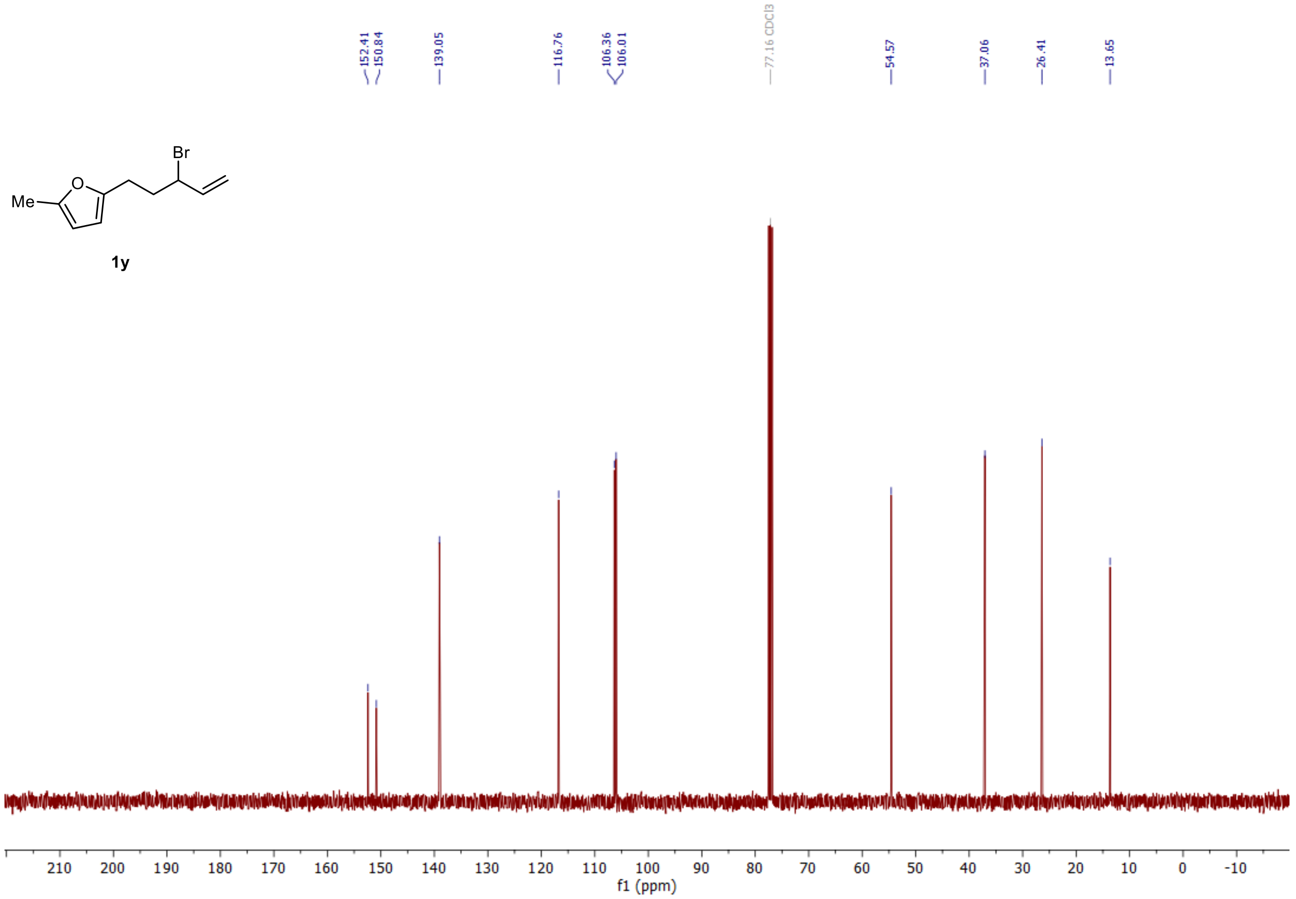

1z ^{1}H NMR (400 MHz, $CDCl_3$)

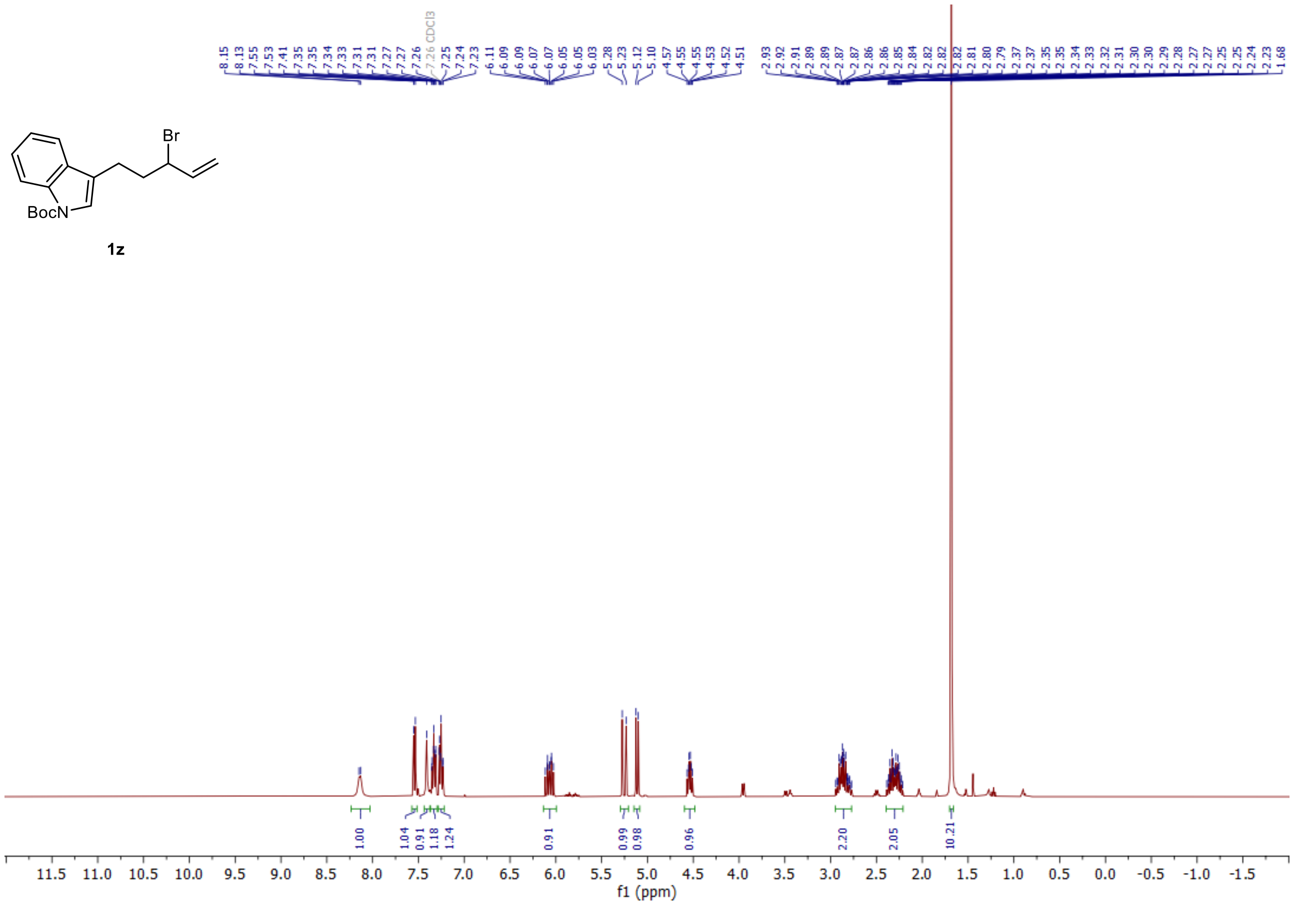

1z ^{13}C NMR (101 MHz, $CDCl_3$)

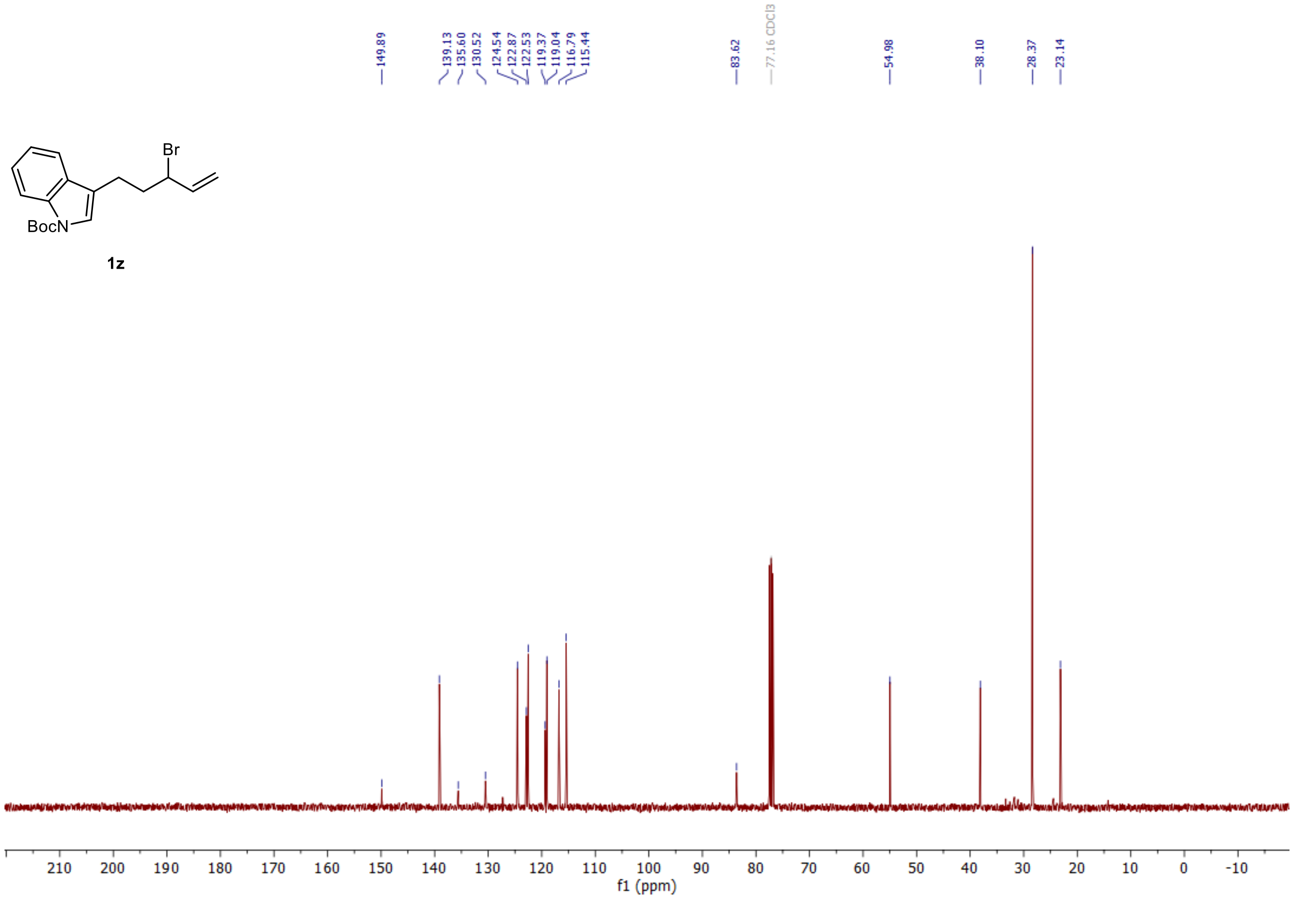

1aa ^{1}H NMR (400 MHz, $CDCl_3$)

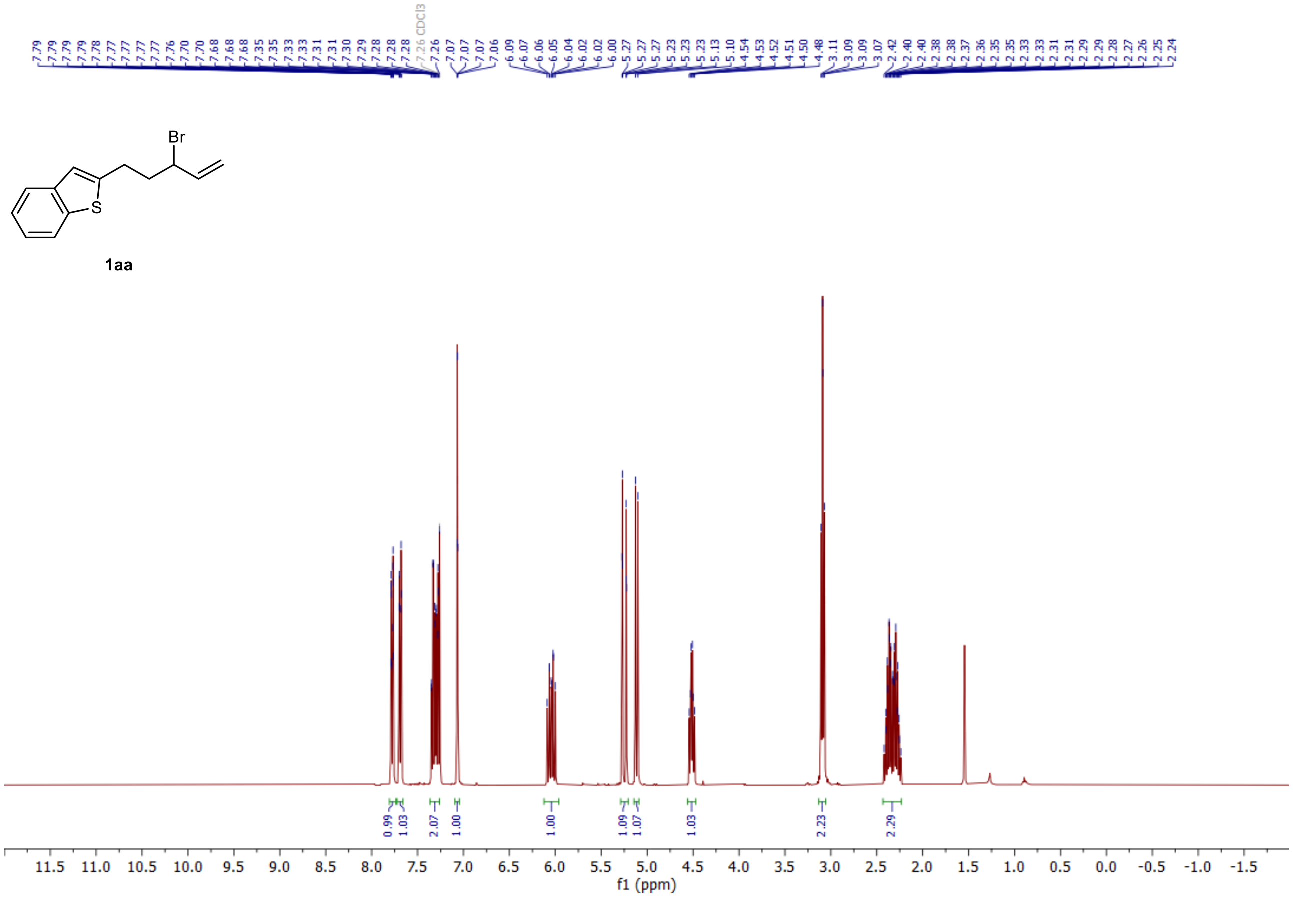

1aa ^{13}C NMR (101 MHz, $CDCl_3$)

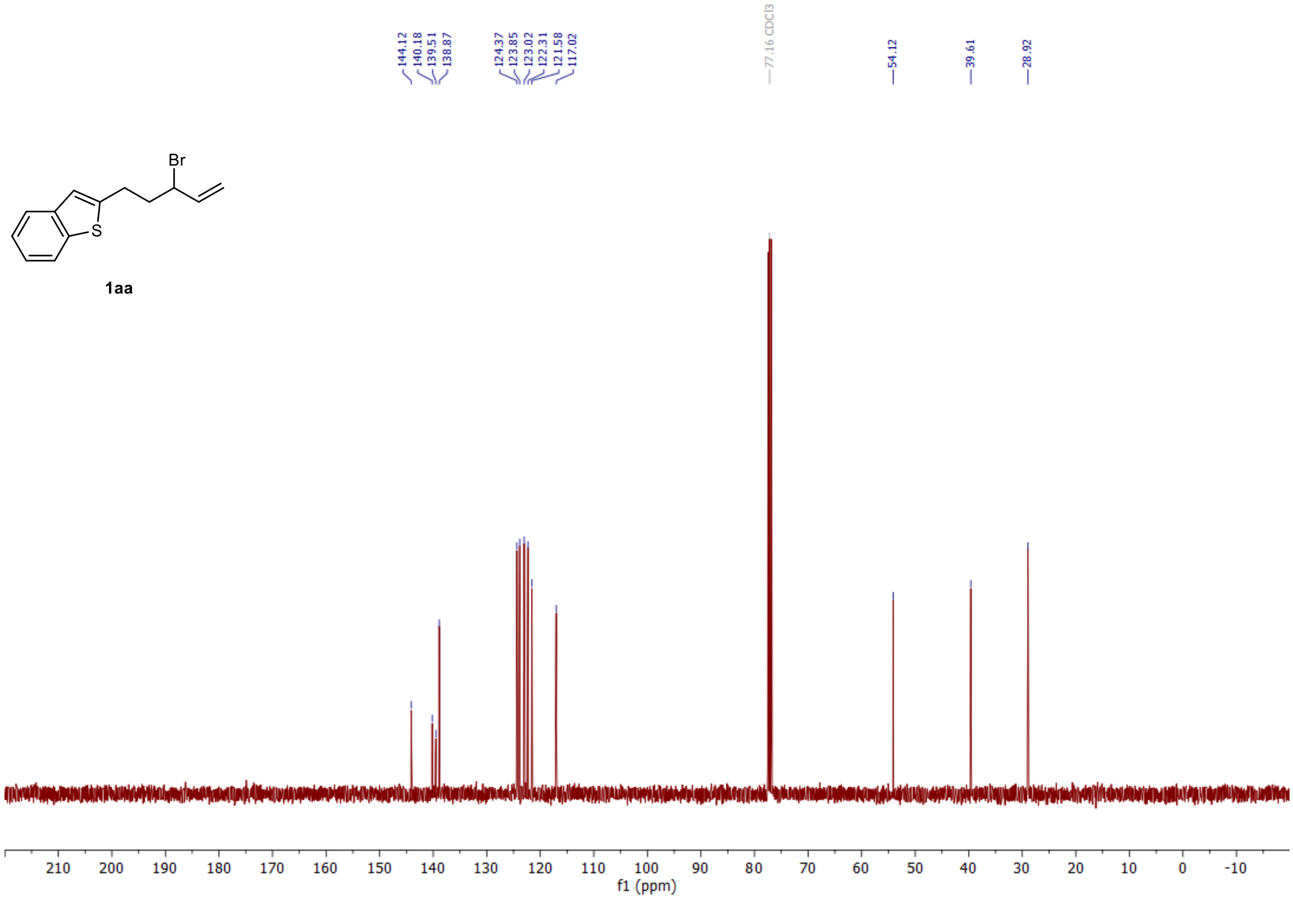

1ab ^{1}H NMR (400 MHz, $CDCl_3$)

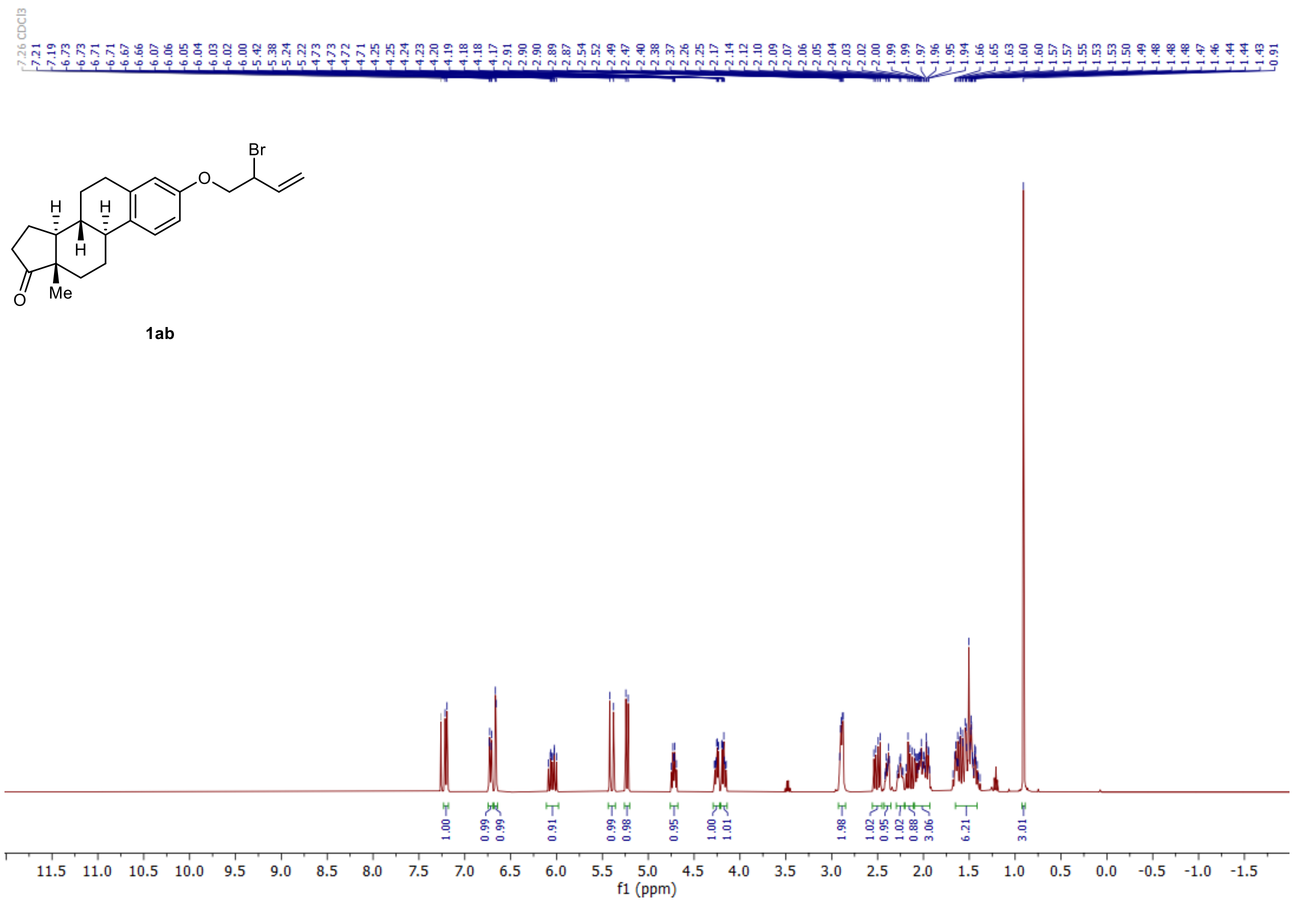

1ab ^{13}C NMR (101 MHz, $CDCl_3$)

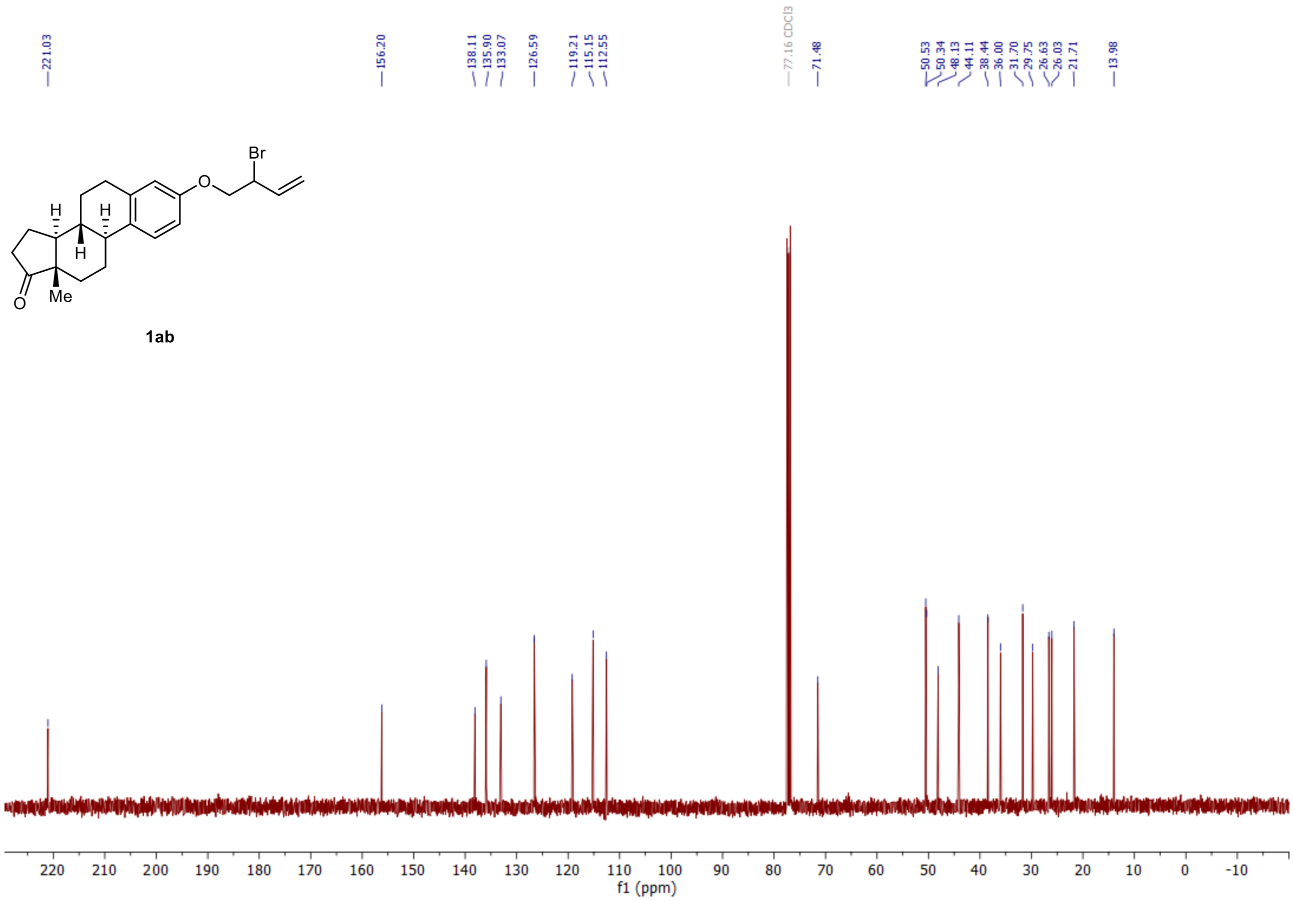

1ac ^{1}H NMR (400 MHz, $CDCl_3$)

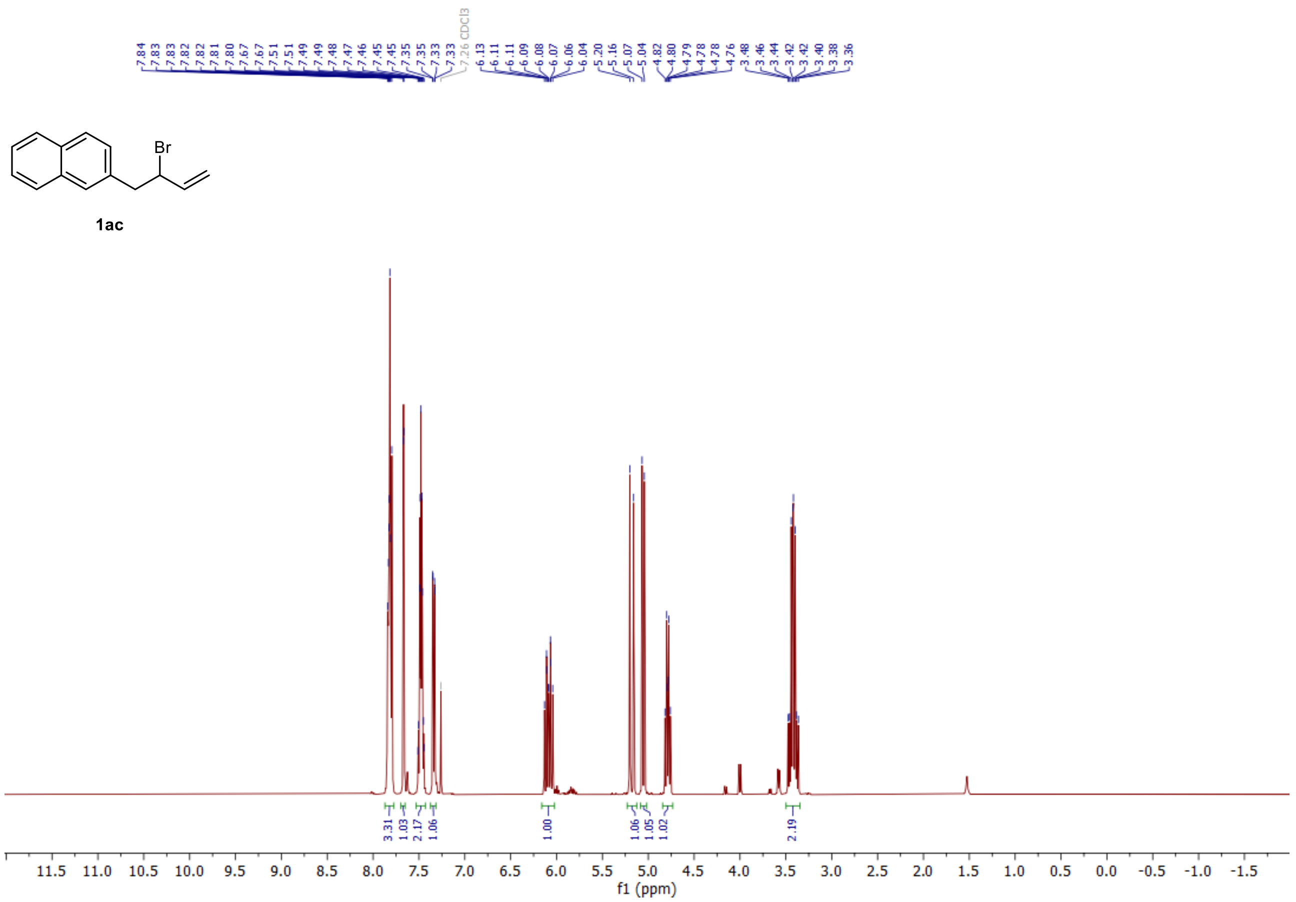

1ac ^{13}C NMR (101 MHz, $CDCl_3$)

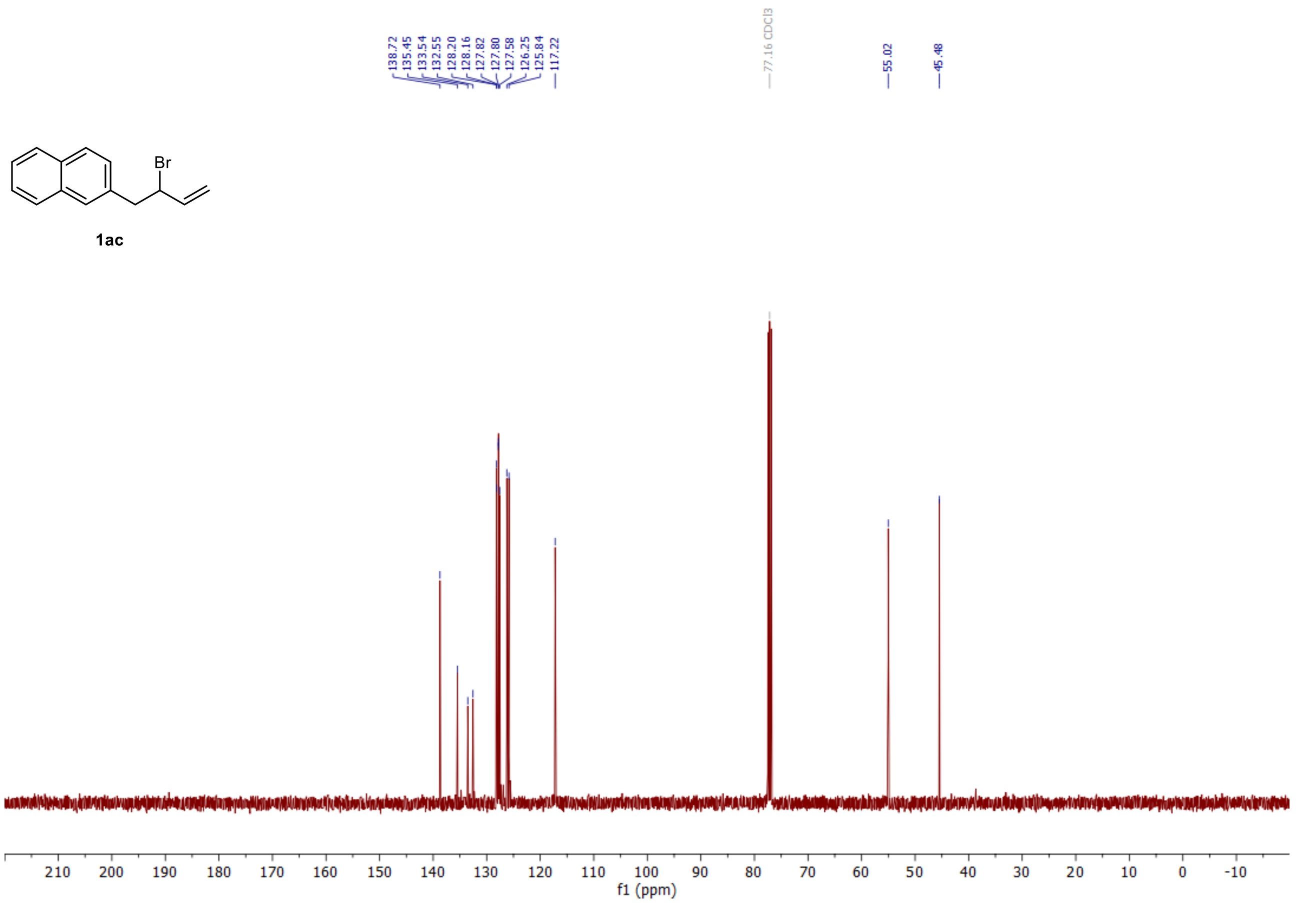

1ad ^{1}H NMR (400 MHz, $CDCl_3$)

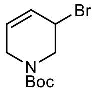

1ad

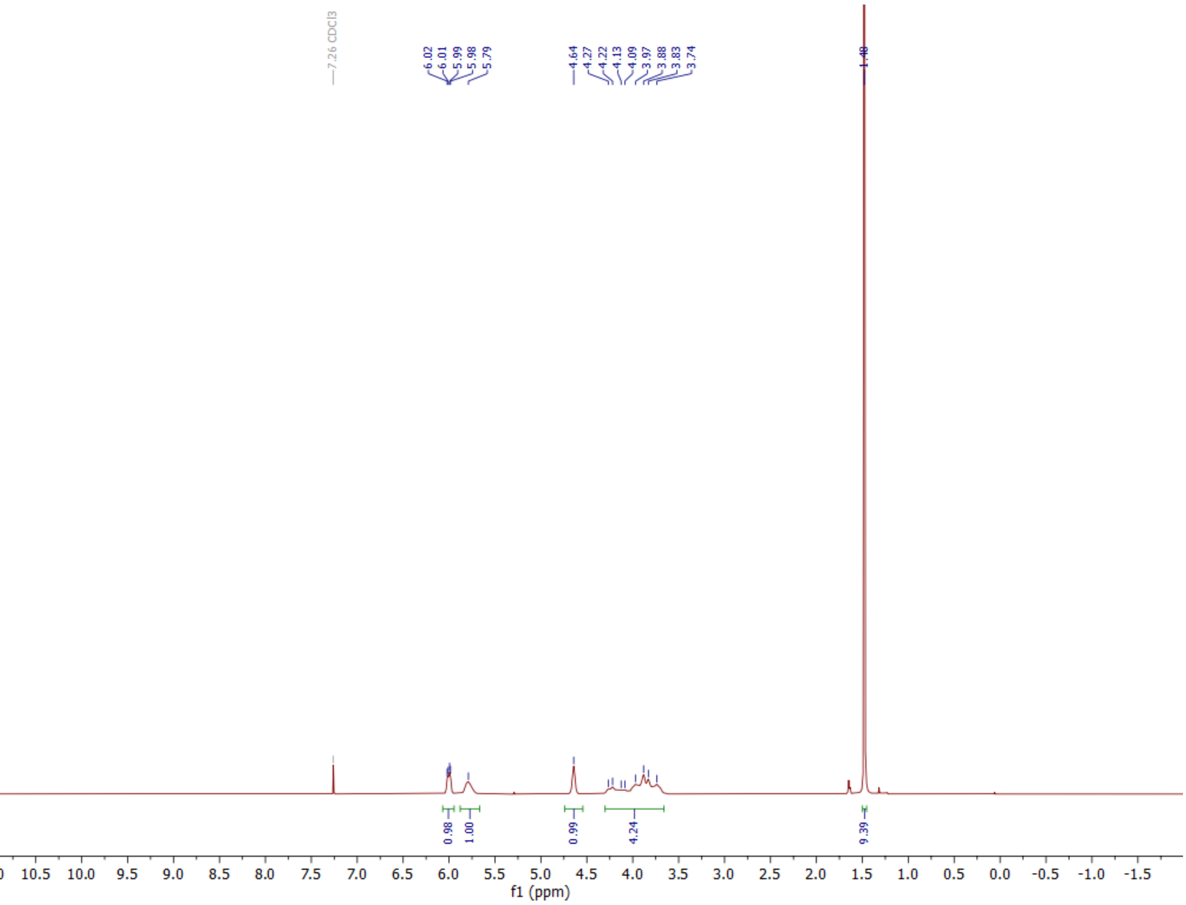

1ad ^{13}C NMR (101 MHz, $CDCl_3$)

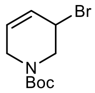

1ad

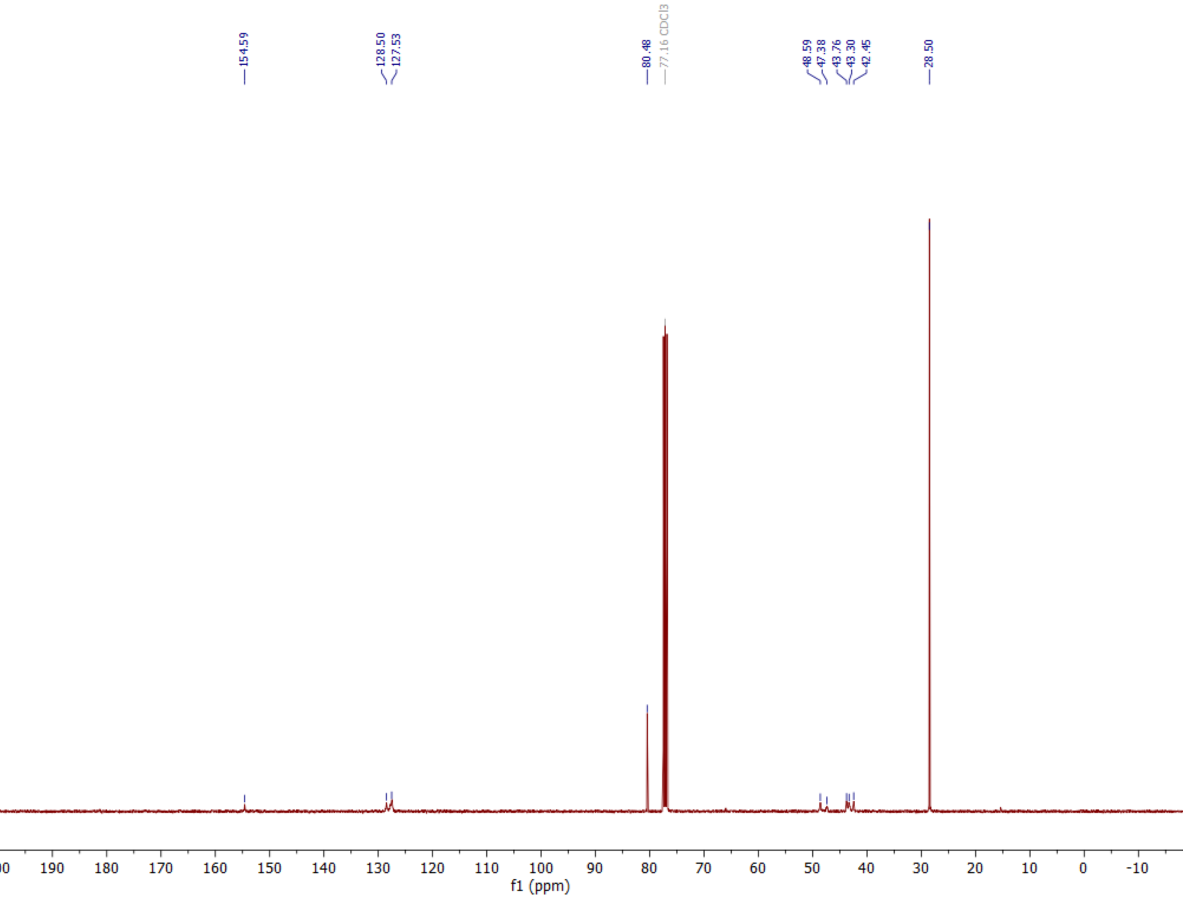

S1 ^{1}H NMR (400 MHz, $CDCl_3$)

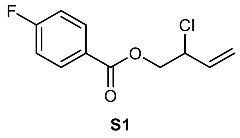

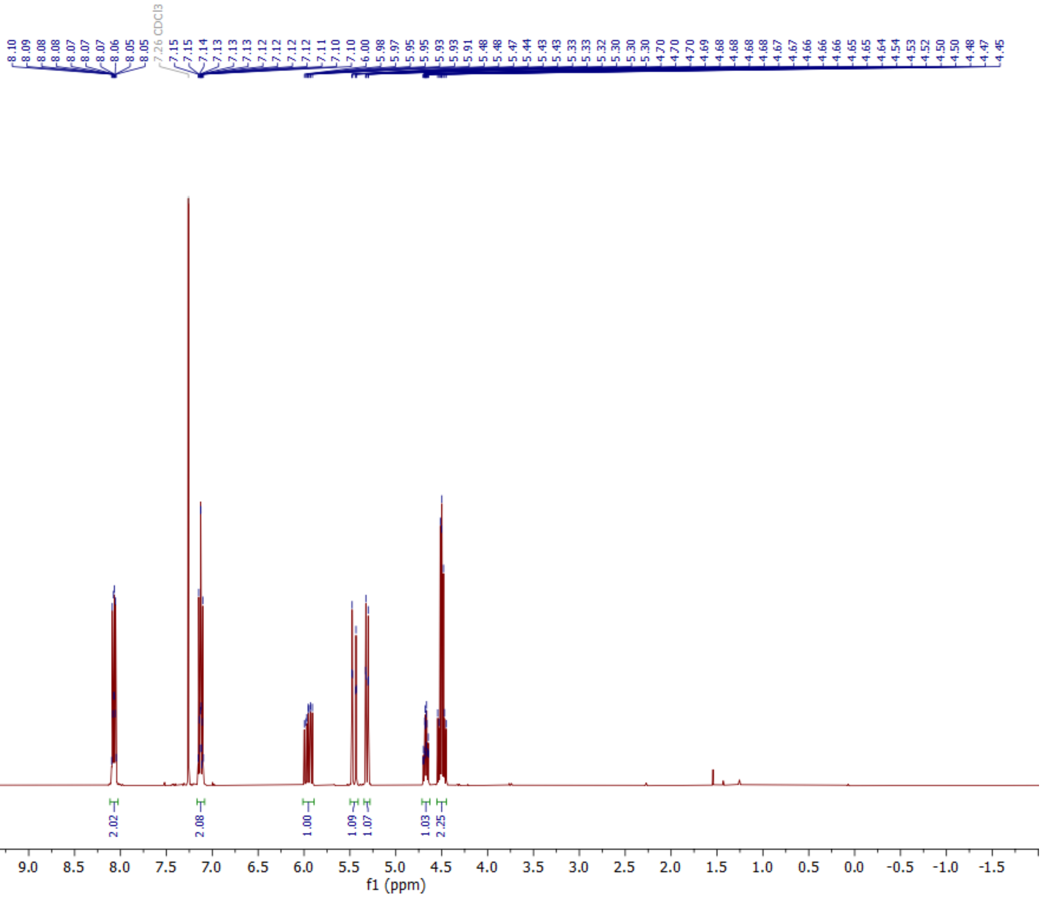

S1 ^{13}C NMR (101 MHz, $CDCl_3$)

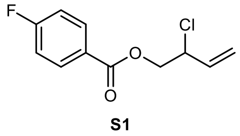

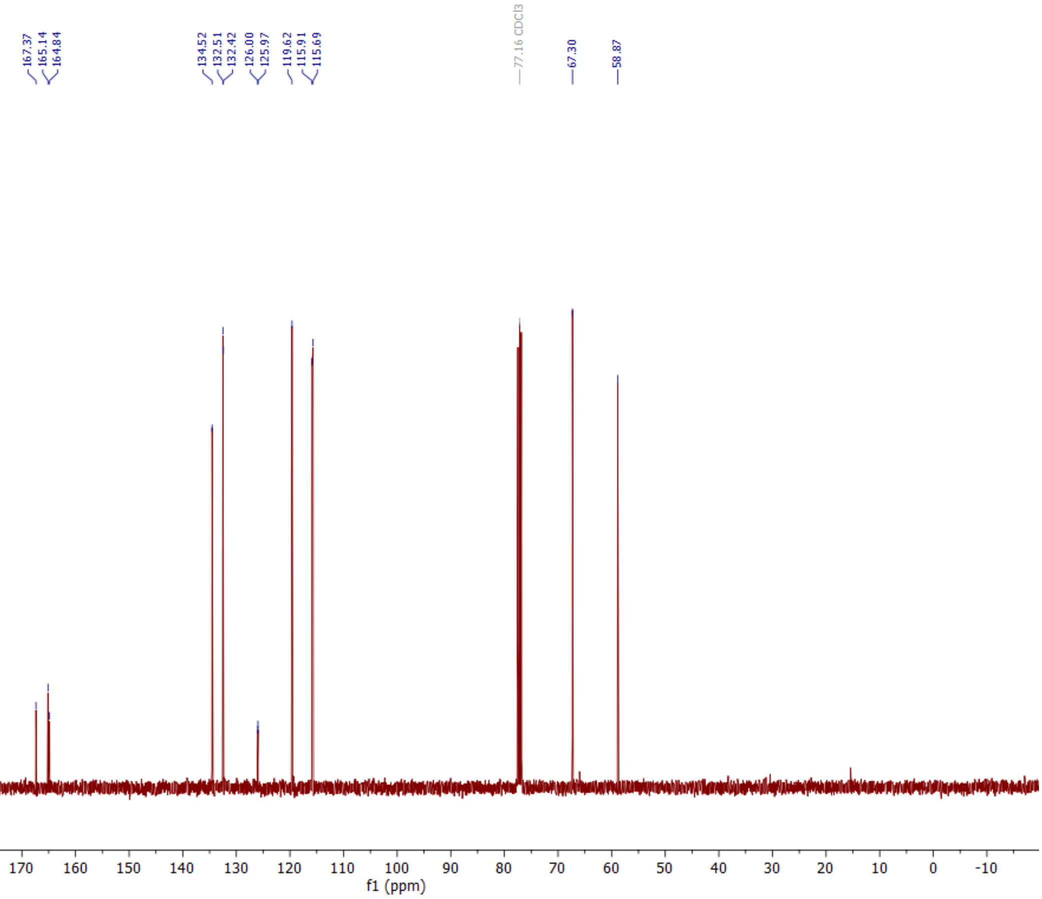

S1 ^{19}F NMR (377 MHz, CDCl$_3$)

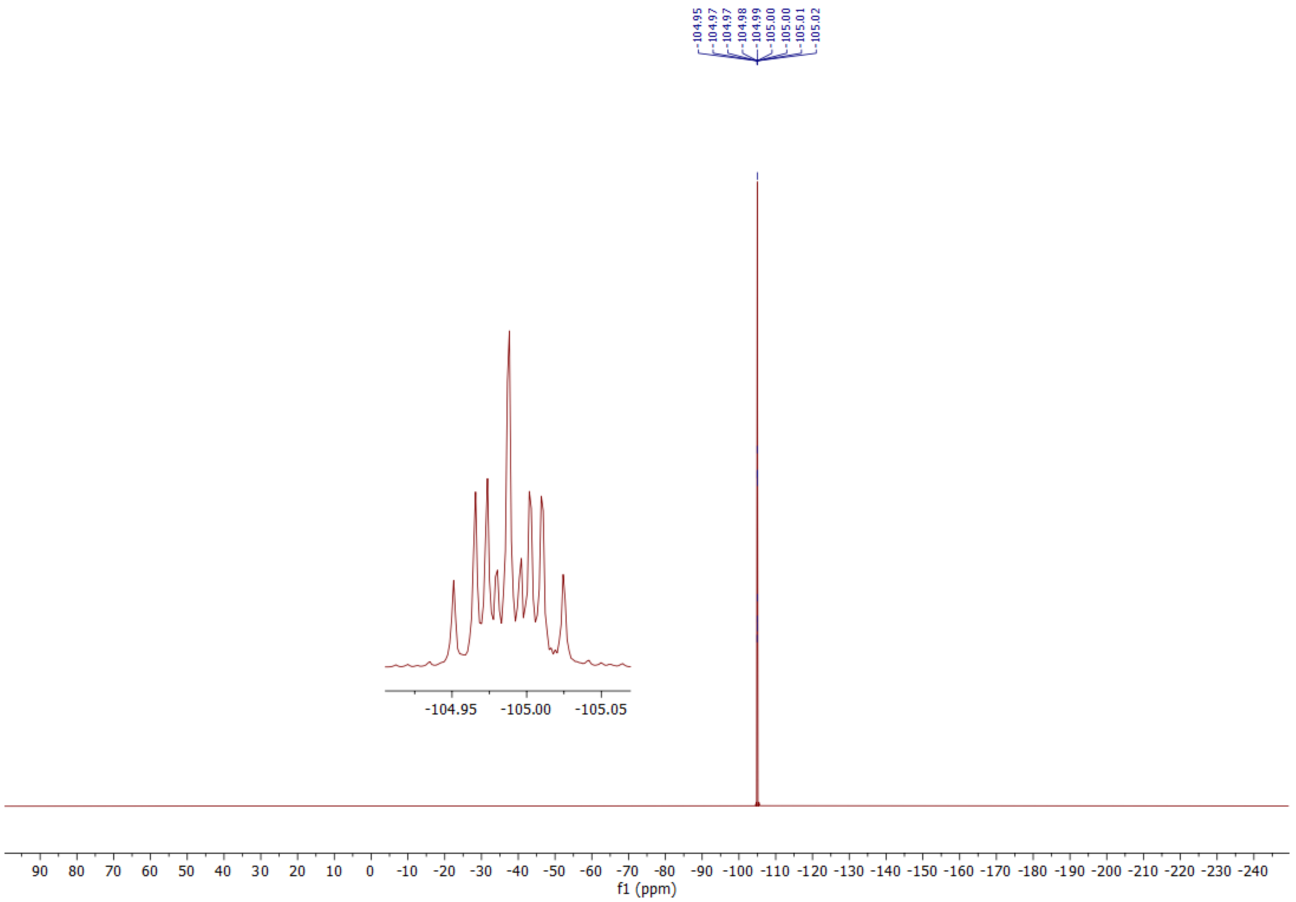

2a ^{1}H NMR (400 MHz, CDCl$_3$)

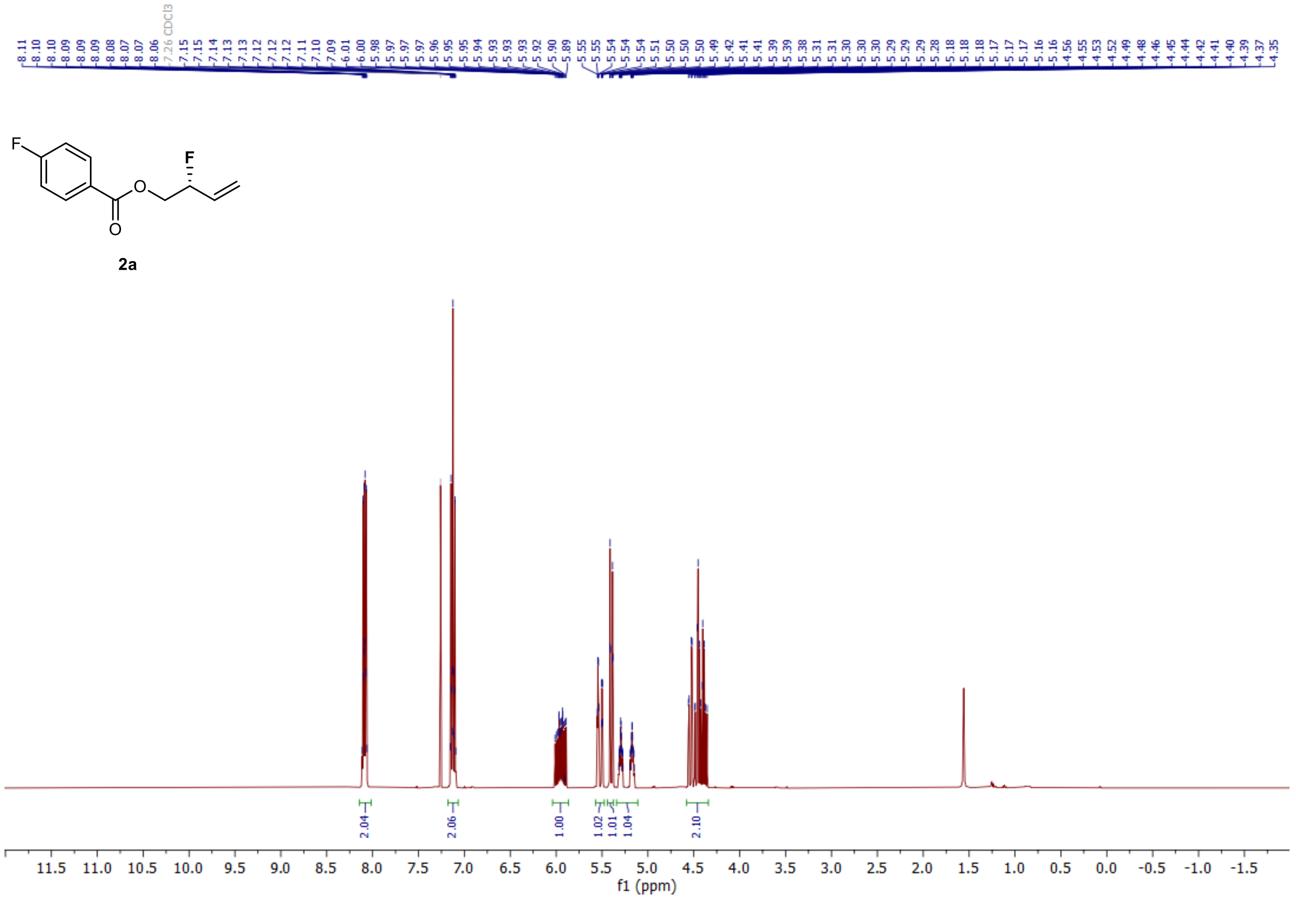

2a ^{13}C NMR (101 MHz, $CDCl_3$)

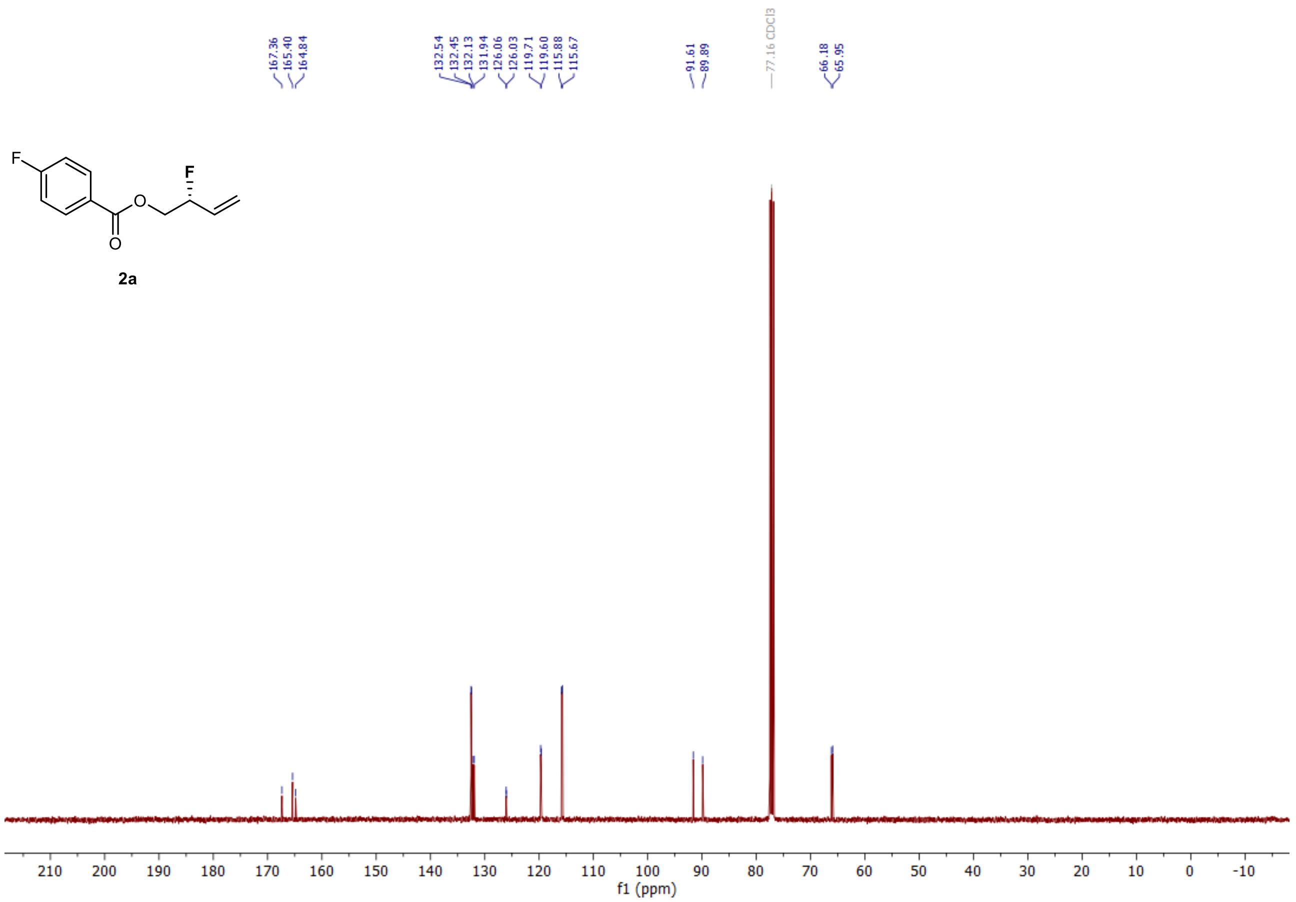

2a ^{19}F NMR (377 MHz, $CDCl_3$)

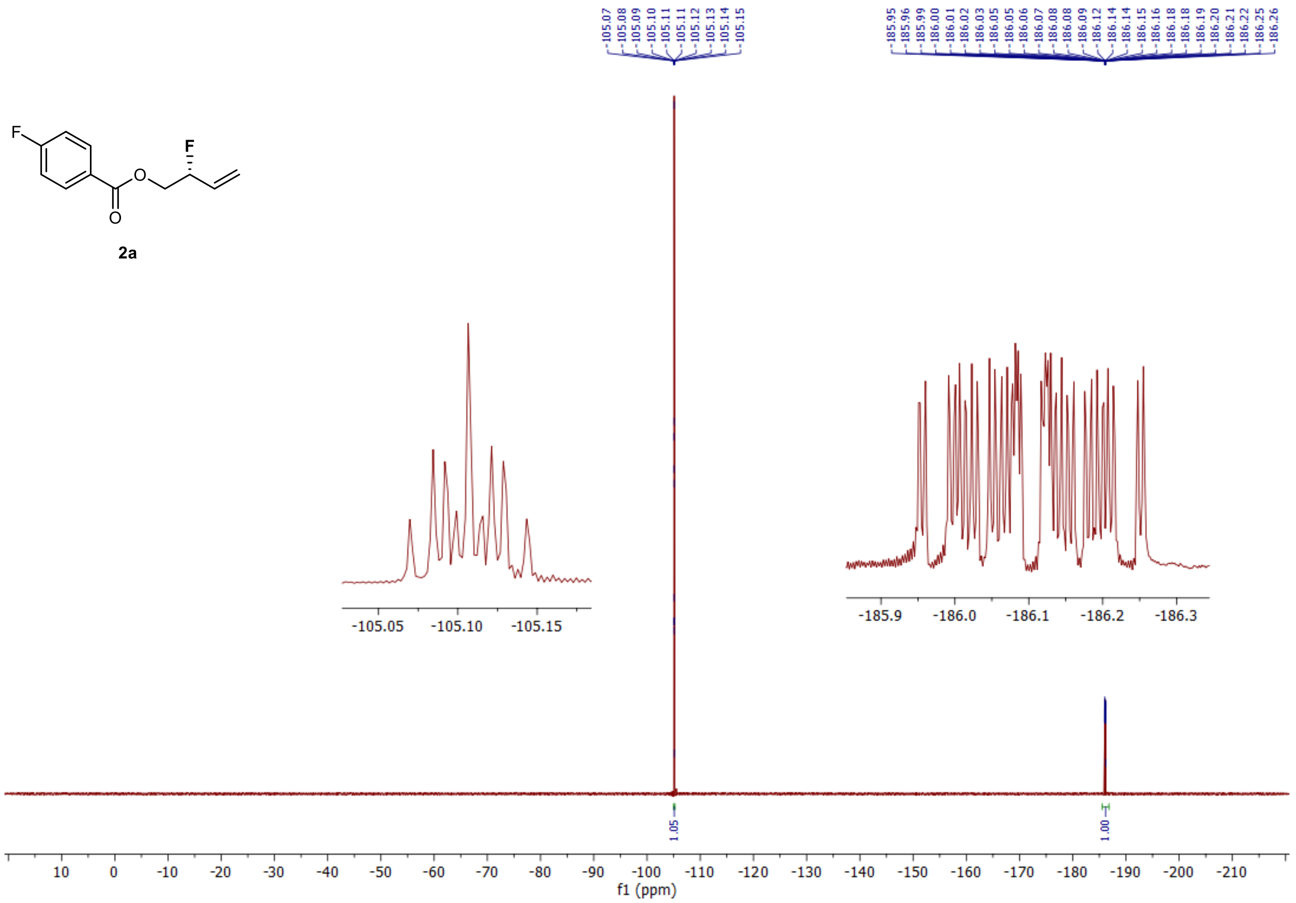

2b ^{1}H NMR (400 MHz, $CDCl_3$)

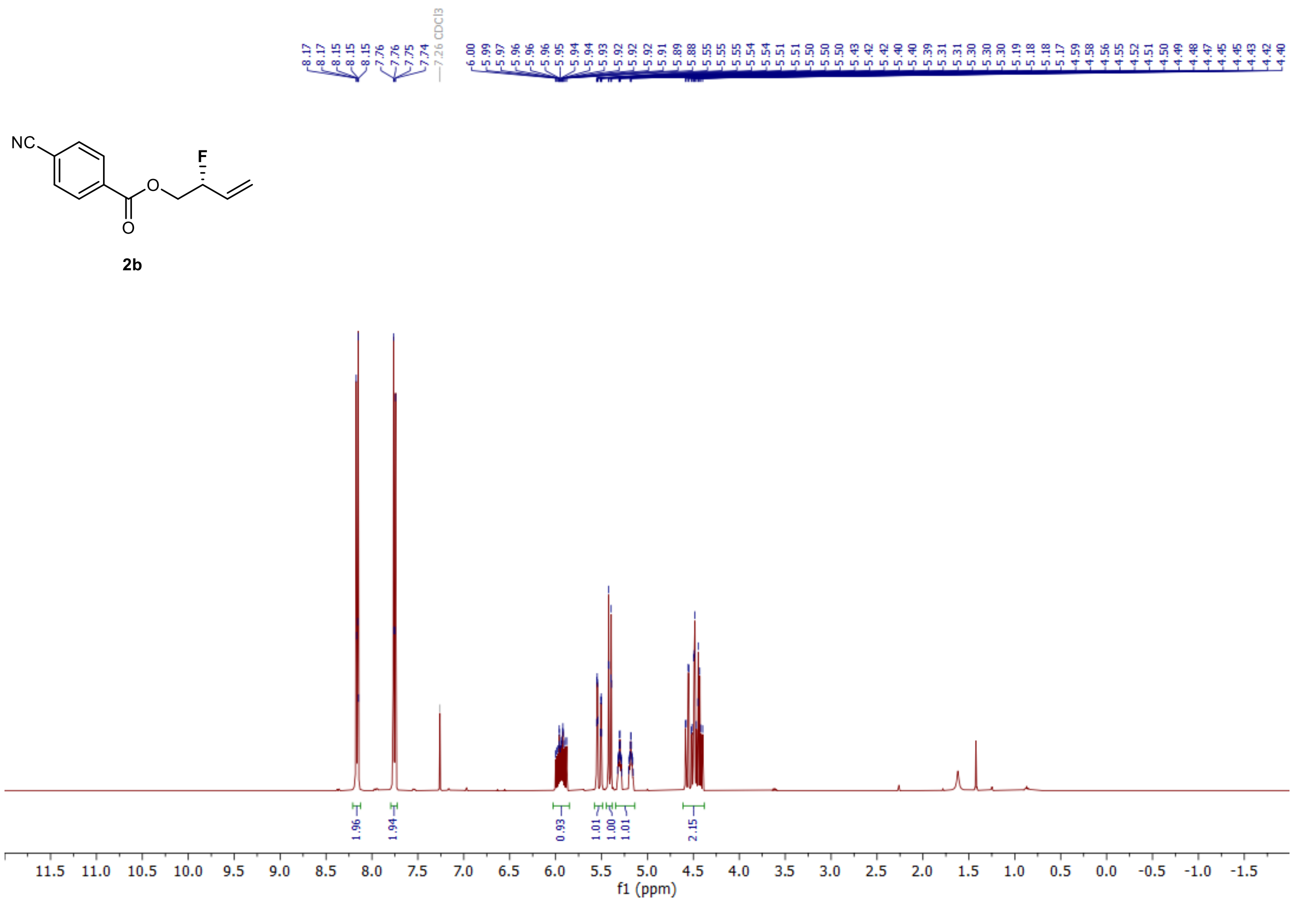

2b ^{13}C NMR (101 MHz, $CDCl_3$)

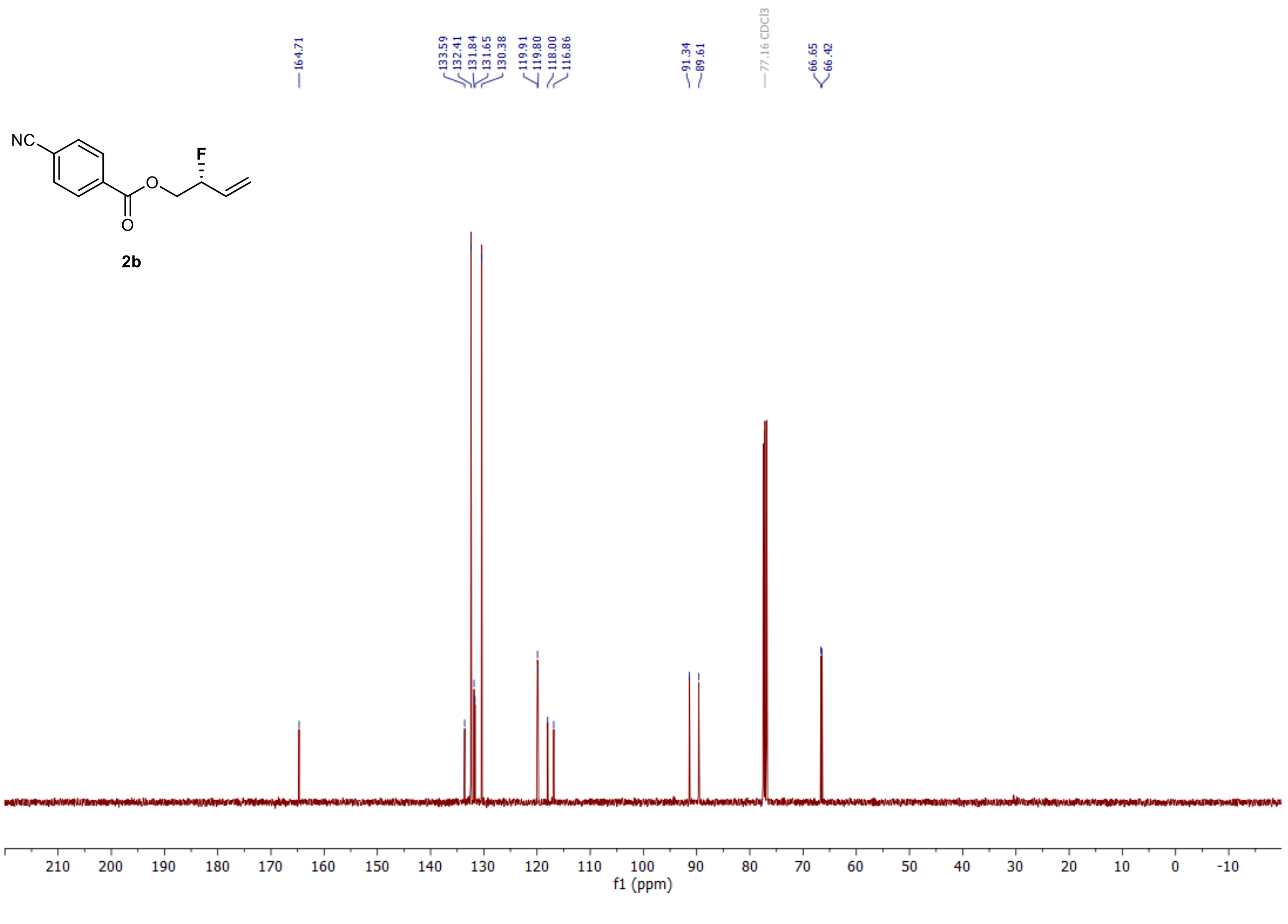

2b ^{19}F NMR (377 MHz, $CDCl_3$)

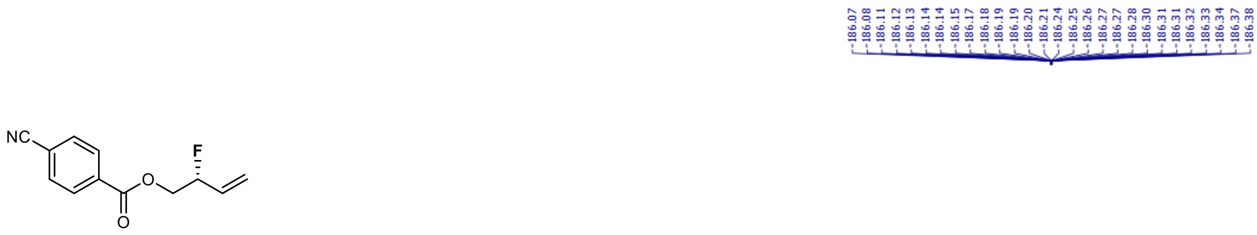

2b

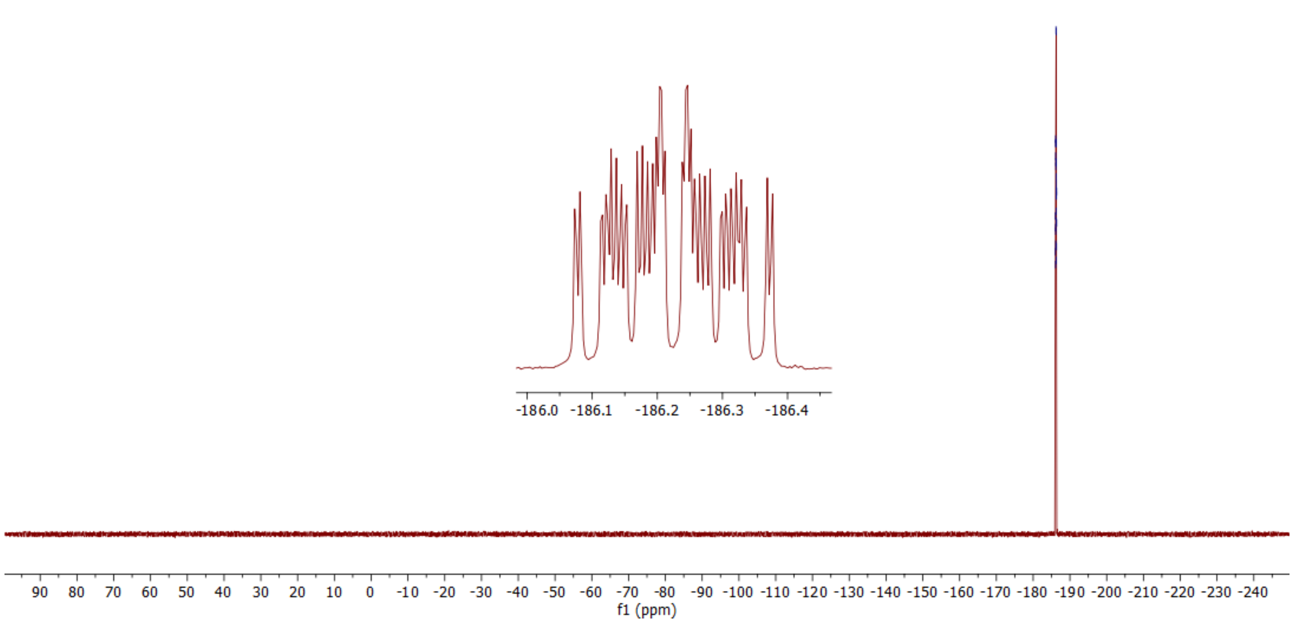

2c ^{1}H NMR (400 MHz, $CDCl_3$)

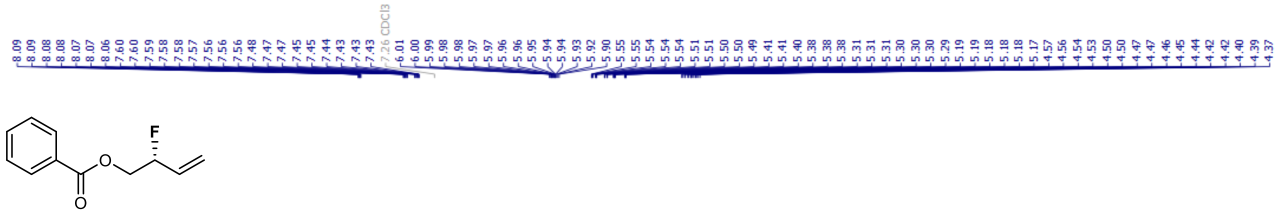

2c

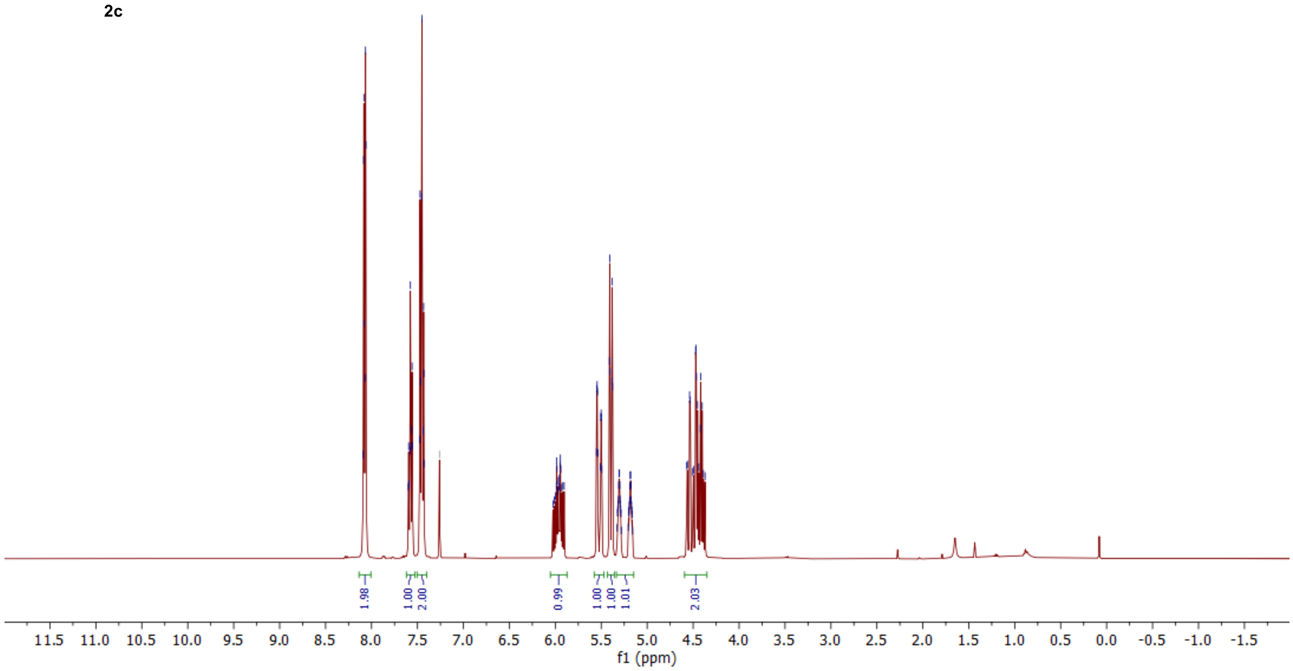

2c ^{13}C NMR (101 MHz, $CDCl_3$)

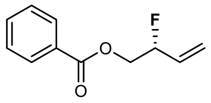

2c

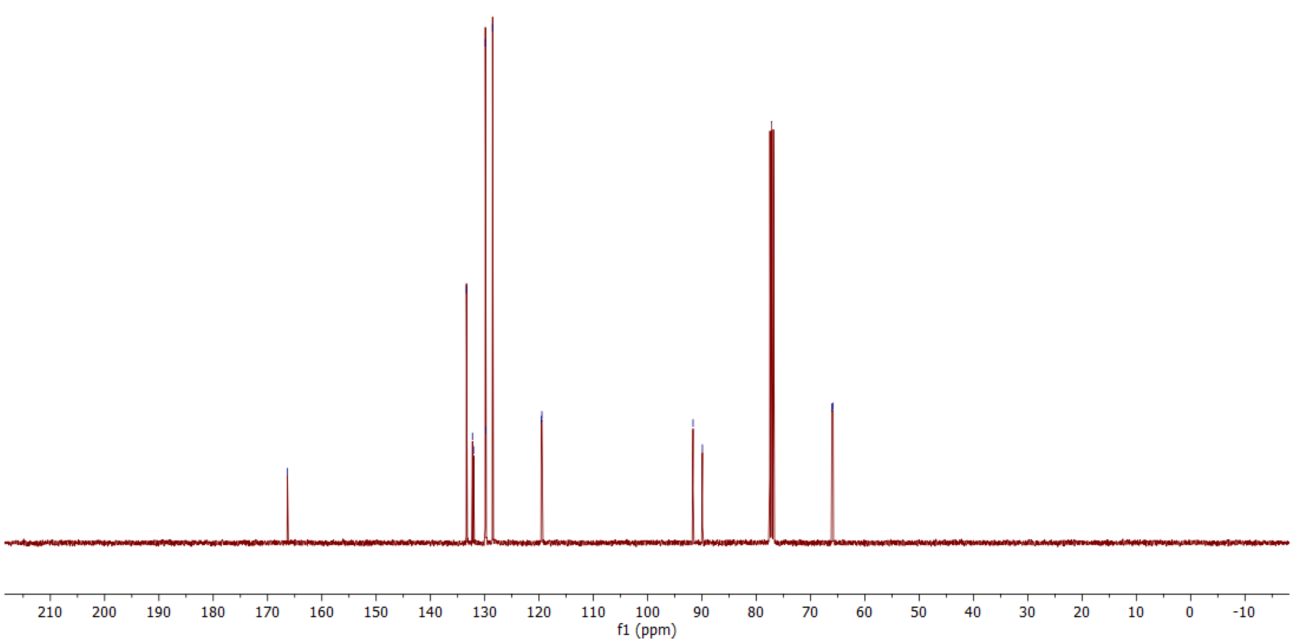

2c ^{19}F NMR (377 MHz, $CDCl_3$)

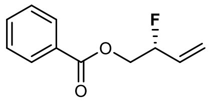

2c

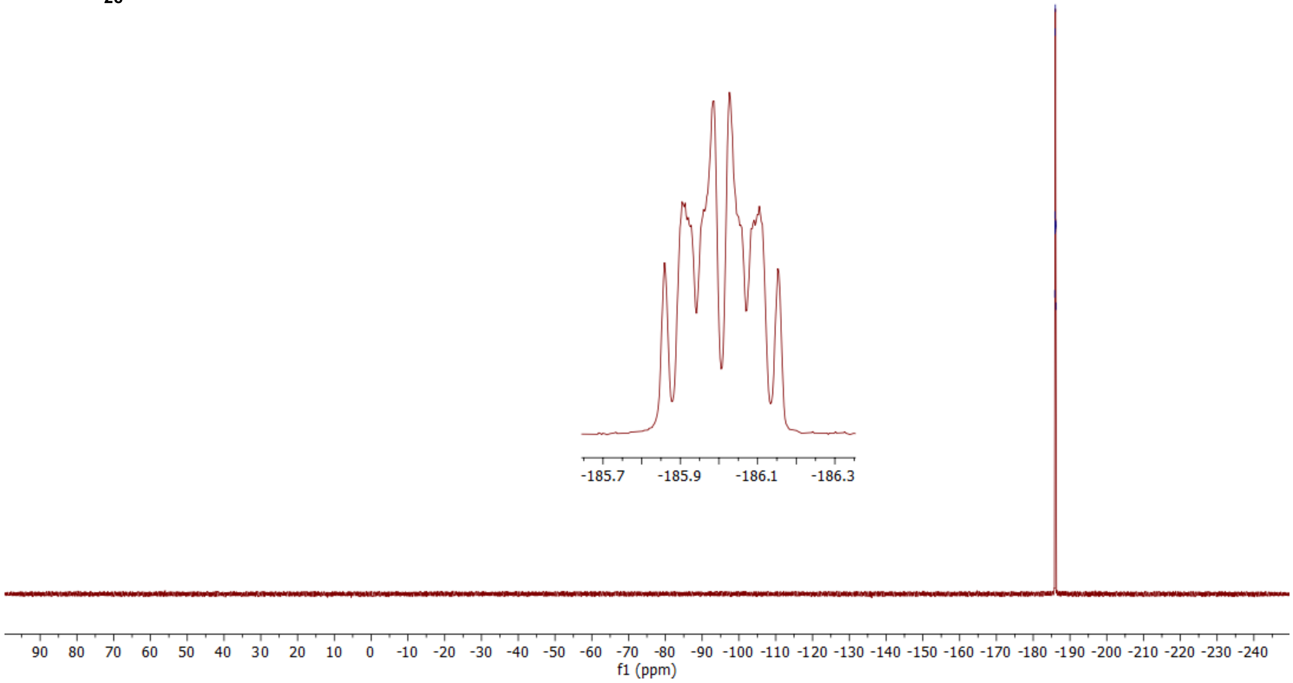

2d ^{1}H NMR (400 MHz, $CDCl_3$)

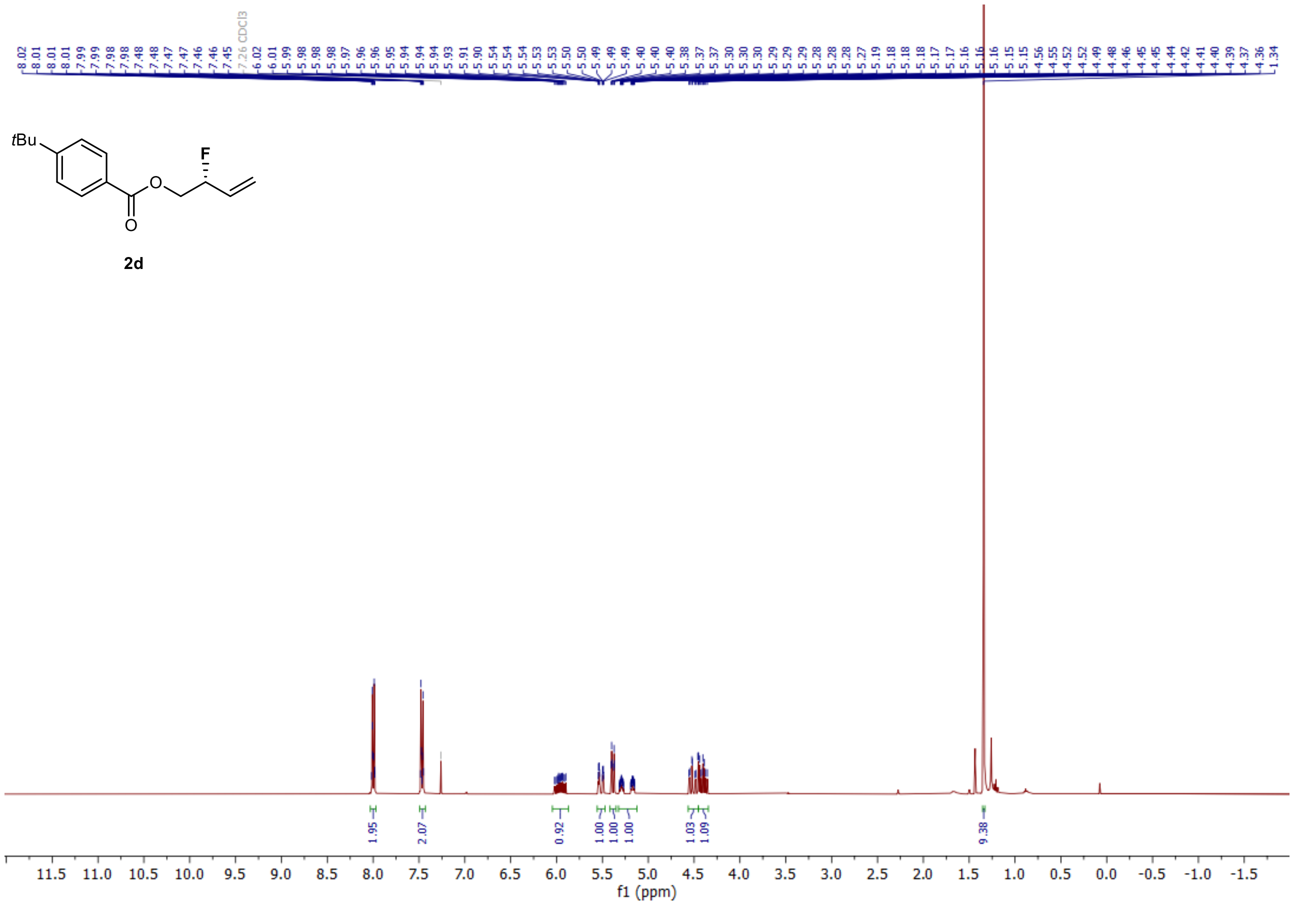

2d ^{13}C NMR (101 MHz, $CDCl_3$)

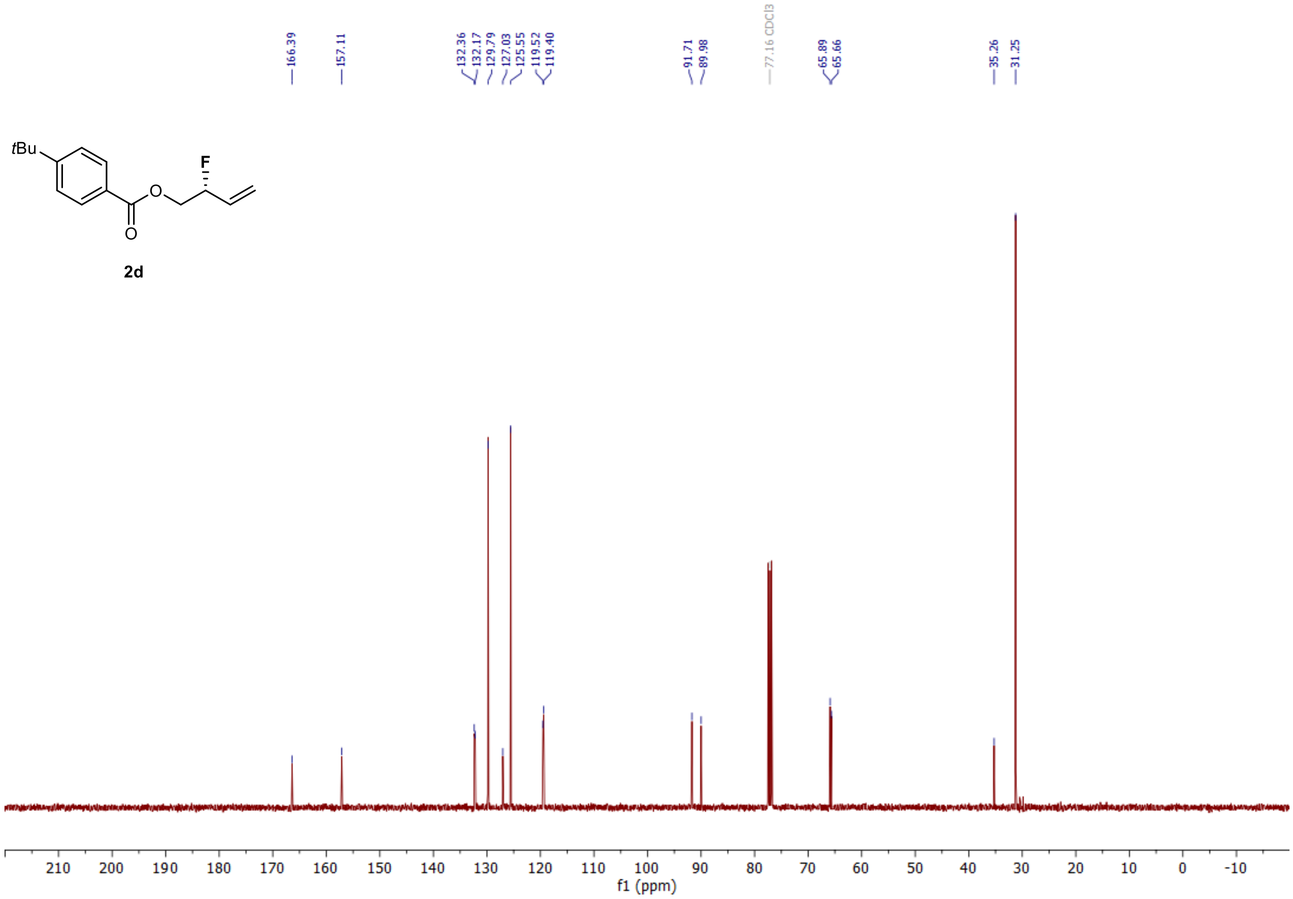

2d ^{19}F NMR (377 MHz, CDCl$_3$)

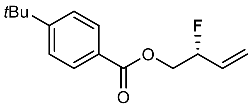

2d

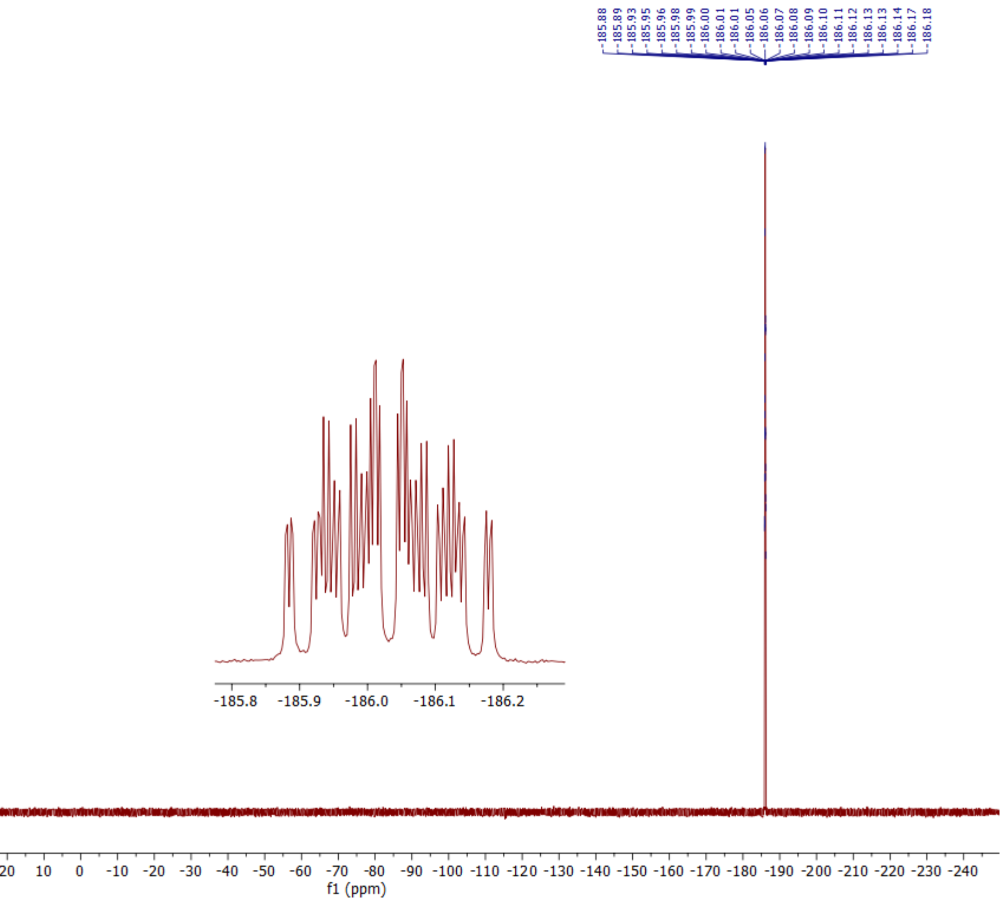

2e ^{1}H NMR (400 MHz, CDCl$_3$)

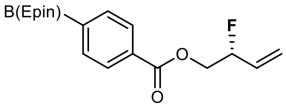

2e

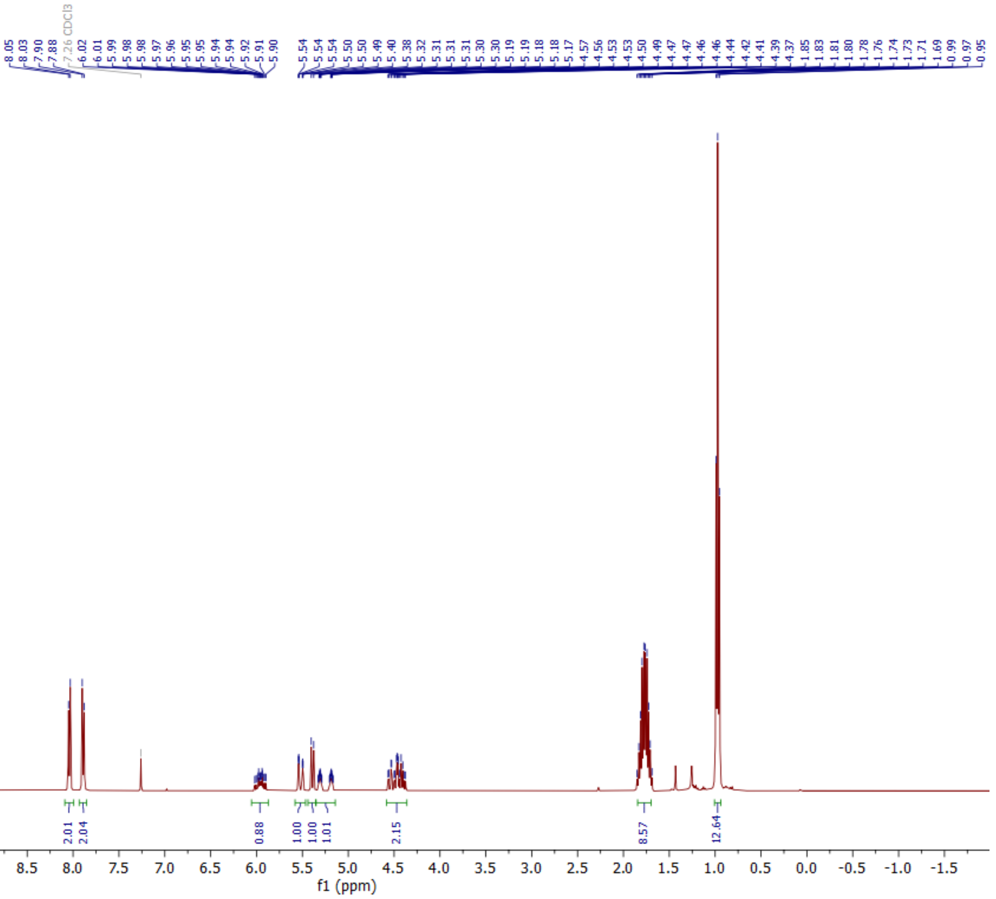

2e ^{13}C NMR (101 MHz, $CDCl_3$)

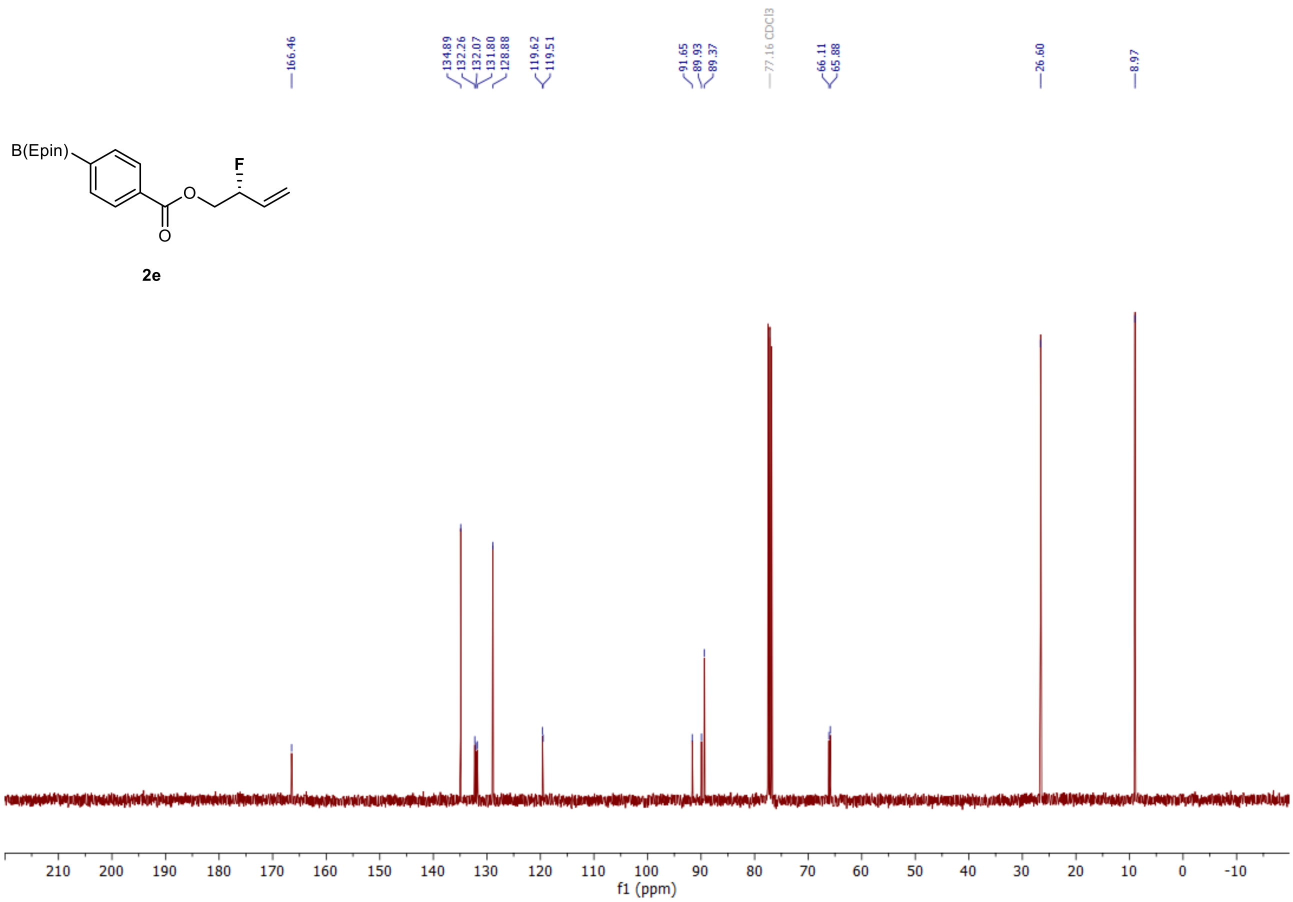

2e ^{19}F NMR (377 MHz, $CDCl_3$)

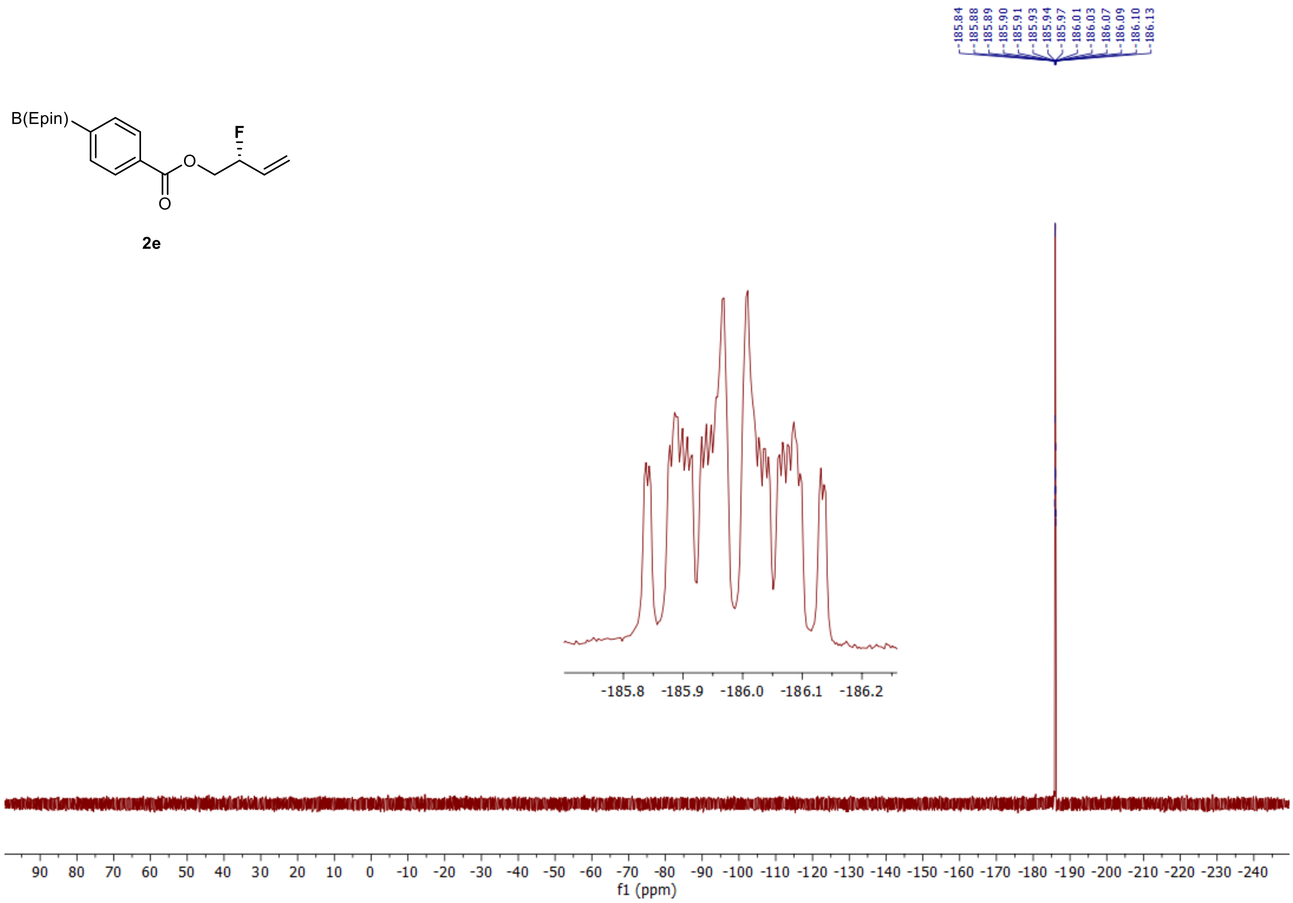

2f ^{1}H NMR (400 MHz, $CDCl_3$)

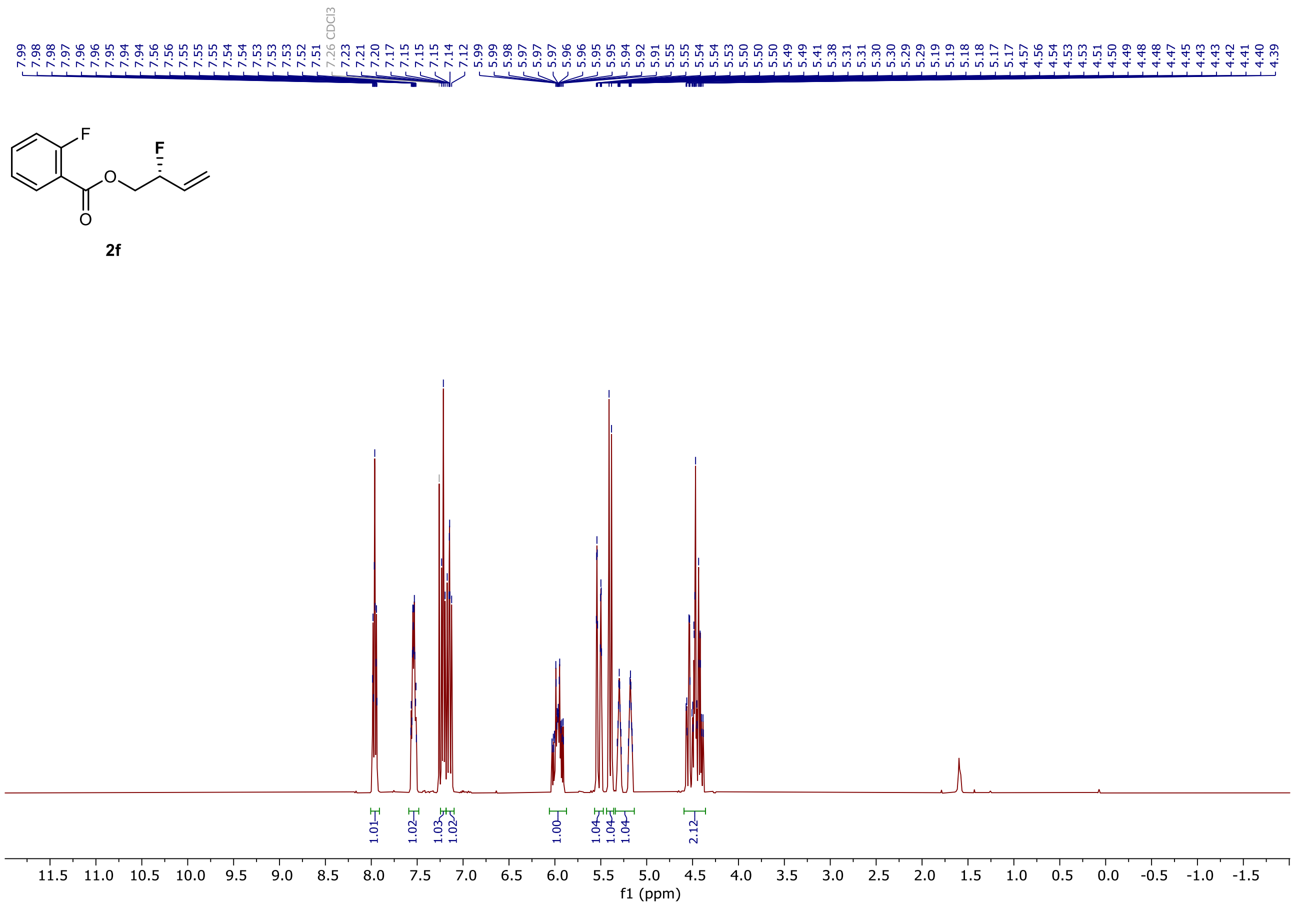

2f ^{13}C NMR (101 MHz, $CDCl_3$)

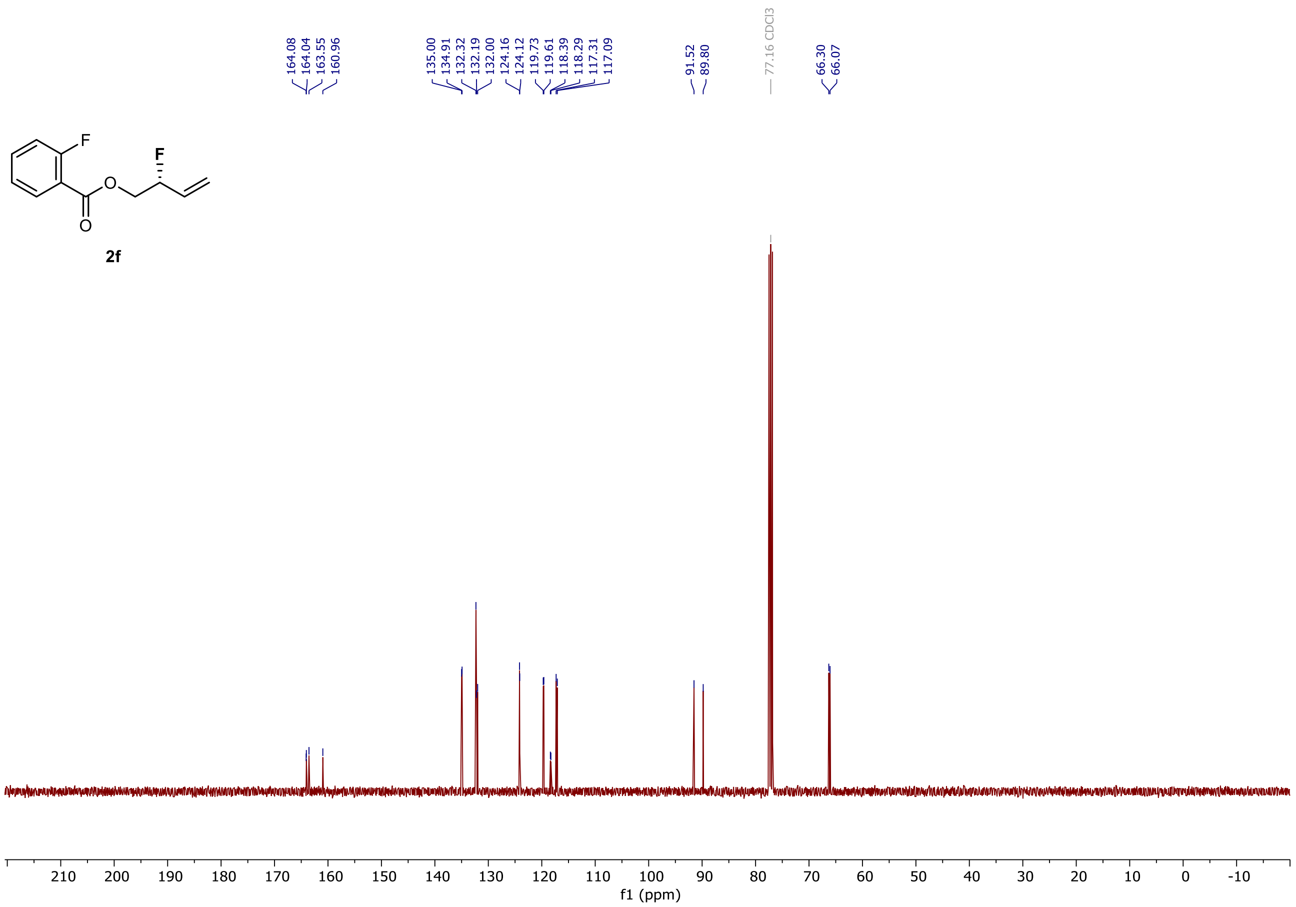

2f ^{19}F NMR (377 MHz, $CDCl_3$)

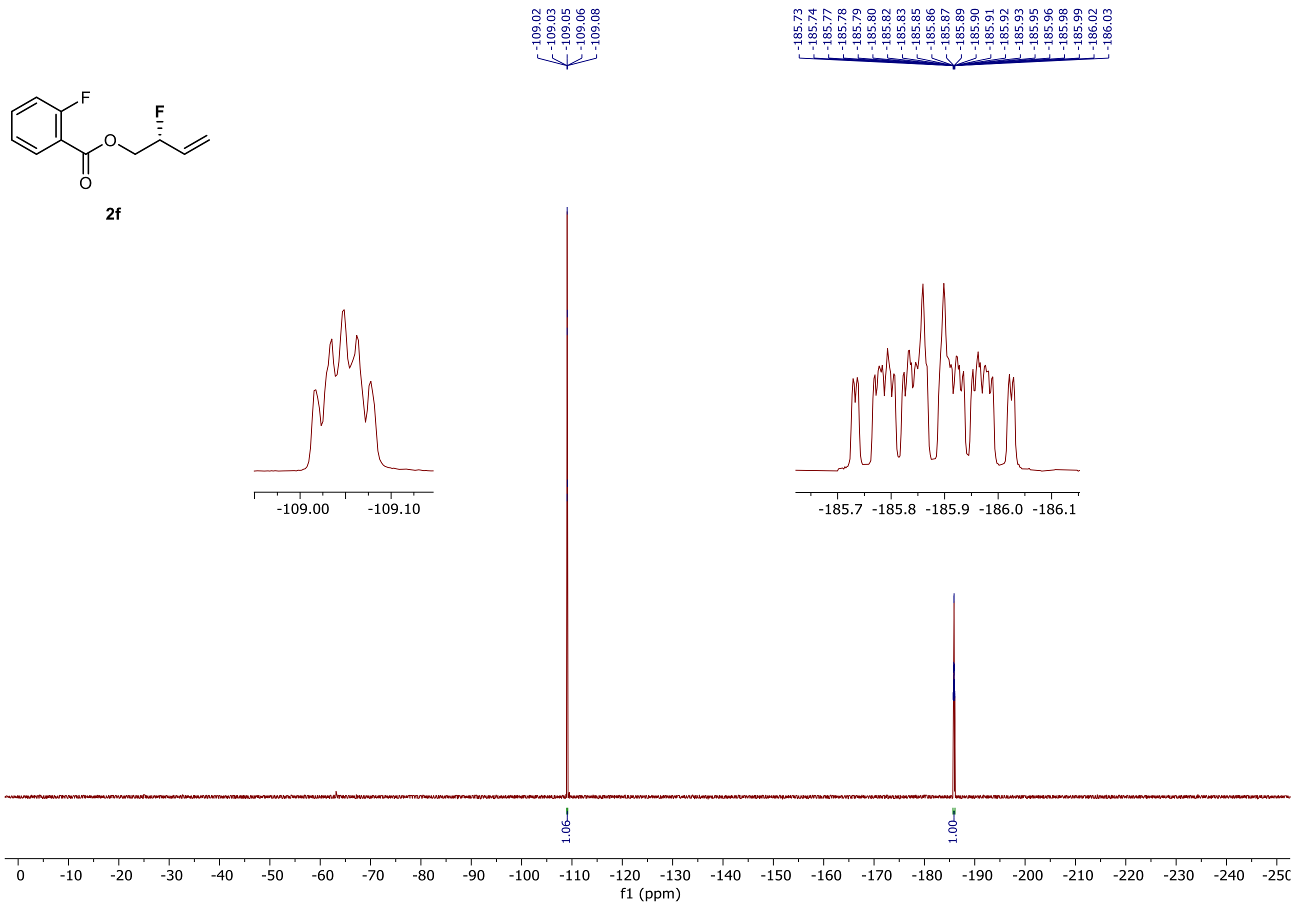

2g ^{1}H NMR (400 MHz, $CDCl_3$)

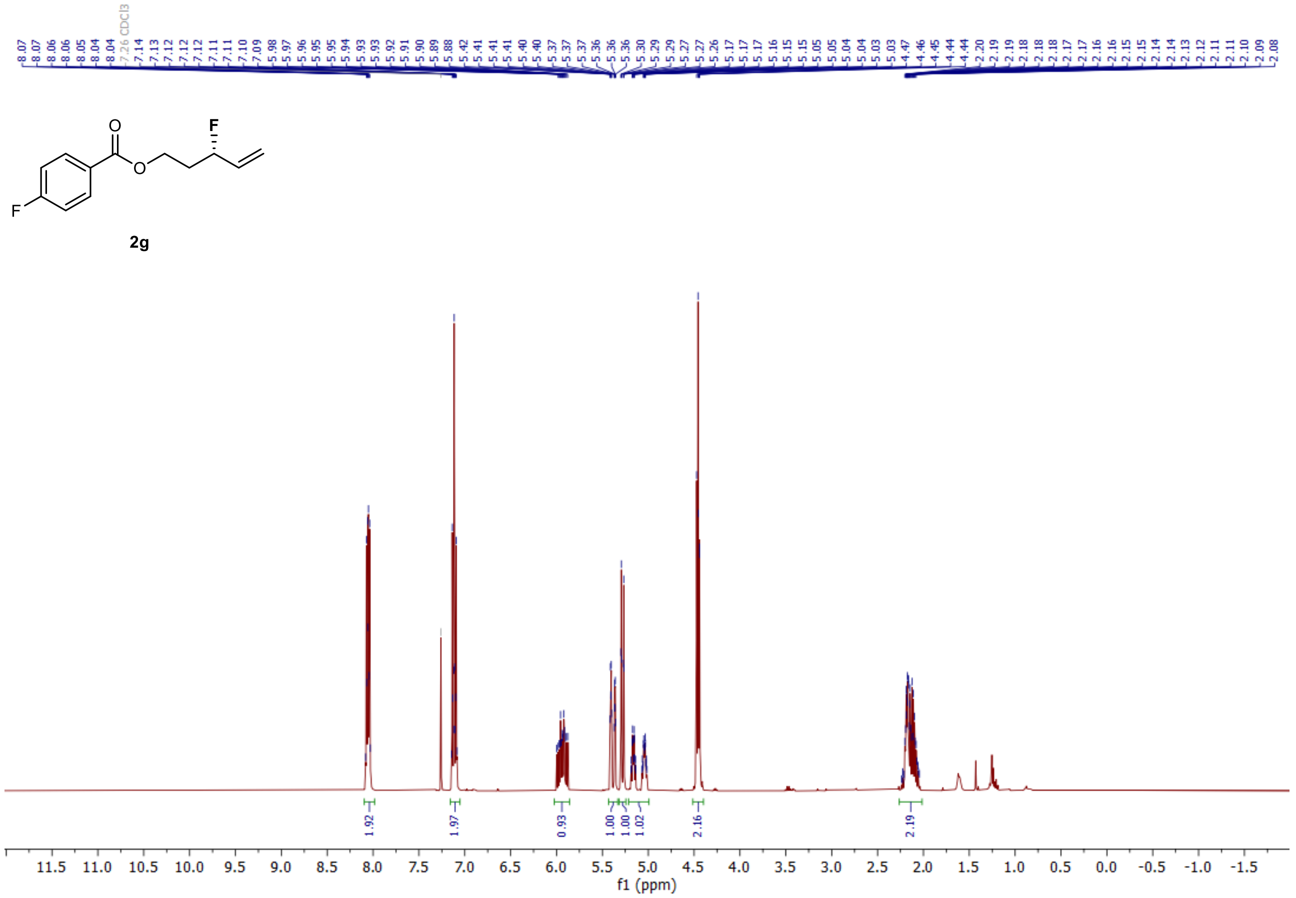

2g ^{13}C NMR (101 MHz, $CDCl_3$)

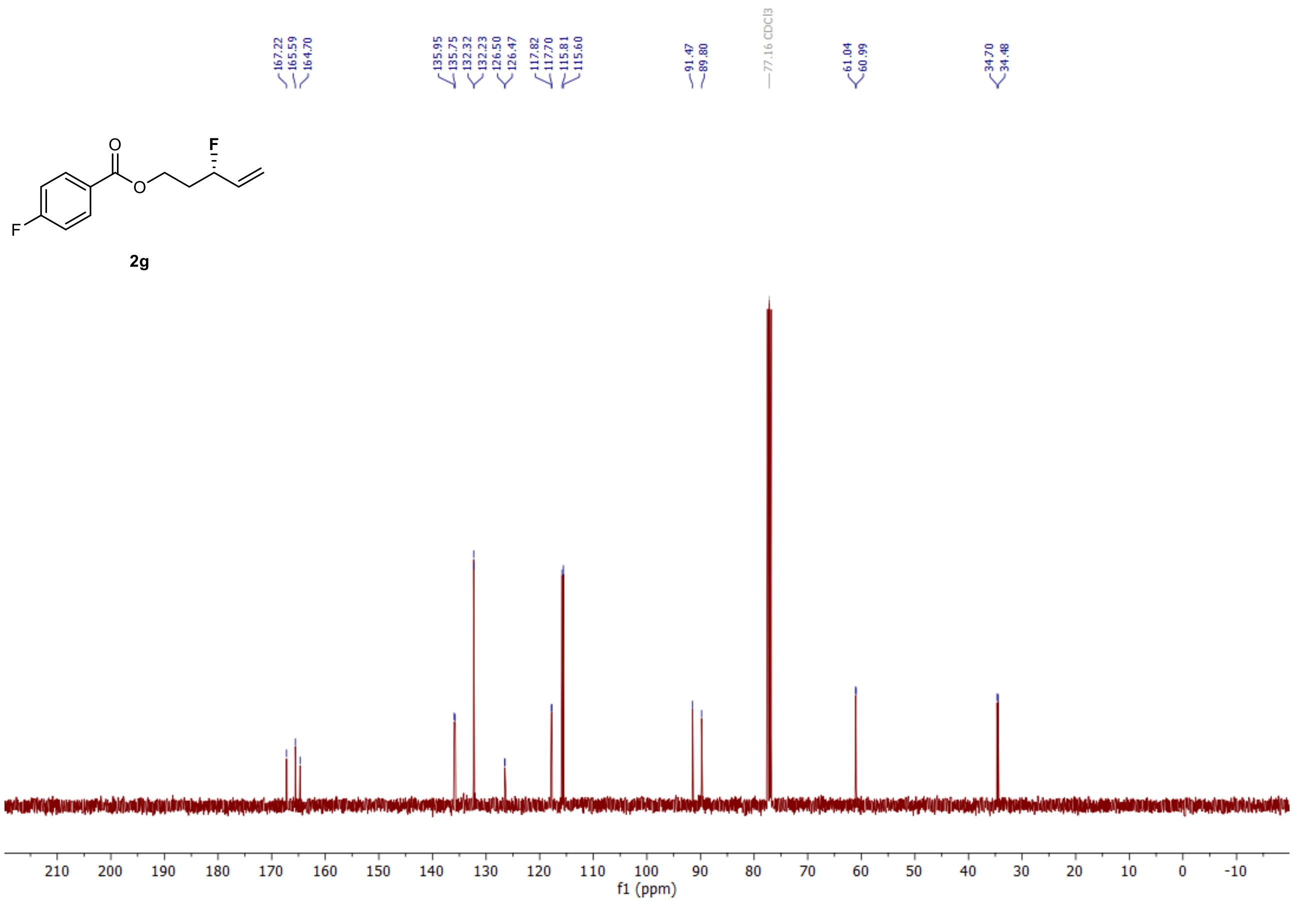

2g ^{19}F NMR (377 MHz, $CDCl_3$)

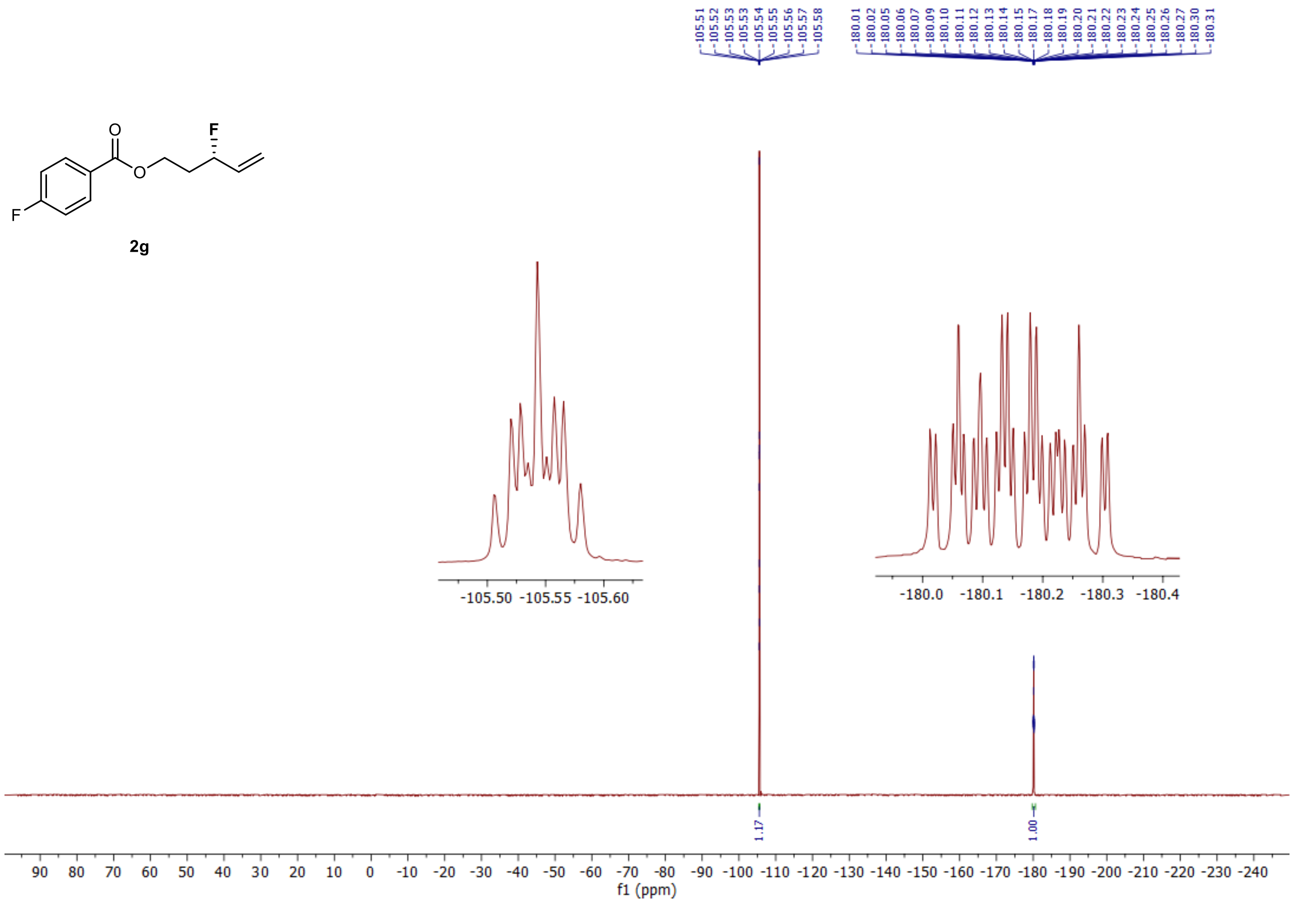

2h ^{1}H NMR (400 MHz, $CDCl_3$)

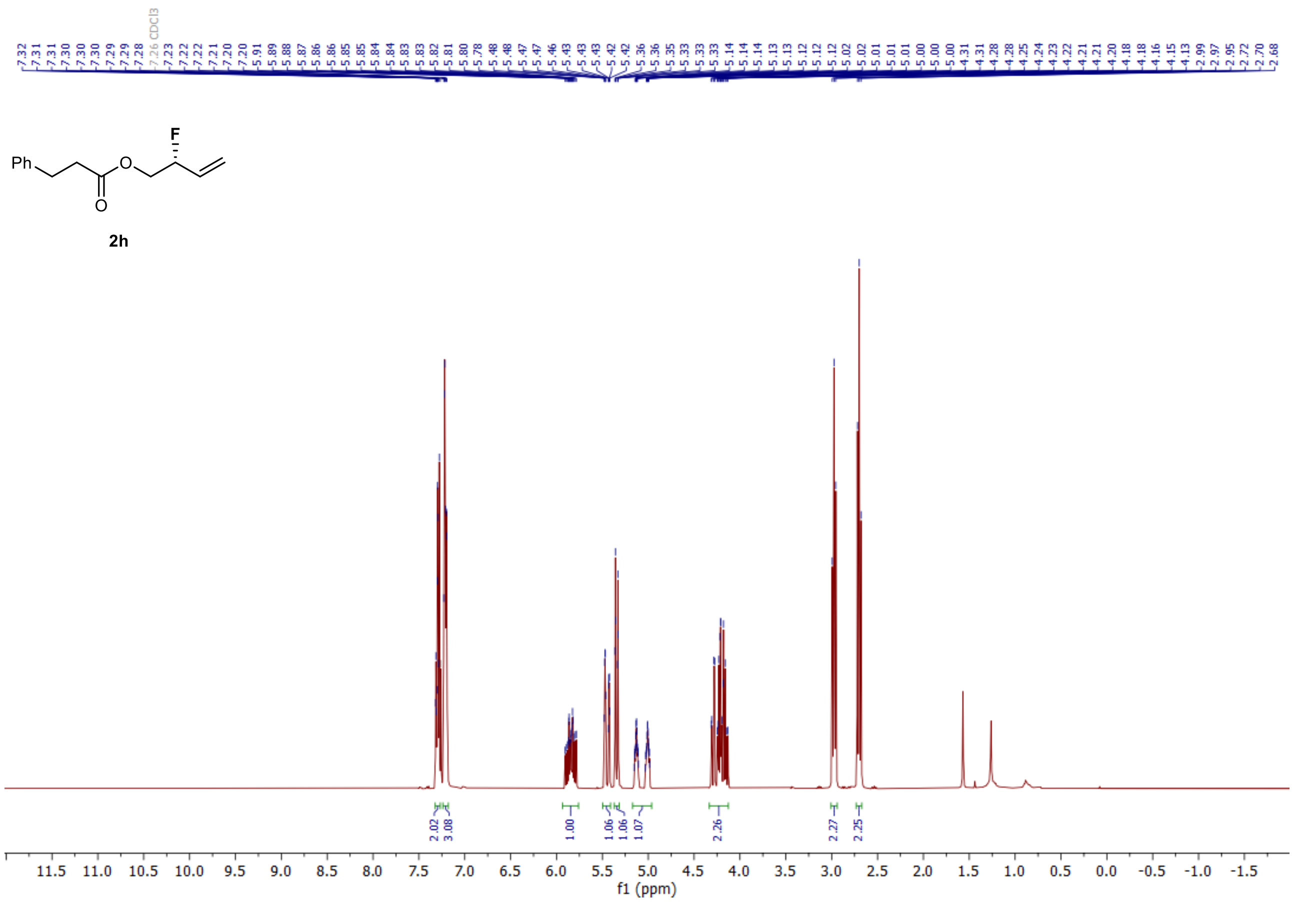

2h ^{13}C NMR (101 MHz, $CDCl_3$)

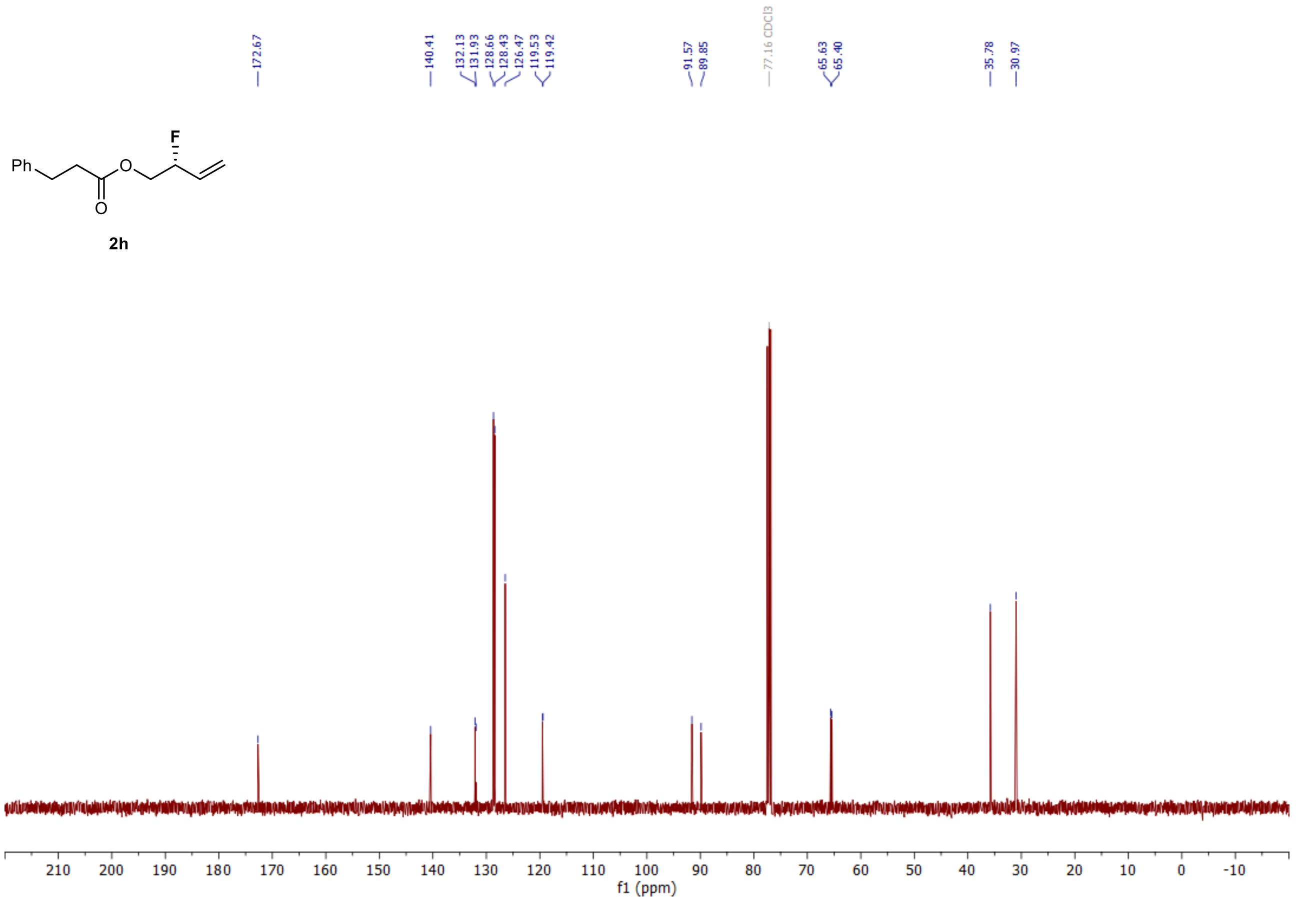

2h ^{19}F NMR (377 MHz, $CDCl_3$)

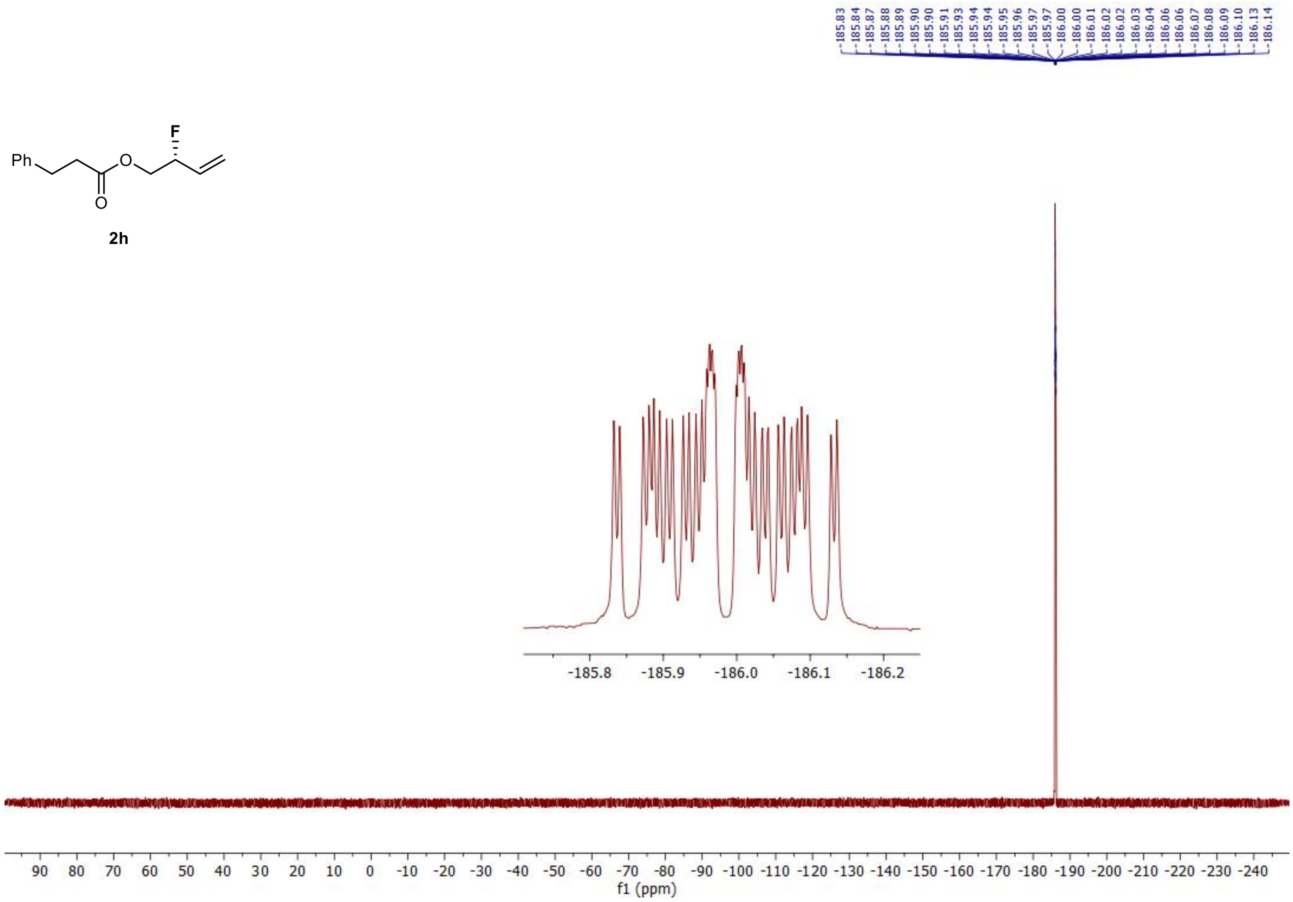

2i ^{1}H NMR (400 MHz, $CDCl_3$)

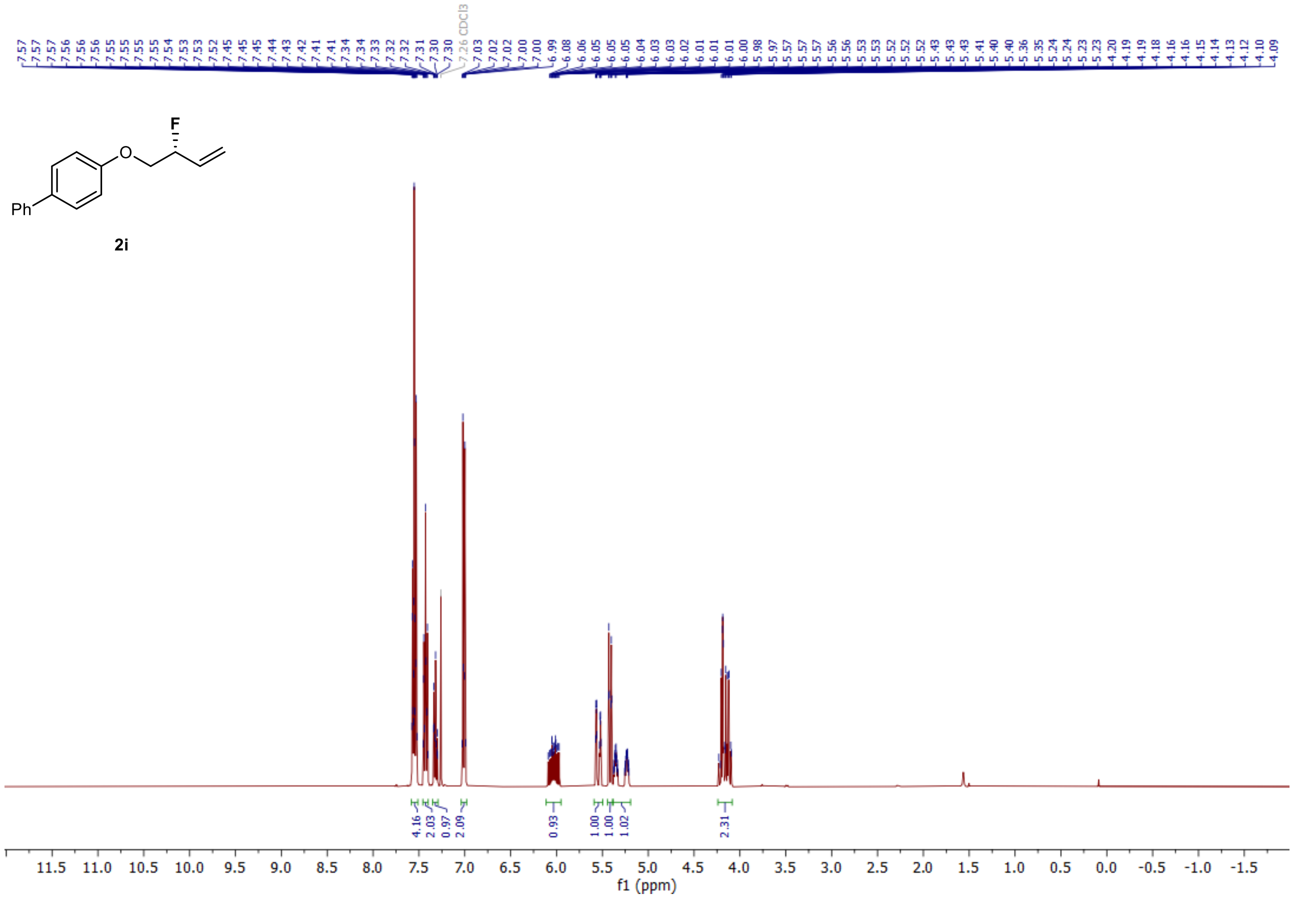

2i ^{13}C NMR (101 MHz, $CDCl_3$)

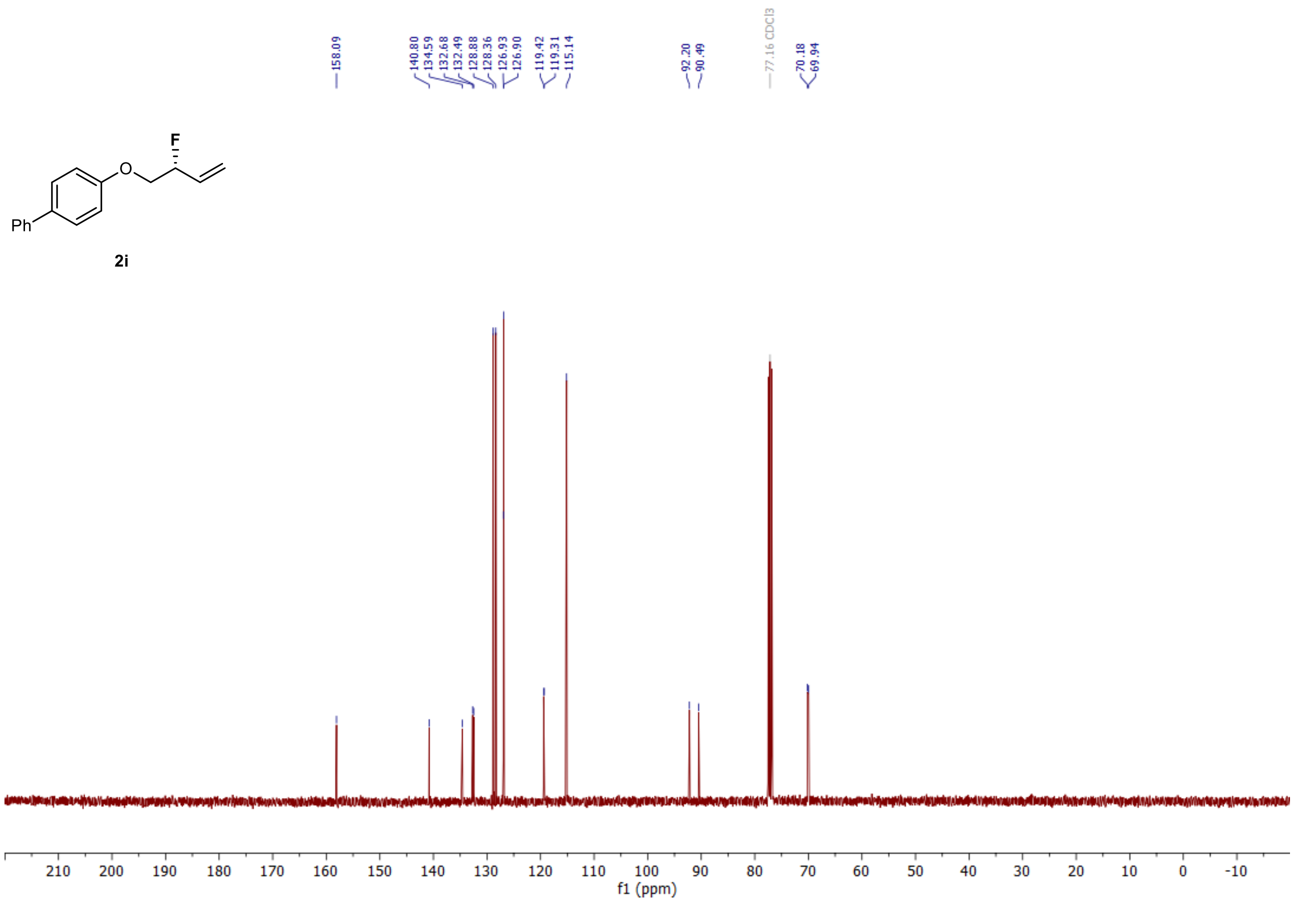

2i ^{19}F NMR (377 MHz, $CDCl_3$)

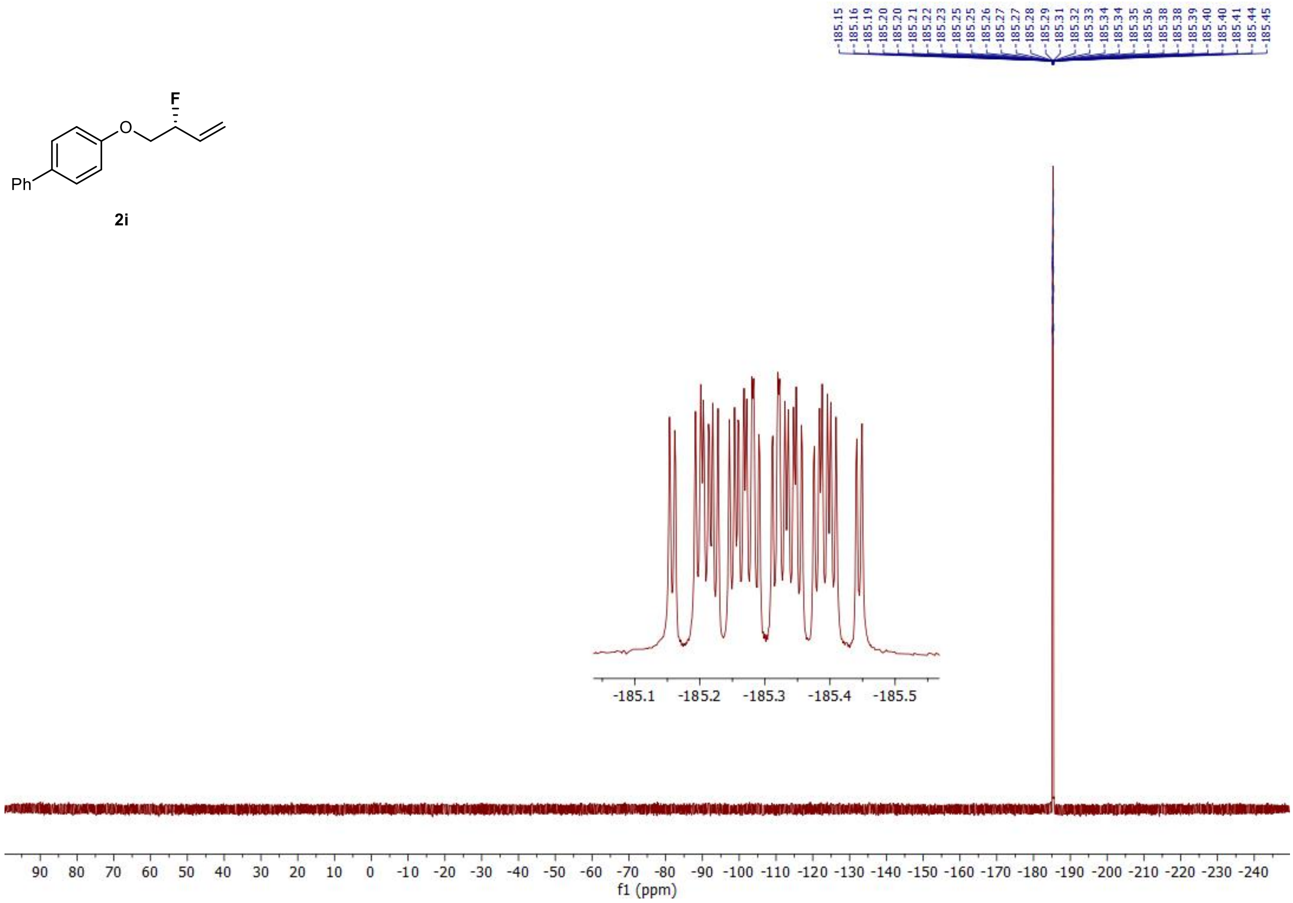

2j ^{1}H NMR (400 MHz, $CDCl_3$)

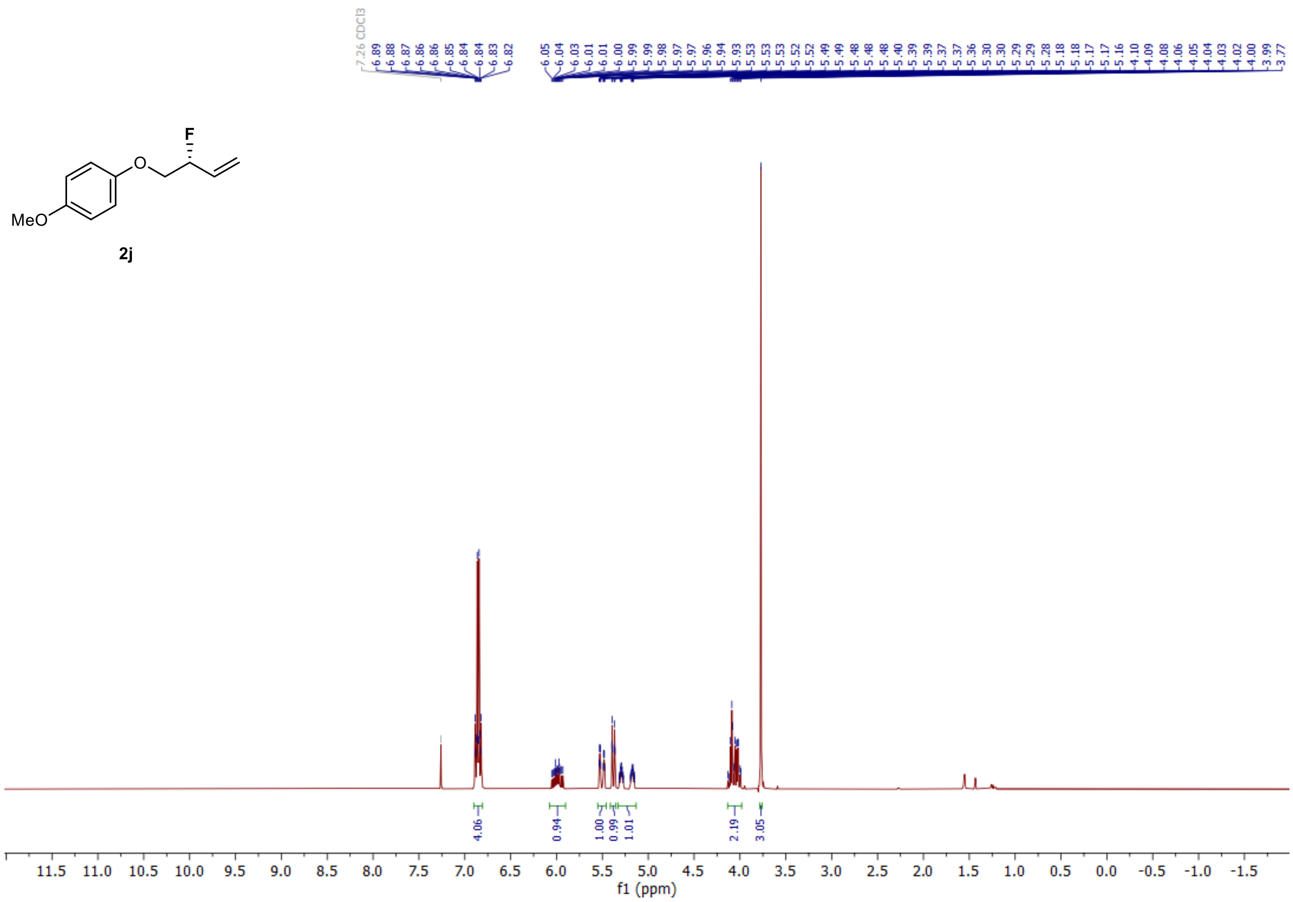

2j ^{13}C NMR (101 MHz, $CDCl_3$)

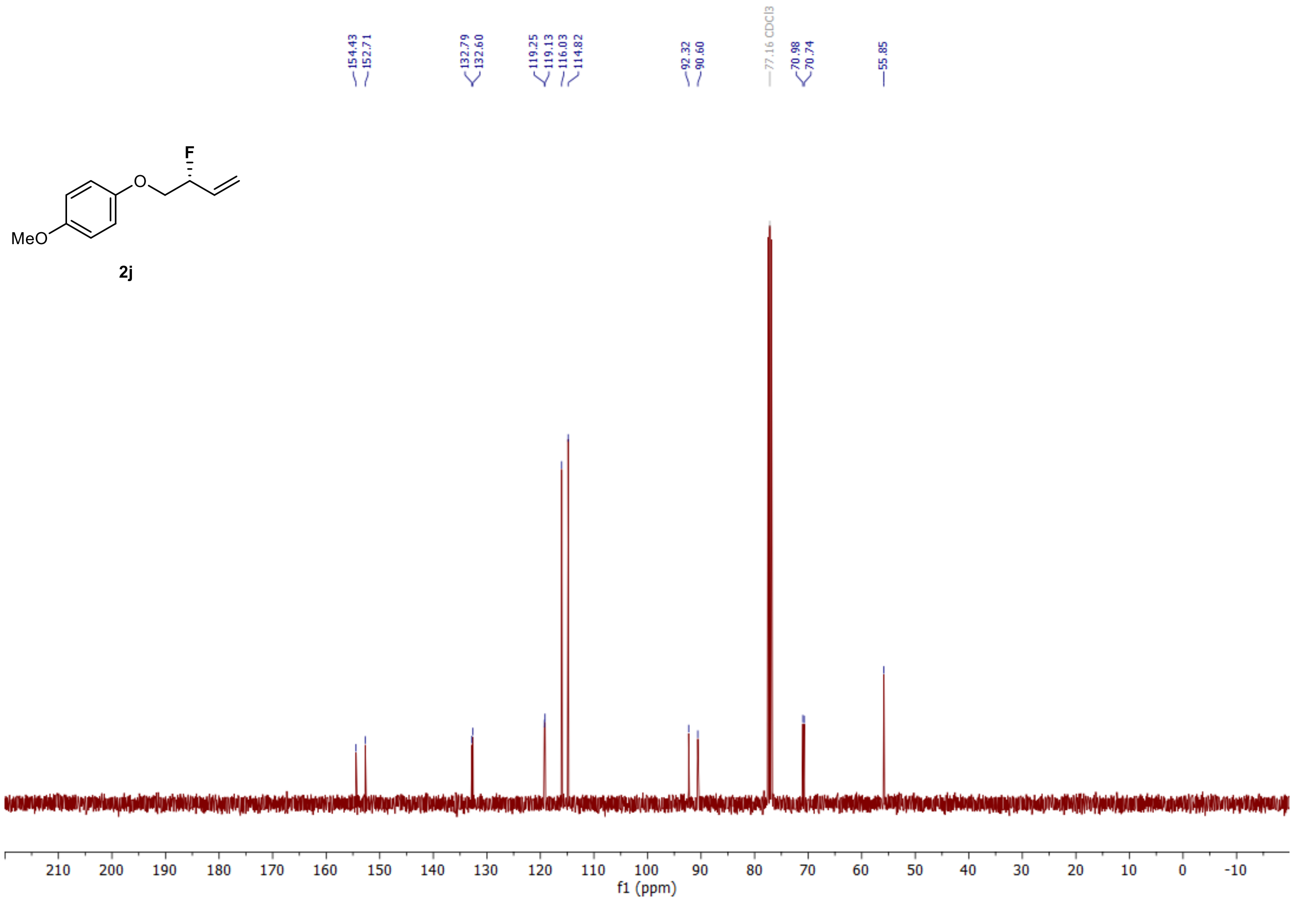

2j ^{19}F NMR (377 MHz, $CDCl_3$)

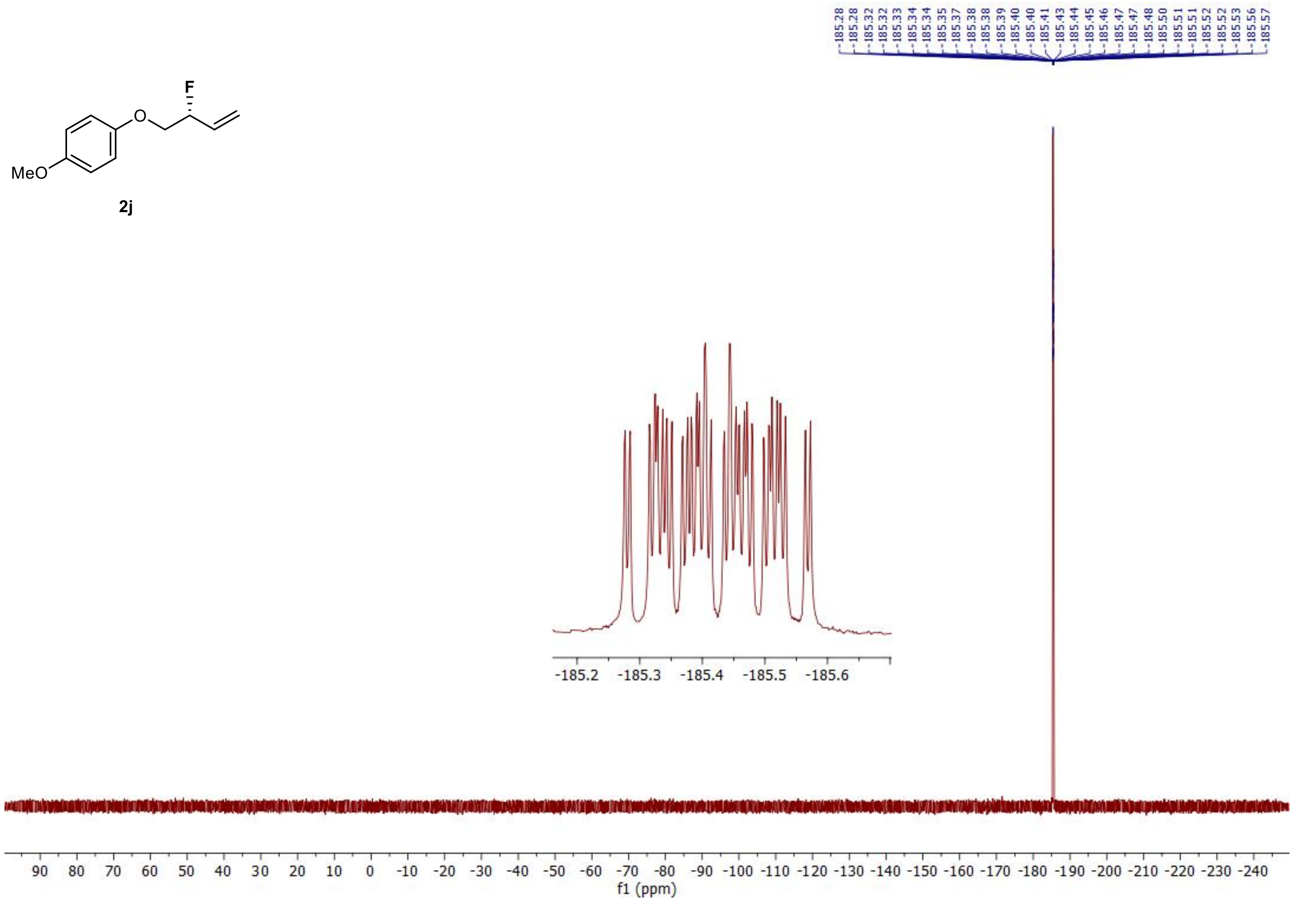

2k ^{1}H NMR (400 MHz, $CDCl_3$)

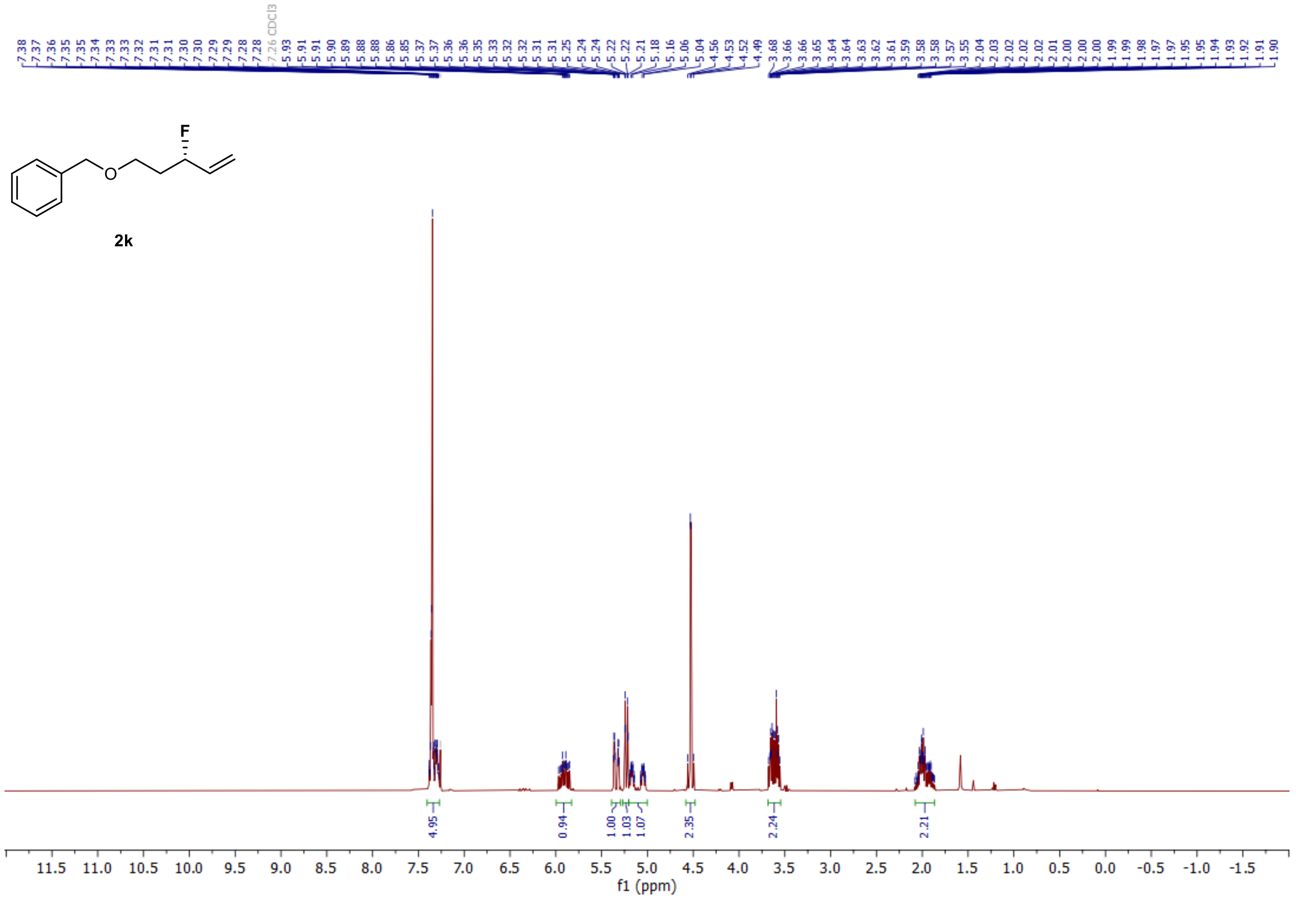

2k ^{13}C NMR (101 MHz, $CDCl_3$)

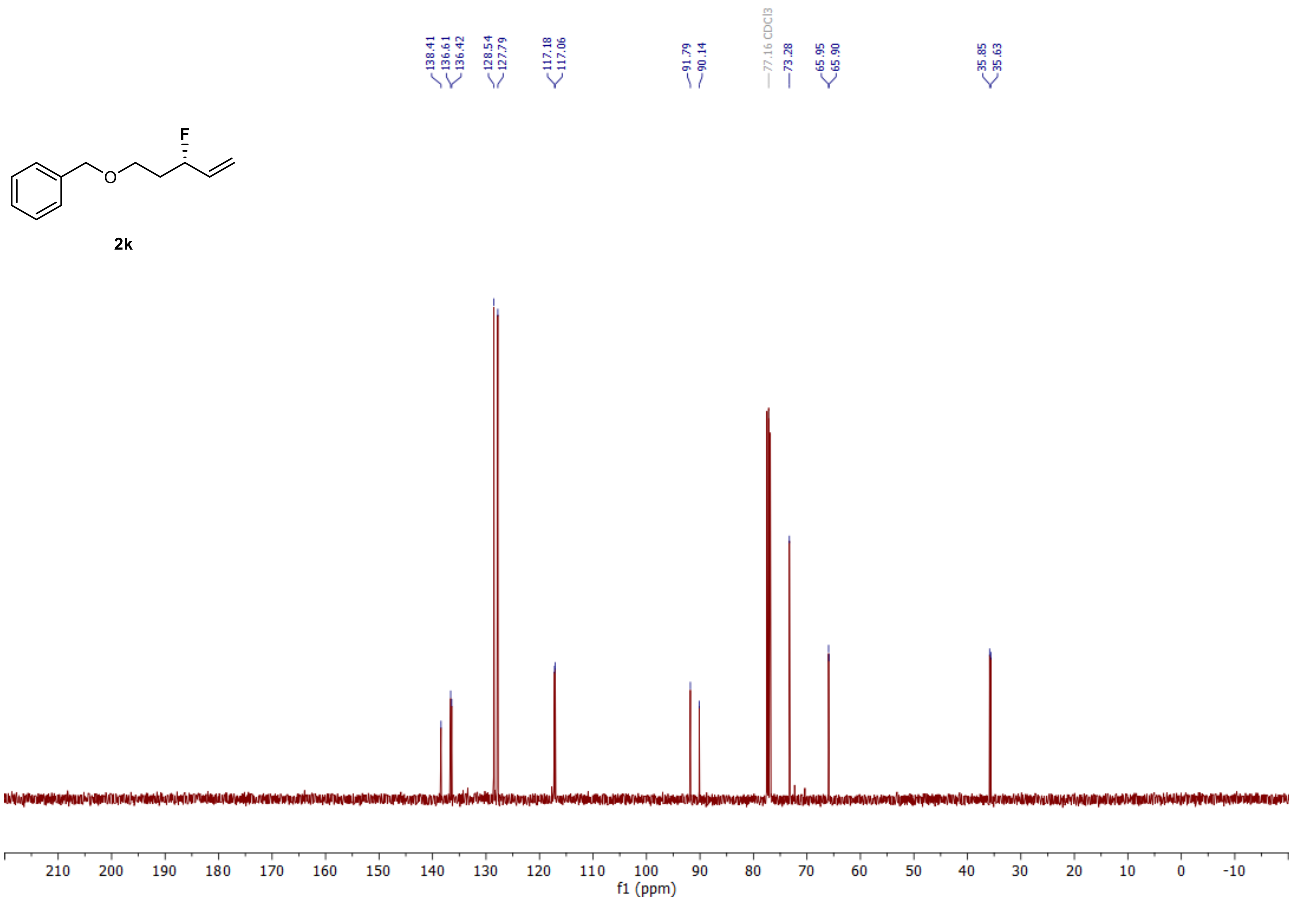

2k ^{19}F NMR (377 MHz, $CDCl_3$)

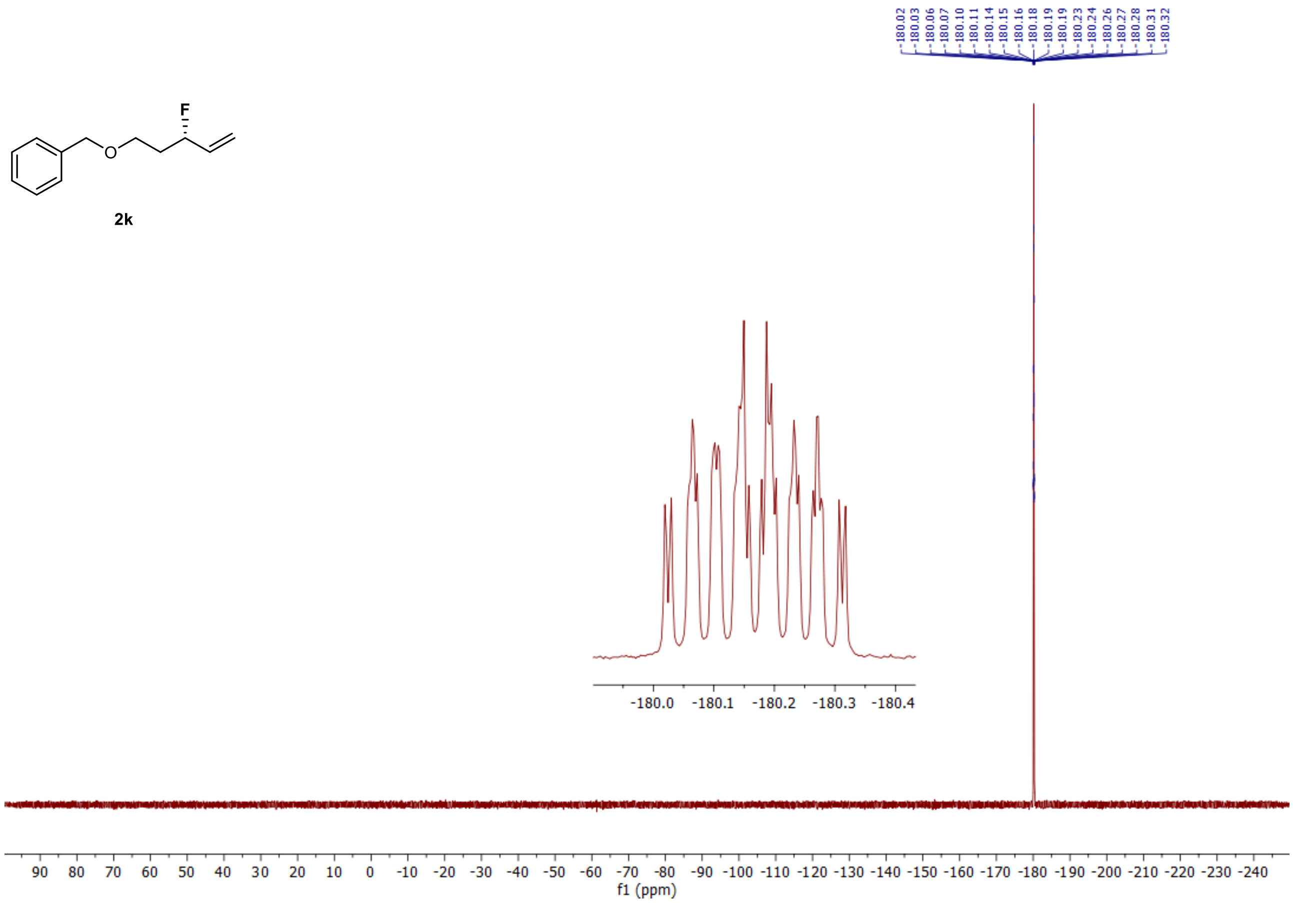

2l ^{1}H NMR (400 MHz, $CDCl_3$)

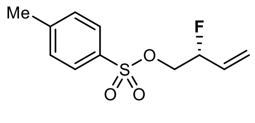

2l

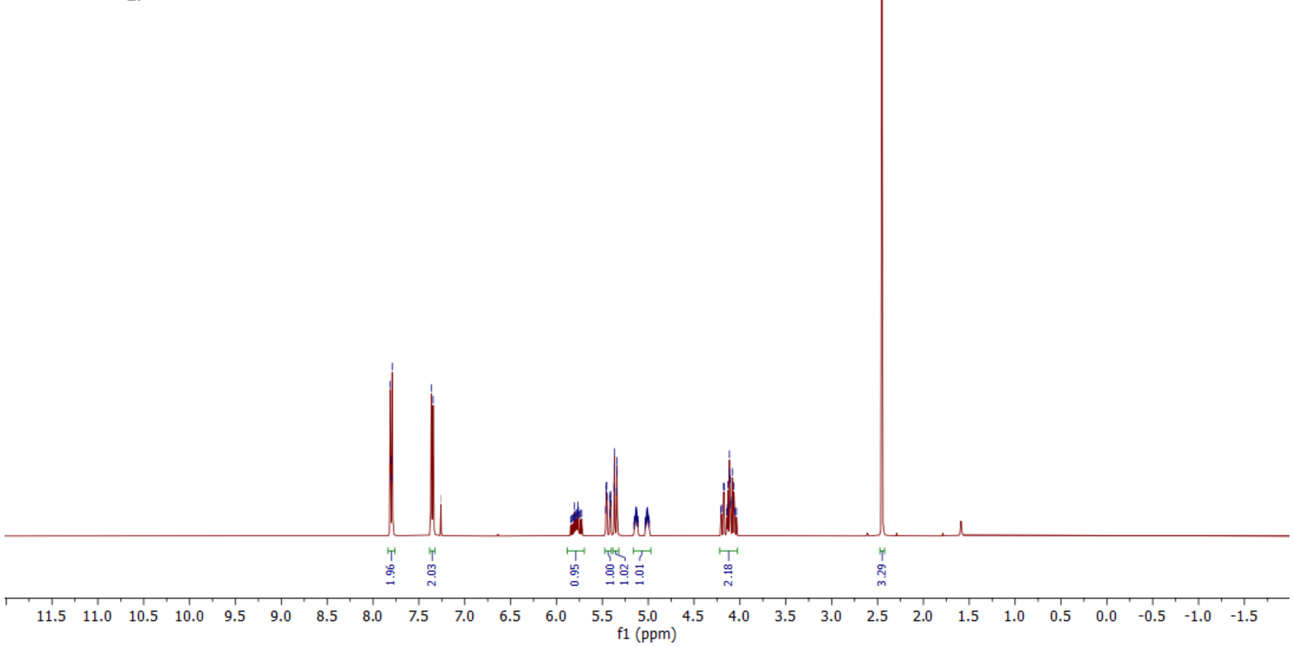

2l ^{13}C NMR (101 MHz, $CDCl_3$)

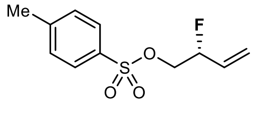

2l

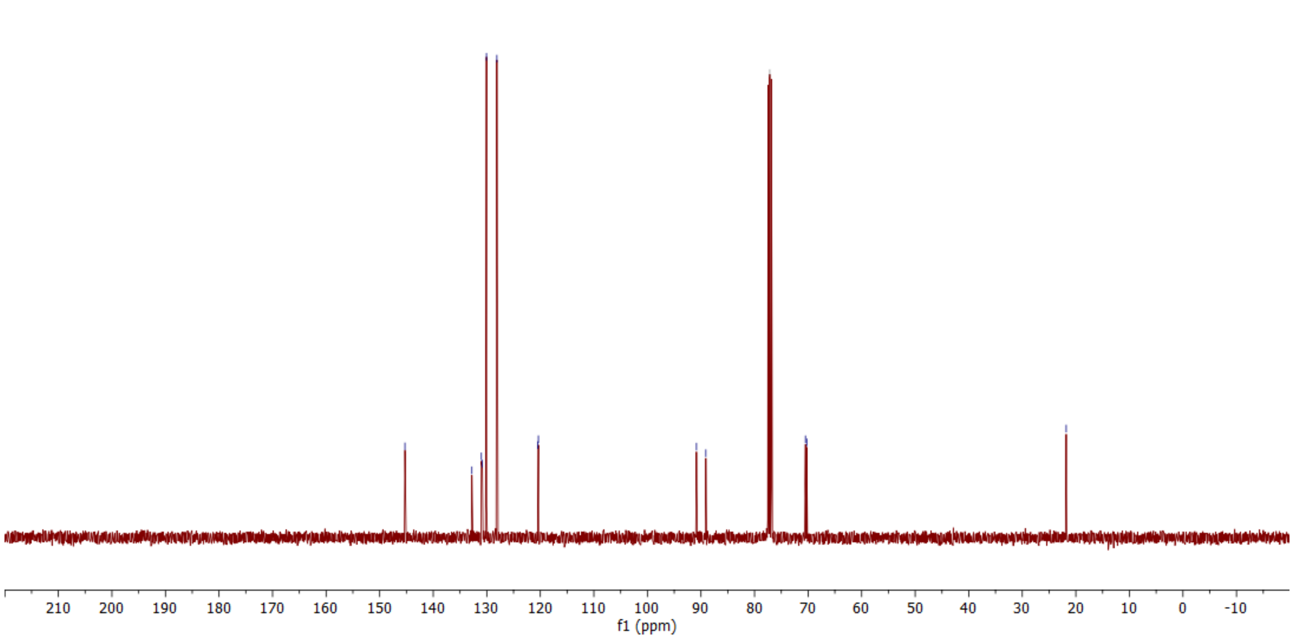

2l ^{19}F NMR (377 MHz, $CDCl_3$)

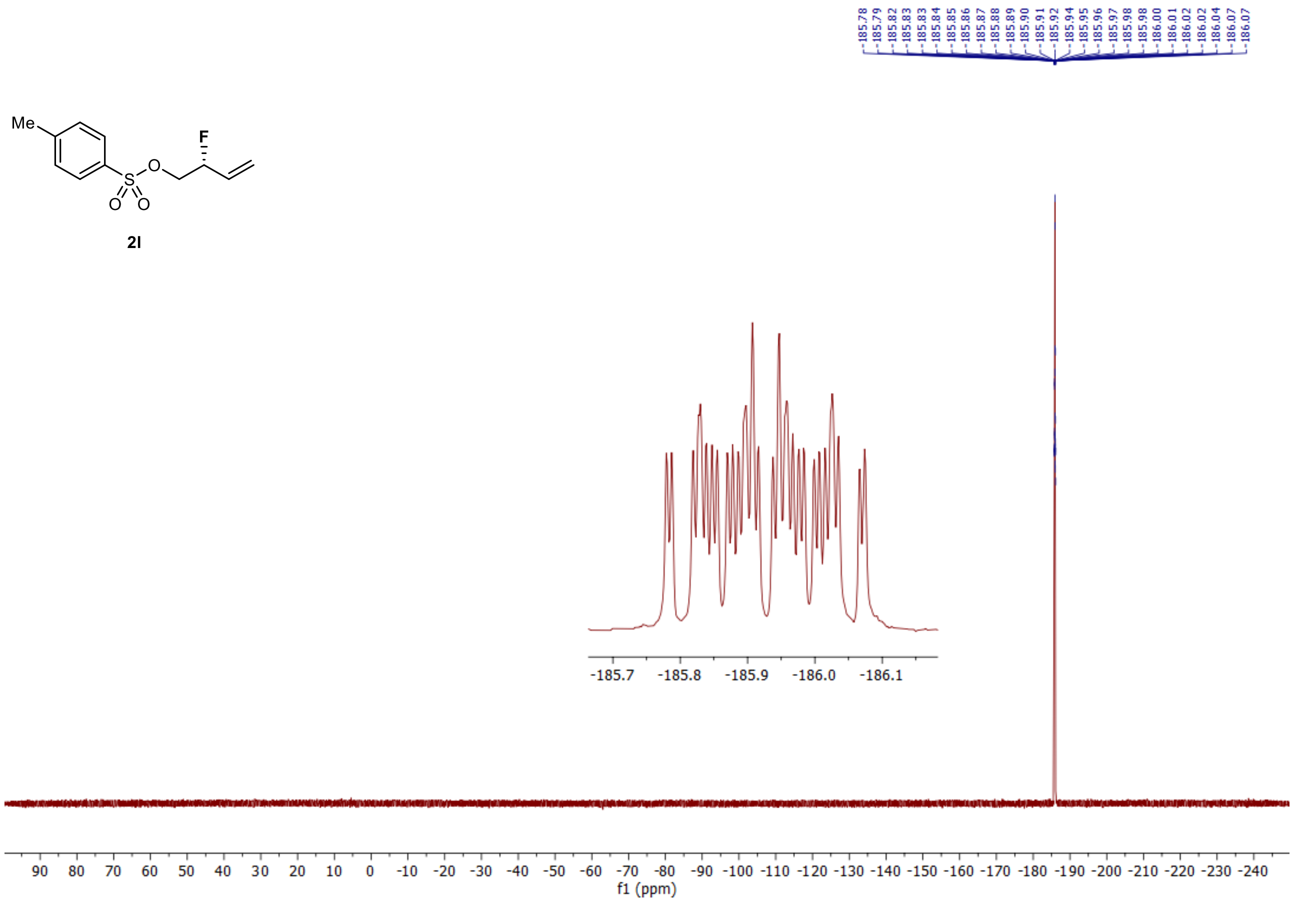

2m ^{1}H NMR (400 MHz, $CDCl_3$)

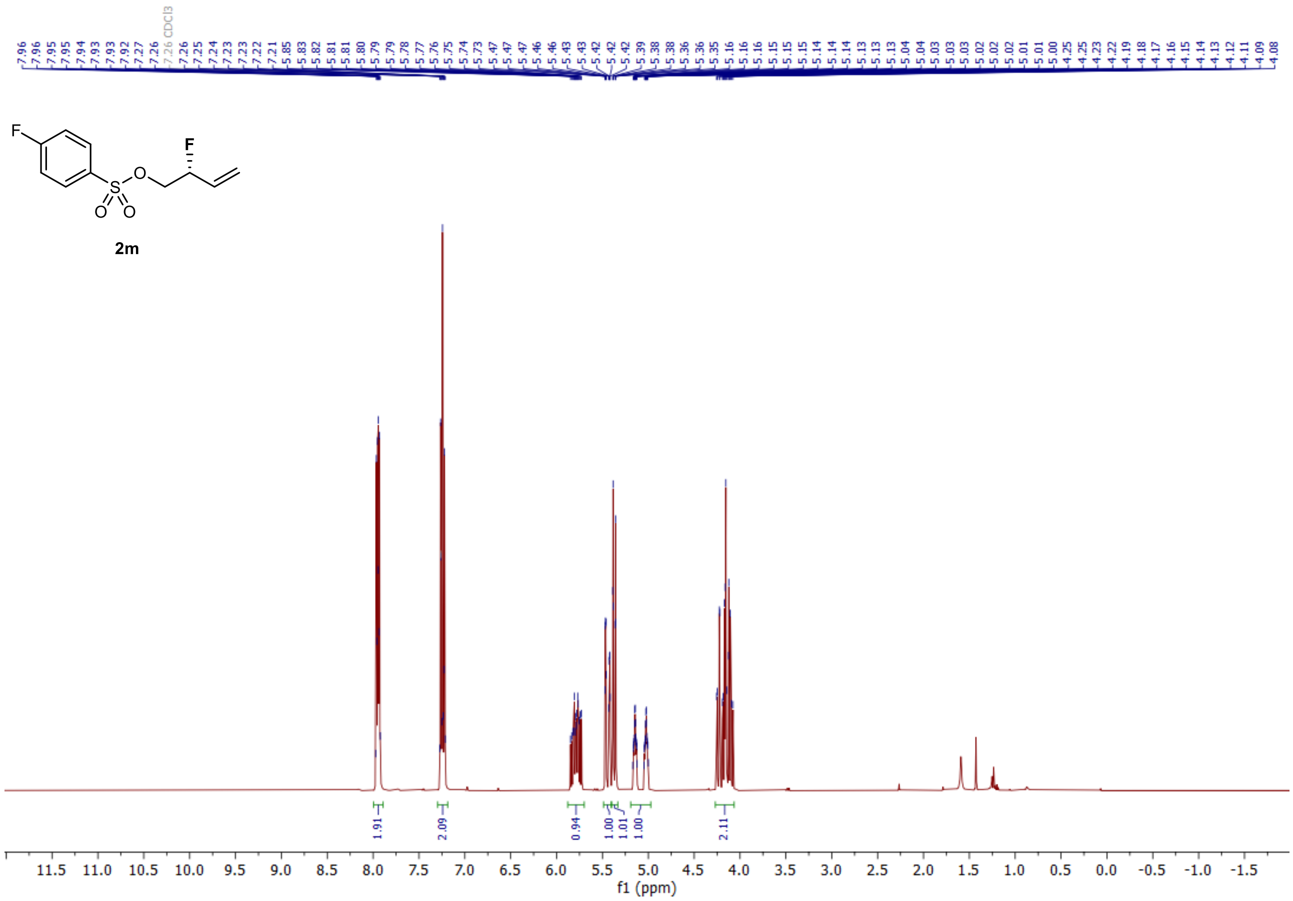

2m ^{13}C NMR (101 MHz, $CDCl_3$)

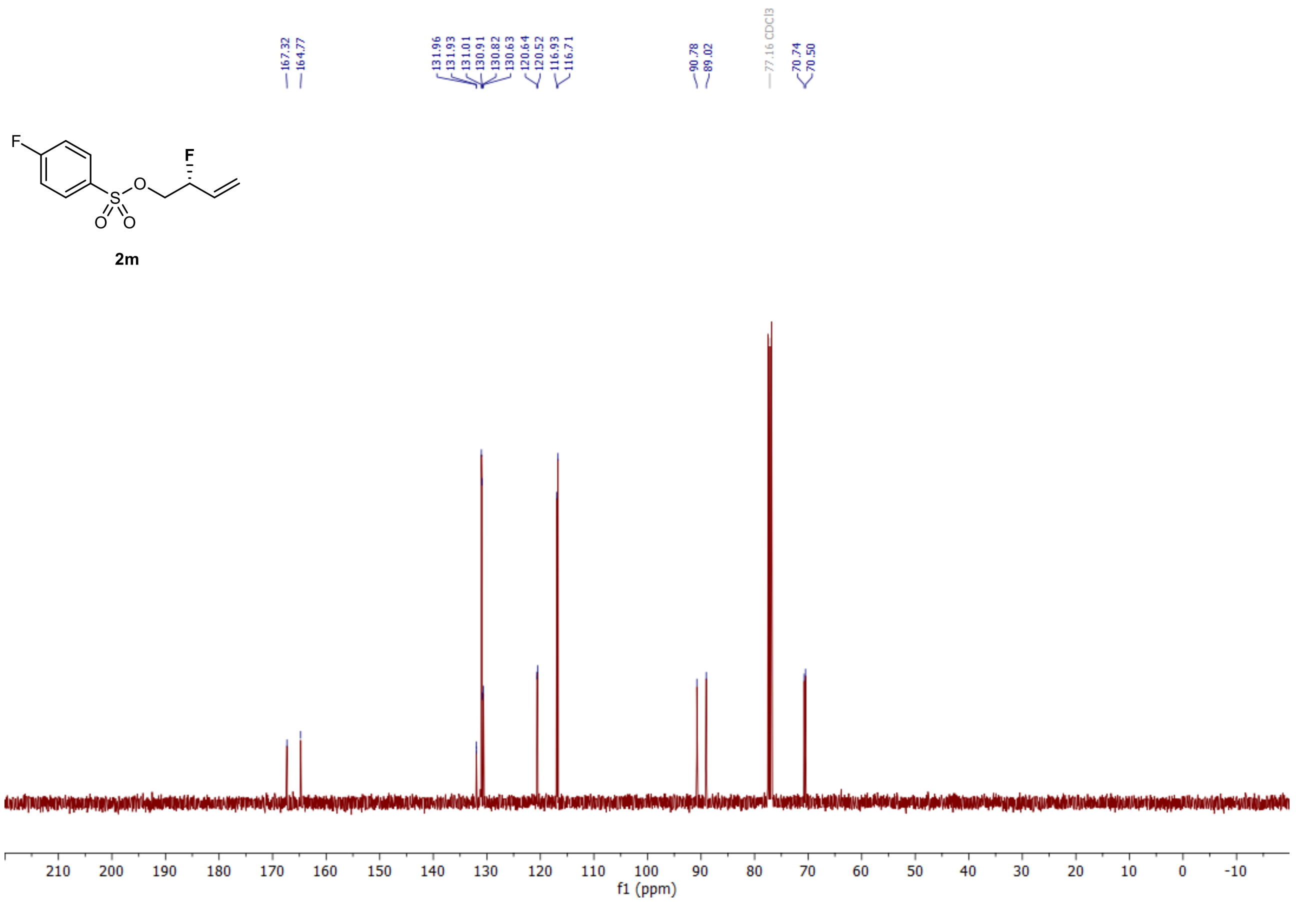

2m ^{19}F NMR (377 MHz, $CDCl_3$)

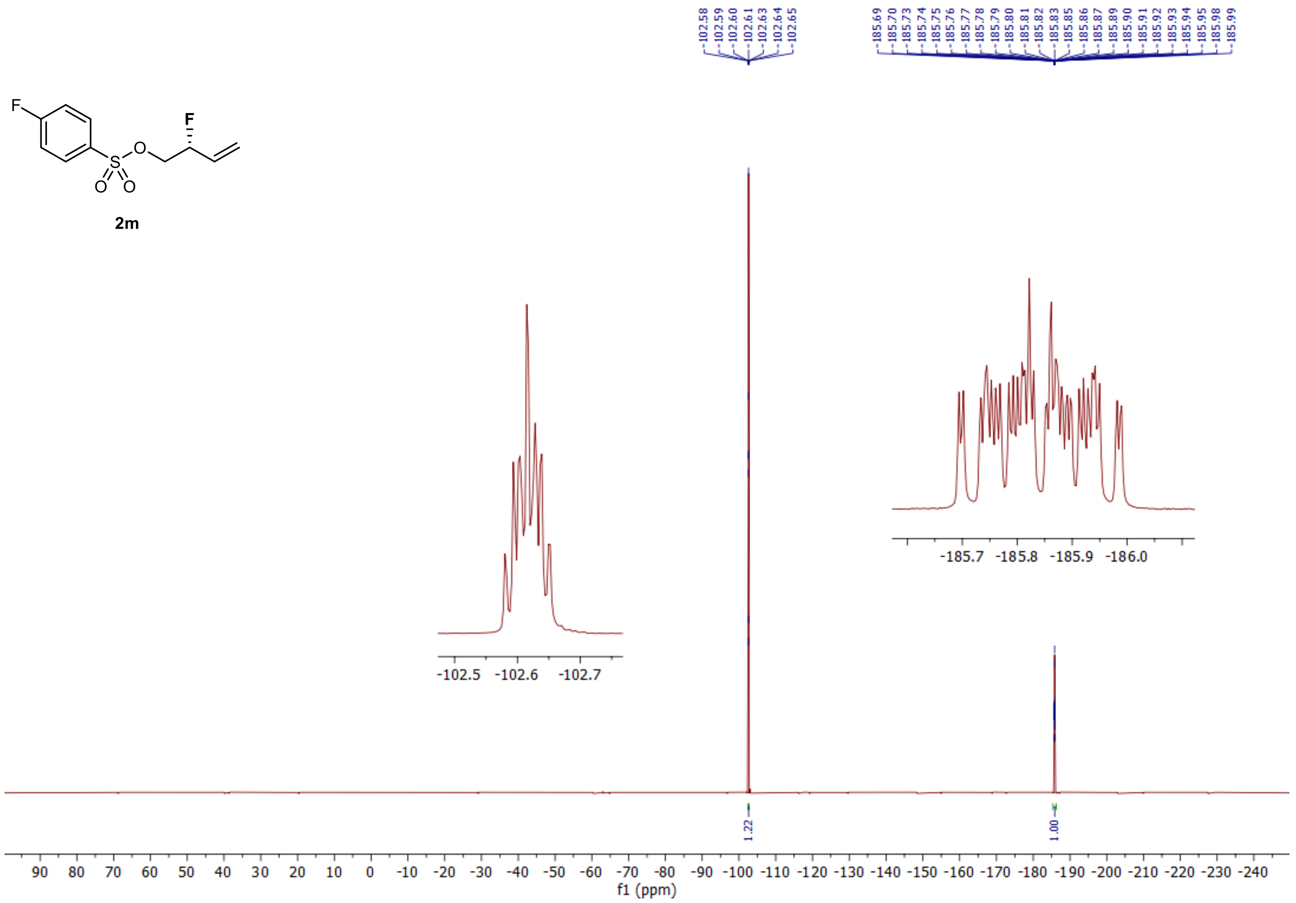

2n ^{1}H NMR (400 MHz, $CDCl_3$)

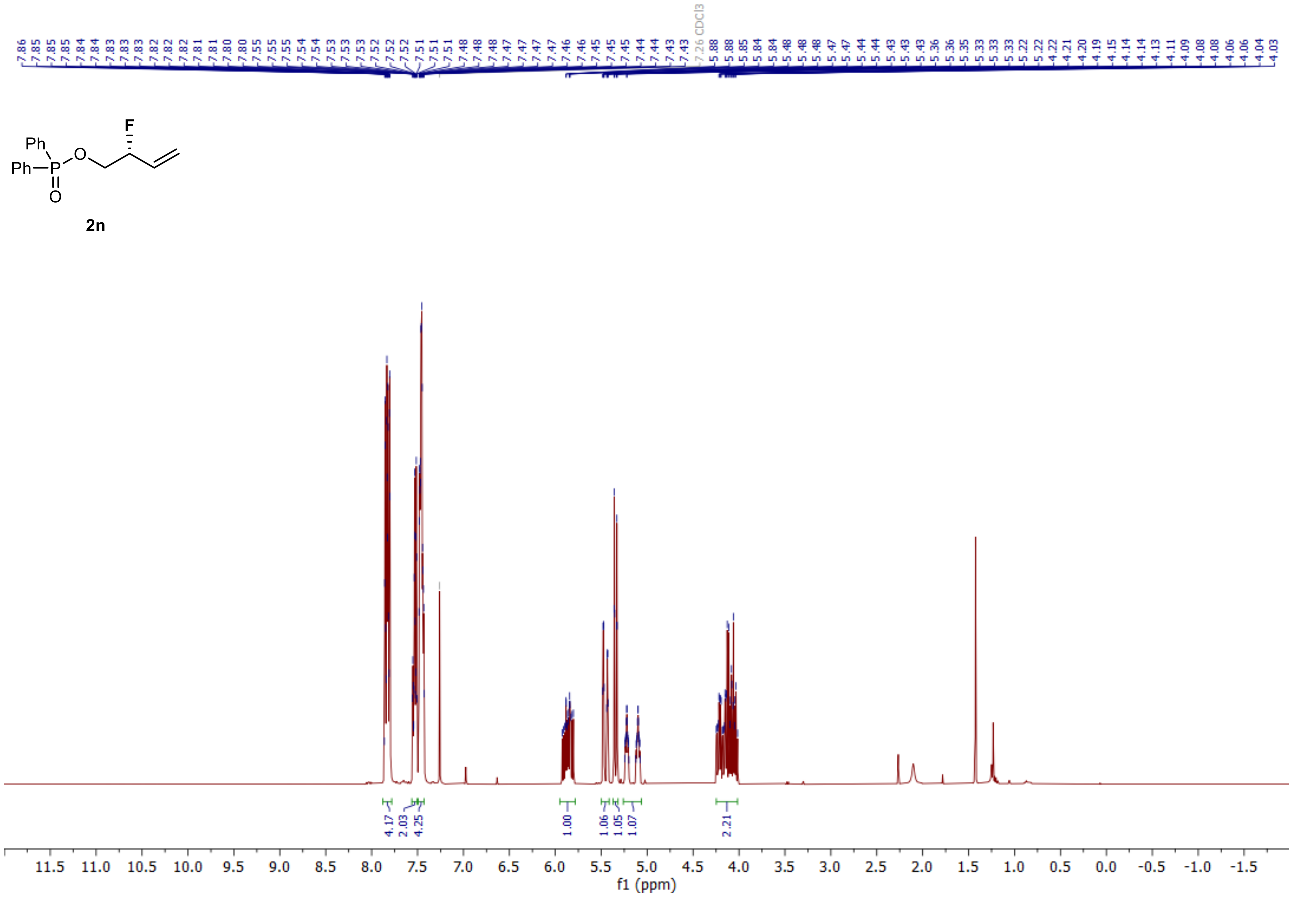

2n ^{13}C NMR (101 MHz, $CDCl_3$)

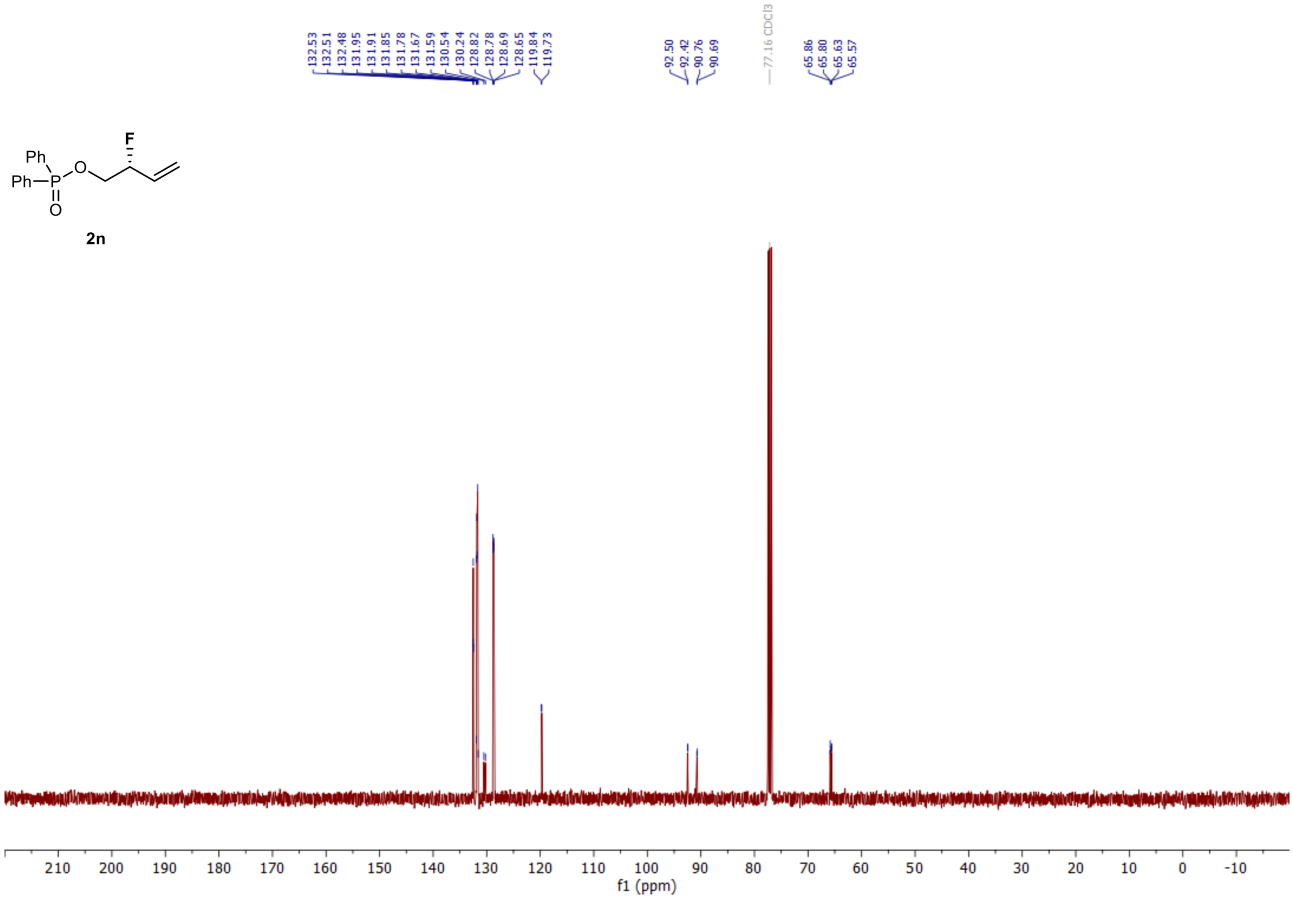

2n ^{19}F NMR (377 MHz, $CDCl_3$)

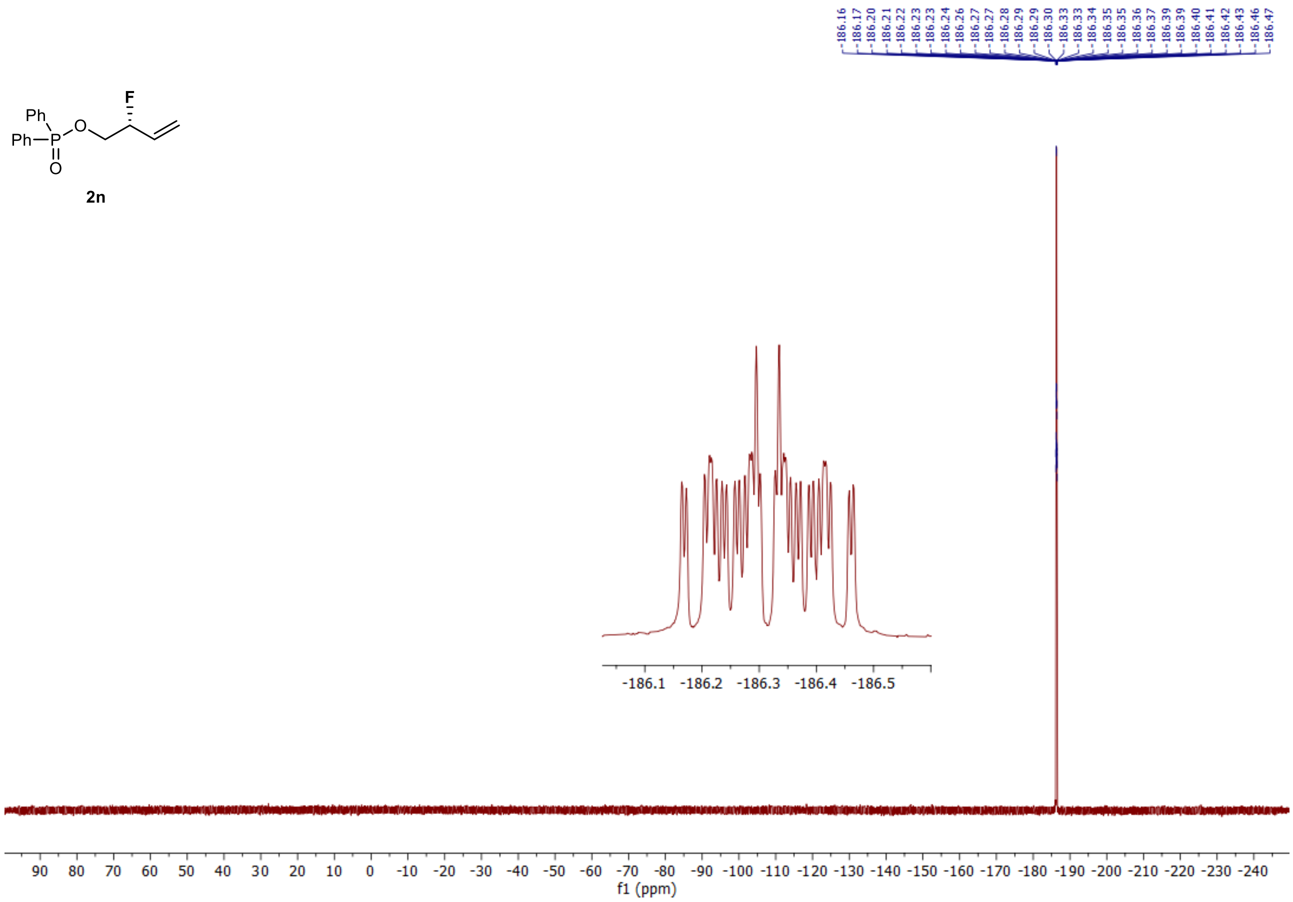

2n ^{31}P NMR (162 MHz, $CDCl_3$)

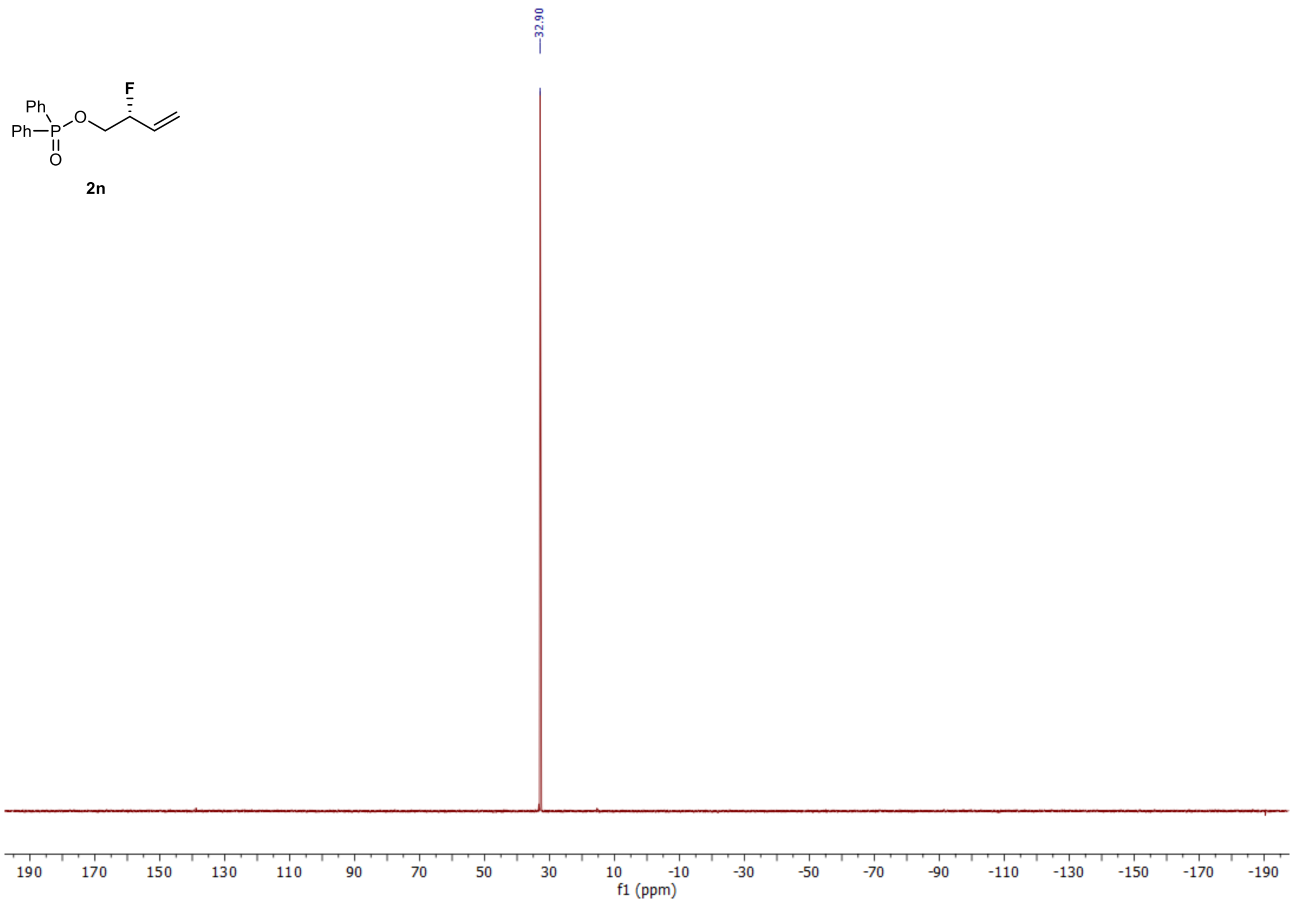

2o ^{1}H NMR (400 MHz, $CDCl_3$)

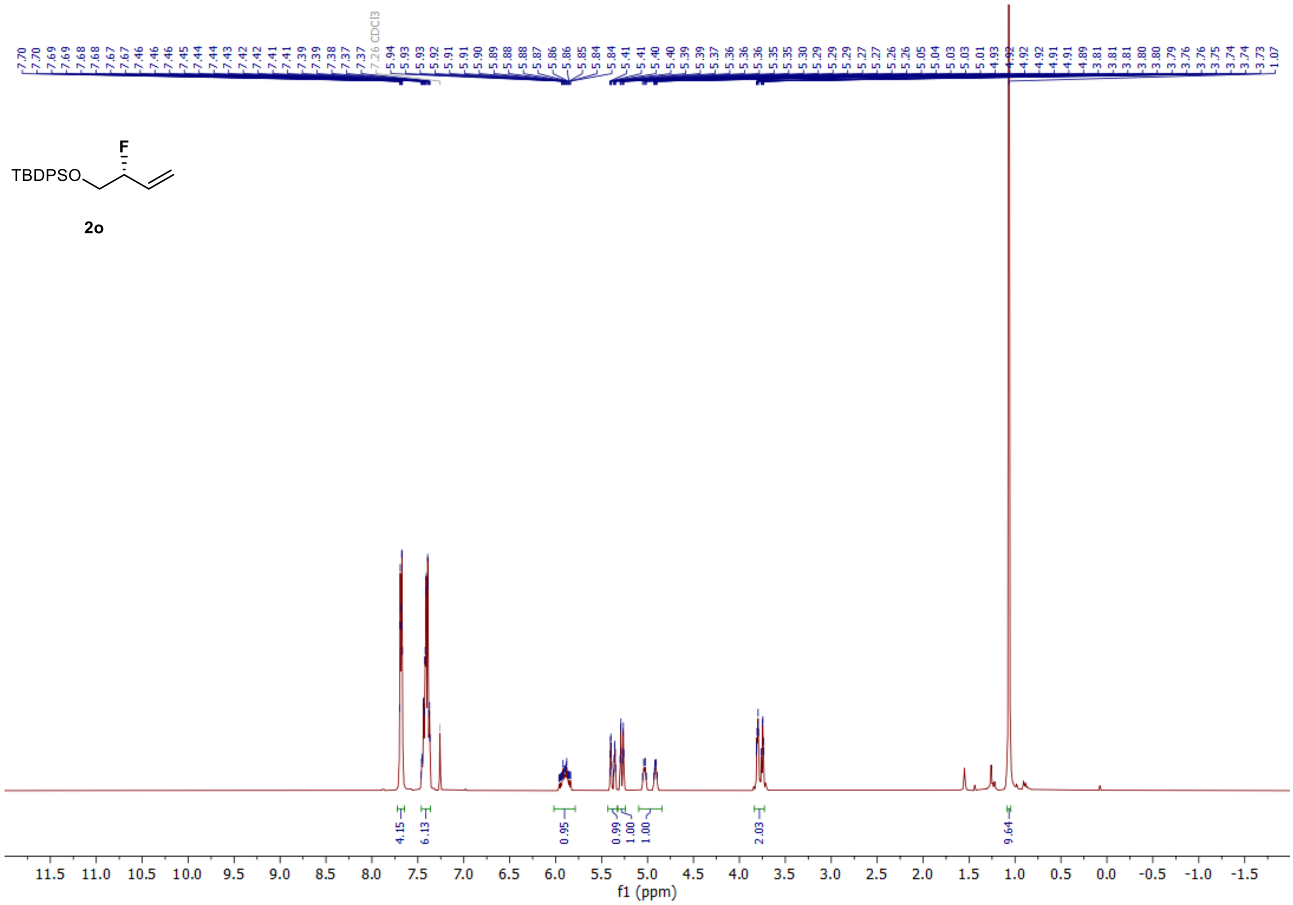

2o ^{13}C NMR (101 MHz, $CDCl_3$)

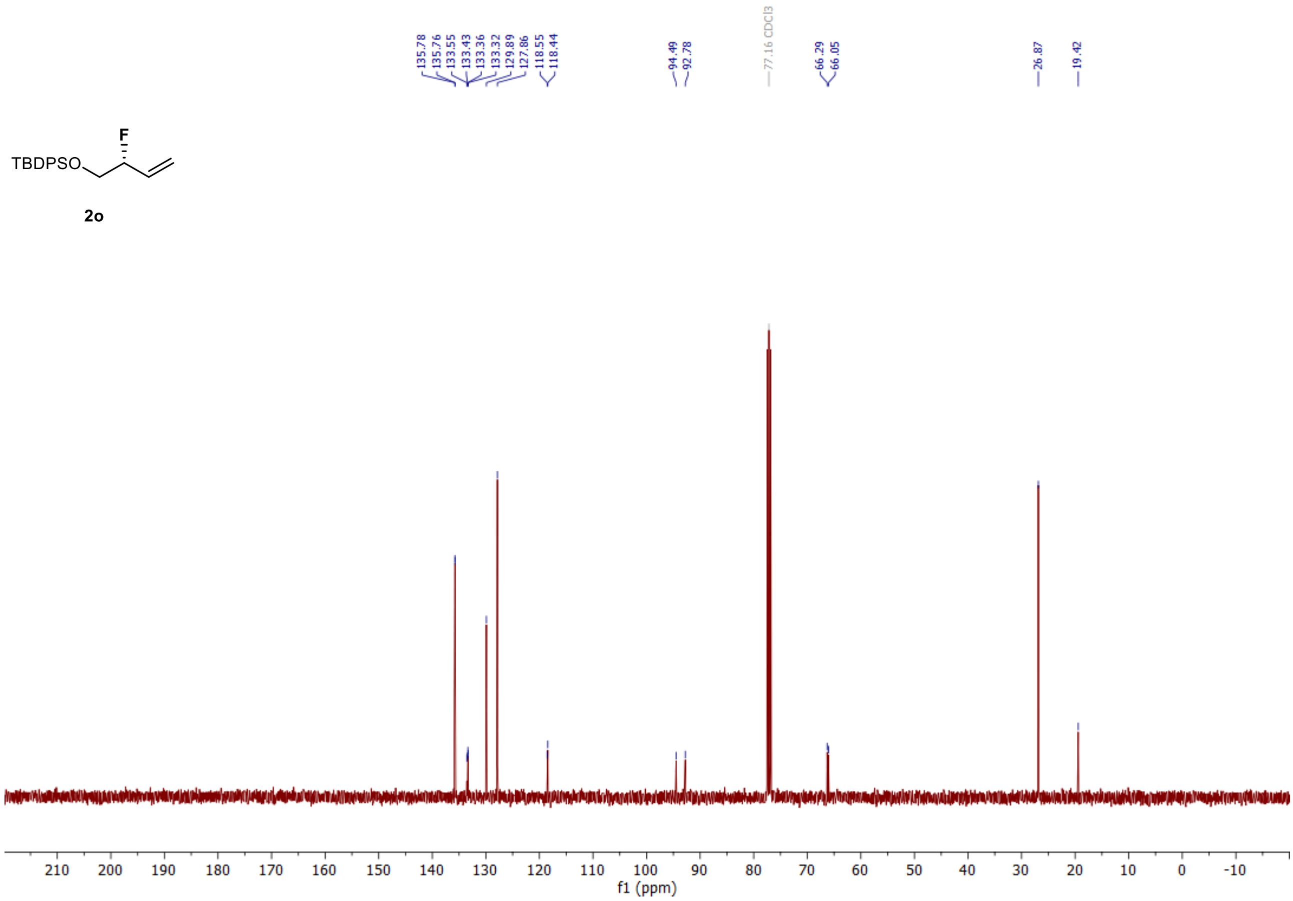

2o ^{19}F NMR (377 MHz, $CDCl_3$)

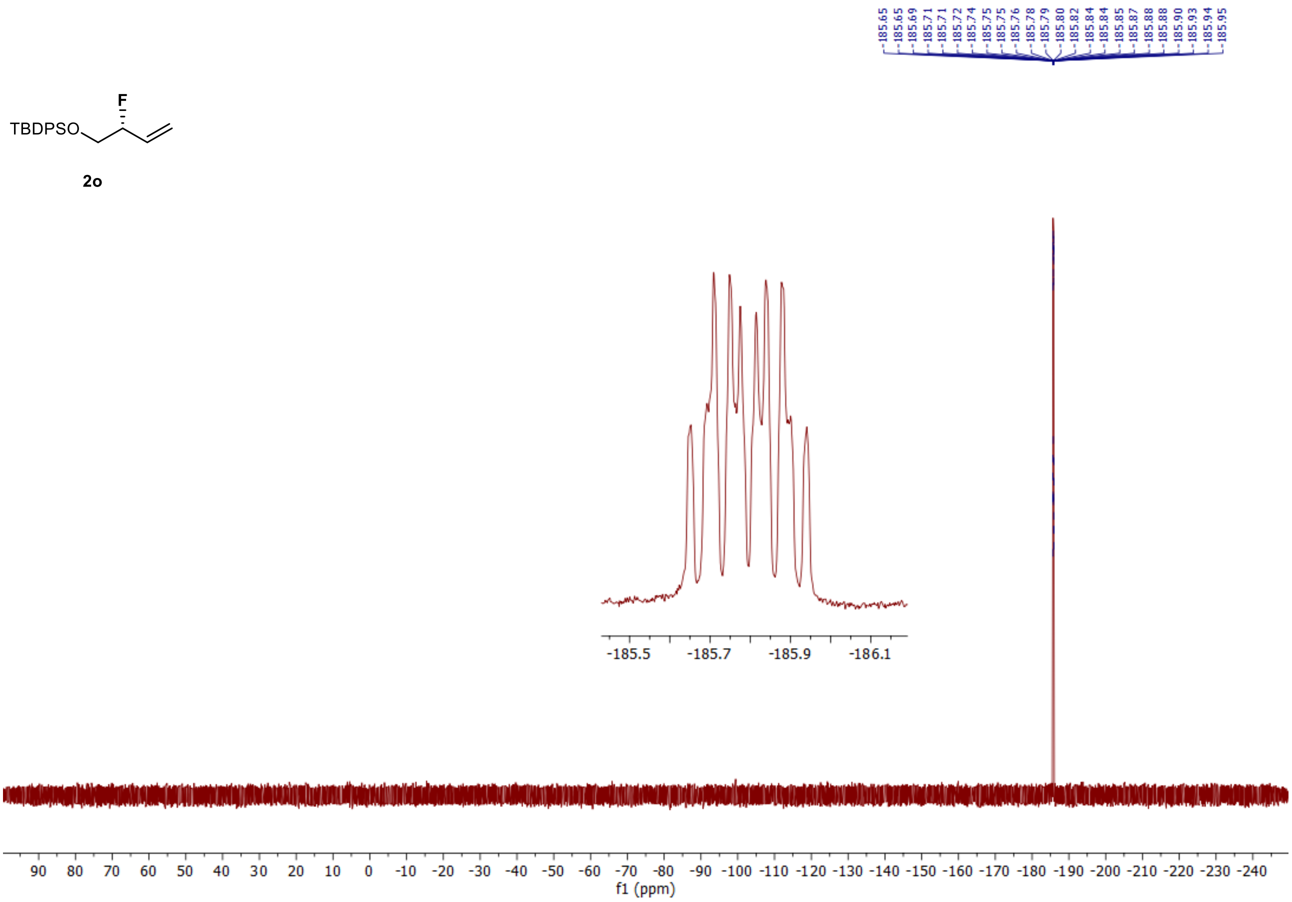

2p ^{1}H NMR (400 MHz, $CDCl_3$)

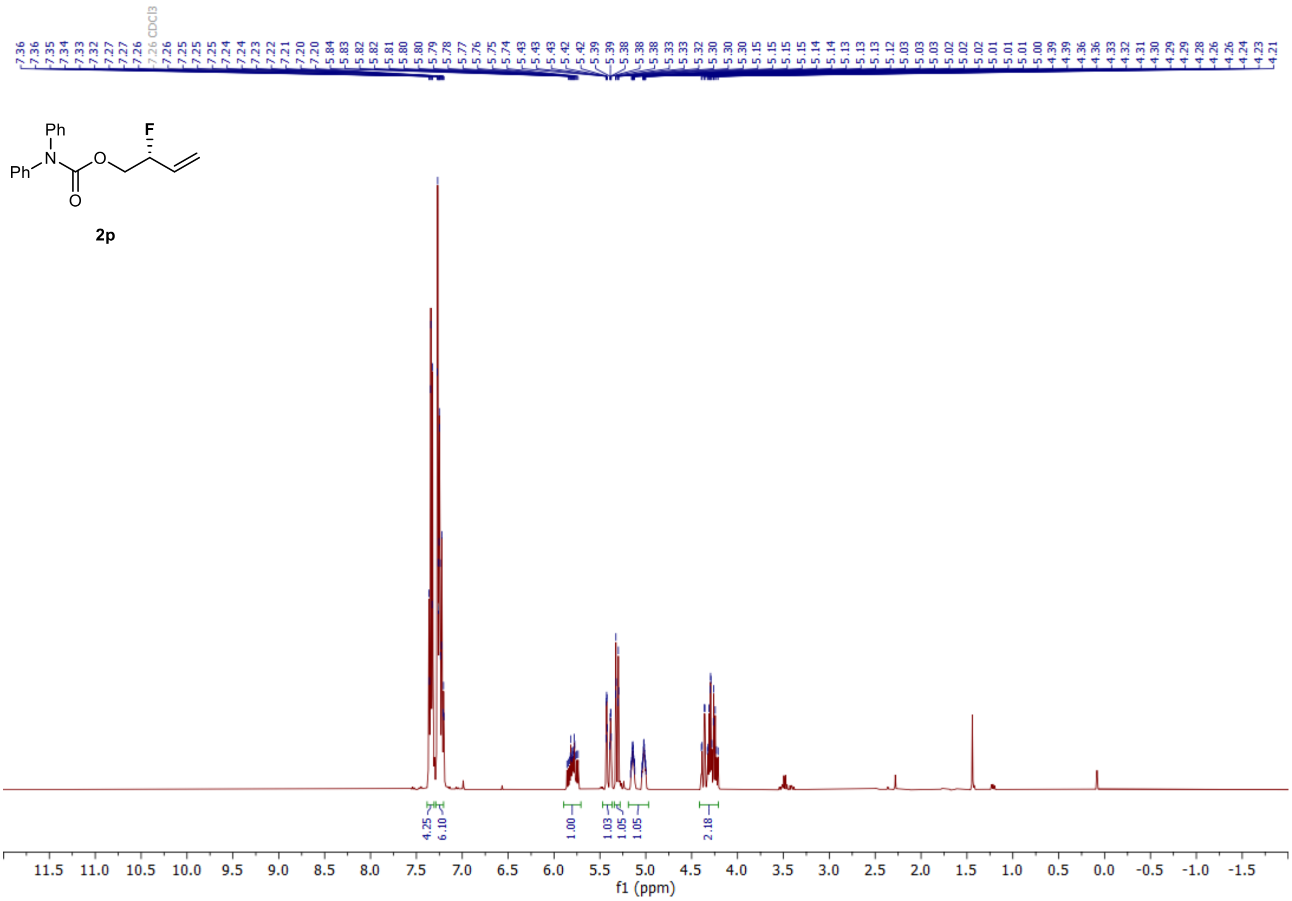

2p ^{13}C NMR (101 MHz, $CDCl_3$)

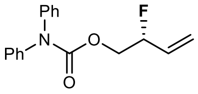

2p

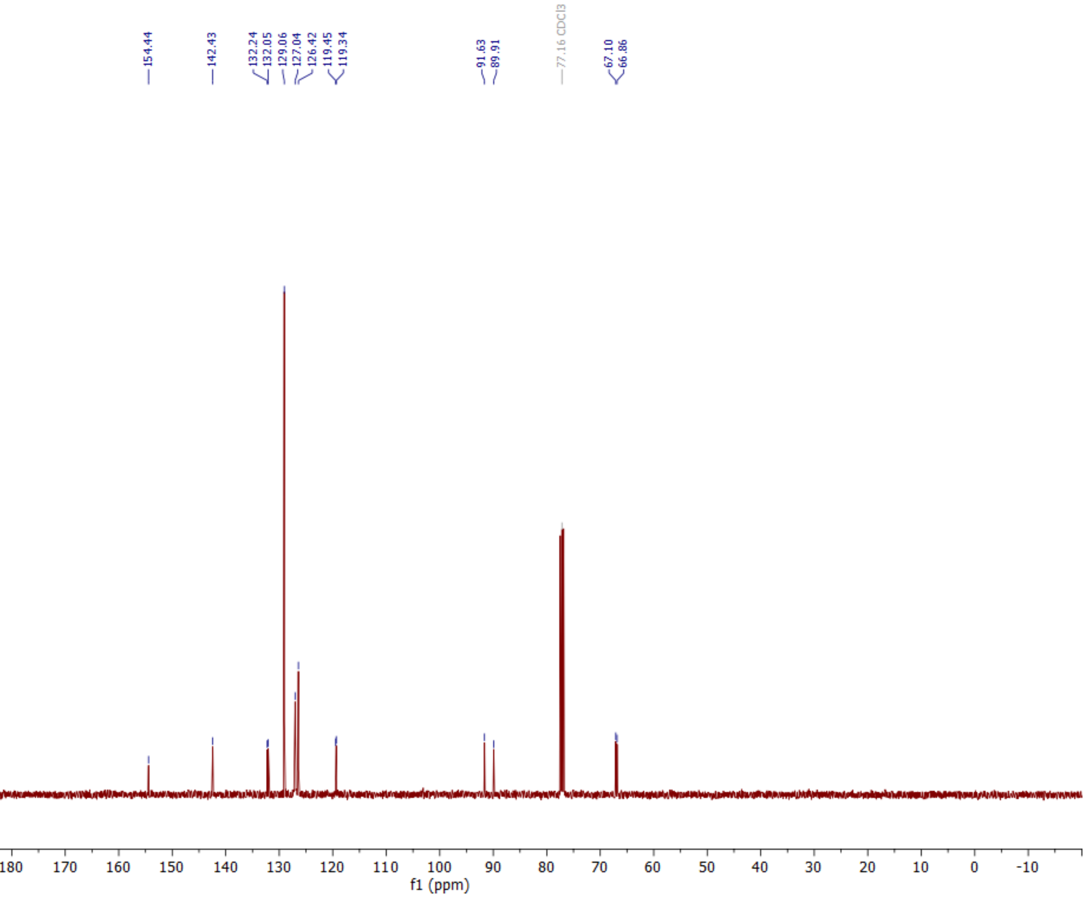

2p ^{19}F NMR (377 MHz, $CDCl_3$)

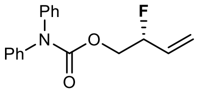

2p

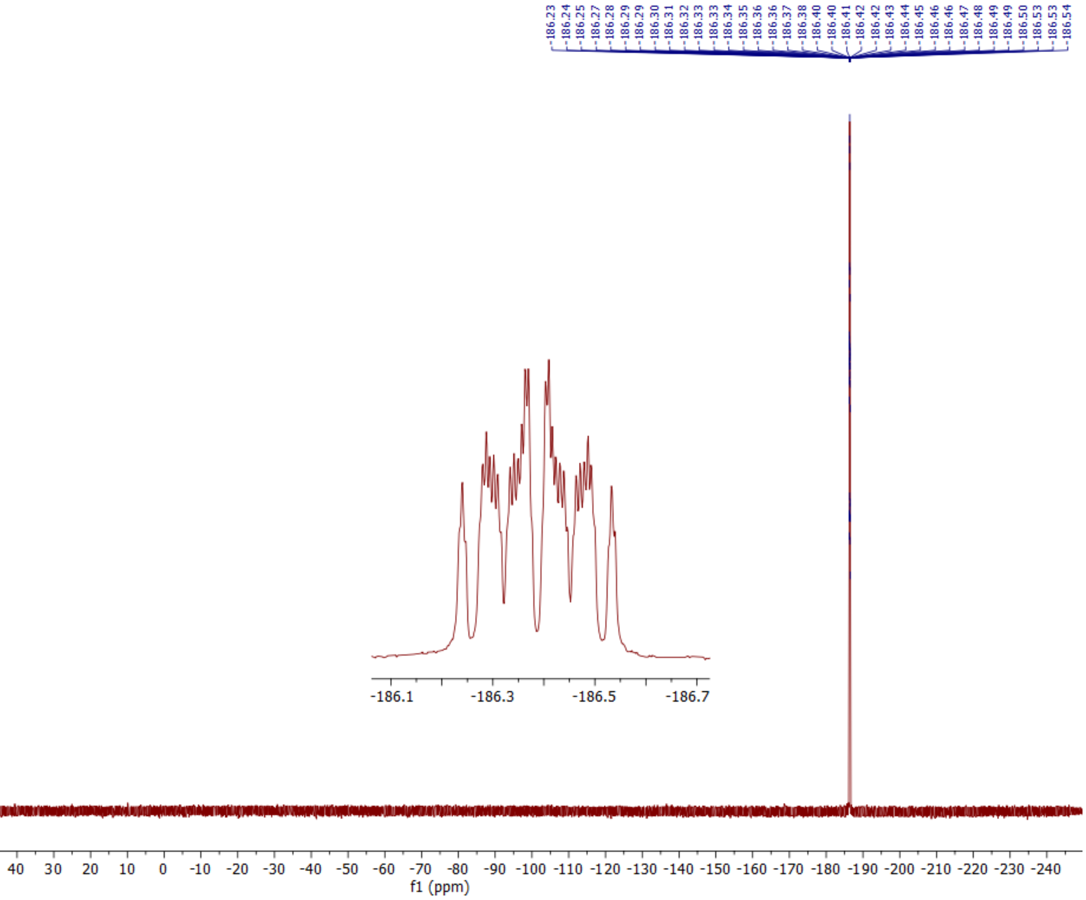

2q ^{1}H NMR (400 MHz, $CDCl_3$)

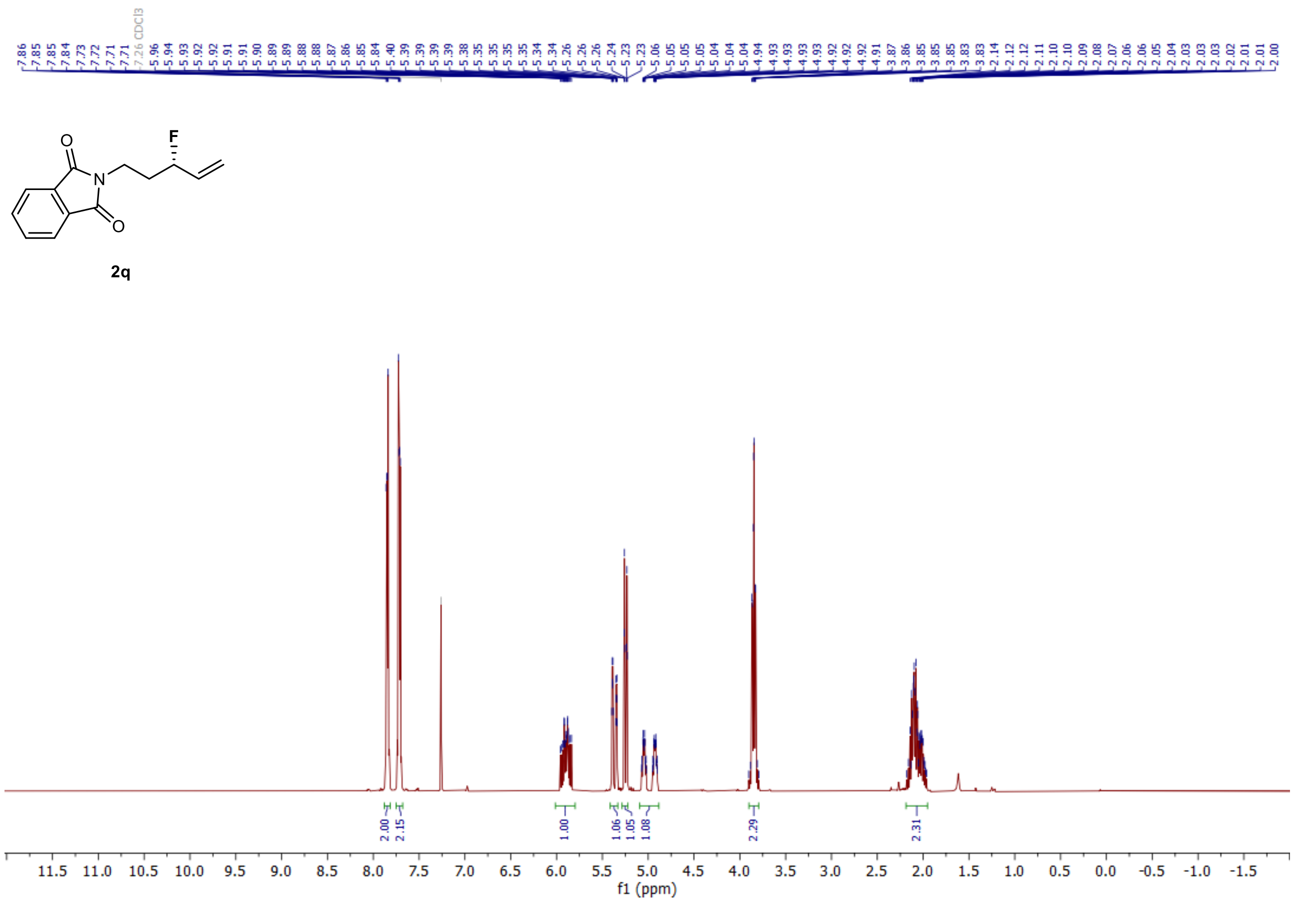

2q ^{13}C NMR (101 MHz, $CDCl_3$)

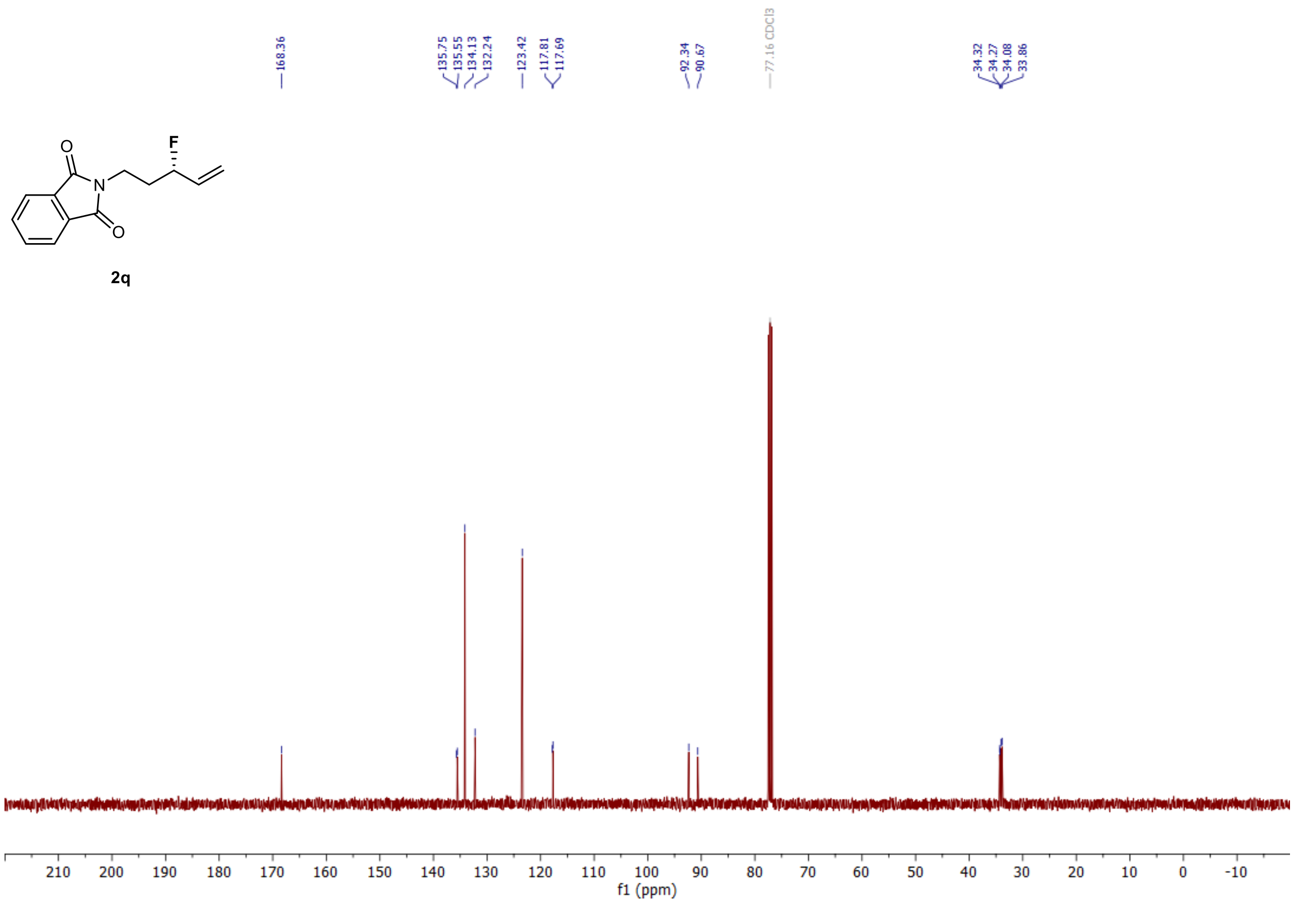

2q ^{19}F NMR (377 MHz, $CDCl_3$)

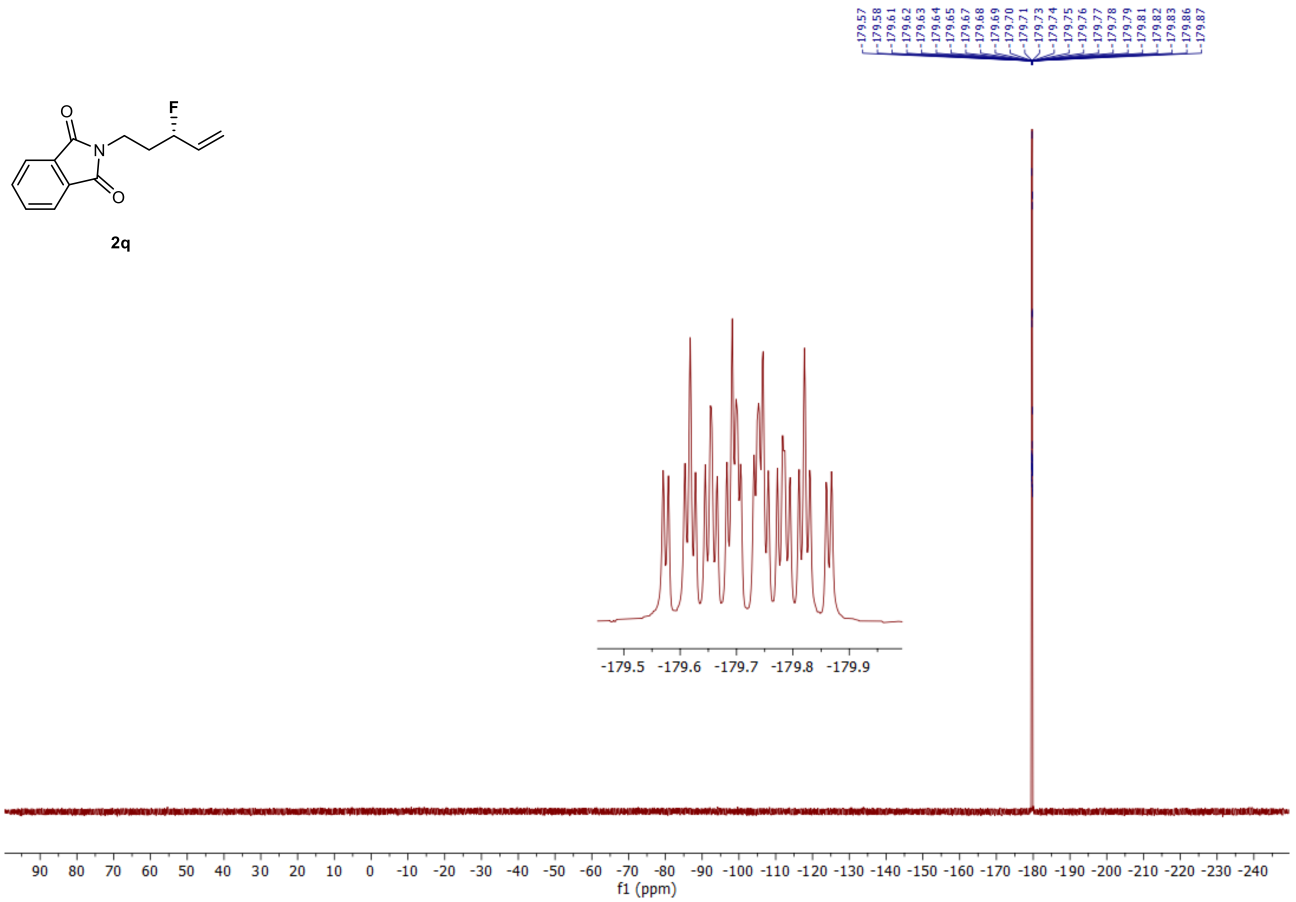

2r ^{1}H NMR (400 MHz, $CDCl_3$)

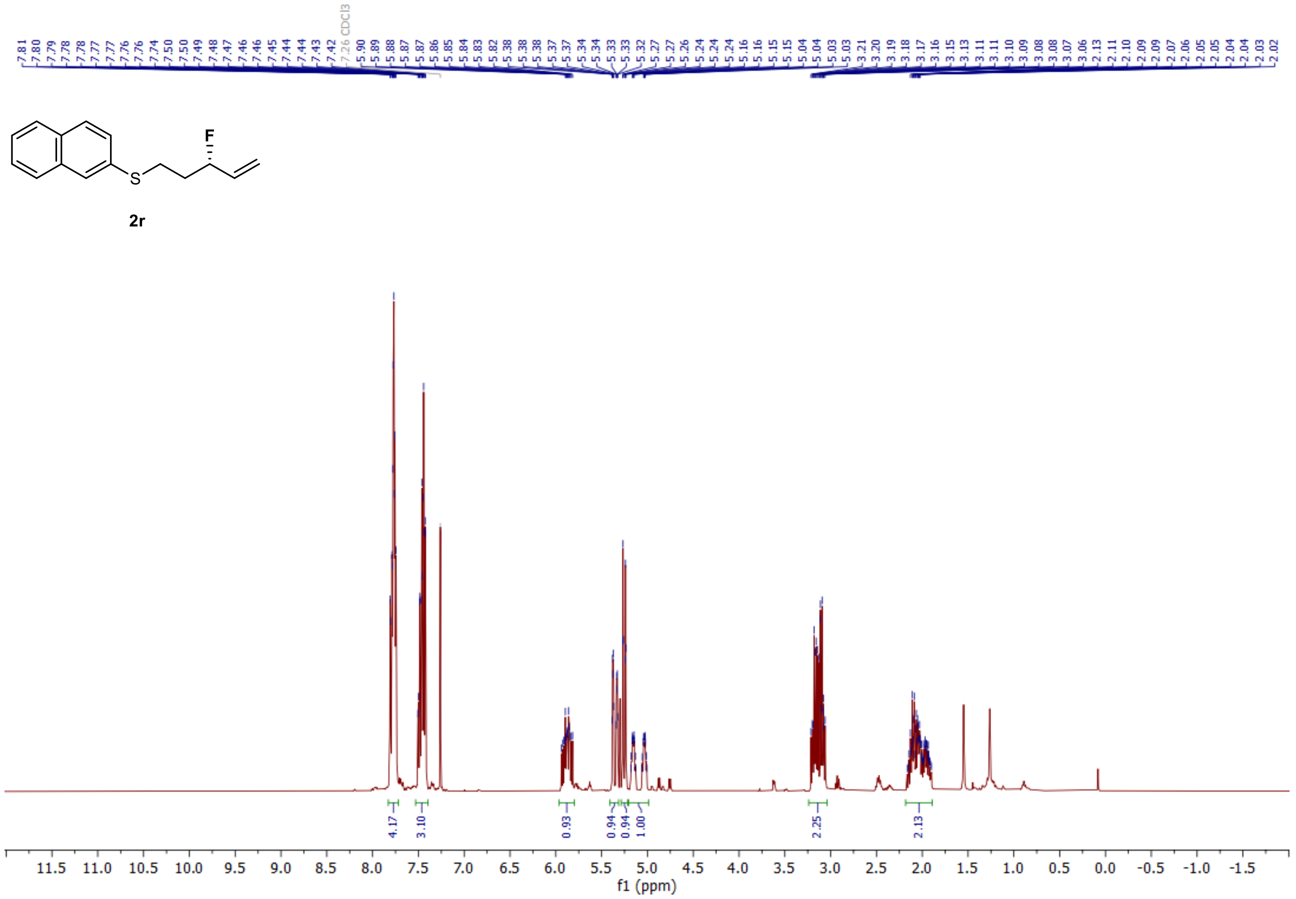

2r ^{13}C NMR (101 MHz, $CDCl_3$)

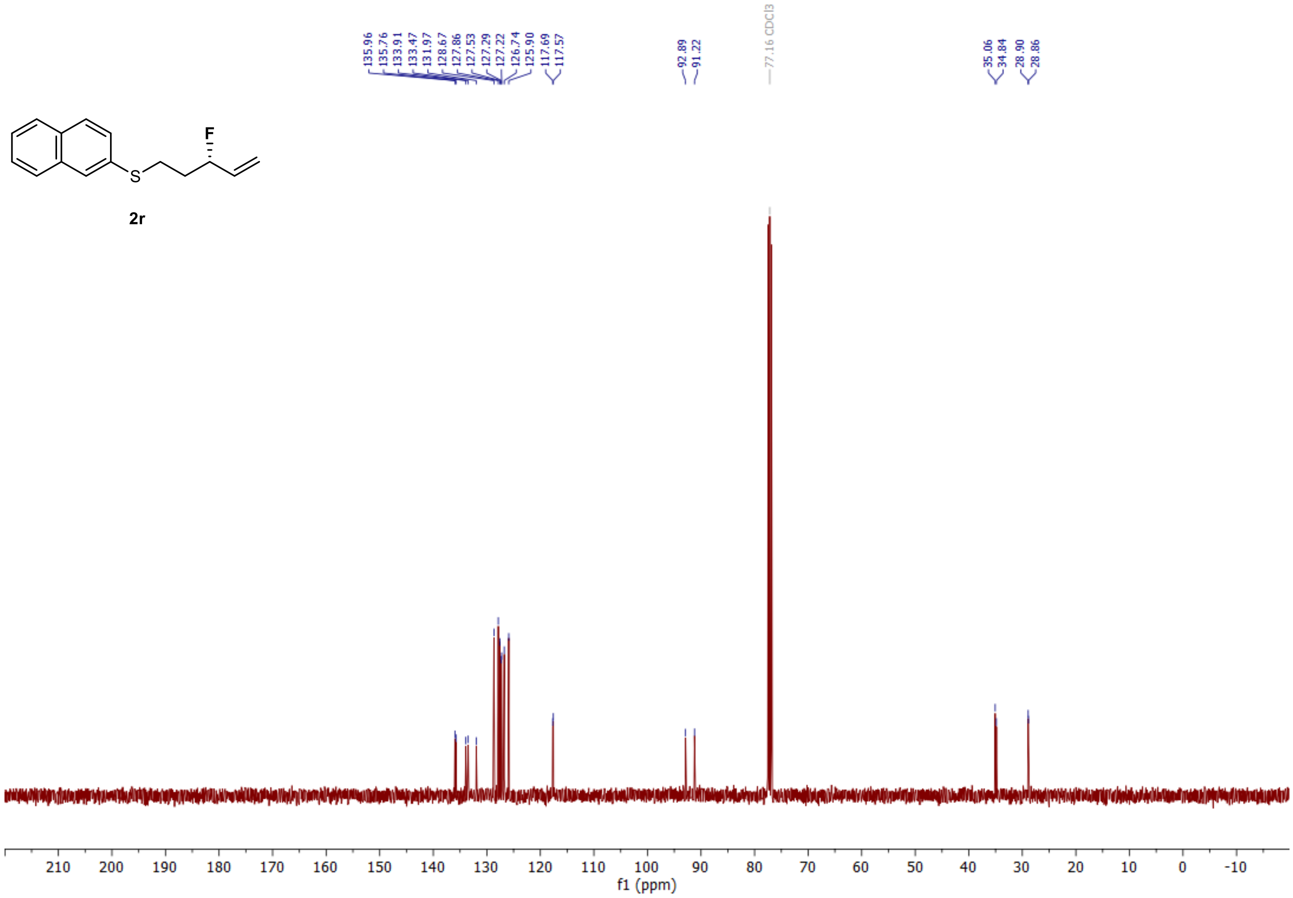

2r ^{19}F NMR (377 MHz, $CDCl_3$)

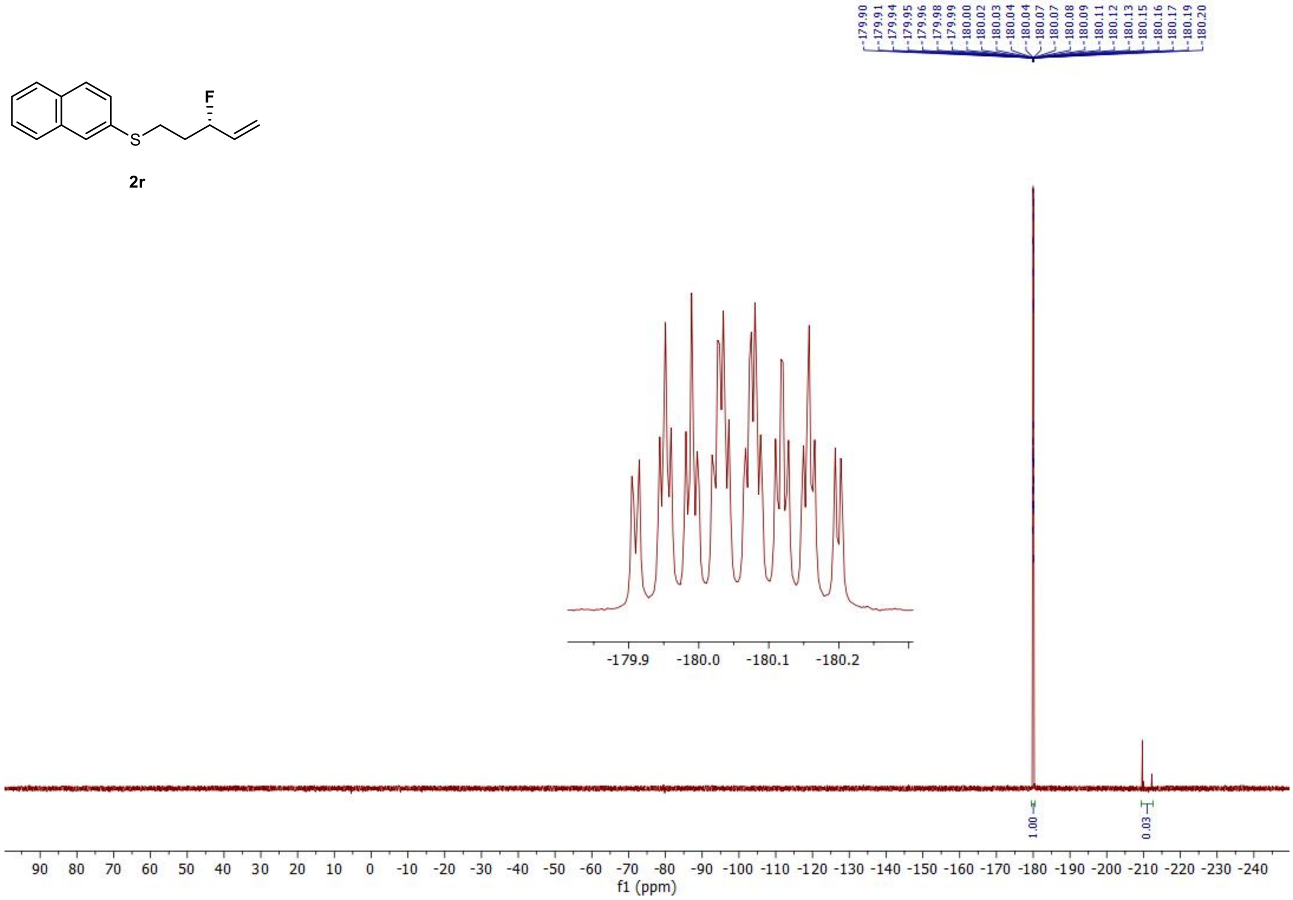

2s ^{1}H NMR (400 MHz, $CDCl_3$)

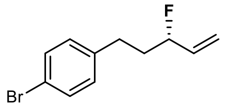

2s

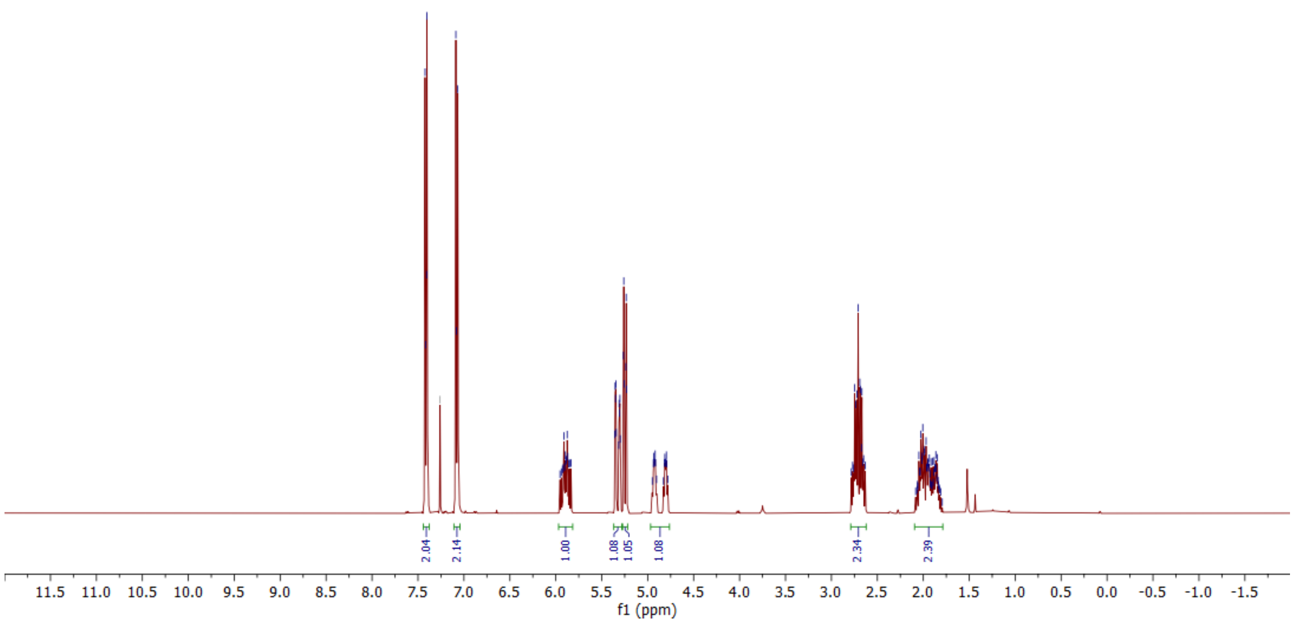

2s ^{13}C NMR (101 MHz, $CDCl_3$)

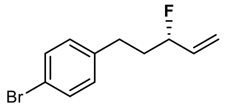

2s

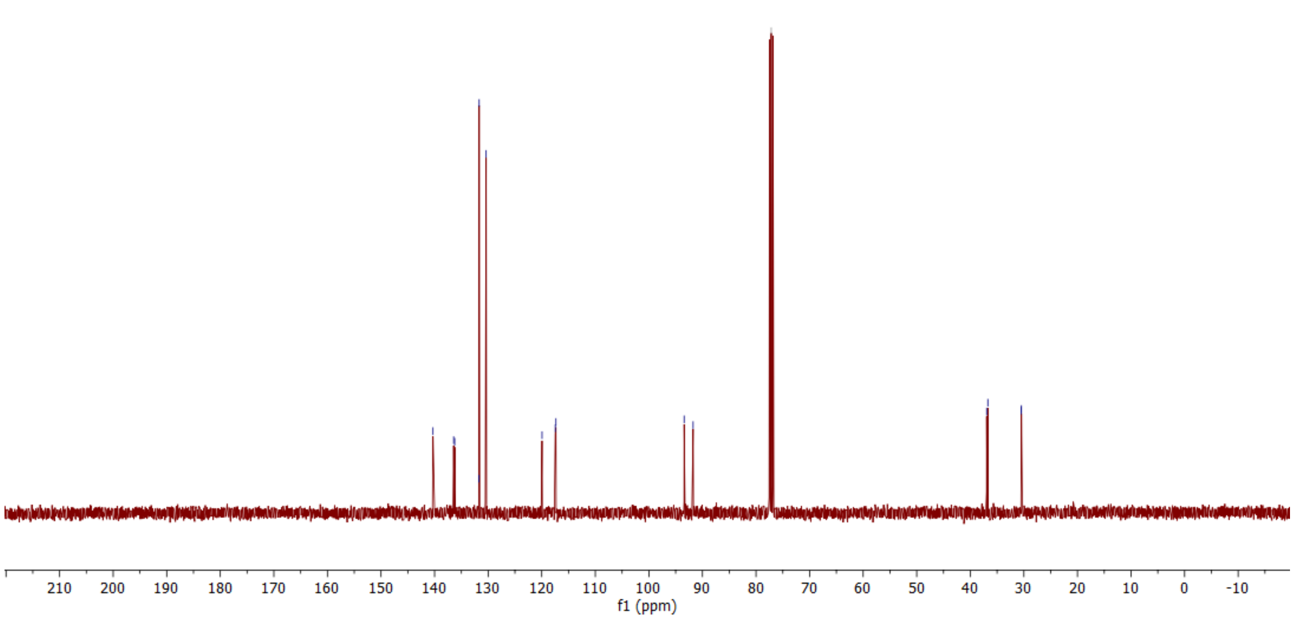

2s ^{19}F NMR (377 MHz, $CDCl_3$)

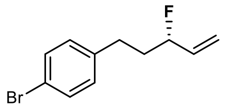

2s

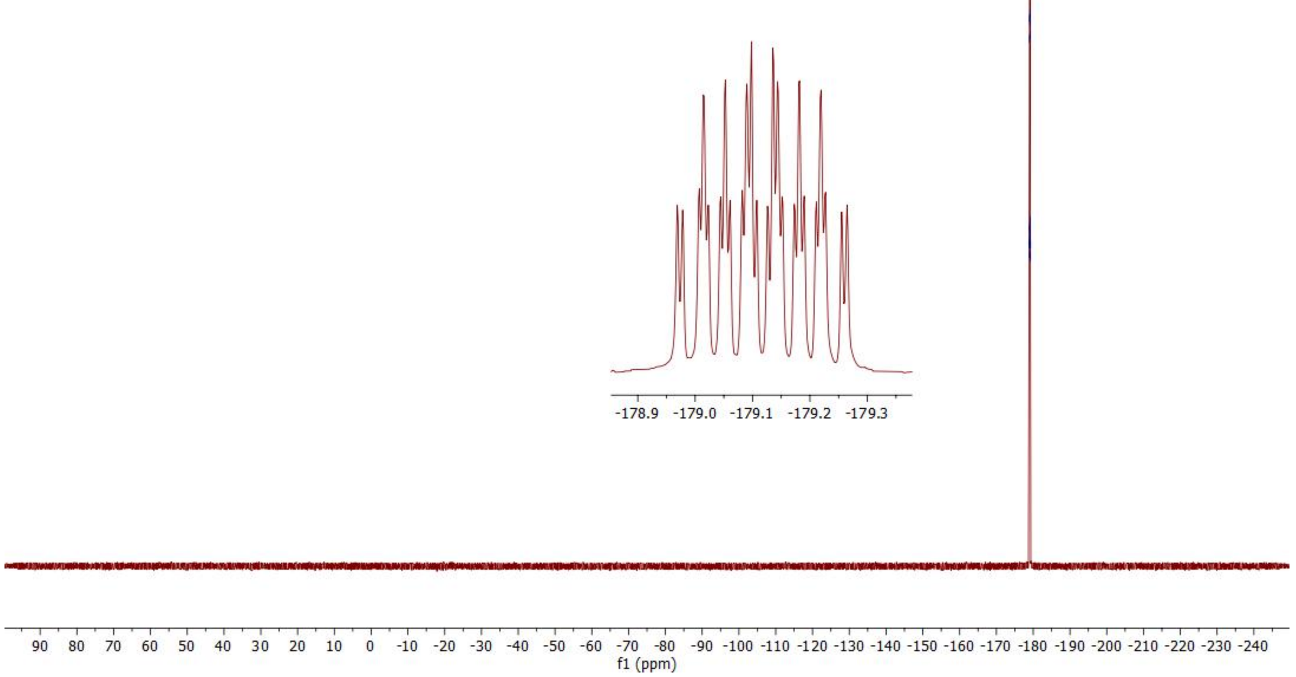

2t ^{1}H NMR (400 MHz, $CDCl_3$)

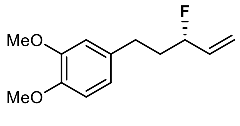

2t

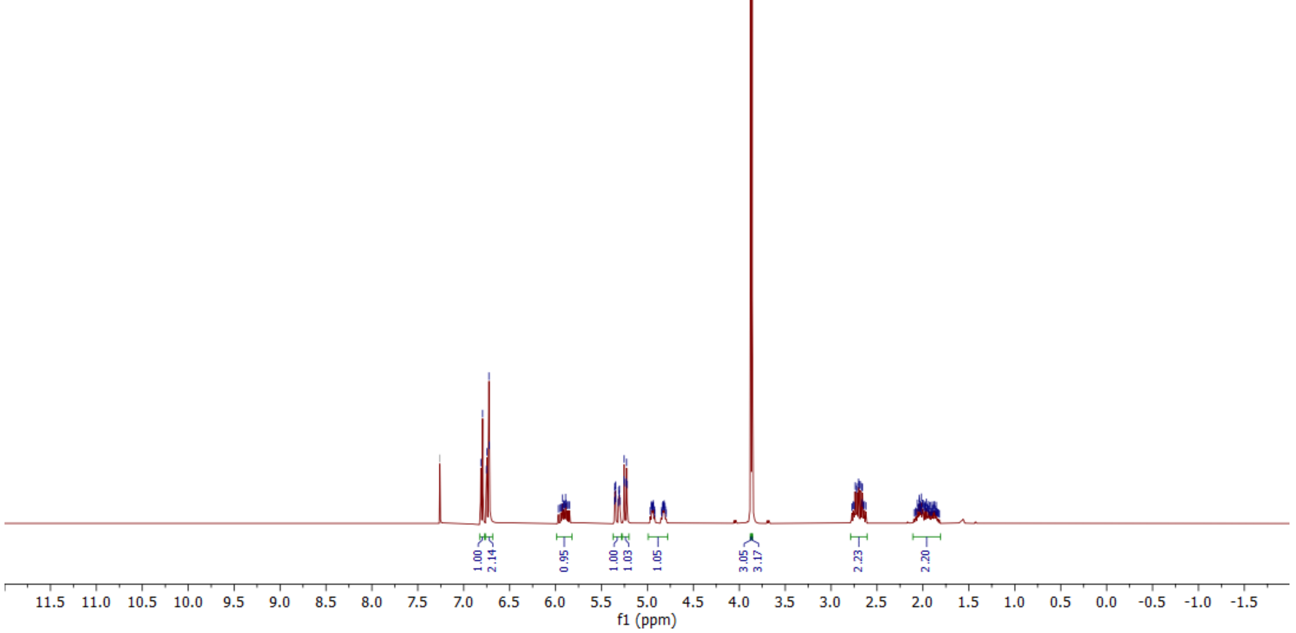

2t ^{13}C NMR (101 MHz, $CDCl_3$)

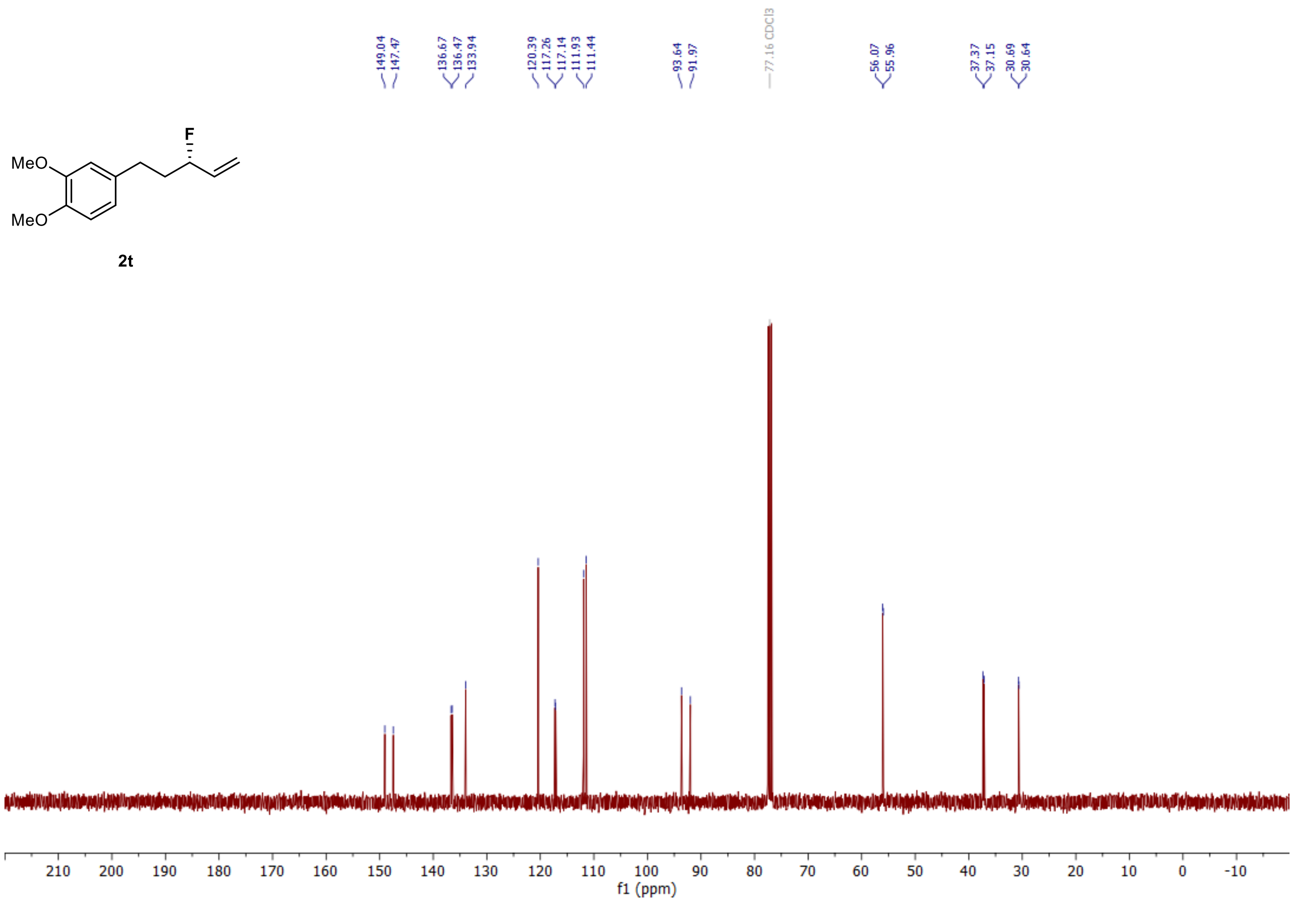

2t ^{19}F NMR (377 MHz, $CDCl_3$)

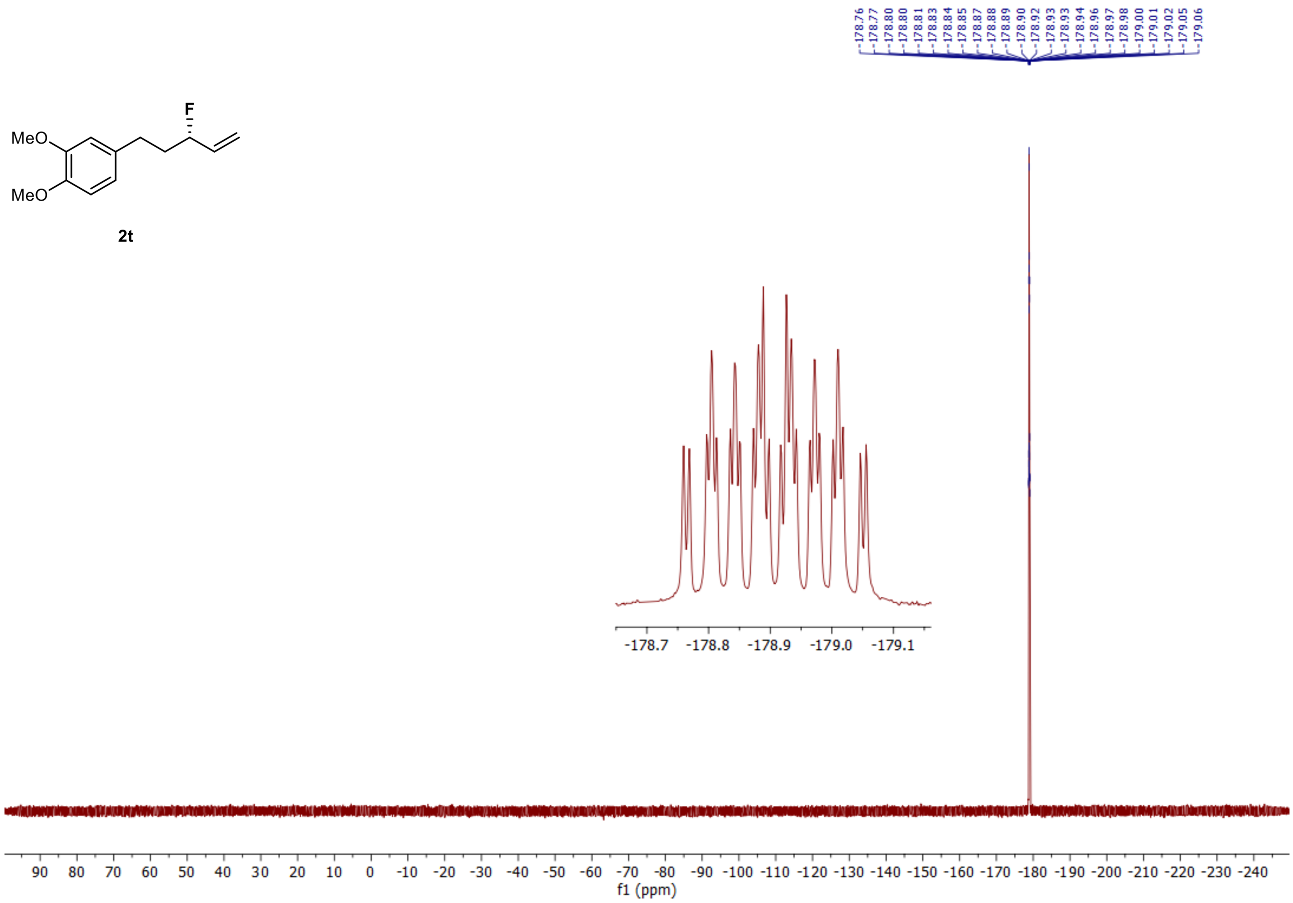

2u ^{1}H NMR (400 MHz, CD_2Cl_2)

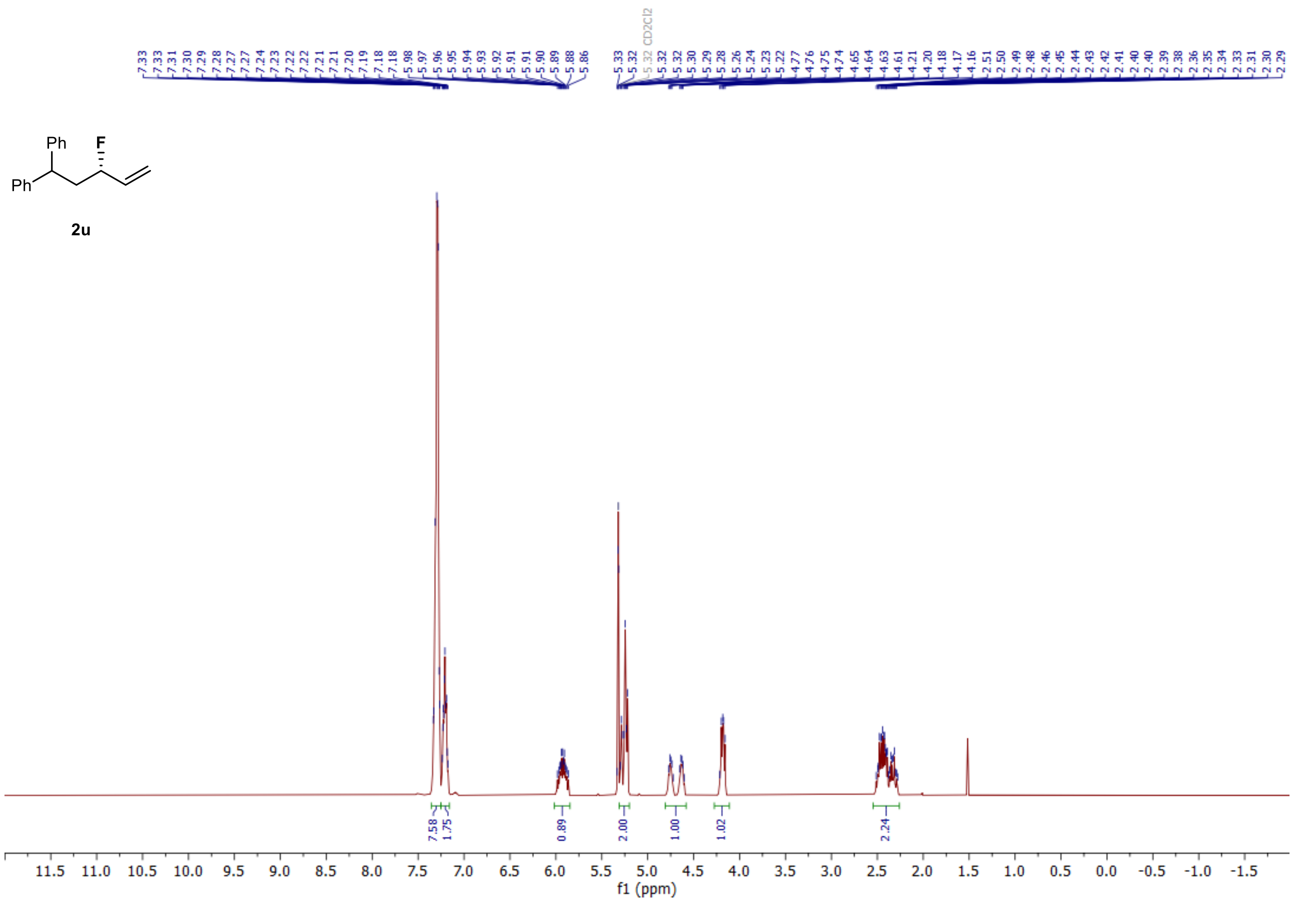

2u ^{13}C NMR (101 MHz, CD_2Cl_2)

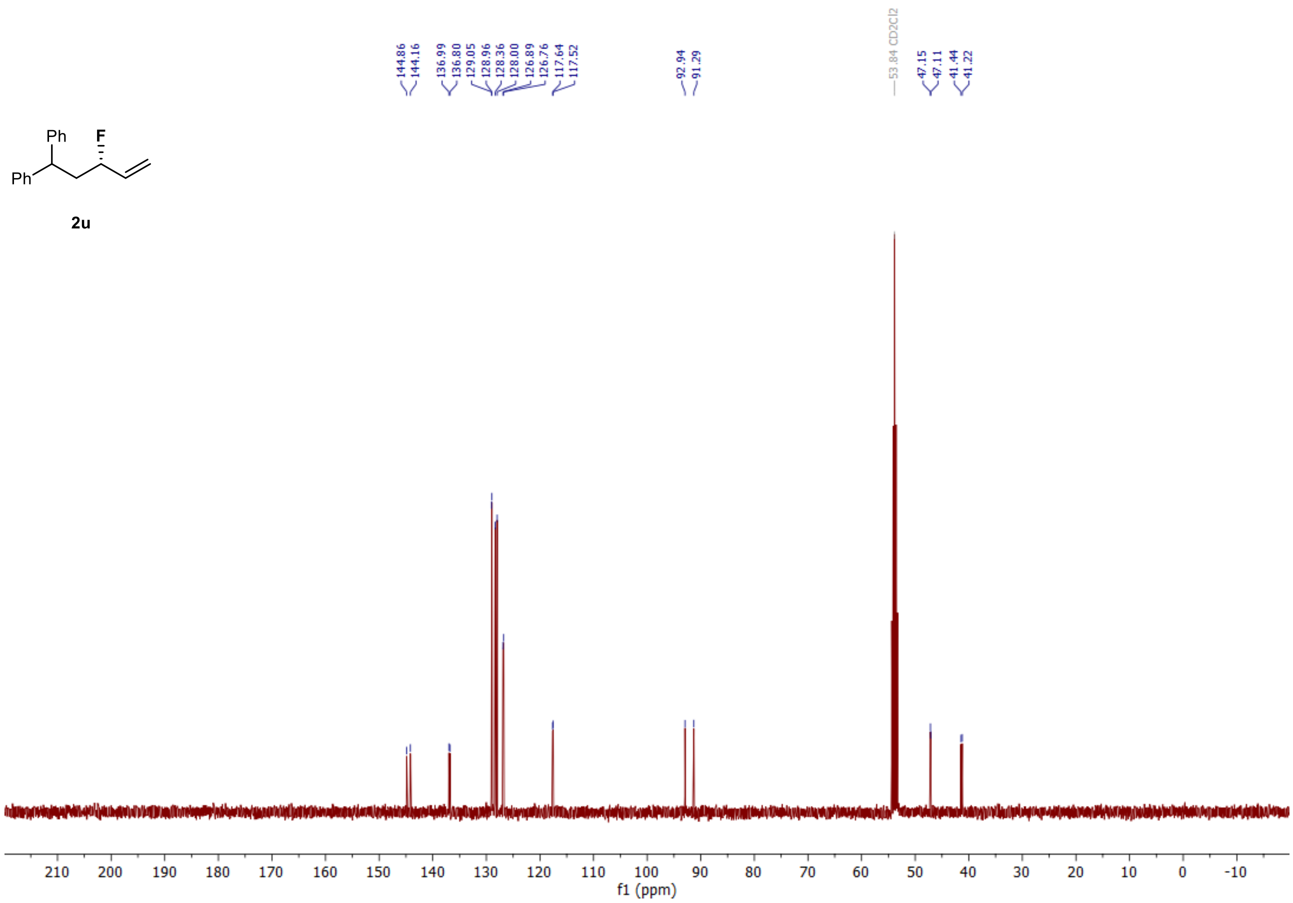

2u ^{19}F NMR (377 MHz, CD_2Cl_2)

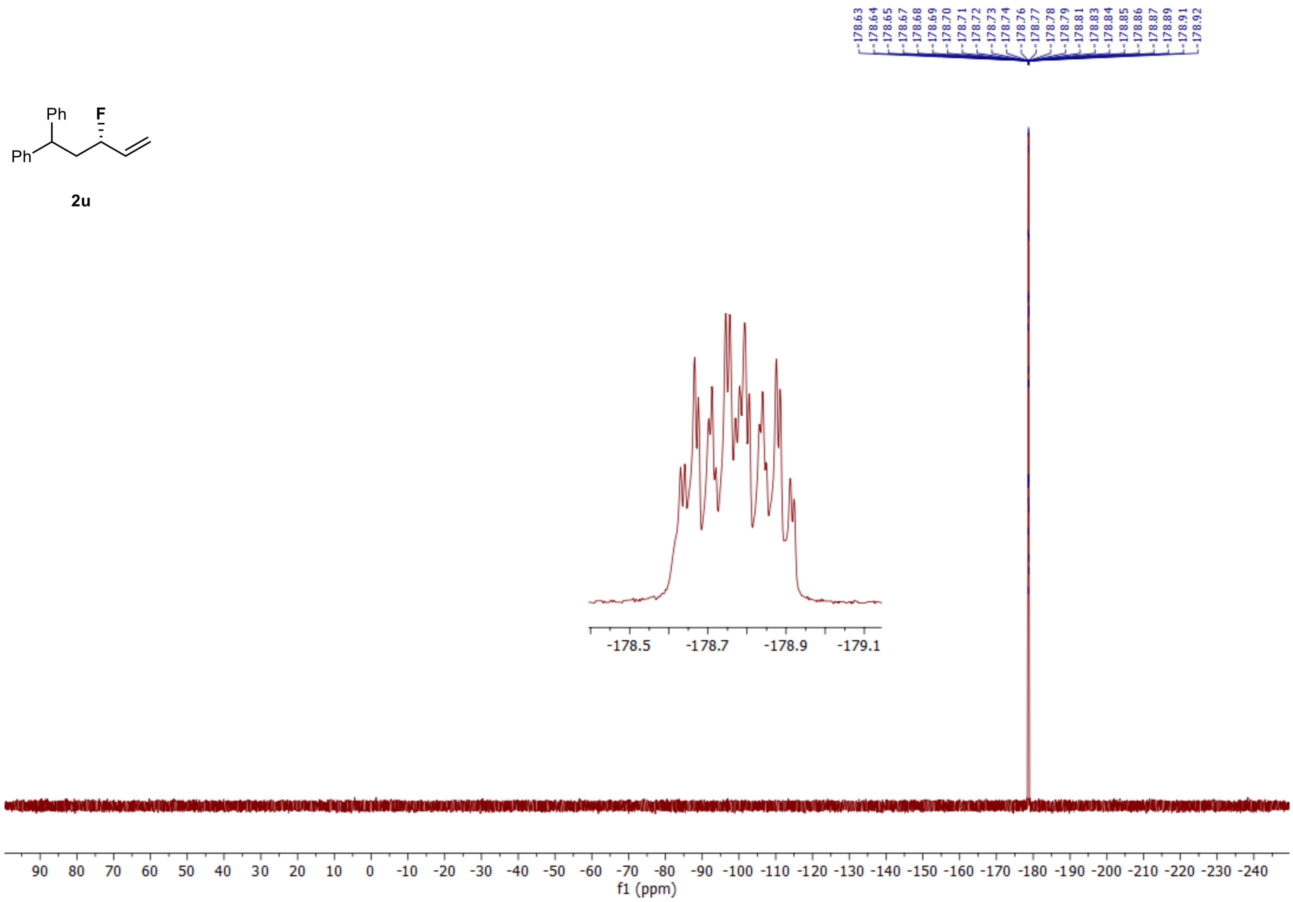

2v ^{1}H NMR (400 MHz, $CDCl_3$)

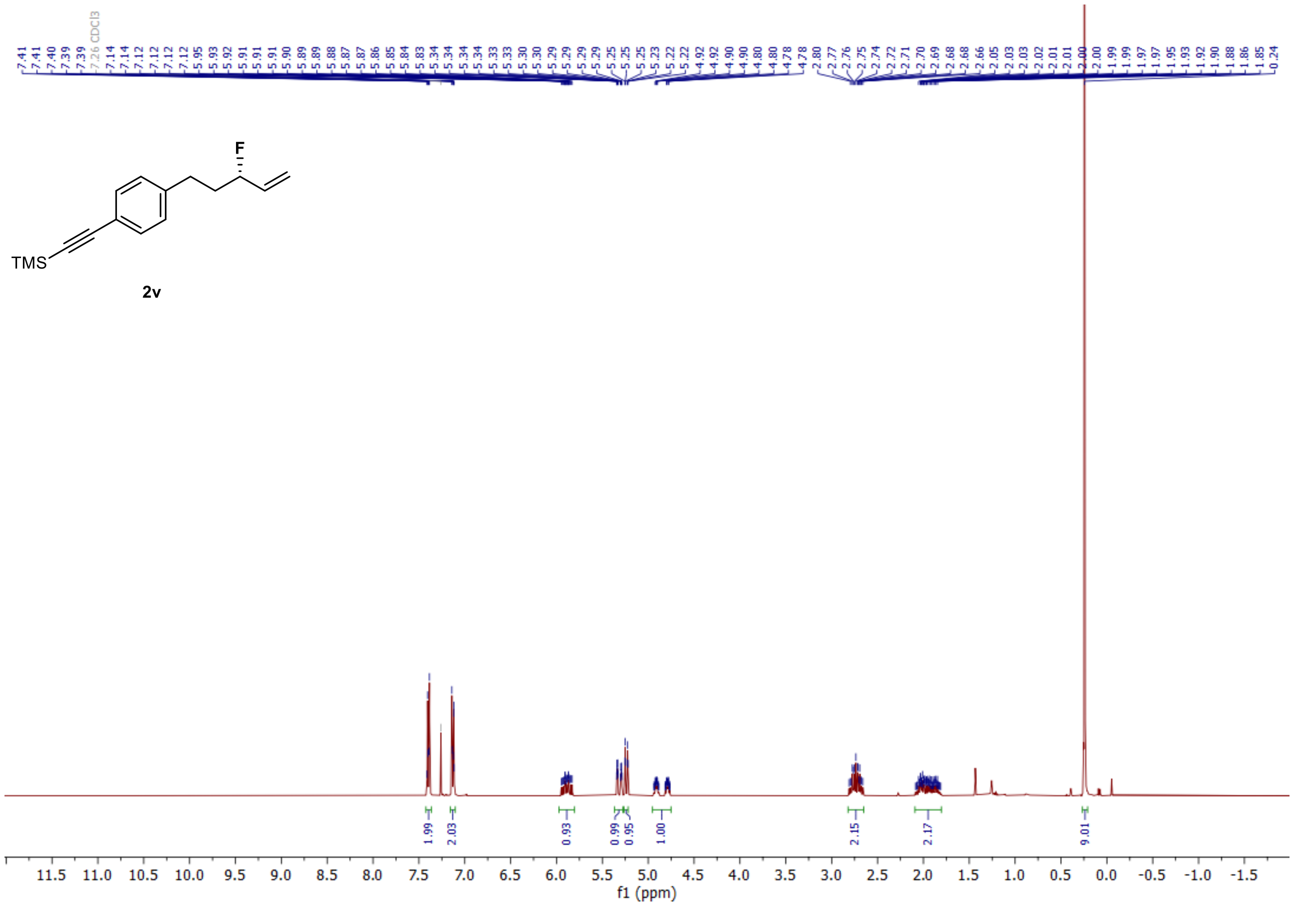

2v ^{13}C NMR (101 MHz, $CDCl_3$)

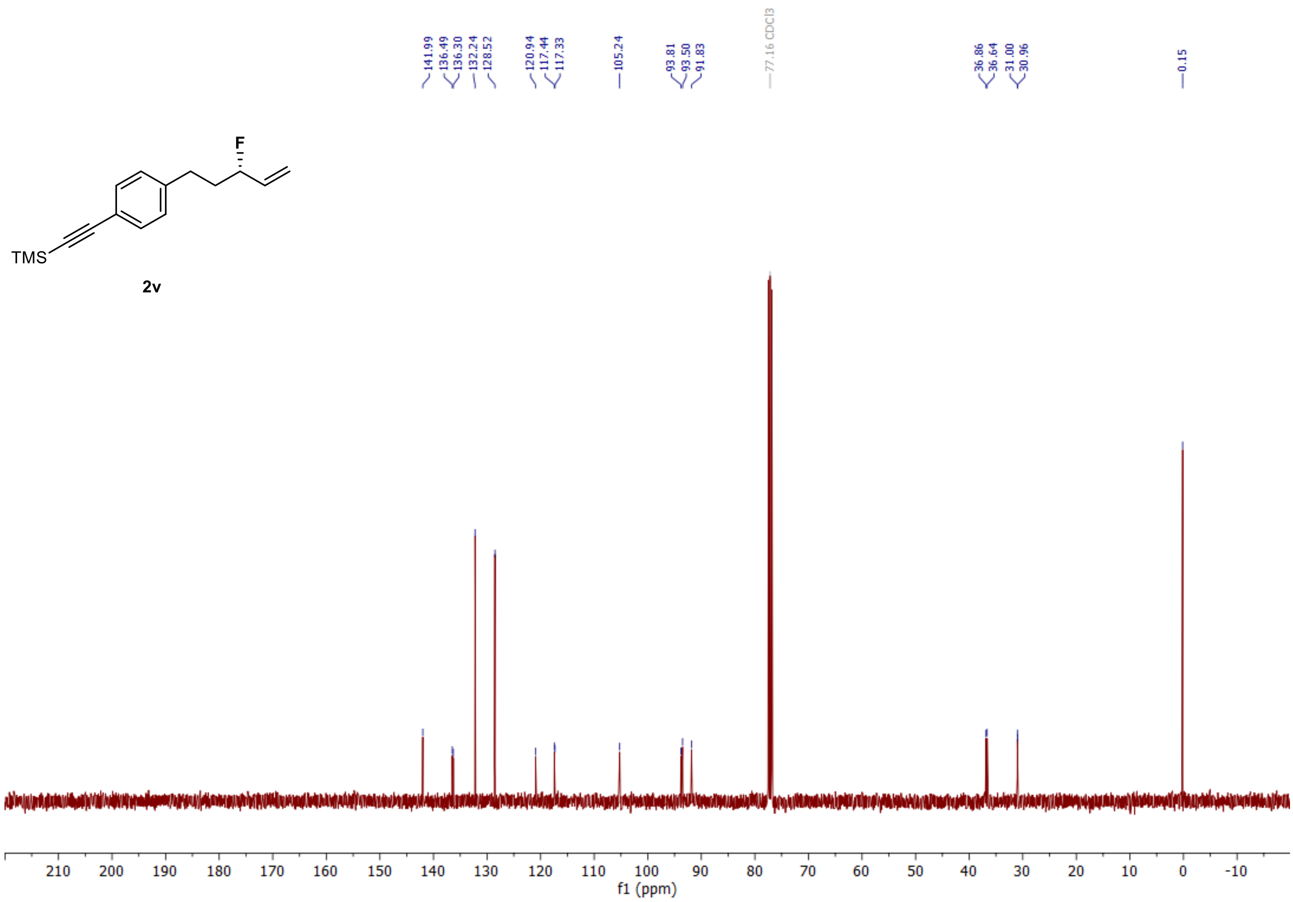

2v ^{19}F NMR (377 MHz, $CDCl_3$)

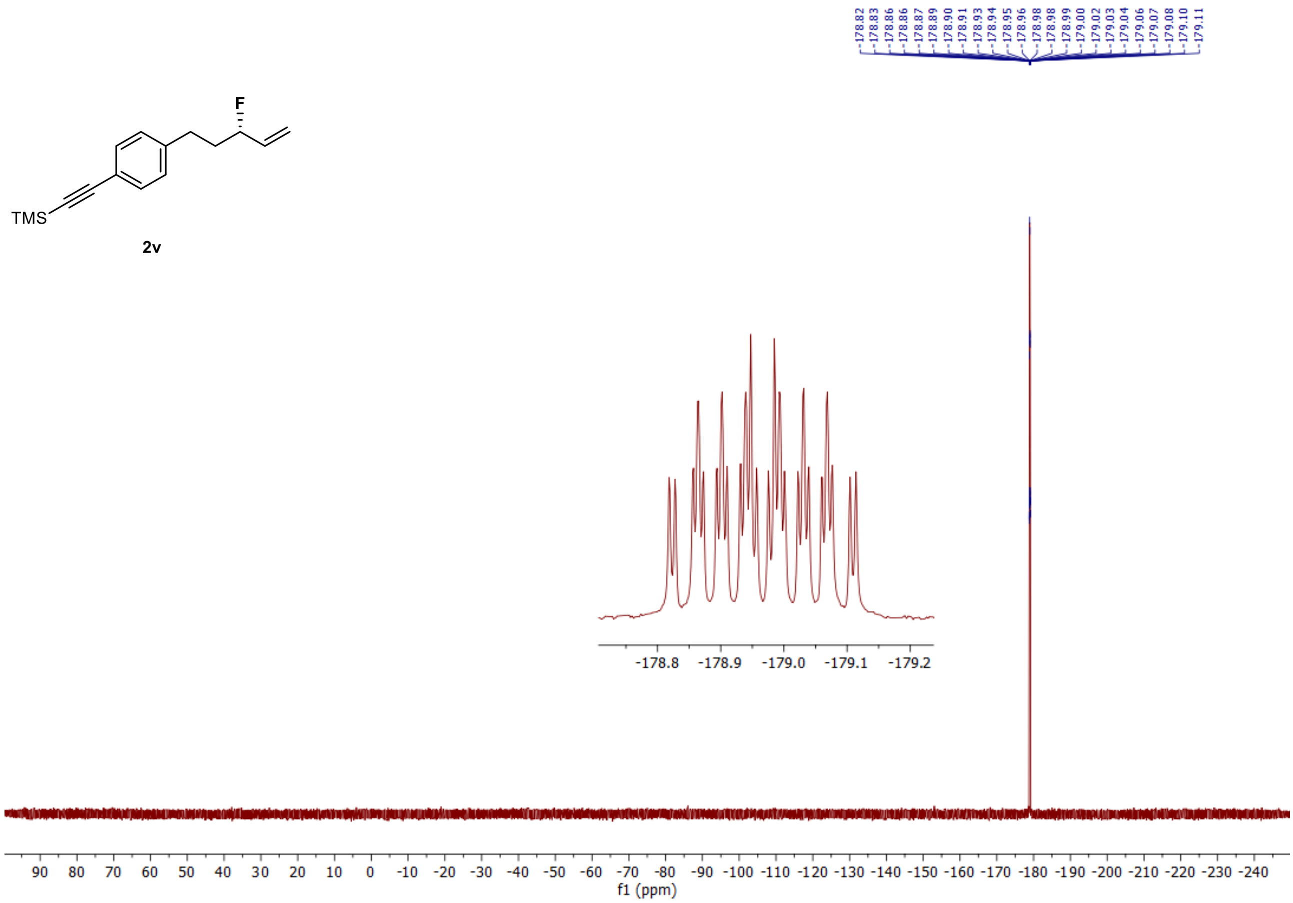

2w ^{1}H NMR (400 MHz, $CDCl_3$)

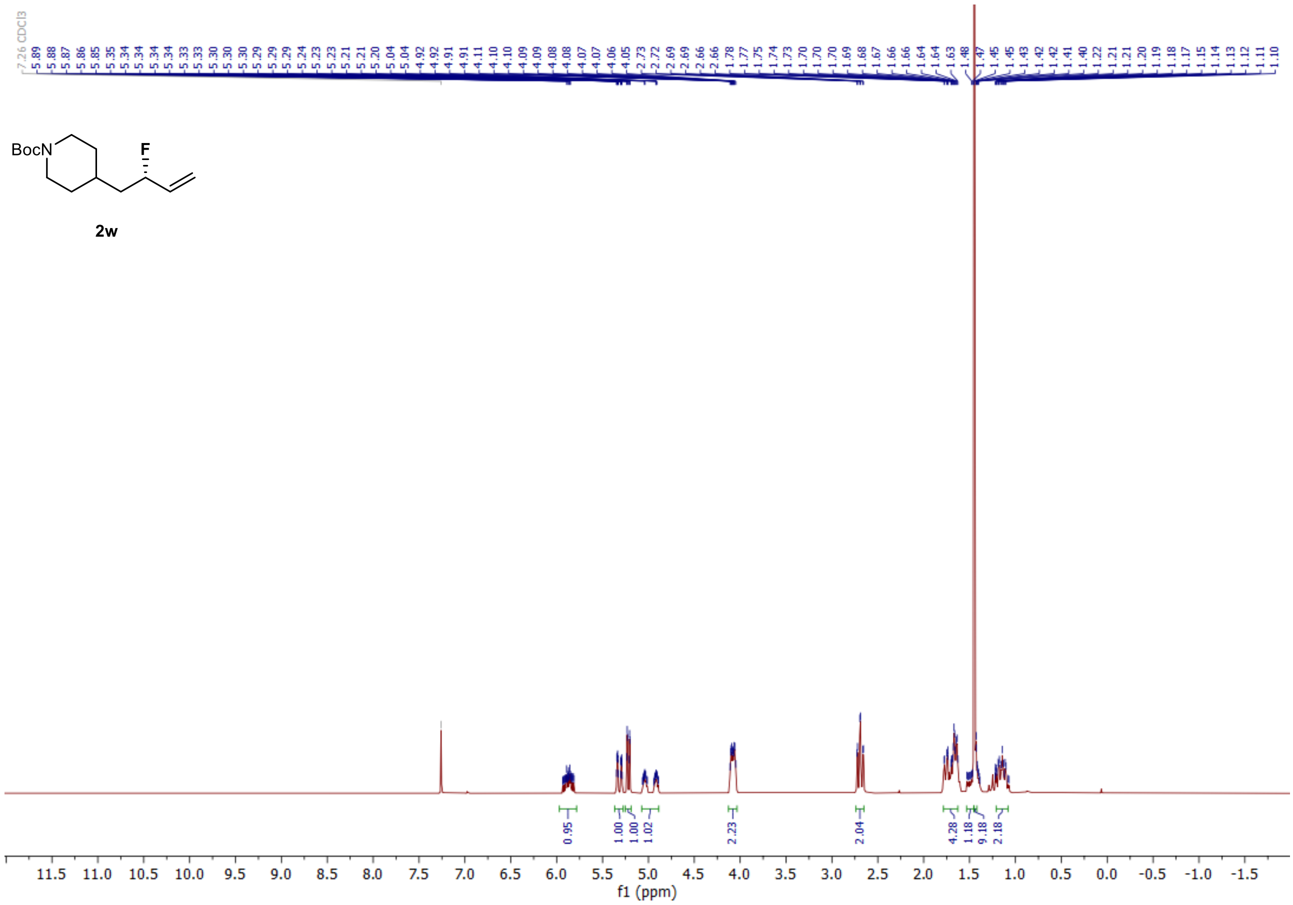

2w ^{13}C NMR (101 MHz, $CDCl_3$)

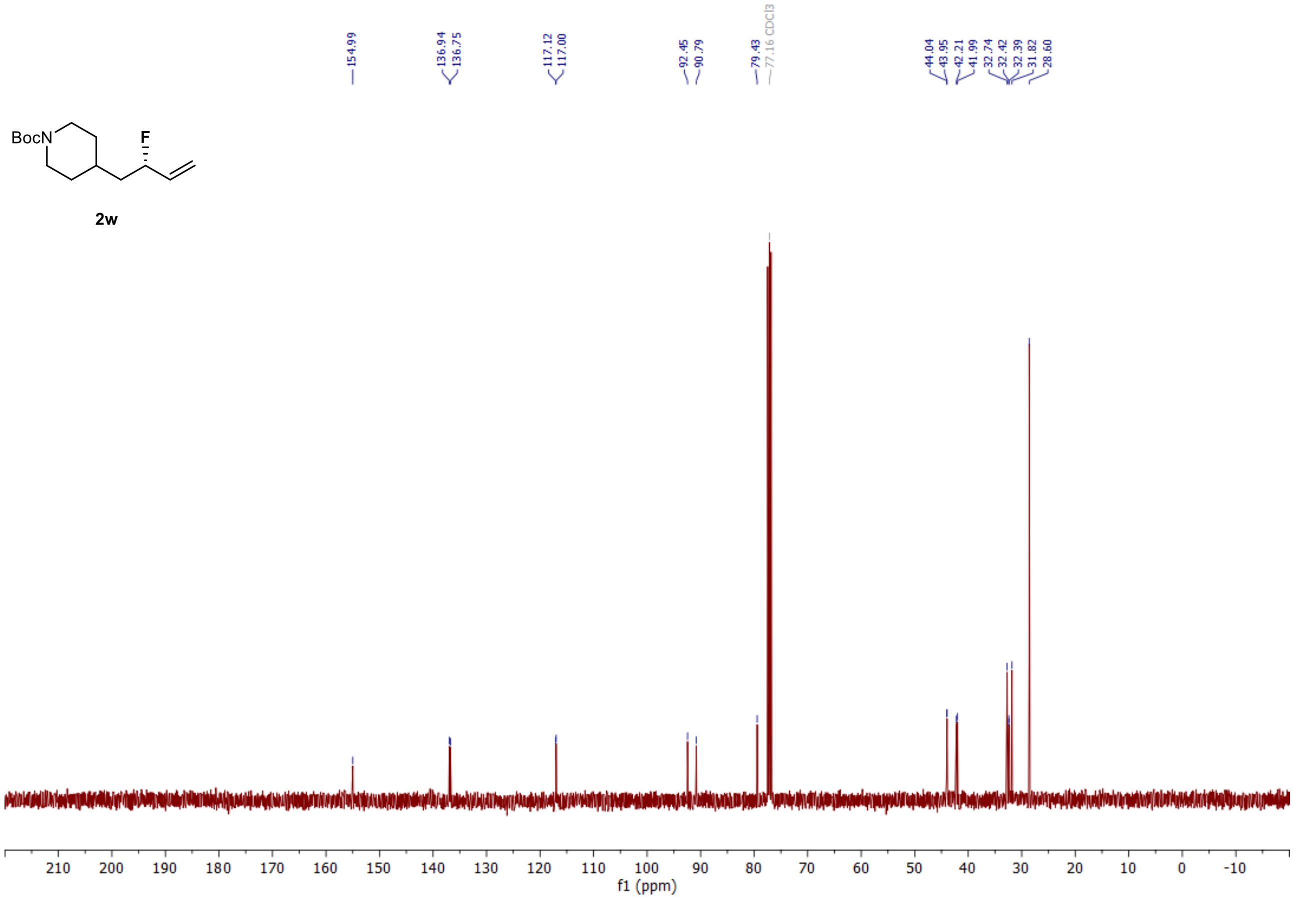

2w ^{19}F NMR (377 MHz, CDCl$_3$)

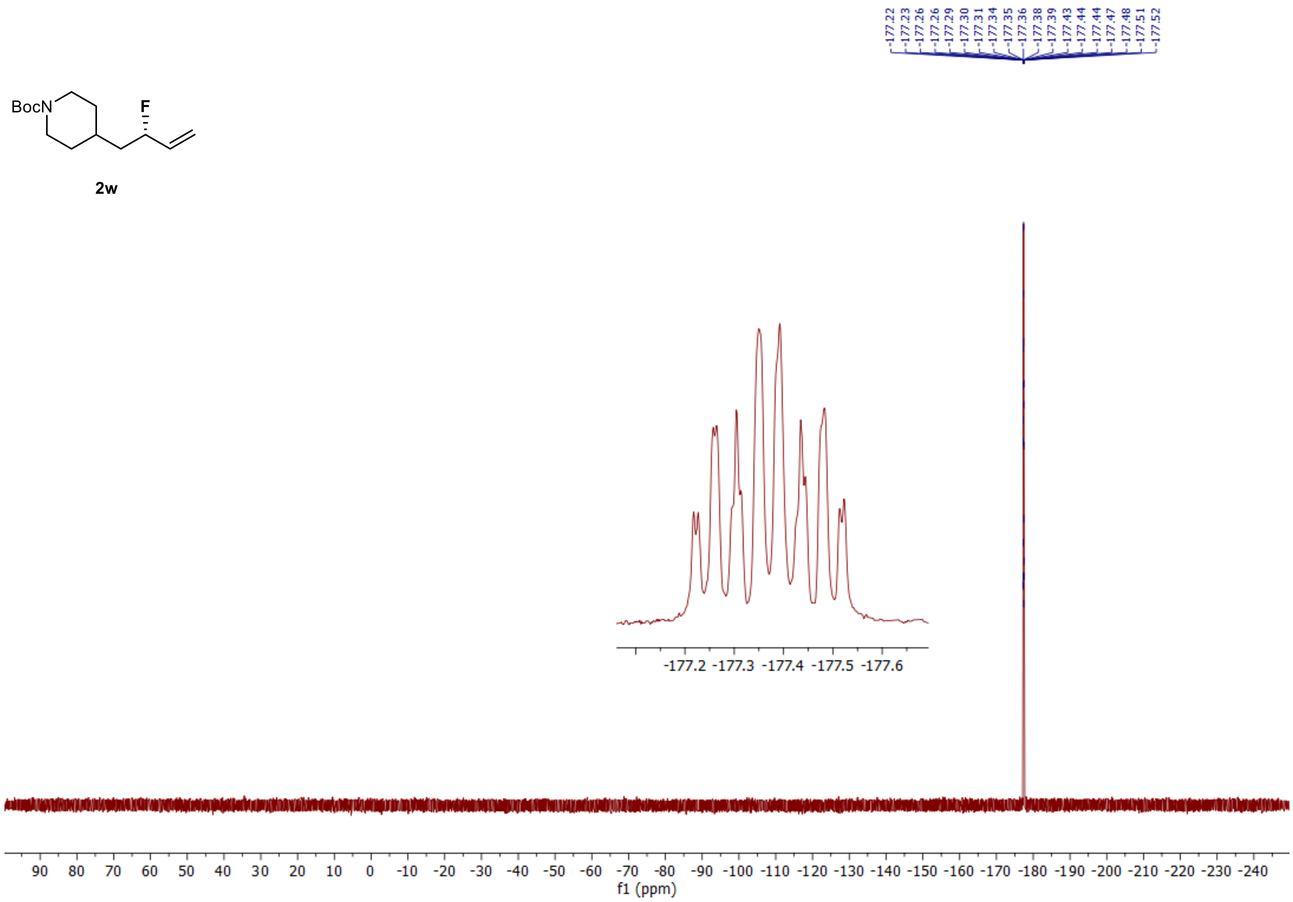

2x ^{1}H NMR (400 MHz, CDCl$_3$)

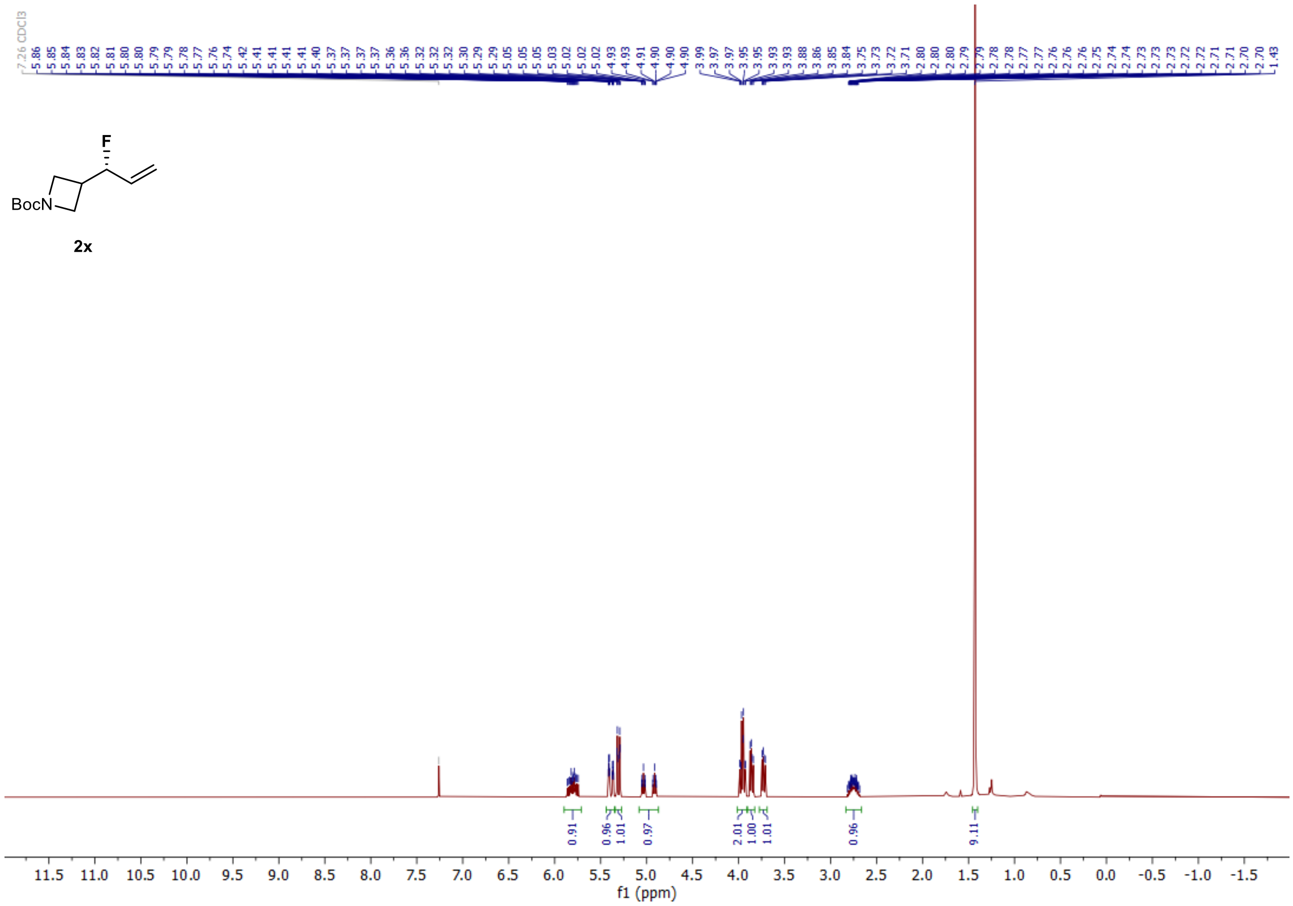

2x ^{13}C NMR (101 MHz, $CDCl_3$)

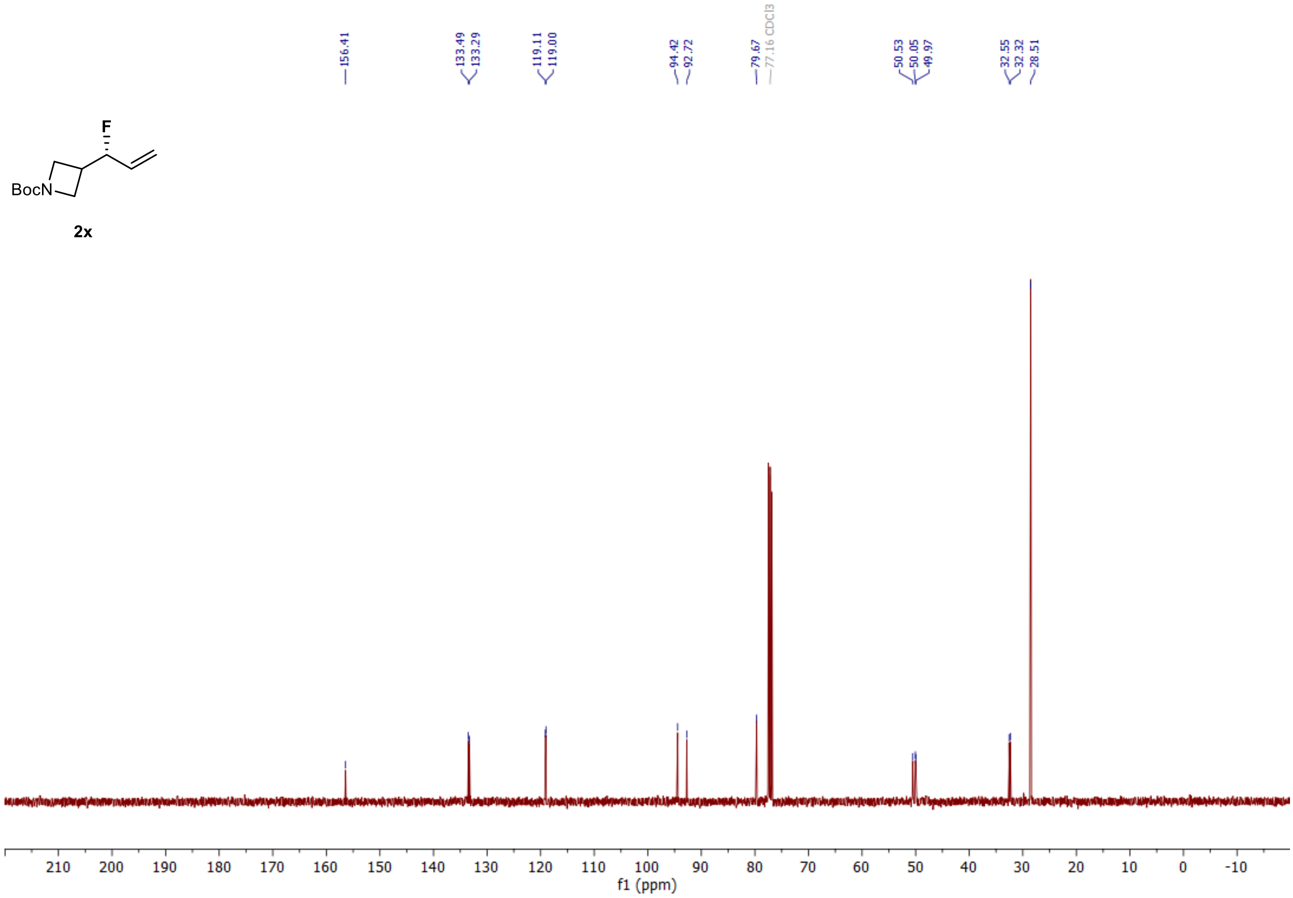

2x ^{19}F NMR (377 MHz, $CDCl_3$)

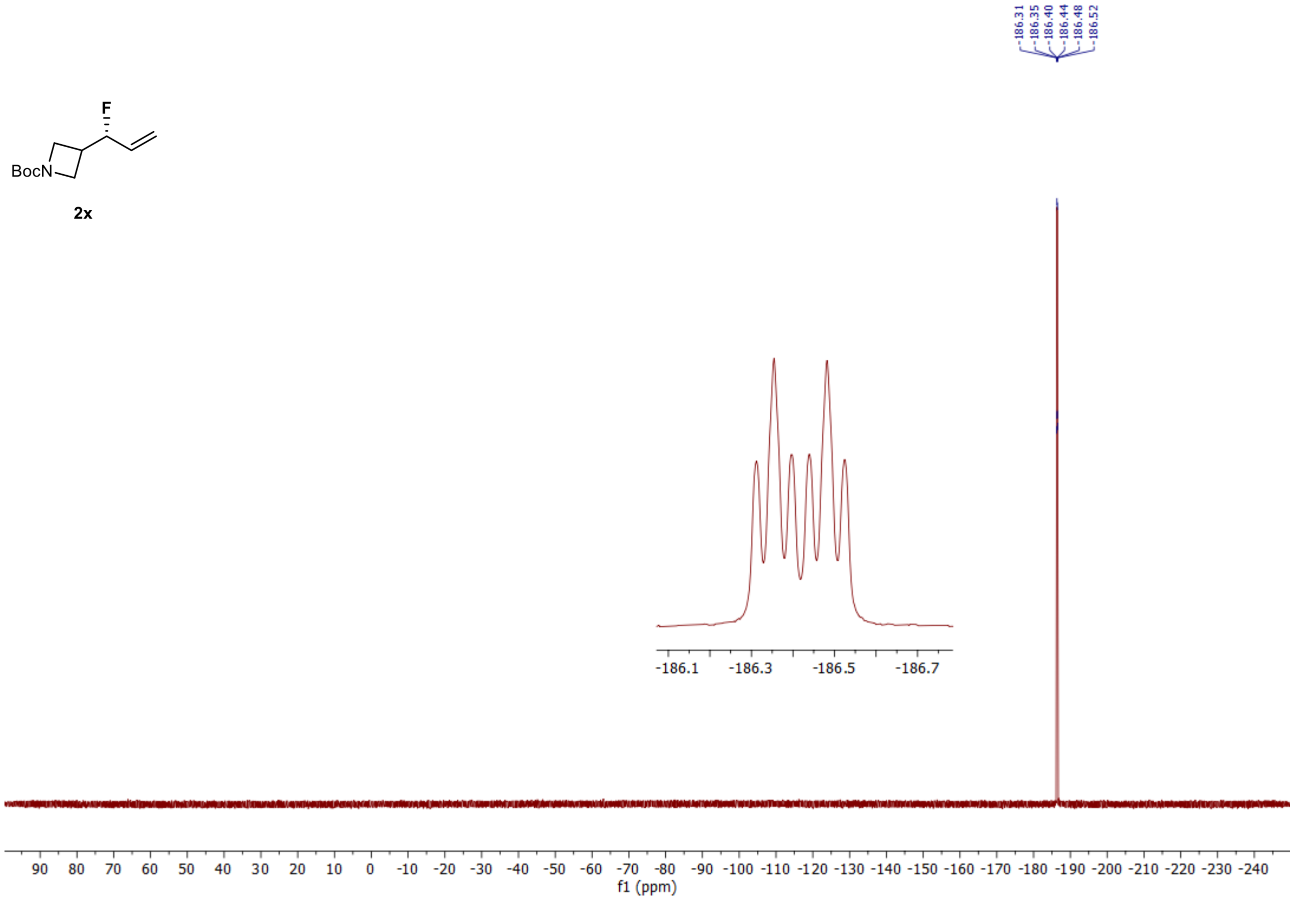

2y ^{1}H NMR (400 MHz, $CDCl_3$)

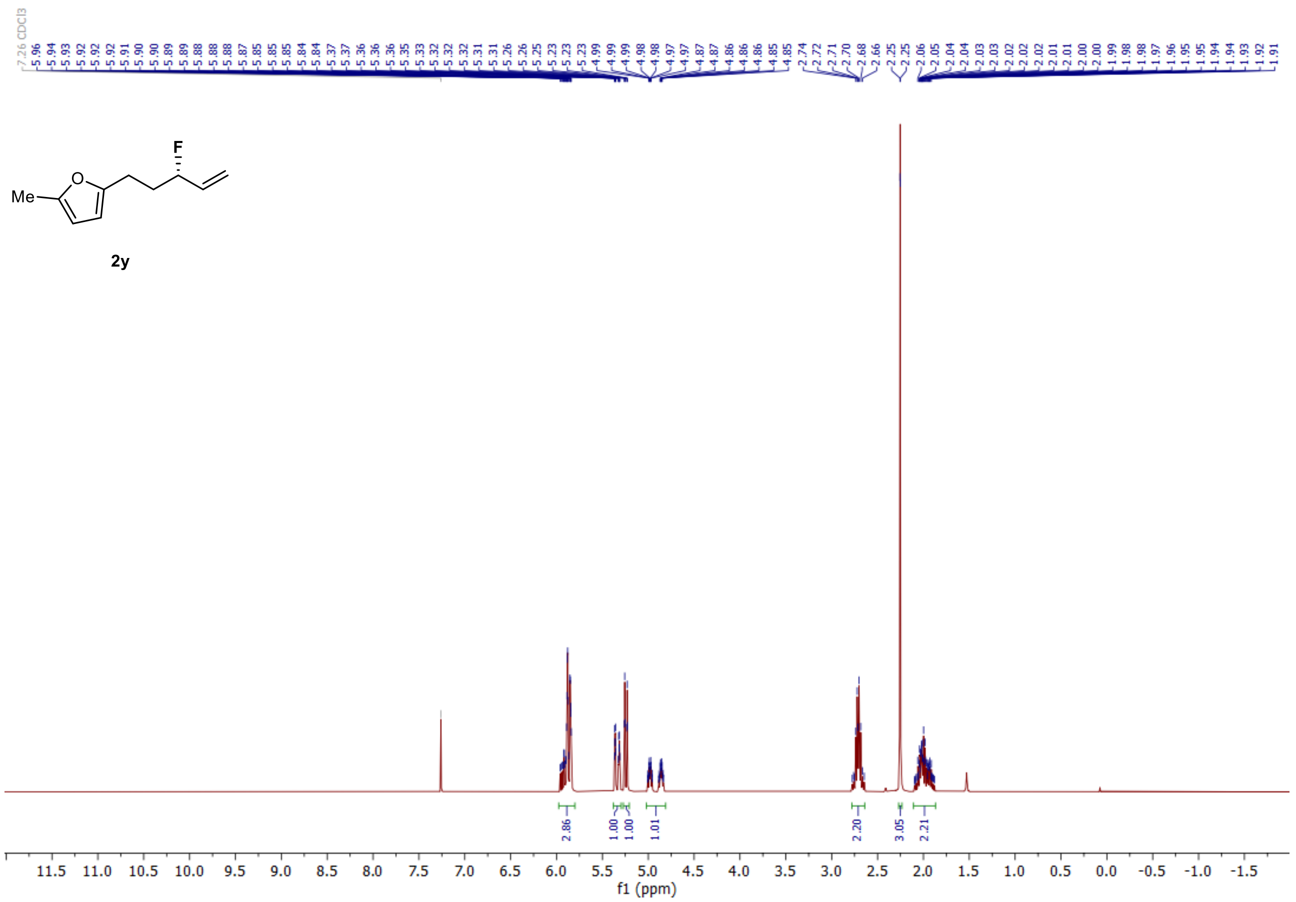

2y ^{13}C NMR (101 MHz, $CDCl_3$)

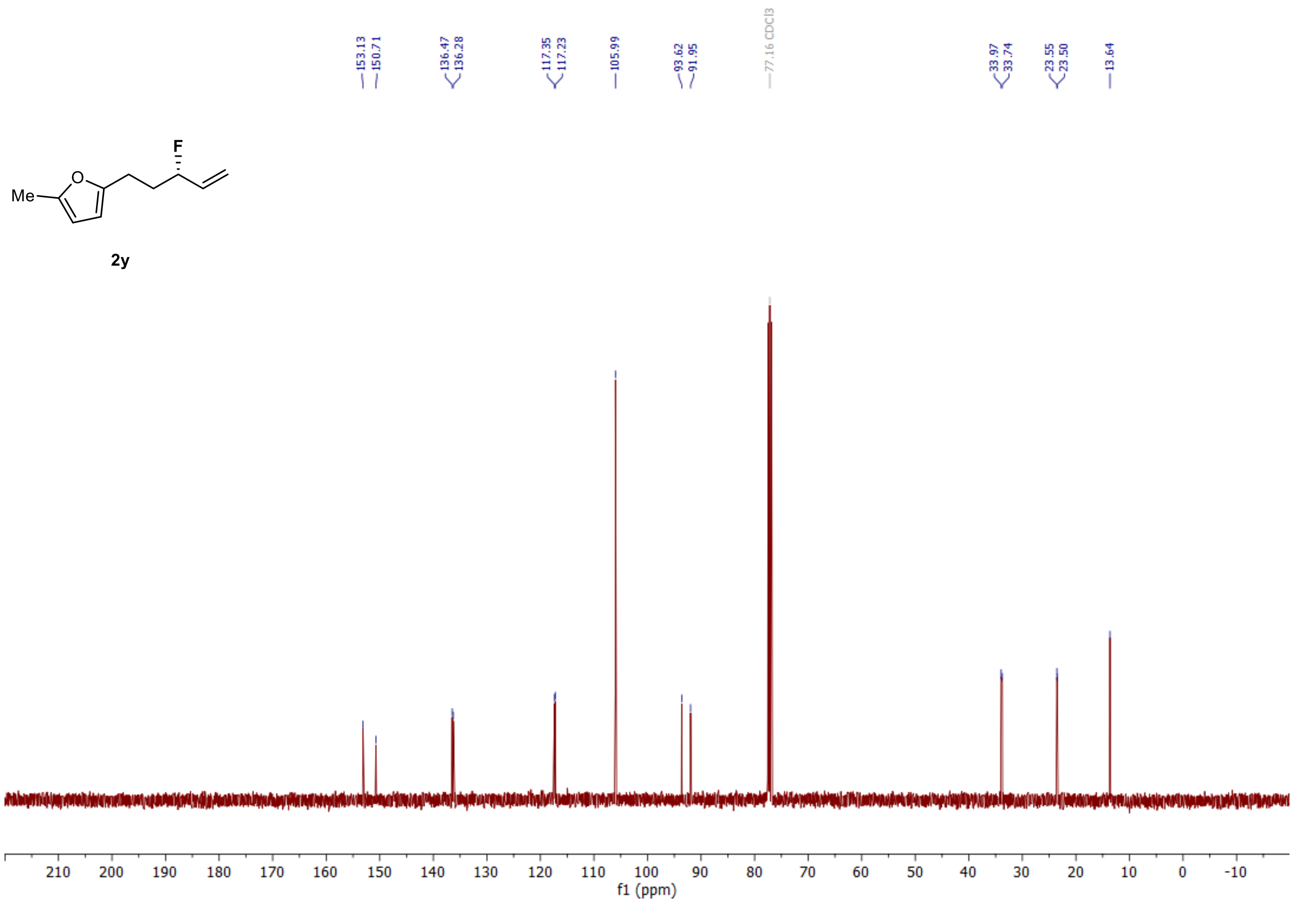

2y ^{19}F NMR (377 MHz, CDCl$_3$)

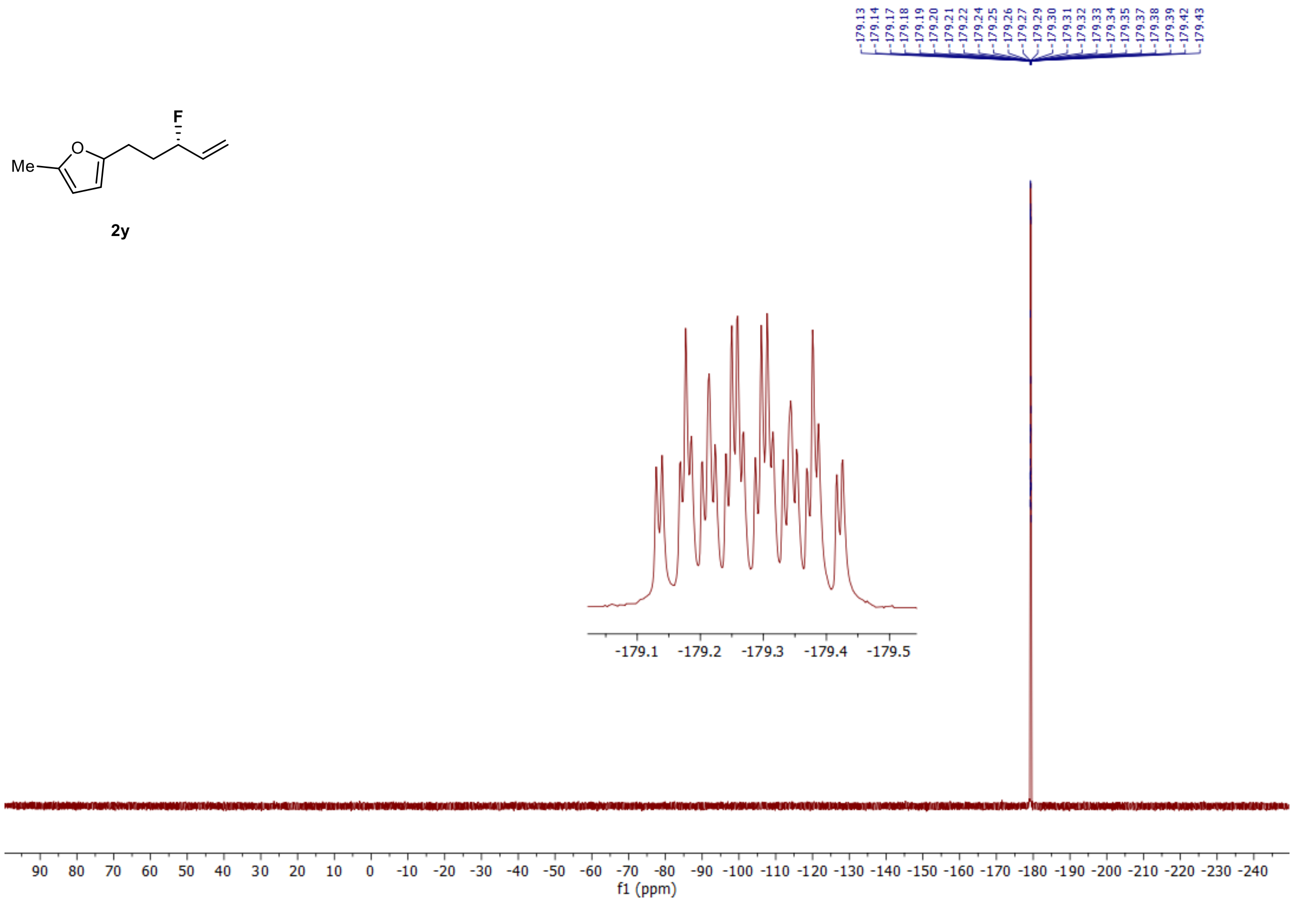

2z ^{1}H NMR (400 MHz, CDCl$_3$)

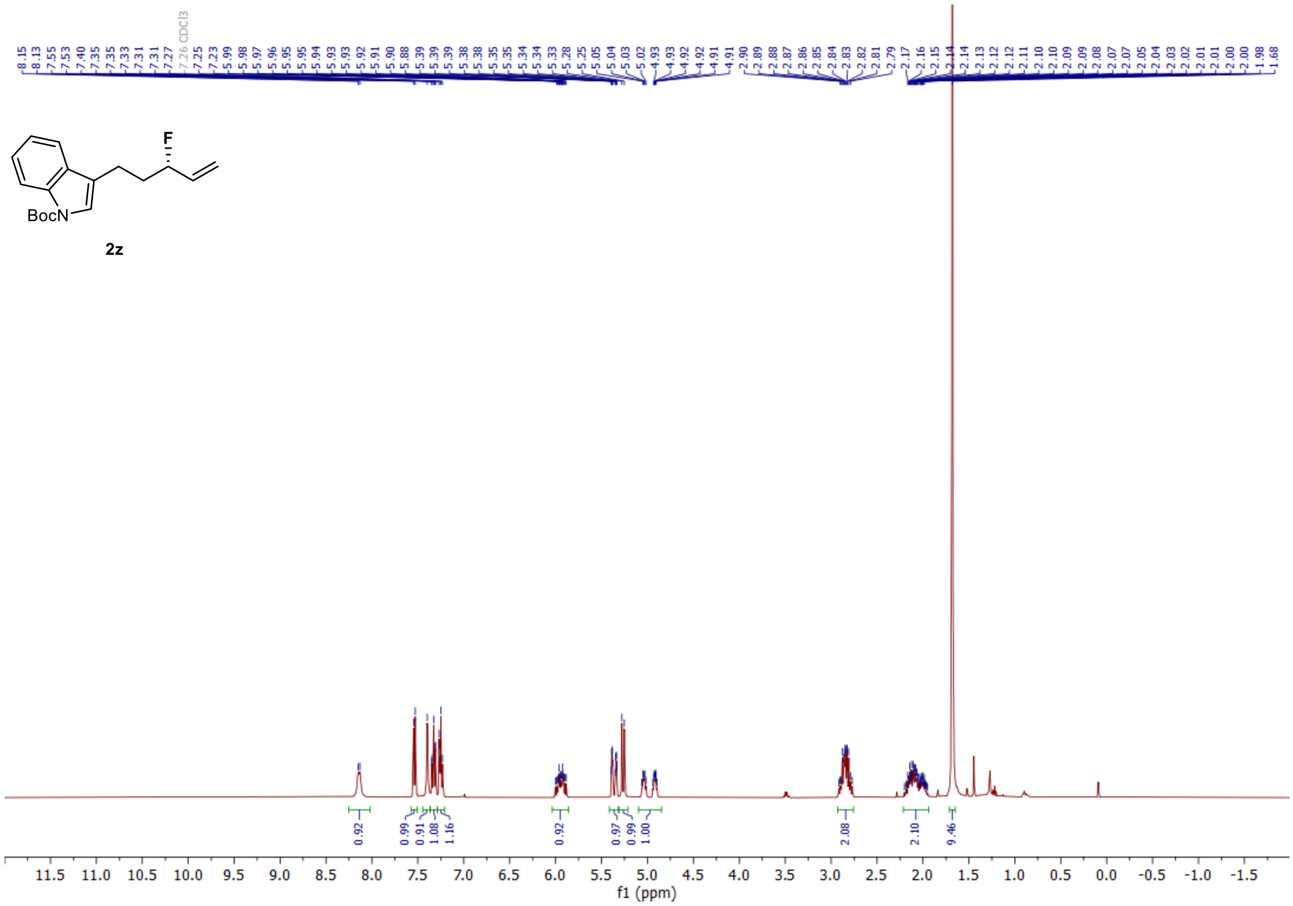

2z ^{13}C NMR (101 MHz, $CDCl_3$)

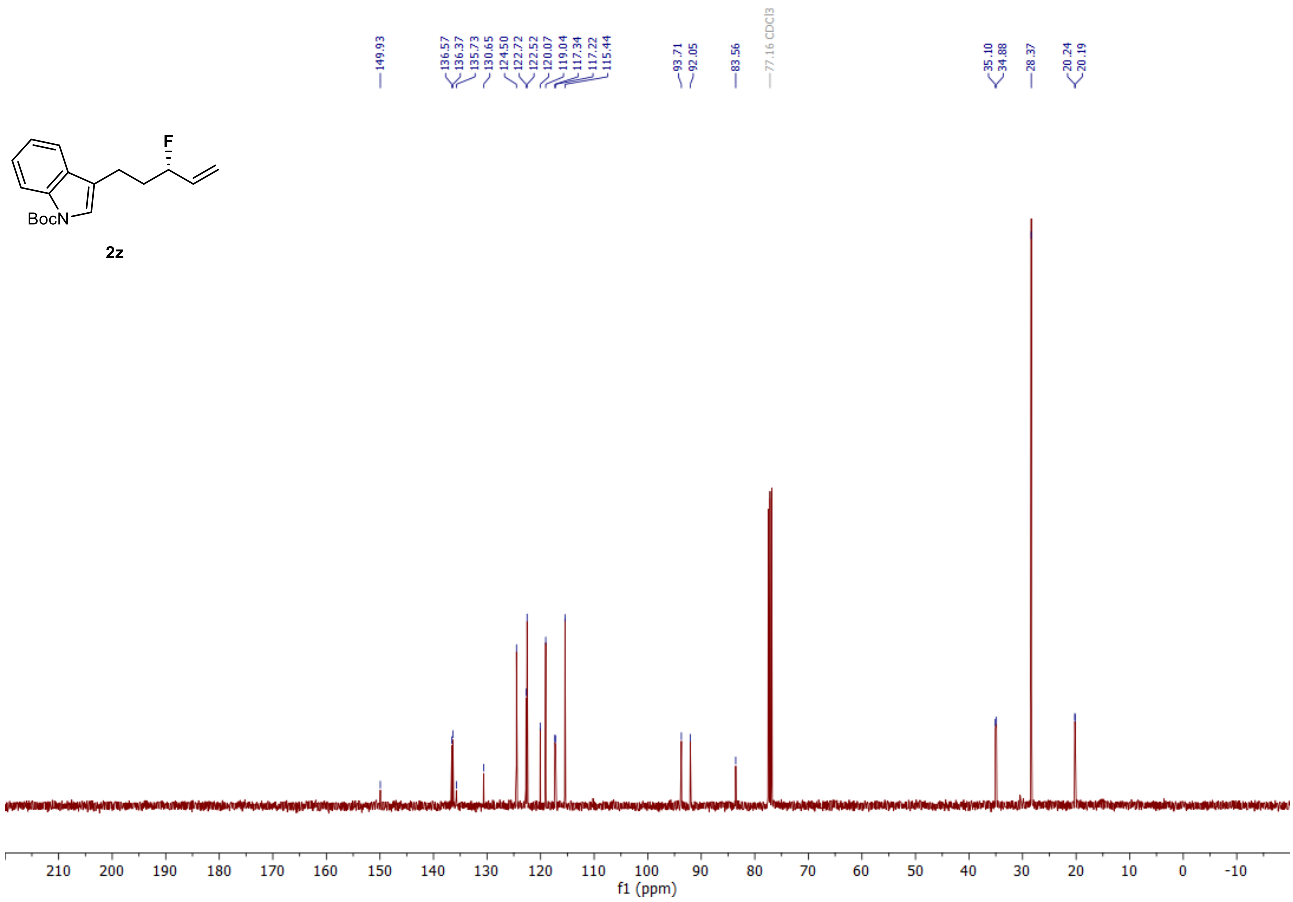

2z ^{19}F NMR (377 MHz, $CDCl_3$)

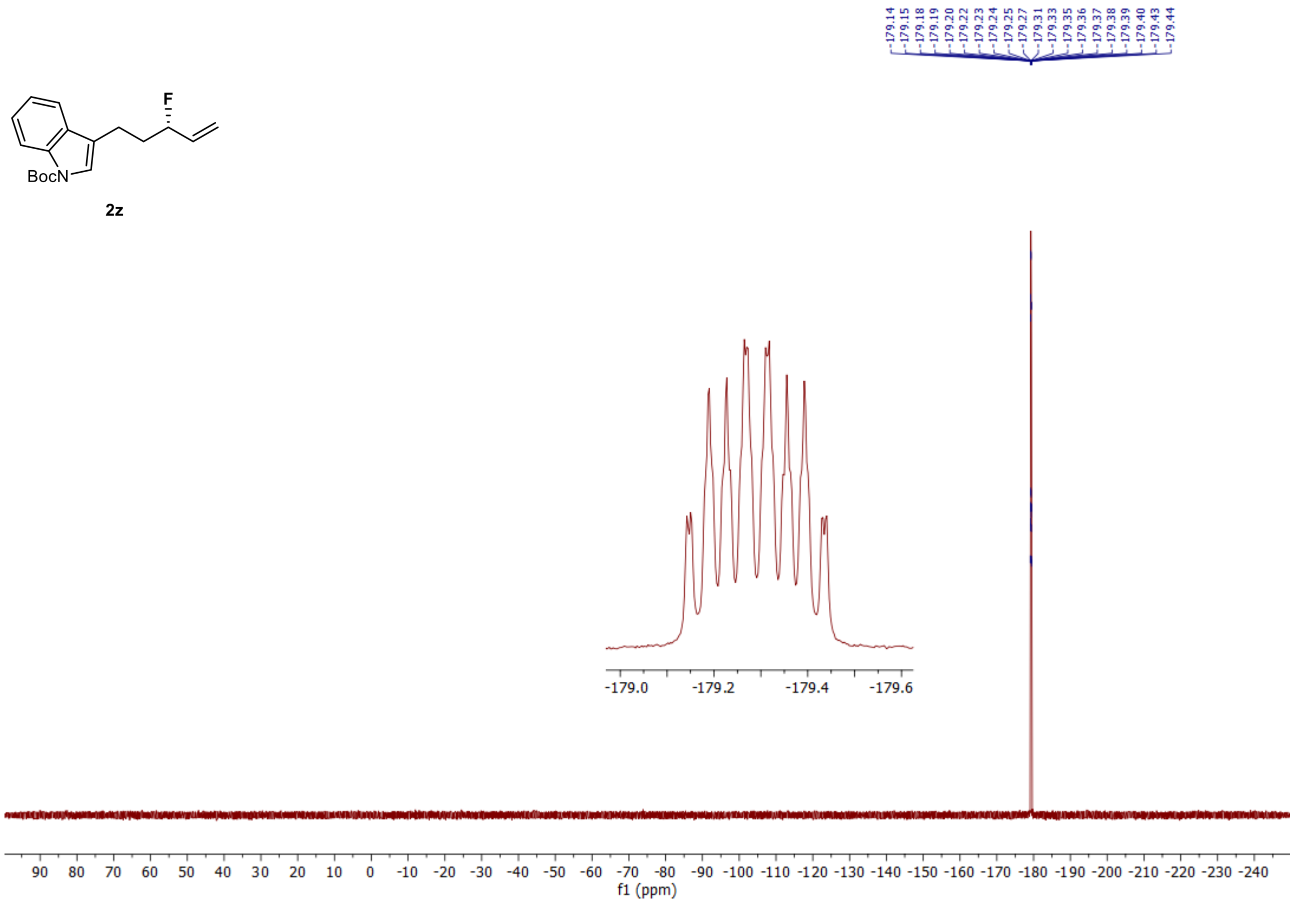

2aa ^{1}H NMR (400 MHz, $CDCl_3$)

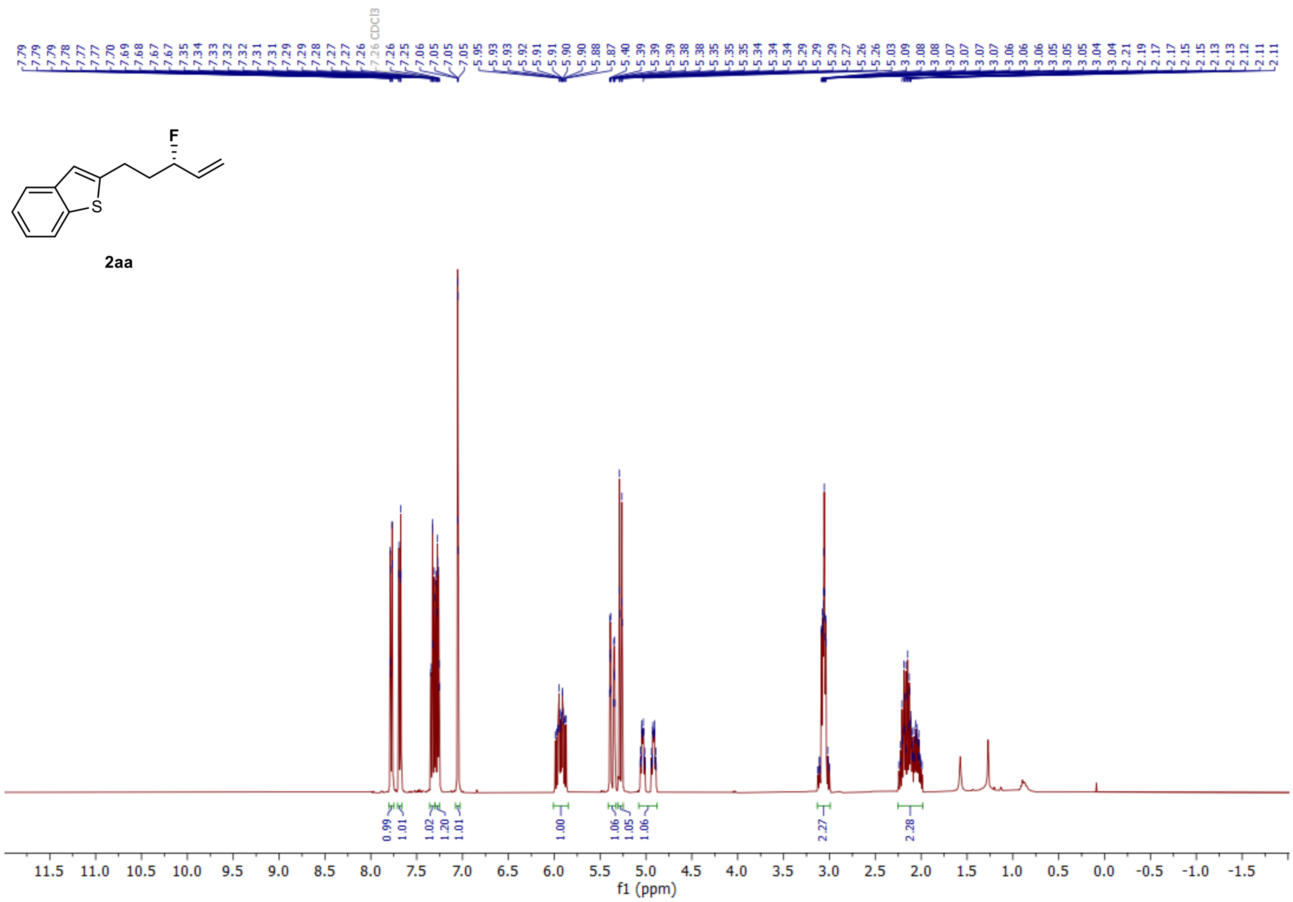

2aa

2aa ^{13}C NMR (101 MHz, $CDCl_3$)

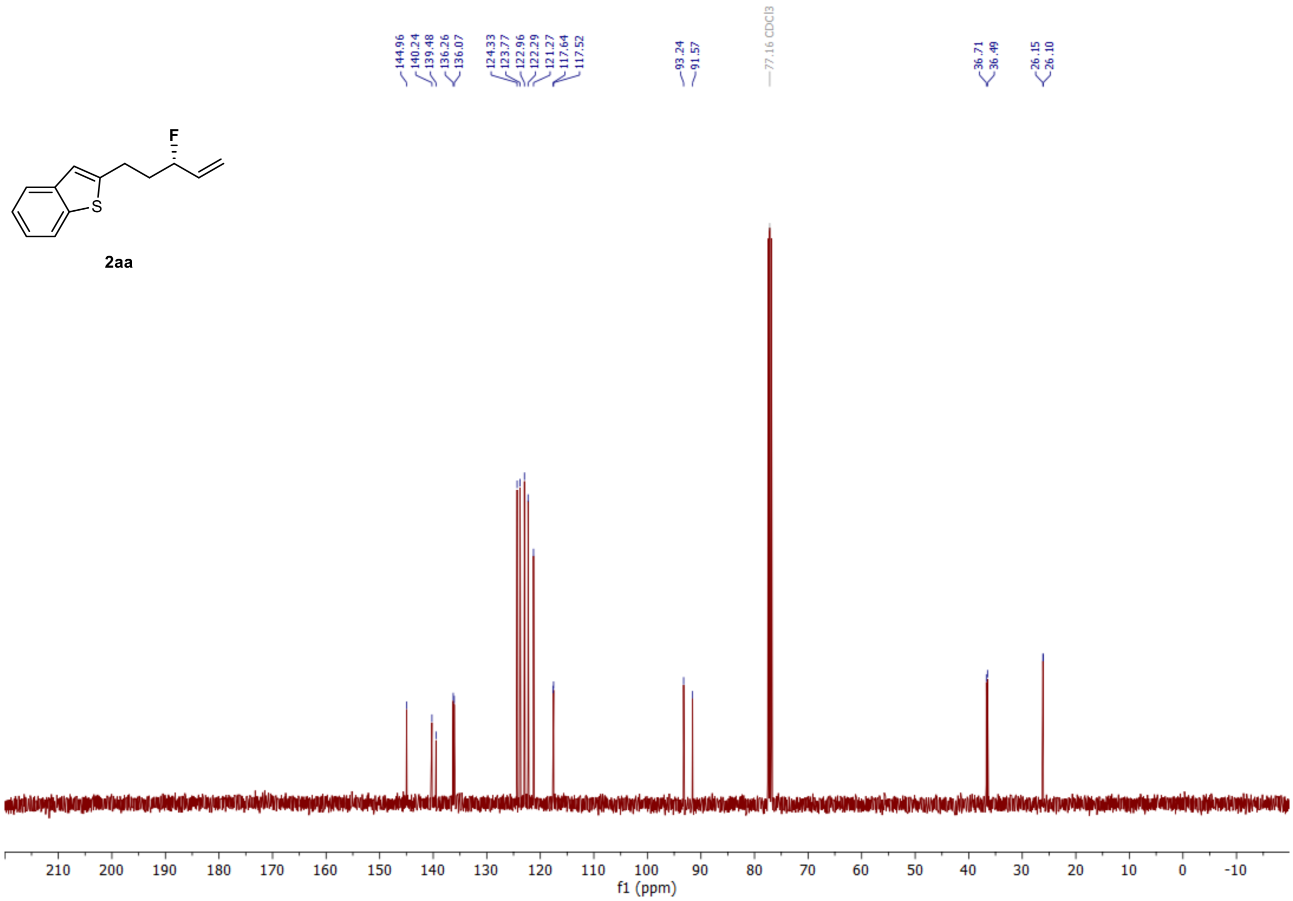

2aa

2aa ^{19}F NMR (377 MHz, $CDCl_3$)

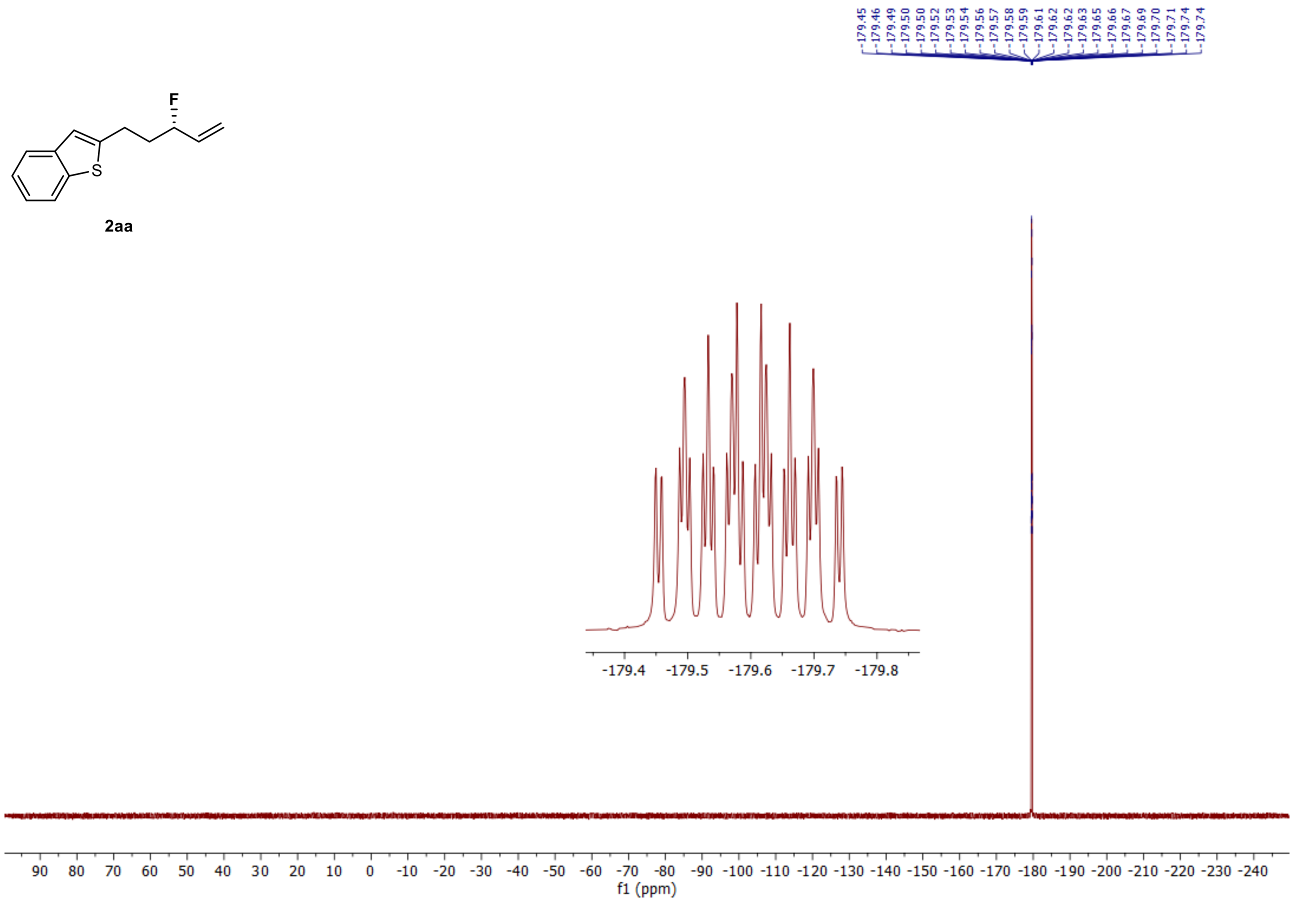

2ab ^{1}H NMR (400 MHz, $CDCl_3$)

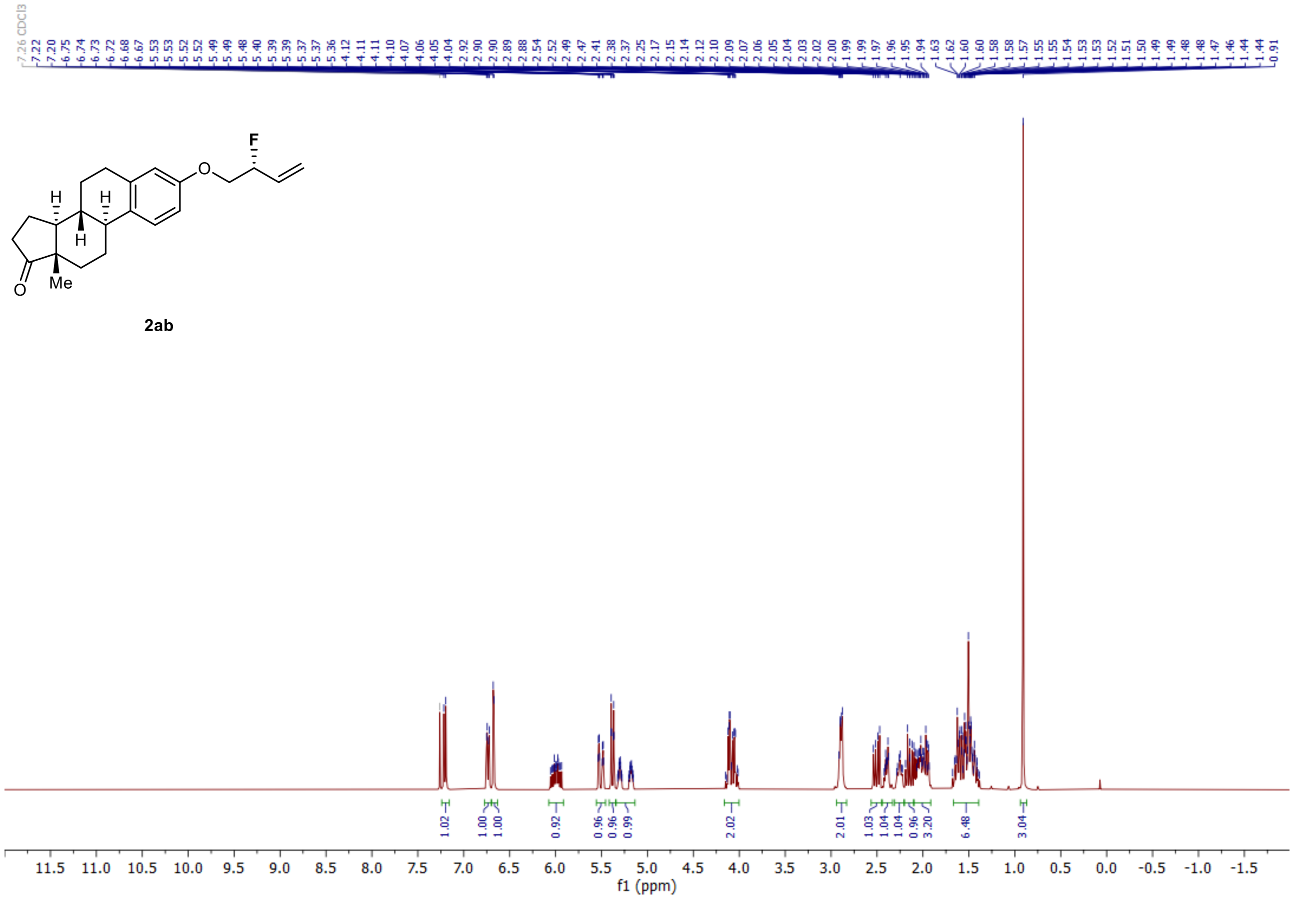

2ab ^{13}C NMR (101 MHz, $CDCl_3$)

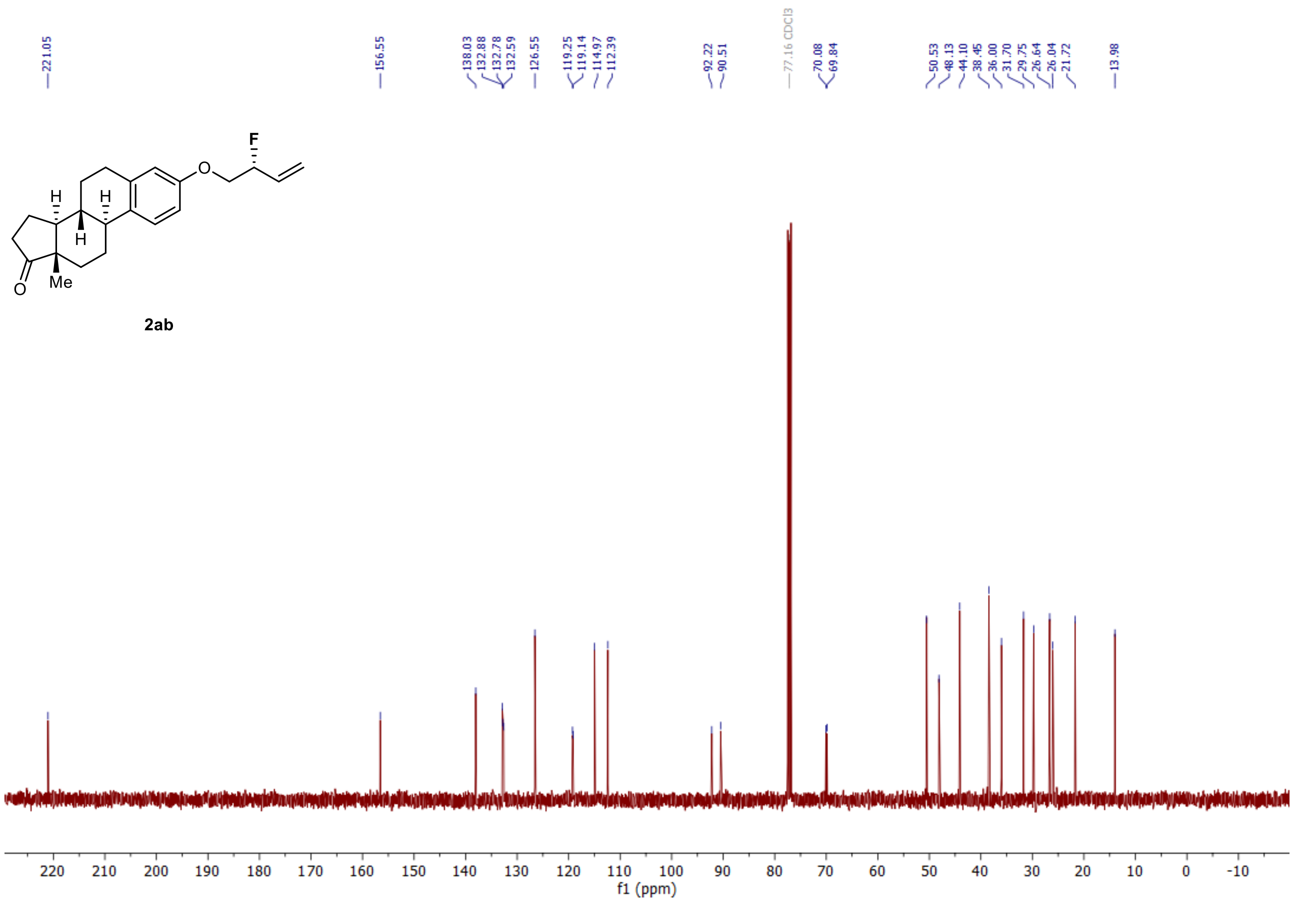

2ab ^{19}F NMR (377 MHz, $CDCl_3$)

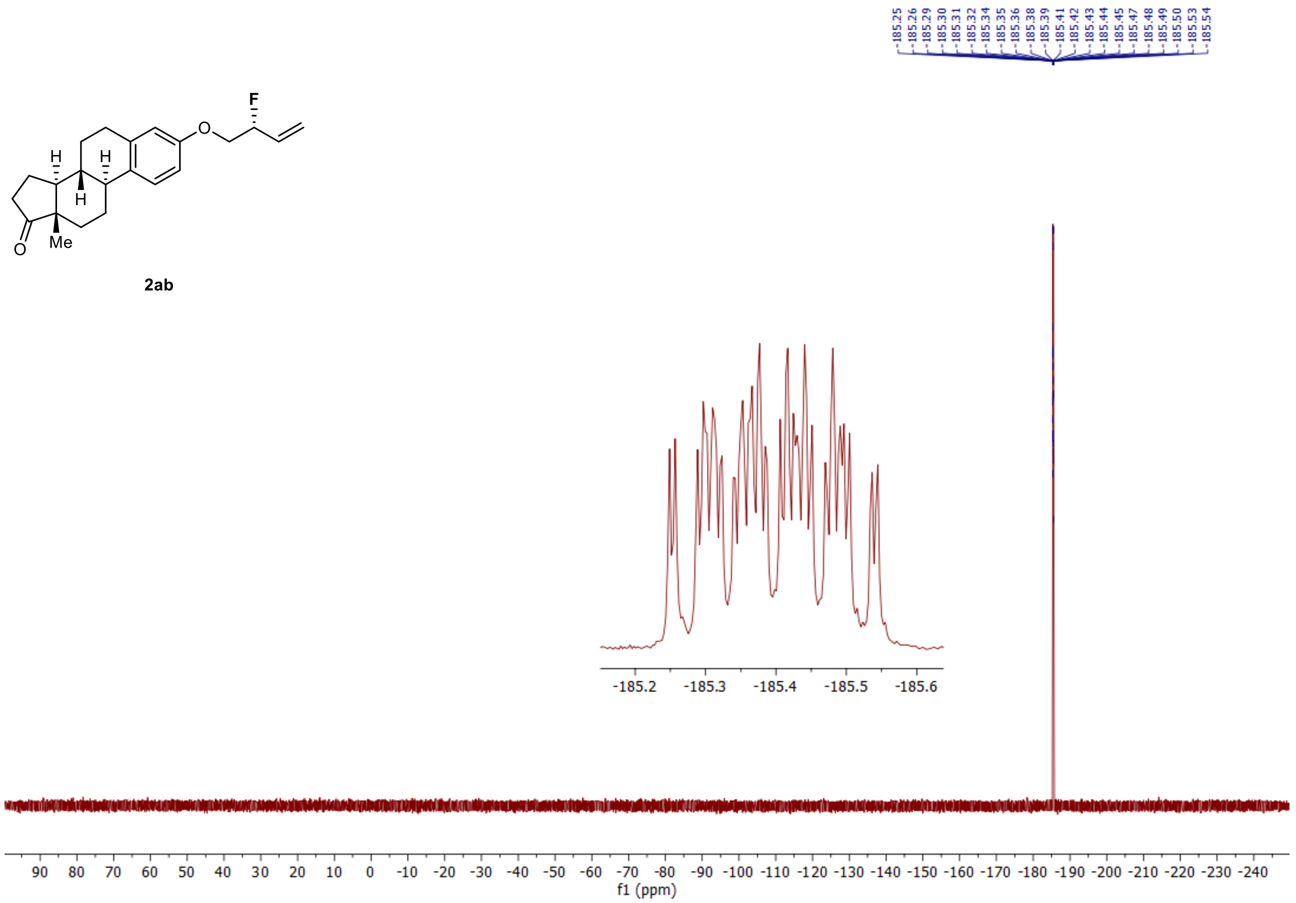

7 ^{1}H NMR (400 MHz, $CDCl_3$)

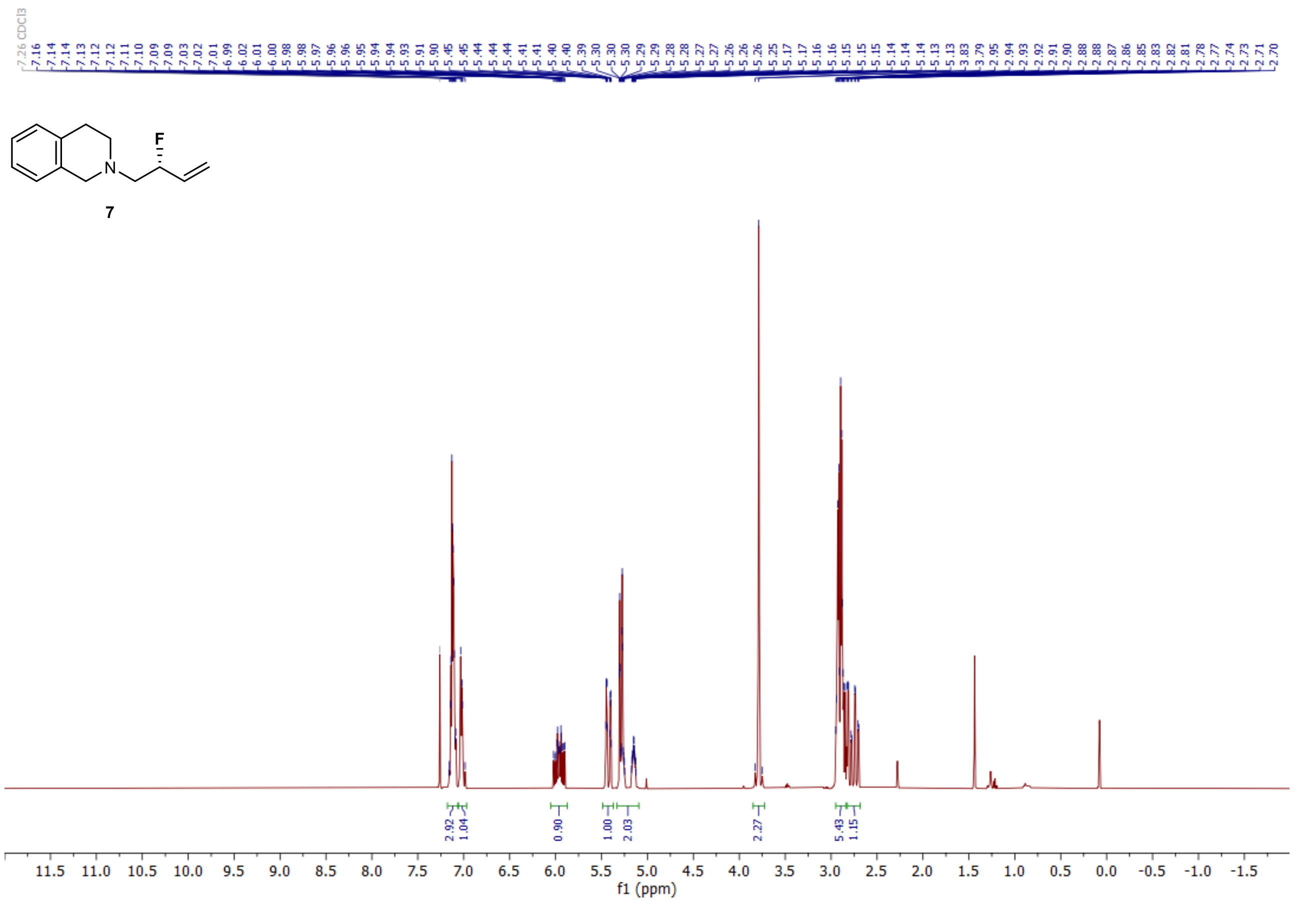

7 ^{13}C NMR (101 MHz, $CDCl_3$)

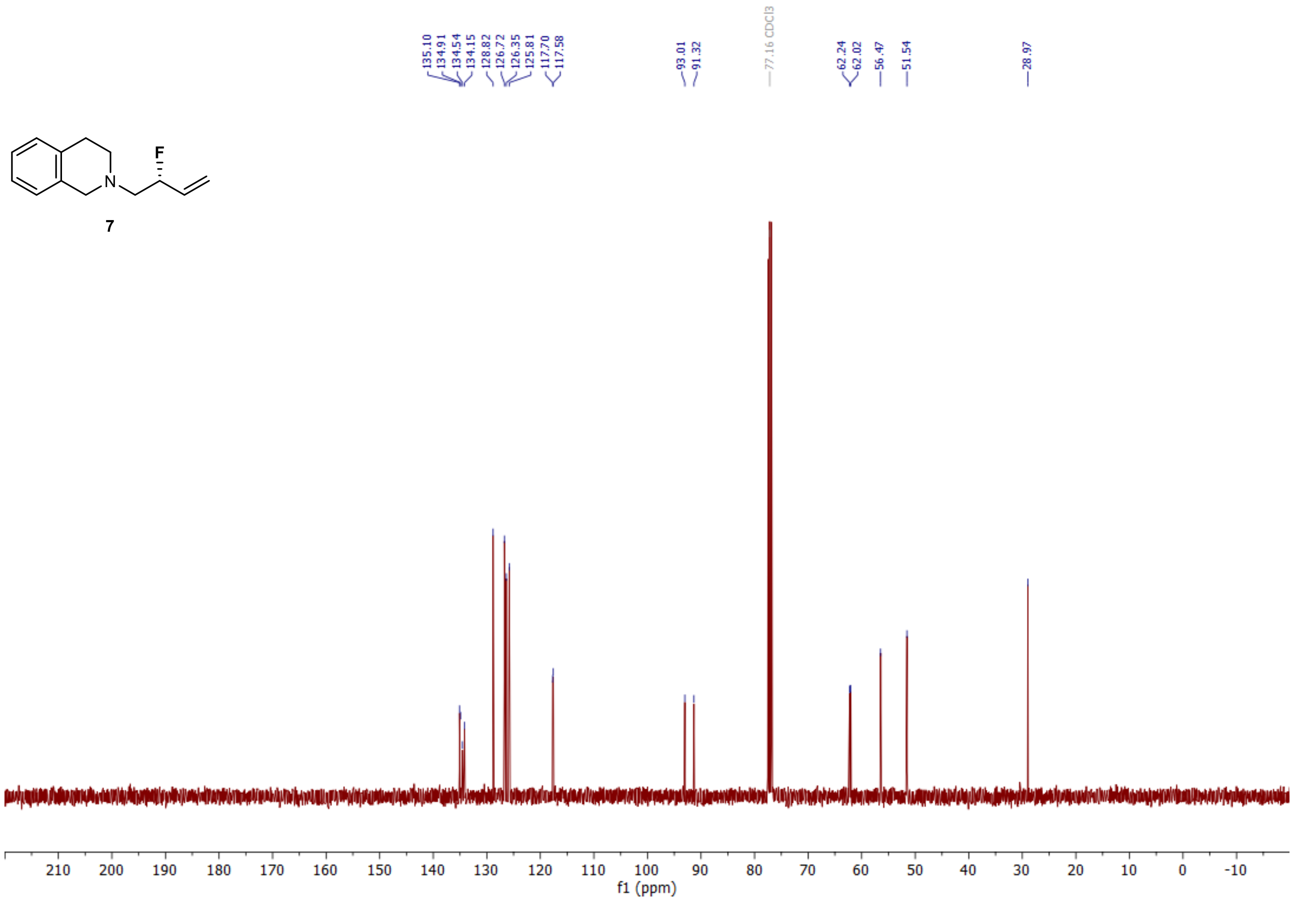

7 ^{19}F NMR (377 MHz, $CDCl_3$)

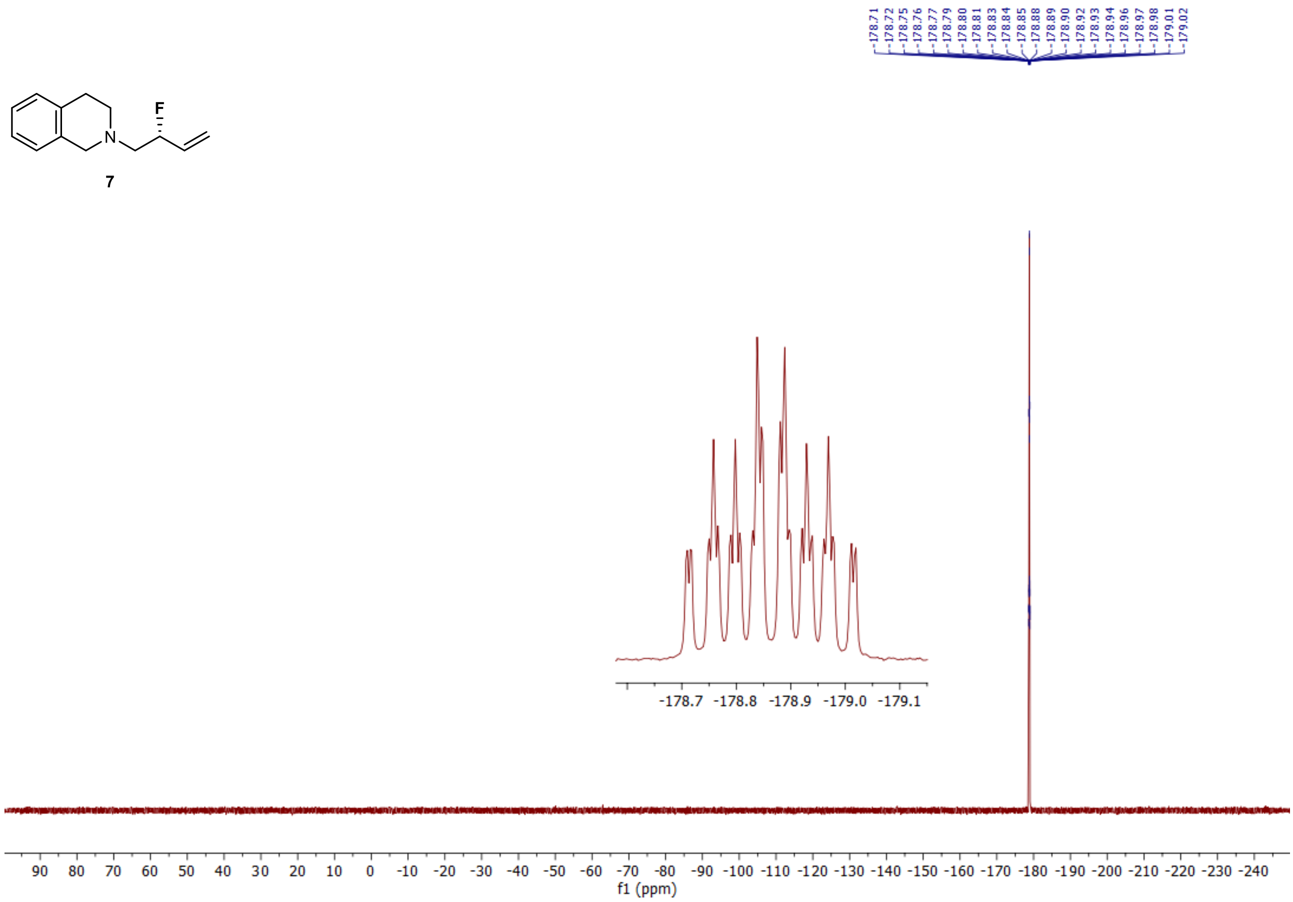

8 ^{1}H NMR (400 MHz, $CDCl_3$)

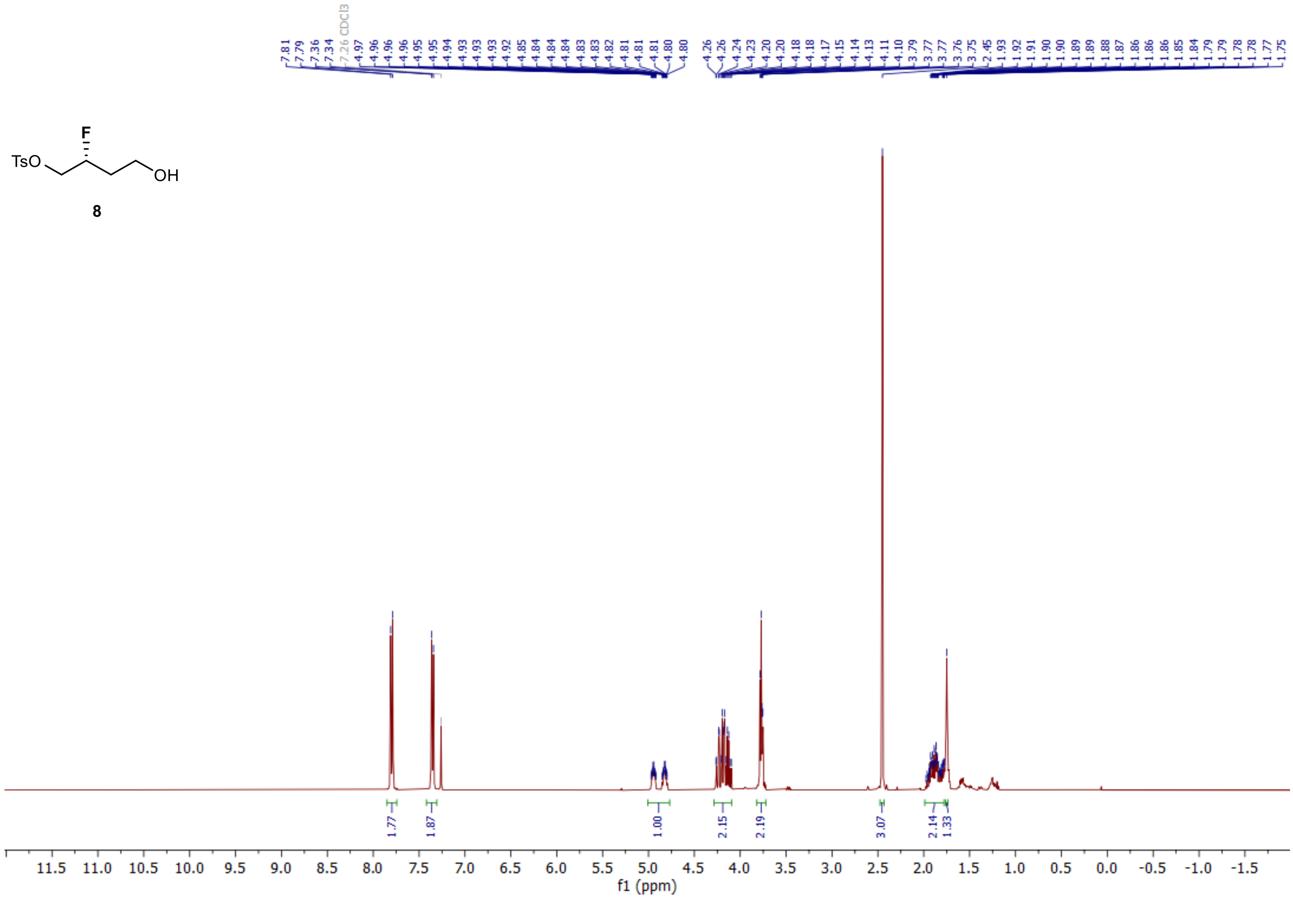

8 ^{13}C NMR (101 MHz, $CDCl_3$)

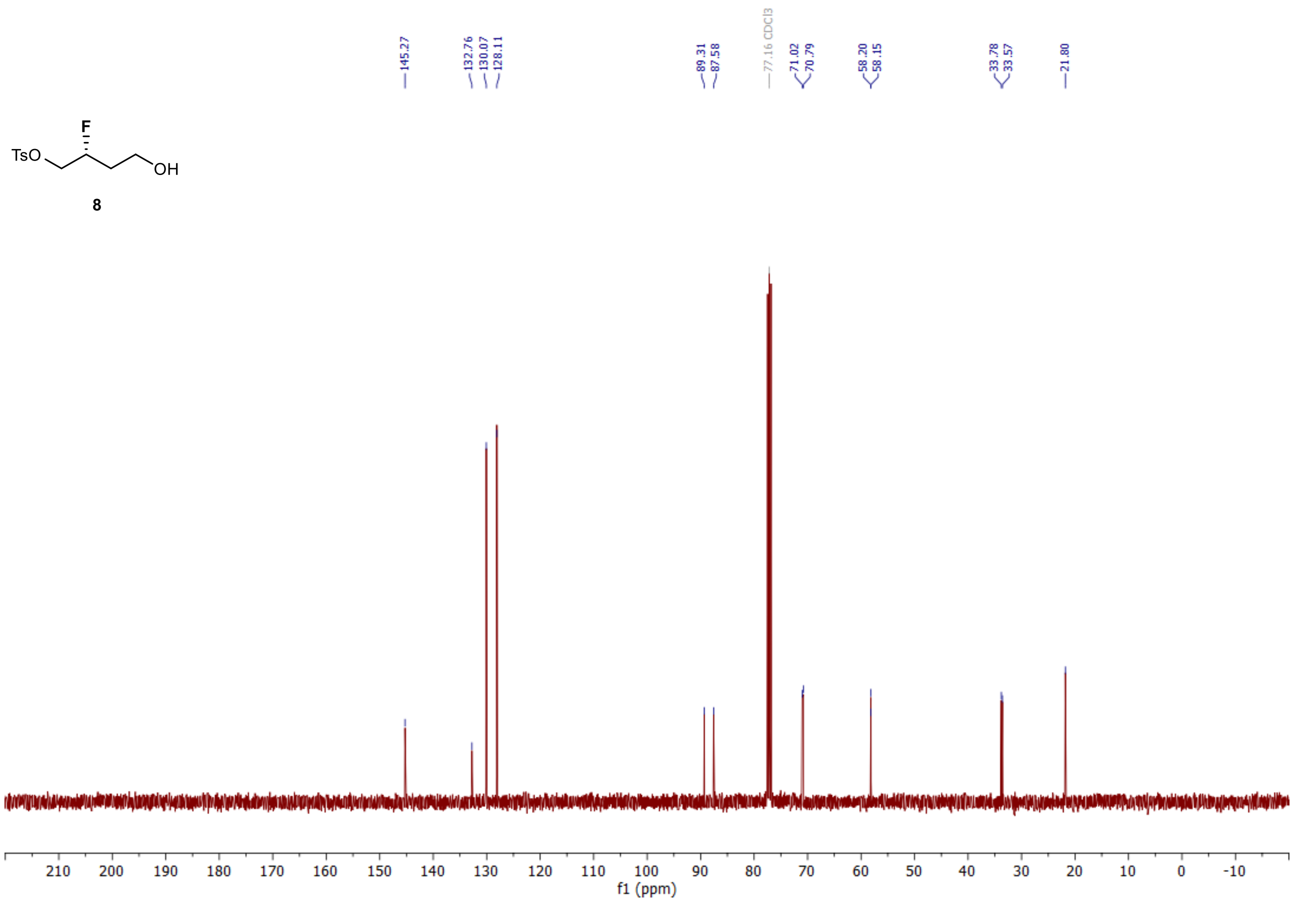

8 ^{19}F NMR (377 MHz, $CDCl_3$)

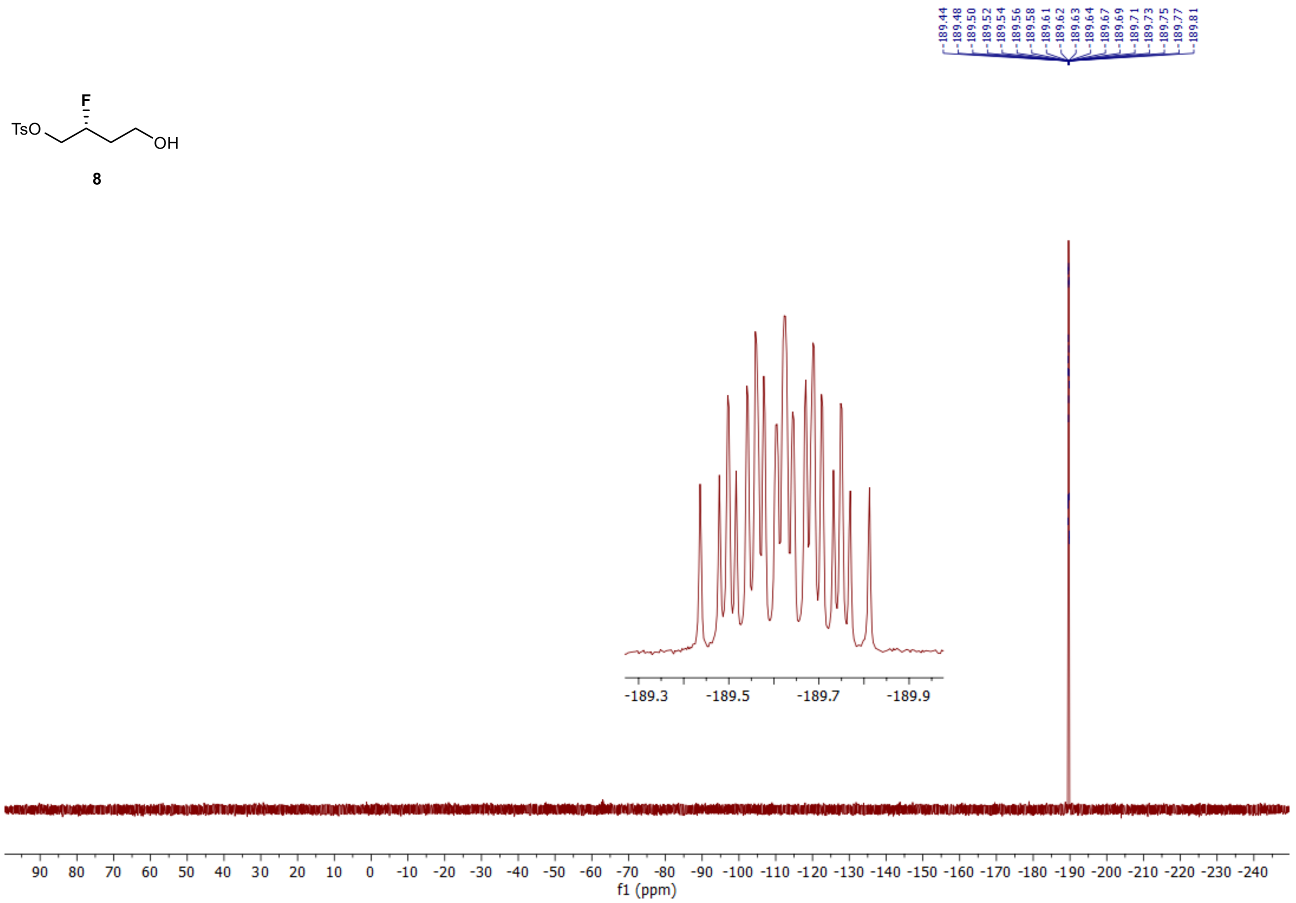

9 ^{1}H NMR (400 MHz, $CDCl_3$)

9

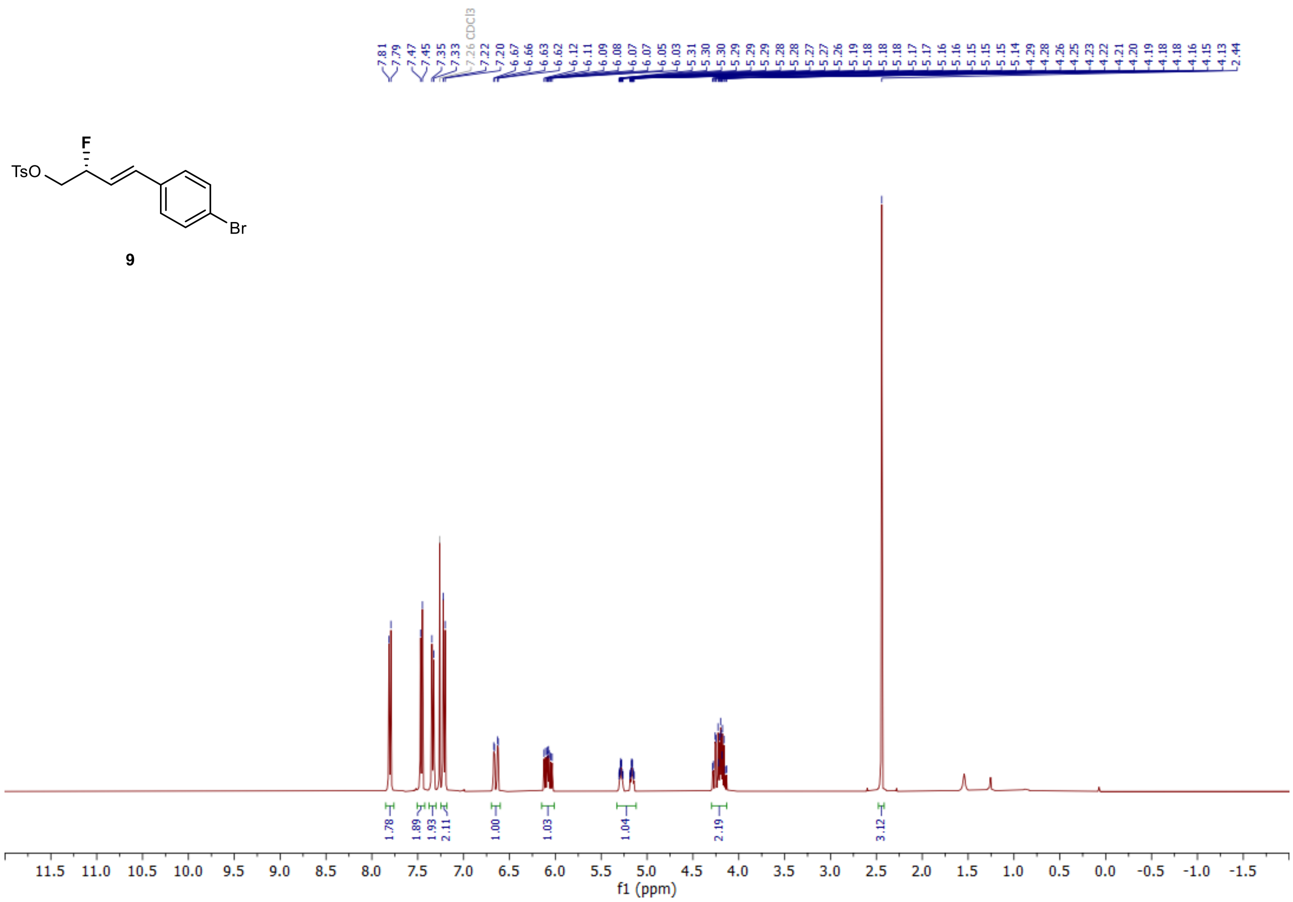

9 ^{13}C NMR (101 MHz, $CDCl_3$)

9

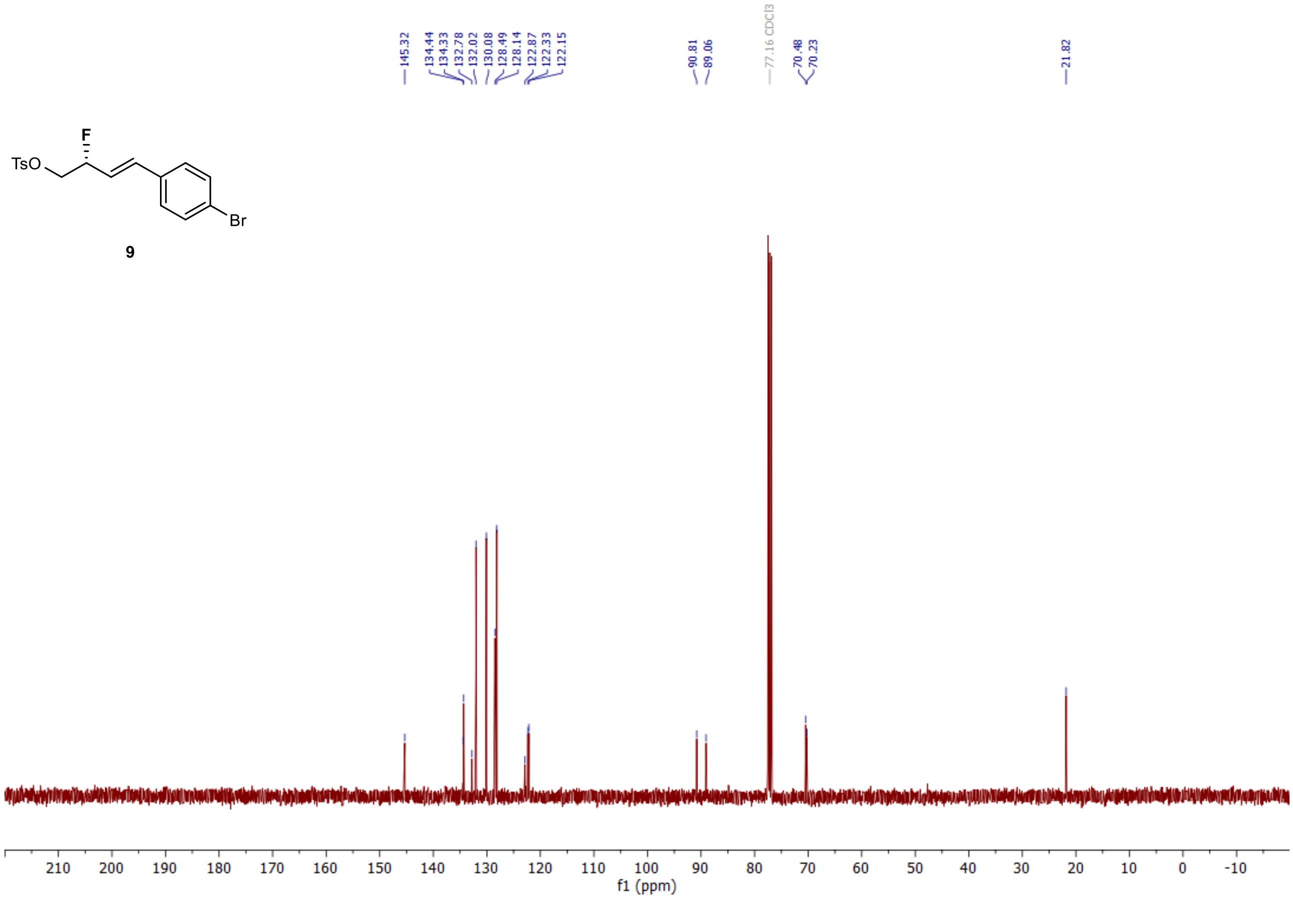

9 ^{19}F NMR (377 MHz, CDCl$_3$)

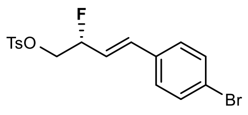

9

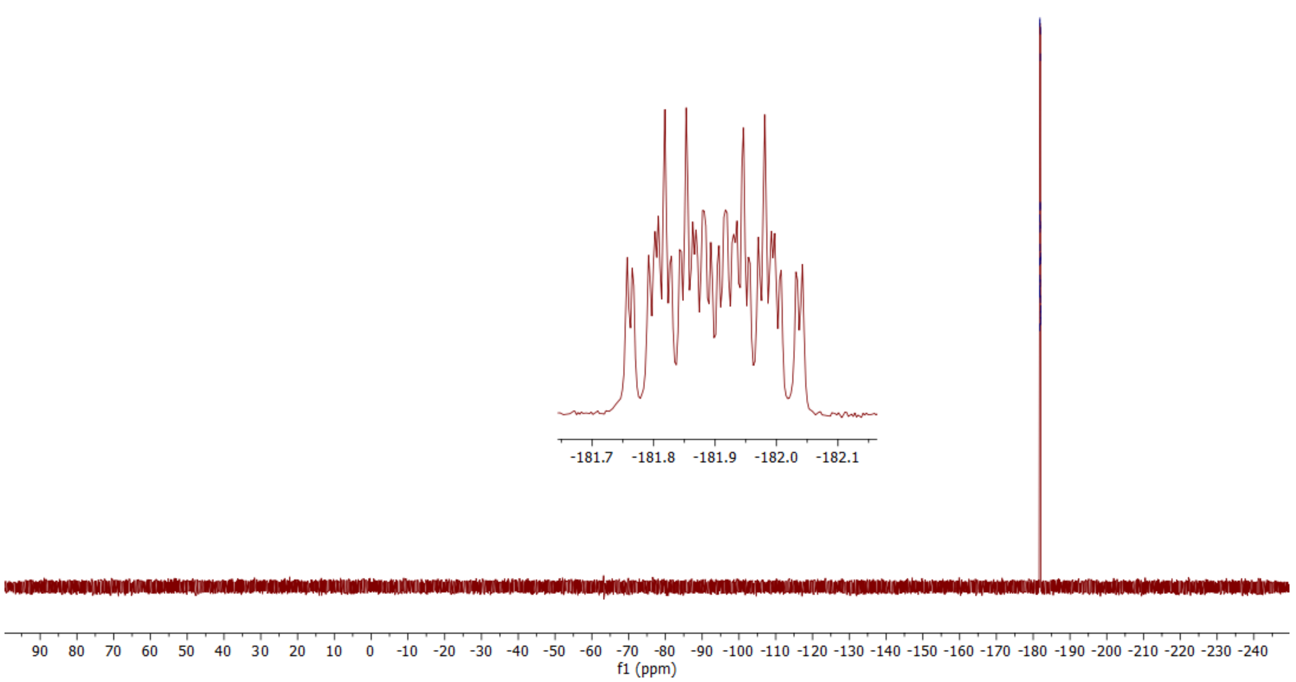

HPLC Traces

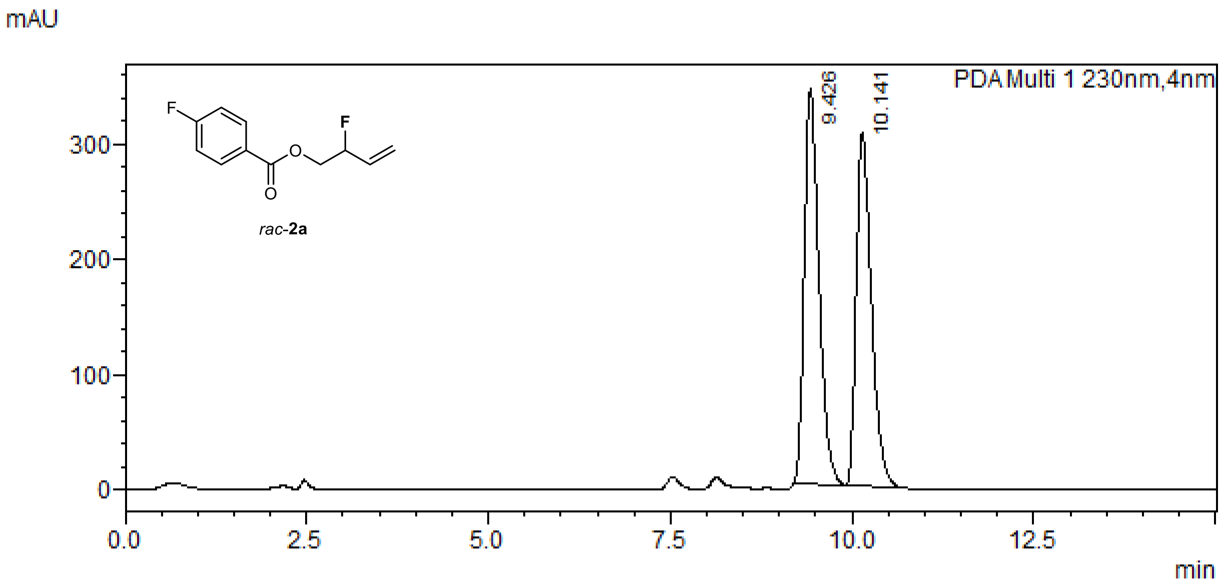

PDA Ch1 230nm

Peak#	Ret. Time	Area%
1	9.426	51.815
2	10.141	48.185
Total		100.000

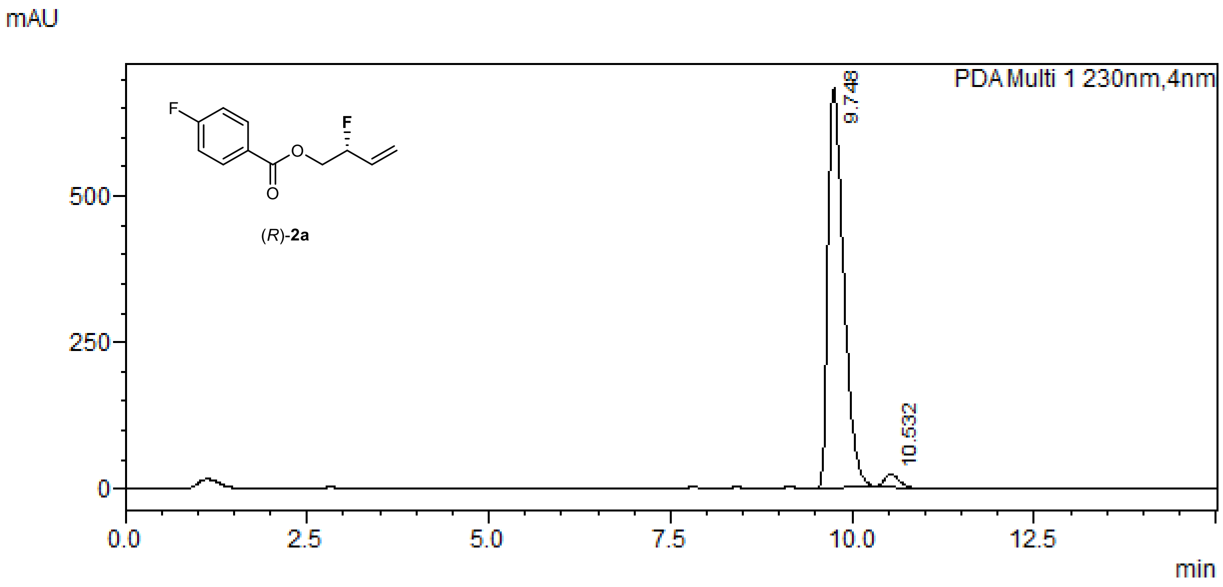

PDA Ch1 230nm

Peak#	Ret. Time	Area%
1	9.748	97.150
2	10.532	2.850
Total		100.000

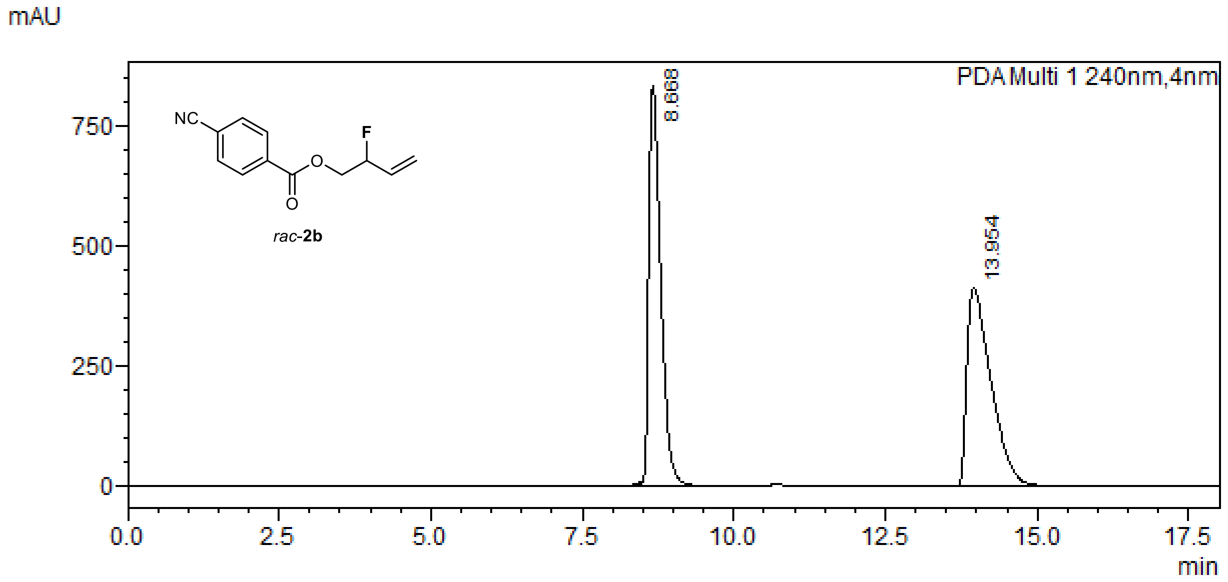

PDA Ch1 240nm

Peak#	Ret. Time	Area%
1	8.668	49.953
2	13.954	50.047
Total		100.000

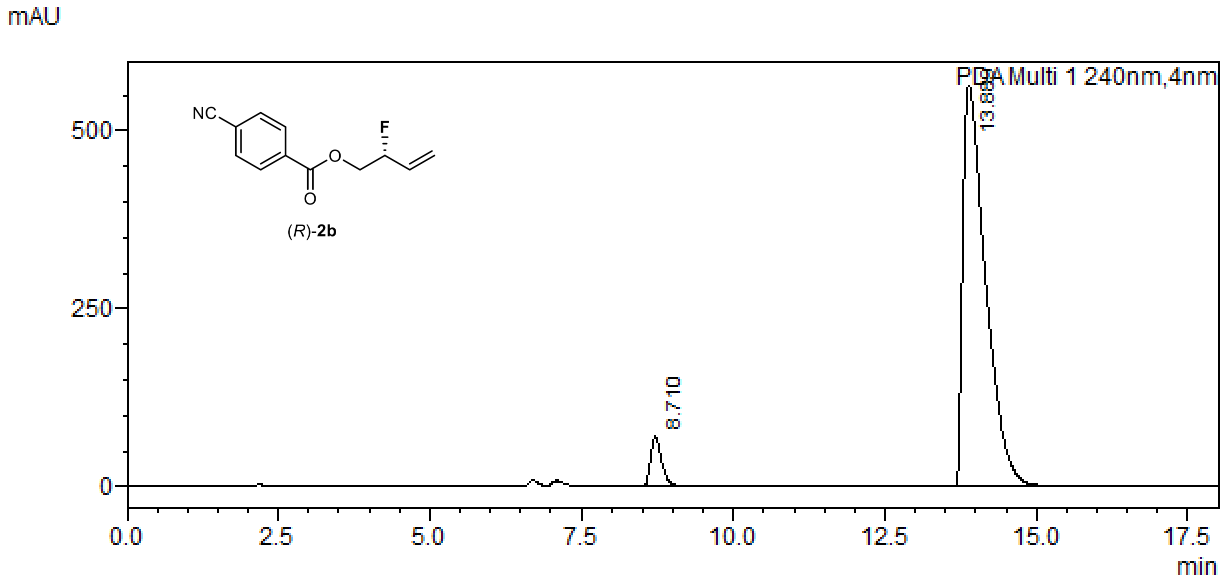

PDA Ch1 240nm

Peak#	Ret. Time	Area%
1	8.710	5.239
2	13.889	94.761
Total		100.000

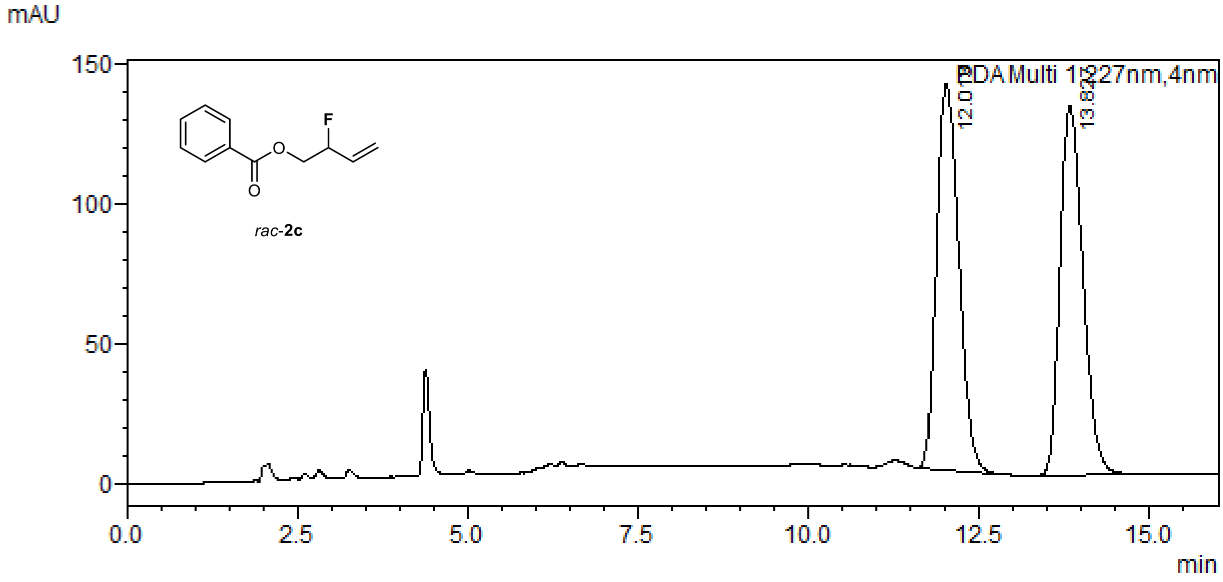

PDA Ch1 227nm

Peak#	Ret. Time	Area%
1	12.013	50.275
2	13.827	49.725
Total		100.000

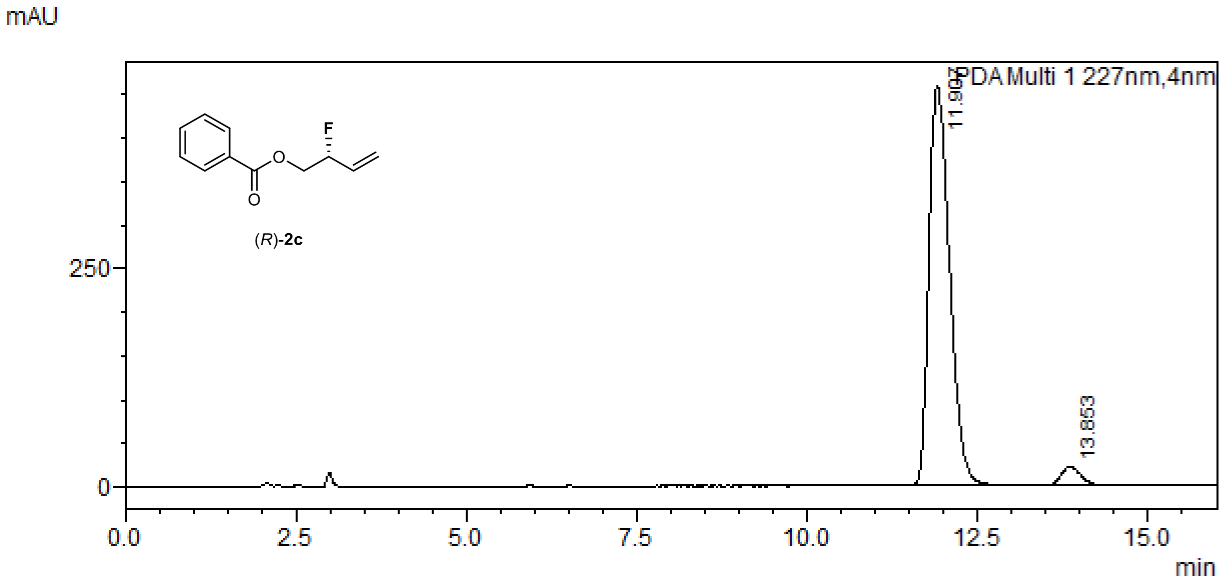

PDA Ch1 227nm

Peak#	Ret. Time	Area%
1	11.907	95.895
2	13.853	4.105
Total		100.000

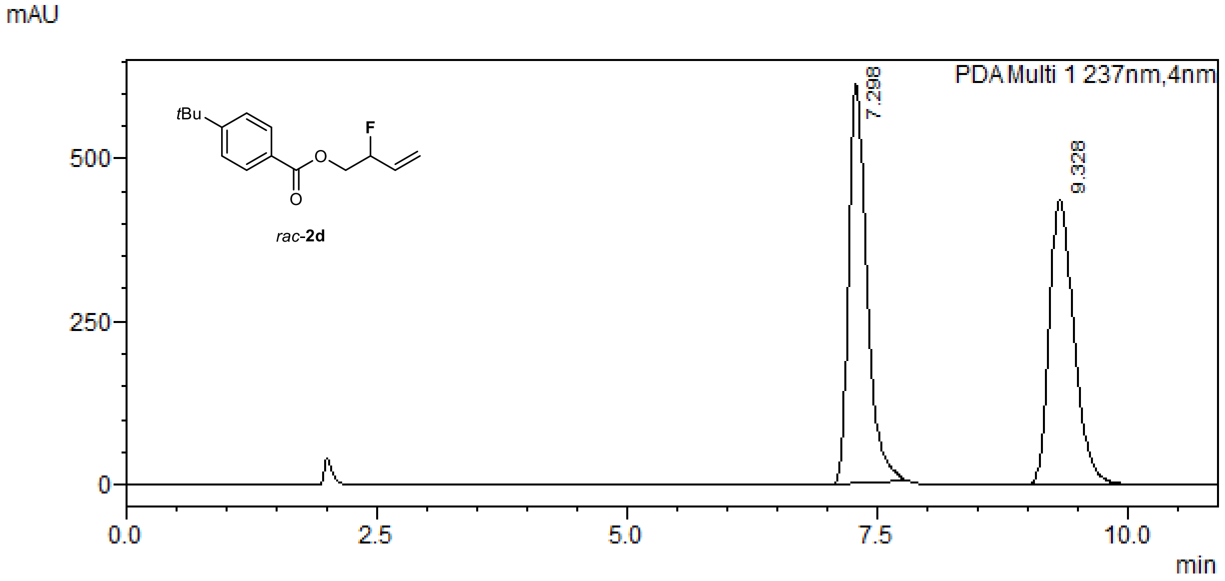

PDA Ch1 237nm

Peak#	Ret. Time	Area%
1	7.298	51.101
2	9.328	48.899
Total		100.000

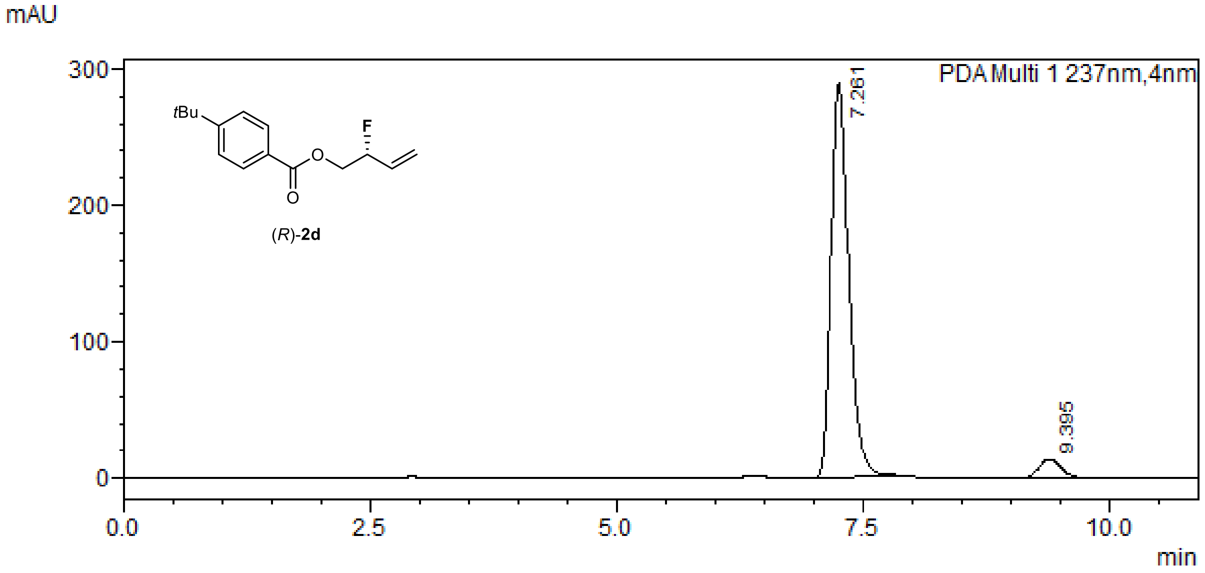

PDA Ch1 237nm

Peak#	Ret. Time	Area%
1	7.261	94.850
2	9.395	5.150
Total		100.000

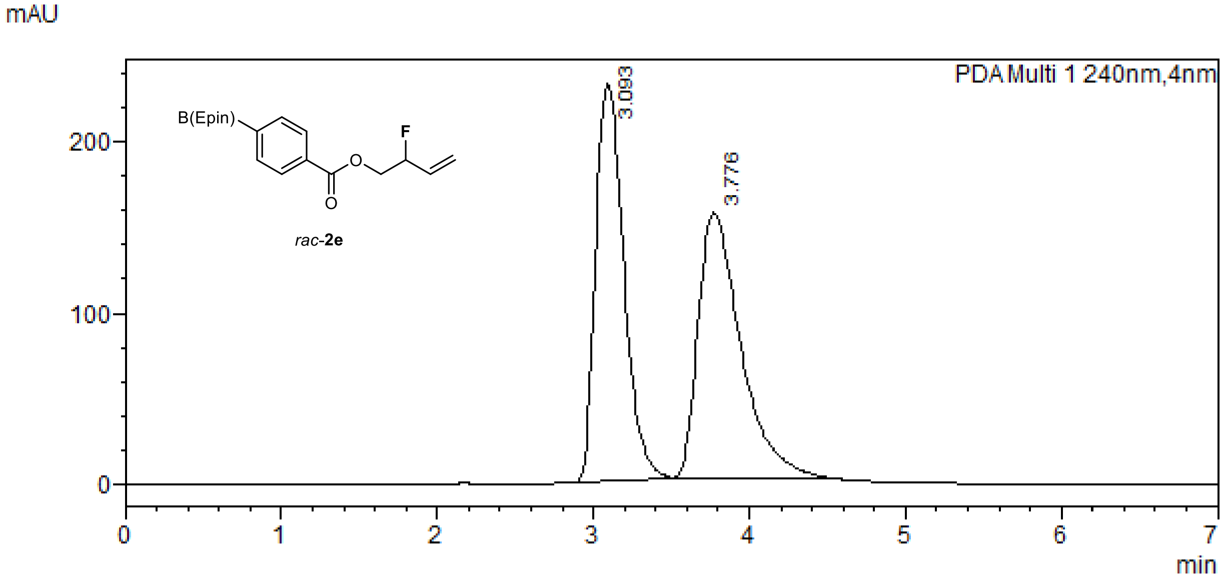

PDA Ch1 240nm

Peak#	Ret. Time	Area%
1	3.093	49.721
2	3.776	50.279
Total		100.000

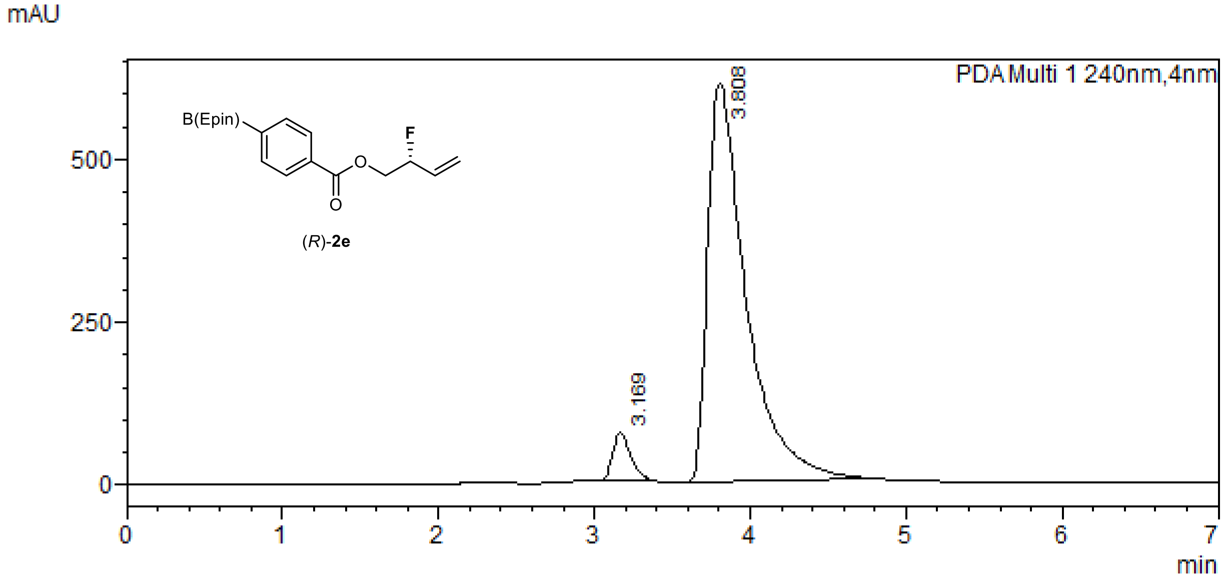

PDA Ch1 240nm

Peak#	Ret. Time	Area%
1	3.169	5.175
2	3.808	94.825
Total		100.000

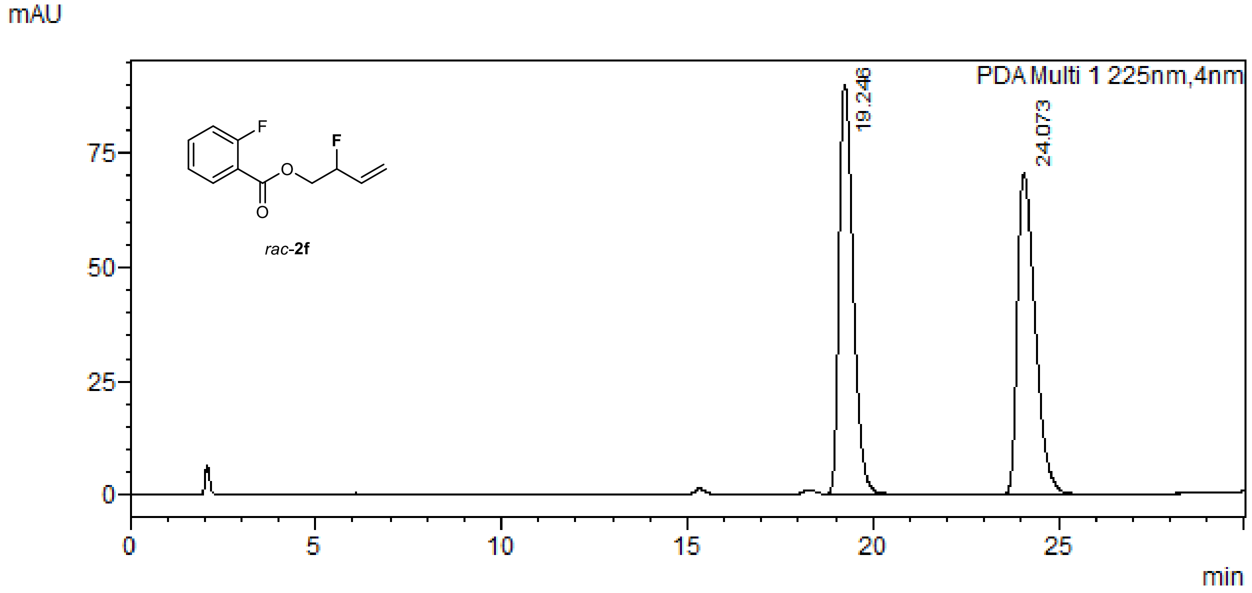

PDA Ch1 225nm

Peak#	Ret. Time	Area%
1	19.246	49.933
2	24.073	50.067
Total		100.000

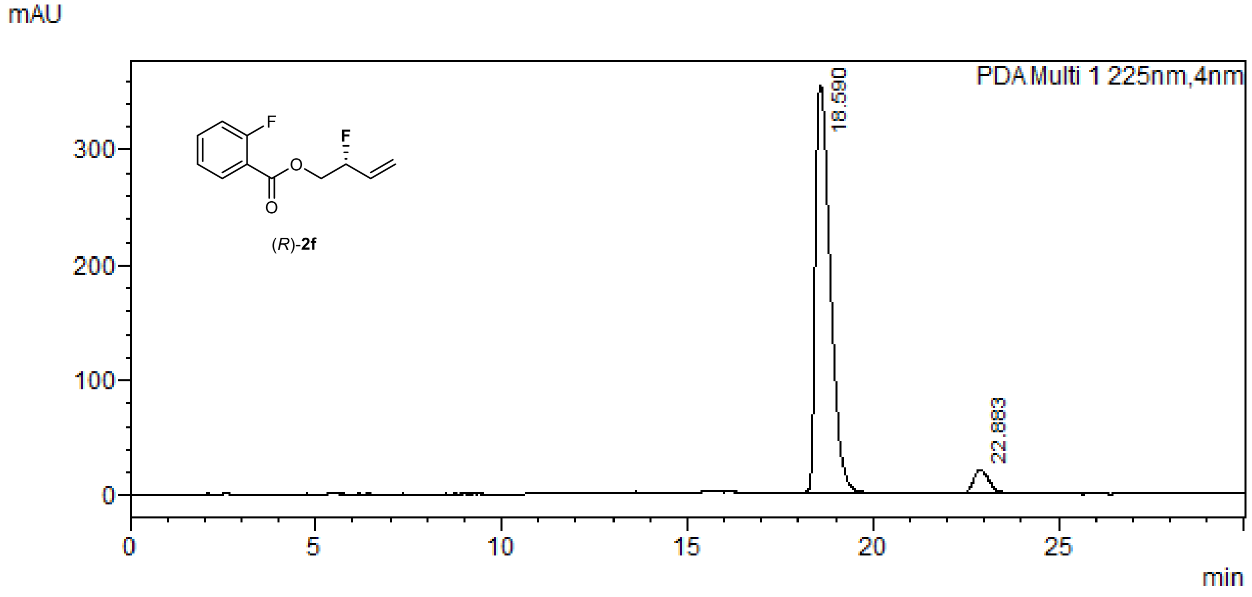

PDA Ch1 225nm

Peak#	Ret. Time	Area%
1	18.590	94.910
2	22.883	5.090
Total		100.000

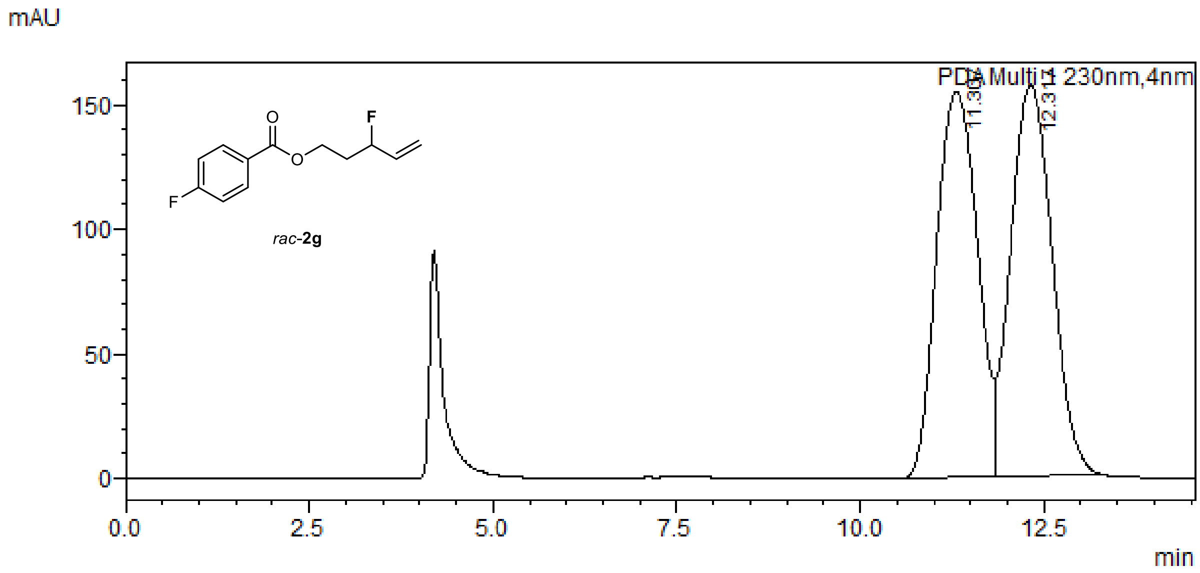

PDA Ch1 230nm

Peak#	Ret. Time	Area%
1	11.307	49.115
2	12.317	50.885
Total		100.000

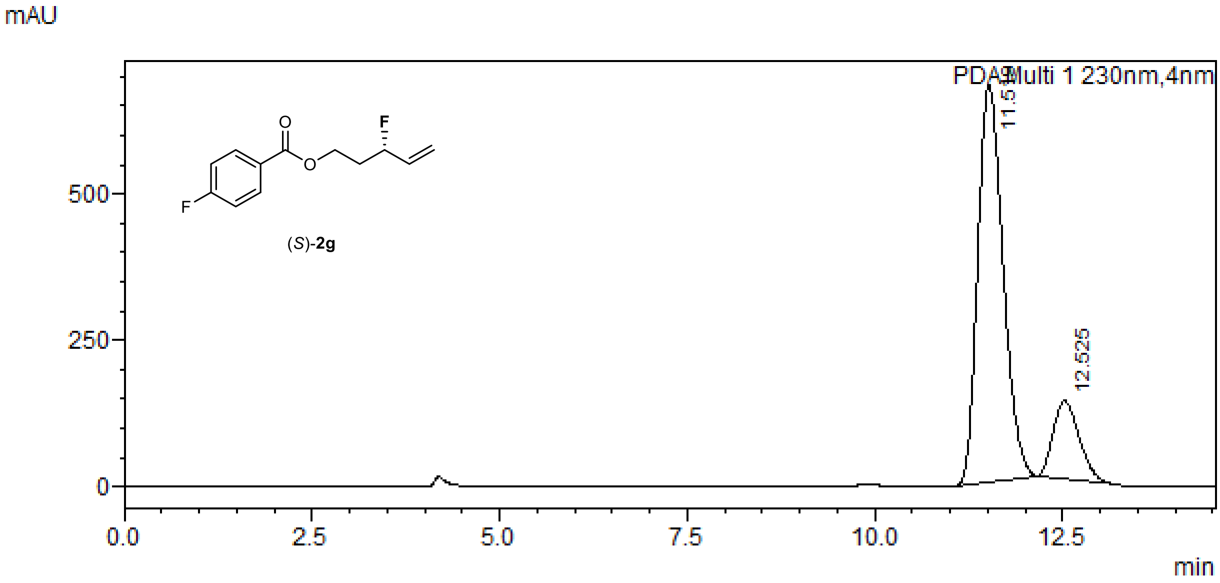

PDA Ch1 230nm

Peak#	Ret. Time	Area%
1	11.519	83.197
2	12.525	16.803
Total		100.000

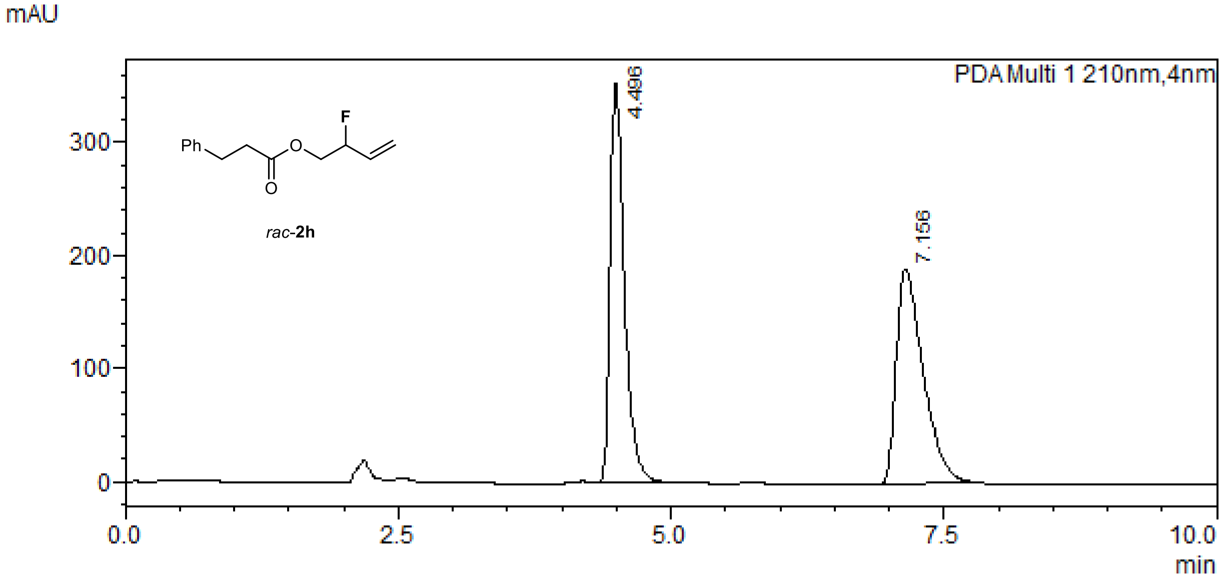

PDA Ch1 210nm

Peak#	Ret. Time	Area%
1	4.496	49.955
2	7.156	50.045
Total		100.000

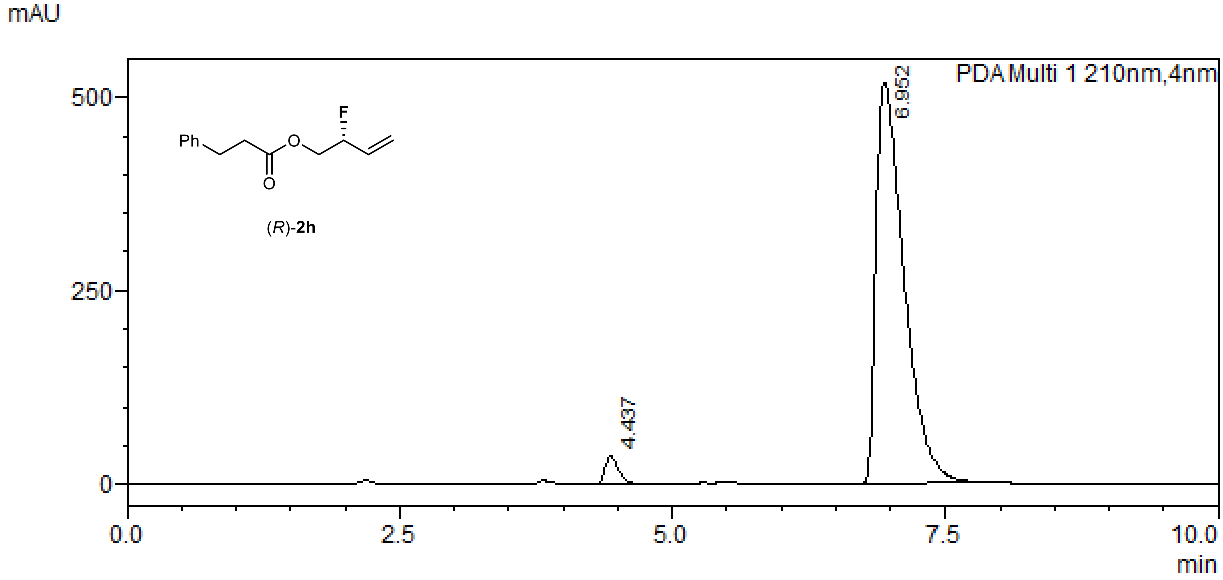

PDA Ch1 210nm

Peak#	Ret. Time	Area%
1	4.437	3.131
2	6.952	96.869
Total		100.000

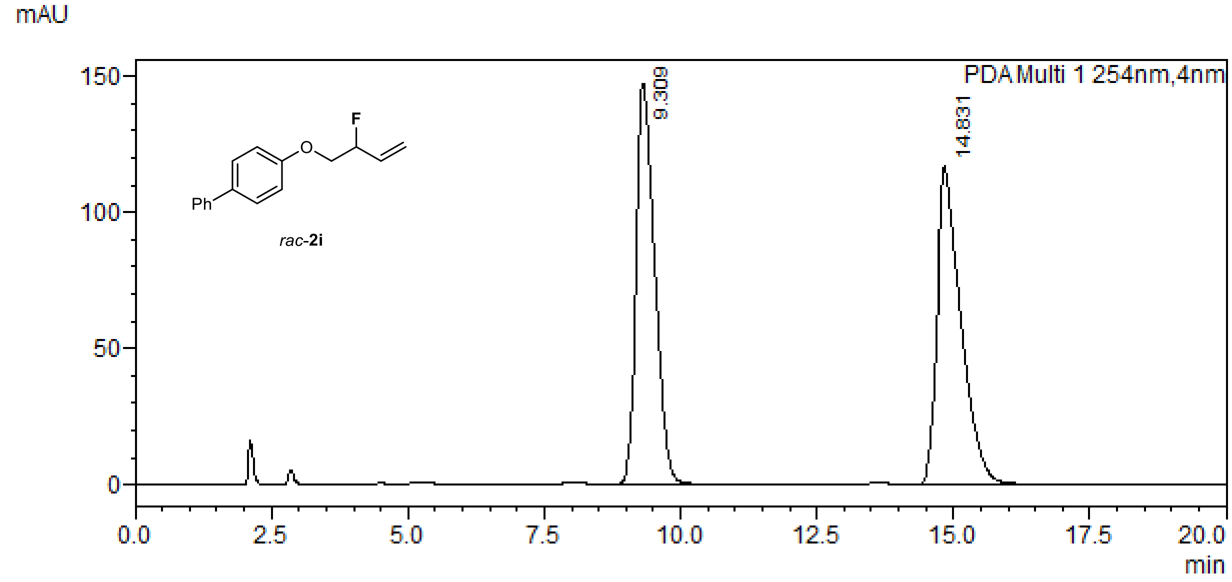

PDA Ch1 254nm

Peak#	Ret. Time	Area%
1	9.309	49.940
2	14.831	50.060
Total		100.000

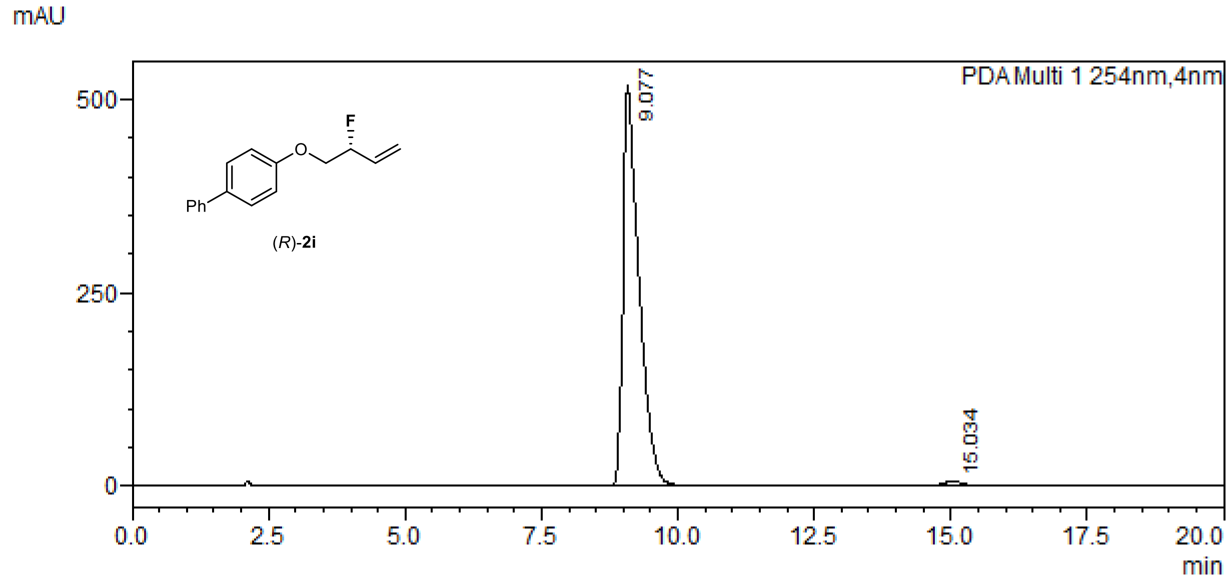

PDA Ch1 254nm

Peak#	Ret. Time	Area%
1	9.077	98.810
2	15.034	1.190
Total		100.000

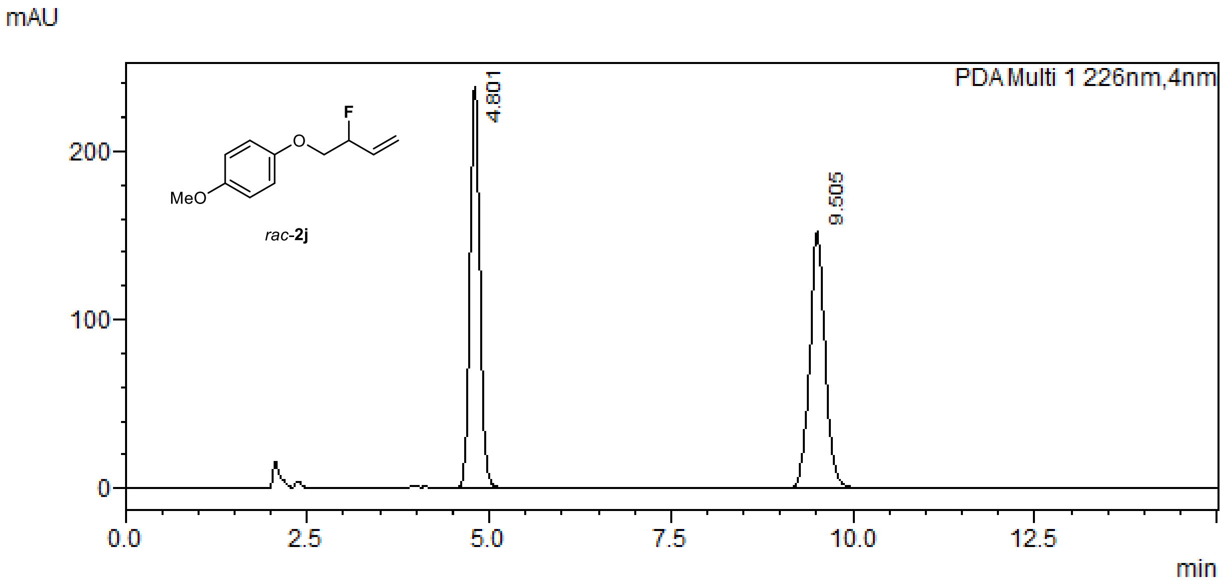

PDA Ch1 226nm

Peak#	Ret. Time	Area%
1	4.801	49.909
2	9.505	50.091
Total		100.000

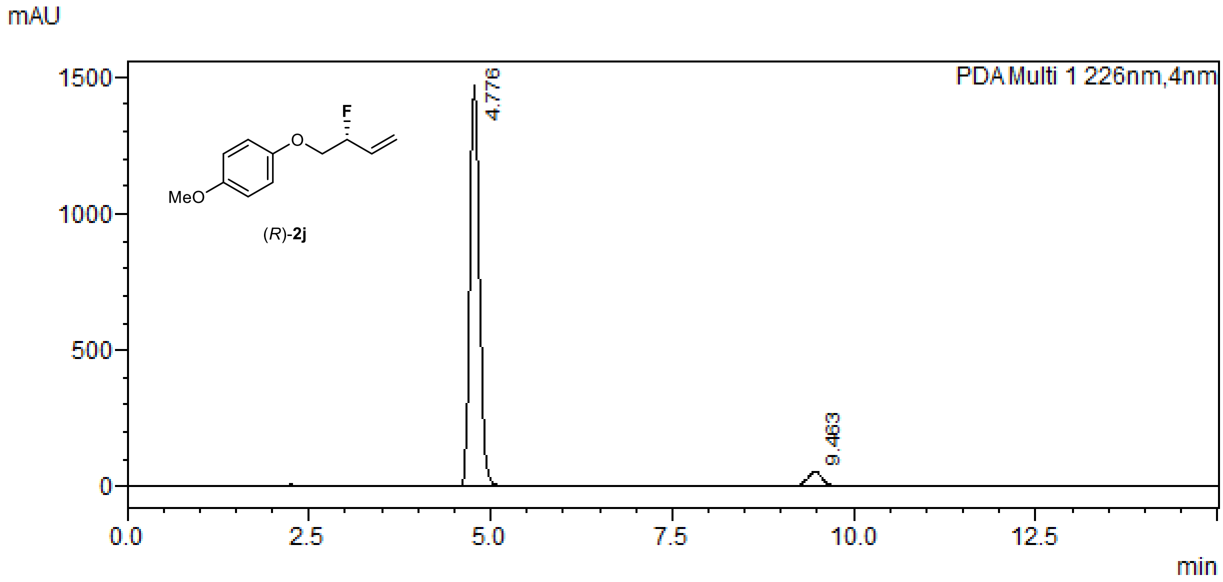

PDA Ch1 226nm

Peak#	Ret. Time	Area%
1	4.776	94.834
2	9.463	5.166
Total		100.000

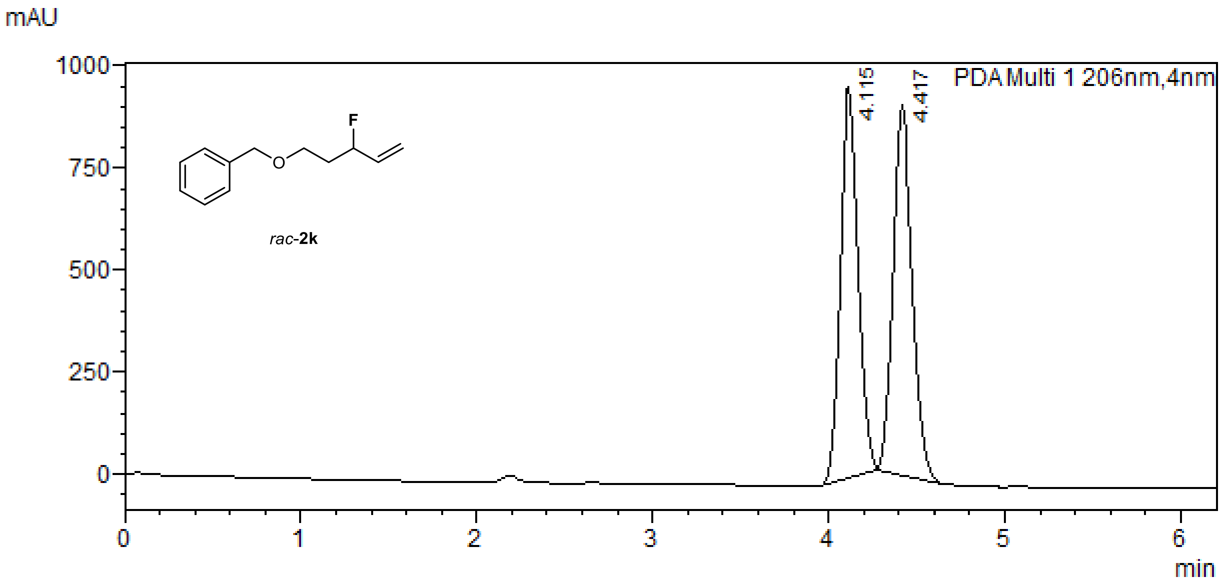

PDA Ch1 206nm

Peak#	Ret. Time	Area%
1	4.115	49.783
2	4.417	50.217
Total		100.000

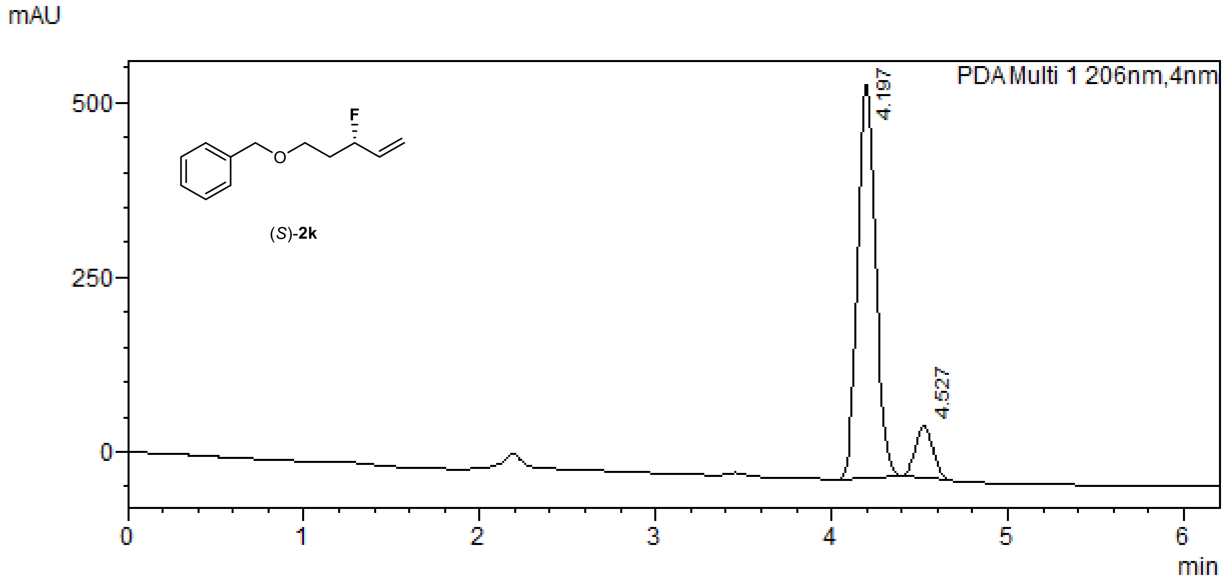

PDA Ch1 206nm

Peak#	Ret. Time	Area%
1	4.197	88.944
2	4.527	11.056
Total		100.000

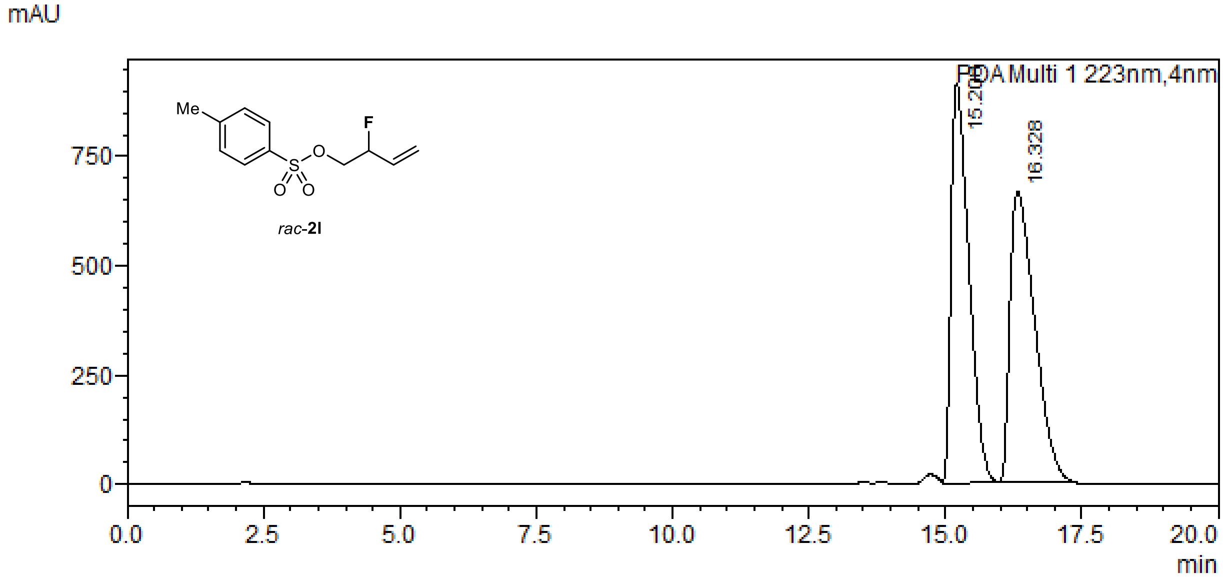

PDA Ch1 223nm

Peak#	Ret. Time	Area%
1	15.208	49.787
2	16.328	50.213
Total		100.000

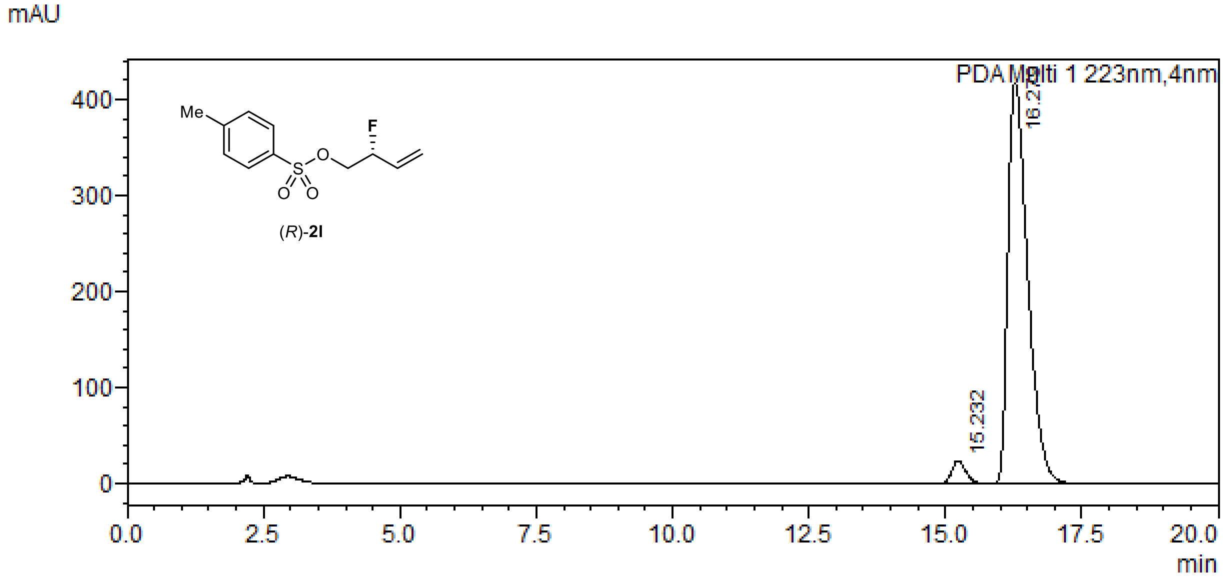

PDA Ch1 223nm

Peak#	Ret. Time	Area%
1	15.232	3.995
2	16.279	96.005
Total		100.000

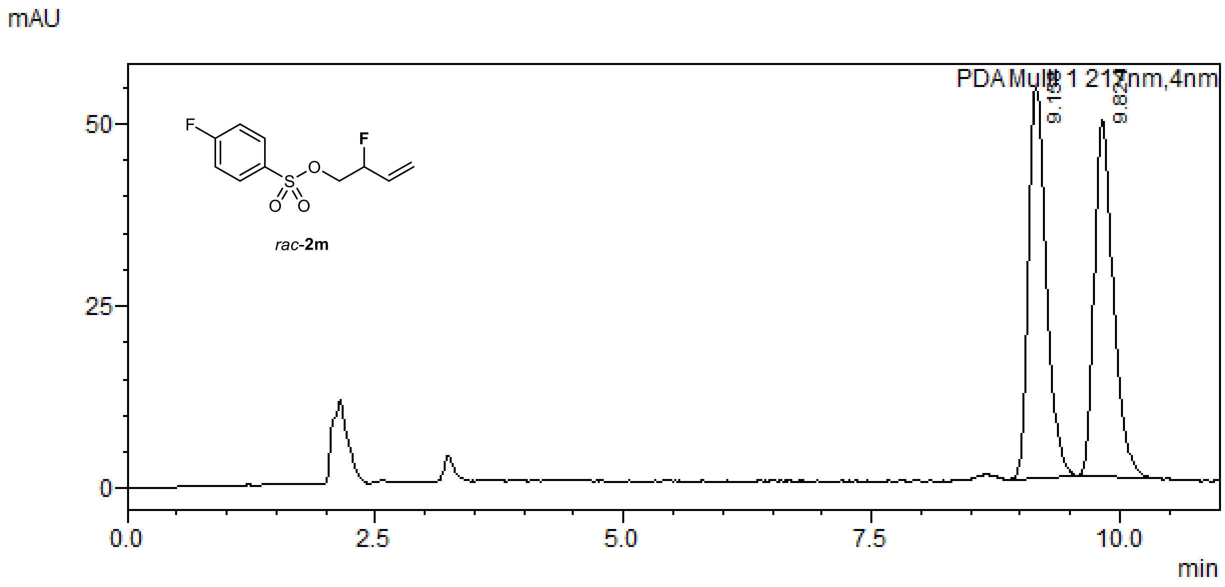

PDA Ch1 217nm

Peak#	Ret. Time	Area%
1	9.158	50.072
2	9.824	49.928
Total		100.000

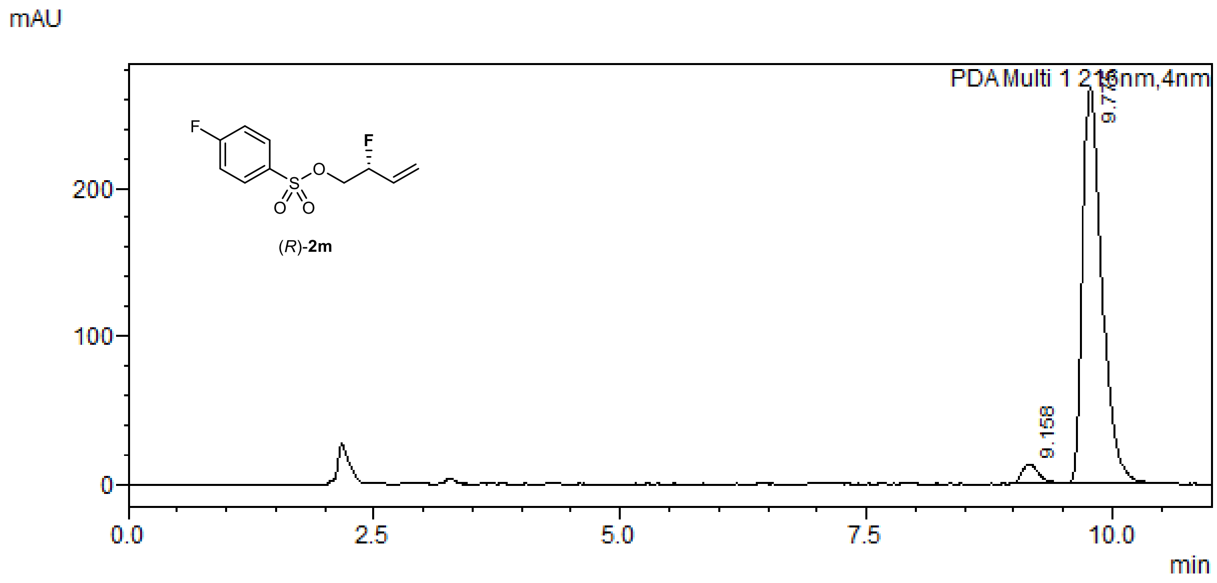

PDA Ch1 216nm

Peak#	Ret. Time	Area%
1	9.158	4.149
2	9.775	95.851
Total		100.000

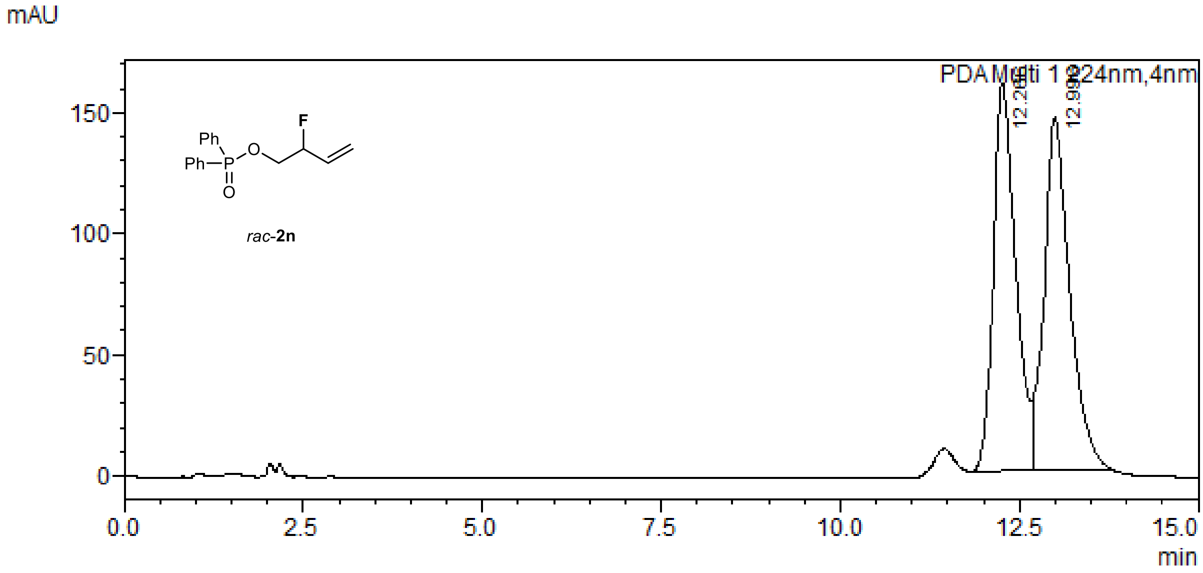

PDA Ch1 224nm

Peak#	Ret. Time	Area%
1	12.266	48.939
2	12.996	51.061
Total		100.000

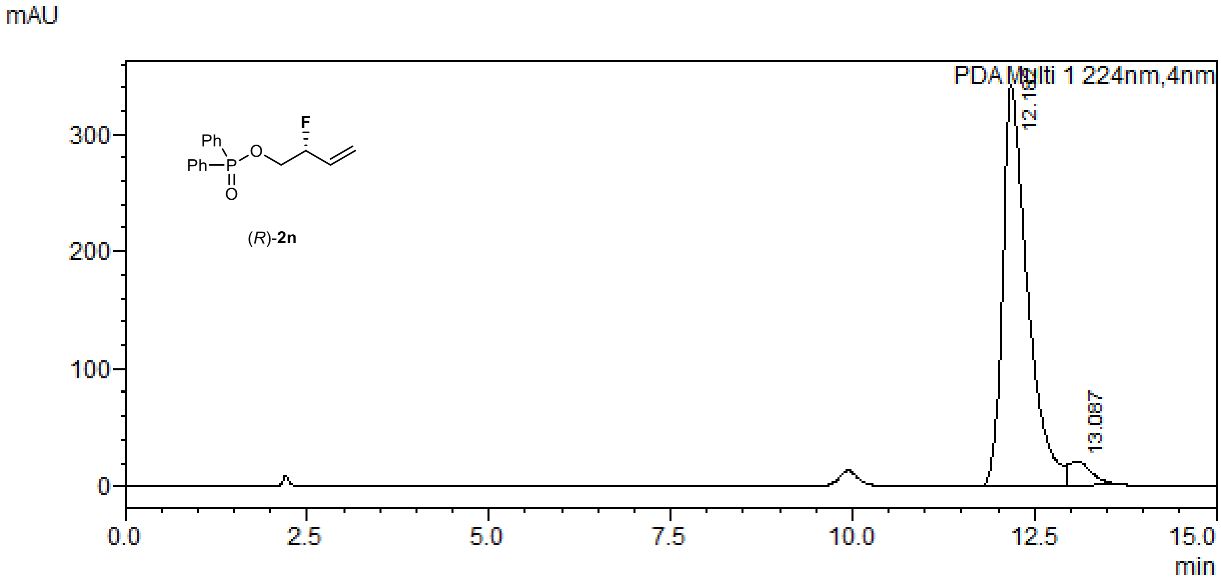

PDA Ch1 224nm

Peak#	Ret. Time	Area%
1	12.182	94.851
2	13.087	5.149
Total		100.000

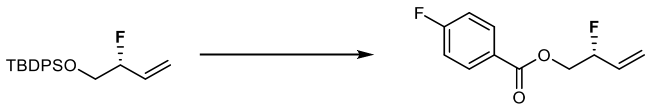

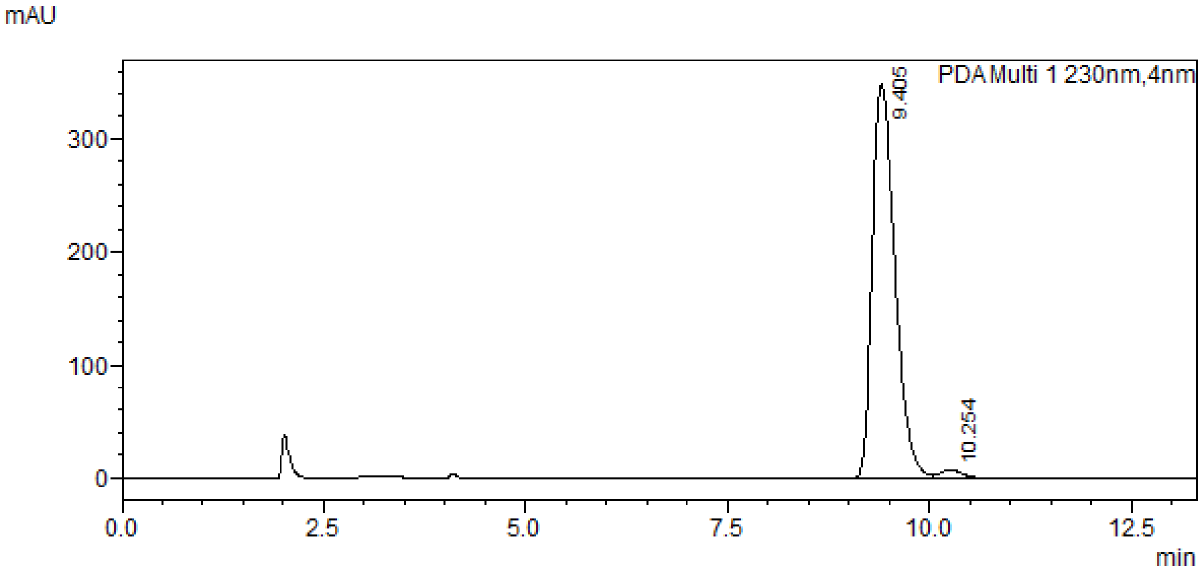

PDA Ch1 230nm

Peak#	Ret. Time	Area%
1	9.405	98.066
2	10.254	1.934
Total		100.000

The HPLC trace for *rac*-**2a** was reported above.

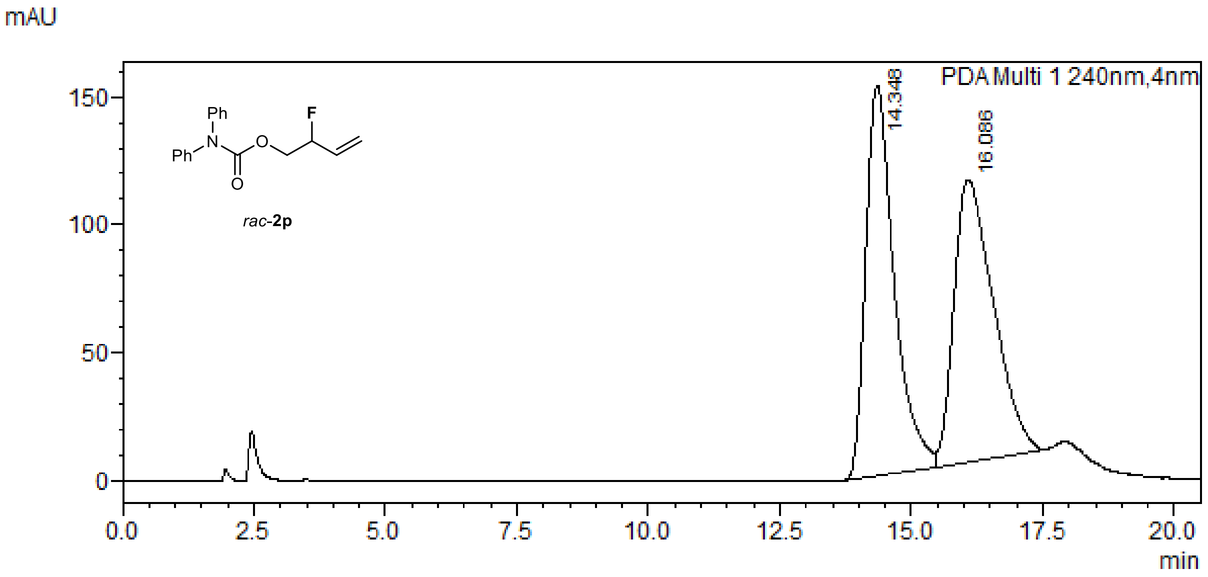

PDA Ch1 240nm

Peak#	Ret. Time	Area%
1	14.348	49.849
2	16.086	50.151
Total		100.000

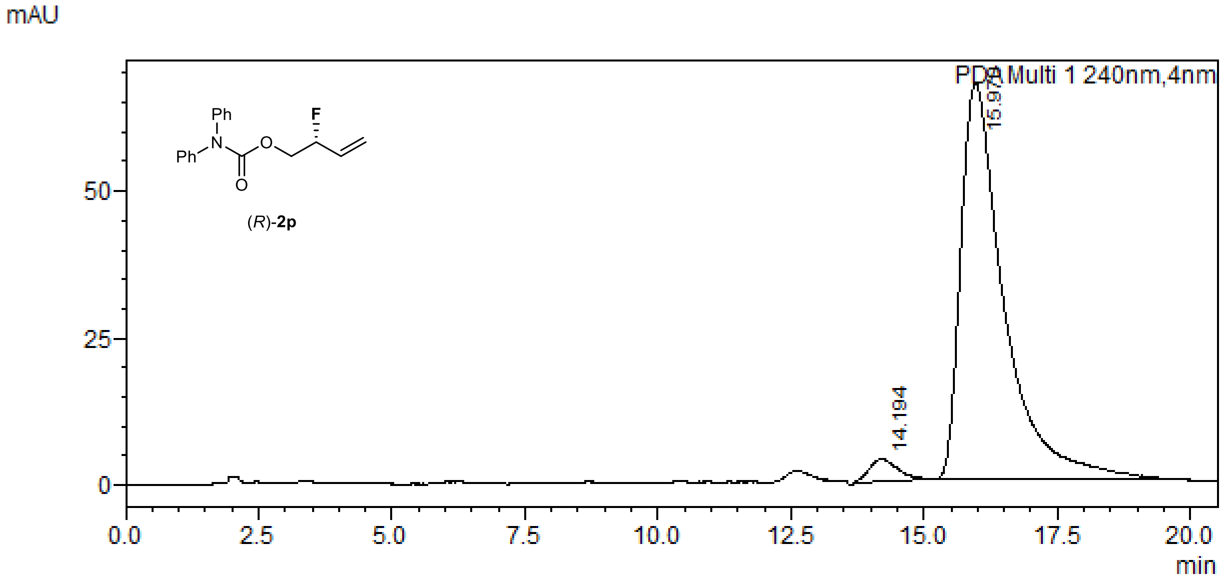

PDA Ch1 240nm

Peak#	Ret. Time	Area%
1	14.194	3.832
2	15.970	96.168
Total		100.000

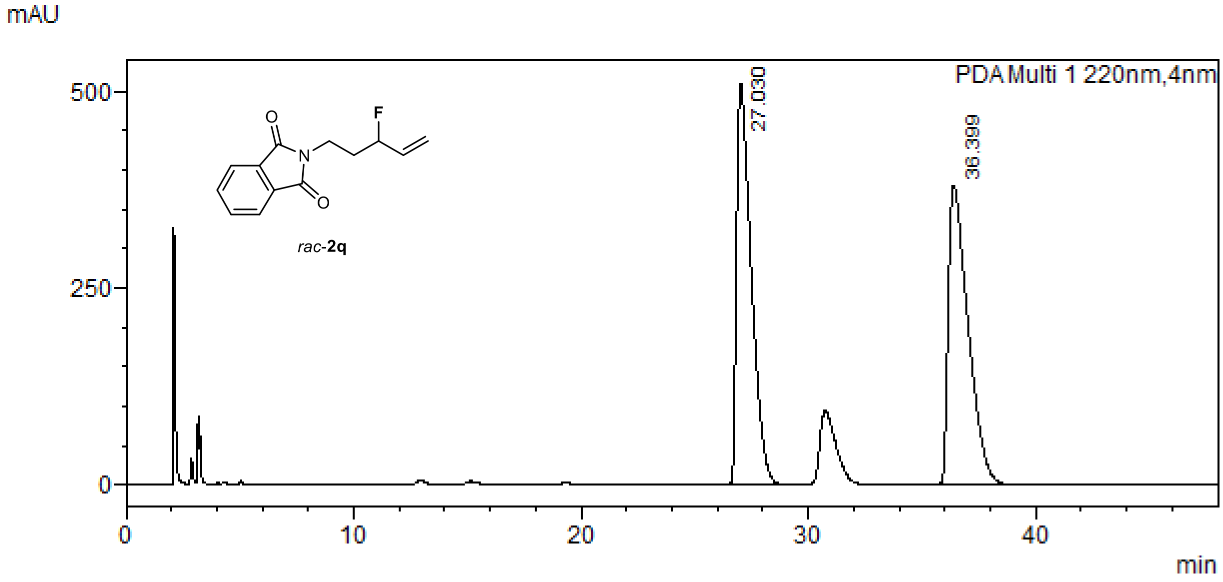

PDA Ch1 220nm

Peak#	Ret. Time	Area%
1	27.030	49.943
2	36.399	50.057
Total		100.000

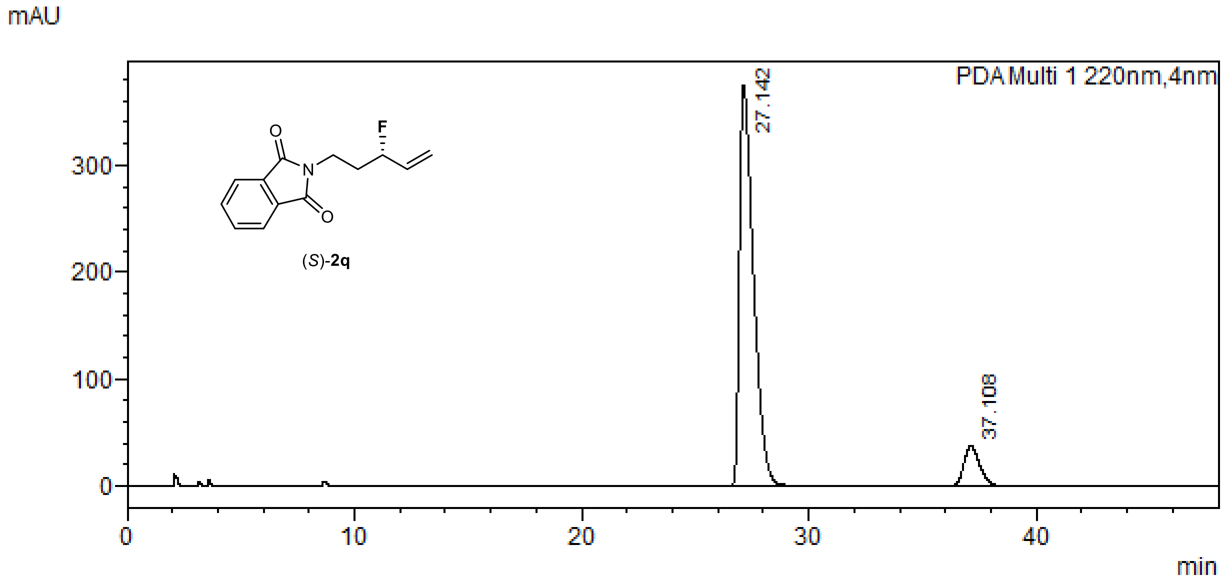

PDA Ch1 220nm

Peak#	Ret. Time	Area%
1	27.142	90.093
2	37.108	9.907
Total		100.000

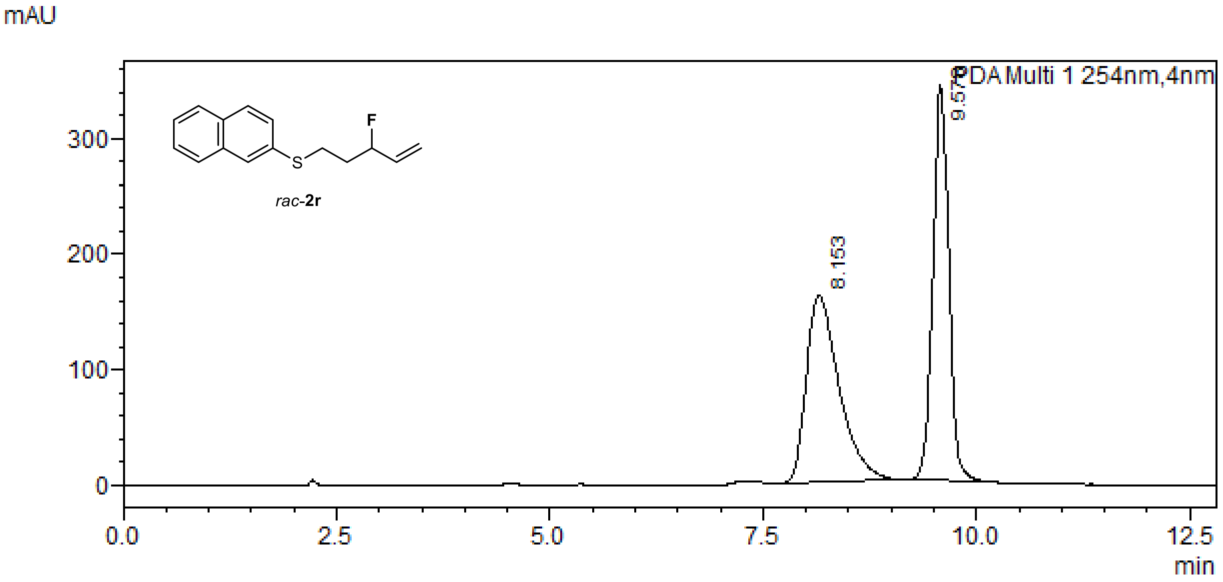

PDA Ch1 254nm

Peak#	Ret. Time	Area%
1	8.153	49.037
2	9.578	50.963
Total		100.000

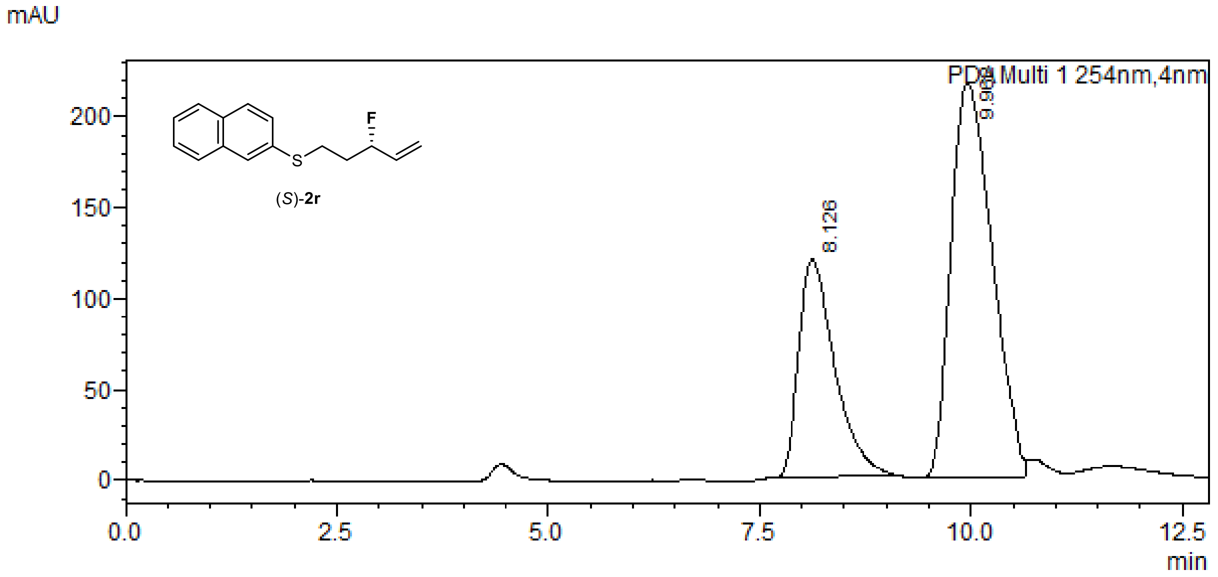

PDA Ch1 254nm

Peak#	Ret. Time	Area%
1	8.126	32.587
2	9.962	67.413
Total		100.000

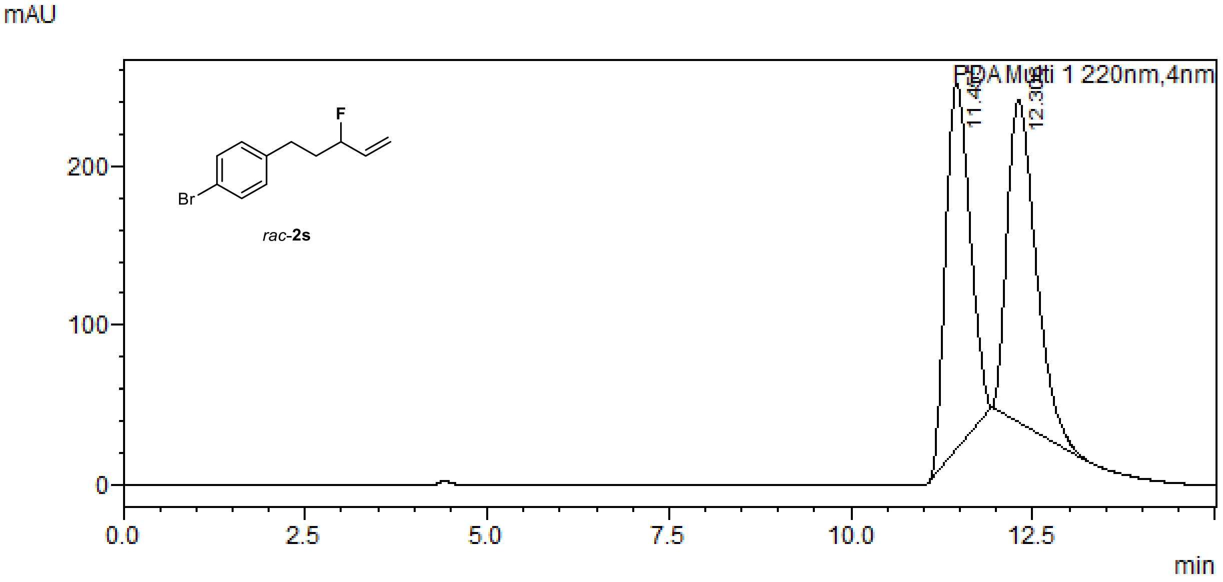

PDA Ch1 220nm

Peak#	Ret. Time	Area%
1	11.455	48.958
2	12.306	51.042
Total		100.000

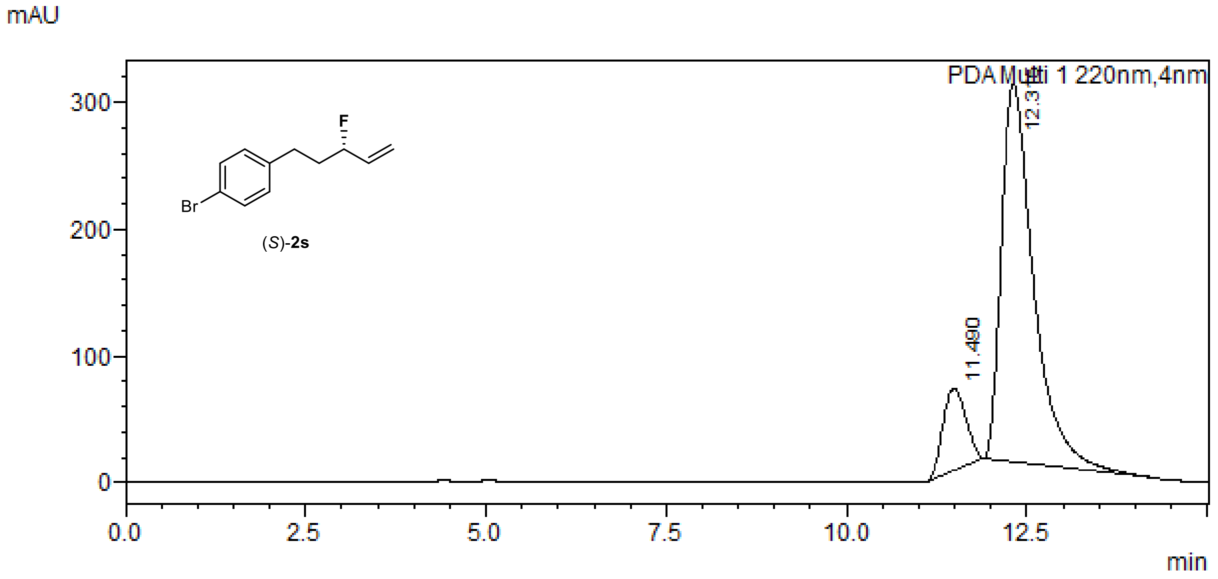

PDA Ch1 220nm

Peak#	Ret. Time	Area%
1	11.490	13.164
2	12.315	86.836
Total		100.000

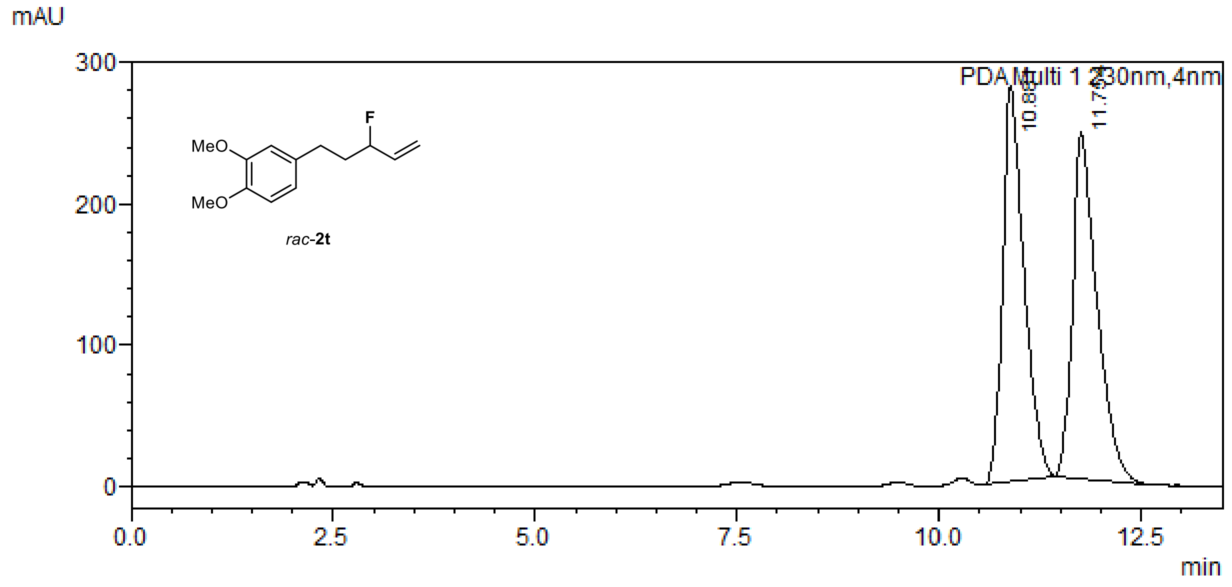

PDA Ch1 230nm

Peak#	Ret. Time	Area%
1	10.881	50.092
2	11.754	49.908
Total		100.000

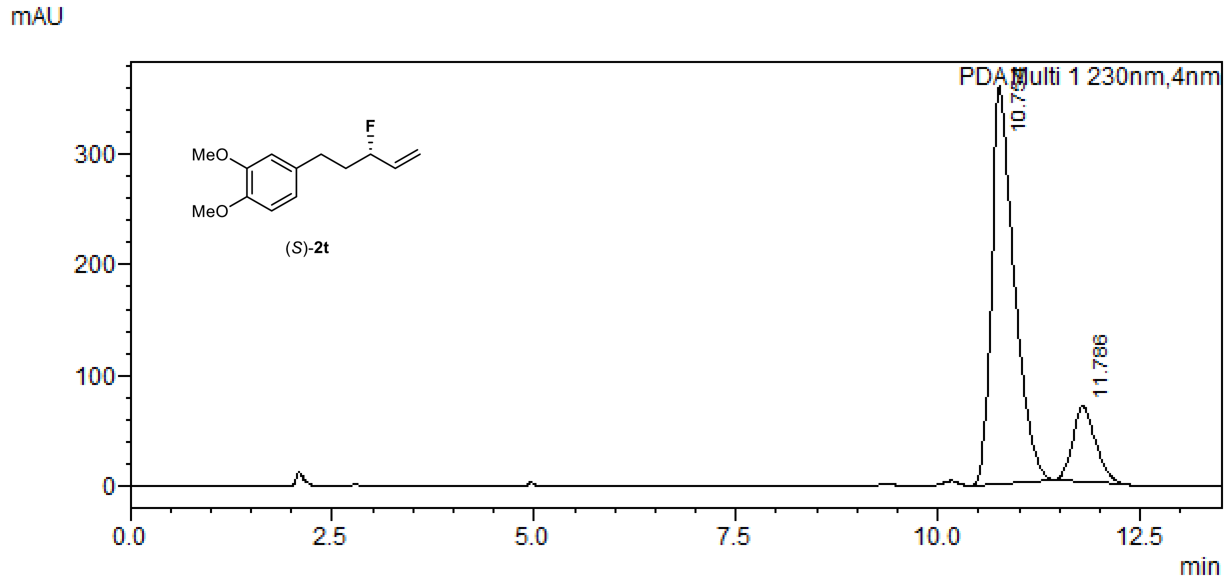

PDA Ch1 230nm

Peak#	Ret. Time	Area%
1	10.754	83.839
2	11.786	16.161
Total		100.000

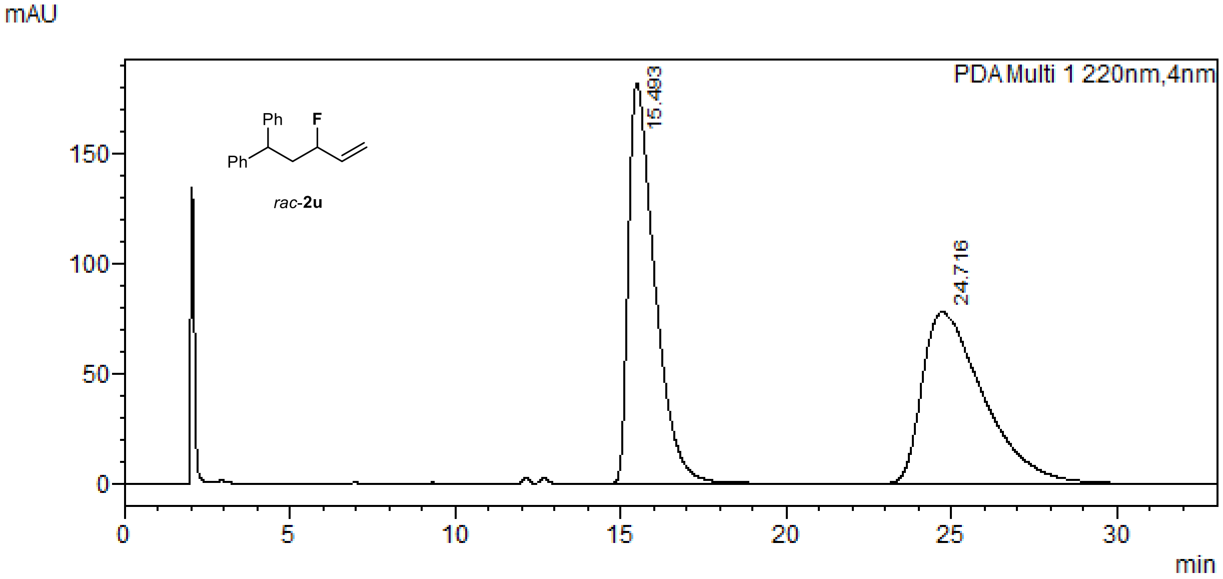

PDA Ch1 220nm

Peak#	Ret. Time	Area%
1	15.493	50.059
2	24.716	49.941
Total		100.000

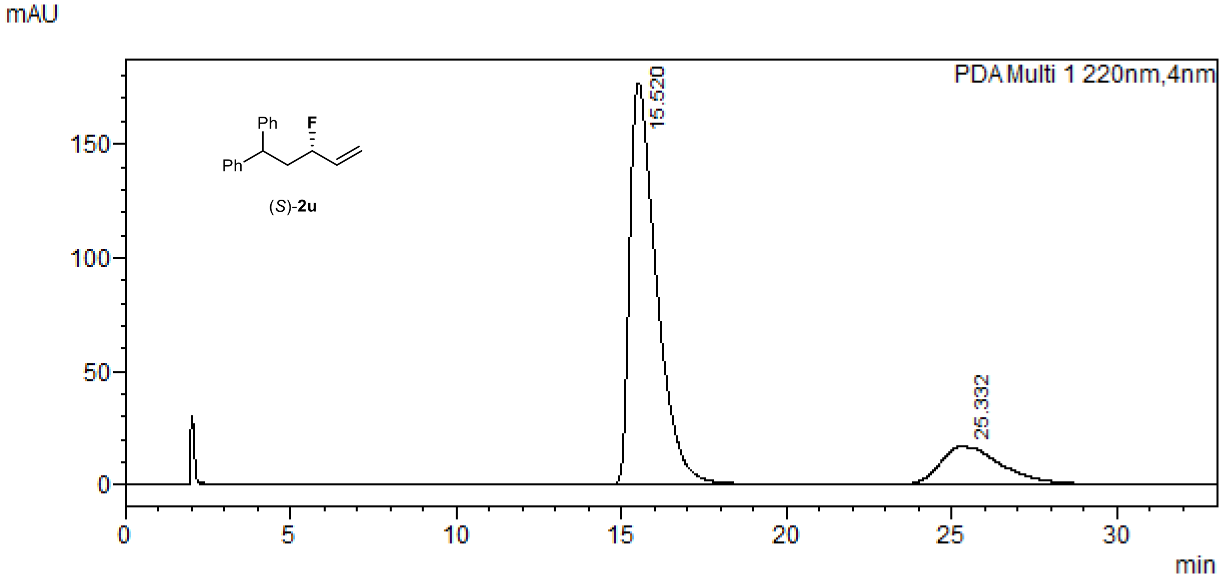

PDA Ch1 220nm

Peak#	Ret. Time	Area%
1	15.520	82.888
2	25.332	17.112
Total		100.000

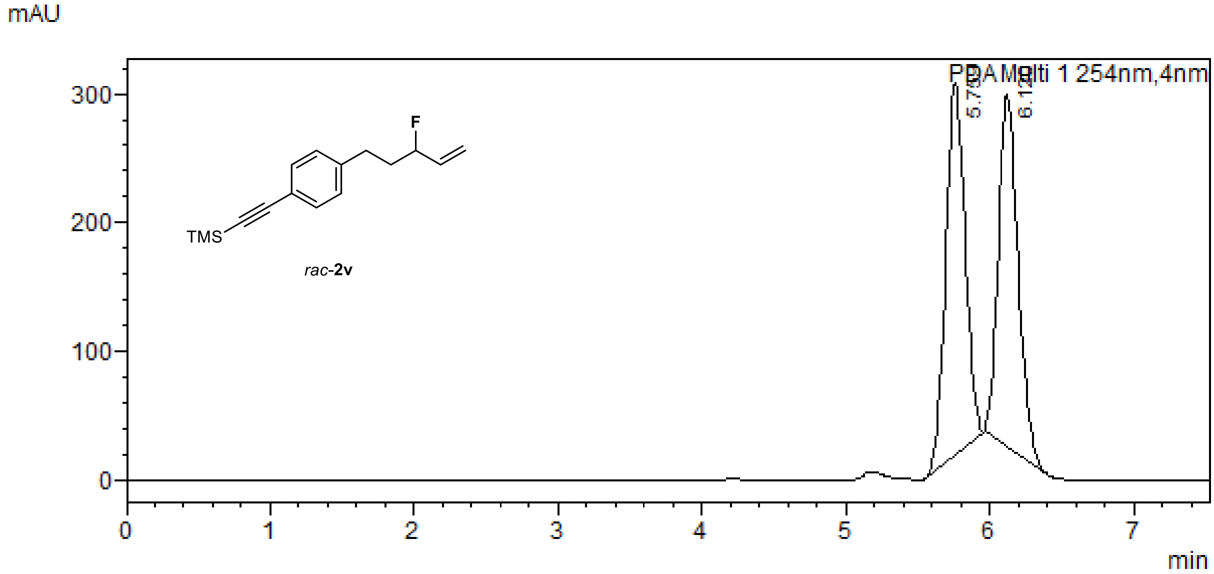

PDA Ch1 254nm

Peak#	Ret. Time	Area%
1	5.759	50.549
2	6.120	49.451
Total		100.000

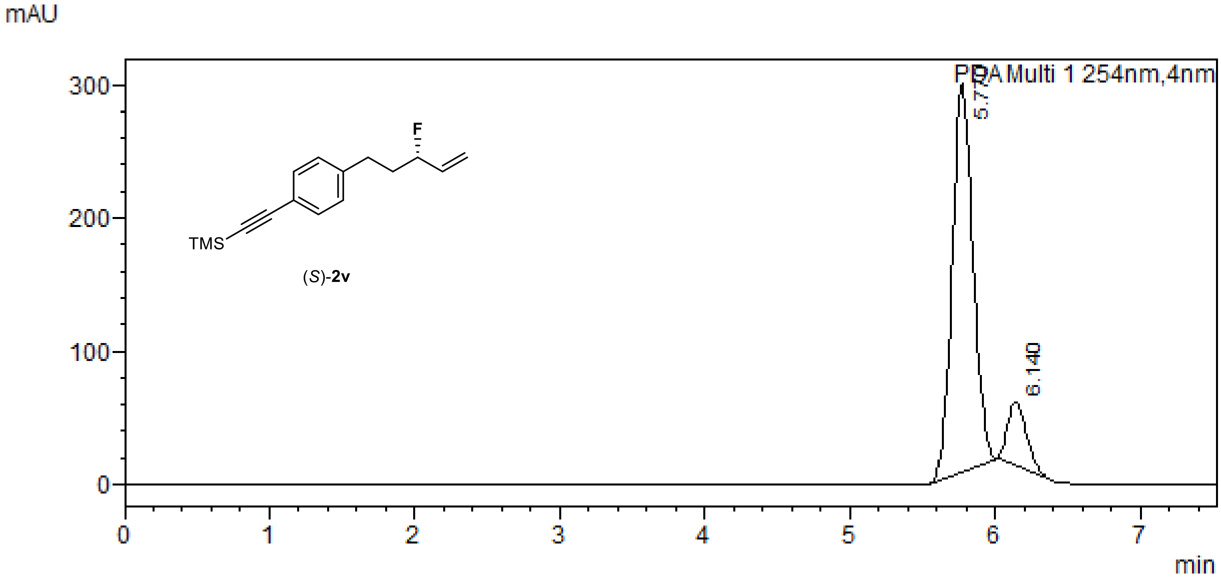

PDA Ch1 254nm

Peak#	Ret. Time	Area%
1	5.770	87.080
2	6.140	12.920
Total		100.000

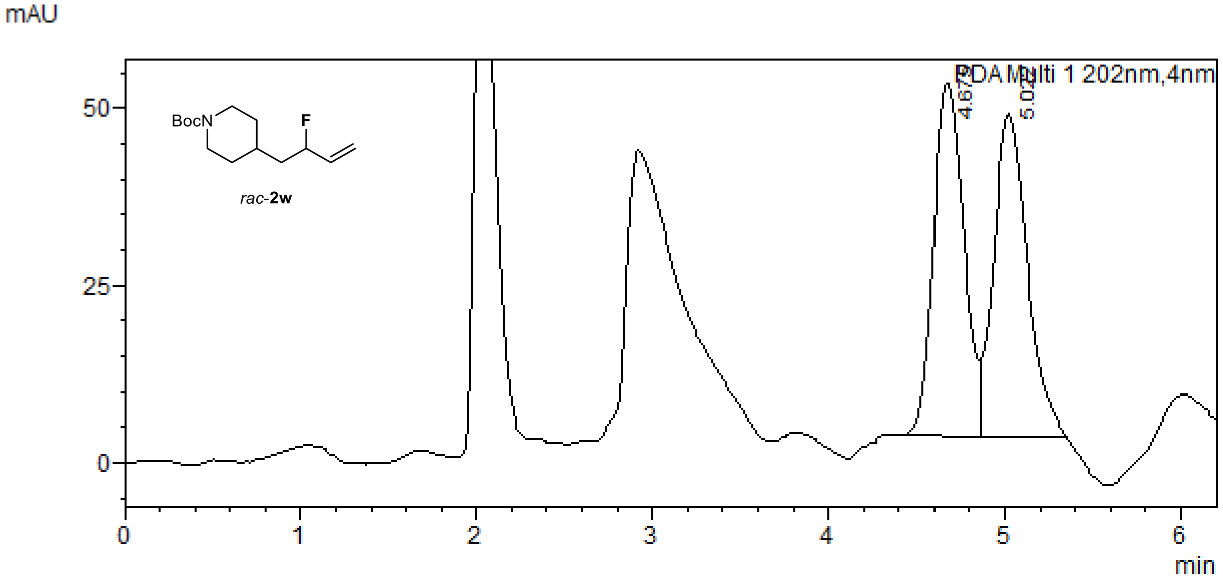

PDA Ch1 202nm

Peak#	Ret. Time	Area%
1	4.675	49.739
2	5.022	50.261
Total		100.000

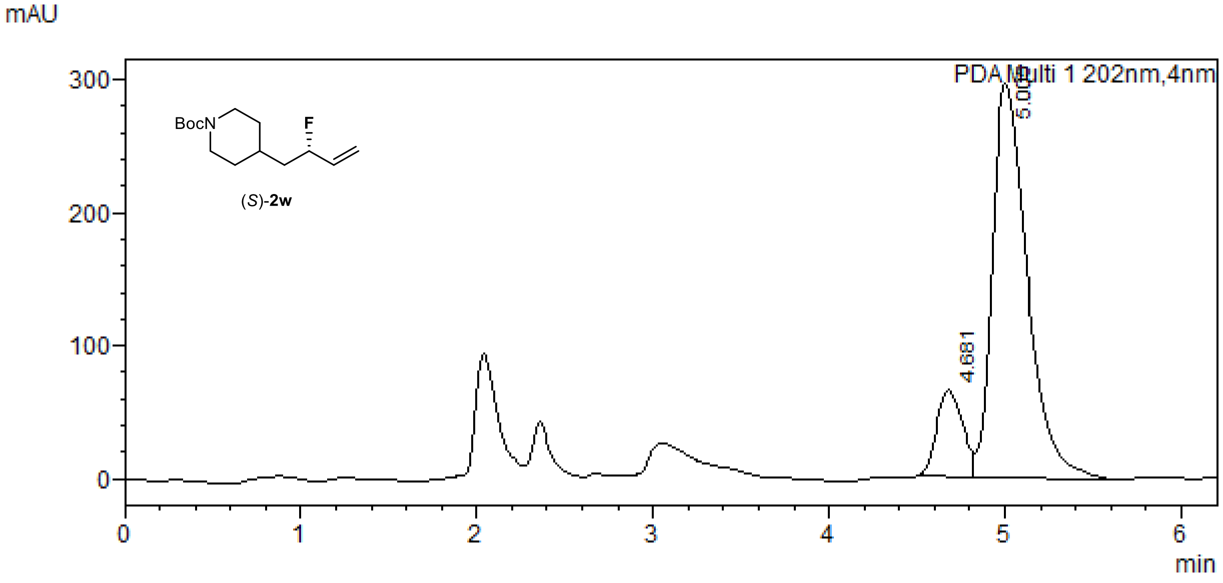

PDA Ch1 202nm

Peak#	Ret. Time	Area%
1	4.681	14.174
2	5.005	85.826
Total		100.000

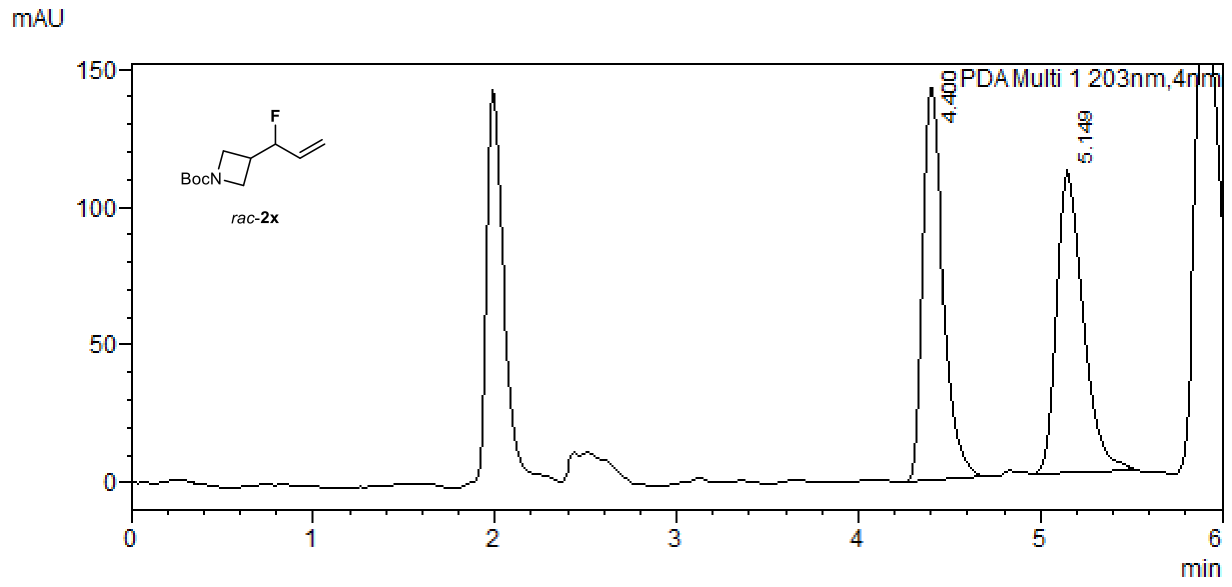

PDA Ch1 203nm

Peak#	Ret. Time	Area%
1	4.400	49.536
2	5.149	50.464
Total		100.000

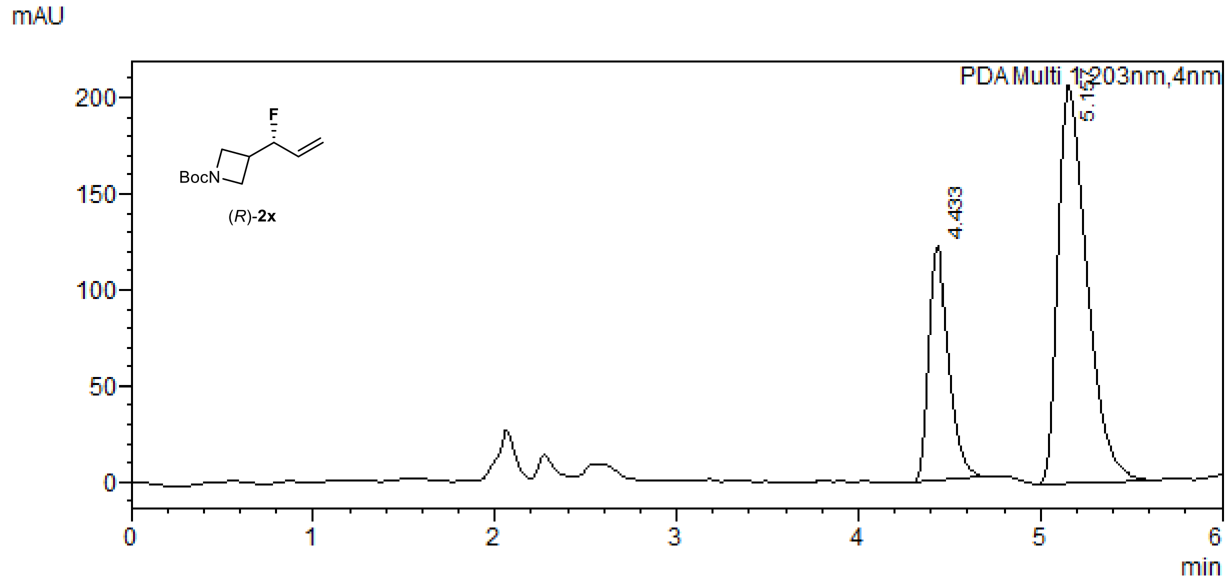

PDA Ch1 203nm

Peak#	Ret. Time	Area%
1	4.433	28.581
2	5.157	71.419
Total		100.000

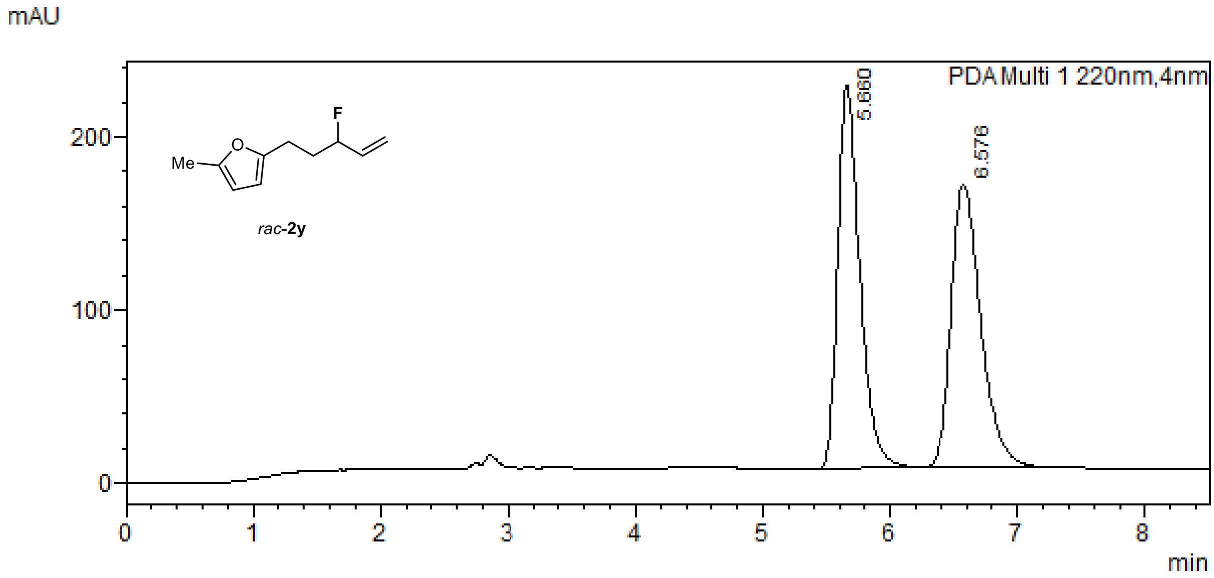

PDA Ch1 220nm

Peak#	Ret. Time	Area%
1	5.660	50.107
2	6.576	49.893
Total		100.000

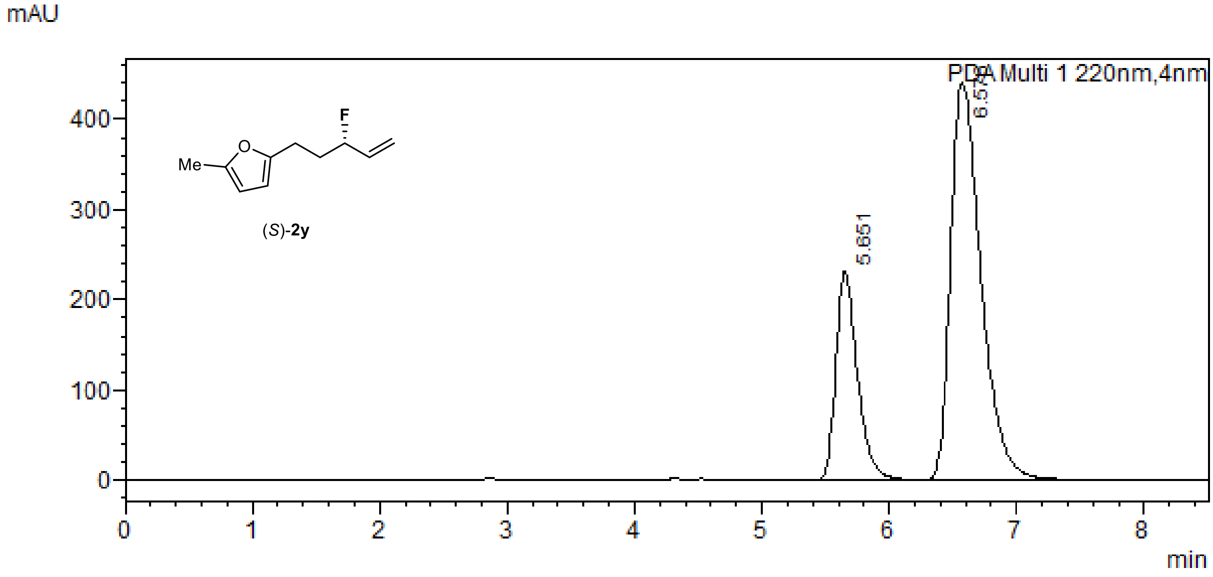

PDA Ch1 220nm

Peak#	Ret. Time	Area%
1	5.651	27.030
2	6.576	72.970
Total		100.000

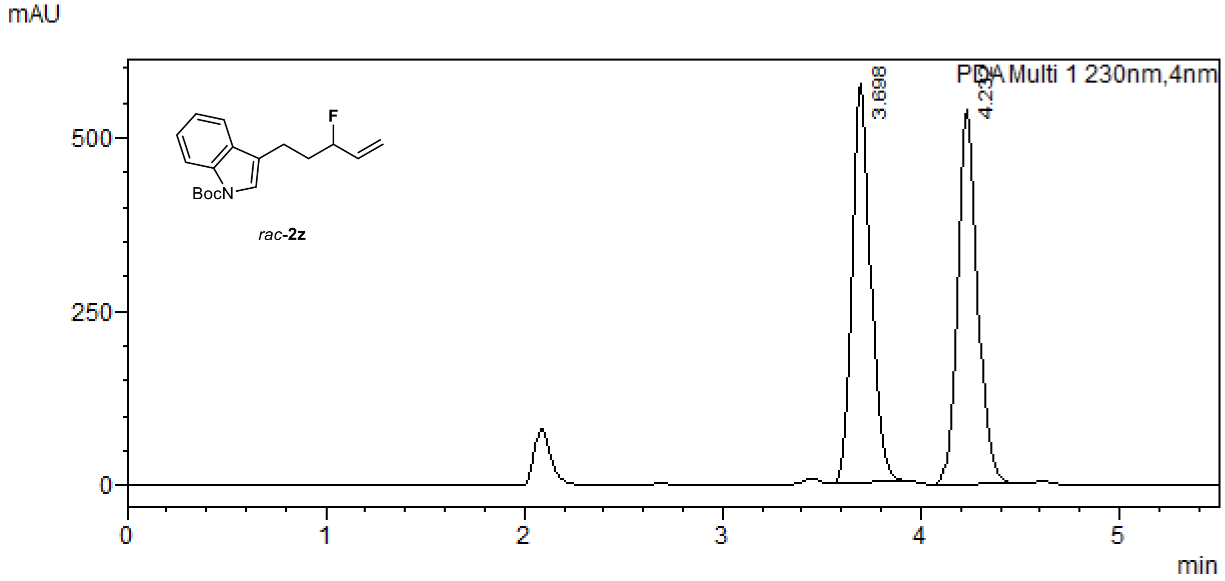

PDA Ch1 230nm

Peak#	Ret. Time	Area%
1	3.698	49.955
2	4.232	50.045
Total		100.000

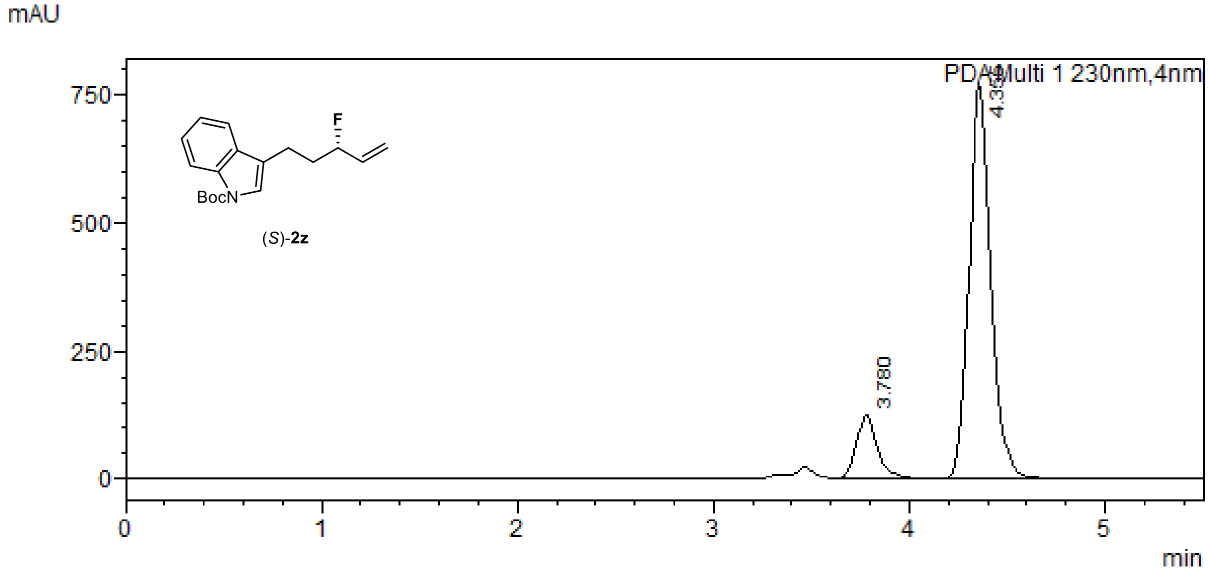

PDA Ch1 230nm

Peak#	Ret. Time	Area%
1	3.780	13.023
2	4.355	86.977
Total		100.000

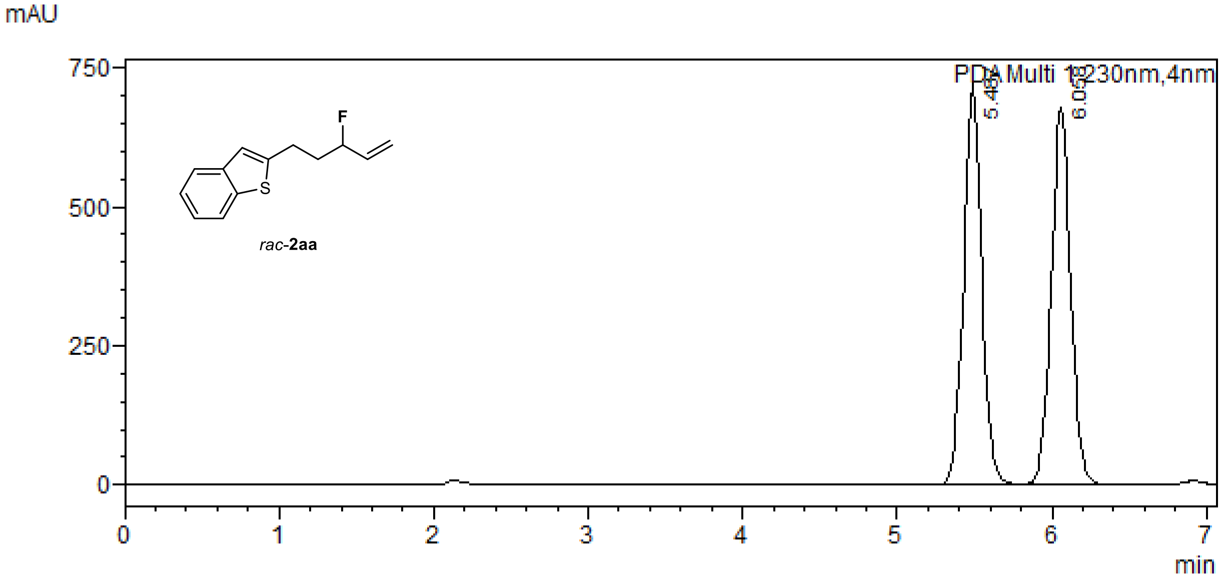

PDA Ch1 230nm

Peak#	Ret. Time	Area%
1	5.487	50.030
2	6.058	49.970
Total		100.000

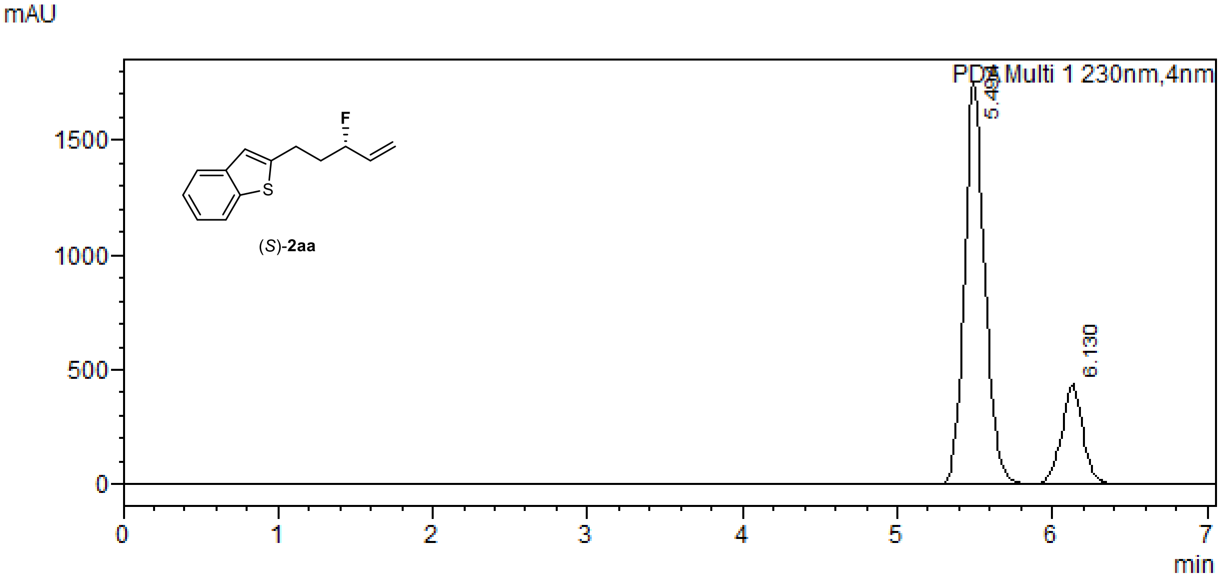

PDA Ch1 230nm

Peak#	Ret. Time	Area%
1	5.494	79.780
2	6.130	20.220
Total		100.000

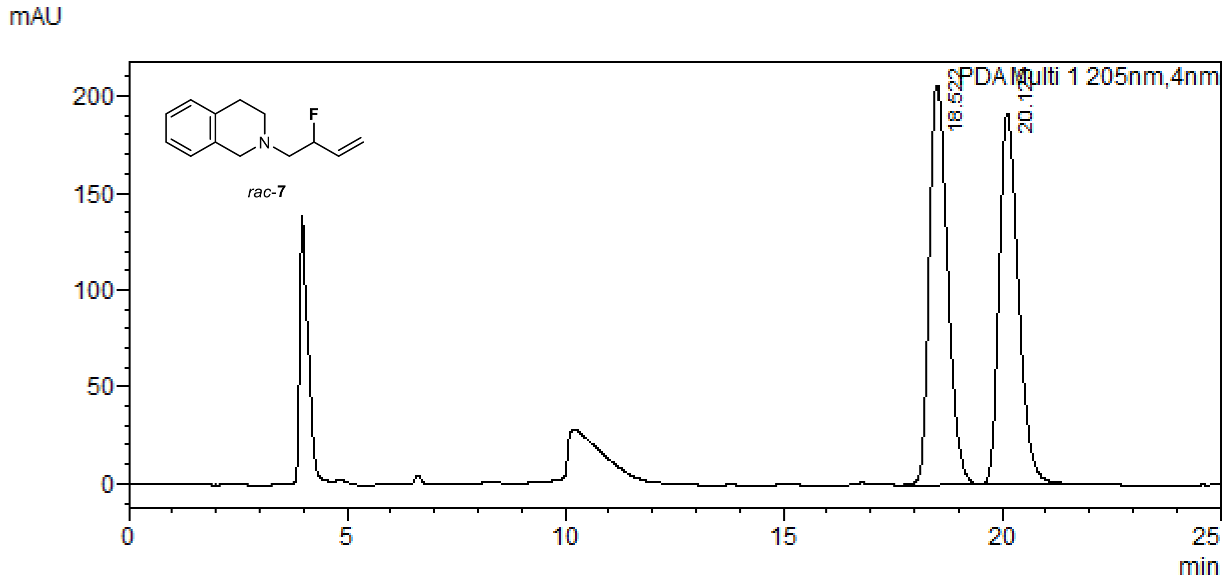

PDA Ch1 205nm

Peak#	Ret. Time	Area%
1	18.522	49.709
2	20.123	50.291
Total		100.000

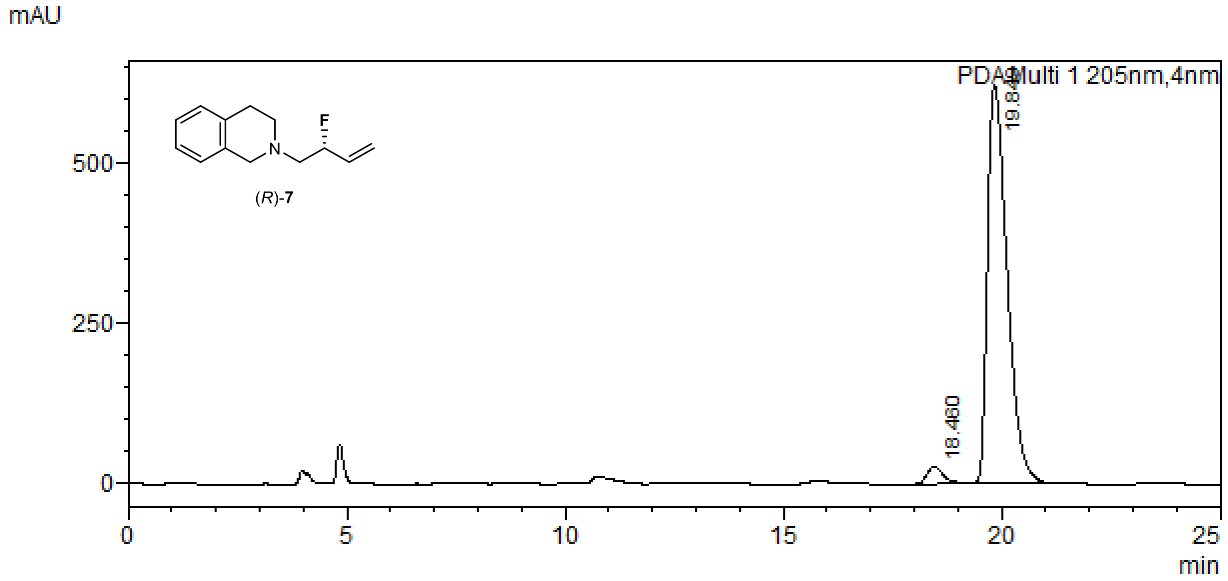

PDA Ch1 205nm

Peak#	Ret. Time	Area%
1	18.460	3.213
2	19.849	96.787
Total		100.000

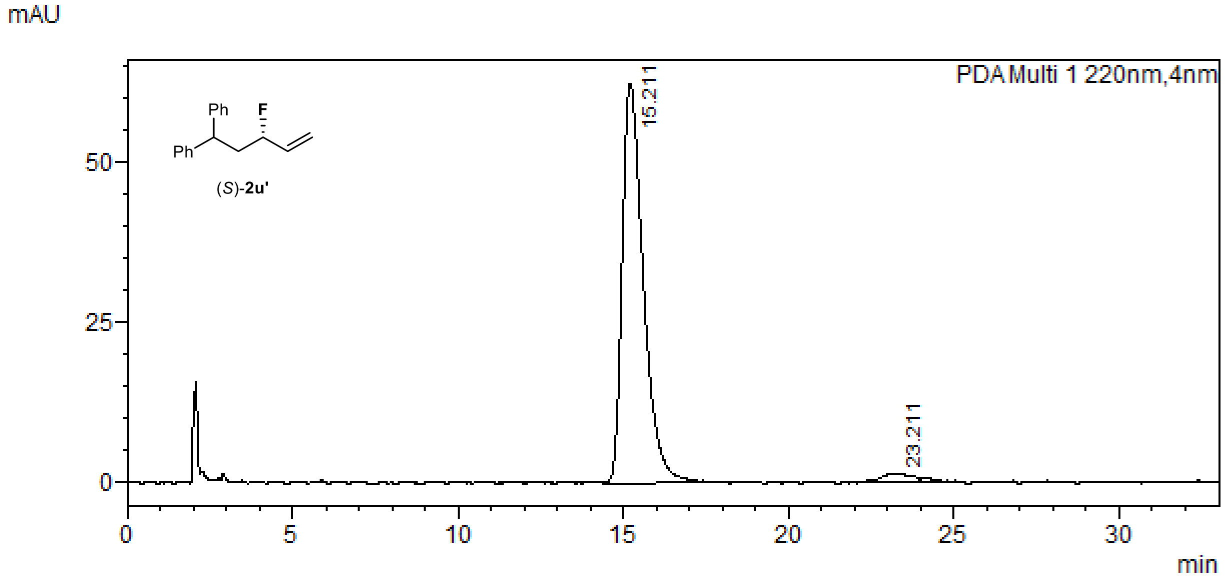

PDA Ch1 220nm

Peak#	Ret. Time	Area%
1	15.211	96.828
2	23.211	3.172
Total		100.000

The HPLC trace for *rac*-**2u** was reported above.

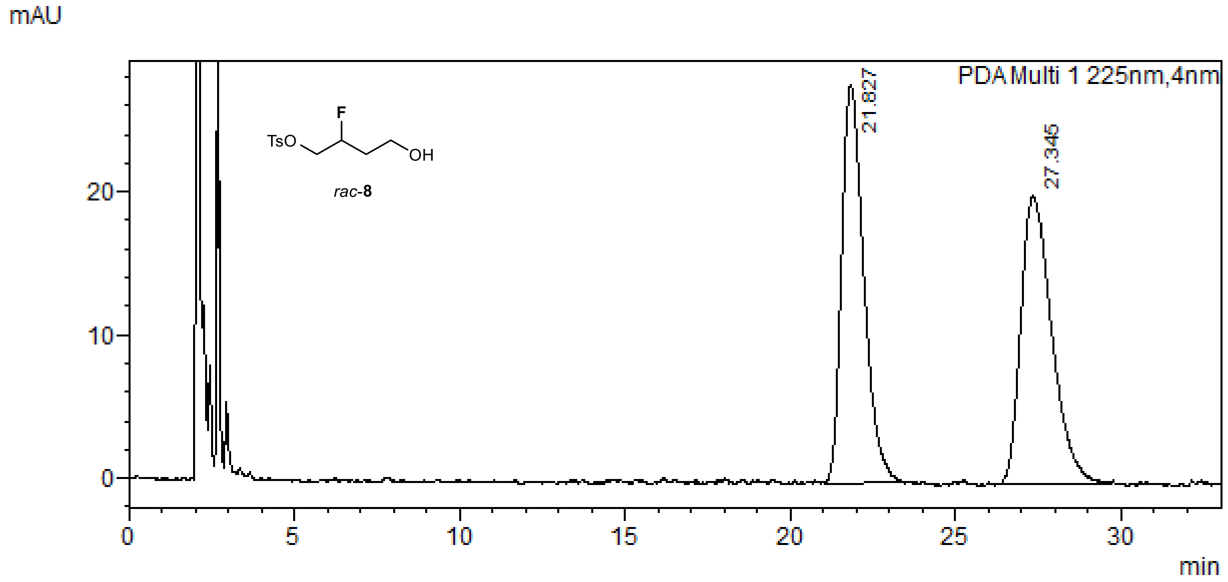

PDA Ch1 225nm

Peak#	Ret. Time	Area%
1	21.827	49.987
2	27.345	50.013
Total		100.000

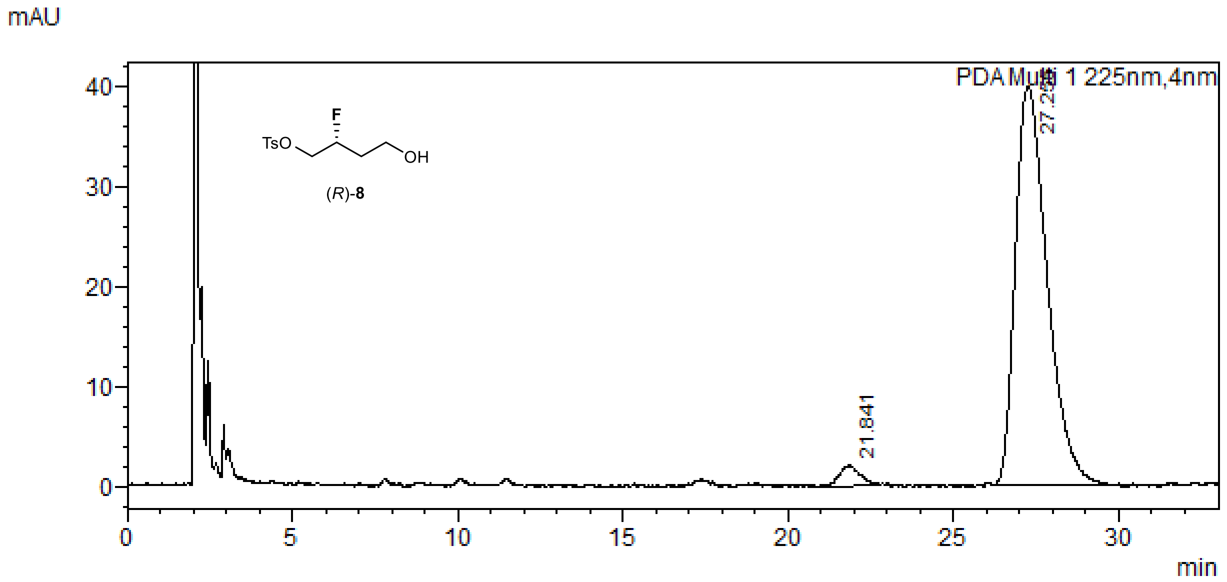

PDA Ch1 225nm

Peak#	Ret. Time	Area%
1	21.841	3.182
2	27.255	96.818
Total		100.000

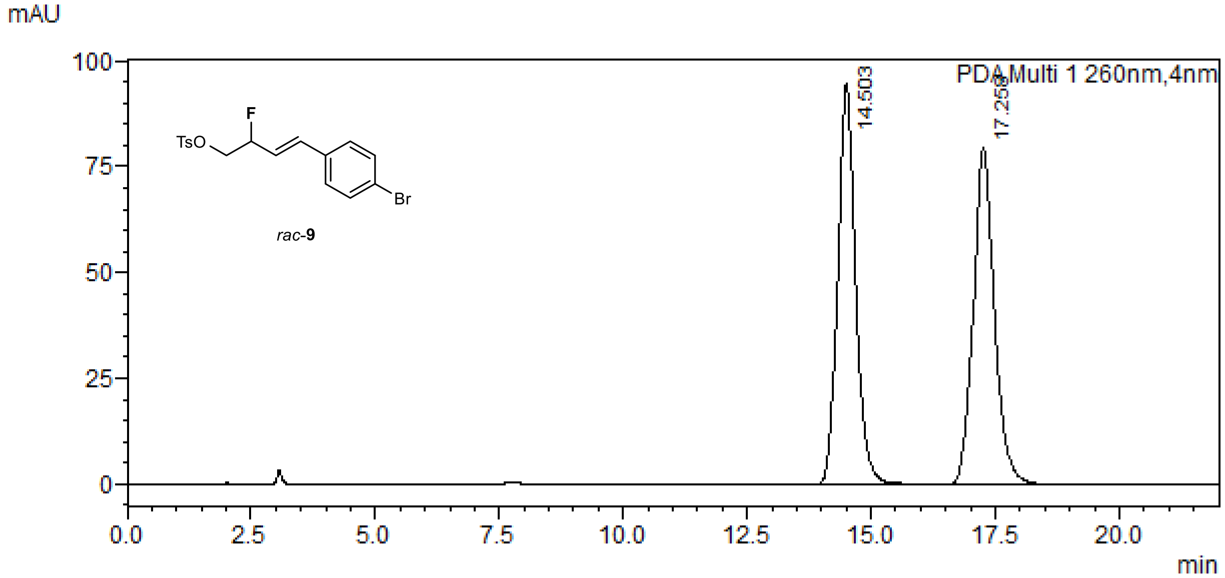

PDA Ch1 260nm

Peak#	Ret. Time	Area%
1	14.503	50.013
2	17.258	49.987
Total		100.000

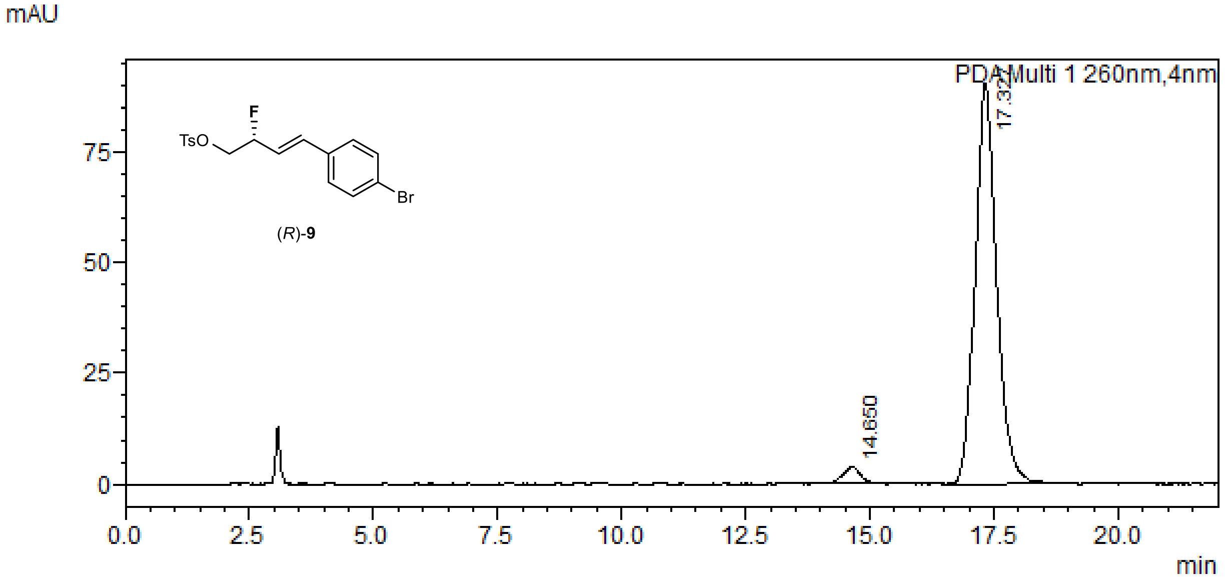

PDA Ch1 260nm

Peak#	Ret. Time	Area%
1	14.650	3.071
2	17.327	96.929
Total		100.000

References

(1) Pupo, G.; Ibba, F.; Ascough, D. M. H.; Vicini, A. C.; Ricci, P.; Christensen, K. E.; Pfeifer, L.; Morphy, J. R.; Brown, J. M.; Paton, R. S.; Gouverneur, V. Asymmetric Nucleophilic Fluorination under Hydrogen Bonding Phase-Transfer Catalysis. *Science* **2018**, *360* (6389), 638–642.

(2) Holakovský, R.; März, M.; Cibulka, R. Urea Derivatives Based on a 1,1′-Binaphthalene Skeleton as Chiral Solvating Agents for Sulfoxides. *Tetrahedron: Asymmetry* **2015**, *26* (23), 1328–1334.

(3) Topczewski, J. J.; Tewson, T. J.; Nguyen, H. M. Iridium-Catalyzed Allylic Fluorination of Trichloroacetimidates. *J. Am. Chem. Soc.* **2011**, *133* (48), 19318–19321.

(4) Pandit, S.; Adhikari, A. S.; Majumdar, N. Iridium-Catalyzed Enantioselective Ring Opening of Alkenyl Oxiranes by Unactivated Carboxylic Acids. *Org. Lett.* **2022**, *24* (40), 7388–7393.

(5) Zhang, L.; Zeng, W.; Xie, D.; Li, J.; Ma, X. Nickel and Chiral Phosphoric Acid Cocatalysis Enables Synthesis of C-Acyl Glycosides. *Org. Lett.* **2024**, *26* (7), 1332–1337.

(6) Batt, F.; Fache, F. Towards the Synthesis of the 4,19-Diol Derivative of (–)-Mycothiazole: Synthesis of a Potential Key Intermediate. *Eur. J. Org. Chem.* **2011**, *2011* (30), 6039–6055.

(7) Ikonnikova, V. A.; Zhigileva, E. A.; Al Mufti, A. M.; Solyev, P. N.; Baranov, M. S.; Mikhaylov, A. A. Merging Johnson-Claisen and Aromatic Claisen [3,3]-Sigmatropic Rearrangements: Ytterbium Triflate/2,6-Di-Tert-Butylpyridine Catalytic System. *J. Org. Chem.* **2023**, *88* (14), 9737–9749.

(8) Mukherjee, J. P.; Sil, S.; Pahari, A. K.; Chattopadhyay, S. K. A Modular Synthesis of Some Biologically Relevant Cyclic Peptides through Late-Stage Functionalization. *Synthesis* **2016**, *48* (08), 1181–1190.

(9) Fernandes, P. H.; Hackfeld, L. C.; Kozekov, I. D.; Hodge, R. P.; Lloyd, R. S. Synthesis and Mutagenesis of the Butadiene-Derived N3 2'-Deoxyuridine Adducts. *Chem. Res. Toxicol.* **2006**, *19* (7), 968–976.

(10) Gunes, Y.; Polat, M. F.; Sahin, E.; Fleming, F. F.; Altundas, R. Enantioselective Synthesis of Cyclic, Quaternary Oxonitriles. *J. Org. Chem.* **2010**, *75* (21), 7092–7098.

(11) Tsui, E.; Metrano, A. J.; Tsuchiya, Y.; Knowles, R. R. Catalytic Hydroetherification of Unactivated Alkenes Enabled by Proton-Coupled Electron Transfer. *Angew. Chem. Int. Ed.* **2020**, *59* (29), 11845–11849.

(12) Purushotham Reddy, S.; Chinnababu, B.; Shekhar, V.; Kumar Reddy, D.; Bhanuprakash, G. V.; Velatoor, L. R.; Venkateswara Rao, J.; Venkateswarlu, Y. Stereoselective Synthesis of Alpinoid-C and Its Analogues and Study of Their Cytotoxic Activity against Cancer Cell Lines. *Bioorg. Med. Chem. Lett.* **2012**, *22* (12), 4182–4184.

(13) Kouridaki, A.; Montagnon, T.; Tofi, M.; Vassilikogiannakis, G. Photooxidations of 2-(Gamma,Epsilon-Dihydroxyalkyl) Furans in Water: Synthesis of DE-Bicycles of the Pectenotoxins. *Org. Lett.* **2012**, *14* (9), 2374–2377.

(14) Kohyama, Y.; Murase, T.; Fujita, M. A Self-Assembled Cage as a Non-Covalent Protective Group: Regioselectivity Control in the Nucleophilic Substitution of Aryl-Substituted Allylic Chlorides. *Chem. Comm.* **2012**, *48* (63), 7811–7813.

(15) Hedhli, A.; Baklouti, A. Ouverture Des Vinyloxiranes Par Le Fluorhydrate De Triéthylamine Triacide. *J. Fluorine Chem.* **1995**, *70* (1), 141–144.

(16) Son, Y. W.; Kwon, T. H.; Lee, J. K.; Pae, A. N.; Lee, J. Y.; Cho, Y. S.; Min, S.-J. A Concise Synthesis of Tetrabenazine: An Intramolecular Aza-Prins-Type Cyclization via Oxidative C–H Activation. *Org. Lett.* **2011**, *13* (24), 6500–6503.

(17) Walborsky, H. M.; Murari, M. P. Electron Transfer Reactions from Alkali Metal Surfaces to (±) and (S)-(−)-1,3-Dimethoxy-1,1-Diphenylbutane. Studies on 1,3-Elimination. *Can. J. Chem.* **1984**, *62* (11), 2464–2470.

(18) Katcher, M. H.; Sha, A.; Doyle, A. G. Palladium-Catalyzed Regio- and Enantioselective Fluorination of Acyclic Allylic Halides. *J. Am. Chem. Soc.* **2011**, *133* (40), 15902–15905.

(19) Zhang, Q.; Stockdale, D. P.; Mixdorf, J. C.; Topczewski, J. J.; Nguyen, H. M. Iridium-Catalyzed Enantioselective Fluorination of Racemic, Secondary Allylic Trichloroacetimidates. *J. Am. Chem. Soc.* **2015**, *137* (37), 11912–11915.

(20) Enthart, A.; Freudenberger, J. C.; Furrer, J.; Kessler, H.; Luy, B. The CLIP/CLAP-HSQC: Pure Absorptive Spectra for the Measurement of One-Bond Couplings. *J. Magn. Reson.* **2008**, *192* (2), 314–322.

(21) Keske, E. C.; West, T. H.; Lloyd-Jones, G. C. Analysis of Autoinduction, Inhibition, and Autoinhibition in a Rh-Catalyzed C–C Cleavage: Mechanism of Decyanative Aryl Silylation. *ACS Catal.* **2018**, *8* (9), 8932–8940.

(22) Cosier, J.; Glazer, A. M. A Nitrogen-Gas-Stream Cryostat for General X-Ray Diffraction Studies. *J. Appl. Crystallogr.* **1986**, *19* (2), 105–107.

(23) Rigaku Oxford Diffraction. **2021**.

(24) Palatinus, L.; Chapuis, G. Superflip - a Computer Program for the Solution of Crystal Structures by Charge Flipping in Arbitrary Dimensions. *J. Appl. Crystallogr.* **2007**, *40* (4), 786–790.

(25) Betteridge, P. W.; Carruthers, J. R.; Cooper, R. I.; Prout, K.; Watkin, D. J. Crystals Version 12: Software for Guided Crystal Structure Analysis. *J. Appl. Crystallogr.* **2003**, *36* (6), 1487.

(26) Cooper, R. I.; Thompson, A. L.; Watkin, D. J. Crystals Enhancements: Dealing with Hydrogen Atoms in Refinement. *J. Appl. Crystallogr.* **2010**, *43*, 1100–1107.

(27) Frisch, M. J.; Trucks, G. W.; Schlegel, H. B.; Scuseria, G. E.; Robb, M. A.; Cheeseman, J. R.; Scalmani, G.; Barone, V.; Petersson, G. A.; Nakatsuji, H.; Li, X.; Caricato, M.; Marenich, A. V.; Bloino, J.; Janesko, B. G.; Gomperts, R.; Mennucci, B.; Hratchian, H. P.; Ortiz, J. V.; Izmaylov, A. F.; Sonnenberg, J. L.; Williams-Young, D.; Ding, F.; Lipparini, F.; Egidi, F.; Goings, J.; Peng, B.; Petrone, A.; Henderson, T.; Ranasinghe, D.; Zakrzewski, V. G.; Gao, J.; Rega, N.; Zheng, G.; Liang, W.; Hada, M.; Ehara, M.; Toyota, K.; Fukuda, R.; Hasegawa, J.; Ishida, M.; Nakajima, T.; Honda, Y.; Kitao, O.; Nakai, H.; Vreven, T.; Throssell, K.; Montgomery, J. A., Jr.; Peralta, J. E.; Ogliaro, F.; Bearpark, M. J.; Heyd, J. J.; Brothers, E. N.; Kudin, K. N.; Staroverov, V. N.; Keith, T. A.; Kobayashi, R.; Normand, J.; Raghavachari, K.; Rendell, A. P.; Burant, J. C.; Iyengar, S. S.; Tomasi, J.; Cossi, M.; Millam, J. M.; Klene, M.; Adamo, C.; Cammi, R.; Ochterski, J. W.; Martin, R. L.; Morokuma, K.; Farkas, O.; Foresman, J. B.; Fox, D. J.. *Gaussian 16*, Revision C.01; Gaussian, Inc., Wallingford CT, 2016.

(28) Zhao, Y.; Truhlar, D. G. A New Local Density Functional for Main-Group Thermochemistry, Transition Metal Bonding, Thermochemical Kinetics, and Noncovalent Interactions. *J. Chem. Phys.* **2006**, *125*, 194101.

(29) Weigend, F.; Ahlrichs, R. Balanced Basis Sets of Split Valence, Triple Zeta Valence and Quadruple Zeta Valence Quality for H to Rn: Design and Assessment of Accuracy. *Phys. Chem. Chem. Phys.* **2005**, *7* (18), 3297–3305.

(30) Grimme, S.; Antony, J.; Ehrlich, S.; Krieg, H. A Consistent and Accurate *ab initio* Parametrization of Density Functional Dispersion Correction (DFT-D) for the 94 Elements H-Pu. *J. Chem. Phys.* **2010**, *132* (15), 154104.

(31) (a) Fuentealba, P.; Preuss, H.; Stoll, H.; Von Szentpály, L. A Proper Account of Core-Polarization with Pseudopotentials: Single Valence-Electron Alkali Compounds. *Chem. Phys. Lett.* **1982**, *89* (5), 418–422; (b) Andrae, D.; Häußermann, U.; Dolg, M.; Stoll, H.; Preuß, H. Energy-Adjusted *ab initio* Pseudopotentials for the Second and Third Row Transition Elements. *Theor. Chim. Acta* **1990**, *77* (2), 123–141.

(32) Cossi, M.; Rega, N.; Scalmani, G.; Barone, V. Energies, Structures, and Electronic Properties of Molecules in Solution with the C-PCM Solvation Model. *J. Comput. Chem.* **2003**, *24* (6), 669–681.

(33) (a) Neese, F. The ORCA Program System. *WIREs Comput. Mol. Sci.* **2012**, *2* (1), 73–78; (b) Neese, F. Software Update: The ORCA Program System − Version 5.0. *WIREs Comput. Mol. Sci.* **2022**, *12* (5), e1606.

(34) Chai, J.-D.; Head-Gordon, M. Long-Range Corrected Hybrid Density Functionals with Damped Atom–Atom Dispersion Corrections. *Phys. Chem. Chem. Phys.* **2008**, *10* (44), 6615–6620.

(35) Metz, B.; Stoll, H.; Dolg, M. Small-Core Multiconfiguration-Dirac–Hartree–Fock-Adjusted Pseudopotentials for Post-d Main Group Elements: Application to PbH and PbO. *J. Chem. Phys.* **2000**, *113* (7), 2563–2569.

(36) Luchini, G.; Alegre-Requena, J.; Funes-Ardoiz, I.; Paton, R. GoodVibes: Automated Thermochemistry for Heterogeneous Computational Chemistry Data. *F1000Research* **2020**, *9*, 291.

(37) Grimme, S. Supramolecular Binding Thermodynamics by Dispersion-Corrected Density Functional Theory. *Chem. Eur. J.* **2012**, *18* (32), 9955–9964.

(38) Boto, R. A.; Peccati, F.; Laplaza, R.; Quan, C.; Carbone, A.; Piquemal, J.-P.; Maday, Y.; Contreras-García, J. NCIPLOT4: Fast, Robust, and Quantitative Analysis of Noncovalent Interactions. *J. Chem. Theory Comput.* **2020**, *16* (7), 4150–4158.

(39) Glendening, E. D.; Landis, C. R.; Weinhold, F. NBO 7.0: New Vistas in Localized and Delocalized Chemical Bonding Theory. *J. Comput. Chem.* **2019**, *40* (25), 2234–2241.

(40) de Souza, B. GOAT: A Global Optimization Algorithm for Molecules and Atomic Clusters. *Angew. Chem. Int. Ed.* **2025**, *64* (18), e202500393.

(41) Bannwarth, C.; Ehlert, S.; Grimme, S. GFN2-xTB—An Accurate and Broadly Parametrized Self-Consistent Tight-Binding Quantum Chemical Method with Multipole Electrostatics and Density-Dependent Dispersion Contributions. *J. Chem. Theory Comput.* **2019**, *15* (3), 1652–1671.

(42) Stahn, M.; Ehlert, S.; Grimme, S. Extended Conductor-like Polarizable Continuum Solvation Model (CPCM-X) for Semiempirical Methods. *J. Phys. Chem. A* **2023**, *127* (33), 7036–7043.

(43) *CRC Handbook of Chemistry and Physics, 97th ed*.; Haynes, W. M., Ed.; CRC Press, 2016. DOI: 10.1201/9781315380476

(44) Marcus, Y.; Loewenschuss, A. Chapter 4. Standard Entropies of Hydration of Ions. *Annu. Rep. Prog. Chem., Sect. C: Phys. Chem.* **1984**, *81*, 81–135.
